# LC-MS/MS-Based Quantitative Proteomics Analysis of Different Stages of Non-Small-Cell Lung Cancer

**DOI:** 10.1155/2021/5561569

**Published:** 2021-02-26

**Authors:** Murong Zhou, Yi Kong, Xiaobin Wang, Wen Li, Si Chen, Li Wang, Chengbin Wang, Qian Zhang

**Affiliations:** ^1^College of Physics and Optoelectronic Engineering, Shenzhen University, Shenzhen, China; ^2^Medical School of Chinese PLA & Medical Laboratory Center, First Medical Center of Chinese PLA General Hospital, Beijing, China; ^3^Shenzhen University Health Science Center, Shenzhen, China; ^4^Department of Immunology, Shenzhen University Health Science Center, Shenzhen, China; ^5^Department of Dermatology, Shenzhen University General Hospital, Shenzhen University, Shenzhen, China; ^6^Department of Dermatology, Huazhong University of Science and Technology Union Shenzhen Hospital, Shenzhen, China

## Abstract

Lung cancer has a higher incidence rate and mortality rate than all other cancers. Early diagnosis and treatment of lung cancer remain a major challenge, and the 5-year survival rate of its patients is only 15%. Basic and clinical research, especially the discovery of biomarkers, is crucial for improving the diagnosis and treatment of lung cancer patients. To identify novel biomarkers for lung cancer, we used the iTRAQ8-plex labeling technology combined with liquid chromatography-tandem mass spectrometry (LC-MS/MS) to analyze the serum and urine of patients with different stages of lung adenocarcinoma and healthy individuals. A total of 441 proteins were identified in the serum, and 1,161 proteins were identified in the urine. The levels of elongation factor 1-alpha 2, proteasome subunit alpha type, and spermatogenesis-associated protein increased significantly in the serum of patients with lung cancer compared with those in healthy controls. The levels of transmembrane protein 143, cadherin 5, fibronectin 1, and collectin-11 decreased significantly in the serum of patients with metastases compared with those of nonmetastatic lung cancer patients. In the urine of stage III and IV lung cancer patients, the prostate-specific antigen and prostatic acid phosphatase decreased significantly, whereas neutrophil defensin 1 increased significantly. The results of LC-MS/MS were confirmed by enzyme-linked immunosorbent assay (ELISA) for transmembrane protein 143, cadherin 5, fibronectin 1, and collectin-11 in the serum. These proteins may be a potential early diagnosis and metastasis biomarkers for lung adenocarcinoma. Furthermore, the relative content of these markers in the serum and urine could be used to determine the progression of lung adenocarcinoma and achieve accurate staging and diagnosis.

## 1. Introduction

The incidence and mortality rates of lung cancer are higher than those of other cancers [1]. The overall 5-year survival rate for lung cancer is only 15% [2]. Lung cancer is divided into small cell carcinoma (SCLC) and non-small-cell carcinoma (NSCLC). SCLC accounts for 10%-15% of lung cancers and is sensitive to radiotherapy and chemotherapy [3, 4]. However, approximately 85% of lung cancers are NSCLC [5]. The median survival time of patients with advanced lung cancer is only 10 months [6]. Although the diagnosis and treatment of lung cancer have significantly improved, the current treatment methods are still not satisfactory. Improved early diagnosis and targeted treatment of lung cancer are required in clinical practices to improve patient outcomes.

Proteomics is the science of studying protein composition and alterations in cells, tissues, and organisms. Proteomics is widely used in basic and clinical medical research [7] for the identification of biomarkers [8, 9], posttranslational protein modifications [10], and the regulation of signaling pathways [11]. Proteomics research of lung cancer has focused on the classification of lung cancer [12], the correlation between protein and gene expressions [13, 14], identification of new molecular targets [15, 16], and the development of new drugs [17, 18]. Early diagnostic markers for lung cancer [19, 20] have been identified, but the markers still lack accuracy and sensitivity. Therefore, exploration and discovery of reliable and sensitive markers for the early diagnosis of lung cancer are a research priority.

Quantitative proteomics can determine relative changes in protein content. Based on this technology, differences in protein abundance between healthy individuals and patients with cancer can be defined to identify disease markers. Proteomics has been widely used in human disease research [21]. The postlabeling analysis of proteins using the iTRAQ8-plex technology, combined with Data Dependent Acquisition (DADA), is currently the standard labeling method used in quantitative proteomics [18, 22, 23]. The iTRAQ8-plex technology is also a commonly used quantitative method, which can be applied to quantitative analysis in proteomics research [24, 25].

For the first time, we combined iTRAQ8-plex labeling with liquid chromatography-tandem mass spectrometry (LC-MS/MS) as a quantitative proteomics analysis approach to compare protein abundance in the serum and urine samples of healthy controls to that in the serum and urine samples of stage I, II, III, and IV lung adenocarcinoma patients. The purpose of this study was to identify a set of biomarkers for the early diagnosis and metastasis prediction in patients with lung adenocarcinoma using serum and urine.

## 2. Materials and Methods

### 2.1. Chemicals and Reagents

Pierce™ Top12 Abundant Protein Depletion Spin Columns, dithiothreitol (DTT), indole-3-acetic acid (IAA), a bicinchoninic acid (BCA) kit, iTRAQ reagents, and Ziptip solid-phase microextraction reagents were purchased from Thermo Fisher Scientific, Inc. (Waltham, MA, USA). Trypsin was obtained from Promega. All organic reagents were high-performance liquid chromatography (HPLC) grade, and Milli-Q ultrapure water was used in all experiments (Millipore, Bradford, USA). Other reagents were analytical grade reagents, unless otherwise indicated.

### 2.2. Collection and Storage of Serum and Urine Samples

Serum and urine samples were collected from patients that were diagnosed with lung adenocarcinoma at the General Hospital of the Chinese People's Liberation Army from June 2017 to June 2018. The discovery set consisted of 30 healthy individuals and 70 lung adenocarcinoma patients at early stages (stage Ia1, *n* = 10; stage Ia2, *n* = 10; stage Ia3, *n* = 10; stage Ib, *n* = 10; and stage II, *n* = 10) and late stages (stage III, *n* = 10; stage IV, *n* = 10). Healthy controls were age- and gender-matched to the lung adenocarcinoma patients (Table 1). Of note, serum and urine samples were collected before the 70 lung adenocarcinoma patients had undergone chemical or medical treatment. All samples were collected from patients with an empty stomach in the morning. All participants provided signed informed consent, and samples were collected following the protocol approval. All methods were carried out in accordance with the approved guidelines, and all experimental protocols were approved by the ethics committee of the Chinese PLA General Hospital (Number S2018-007-001). Each serum sample was allowed to clot for 45 min and then centrifuged at 2,000 rpm for 10 min. For urine samples, a morning midstream urine specimen was collected and centrifuged at 1,500 rpm for 5 min. All samples were aliquoted and stored at −80°C until use.

### 2.3. Sample Preparation and Enzyme Digestion

For the LC-MS analysis, frozen serum and urine samples were thawed on ice. Serum samples with each group (100 *μ*L of serum) were merged; the urine samples within each group (2 mL of urine) were merged. Pierce™ Top12 Abundant Protein Depletion Spin Columns were used to remove high-abundance proteins from the serum samples of each group. The filtrate was collected and denatured with 8 M urea. DTT and IAA were added to reduce alkylation, and 1 *μ*g of Trypsin was then added at a ratio of 1 : 30 (enzyme : protein). The sample was hydrolyzed overnight at 37°C. Urine samples were precipitated with precooled acetone, and urea was used to redissolve the precipitate, followed by protein quantification using a BCA kit. Lastly, 3.3 *μ*g Trypsin was added to 100 *μ*g protein from each group, and the samples were enzymatically hydrolyzed (1 : 30, enzyme : protein).

### 2.4. Use of the iTRAQ-8plex for Labeling and Separation

After digestion, iTRAQ113-119 was used to separately label the serum and urine of stage Ia1-IV, and iTRAQ121 was used to label the serum and urine of the normal control group. For iTRAQ-8plex labeling, 150 *μ*L isopropanol was added to each labeling reagent, and the mixture was added to each polypeptide sample (100 *μ*g) after shaking and mixing. The reaction was carried out at room temperature for 2 hours. Then, water (100 *μ*L) was added to terminate the reaction, and samples were freeze-dried after mixing.

The labeled peptide fragments were mixed, and 15 components were separated using the Agilent 1200 HPLC separation system. The chromatographic column was a high-pH RP C18 (4.6 mm × 250 mm, 5 *μ*m, 300 A). Mobile phase A was the aqueous phase containing 20 mM ammonium acetate (pH 10). Mobile phase B was ACN/water containing 20 mM ammonium acetate (ACN/Water, 9/1, *v*/*v*, pH 10). The mobile phase gradient was 5% B to 35% B and 35%–40% B to 90% B; 90% B was then maintained for 10 minutes. The fractionated samples were desalinated using Ziptip solid-phase microextraction and analyzed.

### 2.5. SDS-PAGE

Serum samples were loaded onto a 10% sodium dodecyl sulfate- (SDS-) polyacrylamide gel electrophoresis (SDS-PAGE gel) (Invitrogen™, Thermo Fisher Scientific, Inc., New York, USA) and run at 100 V for 100 min in a running buffer. A prestained protein standard (Solarbio Science & Technology Co., Ltd., Beijing, China) was used to track protein migration. The resulting gels were stained with a fast silver stain kit (Beyotime, Beijing, China). The protocol was previously described [26].

### 2.6. LC-MS/MS Analysis

The prepared samples were separated and identified by liquid chromatography- (Ultimate3000, Thermo Fisher Scientific, Inc.) tandem mass spectrometry (Q-Exactive, Thermo Fisher Scientific, Inc.). Samples were first loaded separately on a trap column (150 *μ*m × 20 mm) packed with SP-300-ODS-AP (3 *μ*m particle diameter; 100 nm pore size in house). Each sample was eluted into an analytical column (75 *μ*m × 15 mm), packed with SP-300-ODS-AP, and separated at a flow rate of 500 nL∙min^−1^ with an elution gradient consisting of mobile phase B (80% acetonitrile, 20% H_2_O, 0.1% formic acid) and mobile phase A (99.9% H_2_O, 0.1% formic acid). Elution gradient solutions were added as follows: B was increased from 4% to 10% in 5 minutes, from 10% to 12.5% in 10 minutes, from 12.5% to 27.5% in 75 minutes, from 27.5% to 50% in 110 minutes, and from 50% to 95% in 10 minutes. The LC system was automatically equilibrated with mobile phase A for approximately 10 minutes before the next analysis. Fractions were continuously detected in the Q-Exactive hybrid quadrupole-orbitrap mass spectrometer with a nanoelectrospray ionization source at a capillary temperature of 250°C and a spray voltage of 2,500 V.

### 2.7. Enzyme-Linked Immunosorbent Assay (ELISA) Analysis

The 100 serum samples in the experiment were analyzed using an ELISA for transmembrane protein 143, cadherin 5, fibronectin 1, and collectin-11 according to the manufacturer's instructions.

### 2.8. Data Analysis

The MS/MS spectra were searched using the Proteome Discoverer 2.2 against a nonredundant Sequest database (released in January 2010; Homo sapiens, 20,367 entries). For protein identification, we set thresholds of 10 ppm for intact peptide tolerance masses and 0.02 Da for fragment ions. The analysis allowed for two missed cleavages from the trypsin digest, and iTRAQ (N-terminal, 144 Da), iTRAQ (Lys, 144 Da), oxidized methionine (16 Da), and carbamidomethyl (C, 57 Da) were set as potential variable modifications. Each peptide integrated intensity was normalized to the sum of its channel intensities. The normalized channels were averaged over all peptides of a protein, and the standard deviation of the mean was determined for each normalized channel of a peptide. The results of the SEQUEST database search for each reversed-phase elution were further analyzed (Table S1, serum; Table S2, urine). A difference multiple greater than 1.3 or less than 0.77 in serum and that greater than 1.5 or less than 0.67 in urine were considered statistically significant. The clinical characteristics were compared using Student's *t* test, Fisher's exact test, or Wilcoxon rank test, whichever was appropriate. The concentrations of transmembrane protein 143, cadherin 5, fibronectin 1, and collectin-11 in the different stages of lung cancer were compared to those of the control group using Student's *t* test. Data are expressed as the mean ± standard deviation (SD). Results were analyzed using SPSS 8.0 software and Origin 8.5 statistics. The analysis of variance was performed to determine any significant differences (*P* ≤ 0.05).

## 3. Results

### 3.1. Identification of Removed High-Abundance Proteins

**Figure 1:**
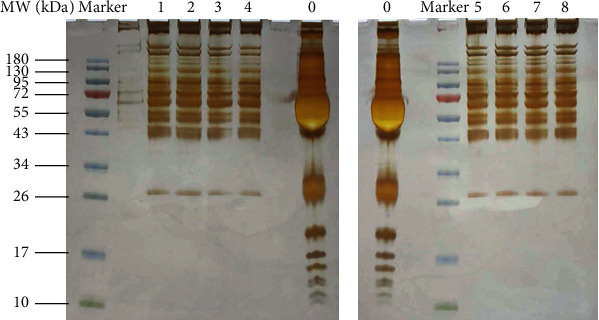
SDS-PAGE electrophoresis of removal high-abundance proteins. 0: nothing was done; 1: healthy control; 2: stage Ia1; 3: stage Ia2; 4: stage Ia; 5: stage Ib; 6: stage II; 7: stage III; 8: stage IV.

We used Pierce™ Top 12 Abundant Protein Depletion Spin Columns to remove high-abundance proteins from each serum group, as indicated by SDS-PAGE electrophoresis (Figure 1). After the removal of high-abundance proteins, the distribution of protein bands was significantly better than that of samples without the removal of high-abundance proteins. The eight samples separated well after removal of high-abundance proteins; the bands were similar, and the color depth was consistent among the samples.

### 3.2. Identification of Polypeptides in Serum and Urine Samples

We examined the iTRAQ labeling efficiency. A total of 12,155 peptides were identified in the urine sample, and 12,153 peptides were labeled using the iTRAQ reagent. Thus, the labeling efficiency of iTRAQ was 99.9%. The labeling efficiency of the serum sample reached 99.86%.

**Figure 2:**
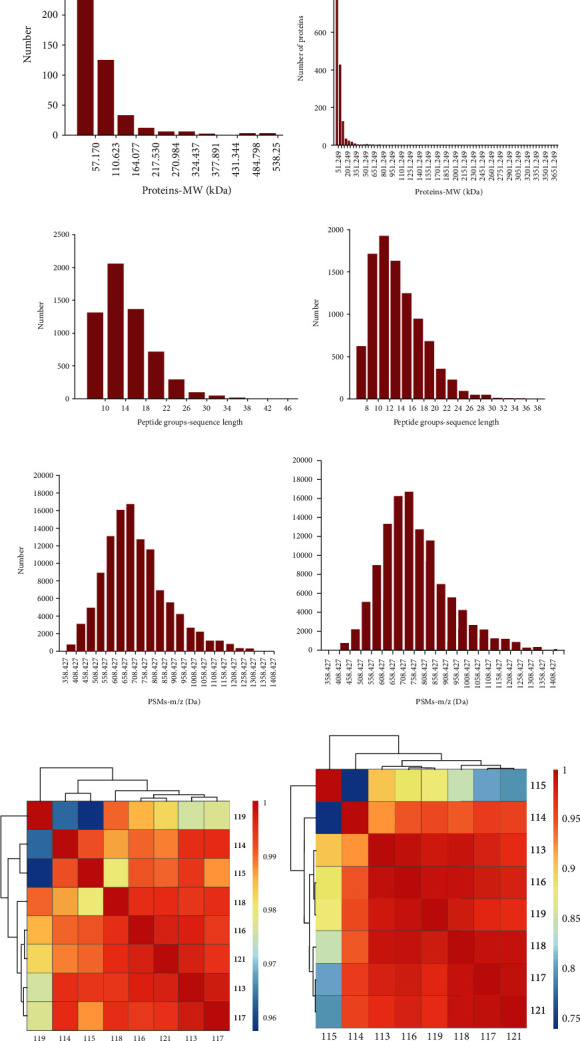
(a) Molecular weight distribution of serum. (b) Molecular weight distribution of urine. (c) The number of peptides of different lengths in the serum specimen. (d) The number of peptides of different lengths in the urine specimen. (e) The distribution of PSMs *m*/*z* in the serum specimen. (f) The distribution of PSMs *m*/*z* in the urine specimen. (g) The diagram of correlation coefficient array in the serum specimen. (h) The diagram of correlation coefficient array in the urine specimen.

According to the LC-MS analysis (Table S1, serum; Table S2, urine), the relative molecular mass distribution of proteins was mainly concentrated below 200 kDa in the serum and urine samples, because the experiment used the classic bottom-up technique (Figures 2(a) and 2(b)). Proteins with molecular weights of up to 540 kDa were identified. Peptide sequence lengths ranged from 10 to 13 peaks; polypeptide lengths were concentrated in the 6–25 range, and 90% of peptide lengths were within 24 kDa, as expected (Figures 2(c) and 2(d)). The theoretical distribution was fitted to a sixth degree polynomial, and *R*^2^ was greater than 0.95. On the polypeptide *m*/*z* distribution map, the abscissa is *m*/*z*, and the ordinate is the number of polypeptides. Most of the polypeptides had an *m*/*z* of 400–1,200, and as *m*/*z* gradually increased, the number of identified polypeptides gradually decreased, as expected (Figures 2(e) and 2(f)). In the final correlation analysis, the normalized protein abundance values were used to analyze the correlation between the samples. Figures 2(g) and 2(h) are the correlation map and correlation coefficient matrix analysis of serum and urine samples. Correlation between the samples was very high, and the correlation coefficient was close to 1.

### 3.3. Identification of Differentially Expressed Proteins in the Urine

**Figure 3:**
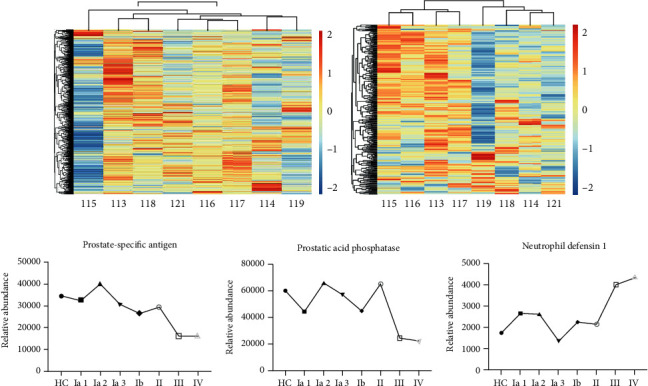
Statistical analysis of differentially expressed proteins and urine protein markers with significant differences between early and late stage lung adenocarcinomas. (a) The heat map of changed in urine proteins. (b) The heat map of changed in serum proteins. (c) Prostate-specific antigen expression in lung adenocarcinoma groups was significantly lower in the metastatic groups than in the non-metastasis groups. (d) Prostatic acid phosphatase expression of the metastatic groups was significantly lower than that of the nonmetastatic groups in lung adenocarcinoma. (e) The expression of neutrophil defensin in the lung adenocarcinoma groups was significantly higher than that in the metastatic group than that in the nonmetastatic groups.

In the urine, 1,161 proteins were identified. Urine samples from healthy controls were compared to urine samples from different lung adenocarcinoma groups to obtain differential protein levels. Proteins that were upregulated by >1.5-fold or downregulated by <0.67-fold in urine were considered significant. Based on this rule, levels of 461 urine proteins were increased, and levels of 332 proteins were decreased (Table 2 and Table S3, urine). Changes in urine proteins are indicated on a heat map (Figure 3(a)). We had carried on the thorough analysis to the result and discovered the expression of the prostate-specific antigen, and prostatic acid phosphatase decreased significantly in the metastatic group (stages III and IV) and neutrophil defensin 1 increased significantly in the metastatic group compared to the nonmetastatic group (stages I and II) (Figures 3(c)–3(e)). Changes in these proteins were consistent with those reported in the literature.

### 3.4. Identification of Differentially Expressed Proteins in the Serum

**Figure 4:**
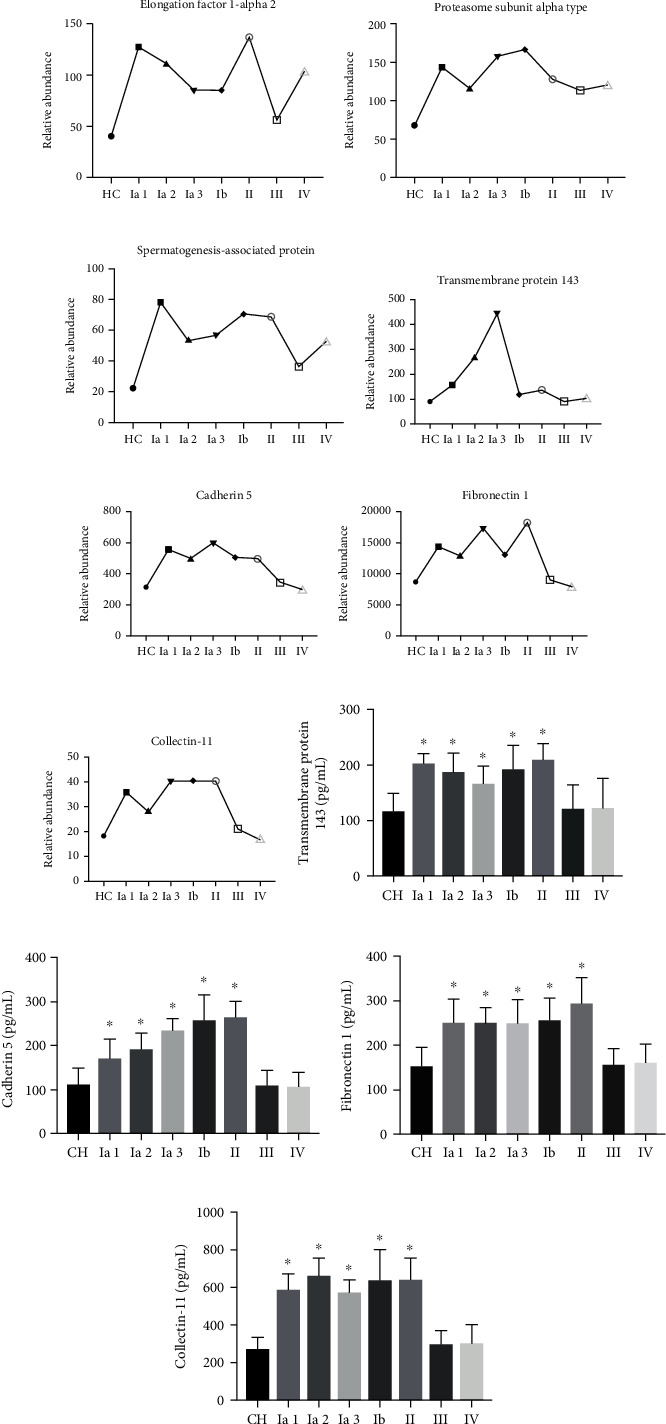
Differences the proteins in serum samples. (a–c) The level of expression elongation factor 1-alpha 2, proteasome subunit alpha type, and spermatogenesis-associated in each group. (d–g) LC-MS/MS of transmembrane protein 143, cadherin 5, fibronectin 1, and collectin-11; (h, k) ELISA of transmembrane protein 143, cadherin 5, fibronectin 1, and collectin-11. ^∗^*P* < 0.05.

A total of 441 proteins were identified in serum samples. Serum samples from healthy controls were compared to serum samples from different lung adenocarcinoma groups to obtain differential protein levels. Proteins that were upregulated by >1.3-fold or downregulated by <0.77-fold in serum were considered significant. Based on this rule, 425 proteins increased, and 73 proteins decreased (Table 3 and Table S4 serum), and then we made the heat map for the changed in serum proteins (Figure 3(b)). Elongation factor 1-alpha 2 (Q05639), proteasome subunit alpha type (B2RDG0), and a spermatogenesis-associated protein (A0A0R4J2F1) were significantly increased (>1.3-fold) in all stages of lung adenocarcinoma compared with those of healthy controls (Figures 4(a)–4(c)). Transmembrane protein 143 (Q96AN5), cadherin 5 (Q59EA3), fibronectin 1 (A0A024R462), and collectin-11 (Q9BWP8) were significantly lower (<0.77-fold) in adenocarcinoma cancer stages III and IV compared with the level of expression in stages I and II (untransferred) (Figures 4(d)–4(g)). ELISAs were used to verify the four proteins, and the results were consistent with those of the LC-MS/MS (Figures 4(h)–4(k)).

### 3.5. Bioinformatics Analysis of Differential Proteins in Serum Samples

**Figure 5:**
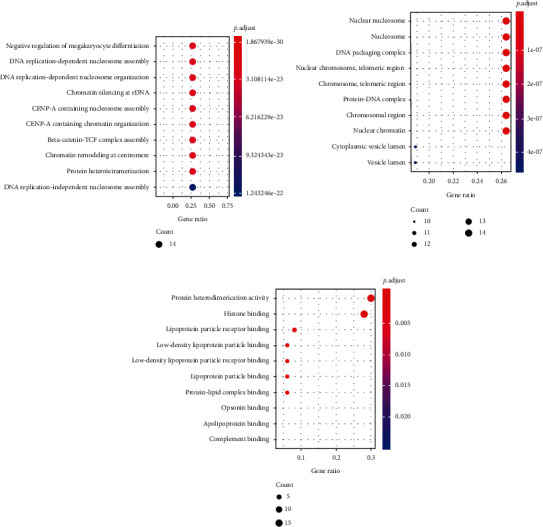
The GO analysis the protein markers in the serum samples. (a) Serum biological process (BP). (b) Serum cellular component (CC). (c) Serum molecular function (MF).

Significantly differentially expressed proteins from the LC-MS/MS were analyzed by Blast2Go and clusterProfiler software with Human as the background library, followed by GO annotations, including a biological process (BP), a cellular component (CC), and a molecular function (MF) (Figures 5(a)–5(c)).

In the BP analysis (Figure 5(a)), the biological processes associated with cell proliferation, including DNA replication, chromosome assembly, and organization, as well as beta-catenin-TCF complex assembly, were significantly altered relative to normal samples. Changes in biological processes also changed cellular components and molecular functions. In the CC analysis (Figure 5(b)), significant changes in cancer patients included the interaction of cellular genetic material and DNA proteins. In the MF analysis (Figure 5(c)), changes in histone binding were most pronounced, because histidine is abundantly present in the cell chromatin, and the protein regulates changes in genetic material by binding to histones. Taken together, the GO analysis demonstrated significant changes in protein function in lung cancer patients, particularly those associated with cell proliferation, differentiation, and metastasis.

### 3.6. Bioinformatics Analysis of the Differential Proteins in Urine Samples

**Figure 6:**
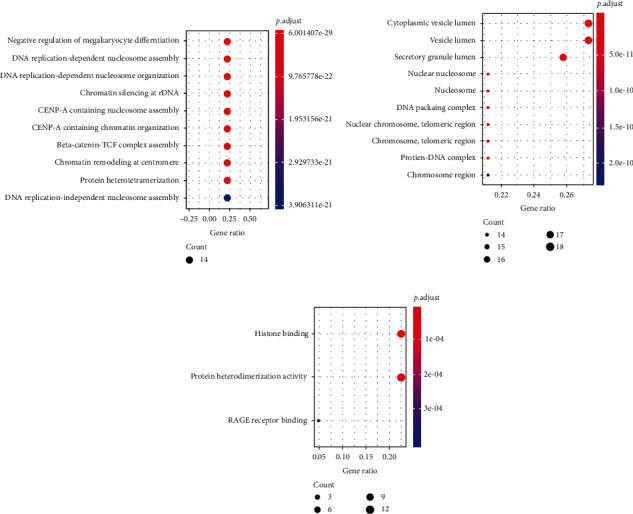
The GO analysis the protein markers in the urine samples. (a) Urine Biological process (BP). (b) Urine cellular component (CC). (c) Urine molecular function (MF).

In urine samples, we used Blast2Go and clusterProfiler software to analyze differentially expressed proteins with human as the background library, followed by GO annotations, including BP, CC, and MF (Figures 6(a)–6(c)). The results of the BP analysis were similar to those for the serum samples, which are consistent with changes in DNA replication and chromosome assembly, and they verify the rapid infinite value added by cancer cells (Figure 6(a)). However, in CC and MF analyses, urine samples still exhibited changes different from serum. In the CC analysis (Figure 6(b)), the result of urine analysis differed from the serum result, mainly reflecting the fact that in the urine, the vesicle lumens changed significantly in the lung cancer group. In the MF analysis (Figure 6(c)), changes in histone binding and protein heteromerization were consistent with the results in the serum.

## 4. Discussion

Using iTRAQ8-plex labeling combined with liquid chromatography-tandem mass spectrometry as a quantitative proteomics analysis method of analyzing the serum and urine samples of healthy controls and stage I, II, III, and IV non-small-cell lung adenocarcinoma patients, we identified a total of 441 proteins in serum samples and 1,161 proteins in urine. In the serum samples, elongation factor 1-alpha 2, proteasome subunit alpha type, and the spermatogenesis-associated protein increased significantly in all stages of lung adenocarcinoma compared with healthy controls. In stage III and IV patients, the expression levels of transmembrane protein 143, cadherin 5, fibronectin 1, and collectin-11 decreased significantly compared with those in stages I and II. In urine samples, the prostate-specific antigen and prostatic acid phosphatase levels were significantly decreased, whereas neutrophil defensin 1 was significantly elevated in stage III and IV lung adenocarcinoma patients. These results are consistent with previous reports verifying that the method is reliable. Moreover, these differentiated proteins may serve as potential diagnostic markers for the early diagnosis of lung adenocarcinoma. In addition, combining the markers in serum and urine may distinguish different stages of lung adenocarcinoma.

Due to the rapid division and proliferation of cancer cells, the synthesis and metabolism of nucleic acids and proteins are increased compared with those in normal cells. Thus, the associated protein content is increased [26, 27] relative to normal cells. Elongation factor 1-alpha 2 has been reported in the literature as a potential marker for cancer [17]. The proteasome subunit alpha type is significantly increased in all cancers, consistent with literature reports [17, 27, 28]. Protein ubiquitination and subsequent proteolysis and degradation by the proteasome are important mechanisms in the cell cycle, cell growth and differentiation, gene transcription, signal transduction, and apoptosis [29]. Proteasomes hydrolyze cells and control apoptosis. Proteosomes bind to the p21 protein and inhibit p21 protein activity [30], leading to cell cycle disorders and, ultimately, cancer. Therefore, the increased expression of this protein in cancer cells inhibits the tumor-suppressing effect of p21. The proteasome subunit alpha type may be significantly associated with the protein metabolism in the body, and its expression is associated with cancer [30, 31]. The spermatogenesis-associated protein is a spermatogenesis-related protein that is mainly found in mammalian tissues and is significantly elevated in prostate cancer tissues [32], testicular cancer tissues [32, 33], and breast cancer tissues [34]. The expression of spermatogenesis-associated 6 (SPATA6) is significantly elevated in testicular cancer, and inhibition of SPATA6 expression can cause cancer cells to die [35]. SPATA20 is significantly elevated in cholangiocarcinoma [36]. Our results are consistent with those reported in the literature.

The prostate-specific antigen (PSA) and prostatic acid phosphatase are markers for the diagnosis of prostate cancer [37]; elevated total PSA (tPSA) and free PSA (fPSA) and decreased tPSA/fPSA indicate prostate cancer [38]. We demonstrated a significant downregulation of PSA in metastatic lung cancer urine relative to nontransfer of lung cancer. Neutrophil defensin 1 protein is important in the neutrophil defense system and has antitumor effects [39]. In advanced metastatic lung cancer, the metabolism and activity of the tumor are increased, and neutrophil defensin 1 is part of its defense system. Thus, neutrophil defensin 1 can be used as an indicator of cancer prognosis.

Transmembrane protein 143 was present at different levels in different stages of lung cancer and may be important in the early diagnosis and prognosis of cancer. These results are novel and have not been reported. E-cadherin acts as an invasion suppressor and a classical tumor suppressor gene. E-cadherin is a biomarker for cancer [18, 40] and is attenuated and reduced in many cancers [41–43], especially in lung and breast cancers. E-cadherin loss in tumor cells leads to decreased adhesion between tumor cells, which favors epithelial-mesenchymal transition [44] and promotes the ability of tumor cells to invade and metastasize. In metastatic lung cancer (stages III and IV), E-cadherin protein levels were significantly lower than those in nonmetastatic stages (I and II). Fibronectin can regulate cancer and the migration of cancer cells, which is closely associated with the prognosis of tumor formation and development. However, the mechanisms underlying this relationship are unclear [45]. Fibronectin is a biomarker [18, 45–48], which is upregulated in cancer, and our results are consistent with the literature. Fibronectin 1 can bind to cancer cells and favors metastasis and invasion [48, 49]. Cancer prognosis studies demonstrate that the higher the level of fibronectin 1 in vivo, the worse the prognosis and survival rate [45]. When cancer occurs, fibronectin 1 expression increases, promoting adhesion and invasion of cancer cells and increasing the damage to normal tissues. Therefore, fibronectin 1 is also an important indicator of cancer prognosis. After cancer treatment, if fibronectin 1 levels are still high, the prognosis is poor.

Collectin-11 decreased significantly in stage III and IV adenocarcinomas compared to earlier stages. Collectins are a family of collagenous calcium-dependent defense lectins in animals [50]. Collectins are humoral molecules of the innate immune system that modulate inflammatory and allergic responses, the adaptive immune system, and clearance of apoptotic cells [51, 52]. Collectin-11 is directly related to cancer and the human immune system. In stage I and stage II lung cancer, collectin-11 is elevated because the human immune system is still in the early stage of cancer development. In stage III and stage IV cancer, collectin-11 is decreased, by the massive proliferation and metastasis of cancer cells, a process that destroys the normal immune system. This report is the first to identify changes in collectin-11 in lung cancer, which can be used as a marker for staging non-small-cell lung cancer.

Based on the biological analysis of differential proteins in serum and urine, the protein changes in serum and urine were similar, indicating that the biological processes of blood and urine were interrelated and consistent. In the CC analysis, the changes in urine vesicles in the lung cancer group were more pronounced compared to those in the blood. The results of serum and urine are partially consistent, but urine and serum also have unique characteristics, so the combination provides a better reference for the selection of biomarkers. In summary, these differentiated proteins may be potential diagnostic markers for lung adenocarcinoma and may serve as a basis for the early diagnosis of lung adenocarcinoma. Further research is required to verify the experimental results.

## Figures and Tables

**Table 1:** Clinical profiles and demographics of healthy controls and lung adenocarcinoma patients with different stages.

Demographics	Control	Stage Ia1	Stage Ia2	Stage Ia3	Stage Ib	Stage II	Stage III	Stage IV
n	30	10	10	10	10	10	10	10
Age	55 ± 8.32	53 ± 5.91	55.9 ± 6.79	54.3 ± 4.06	57.4 ± 8.37	57.9 ± 6.35	57.2 ± 5.39	57.8 ± 9.96
Male	21	7	7	7	7	7	7	7
Female	9	3	3	3	3	3	3	3
Smoking history	12	4	4	4	4	4	4	4
Ethnicity	Han (30)	Han (10)	Han (10)	Han (10)	Han (10)	Han (10)	Han (10)	Han (10)

121: healthy control; 113: stage Ia 1; 114: stage Ia2; 115: stage Ia3; 116: stage Ib; 117: stage II; 118: stage III; 119: stage IV.

**Table 2:** Differential protein statistics of urine samples from different stages of lung adenocarcinoma.

	113/121	114/121	115/121	116/121	117/121	118/121	119/121
Upregulated	142	74	47	33	38	77	50
Downregulated	10	42	197	14	6	26	37

121: control group; 113: stage Ia 1; 114: stage Ia2; 115: stage Ia3; 116: stage Ib; 117: stage II; 118: stage III; 119: stage IV.

**Table 3:** Differential protein statistics of serum samples from different stages of lung adenocarcinoma.

iTRAQ	113/121	114/121	115/121	116/121	117/121	118/121	119/121
Upregulated	93	41	98	78	51	35	29
Downregulated	5	4	4	8	5	8	39

121: control group; 113: stage Ia 1; 114: stage Ia2; 115: stage Ia3; 116: stage Ib; 117: stage II; 118: stage III; 119: stage IV.

## Data Availability

The datasets used and/or analyzed during the present study are available from Tables S1, S2, S3, and S4.

## Supplementary Materials

Supplementary 1 Table S1: the results of serum reversed-phase elution test and searching in the SEQUEST database.

Supplementary 2 Table S2: the results of urine reversed-phase elution test and searching in the SEQUEST database.

Supplementary 3 Table S3: a further study on the LC-MS analysis results of urine in Table S2. Urine samples from healthy controls were compared to urine samples from different lung adenocarcinoma groups to obtain differential protein levels.

Supplementary 4 Table S4: a further study on the LC-MS analysis results of serum in Table S1. Serum samples from healthy controls were compared to serum samples from different lung adenocarcinoma groups to obtain differential protein levels.
