# Supplementary material for: LC-MS/MS-Based Quantitative Proteomics Analysis of Different Stages of Non-Small-Cell Lung Cancer
Source: Biomed Res Int. 2021 Feb 26;2021:5561569. doi: 10.1155/2021/5561569 (PMC7937045; doi:10.1155/2021/5561569)
Supplement: Supplementary 1 — Table S1: the results of serum reversed-phase elution test and searching in the SEQUEST database. [file 5561569.f1.pdf]

Uniprot Accession Number	Protein	protein amino acid sequence	protein amino acid sequence matching degree （%）	number of identified polypeptides	total number of identified polypeptides	characteristic polypeptide number identified	ength of amino acid	The molecular weight KDa	isoelectric point	relative abundance of normalized protein - sample 113	relative abundance of normalized protein - sample 114	relative abundance of normalized protein - sample 115	relative abundance of normalized protein - sample 116	relative abundance of normalized protein - sample 117	relative abundance of normalized protein - sample 118	relative abundance of normalized protein - sample 119	relative abundance of normalized protein - sample 121
C0JYY2	Apolipopro	MDPPRPAL	48	227	8770	178	4563	515.2	7.05	587089.8	722432.2	577726.5	553022.3	631905.3	553297.9	487383.6	510456.9
V9HWA9	Epididymis	MGPTSGPS	65	117	13443	117	1663	187	6.4	1038496	987768	1014543	1059000	1036689	1058344	1125990	1086362
A0A140TA	Compleme	MRLLWGLI	48	76	5311	1	1698	187.5	7.33	10298.9	11246.3	10190.4	9787.3	10555.8	9545.9	7018.6	10069.7
A0A140TA	Compleme	MRLLWGLI	47	75	5311	0	1698	187.6	7.12								
P0C0L4	Compleme	MRLLWGLI	47	78	5222	4	1744	192.7	7.08	354515.7	358550.6	353533.8	371096.1	369917.8	392042.8	352593.5	379640.7
A8K5A4	cDNA FLJ76	MKILILGIFL	49	48	2852	3	1065	122.1	5.74	184669.2	167773	172794.9	189477.9	181011.4	192480.9	188787.6	181927.1
P08603	Compleme	MRLLAKIIC	58	65	2223	2	1231	139	6.61	139326.8	138368.7	152055.5	147415.7	134161.2	124277.4	143670	127138.9
A8K5T0	cDNA FLJ75	MRLLAKIIC	57	64	2215	1	1231	138.9	6.71	2166.5	1478.4	1667	1764.5	1600.3	1443.2	1747.7	1595.6
Q7Z7Q0	APOB prot	MDPPRPAL	58	50	3404	1	825	92.3	7.42	378.4	434.3	176	338.2	290.4	163	248.4	369.3
E9PFZ2	Ceruloplas	MKILILGIFL	53	46	2570	1	946	108.8	5.77	305.6	1344.1	340.1	294.9	245.3	225.6	226.5	232
P01023	Alpha-2-ma	MGKNKLLH	50	60	2148	54	1474	163.2	6.46	159242.4	150022.1	148585.9	127199.8	139287.4	196290.4	150833.5	155893
B4E1Z4	cDNA FLJ55	MGPLMVLI	46	58	2410	38	1266	140.9	7.18	168887.5	175330.4	170379.3	181366.8	186553.2	182496	179532.4	179991.2
P02768	Serum albu	MKWVTFIS	62	47	4176	47	609	69.3	6.28	393627.8	415295.5	553515.2	401168.8	313800.8	273544.9	216497.6	374998.6
P00747	Plasminoge	MEHKEVVL	64	54	1676	46	810	90.5	7.24	123328	118392.6	116615.2	117254.4	115644.8	104325.5	105844.6	110712.8
P02790	Hemopexin	MARVLGAF	64	31	5337	31	462	51.6	7.02	374618.6	386591	389342.2	396054.9	408398.8	392817.7	388013.2	407277.3
P00738	Haptoglobi	MSALGAVI	57	27	2184	12	406	45.2	6.58	185102	162600.1	155639.9	279577.3	162525.6	258436.5	410792.3	185933.7
A0A024R3	Apolipopro	MKAAVLTL	86	33	4430	33	267	30.8	5.76	338583	381625.8	366565	373110.1	364223.9	375076	391751.5	411885.3
P00734	Prothromb	MAHVRGL	55	32	1104	32	622	70	5.9	69844.1	67971.2	72856.6	63897.5	65950.1	56143.8	58040.4	57305.1
B4E1C2	Kininogen	MKLITILFL	40	32	1754	5	644	71.9	6.81	125717	122596.9	124775.5	130027.4	132243.9	130159.7	124968.7	131253.4
P01031	Compleme	MGLLGILCF	36	63	1230	63	1676	188.2	6.52	94890.7	90259.9	89862.7	87867	94067.8	91781.4	92978.4	93176.8
D3DNU8	Kininogen	MKLITILFL	54	29	1813	2	427	47.8	6.65	6418.8	6744.1	6171.8	6016	6433	9059.8	8373.3	7787.9
B4E1B2	cDNA FLJ53	MRLAVGAL	63	47	1180	5	678	74.8	7.12	92039.8	81713.4	90197.9	70892.5	76534	83395	66649.1	83286.1
E9KL23	Epididymis	MPSSVSW	61	31	1738	1	418	46.7	5.59	8613.5	5499.6	5387.8	4010.7	5925.8	6670.1	4887.9	6306.2
A0A024R6	Alpha-1-an	MPSSVSW	61	31	1672	1	418	46.7	5.59	129336.8	145830.8	133687.4	163476	143911.9	158637.4	254797.8	165755.8
A0A0K0K1	Epididymis	MRLAVGAL	59	44	1153	2	698	77	7.21	905.6	975.5	1107.8	873	818.1	1068.3	849.7	866.1
D6RF35	Vitamin D-	MKRVLVLL	62	32	2227	2	476	53	5.52	191458.5	193095.1	175613.7	171725	181511.4	200116.5	203654.9	203990.6
V9HWI6	Epididymis	MKRVLVLL	66	32	2121	2	474	52.9	5.54	1493.4	1742	1355.4	1572.2	2818.7	2236	1573.4	1938.8
D3DRR6	Inter-alpha	MKRLTCFF	32	32	1638	32	947	106.5	6.95	108481.7	98996.5	99369.2	94019.8	105151.6	106137.5	95323.2	108810.6
B2RMS9	Inter-alpha	MKPPRPVR	35	32	1588	32	930	103.3	6.98	108110.8	103576.1	102139.3	101346.1	109288.1	106762.4	106066.8	104677.5
A8K2T4	cDNA FLJ78	MKVISLFIL	50	37	618	4	843	93.3	6.51	39819.6	35815.5	37899.4	38127.9	37936.9	36598.8	35816.4	34968
B7Z539	cDNA FLJ56	MDGAMGF	40	23	951	16	645	72.1	7.68	54625.6	50116.6	55433.1	58397.6	56789.4	51279.7	46599.2	53820
P06727	Apolipopro	MFLKAVVL	70	36	1655	36	396	45.4	5.38	121784.2	122790	118549.5	111855.3	125959.3	118246.5	98547	132070.1
P04003	C4b-bindin	MHPPKTPS	59	28	1262	1	597	67	7.3	98140.4	87061.7	94314.1	96031.3	95727.1	96432.8	93592.4	89101.1
B2R6W1	cDNA, FLJ9	MKVISLFIL	48	35	595	2	843	93.5	6.48	467.6	371.3	403.2	355.5	366.1	384.4	402.6	340
B4E1D8	cDNA FLJ51	MDITLTETF	66	28	1251	1	536	60.4	6.65	419.3	405.5	393	441.7	454.7	390.7	344.8	356.1

P00739	Haptoglobi	MSDLGAVI	53	21	1147	6	348	39	7.09	2606.9	2512.7	2255.3	2714.9	2471.1	3397.7	4403	2635.5
B7Z1F8	cDNA FLJ53	MCPEDSLQ	67	18	1520	3	270	30.4	7.99	6148.8	5133	6345.8	5999.2	5232.9	5756.8	5222.4	5945
P06396	Gelsolin OS	MAPHRPAI	41	30	734	28	782	85.6	6.28	51360.2	48208.4	50016.7	46518.3	50644.9	50760.1	44495	53072.9
D9IWP9	Beta-2-glyc	GRTCPKPD	53	17	1394	17	326	36.2	8	95869.3	94684.3	92089.5	92442.1	97157.4	95290.3	97045.8	95095.7
A0A024R94	Serpin pep	MYSNVIGT	44	28	1040	28	464	52.6	6.71	81924	75341.2	73519.1	66972.7	79640.7	77286.2	59152.2	76446.5
B3KS79	cDNA FLJ35	MKIHYSRQ	34	19	1335	19	448	50.6	5.63	93851	98190.2	91704	98831.5	103081.2	95366.8	132515.7	99858
P13671	Compleme	MARRSVLY	39	35	538	4	934	104.7	6.76	36577.2	33403.3	34161.9	36176	36281.2	33438.1	34559.2	34641.2
E9PGN7	Plasma pro	MGAGPGR	27	17	1413	17	543	59.5	6.76	100248.8	112257.1	98724.4	106678.1	114992.3	110430.4	101300.1	110273.8
P02760	Protein AM	MRSLGALL	47	14	816	14	352	39	6.25	40442	36134.6	39749.4	40433.6	41020	38398	34490.9	38024
A8K8Z4	cDNA FLJ78	MARRSVLY	36	32	516	1	934	104.6	6.62	91.3	102.6	84.4	91.7	76	104.5	85.7	91.7
B2R8I2	cDNA, FLJ9	MKALIAALL	51	21	701	1	525	59.5	7.44	56488.4	55925.9	42986.9	48963.1	56480.5	61840.2	50180	53687.5
H0YAC1	Plasma kall	XSEDCEVA(	46	30	491	30	686	76.8	8.06	34217.8	32918	34183.6	32889.7	34399	32603.1	29661.8	33082.6
P04196	Histidine-ri	MKALIAALL	51	21	685	1	525	59.5	7.5	866.1	992.4	590.2	622.6	688.2	799.8	706.1	661.8
P07358	Compleme	MKNSRTW	47	25	410	25	591	67	8.13	24943.9	24633.6	24581.7	24823.5	24915.6	24972.2	23582.2	25346.9
P43652	Afamin OS=	MKLLKLTGI	48	33	792	33	599	69	5.9	62649.1	58267.1	59622.5	58119.3	61682.8	62913.2	54287.5	60761.3
P06681	Compleme	MGPLMVLI	40	26	426	6	752	83.2	7.42	5761.7	5226.7	5861.4	5486.3	5452.7	5408.6	5137.8	5096.4
P04275	von Willeb	MIPARFAG	23	51	243	2	2813	309.1	5.48	13089.5	11473.5	12553.1	13082	13206.5	13496.9	11111.4	10196
A0A024R9(	Thrombosp	MGLAWGL	37	39	387	1	1170	129.3	4.94	26614.2	21742.9	25643.9	21725	27021.2	22529.8	20044.7	21666.3
P07996	Thrombosp	MGLAWGL	37	39	390	1	1170	129.3	4.94	557.4	499.6	559	467	606.6	606.1	458.5	392.8
P10909	Clusterin O	MMKTLLLF	39	18	653	18	449	52.5	6.27	50095.5	45302.4	48175.9	48440.7	47018.7	34883.3	34107.1	33598.7
O75882	Attractin O	MVAAAAA	25	32	349	32	1429	158.4	7.31	27658.2	25777.2	25986.1	25151.2	26915.2	24672.1	23733.1	25522.8
E7ETH0	Compleme	MKLLHVFLI	35	19	499	5	591	66.6	7.59	28620.6	26667.1	27252.9	26287.2	27657.8	25695.2	27742.1	26360
L8E853	von Willeb	MIPARFAG	23	50	239	1	2715	298.2	5.48								
B3KNX0	cDNA FLJ3(	MWCIVLFS	41	23	366	23	688	76.6	4.96	27673.9	25972.1	24866.1	26889	25674.2	23715.2	25775	24353
A0A024R4(	Fibronectir	MLRGPGP(	22	43	227	43	2355	259	5.73	14350.7	12920.5	17208	13008.3	18122.1	8789.5	7557	8653
B4DPQ0	Compleme	MSVRFRVG	39	24	381	22	719	81.8	6.37	29398.6	29623.9	30198.6	30114.2	28399.3	25563.7	28589.3	27059.9
P02649	Apolipopro	MKVLWAA	61	22	910	20	317	36.1	5.73	60795.2	53606.3	61843.3	62405.6	66560.6	58524.1	55467.3	54616
P07357	Compleme	MFAVVFFIL	41	22	359	22	584	65.1	6.47	21076	19665.2	20194.4	19189.5	20228.2	19921.3	19774.3	19863.5
A0A024R03	Compleme	MSACWSFA	36	23	501	23	559	63.2	5.52	28186.5	29862.6	26992.6	31248.7	30833.4	31964.7	39963.3	31276.8
Q6LAM1	Heavy chai	KVTYTSQEI	50	16	369	2	321	35.9	7.59	5884.2	5922.3	5458.6	5879.3	6019	6552.1	9038.4	7000
P05546	Heparin co	MKHSLNAL	42	22	614	22	499	57	6.9	42058.8	39849.4	38495	41473.5	42452	44464.2	39529.8	40235.4
Q9UNU2	Compleme	HRGRTLDIF	64	15	894	3	354	38.1	8.53	2438.3	2216	2211.7	1819.1	2243	1953.8	2485	2086.8
V9HWD8	Epididymis	MSMLVVFL	41	13	1100	8	495	54.2	5.86	85465.5	85173.4	82675.7	85248.3	87032.2	83186	82018.4	87354.8
A0A0S2Z4L	Protein S is	MRVLGGR(	34	21	322	21	708	78.8	5.74	20521.9	19479.9	20695.3	18904.4	20998.2	20174.6	20330.7	20335.7
D9ZGG2	Vitronectir	MAPLRPLLI	24	10	849	10	478	54.3	5.8	47676.1	42103.8	50280.8	50872	50370.3	44782.5	40512.9	45241.8
B7Z8Q2	cDNA FLJ55	MFAGCFFF	26	12	917	12	433	46.6	6.28	72510.6	71430.7	69072.4	68315.1	75454.7	69386.5	66683.6	78902.1
D9YZU5	Beta-globir	MVHLTPEE	79	12	430	1	147	16	7.28	26762	25508.7	21785.1	27519.4	20787.3	30161.5	30166.4	26416.6
B7Z8B6	cDNA FLJ54	MNKNVVF\	27	10	354	3	623	69.5	6.16	4851.3	3182.4	3426.6	2914.7	3116.9	4486.4	3978.8	3715.2
A0A1B0GU	Ig mu chair	GSASAPTLF	38	16	591	3	474	51.9	6.15	33261.6	33963.5	33291.6	31512.1	39637.9	55273.6	42527.6	41758.8
B2R950	cDNA, FLJ9	MRKDRLLH	19	23	307	17	1482	163.8	6.38	2645.4	2669.2	2807.5	2688	3024	2831.3	3108.6	2893
Q96PD5	N-acetylmu	MAQGVLW	26	10	366	10	576	62.2	7.55	20449.1	16845.4	17634.1	17893.7	18152.3	17621.4	16026.7	18131.8
P02763	Alpha-1-ac	MALSWVLT	45	9	562	6	201	23.5	5.02	20978.4	23424.7	17359	26223.1	21535.6	24203.1	45114	21588.2

B0AZL7	cDNA, FLJ7	MALRKGGL	37	19	355	19	605	66	6.79	22324.3	19098.3	20124.5	19414.7	21036	19426.1	16382.5	18319.3
B2R815	cDNA, FLJ9	MHLIDYLLL	38	15	289	15	427	48.5	7.75	20326.1	19314.7	17136.1	15861.9	19134.1	18435.3	13815.6	18523.7
P08697	Alpha-2-an	MALLWGLL	32	16	311	16	491	54.5	6.29	15568.9	15471.1	16057.6	15651.7	15778.6	15332.5	15477.2	16208.5
P25311	Zinc-alpha-	MVRMVPV	51	18	353	18	298	34.2	6.05	24197	22776.3	22025	20191.2	24868.6	21172.2	17749	22823.9
Q86TT1	Full-length	MQGTDEH	41	14	575	1	375	41.2	6.79	731.5	612.5	656.3	623.5	686.7	818	646.6	628.3
V9HW68	Epididymis	MELGLSW	42	15	216	1	470	51.7	7.75	14200.7	13471.4	14091.7	14781.3	14841.2	12780.1	15179.6	14318
Q06033	Inter-alpha	MAFAWWI	18	14	267	14	890	99.8	5.74	13567.8	12959.8	12149.5	12249.6	12309.6	12294.9	12842.5	12468.4
P80108	Phosphatic	MSAFRLWI	24	20	227	20	840	92.3	6.37	11082	10784.2	10919.1	10552.3	10448.7	10629.6	9586.6	9864.1
P07360	Compleme	MLPPGTAT	58	11	203	11	202	22.3	8.31	10529.2	9986.8	10975.9	10507.7	10028.2	10438.8	9234.4	10063.5
P22792	Carboxype	MLPGAWLI	22	11	310	11	545	60.5	5.99	18835.2	18865.2	17056.8	16192.8	17940.2	16797.5	14238.6	16767.1
A0A140VK	Testis tissu	MQALVLLL	35	14	372	14	418	46.3	6.38	25131.6	22440.4	22366.6	20490.3	23660.7	22065.7	21306.7	22611
P51884	Lumican O	MSLSAFTLF	36	11	361	11	338	38.4	6.61	21871.6	20178.2	20998.2	20168.7	21854.5	20957.9	18878.5	21478.9
A0A0A0MI	Coagulatio	MFPGCPRL	15	31	140	30	2229	252.1	6.05	8192.9	7006.1	7385.3	6954.2	8063.6	6384.5	6217.3	6480.2
Q59EP2	Angiotensi	VLQQKGM	26	13	474	13	491	53.7	6.42	24341.2	23979.5	23177.3	23539.2	25181.7	23425	22223.6	24089.4
B2R582	cDNA, FLJ9	MELWGAY	57	10	217	1	202	22.5	6.04	921.9	782	830.3	714.9	798.1	808.3	819.5	798
Q4TZM4	Hemoglobi	TPEEKSAVT	89	9	235	2	101	11	6.52	1405	1277.4	979.5	1322.2	935.1	1697.9	1700.3	1332.1
Q6MZQ6	Putative ur	MEFGLSWI	35	13	199	2	475	52	8.06								
P27169	Serum para	MAKLIALTL	33	9	411	7	355	39.7	5.22	24841.5	26246	25042.9	24302	27227.6	26242.8	22453.9	26425.9
D6RF20	Vitamin D-	MKRVLVLLI	76	10	581	1	145	16.1	5.83	28.9	18.6	31.5	25.4	23.9	27.1	21.2	24.1
Q96K68	cDNA FLJ14	MELGLRW	29	12	184	4	494	53.1	6.86	9530.9	9767.8	10313.7	9694.6	9181.8	9114	12195.3	9509.7
G3XAK1	Hepatocyte	MGLWWV	34	20	129	20	725	81.9	7.8	6901.6	6318.4	6943.5	6491.8	6537.2	5956.3	5849.2	6404.4
A0A024R2	C-type lect	MELWGAY	57	10	189	1	202	22.5	5.67	8337.9	7454.1	7456.1	6832.4	8007.7	7034	6269.8	7606
B2R9F2	cDNA, FLJ9	MPLLLYTCL	23	8	306	8	405	45.1	6.04	24324.2	24773.4	18653.9	20418.2	23084.3	23221.8	21849.1	21182.2
P05543	Thyroxine-	MSPFLYLVL	29	13	196	13	415	46.3	6.3	10969	11223.3	10323.5	10070	11408.6	11465.7	10219.8	10522.1
A0N071	Delta globi	MVHLTPEE	57	9	216	3	147	16	8.05	124.5	109	104.9	128.9	97.3	127.9	133	110.6
P02753	Retinol-bin	MKWVWAI	50	9	473	9	201	23	6.07	34673.7	31209.4	35599.5	34712.9	35446.5	33435.2	27320.1	33157.6
C9JF17	Apolipopro	MFKQLSCIS	31	7	421	7	215	24.1	5.6	26206.3	26020.4	27458.5	23217.7	27609.1	23713.6	21141.3	24386.8
A8K1K1	cDNA FLJ76	MDPKLGRN	34	14	150	14	507	56.7	5.36	7542.4	7262.6	7434.9	6611.6	6953.1	6538.6	5646.1	6742.3
A0A1K0GX	Globin C1 (	MSAARPGV	51	7	179	7	177	19.2	9.77	14157.8	14141.1	9465.6	14883	10905.9	15034.8	18249.7	14723.1
D6RAR4	Hepatocyte	MGRWAW	26	13	120	1	662	71.4	7.05	88.7	66.5	89.3	80.3	82.1	88.3	60.3	76.6
Q16610	Extracellula	MGTTARA	28	13	102	13	540	60.6	6.71	4777.3	4009.6	4909.4	4730.8	4439.4	4352.6	3766.9	4476.7
Q04756	Hepatocyte	MGRWAW	26	13	127	1	655	70.6	7.24	6577.1	5836.5	6665.3	5873.9	6133.9	5968.9	5362	6012.3
Q8TCD0	Uncharacte	MRLPAQLL	41	6	154	1	239	26.2	8.06	415.3	418.3	400.5	341.9	406.3	519.1	440.6	466.9
P03951	Coagulatio	MIFLYQVVI	30	17	87	17	625	70.1	8.1	5192.9	4314.6	4788.6	4719.5	4850.2	4739	4267.9	4345.1
Q03591	Compleme	MWLLVSVI	28	10	157	1	330	37.6	7.39	1251.5	1403.4	1466.7	1296.1	1136.9	1164.7	1201	1223.8
A0A0S2Z3	Lectin gala	MTPPRLFW	29	13	127	13	585	65.3	5.27	7320.9	6436	6925.7	6615.1	6572.9	8034.7	8214.9	6950.6
Q6PIL8	IGK@ prote	METPAQLL	40	6	151	1	236	25.8	6.55	7606.5	6323.7	5824.9	5681	5845.4	8047.9	8245.1	7153.1
P02746	Compleme	MMMKIPW	21	4	210	4	253	26.7	8.63	8778.3	8648.3	9690.7	9963.4	8865	8777.6	8280.1	8644
A0A096LP	Protein SA	MKLLTGLV	32	7	151	5	208	23.3	8.98	10688.6	10717.2	11896	12038.4	11679.4	11065.4	11844.1	10462.9
Q6MZU6	Putative ur	MELGLRW	36	13	102	4	464	51.1	7.71	4359	3634.2	4226.4	4274.9	4095.3	3280.3	3843.7	3853.2
P02750	Leucine-ric	MSSWSRQ	28	9	294	9	347	38.2	6.95	19280.5	19204.6	19544.7	22329.2	19626.4	21509.2	27178.7	20434.1
P48740	Mannan-bi	MRWLLLYY	28	15	103	15	699	79.2	5.49	7116.6	6236	6539.9	6207.5	6035.4	6160.2	5467.1	5616.9

P02775	Platelet ba	MSLRLDTTI	45	7	212	7	128	13.9	8.79	17782.9	15010.3	17397.2	17472.2	19286.4	15447.8	15413.3	15640.4
A6XNE2	Compleme	MHSWERL	39	7	65	7	260	27.8	7.25	3661.6	3079	3909.2	4144.5	3542.2	3244.9	2871.5	3098.3
P02747	Compleme	MDVGPSSL	25	5	143	5	245	25.8	8.41	8248.2	8431.2	8152.3	8062.2	7885.1	7807.7	8072.6	8282.3
P15169	Carboxype	MSDLLSVFI	25	9	104	9	458	52.3	7.34	6941.6	6477.7	7257	7312.7	7140.8	6848.2	6112.5	6584.9
P00742	Coagulatio	MGRPLHLV	30	14	112	14	488	54.7	5.94	8976.6	8245.7	8527.9	8393.9	8808	7701.3	7101.8	8124.9
P19652	Alpha-1-ac	MALSWVLT	47	10	310	7	201	23.6	5.11	19165	20571.4	17406.3	19854.6	18726.5	18554.9	27901.1	19263.5
Q14520	Hyaluronar	MFARMSD	28	15	167	15	560	62.6	6.54	11316.7	10532.6	11084.5	11173.5	11337.9	10612	10238.1	11039.1
F2RM37	Coagulatio	MQRVNMI	34	14	90	14	461	51.7	5.47	6699.2	6153.5	6191.1	6563.2	6383.7	6621.1	6072.5	6388.3
Q96IY4	Carboxype	MKLCSLAV	24	10	161	10	423	48.4	7.71	12269.4	11550.9	13003.2	12811.5	12370	12796.3	10423.8	11789.6
A2NUT2	Lambda-ch	MTCSPLLLT	35	7	219	2	235	24.6	7.62	15187.4	14275.1	15722.5	15169.7	16572.9	14111.3	15719	14755.2
O75636	Ficolin-3 O	MDLLWILP	21	7	227	7	299	32.9	6.67	17638.8	18165.6	16665	14385.8	16004	16769.9	14579.7	15367.2
Q9NPP6	Immunoglo	PGKGLEWV	23	9	119	1	416	44.8	6.13	454.8	457.1	412.1	401.7	470.5	460.3	526.6	439.2
Q567P1	IGL@ prote	MASFPLLLT	38	7	205	1	235	24.8	7.3	1092.4	1442.3	1467.9	1287.8	1469.6	959	1225	1174.7
A0A024R6I	Serpin pep	MQLFLLLCL	27	11	75	11	406	45.6	9.26	4175.3	3766.7	4461.6	4485.9	4064	4083.4	2912.8	3529
H6VRG1	Keratin 1 C	MSRQFSSR	26	15	79	13	645	66.1	8.12	3451.6	3228.1	2797.8	3184.2	3565.9	2341.6	2726	2159.3
A0A0R7FJH	Coagulatio	MRALLLLGI	20	8	53	8	615	67.8	7.8	2998.3	2976.7	2986.4	2883.8	2602.2	2378.2	2213.3	2366.7
P20851	C4b-bindin	MFFWCAC	36	8	125	8	252	28.3	5.14	9027.5	8504	9754.4	9465.3	9543.2	9385.3	9386.6	8738.8
P63261	Actin, cyto	MEEEIAALV	46	12	73	5	375	41.8	5.48	5034.2	4421.8	4569	4498.6	4666.4	4301.8	3602.9	3679
S6AWE6	IgG L chain	MAWFPLFL	32	6	201	1	215	22.9	8.43	446.9	440.7	498.9	495.6	479.3	490	532	443.9
A0A024R0	Apolipopro	MRLFLSLPV	40	7	232	7	83	9.3	8.47	21308.9	26544.7	20263.1	17356.9	22290.7	21967.1	21581	20202.7
O00391	Sulfhydryl	MRRCNSGS	18	11	57	11	747	82.5	8.92	3092.7	2603.2	2911.4	2915	2811.2	2625.9	2246.1	2526.4
P02743	Serum amy	MNKPLLWI	25	6	181	6	223	25.4	6.54	13581.8	12697.6	13609.2	12915.9	14808.5	11096.7	9575.8	12930.8
P02776	Platelet fac	MSSAAGFC	36	4	140	2	101	10.8	8.62	7328	14120.9	14224.7	9230.1	9519.2	9673.7	7847.5	7708.1
P09172	Dopamine	MPALSRW	25	13	57	13	617	69	6.42	2206.3	2263.1	2213	2303	1895.8	2180.4	1778.4	2072.2
P43251	Biotinidase	MAHAHIQ	15	8	72	8	543	61.1	6.25	4160.3	3812.2	3998.6	3618	3739.1	3523.6	2741.5	3353.8
P17936	Insulin-like	MQRARPTL	36	9	124	9	291	31.7	8.69	5551.8	4713.9	4955.8	5015.6	5662.5	5154.3	4313.3	5061.2
A6XGL1	Transthyre	MASHRLLL	27	4	117	4	184	20.2	5.24	7120.2	5982.7	6096.6	5738.3	6744	7894.5	6887.9	6699.7
V9GYM3	Apolipopro	MCEQHPKI	32	6	446	6	133	14.9	8.27	44115.6	54074.9	48466.6	40529.6	47555	51115.9	44102	58257.6
B4DPQ3	cDNA FLJ51	MAAGRRT	15	8	53	8	517	57.4	7.53	3156	2875.5	3008.6	3181.3	3251	3124.7	2837.3	3019.2
Q1HP67	Lipoprotein	MEHKEVVL	18	8	62	8	2040	226.4	6.07	3572.9	3229.5	5065.3	3792.2	3698.1	3973.9	3843.8	4008
B2R888	Monocyte	MERASCLLI	27	10	52	10	375	40	6.23	2126.2	1838	2245	2263	2124.1	1927.7	1746.3	1767.4
Q6GMV7	Uncharacte	MASFPLLLT	20	4	121	2	236	24.9	6.89	719.1	817	835.9	834.4	988	992.9	973.6	874.1
P13645	Keratin, typ	MSVRYSSSI	19	12	89	9	584	58.8	5.21	6299.9	5141.4	5083.2	5122.4	5879.9	4691.1	4037.6	4187.6
A0A024R1	Apolipopro	MRFKSHTV	23	9	68	9	414	45.8	6.29	6244	5517.6	7231.8	5956.6	5785.2	6265	6103	5734.4
P36980	Compleme	MWLLVSVI	29	7	94	4	270	30.6	6.38	697.3	723	663.2	683.4	651.3	658.5	640.3	655.8
O95445	Apolipopro	MFHQIWA	30	7	111	7	188	21.2	6.01	8296.5	9042	8840.9	8126.8	8617.6	8163.2	7562.5	8414.8
Q6MZX7	Putative ur	MELMCKKI	16	6	73	2	476	52.4	7.77	1342.6	1183.7	1099.8	1043.5	1109.7	938.7	1199.3	1228.3
P22352	Glutathion	MARLLQAS	40	8	137	8	226	25.5	8.13	10412	9504.2	9810.5	9756.1	9806.6	9337.8	8045.1	9294
P04278	Sex hormo	MESRGPLA	31	10	62	10	402	43.8	6.71	3130.4	2779.4	2783.5	2998.7	2665.9	2874.3	2493.8	2653.1
K7ER74	Protein AP	MSLLRNRL	26	6	180	4	178	20	6.64	13740.2	12868.2	16333.2	13350.9	13390.5	13986.8	16334.8	13980.3
Q8NF17	FLJ00385 p	LWLWGRE	23	10	100	2	509	56.1	7.59	320.1	247.7	331.5	279.1	251.7	370.4	292.4	242.4
S6BGE0	IgG H chair	MEFGLSW	30	6	66	1	300	32.2	7.85								

A0A0S2Z4I	Compleme	MITEGAQA	23	9	41	9	469	51.2	7.9	4095.2	3644.8	3830.2	3920.4	4101.1	4187.3	3549.1	3322.3
D3DNN4	Carboxylic	MSVQSNLC	15	11	79	11	643	72.8	7.55	5064.1	5240.3	5522.4	4966.8	5218	4797.5	4317.1	5130.5
A0A161I20	Lactoferrin	MKLVFLVLI	25	14	36	14	711	78.3	8.18	1147.8	1147.8	1211.5	1145.7	1138.4	1107.8	981.9	879.4
P00915	Carbonic a	MASPDWG	36	7	55	7	261	28.9	7.12	2162.4	2609.2	1957.8	2055.7	1812.4	2027.6	2103.9	2002.4
Q9UGM5	Fetuin-B O	MGLLLPLAI	34	10	93	10	382	42	6.83	4351.3	3769.4	4123.9	4226.1	4171.8	4422.2	3868.4	4107.9
A0A0F7G8.	Plasminoge	MDHKEVVI	9	9	213	1	809	90.6	7.36								
Q8TCZ8	Apolipopro	RLGADMED	54	3	161	1	57	6.7	9.98	476.4	343.4	456.2	890	571.4	559.4	698.9	380.9
P11226	Mannose-b	MSLFPSLPL	43	9	50	9	248	26.1	5.49	2010.3	1675.5	1836.5	1840.5	1926	1449.9	1492.7	1549.9
P14780	Matrix met	MSLWQPLV	18	11	38	11	707	78.4	6.06	1945.1	1718.1	1892.8	1825.1	1878.1	1733.5	1533.9	1462.7
Q9NZP8	Compleme	MPGPRVW	25	9	49	2	487	53.5	7.2	2606.4	2149.6	2431	2787.5	2422.7	2219.6	2302	2150.2
A8K3K1	cDNA FLJ78	MCDDEETT	31	9	48	2	377	42	5.39	82.1	62.4	92.7	91.6	73.8	70.5	64.1	62.2
P35527	Keratin, typ	MSCRQFSS	13	8	64	7	623	62	5.24	4828.9	4184.2	4681.3	4297.7	4160.6	3806.3	3832.4	3567.9
A8KAJ3	cDNA FLJ77	MLKALFLTN	24	10	39	10	493	54.6	5.14	1837.5	1648.2	1889.4	1779.4	1797	1394.9	1262.5	1343.2
Q9UHG3	Prenylcyste	MGRVVAEI	22	9	36	9	505	56.6	6.18	1623	1448	1676.7	1553.9	1683.2	1543.2	1253.5	1396.4
P10720	Platelet fac	MSSAARSR	38	3	102	1	104	11.5	9.1								
S6B2A1	IgG L chain	MVLQTQVI	23	3	107	1	184	20.4	5.52	16.1	15.7	18.2	14.9	14	20.7	13.1	15.4
B0YIW2	Apolipopro	MGTWGAF	47	4	206	4	117	12.8	8.18	15946.8	14299.1	16740.7	12479.1	14347.2	18360.1	14244.5	15931.1
Q13790	Apolipopro	MTGLCGYS	8	2	52	2	326	35.4	5.64	2383.7	2210.3	2269.3	2033.8	2229.3	2090	1990.1	2324.8
P35908	Keratin, typ	MSCQISCKS	19	10	39	5	639	65.4	8	1259.7	955	943.1	1154.5	1302.1	818.6	752.4	701.5
A0A024RD	Lymphocyt	MARGSVSD	18	10	31	10	627	70.2	5.43	1464.3	1267.8	1430.2	1427	1398	1284.8	1092.9	1247.4
P40197	Platelet gly	MLRGTLLCA	18	8	31	8	560	60.9	9.63	992.6	1028.8	1166.6	1082.7	1030.5	1056.7	824.5	882
P16070	CD44 antig	MDKFWWH	6	5	44	5	742	81.5	5.33	3521.4	3226.7	3652	3836.3	3838	3254.7	2930.4	3109.9
A0A024R93	Proteoglyca	MAWKTLPI	11	15	34	7	1404	151	9.5	4220.7	3217.9	3556.5	3585.4	3689.6	3751.3	3316.8	3044.2
A2KBC2	Anti-(ED-B)	EVQLLESGC	21	4	45	2	238	25.2	7.71	1521.9	1577	1624.3	1472.1	1518.2	1839.6	1784.3	1576.5
A5YAK2	Apolipopro	MSLLRNRL(	30	5	79	3	127	14.6	9.13	4272.5	3741.9	4575.6	4034	4428.9	4371	4010.6	3983.5
G3V2W1	Protein Z-d	MSRSTQEL	17	8	47	8	484	55.1	7.64	2292.1	2045.7	2003.8	1918.6	2069.7	2209.2	2075.1	1874.1
A0A140VK	Testicular s	MGPPGSPV	14	5	40	5	440	49.5	6.11	2812	2487.7	2736.8	2375.6	2635.4	2525.6	2230.7	2477.8
O43866	CD5 antige	MALLFSLILA	33	8	25	8	347	38.1	5.47	1335.5	1384.5	1606.8	1391.1	1358.8	1626.1	1351.8	1243.5
P01591	Immunoglo	MKNHLLFW	34	5	58	5	159	18.1	5.24	3113	3322.8	3143.9	3267.3	3614.4	3594.7	3742.6	3550.8
A8K3I0	cDNA FLJ78	MVPDTACV	13	7	17	6	757	82.8	4.59	652.6	540.7	621.8	590.1	595.1	527.4	506.8	549.7
Q9H804	cDNA FLJ14	MPGPRVW	21	7	38	1	438	48	7.46	188.7	150.8	254.6	222.8	105.8	138.6	212.9	156.4
B0YJC6	Vitamin K-c	MAGCVPLL	21	7	21	7	421	46.9	6.04	1103.8	1108.9	1160.9	1103.9	913.5	890.5	832.8	933.3
Q6UXB8	Peptidase i	MHGSCSFL	11	5	28	5	463	49.4	5.39	905.3	840.5	932.9	822	835.3	645.3	631.8	818.1
B3KWB5	cDNA FLJ42	MAPVSWIT	31	6	95	1	345	37	5.62								
P08709	Coagulatio	MVSQALRL	14	4	14	4	466	51.6	7.23	407.9	288.2	410	429.3	361	314.7	263.8	328
V9HWB4	Epididymis	MKLSLVAA	12	7	17	7	654	72.3	5.16	779.4	658	736.6	713.6	670.1	707.2	577.6	597.3
P02741	C-reactive	MEKLLCFLV	24	6	38	6	224	25	5.63	830.7	1080.4	1074.1	1604.3	1017.9	930.1	4798.1	1082.9
Q9UL72	Myosin-rea	EVQLVESG(	25	2	40	1	118	12.9	6.58	481.2	387	447.7	412.4	424.7	408.7	401.4	364.5
P49908	Selenoprot	MWRSLGLA	14	6	50	6	381	43.2	7.87	3795	3274.3	3369.5	3224.2	3638.3	3596.3	2561.1	3441.9
V9HW12	Epididymis	MASGNARI	37	7	28	7	198	21.9	5.97	1592.7	1603.5	1412.8	1548.9	1461.7	1574.2	1641.4	1538.3
P07359	Platelet gly	MPLLLLLLLI	12	6	55	6	652	71.5	6.29	2648.4	2443.9	2770	2679.9	2718.9	2386.6	2117.4	2084.5
D3DQH8	Secreted p	MHFMCDL	17	5	27	5	315	36	4.88	2082.9	1926.3	2272.4	2198	1998.8	1964.4	1970.9	2098

P08779	Keratin, typ	MTTCSRQF	16	9	45	3	473	51.2	5.05	1891.4	1570.9	1774.5	1739.5	1762.6	1580.2	1378	1452.8
P61626	Lysozyme (	MKALIVLGI	41	4	25	4	148	16.5	9.16	1304.7	1215.2	1329.5	1310.4	1321.3	1230.1	1218.8	1103.7
A0A024R8	L-selectin (	MGCRRTRE	16	5	21	5	385	43.6	7.15	1334.2	1161.3	1309.8	1228.2	1343.6	1204.4	1086.6	1228.5
S6BAQ4	IgG H chair	MEFGLSWV	16	3	27	1	236	25.3	8.44								
Q59E93	Membrane	GSLSRLPTIT	10	8	19	8	977	110.5	5.53	515.6	400.2	502.8	474.2	465	389	363.5	406
A0A193CH	10E8 heavy	EVQLVESG(	28	3	26	1	131	14.4	7.96	158.8	163.2	155.1	171.8	181.3	163	182.6	157.8
Q15166	Serum para	MGKLVALV	16	6	172	4	354	39.6	5.41	388.4	362.1	443.9	424	404.1	387.6	306.9	355.2
B2RA39	cDNA, FLJ9	MLLLFSVILI	12	6	20	5	569	64.3	7.06	981	877	968.8	973.7	916.8	847.9	950.7	781.4
P0DJI9	Serum amy	MKLLTGLVI	25	4	16	2	122	13.5	9.14	332.3	257.1	325.2	329	287.9	283.8	467.6	294.1
P0DJI8	Serum amy	MKLLTGLVI	41	4	22	2	122	13.5	6.79	384.7	312.6	424.2	400.2	334.5	316.6	683.4	389.3
A0A068LKI	Ig heavy ch	QVQLVESG	33	3	12	1	125	13.9	9.01								
Q15582	Transformi	MALFVRLLA	11	7	25	7	683	74.6	7.71	1217.3	1181.9	1282.9	1272.7	1157.6	1193.1	1078.2	1222.9
A0A024R8	Prostaglan	MATHHTLV	21	3	33	3	190	21	7.8	868.5	780.4	887.8	877.6	863.8	666	610.6	727.8
O00187	Mannan-bi	MRLLTLLGL	10	6	35	6	686	75.7	5.63	2314.3	2064	2289	2051	2010.4	1745.3	1793.4	1989
Q65ZC9	Single-chai	QVQLVQSG	17	3	25	1	240	25.6	9.11								
P18065	Insulin-like	MLPRVGCP	22	5	11	5	325	34.8	7.5	411.4	349.1	408.5	403.6	332.6	324.6	270.7	301.5
A0A140TA	Tenascin-X	MMPAQYA	3	10	14	10	4242	458	5.17	402.7	380.1	427.2	385.6	348.8	371.3	319.7	362.7
P18428	Lipopolysa	MGALARAL	9	4	26	4	481	53.4	6.7	1117.8	1038.8	1148.7	1059.3	991.4	1082.1	1446.9	996.7
A0A1B1CY	Vitamin D I	LAERLKAKL	38	2	104	2	34	3.7	8.56	10328.3	9334.3	10642.8	10623.2	7147.6	8648.4	3808.9	10424.2
A2NYQ9	Anti-folate	QVQLVESG	32	3	16	1	120	13.3	8.44	45.6	42	53.7	41.3	43.9	53.3	44.4	53.9
Q9Y5Y7	Lymphatic	MARCFSLV	11	4	24	4	322	35.2	8.28	1481	1276.5	1382.1	1324.6	1456	1268.8	964.9	1364.9
P02671	Fibrinogen	MFSMRIVC	5	4	11	4	866	94.9	6.01	989.7	850.9	726.5	790.5	773.6	947.4	939.9	771.5
P02533	Keratin, typ	MTTCSRQF	16	8	40	2	472	51.5	5.16	86.1	62.7	80	78.1	73.8	79	51	55.3
Q6EMK4	Vasorin OS	MCSRVPLLI	6	3	13	3	673	71.7	7.39	507	411	474.9	446.2	485.5	398	338	363.3
A0A0S2Z42	HCG20398	MASTSTTIF	9	6	20	3	564	60	8	557.3	425.9	473.6	446.2	492.1	391.6	358.9	406.5
B7Z4R8	cDNA FLJ53	MAWKTLPI	11	9	23	1	853	89.5	8.91								
B3KUE5	Phospholip	MGLSGSDN	9	4	16	4	513	56.6	6.64	1026.7	1001.6	1132.5	1000.6	1019.8	992.1	662.1	901.5
A2NB46	Cold agglut	EIVLTQSPG	25	2	19	1	109	11.9	9.01	438.5	393.2	457.3	431.9	398.7	419.1	400.9	397.6
B2R773	cDNA, FLJ9	MLLLGAVLI	12	2	6	2	244	26.4	5.74	835.1	1129.4	751.6	746	632.2	663.9	539.3	546.2
A8K6K4	cDNA FLJ77	MTLLWCVV	7	4	18	4	570	65.4	8.22	1248.6	1077.3	1346	1536.6	1482.1	1344.5	1075.5	1308
A0A087WX	Cadherin-1	MGPWSRS	3	2	6	2	903	100	4.79	177.2	154.2	206.2	194.4	167	155.5	132.6	167.8
A8K6A6	cDNA FLJ78	MGLPRLVC	11	6	13	6	646	71.6	5.67	759.3	704	749.6	701.3	759.5	878.3	610.8	640.8
B3KQF4	cDNA FLJ90	MAPFEPLA	19	3	13	3	207	23.2	8.1	222.9	170.1	180	186.7	152.8	165.9	169.3	166.7
A8K430	Fructose-b	MAHRFPAL	21	5	9	5	364	39.4	8.21	159.5	123.1	169.1	169.2	125.7	152.2	103.7	111.1
A0A0K0K1	Cystatin OS	MAGPLRAF	31	3	19	3	146	15.8	8.75	842.1	825.4	805.6	821	888.1	813	778.2	801.8
A8K335	cDNA FLJ76	MASPGRLL	15	4	20	4	318	36	7.42	727.5	654.2	707.8	763.3	715.1	602	560	626.5
Q9NQ79	Cartilage a	MAPSADPC	7	5	13	5	661	71.4	5.12	908.4	874.4	1227.9	829.9	874.3	746.8	679.4	792.2
Q15485	Ficolin-2 O	MELDRAVG	15	6	19	6	313	34	6.77	1636.6	1319.3	1375.2	1231.1	1469.1	1458.5	1153.2	1232.5
B4E0X1	Beta-2-mic	MSRSVALA	19	3	14	3	122	13.9	7.44	1324.5	1277	1268.4	1283.3	1219.7	1453.9	1226.4	1181.8
P05362	Intercellula	MAPSSPRP	9	4	8	4	532	57.8	7.99	238.6	210.4	221.3	230.1	208.3	214.7	218.3	202.9
Q76LX8	A disintegr	MHQRHPR	4	5	9	5	1427	153.5	7.17	543.8	327.7	425.6	395.9	310.8	349.8	260.2	311.2
A0A075B7	Protein IGH	MEFELSWV	19	2	21	0	117	12.9	6.51								

P21333	Filamin-A (	MSSSHSRA	2	4	8	4	2647	280.6	6.06	169.3	148	168.1	169.7	144.1	141.4	110.8	123.6
Q92496	Compleme	MLLLINVIL	13	5	24	5	578	65.3	4.94	1447.6	1316	1489.3	1467.8	1465.4	1237.4	1561.3	1422.9
A0A024RD	Phospholip	MVPPKLHV	8	3	8	3	441	50	7.56	565	425.7	544.9	555.5	541	451.2	424.7	431.5
Q86VB7	Scavenger	MSKLRMVI	5	4	7	4	1156	125.4	5.95	208.9	181	222.9	208.4	189.1	157.1	166.1	167.3
P07737	Profilin-1 C	MAGWNAY	27	3	12	3	140	15	8.27	387.8	353.3	350.2	331.5	356.3	337.3	253.1	247.6
P37802	Transgelin-	MANRGPA	30	5	14	5	199	22.4	8.25	690.4	625.6	751.4	676.2	673.6	605.5	526.5	445.5
Q13103	Secreted p	MISRMEKN	27	5	11	5	211	24.3	8.32	285.6	310.5	260.6	242.5	241.1	256.7	215.2	286.8
B1AHL2	Fibulin-1 O	MERAAPSR	7	4	8	4	721	78.3	5.39	239.3	197.7	215.8	222.6	261.5	166	171.8	181.8
Q59EA3	Cadherin 5	TDRQSNGT	7	5	13	5	807	89.9	5.48	558.2	497.9	596.1	503.1	496.3	329.5	293.5	314
Q0IIN1	Keratin 77	MSHQFSSC	5	3	24	1	578	61.8	5.85								
Q16853	Membrane	MNQKTILV	9	5	9	5	763	84.6	6.52	129.3	94.8	137.7	148	126.3	94.6	76.7	99.9
P02745	Compleme	MEGPRGW	9	2	37	2	245	26	9.11	2073.6	2290.7	2089.9	2080.8	2050.6	2014.3	2287.5	2193.5
A0N5G5	Rheumatoi	EIVLTQSPA	23	2	9	2	118	12.8	8.97	136.1	97.1	132.6	124.5	110.3	101.2	108.4	94.5
Q9HCC1	Single chai	EVQLVESG	37	3	8	1	112	12.2	8.47	31.1	15.9	22.6	19.8	18.6	20.8	18.8	19
Q9Y490	Talin-1 OS=	MVALSLKIS	2	5	6	5	2541	269.6	6.07								
P33908	Mannosyl-	MPVGGLLP	7	4	11	4	653	72.9	6.47	388.2	349.4	419.1	421.2	385.4	302.6	327	296.9
A8K6C9	cDNA FLJ78	MGIPMGK	14	2	22	2	180	20.2	9.32	1228.8	985.8	1209.8	986.1	1181.6	934.7	818.7	934.1
A0A087WT	Neural cell	MLQTKDLI	6	5	14	5	848	93.3	4.87	1126.6	994.6	1094.7	1013.7	997.6	1054.1	759.1	888.9
V9H1C1	Gelsolin ex	PNSMVVEH	35	3	48	1	69	8	5.78								
Q9HDC9	Adipocyte	MSEADGLR	10	4	16	4	416	46.5	6.16	696.8	716.8	782.3	793.5	763.3	642.7	620.9	668.2
A0A068LL6	Ig heavy ch	QVQLVQSG	19	2	16	1	120	13.3	7.93	55.8	60.9	56.5	48	77.1	57.8	80.2	59.3
O00533	Neural cell	MEPLLLGR	4	5	13	5	1208	135	5.76	401.8	325.1	460.4	348.5	365.9	330.3	276.8	308.4
P35443	Thrombosp	MLAPRGA	3	2	6	1	961	105.8	4.68	47.3	37.8	54.5	40.4	45.5	37.1	36.5	35.7
P23470	Receptor-t	MRRLLEPC	4	4	5	4	1445	161.9	6.42	180.8	144	201.5	200.5	186.1	134.1	104.6	147.9
E9PR17	CD59 glyco	MGIQGGS	15	2	5	2	130	14.5	7.77	191.7	175.9	212	214.1	197.9	177	156.6	192.7
B2R9V7	Superoxide	MLALLCSCL	12	2	5	2	240	25.9	6.79	123.8	106.3	144.8	116.1	109.3	109.1	73.1	103
A0A125QY	GCT-A9 ligh	DIQMTQSP	32	2	5	2	107	11.6	4.65	97.4	98.7	124.6	111.1	94.1	115.4	270.5	100.7
V9HW31	ATP syntha	MLGFVGR	4	2	4	2	529	56.5	5.4	149.2	140.3	154.3	152	138.6	89.4	122.9	83.6
M9MML0	Fc of IgG lo	MGGGAGE	11	4	7	4	290	32.7	8.1	649.1	555.7	596.8	601.8	549.8	534.9	510.7	525.7
Q13201	Multimerir	MKGARLFV	3	3	12	3	1228	138	7.93	578.8	527.2	603.9	553.1	542.8	563.8	501.9	484.6
P05164	Myelopero	MGVPFFSS	7	5	7	5	745	83.8	8.97	216.2	219.4	213.8	211.2	238.4	216	165.9	150.6
B2R7D2	cDNA, FLJ9	MLRAPGCL	7	3	6	3	487	55.1	7.97	201.9	179.8	203.8	180.4	175.6	167.6	145.5	180
G9K388	YWHAE/FA	RADPAGAA	5	2	5	2	384	41.2	4.97	382.4	307.1	367.6	384.3	322.6	149.6	340.3	139.1
A0A125QY	IBM-B2 hea	EVQLVESG	13	1	8	1	123	13.6	7.12	351.7	286.3	377.4	331.4	326.2	328.2	296.8	338.5
A0A125QY	GCT-A7 hea	QVQLVQSG	21	2	16	1	119	13.1	7.94								
Q8NBP7	Proprotein	MGTVSSRR	5	3	4	3	692	74.2	6.61	190.5	139.5	186.7	178	184.5	130.9	92.9	138.5
A4D2D2	Procollager	MLPAATAS	12	4	7	4	449	47.9	7.43	157.4	139.2	137.5	118.9	136	122.5	96.7	134.5
P07195	L-lactate de	MATLKEKL	15	4	10	4	334	36.6	6.05	219	207.3	205.5	176	197.2	192.4	191.6	187.3
E7EQ64	Trypsin-1 C	MNPLLILTF	11	2	20	2	261	28.1	7.25	485.9	434	340.8	333.8	442.1	322.1	229.1	329.5
Q9UL85	Myosin-rea	EIVMTQSP	25	2	14	2	109	11.8	8.51	1238.1	1267.8	1233.3	1110.3	1252.6	1142.2	1319.2	1213.3
P19320	Vascular ce	MPGKMVV	5	3	4	3	739	81.2	5.22	165.9	124.6	135.8	129.9	127.7	132.7	109.2	119.6
A0A140VJF	Testicular t	MAAATGPS	7	3	3	3	493	54.5	6	133.4	108.5	133.7	124.3	118.2	114.2	84.4	106.7

P05019	Insulin-like	MGKISSLPT	7	1	4	1	195	21.8	9.72								
Q15430	Coagulatio	PPESTVMA	9	2	13	1	236	26.1	5.48								
A2J1N5	Rheumatoi	VESGGGVV	23	2	5	1	94	10.4	9.13	128.6	126.1	218.9	147	127.4	123.3	107.3	112.9
Q07954	Prolow-der	MLTPPLLLL	1	2	4	2	4544	504.3	5.39	23.3	22.5	29.5	30	27.7	25.7	31.1	22.3
P24592	Insulin-like	MTPHRLLP	10	2	5	2	240	25.3	7.81	139.1	119	147.8	160	139.5	141	102.9	120.4
B2RBF5	cDNA, FLJ9	MSRPQLRR	9	3	3	3	385	43.8	6.64	110	98.6	119.6	98.5	117	103.3	121.3	95.9
P12111	Collagen al	MRKHRHLF	1	3	6	3	3177	343.5	6.68	112.8	84.9	109	100.6	97.6	87.7	70.1	83.9
B7Z9B1	cDNA FLJ52	MKTPPGAS	2	1	4	1	760	83.4	5.12	213	182	223.4	245.4	200.6	175	158.9	183.5
B2R4R0	Histone H4	MSGRGKG	17	2	10	2	103	11.4	11.36	875.9	676.1	749.1	753.8	704.1	320.6	564.9	266.4
A8K6C1	cDNA FLJ76	MLAATVLT	5	2	5	2	493	54.7	6.09	730.8	691	887	885.6	756.5	766.5	666.3	679.2
A0N5G1	Rheumatoi	DIQMTQSP	25	2	4	2	116	12.5	8.46	293.3	244.9	279.1	241.5	264.9	263.1	246.1	236.4
X6RBG4	Uromoduli	MRHIMTRL	4	2	3	2	689	75.6	5.87	213.9	141	221.9	221.6	205.7	147.3	149.8	174.1
P49746	Thrombosp	METQELRG	3	3	17	2	956	104.1	4.65	765.2	612.7	777.6	651.6	854.1	638.2	558.4	626.2
A0A0X9UW	MS-D4 hea	EVQLVQSG	10	1	15	1	122	13.5	7.12	710.5	592.1	723.2	664	740.1	668.3	688.6	644.4
P00918	Carbonic ar	MSHHWGY	12	3	5	3	260	29.2	7.4	163.1	160	141.6	152	162.7	170	149.8	122.8
P08253	72 kDa typ	MEALMAR	2	1	2	1	660	73.8	5.47								
J3KPA1	Cysteine-ri	MKQILHPA	9	3	13	3	276	31	7.61	2029.3	1726.2	1542.2	1556.9	1752.8	1671	1279.2	1673.4
P22692	Insulin-like	MLPLCLVA	11	2	3	2	258	27.9	7.15								
Q4ZG40	Macrophag	MRNKKILKI	3	1	2	1	520	52.6	8.78	200.9	143.7	187.4	181.2	159.4	157.2	131.7	171.9
E2RVJ0	Anion exch	MEELQDDY	3	2	4	2	912	101.9	5.22	45.7	41.6	51.8	36.3	32.8	41.9	22.8	23.2
A6NC48	ADP-ribosy	MAAQGCA	7	2	5	2	333	37.5	7.97	275.1	235.9	310.2	251.1	259.8	236.7	225.3	236.2
Q6MZL2	Putative ur	NPGIYTCSA	6	2	10	2	321	35.1	5.97	701.4	643.4	639.9	593	599.9	603.8	543.8	569.9
P04040	Catalase OS	MADSRDPA	7	3	5	3	527	59.7	7.39	325.4	234.3	271	208.5	178	199.2	194.1	182.4
D3YTG3	Target of N	MRGGKCN	1	2	4	2	1777	195.2	9.73	157.3	163.3	221.3	176.3	176	133.7	123.8	153.2
Q9UL89	Myosin-rea	VQSGAEVK	10	1	2	1	116	12.6	9.1	83.3	66	98.2	70.9	86	68.2	64.1	74.9
X6R8F3	Neutrophil	MPLGLLWL	14	2	6	2	200	22.8	8.5	369.7	353.7	377.1	359.3	354	316.2	289.9	264.9
A0A024RD	SPARC-like	MKTGLFFL	2	1	3	1	664	75.2	4.81	108.7	78	88.3	113.9	86.9	81.4	62.4	67.4
P35916	Vascular er	MQRGAAL	2	1	1	1	1363	152.7	6.3	45.6	35.5	48.1	50.6	34.5	40.7	31.2	44.5
B7ZMD7	Alpha-amy	MKLFWLLF	3	1	1	1	511	57.7	6.93	56.2	35.9	53.3	50.1	44	49	42.1	35.3
J3KPS3	Fructose-b	MPYQYPAL	8	2	2	2	368	39.8	8.09	197.8	164.3	206.9	205.3	173.7	144.9	133.8	133.9
Q14126	Desmoglei	MARSPGRA	2	2	2	2	1118	122.2	5.24								
P23284	Peptidyl-pr	MLRLSERN	6	1	10	1	216	23.7	9.41								
Q8WZ75	Roundabou	MGSGGDSI	2	1	3	1	1007	107.4	6.64	221.9	170.2	207.2	220.8	185.4	179.8	158.2	152.6
A0A087X1I	Calcium-de	MLDPSSSEI	1	1	49	1	1301	148.2	6.05	326.4	282.7	391.4	337.5	297.2	291.3	284.7	298
A0A0B4J1Y	Immunoglo	MEFGLSW	15	2	4	1	119	13.2	7.85								
Q86YW5	Trem-like t	MGLTLLLLL	5	1	2	1	311	32.7	6.05	143.9	116.3	143.2	132.4	126.9	121.4	99.9	115.4
A0A024R9.	Collectin su	MNGFASLL	12	3	4	3	277	30.7	7.33								
Q6UY14	ADAMTS-li	MENWTGR	2	1	1	1	1074	116.5	8.34	62.8	54.2	65.4	56.2	53.3	47	39.6	42.9
Q6UX71	Plexin dom	MARFPKAD	2	1	4	1	529	59.5	6.46	226.6	197.4	227.5	181.4	199.7	211.7	150.5	179.8
P03950	Angiogenir	MVMGLGV	18	2	3	2	147	16.5	9.64	420	390.8	489.1	518.6	472.9	389.7	343.2	363.2
Q05639	Elongation	MGKEKTHI	2	1	2	1	463	50.4	9.03	127.6	111.4	84.2	85.3	136.9	56.2	104.1	40.3
Q59ED3	Intercellula	LIVTCSLQQ	4	1	2	1	344	38.4	7.78	87.6	66	99.7	102.8	70	66	56.5	68.1

Q8NGE6	Olfactory r	MGDKGTG	6	1	16	1	254	28.1	7.91	1274.4	1087.1	1695.4	1970.5	1579.6	898.4	1144.2	1201.7
P07333	Macrophag	MGPGVLLL	3	2	3	2	972	107.9	6.37	52.1	37.1	42.2	44.6	33.9	37.4	31.8	32.6
Q9H8L6	Multimerin	MILSLLFSL	2	2	3	2	949	104.3	5.86	227.9	155.2	220	188.5	186.5	178.5	142.2	160.8
P04066	Tissue alph	MRAPGMR	5	2	4	2	466	53.7	6.84	152.2	118.8	179.1	166.6	134.4	125	104	124.2
A8K7T4	cDNA FLJ75	MAAEGWI	4	1	1	1	356	40.2	6.95								
A0A140VJC	Testicular t	MLPGLALL	4	3	4	3	770	86.9	4.82	139.9	137	167.7	161.9	160.1	141.1	137.8	134.5
A0A1C9J6F	B cell recep	EVQLVQSG	14	1	2	1	132	14.6	8.02	87.8	61.3	96.2	87.2	86.2	79	58.1	61.6
B4DZM1	cDNA FLJ58	MTKLPDDP	2	1	16	1	872	100.5	5.02	1136	998.7	907.2	924.7	1086.3	1158.3	1028.7	1025.2
A0A024R6Y	Dipeptidas	MQPSGLEG	3	1	1	1	486	53.3	6.54								
Q4LE64	NUMA1 va	LSGITKMTL	1	1	45	1	2121	238.7	5.81	1029.2	821	1000.5	1075	1117.1	684	571.4	802.5
P11279	Lysosome-a	MAAPGSAF	4	2	3	2	417	44.9	8.75	315.2	278.2	311.7	266.4	250.5	229.6	221.9	238.8
Q99784	Noelin OS=	MSVPLLKIG	4	2	3	2	485	55.3	6.95	230.4	224.4	214.6	214.5	211.6	203.3	219.5	236.4
Q9UIU0	Dihydropyr	MAAGCLLA	2	2	4	2	1110	125.2	5.36	86.9	72.3	95.2	65.3	68.7	75.9	56.5	64.3
P07998	Ribonuclea	MALEKSLVI	15	1	2	1	156	17.6	8.79	36.7	32	38.4	41.7	42.1	36.3	29.5	34.2
K4DIA0	ICOS ligand	MRLGSPGL	2	1	7	1	473	51.9	8.05	570.7	574.2	552.6	517.5	534.7	498.4	476.9	522.3
Q9ULV0	Unconvent	MSVGELYS	0	2	8	2	1848	213.5	7.2	63.5	46.6	53.9	82.8	56	64.5	84.6	53.7
O14786	Neuropilin-	MERGLPLL	1	1	1	1	923	103.1	5.88								
B7Z3G0	cDNA FLJ58	MSARRTSH	2	1	16	1	833	94.1	6.87	219.8	169.4	259.1	320.3	272.7	313.1	191.9	295.9
Q99969	Retinoic ac	MRRLLIPLA	7	1	2	1	163	18.6	9.09	55.7	50.7	68.1	56.8	50.2	45.7	42.4	40.7
Q9HCI6	E3 SUMO-p	MGDPGSEI	1	1	6	1	787	89.6	7.74	303.5	614.6	350.2	420.3	379.6	224.3	259.3	247.6
A0A087WV	Receptor-ty	MKPAAREA	2	2	2	2	1342	146.5	5.62	75	66.9	70.1	55	58.1	70	55	58
P67936	Tropomyos	MAGLNSLE	8	2	4	2	248	28.5	4.69	474	453.2	532.2	517.2	458.3	433.9	343.7	328.1
A0A024RCV	Peroxisome	MEQPQEEA	2	1	5	1	441	49.9	7.59	477.6	483.8	540.7	509.1	603.2	356.8	456.9	446.8
P98160	Basement r	MGWRAAG	1	2	3	2	4391	468.5	6.51	180.2	126.8	126.6	132.4	138.4	159.1	95.7	91.9
A0A0S4XQ	MHC Class	MAVMAPR	4	1	1	1	365	40.8	6								
Q8IY21	Probable A	MERNVLTT	1	1	12	1	1712	197.7	7.59	976.1	858.9	1005.7	930.4	1047.1	730.4	754.9	795
A0A0U1RR	Histone H2	MSGRGKQ	9	2	2	2	170	18.5	11.52	257.8	229.1	272.6	266.6	263.5	232.6	186	218.4
G3XAI2	Laminin su	MDCPLSAP	1	1	1	1	1810	200.3	4.96	118.5	108.6	150.7	133.7	113.4	107.6	80.6	99.6
C9JC84	Fibrinogen	MSWSLHPF	3	1	2	1	461	52.3	5.63								
Q9H7P6	Multivesicu	MRSCFCVR	3	1	13	1	319	35.6	8.15								
B7Z351	Secreted p	MGIVPRSLI	5	1	1	1	327	37.2	4.73	47.7	36.1	40.2	46.4	41.8	46.3	34.7	40.3
Q9BWP8	Collectin-1	MRGNLALV	6	2	2	2	271	28.6	5.41	36	28.1	40.2	40.5	40.3	21.1	16.9	18.3
A0A024QZ	Serine/thre	MELLRTITY	2	1	3	1	685	78.2	8.24	297.4	286.2	380.1	538.4	430	246.8	223.6	359.7
A0A024QZ	Proteoglyc	MMQKLLK	8	1	2	1	158	17.6	4.86	161.4	127.2	176.4	128.5	136.2	156.7	108.9	132.4
A8K9X5	cDNA FLJ76	MASNMDR	2	1	10	1	650	73.9	4.88	1183.1	1057.9	1665	1951.7	1903.3	2038.2	880.6	1282.7
P11717	Cation-inde	MGAAAGR	0	1	1	1	2491	274.2	5.94								
A0A024R2Y	Dystroglyca	MRMSVGL	2	1	1	1	895	97.5	8.56								
D6W5L6	Pulmonary	MHQAGYP	3	1	2	1	393	43.3	5.74	35.8	23.6	32.4	34.2	28.6	30.3	38.6	27.1
P20023	Compleme	MGAAGLL	1	1	1	1	1033	112.8	7.52								
A0A060VC	MHC class	SHSMRYFF	5	1	9	1	340	38.5	6.15	990.7	1154.2	874.8	858.5	929.5	1310.8	899.2	1005.6
A8K2T7	Receptor p	MRPSGTAG	1	1	2	1	1210	134.1	6.7								
A0A024R1	Contactin 1	MKMWLLV	1	1	2	1	1018	113.2	5.9	50	47.3	56.2	40.4	46.9	43.8	29.5	41.9

Q9BTY2	Plasma alp	MRPQELPR	2	1	6	1	467	54	6.25	282.1	242.6	252.4	280.7	273.7	242.1	220.1	237.3
Q8IXL6	Extracellula	MKMMLVF	3	1	1	1	584	66.2	7.74	88.7	82.9	112.4	78.9	103.9	92.9	48.6	121.2
A0A075B6I	Dynein hea	MEKDAED(	0	1	6	1	4707	538.3	6.13	147.4	89.2	150.5	147.8	144.5	106.1	77	130.9
A0A087WX	IgGFc-bind	MGALWSW	0	1	1	1	4204	444.9	5.35	310.1	245.6	327.3	268	250	265.7	235.5	260.4
Q99727	Metallopro	MPGSPRPA	6	1	16	1	224	25.5	7.8	93.4	109.7	130.5	142.1	111.3	116.6	118.5	135
Q0ZCH6	Immunglob	LVQLVESG(	7	1	1	1	131	14.3	6.55	85.4	69.3	100	97.9	77.4	76.1	67.1	71.2
Q9UNN8	Endothelia	MLTTLLPILI	4	1	3	1	238	26.7	7.18	146.2	153.2	181.9	174.8	162.8	146.9	150.4	148.8
A0A0C4DG	Poliovirus r	MARAMAA	2	1	2	1	417	45.3	6.52	336	313.3	359.9	385.4	333.7	411	253.4	336.2
J3KNB4	Cathelicidi	MGTMKTQ	5	1	1	1	173	19.6	9.41	351.3	340.1	421	395.6	385.8	382.4	289.6	316.2
Q96RZ2	N-acetylglu	MAAGLARI	3	1	1	1	331	36.8	6.79	70.6	65.6	59.8	47.2	49.8	40	42.2	45
Q4LE33	TNC varian	SLLCASKSP	1	1	1	1	2233	244.2	4.94								
A8K486	Peptidyl-pr	MVNPTVFF	5	1	1	1	165	18	6.9	24.4	19	13.1	15.1	13.1	13.8	8.2	9.5
B2R6V9	cDNA, FLJ9	MSETSRTA	2	1	1	1	732	83.2	6	69.4	56.9	69.2	56.4	69.9	60.9	37.2	52.7
Q16706	Alpha-man	MKLSRQFT	1	1	2	1	1144	131.1	7.58								
H3BQD0	Cerebellin-	MLGVLELLI	11	1	2	1	140	14.7	5.48	13.6	18.9	16.2	12	15.3	18.1	12.7	13
Q86U17	Serpin A11	MGPAWLW	2	1	1	1	422	47	7.68	74	58.2	76.4	76.2	78.9	89.4	54	54
A0A140VK	Leukotrien	MPEIVDTC	2	1	1	1	611	69.2	6.18	35.1	29	31.9	27.7	33.2	24	28.4	31.4
A0A140GX	Platelet me	MGSGPRG/	5	1	1	1	206	21.7	8.91	62.2	51.3	81.4	44.4	39.6	41.9	35.7	40.9
Q96S96	Phosphatic	MGWTMRI	4	1	2	1	227	25.7	6.54	34.8	29	43.1	35	33.3	46.8	26.7	32.3
O00151	PDZ and LII	MTTQQIDL	3	1	1	1	329	36	7.02	103.2	93.2	99.7	104.3	97.3	94.3	70.4	78.2
A0A068B0	Cis-AB glyc	MAEVLRTL	5	1	1	1	354	40.8	8.87	92.9	74.9	93.7	95.4	75.6	124.2	72.3	74.1
Q6YHK3	CD109 anti	MQGPPLLT	1	1	1	1	1445	161.6	5.85	197.5	155.1	190.1	219.3	176.1	195.4	149.5	163.1
B3KSS6	Phosphodi	MESPTKEIE	1	1	3	1	709	80.7	8.82	316.3	291.1	329.9	343.6	295.2	417.6	321.5	385.6
B4DQY7	cDNA FLJ51	MSWFNAS	1	1	1	1	992	111.7	4.87	22.3	20.1	20.8	17.9	17.2	20	22.6	15.6
A0A024RD	Periostin, c	MIPFLPMF	1	1	1	1	836	93.3	7.53								
P06733	Alpha-enol	MSILKIHAR	3	1	2	1	434	47.1	7.39	71.8	57.6	92.1	66.6	70	62.9	52.2	49.7
Q86SQ4	Adhesion G	MMFRSDR	1	1	1	1	1221	136.6	7.87								
Q8TER0	Sushi, nido	MRHGVAW	1	1	1	1	1413	152.1	6.98								
A1L4H1	Soluble sca	MRVLACLL	1	1	1	1	1573	165.6	6.13								
Q13740	CD166 anti	MESKGASS	2	1	1	1	583	65.1	6.25	60	43.9	54.1	50.8	48.3	47	37.2	39.8
O94818	Nucleolar p	MESERDM	3	1	1	1	638	71.3	5.62	66.8	54.2	83	70.8	68.8	56.3	44.9	60.9
P06703	Protein S10	MACPLDQ/	9	1	2	1	90	10.2	5.48								
D3DPK5	SH3 domai	KQLKPSKAI	4	1	1	1	257	26.8	8.38	99	106.2	123.2	78.5	81.8	83.4	85	63.5
E9PK25	Cofilin-1 O	MVSPGHGI	5	1	1	1	204	22.7	8.34								
A0A125U0	GCT-A2 hea	EVQLVESG(	15	1	1	1	126	13.8	5.45	24.4	19.9	30.3	26.6	20.8	26.9	15.2	18.9
D6W5K2	Thymosin,	GVSSRFPEF	4	1	1	1	169	17.9	10.48	215	183.4	144.4	169.2	204.7	181.3	76.5	88
Q6PIY7	Poly(A) RN.	MFPNSILGI	2	1	1	1	484	56	9.38	60.9	63.3	61.6	56.5	52.3	61.6	43	67.2
Q96AN5	Transmem	MTVELWLF	3	1	4	1	459	51.7	9.67	156.9	268.8	443.2	179.8	136.5	83.9	93.9	90
Q6P4F1	Alpha-(1,3)	MVRIQRRK	5	1	2	1	479	56.1	8.37								
Q9UL82	Myosin-rea	SYELTQPSS	7	1	1	1	107	11.4	6.6								
Q7Z7M0	Multiple ep	MALGKVLA	1	1	1	1	2845	302.9	6.87								
Q2KHR2	DNA-bindir	MSSSRAQ(	1	1	1	1	1363	146.8	6.76								

Q75KY0	Putative ur	LSGSILDVYS	13	1	1	1	258	29	6.7								
V9HVY1	Epididymis	MKRMVSW	2	1	1	1	491	55.9	8.27	85.5	127.8	209.8	85.8	64.8	51.9	68	51.6
Q59EB6	Compleme	KLLSPLGFSI	2	1	2	1	671	70.4	5.44	66.1	46.1	55.1	58.8	50.3	63.9	59.5	49.4
Q13131	5'-AMP-act	MRRLSSWF	2	1	1	1	559	64	8.12								
Q96RR4	Calcium/ca	MSSCVSSQ	2	1	1	1	588	64.7	6.68								
A0A0R4J2F	Spermatog	MPRAQLLE	1	1	2	1	1361	150	8.81	78.2	53.8	56.5	70.6	68.9	36.4	52.8	22.3
P25391	Laminin su	MRGGVLLV	0	1	2	1	3075	336.9	6.35	83.6	71.2	80.7	80.8	84.7	74	49.1	67.6
A0A0G2JN	Transcripti	MFRRARLS	0	1	5	1	2624	293.7	5.11								
E5RG27	Casein kina	MASSSGSK	17	1	1	1	78	8.5	9.7	125	109	168.1	173.4	152.4	80	88.9	115.8
Q12841	Follistatin-r	MWKRWLA	3	1	1	1	308	35	5.52	34.7	35.6	29.1	27.7	31.4	39.1	31.4	26.6
Q92833	Protein Jur	MSKERPKR	1	1	7	1	1246	138.6	9.38	532.8	542.3	572.6	447.8	479.1	615.9	610.2	606.3
J3KMX3	Alpha-feto	MKWVESIF	1	1	3	1	622	70.4	5.55	292.2	88.4	153.4	117.6	104.8	75.4	46.4	61.9
R4GMU1	GDH/6PGL	MLAEPFNV	1	1	2	1	802	90.1	7.23	63	46.1	62.3	49.4	56.1	53.7	39.6	47.6
Q14766	Latent-trar	MAGAWLR	1	1	1	1	1721	186.7	5.96	34.6	33.9	34.6	32.3	33.2	26.6	38	32.2
A0A068LRV	Ig heavy ch	QVTLRESGI	6	1	4	1	127	14	8.15	84.7	83.7	147	163.1	112.7	102.3	73.3	104.6
K7ELC7	60S ribosor	XRKAVIVKV	14	1	1	1	144	16.5	10.58								
A0A1B0GW	GRAM dom	MVQNLQP	3	1	1	1	767	88.6	6.44	45.3	36	32.6	36.1	41.3	36.9	46.9	33.8
V9HW26	ATP syntha	MLSVRVAA	1	1	2	1	553	59.7	9.13	175.9	164.9	168.9	169.9	162.5	110.7	140.9	117.8
Q9H628	Ras-relatec	MSNFLHLK	4	1	3	1	205	23.8	9.51	209.3	167.6	194	317.1	223.3	295.1	349.6	196
B2RDG0	Proteasom	MSRRYDSR	3	1	2	1	261	29.4	7.72	143.8	116.1	156.9	166.8	127.9	113.5	120.2	67.9
Q8WWZ8	Oncoprote	MPPFLLLTC	2	1	1	1	545	60	5.58	61.3	50.8	61.7	76.5	55.4	45.1	43.2	53.5
Q9Y5Z9	UbiA preny	MAASQVLC	3	1	1	1	338	36.8	8.15	94.4	89.7	109.1	111.7	112	82.5	80.9	81.4
B2R7N9	cDNA, FLJ9	MGFLSPIYV	2	1	1	1	421	49.4	5.52								
A0A024R8	Glucosidas	MGVRHPP	1	1	1	1	952	105.3	6	61.9	56.9	74.9	75	63.9	66.8	42	61.2
Q96JG6	Syndetin O	MQKIKSLM	1	1	4	1	964	111.1	6.2	269.8	285.1	225.2	172.5	241.3	264.5	189.2	264.9
Q66K66	Transmem	MPGTVATL	3	1	5	1	360	39.4	9.92	650.3	714.8	829.3	821.1	623.6	527.9	380.9	522.4
A0A024R7	DNA (cytos	MAGSVPSF	1	1	2	1	1678	189.4	8.02								
Q6ZWJ1	Syntaxin-bi	MNKNTSTV	3	1	2	1	553	61.6	5.2								

Uniprot Accession Number	protein	Protein amino acid sequence	Protein amino acid sequence matching degree(%)	The number of peptides identified	The total number of times a polypeptide was identified	The number of characteristic peptides identified	Amino acid length	KDa	isoelectric point	relative abundance of normalized protein - sample 113	relative abundance of normalized protein - control sample 121	ratio(113/121)
J3KMX3	Alpha-feto	MKWVESIFLIFLL	1	1	3	1	622	70.4	5.55	292.2	61.9	4.720516963
A0A0R4J2F	Spermatog	MPRAQLLESNAF	1	1	2	1	1361	150	8.81	78.2	22.3	3.506726457
B2R4R0	Histone H4	MSGRGKGGKGL	17	2	10	2	103	11.4	11.36	875.9	266.4	3.287912913
Q05639	Elongation	MGKEKTHINIVVI	2	1	2	1	463	50.4	9.03	127.6	40.3	3.166253102
G9K388	YWHAE/FA	RADPAGAAAAM	5	2	5	2	384	41.2	4.97	382.4	139.1	2.749101366
A8K486	Peptidyl-pı	MVNPTVFFDIAV	5	1	1	1	165	18	6.9	24.4	9.5	2.568421053
D6W5K2	Thymosin,	GVSSRFPERGNV.	4	1	1	1	169	17.9	10.48	215	88	2.443181818
B2RDG0	Proteasom	MSRRYDSRTTIFS	3	1	2	1	261	29.4	7.72	143.8	67.9	2.117820324
E2RVJ0	Anion exch	MEELQDDYEDM	3	2	4	2	912	101.9	5.22	45.7	23.2	1.969827586
Q9BWP8	Collectin-1	MRGNLALVGVLI	6	2	2	2	271	28.6	5.41	36	18.3	1.967213115
P98160	Basement	MGWRAAGALLL	1	2	3	2	4391	468.5	6.51	180.2	91.9	1.960826986
P35908	Keratin, ty	MSCQISCKSRGR	19	10	39	5	639	65.4	8	1259.7	701.5	1.79572345
V9HW31	ATP syntha	MLGFVGRVAAAI	4	2	4	2	529	56.5	5.4	149.2	83.6	1.784688995
P04040	Catalase O	MADSRDPASDQ	7	3	5	3	527	59.7	7.39	325.4	182.4	1.783991228
Q59EA3	Cadherin 5	TDRQSNGTETSLS	7	5	13	5	807	89.9	5.48	558.2	314	1.777707006
Q76LX8	A disintegr	MHQRHPRARCP	4	5	9	5	1427	153.5	7.17	543.8	311.2	1.747429306
Q96AN5	Transmem	MTVELWLRLRGK	3	1	4	1	459	51.7	9.67	156.9	90	1.743333333
A0A024R4	Fibronectir	MLRGPGPGLLLL	22	43	227	43	2355	259	5.73	14350.7	8653	1.658465272
V9HVY1	Epididymis	MKRMVSWSFHK	2	1	1	1	491	55.9	8.27	85.5	51.6	1.656976744
Q9HCC1	Single chai	EVQLVESGGGVV	37	3	8	1	112	12.2	8.47	31.1	19	1.636842105
A0A024RD	SPARC-like	MKTGLFFLCLLGT	2	1	3	1	664	75.2	4.81	108.7	67.4	1.612759644
H6VRG1	Keratin 1 C	MSRQFSSRSGYR	26	15	79	13	645	66.1	8.12	3451.6	2159.3	1.598480989
P07333	Macropha	MGPGVLLLLLVAT	3	2	3	2	972	107.9	6.37	52.1	32.6	1.598159509
B7ZMD7	Alpha-amy	MKLFWLLFTIGFC	3	1	1	1	511	57.7	6.93	56.2	35.3	1.592067989
Q96RZ2	N-acetylglı	MAAGLARLLLLL	3	1	1	1	331	36.8	6.79	70.6	45	1.568888889
P07737	Profilin-1 (	MAGWNAYIDNL	27	3	12	3	140	15	8.27	387.8	247.6	1.566235864
D3DPK5	SH3 domai	KQLKPSKAPSFPV	4	1	1	1	257	26.8	8.38	99	63.5	1.559055118
P02533	Keratin, ty	MTTCSRQFTSSSS	16	8	40	2	472	51.5	5.16	86.1	55.3	1.556962025
P37802	Transgelin	MANRGPAYGLSF	30	5	14	5	199	22.4	8.25	690.4	445.5	1.549719416
B2R773	cDNA, FLJ9	MLLLGAVLLLLAL	12	2	6	2	244	26.4	5.74	835.1	546.2	1.528927133
A0A140GX	Platelet me	MGSGPRGALSLL	5	1	1	1	206	21.7	8.91	62.2	40.9	1.520782396
Q13740	CD166 anti	MESKGASSCRLLF	2	1	1	1	583	65.1	6.25	60	39.8	1.507537688
P13645	Keratin, ty	MSVRYSSSKHYSS	19	12	89	9	584	58.8	5.21	6299.9	4187.6	1.504417805
V9HW26	ATP syntha	MLSVRVAAAVVF	1	1	2	1	553	59.7	9.13	175.9	117.8	1.493208829
P10909	Clusterin C	MMKTLLLFVGLL	39	18	653	18	449	52.5	6.27	50095.5	33598.7	1.490995187
J3KPS3	Fructose-b	MPYQYPALTPEC	8	2	2	2	368	39.8	8.09	197.8	133.9	1.477221807
E7EQ64	Trypsin-1 (	MNPLLILTFVAAA	11	2	20	2	261	28.1	7.25	485.9	329.5	1.474658574

Q6UY14	ADAMTS-li	MENWTGRPWL	2	1	1	1	1074	116.5	8.34	62.8	42.9	1.463869464
Q8WZ75	Roundabo	MGSGGDSLLGG	2	1	3	1	1007	107.4	6.64	221.9	152.6	1.45412844
P67936	Tropomyo	MAGLNSLEAVKR	8	2	4	2	248	28.5	4.69	474	328.1	1.4446815
P06733	Alpha-enol	MSILKIHAREIFD	3	1	2	1	434	47.1	7.39	71.8	49.7	1.444668008
A0N5G5	Rheumato	EIVLTQSPATLSL	23	2	9	2	118	12.8	8.97	136.1	94.5	1.44021164
A8K430	Fructose-b	MAHRFPALTQE	21	5	9	5	364	39.4	8.21	159.5	111.1	1.435643564
P05164	Myeloperc	MGVPFFSSLRC	7	5	7	5	745	83.8	8.97	216.2	150.6	1.435590969
B4DQY7	cDNA FLJ5	MSWFNASQLSS	1	1	1	1	992	111.7	4.87	22.3	15.6	1.429487179
A0A1C9J6F	B cell rece	EVQLVQSGGGV	14	1	2	1	132	14.6	8.02	87.8	61.6	1.425324675
P07996	Thrombos	MGLAWGLGVLF	37	39	390	1	1170	129.3	4.94	557.4	392.8	1.41904277
Q9H8L6	Multimerir	MILSLLFSLGGPL	2	2	3	2	949	104.3	5.86	227.9	160.8	1.417288557
X6R8F3	Neutrophil	MPLGLLWLGLAL	14	2	6	2	200	22.8	8.5	369.7	264.9	1.395620989
Q6EMK4	Vasorin OS	MCSRVPLLLPLLL	6	3	13	3	673	71.7	7.39	507	363.3	1.395540875
P19320	Vascular ce	MPGKMVVILGA	5	3	4	3	739	81.2	5.22	165.9	119.6	1.387123746
A0A024R9	Proteoglyc	MAWKTLPIYLLLL	11	15	34	7	1404	151	9.5	4220.7	3044.2	1.386472636
Q8NBP7	Proprotein	MGTVSSRRSWW	5	3	4	3	692	74.2	6.61	190.5	138.5	1.375451264
B2R6W1	cDNA, FLJ9	MKVISLFILVGFIG	48	35	595	2	843	93.5	6.48	467.6	340	1.375294118
A0A0S2Z4	HCG20398	MASTSTTIRSHSS	9	6	20	3	564	60	8	557.3	406.5	1.37097171
Q86U17	Serpin A11	MGPAWLWLLG	2	1	1	1	422	47	7.68	74	54	1.37037037
P21333	Filamin-A (	MSSSHSRAGQS	2	4	8	4	2647	280.6	6.06	169.3	123.6	1.3697411
Q99969	Retinoic ac	MRRLLIPLALWL	7	1	2	1	163	18.6	9.09	55.7	40.7	1.368550369
P63261	Actin, cyto	MEEEIAALVIDN	46	12	73	5	375	41.8	5.48	5034.2	3679	1.368360968
A8KAJ3	cDNA FLJ7	MLKALFLTMLTL	24	10	39	10	493	54.6	5.14	1837.5	1343.2	1.368001787
E9KL23	Epididymis	MPSSVSWGILLL	61	31	1738	1	418	46.7	5.59	8613.5	6306.2	1.365878025
P18065	Insulin-like	MLPRVGCPALPL	22	5	11	5	325	34.8	7.5	411.4	301.5	1.364510779
A8K5T0	cDNA FLJ7	MRLLAKIICLMLV	57	64	2215	1	1231	138.9	6.71	2166.5	1595.6	1.35779644
P35527	Keratin, ty	MSCRQFSSSYLS	13	8	64	7	623	62	5.24	4828.9	3567.9	1.353429188
Q9UIU0	Dihydropy	MAAGCLLALTLT	2	2	4	2	1110	125.2	5.36	86.9	64.3	1.351477449
P12111	Collagen al	MRKHRHLPLVA	1	3	6	3	3177	343.5	6.68	112.8	83.9	1.344457688
A0A1B0GV	GRAM don	MVQNLQPETVS	3	1	1	1	767	88.6	6.44	45.3	33.8	1.340236686
Q59EB6	Compleme	KLLSPLGFSPPAE	2	1	2	1	671	70.4	5.44	66.1	49.4	1.33805668
B3KQF4	cDNA FLJ9	MAPFEPLASGILL	19	3	13	3	207	23.2	8.1	222.9	166.7	1.337132573
P14780	Matrix me	MSLWQPLVLVLL	18	11	38	11	707	78.4	6.06	1945.1	1462.7	1.329801053
P00918	Carbonic a	MSHHWGYGKH	12	3	5	3	260	29.2	7.4	163.1	122.8	1.328175896
Q15485	Ficolin-2 O	MELDRAVGVLG	15	6	19	6	313	34	6.77	1636.6	1232.5	1.327870183
P35443	Thrombos	MLAPRGAAVLLL	3	2	6	1	961	105.8	4.68	47.3	35.7	1.324929972
R4GMU1	GDH/6PGL	MLAEPFNWHPG	1	1	2	1	802	90.1	7.23	63	47.6	1.323529412
D6W5L6	Pulmonary	MHQAGYPGCRG	3	1	2	1	393	43.3	5.74	35.8	27.1	1.32103321
Q8NF17	FLJ00385 p	LWLWGREWAK	23	10	100	2	509	56.1	7.59	320.1	242.4	1.320544554
Q9UL72	Myosin-rea	EVQLVESGGGLV	25	2	40	1	118	12.9	6.58	481.2	364.5	1.320164609
A8K3K1	cDNA FLJ7	MCDDEETTALV	31	9	48	2	377	42	5.39	82.1	62.2	1.319935691
P11279	Lysosome-	MAAPGSARRPLL	4	2	3	2	417	44.9	8.75	315.2	238.8	1.319932998

O00151	PDZ and LI	MTTQQIDLQGP(	3	1	1	1	329	36	7.02	103.2	78.2	1.319693095
E9PFZ2	Ceruloplas	MKILILGIFLFLCST	53	46	2570	1	946	108.8	5.77	305.6	232	1.317241379
B2R6V9	cDNA, FLJ9	MSETSRTAFGGR	2	1	1	1	732	83.2	6	69.4	52.7	1.316888046
B1AHL2	Fibulin-1 C	MERAAPSRRVPL	7	4	8	4	721	78.3	5.39	239.3	181.8	1.316281628
A8K6C9	cDNA FLJ7	MGIPMGKSMLV	14	2	22	2	180	20.2	9.32	1228.8	934.1	1.315490847
A0A024RD	Phospholi	MVPPKLHVLFCL	8	3	8	3	441	50	7.56	565	431.5	1.309385863
P04196	Histidine-r	MKALIAALLLITL(	51	21	685	1	525	59.5	7.5	866.1	661.8	1.308703536
P33908	Mannosyl-	MPVGGLLPLFSS	7	4	11	4	653	72.9	6.47	388.2	296.9	1.307510946
B7Z8B6	cDNA FLJ5	MNKNVVFVIDIS(	27	10	354	3	623	69.5	6.16	4851.3	3715.2	1.305797804
A0A161I20	Lactoferrin	MKLVFLVLLFLGA	25	14	36	14	711	78.3	8.18	1147.8	879.4	1.305208096
V9HWB4	Epididymis	MKLSLVAAMLLL	12	7	17	7	654	72.3	5.16	779.4	597.3	1.304871924
Q12841	Follistatin-	MWKRWLALALA	3	1	1	1	308	35	5.52	34.7	26.6	1.304511278
O00533	Neural cell	MEPLLLGRGLIVY	4	5	13	5	1208	135	5.76	401.8	308.4	1.302853437
P08779	Keratin, ty	MTTCSRQFTSSS	16	9	45	3	473	51.2	5.05	1891.4	1452.8	1.30189978
P02741	C-reactive	MEKLLCFLVLTSL	24	6	38	6	224	25	5.63	830.7	1082.9	0.767106843
V9GYM3	Apolipopro	MCEQHPKRPGL(	32	6	446	6	133	14.9	8.27	44115.6	58257.6	0.757250556
B7Z3G0	cDNA FLJ5	MSARRTSHGEPK	2	1	16	1	833	94.1	6.87	219.8	295.9	0.74281852
Q8IXL6	Extracellul	MKMMLVRRFRV	3	1	1	1	584	66.2	7.74	88.7	121.2	0.731848185
Q99727	Metallopro	MPGSPRPAPSW	6	1	16	1	224	25.5	7.8	93.4	135	0.691851852

Uniprot Accession Number	protein	Protein amino acid sequence	Protein amino acid sequence matching degree(%)	The number of peptides identified	The total number of times a polypeptide was identified	The number of characteristic peptides identified	Amino acid length	KDa	isoelectric point	relative abundance of normalized protein - sample 114	relative abundance of normalized protein - control sample 121	ratio(114/121)
E9PFZ2	Ceruloplas	MKILILGIFL	53	46	2570	1	946	108.8	5.77	1344.1	232	5.793534
Q96AN5	Transmem	MTVELWLF	3	1	4	1	459	51.7	9.67	268.8	90	2.986667
Q05639	Elongation	MGKEKTHI	2	1	2	1	463	50.4	9.03	111.4	40.3	2.764268
B2R4R0	Histone H4	MSGRGKG	17	2	10	2	103	11.4	11.36	676.1	266.4	2.537913
Q9HCI6	E3 SUMO-p	MGDPGSEI	1	1	6	1	787	89.6	7.74	614.6	247.6	2.482229
V9HVY1	Epididymis	MKRMVSW	2	1	1	1	491	55.9	8.27	127.8	51.6	2.476744
A0A0R4J2F	Spermatog	MPRAQLLE	1	1	2	1	1361	150	8.81	53.8	22.3	2.412556
G9K388	YWHAE/FA	RADPAGAA	5	2	5	2	384	41.2	4.97	307.1	139.1	2.207764
D6W5K2	Thymosin,	GVSSRFPEF	4	1	1	1	169	17.9	10.48	183.4	88	2.084091
B2R773	cDNA, FLJ9	MLLLGAVL	12	2	6	2	244	26.4	5.74	1129.4	546.2	2.067741
A8K486	Peptidyl-pr	MVNPTVFF	5	1	1	1	165	18	6.9	19	9.5	2
P02776	Platelet fac	MSSAAGFC	36	4	140	2	101	10.8	8.62	14120.9	7708.1	1.831956
E2RVJ0	Anion exch	MEELQDDY	3	2	4	2	912	101.9	5.22	41.6	23.2	1.793103
B2RDG0	Proteasom	MSRRYDSR	3	1	2	1	261	29.4	7.72	116.1	67.9	1.709867
V9HW31	ATP syntha	MLGFVGRV	4	2	4	2	529	56.5	5.4	140.3	83.6	1.67823
D3DPK5	SH3 domai	KQLKPSKAI	4	1	1	1	257	26.8	8.38	106.2	63.5	1.672441
Q59EA3	Cadherin 5	TDRQSNGT	7	5	13	5	807	89.9	5.48	497.9	314	1.585669
Q9BWP8	Collectin-1	MRGNLALV	6	2	2	2	271	28.6	5.41	28.1	18.3	1.535519
P04196	Histidine-ri	MKALIAALI	51	21	685	1	525	59.5	7.5	992.4	661.8	1.499547
H6VRG1	Keratin 1 C	MSRQFSSR	26	15	79	13	645	66.1	8.12	3228.1	2159.3	1.494975
A0A024R4	Fibronectir	MLRGPGP(	22	43	227	43	2355	259	5.73	12920.5	8653	1.493182
Q96RZ2	N-acetylglu	MAAGLARI	3	1	1	1	331	36.8	6.79	65.6	45	1.457778
P05164	Myelopero	MGVPFFSS	7	5	7	5	745	83.8	8.97	219.4	150.6	1.456839
H3BQD0	Cerebellin-	MLGVLELLI	11	1	2	1	140	14.7	5.48	18.9	13	1.453846
J3KMX3	Alpha-feto	MKWVESIF	1	1	3	1	622	70.4	5.55	88.4	61.9	1.42811

P07737	Profilin-1 C	MAGWNAY	27	3	12	3	140	15	8.27	353.3	247.6	1.426898
C0JYY2	Apolipoprc	MDPPRPAL	48	227	8770	178	4563	515.2	7.05	722432.2	510456.9	1.415266
P37802	Transgelin-	MANRGPA	30	5	14	5	199	22.4	8.25	625.6	445.5	1.404265
V9HW26	ATP syntha	MLSVRVAA	1	1	2	1	553	59.7	9.13	164.9	117.8	1.39983
P67936	Tropomyos	MAGLNSLE	8	2	4	2	248	28.5	4.69	453.2	328.1	1.381286
P98160	Basement	MGWRAAC	1	2	3	2	4391	468.5	6.51	126.8	91.9	1.379761
Q66K66	Transmem	MPGTVATL	3	1	5	1	360	39.4	9.92	714.8	522.4	1.3683
P35908	Keratin, ty	MSCQISCKS	19	10	39	5	639	65.4	8	955	701.5	1.361368
P10909	Clusterin O	MMKTLLLF	39	18	653	18	449	52.5	6.27	45302.4	33598.7	1.348338
Q12841	Follistatin-	MWKRWLA	3	1	1	1	308	35	5.52	35.6	26.6	1.338346
X6R8F3	Neutrophil	MPLGLLWL	14	2	6	2	200	22.8	8.5	353.7	264.9	1.335221
E7EQ64	Trypsin-1 C	MNPLLILTF	11	2	20	2	261	28.1	7.25	434	329.5	1.317147
A0A024R0	Apolipoprc	MRLFLSLPV	40	7	232	7	83	9.3	8.47	26544.7	20202.7	1.313918
A0A161I20	Lactoferrin	MKLVFLVLI	25	14	36	14	711	78.3	8.18	1147.8	879.4	1.305208
P00915	Carbonic a	MASPDWG	36	7	55	7	261	28.9	7.12	2609.2	2002.4	1.303036
P00918	Carbonic a	MSHHWGY	12	3	5	3	260	29.2	7.4	160	122.8	1.302932
B3KSS6	Phosphodi	MESPTKEIE	1	1	3	1	709	80.7	8.82	291.1	385.6	0.754927
Q8IXL6	Extracellul	MKMMLVF	3	1	1	1	584	66.2	7.74	82.9	121.2	0.683993
A0A075B6	Dynein hea	MEKDAED	0	1	6	1	4707	538.3	6.13	89.2	130.9	0.681436
B7Z3G0	cDNA FLJ5	MSARRTSH	2	1	16	1	833	94.1	6.87	169.4	295.9	0.572491

Uniprot Accession Number	protein	Protein amino acid sequence	Protein amino acid sequence matching degree(%)	The number of peptides identified	The total number of times a polypeptide was identified	The number of characteristic peptides identified	Amino acid length	KDa	isoelectric point	relative abundance of normalized protein - sample 115	relative abundance of normalized protein - control sample 121	ratio(115/1
Q96AN5	Transmem	MTVELWLF	3	1	4	1	459	51.7	9.67	443.2	90	4.924444
V9HVY1	Epididymis	MKRMVSW	2	1	1	1	491	55.9	8.27	209.8	51.6	4.065891
B2R4R0	Histone H4	MSGRGKG	17	2	10	2	103	11.4	11.36	749.1	266.4	2.811937
G9K388	YWHAE/FA	RADPAGAA	5	2	5	2	384	41.2	4.97	367.6	139.1	2.642703
A0A0R4J2F	Spermatog	MPRAQLLE	1	1	2	1	1361	150	8.81	56.5	22.3	2.533632
J3KMX3	Alpha-feto	MKWVESIF	1	1	3	1	622	70.4	5.55	153.4	61.9	2.478191
B2RDG0	Proteasom	MSRRYDSR	3	1	2	1	261	29.4	7.72	156.9	67.9	2.310751
E2RVJ0	Anion exch	MEELQDDY	3	2	4	2	912	101.9	5.22	51.8	23.2	2.232759
Q9BWP8	Collectin-1	MRGNLALV	6	2	2	2	271	28.6	5.41	40.2	18.3	2.196721
Q05639	Elongation	MGKEKTHI	2	1	2	1	463	50.4	9.03	84.2	40.3	2.08933
A0A140GX	Platelet me	MGSGPRGA	5	1	1	1	206	21.7	8.91	81.4	40.9	1.99022
A0A024R4	Fibronectir	MLRGPGPG	22	43	227	43	2355	259	5.73	17208	8653	1.988674
D3DPK5	SH3 domai	KQLKPSKAI	4	1	1	1	257	26.8	8.38	123.2	63.5	1.940157
A2J1N5	Rheumatoi	VESGGGVV	23	2	5	1	94	10.4	9.13	218.9	112.9	1.938884
Q59EA3	Cadherin 5	TDRQSNGT	7	5	13	5	807	89.9	5.48	596.1	314	1.898408
P06733	Alpha-enol	MSILKIHAR	3	1	2	1	434	47.1	7.39	92.1	49.7	1.853119
V9HW31	ATP syntha	MLGFVGRV	4	2	4	2	529	56.5	5.4	154.3	83.6	1.845694
P02776	Platelet fac	MSSAAGFC	36	4	140	2	101	10.8	8.62	14224.7	7708.1	1.845422
P37802	Transgelin-	MANRGPA	30	5	14	5	199	22.4	8.25	751.4	445.5	1.686644
Q99969	Retinoic ac	MRRLLIPLA	7	1	2	1	163	18.6	9.09	68.1	40.7	1.673219
D6W5K2	Thymosin,	GVSSRFPEF	4	1	1	1	169	17.9	10.48	144.4	88	1.640909
Q9H804	cDNA FLJ14	MPGPRVW	21	7	38	1	438	48	7.46	254.6	156.4	1.627877
P67936	Tropomyos	MAGLNSLE	8	2	4	2	248	28.5	4.69	532.2	328.1	1.622066
A0A125U0	GCT-A2 hea	EVQLVESG	15	1	1	1	126	13.8	5.45	30.3	18.9	1.603175
Q66K66	Transmem	MPGTVATL	3	1	5	1	360	39.4	9.92	829.3	522.4	1.587481

A0A1C9J6F	B cell recep	EVQLVQSG	14	1	2	1	132	14.6	8.02	96.2	61.6	1.561688
Q9NQ79	Cartilage a	MAPSADP(	7	5	13	5	661	71.4	5.12	1227.9	792.2	1.549987
J3KPS3	Fructose-b	MPYQYPAL	8	2	2	2	368	39.8	8.09	206.9	133.9	1.545183
P35443	Thrombos	MLAPRGA/	3	2	6	1	961	105.8	4.68	54.5	35.7	1.526611
Q6UY14	ADAMTS-li	MENWTGR	2	1	1	1	1074	116.5	8.34	65.4	42.9	1.524476
A8K430	Fructose-b	MAHRFPAL	21	5	9	5	364	39.4	8.21	169.1	111.1	1.522052
G3XAI2	Laminin su	MDCPLSAP	1	1	1	1	1810	200.3	4.96	150.7	99.6	1.513052
B7ZMD7	Alpha-amy	MKLFWLLF	3	1	1	1	511	57.7	6.93	53.3	35.3	1.509915
O00533	Neural cell	MEPLLLGR	4	5	13	5	1208	135	5.76	460.4	308.4	1.492866
A8K3K1	cDNA FLJ78	MCDDEETT	31	9	48	2	377	42	5.39	92.7	62.2	1.490354
P04040	Catalase O	MADSRDP/	7	3	5	3	527	59.7	7.39	271	182.4	1.485746
Q9UIU0	Dihydropy	MAAGCLLA	2	2	4	2	1110	125.2	5.36	95.2	64.3	1.48056
P02768	Serum albu	MKWVTFIS	62	47	4176	47	609	69.3	6.28	553515.2	374998.6	1.476046
E9PFZ2	Ceruloplas	MKILILGIFL	53	46	2570	1	946	108.8	5.77	340.1	232	1.465948
Q59ED3	Intercellula	LIVTCSLQQ	4	1	2	1	344	38.4	7.78	99.7	68.1	1.464023
E5RG27	Casein kina	MASSSGSK	17	1	1	1	78	8.5	9.7	168.1	115.8	1.451641
P02533	Keratin, ty	MTTCSRQF	16	8	40	2	472	51.5	5.16	80	55.3	1.446655
D3YTG3	Target of N	MRGGKCN	1	2	4	2	1777	195.2	9.73	221.3	153.2	1.444517
P04066	Tissue alph	MRAPGMR	5	2	4	2	466	53.7	6.84	179.1	124.2	1.442029
P10909	Clusterin O	MMKTLLLF	39	18	653	18	449	52.5	6.27	48175.9	33598.7	1.433862
V9HW26	ATP syntha	MLSVRVAA	1	1	2	1	553	59.7	9.13	168.9	117.8	1.433786
X6R8F3	Neutrophil	MPLGLLWL	14	2	6	2	200	22.8	8.5	377.1	264.9	1.423556
P07996	Thrombos	MGLAWGL	37	39	390	1	1170	129.3	4.94	559	392.8	1.423116
P05164	Myelopero	MGVPFFSS	7	5	7	5	745	83.8	8.97	213.8	150.6	1.419655
Q86U17	Serpin A11	MGPAWLV	2	1	1	1	422	47	7.68	76.4	54	1.414815
P07737	Profilin-1 C	MAGWNAY	27	3	12	3	140	15	8.27	350.2	247.6	1.414378
Q9HCI6	E3 SUMO-p	MGDPGSEI	1	1	6	1	787	89.6	7.74	350.2	247.6	1.414378
P33908	Mannosyl-	MPVGGLLF	7	4	11	4	653	72.9	6.47	419.1	296.9	1.411586
Q8NGE6	Olfactory r	MGDKGTG	6	1	16	1	254	28.1	7.91	1695.4	1201.7	1.410835
A8KAJ3	cDNA FLJ7	MLKALFLTI	24	10	39	10	493	54.6	5.14	1889.4	1343.2	1.406641
B2R9V7	Superoxide	MLALLCSCI	12	2	5	2	240	25.9	6.79	144.8	103	1.405825
A0A068LR\	Ig heavy ch	QVTLRESGI	6	1	4	1	127	14	8.15	147	104.6	1.405354
Q0ZCH6	Immunglob	LVQLVESG(	7	1	1	1	131	14.3	6.55	100	71.2	1.404494

A0N5G5	Rheumatoi	EIVLTQSPA	23	2	9	2	118	12.8	8.97	132.6	94.5	1.403175
A8K486	Peptidyl-pr	MVNPTVFF	5	1	1	1	165	18	6.9	13.1	9.5	1.378947
Q16853	Membrane	MNQKTILV	9	5	9	5	763	84.6	6.52	137.7	99.9	1.378378
A0A161I20	Lactoferrin	MKLVFLVLI	25	14	36	14	711	78.3	8.18	1211.5	879.4	1.377644
P98160	Basement	MGWRAAG	1	2	3	2	4391	468.5	6.51	126.6	91.9	1.377584
B2R773	cDNA, FLJ9	MLLLGAVL	12	2	6	2	244	26.4	5.74	751.6	546.2	1.376053
Q9H8L6	Multimerir	MILSLLFSL(	2	2	3	2	949	104.3	5.86	220	160.8	1.368159
Q76LX8	A disintegr	MHQRHPR	4	5	9	5	1427	153.5	7.17	425.6	311.2	1.367609
Q8NF17	FLJ00385 p	LWLWGRE'	23	10	100	2	509	56.1	7.59	331.5	242.4	1.367574
O94818	Nucleolar p	MESERDM'	3	1	1	1	638	71.3	5.62	83	60.9	1.36289
P23470	Receptor-t	MRRLLEPC	4	4	5	4	1445	161.9	6.42	201.5	147.9	1.362407
P21333	Filamin-A (	MSSSHSRA	2	4	8	4	2647	280.6	6.06	168.1	123.6	1.360032
Q13740	CD166 anti	MESKGASS	2	1	1	1	583	65.1	6.25	54.1	39.8	1.359296
Q8WZ75	Roundabou	MGSGGDSI	2	1	3	1	1007	107.4	6.64	207.2	152.6	1.357798
P18065	Insulin-like	MLPRVGCF	22	5	11	5	325	34.8	7.5	408.5	301.5	1.354892
Q8NBP7	Proprotein	MGTVSSRR	5	3	4	3	692	74.2	6.61	186.7	138.5	1.348014
P03950	Angiogenir	MVMGLGV	18	2	3	2	147	16.5	9.64	489.1	363.2	1.346641
P35908	Keratin, ty	MSCQISCKS	19	10	39	5	639	65.4	8	943.1	701.5	1.344405
A0A024R1(	Contactin 1	MKMWLLV	1	1	2	1	1018	113.2	5.9	56.2	41.9	1.341289
Q9Y5Z9	UbiA preny	MAASQVL(	3	1	1	1	338	36.8	8.15	109.1	81.4	1.340295
Q96S96	Phosphatic	MGWTMRI	4	1	2	1	227	25.7	6.54	43.1	32.3	1.334365
B4DQY7	cDNA FLJ5	MSWFNAS	1	1	1	1	992	111.7	4.87	20.8	15.6	1.333333
Q86VB7	Scavenger	MSKLRMVI	5	4	7	4	1156	125.4	5.95	222.9	167.3	1.332337
A0A024QZ	Proteoglyc	MMQKLLK	8	1	2	1	158	17.6	4.86	176.4	132.4	1.332326
J3KNB4	Cathelicidi	MGTMKTQ	5	1	1	1	173	19.6	9.41	421	316.2	1.331436
Q96RZ2	N-acetylglu	MAAGLARI	3	1	1	1	331	36.8	6.79	59.8	45	1.328889
P07359	Platelet gly	MPLLLLLLL	12	6	55	6	652	71.5	6.29	2770	2084.5	1.328856
Q07954	Prolow-de	MLTPPLLLL	1	2	4	2	4544	504.3	5.39	29.5	22.3	1.32287
P40197	Platelet gly	MLRGTLLC	18	8	31	8	560	60.9	9.63	1166.6	882	1.322676
A0A087X1I	Calcium-de	MLDPSSSEI	1	1	49	1	1301	148.2	6.05	391.4	298	1.313423
A6NC48	ADP-ribosy	MAAQGCA	7	2	5	2	333	37.5	7.97	310.2	236.2	1.313294
B2R6V9	cDNA, FLJ9	MSETSRTA	2	1	1	1	732	83.2	6	69.2	52.7	1.313093
P35527	Keratin, ty	MSCRQFSS	13	8	64	7	623	62	5.24	4681.3	3567.9	1.31206

Q9UL89	Myosin-rea	VQSGAEVK	10	1	2	1	116	12.6	9.1	98.2	74.9	1.311081
A0A024RD	SPARC-like	MKTGLFFL	2	1	3	1	664	75.2	4.81	88.3	67.4	1.310089
R4GMU1	GDH/6PGL	MLAEPFNV	1	1	2	1	802	90.1	7.23	62.3	47.6	1.308824
Q6EMK4	Vasorin OS	MCSRVPLL	6	3	13	3	673	71.7	7.39	474.9	363.3	1.307184
D6RF20	Vitamin D-	MKRVLVLL	76	10	581	1	145	16.1	5.83	31.5	24.1	1.307054
A8K6C1	cDNA FLJ7(	MLAATVLT	5	2	5	2	493	54.7	6.09	887	679.2	1.305948
P11279	Lysosome-	MAAPGSAI	4	2	3	2	417	44.9	8.75	311.7	238.8	1.305276
Q4TZM4	Hemoglobi	TPEEKSAVT	89	9	235	2	101	11	6.52	979.5	1332.1	0.735305
V9HWI6	Epididymis	MKRVLVLL	66	32	2121	2	474	52.9	5.54	1355.4	1938.8	0.699092
A0A1K0GX	Globin C1 (	MSAARPGV	51	7	179	7	177	19.2	9.77	9465.6	14723.1	0.642908
Q7Z7Q0	APOB prot	MDPPRPAL	58	50	3404	1	825	92.3	7.42	176	369.3	0.476577

Uniprot Accession Number	protein	Protein amino acid sequence	Protein amino acid sequence matching degree(%)	The number of peptides identified	The total number of times a polypeptide was identified	The number of characteristic peptides identified	Amino acid length	KDa	isoelectric point	relative abundance of normalized protein - sample 116	relative abundance of normalized protein - control sample 121	ratio(116/1
A0A0R4J2F	Spermatog	MPRAQLLE	1	1	2	1	1361	150	8.81	70.6	22.3	3.165919
B2R4R0	Histone H4	MSGRGKG	17	2	10	2	103	11.4	11.36	753.8	266.4	2.82958
G9K388	YWHAE/FA	RADPAGAA	5	2	5	2	384	41.2	4.97	384.3	139.1	2.762761
B2RDG0	Proteasom	MSRRYDSR	3	1	2	1	261	29.4	7.72	166.8	67.9	2.456554
Q8TCZ8	Apolipoprc	RLGADMEI	54	3	161	1	57	6.7	9.98	890	380.9	2.336571
Q9BWP8	Collectin-1	MRGNLAL\	6	2	2	2	271	28.6	5.41	40.5	18.3	2.213115
Q05639	Elongation	MGKEKTHI	2	1	2	1	463	50.4	9.03	85.3	40.3	2.116625
Q96AN5	Transmem	MTVELWLF	3	1	4	1	459	51.7	9.67	179.8	90	1.997778
D6W5K2	Thymosin,	GVSSRFPEF	4	1	1	1	169	17.9	10.48	169.2	88	1.922727
J3KMX3	Alpha-feto	MKWVESIF	1	1	3	1	622	70.4	5.55	117.6	61.9	1.899838
V9HW31	ATP syntha	MLGFVGR\	4	2	4	2	529	56.5	5.4	152	83.6	1.818182
Q9HCI6	E3 SUMO-p	MGDPGSEI	1	1	6	1	787	89.6	7.74	420.3	247.6	1.697496
A0A024RD	SPARC-like	MKTGLFFL	2	1	3	1	664	75.2	4.81	113.9	67.4	1.689911
V9HVY1	Epididymis	MKRMVSW	2	1	1	1	491	55.9	8.27	85.8	51.6	1.662791
P35908	Keratin, typ	MSCQISCKS	19	10	39	5	639	65.4	8	1154.5	701.5	1.645759
Q8NGE6	Olfactory r	MGDKGTG	6	1	16	1	254	28.1	7.91	1970.5	1201.7	1.63976
Q9H628	Ras-relatec	MSNFLHLK	4	1	3	1	205	23.8	9.51	317.1	196	1.617857
Q59EA3	Cadherin 5	TDRQSNGT	7	5	13	5	807	89.9	5.48	503.1	314	1.602229
A8K486	Peptidyl-pr	MVNPTVFF	5	1	1	1	165	18	6.9	15.1	9.5	1.589474
P67936	Tropomyos	MAGLNSLE	8	2	4	2	248	28.5	4.69	517.2	328.1	1.576349
Q66K66	Transmem	MPGTVATL	3	1	5	1	360	39.4	9.92	821.1	522.4	1.571784
E2RVJ0	Anion exch	MEELQDDY	3	2	4	2	912	101.9	5.22	36.3	23.2	1.564655
A0A068LR\	Ig heavy ch	QVTLRESGI	6	1	4	1	127	14	8.15	163.1	104.6	1.559273
Q9ULV0	Unconvent	MSVGELYS	0	2	8	2	1848	213.5	7.2	82.8	53.7	1.541899
J3KPS3	Fructose-b	MPYQYPAL	8	2	2	2	368	39.8	8.09	205.3	133.9	1.533234

A8K430	Fructose-b	MAHRFPAL	21	5	9	5	364	39.4	8.21	169.2	111.1	1.522952
A8K9X5	cDNA FLJ7(	MASNMDR	2	1	10	1	650	73.9	4.88	1951.7	1282.7	1.521556
P37802	Transgelin-	MANRGPA	30	5	14	5	199	22.4	8.25	676.2	445.5	1.517845
Q59ED3	Intercellula	LIVTCSLQQ	4	1	2	1	344	38.4	7.78	102.8	68.1	1.509545
P00738	Haptoglobi	MSALGAVI.	57	27	2184	12	406	45.2	6.58	279577.3	185933.7	1.50364
A0A024R4(	Fibronectir	MLRGPGP(	22	43	227	43	2355	259	5.73	13008.3	8653	1.503328
E5RG27	Casein kina	MASSSGSK	17	1	1	1	78	8.5	9.7	173.4	115.8	1.497409
A0A024QZ	Serine/thre	MELLRTITY	2	1	3	1	685	78.2	8.24	538.4	359.7	1.496803
P02741	C-reactive	MEKLLCFL\	24	6	38	6	224	25	5.63	1604.3	1082.9	1.481485
Q16853	Membrane	MNQKTILV	9	5	9	5	763	84.6	6.52	148	99.9	1.481481
H6VRG1	Keratin 1 C	MSRQFSSR	26	15	79	13	645	66.1	8.12	3184.2	2159.3	1.474645
A8K3K1	cDNA FLJ7ȩ	MCDDEETT	31	9	48	2	377	42	5.39	91.6	62.2	1.472669
Q8WZ75	Roundabou	MGSGGDSI	2	1	3	1	1007	107.4	6.64	220.8	152.6	1.44692
V9HW26	ATP syntha	MLSVRVAA	1	1	2	1	553	59.7	9.13	169.9	117.8	1.442275
P10909	Clusterin O	MMKTLLLF	39	18	653	18	449	52.5	6.27	48440.7	33598.7	1.441743
P98160	Basement	MGWRAA(	1	2	3	2	4391	468.5	6.51	132.4	91.9	1.440696
Q8WWZ8	Oncoprote	MPPFLLLT(	2	1	1	1	545	60	5.58	76.5	53.5	1.429907
P03950	Angiogenir	MVMGLGV	18	2	3	2	147	16.5	9.64	518.6	363.2	1.427863
Q9H804	cDNA FLJ1	MPGPRVW	21	7	38	1	438	48	7.46	222.8	156.4	1.424552
B7ZMD7	Alpha-amy	MKLFWLLF	3	1	1	1	511	57.7	6.93	50.1	35.3	1.419263
P33908	Mannosyl-	MPVGGLLF	7	4	11	4	653	72.9	6.47	421.2	296.9	1.418659
A0A1C9J6F	B cell recep	EVQLVQSG	14	1	2	1	132	14.6	8.02	87.2	61.6	1.415584
P02533	Keratin, typ	MTTCSRQF	16	8	40	2	472	51.5	5.16	78.1	55.3	1.412297
Q86U17	Serpin A11	MGPAWLV	2	1	1	1	422	47	7.68	76.2	54	1.411111
A0A125U0	GCT-A2 he	EVQLVESG	15	1	1	1	126	13.8	5.45	26.6	18.9	1.407407
P05164	Myelopero	MGVPFFSS	7	5	7	5	745	83.8	8.97	211.2	150.6	1.40239
Q99969	Retinoic ac	MRRLLIPLA	7	1	2	1	163	18.6	9.09	56.8	40.7	1.395577
Q0ZCH6	Immunglob	LVQLVESG(	7	1	1	1	131	14.3	6.55	97.9	71.2	1.375
P21333	Filamin-A (	MSSSHSRA	2	4	8	4	2647	280.6	6.06	169.7	123.6	1.372977
Q9Y5Z9	UbiA preny	MAASQVL(	3	1	1	1	338	36.8	8.15	111.7	81.4	1.372236
P07333	Macrophag	MGPGVLLL	3	2	3	2	972	107.9	6.37	44.6	32.6	1.368098
B2R773	cDNA, FLJ9	MLLLGAVL	12	2	6	2	244	26.4	5.74	746	546.2	1.3658
X6R8F3	Neutrophil	MPLGLLWI	14	2	6	2	200	22.8	8.5	359.3	264.9	1.356361

P23470	Receptor-t	MRRLLEPC	4	4	5	4	1445	161.9	6.42	200.5	147.9	1.355646
Q07954	Prolow-de	MLTPPLLLL	1	2	4	2	4544	504.3	5.39	30	22.3	1.345291
Q6YHK3	CD109 anti	MQGPPLLT	1	1	1	1	1445	161.6	5.85	219.3	163.1	1.344574
G3XAI2	Laminin su	MDCPLSAP	1	1	1	1	1810	200.3	4.96	133.7	99.6	1.342369
P04066	Tissue alph	MRAPGMR	5	2	4	2	466	53.7	6.84	166.6	124.2	1.341385
P06733	Alpha-enol	MSILKIHAR	3	1	2	1	434	47.1	7.39	66.6	49.7	1.34004
Q4LE64	NUMA1 va	LSGITKMTL	1	1	45	1	2121	238.7	5.81	1075	802.5	1.339564
P07737	Profilin-1 C	MAGWNAY	27	3	12	3	140	15	8.27	331.5	247.6	1.338853
P18065	Insulin-like	MLPRVGCP	22	5	11	5	325	34.8	7.5	403.6	301.5	1.33864
A6XNE2	Compleme	MHSWERL	39	7	65	7	260	27.8	7.25	4144.5	3098.3	1.337669
B7Z9B1	cDNA FLJ5	MKTPPGAS	2	1	4	1	760	83.4	5.12	245.4	183.5	1.33733
O00151	PDZ and LI	MTTQQIDL	3	1	1	1	329	36	7.02	104.3	78.2	1.33376
P24592	Insulin-like	MTPHRLLP	10	2	5	2	240	25.3	7.81	160	120.4	1.328904
A8KAJ3	cDNA FLJ7	MLKALFLTI	24	10	39	10	493	54.6	5.14	1779.4	1343.2	1.324747
A0N5G5	Rheumatoi	EIVLTQSPA	23	2	9	2	118	12.8	8.97	124.5	94.5	1.31746
Q6UY14	ADAMTS-li	MENWTGR	2	1	1	1	1074	116.5	8.34	56.2	42.9	1.310023
P08709	Coagulatio	MVSQALRL	14	4	14	4	466	51.6	7.23	429.3	328	1.308841
A8K6C1	cDNA FLJ7	MLAATVLT	5	2	5	2	493	54.7	6.09	885.6	679.2	1.303887
A0A161I20	Lactoferrin	MKLVFLVLI	25	14	36	14	711	78.3	8.18	1145.7	879.4	1.30282
A2J1N5	Rheumatoi	VESGGGVV	23	2	5	1	94	10.4	9.13	147	112.9	1.302037
A2NYQ9	Anti-folate	QVQLVESG	32	3	16	1	120	13.3	8.44	41.3	53.9	0.766234
A0A1B0GU	Ig mu chair	GSASAPTLF	38	16	591	3	474	51.9	6.15	31512.1	41758.8	0.754622
Q92833	Protein Jur	MSKERPKR	1	1	7	1	1246	138.6	9.38	447.8	606.3	0.738578
Q8TCD0	Uncharacte	MRLPAQLL	41	6	154	1	239	26.2	8.06	341.9	466.9	0.732277
V9GYM3	Apoliporc	MCEQHPKI	32	6	446	6	133	14.9	8.27	40529.6	58257.6	0.695696
Q96JG6	Syndetin O	MQKIKSLM	1	1	4	1	964	111.1	6.2	172.5	264.9	0.651189
Q8IXL6	Extracellul	MKMMLVF	3	1	1	1	584	66.2	7.74	78.9	121.2	0.65099
E9KL23	Epididymis	MPSSVSW	61	31	1738	1	418	46.7	5.59	4010.7	6306.2	0.635993

Uniprot Accession Number	protein	Protein amino acid sequence	Protein amino acid sequence matching degree(%)	The number of peptides identified	The total number of times a polypeptide was identified	The number of characteristic peptides identified	Amino acid length	KDa	isoelectric point	relative abundance of normalized protein - sample 117	relative abundance of normalized protein - control sample 121	ratio(117/1
Q05639	Elongation	MGKEKTHI	2	1	2	1	463	50.4	9.03	136.9	40.3	3.397022
A0A0R4J2F	Spermatog	MPRAQLLE	1	1	2	1	1361	150	8.81	68.9	22.3	3.089686
B2R4R0	Histone H4	MSGRGKG	17	2	10	2	103	11.4	11.36	704.1	266.4	2.643018
D6W5K2	Thymosin,	GVSSRFPEF	4	1	1	1	169	17.9	10.48	204.7	88	2.326136
G9K388	YWHAE/FA	RADPAGAA	5	2	5	2	384	41.2	4.97	322.6	139.1	2.319195
Q9BWP8	Collectin-1	MRGNLALV	6	2	2	2	271	28.6	5.41	40.3	18.3	2.202186
A0A024R4	Fibronectir	MLRGPGPC	22	43	227	43	2355	259	5.73	18122.1	8653	2.094314
B2RDG0	Proteasom	MSRRYDSR	3	1	2	1	261	29.4	7.72	127.9	67.9	1.883652
P35908	Keratin, ty	MSCQISCKS	19	10	39	5	639	65.4	8	1302.1	701.5	1.856165
J3KMX3	Alpha-feto	MKWVESIF	1	1	3	1	622	70.4	5.55	104.8	61.9	1.693053
V9HW31	ATP syntha	MLGFVGRV	4	2	4	2	529	56.5	5.4	138.6	83.6	1.657895
H6VRG1	Keratin 1 C	MSRQFSSR	26	15	79	13	645	66.1	8.12	3565.9	2159.3	1.651415
P05164	Myelopero	MGVPFFSS	7	5	7	5	745	83.8	8.97	238.4	150.6	1.583001
Q59EA3	Cadherin 5	TDRQSNGT	7	5	13	5	807	89.9	5.48	496.3	314	1.580573
P07996	Thrombos	MGLAWGL	37	39	390	1	1170	129.3	4.94	606.6	392.8	1.544297
Q9HCI6	E3 SUMO-	MGDPGSEI	1	1	6	1	787	89.6	7.74	379.6	247.6	1.533118
Q96AN5	Transmem	MTVELWLF	3	1	4	1	459	51.7	9.67	136.5	90	1.516667
P37802	Transgelin-	MANRGPA	30	5	14	5	199	22.4	8.25	673.6	445.5	1.512009
P98160	Basement	MGWRAAC	1	2	3	2	4391	468.5	6.51	138.4	91.9	1.505985
Q8TCZ8	Apolipoprc	RLGADMED	54	3	161	1	57	6.7	9.98	571.4	380.9	1.500131
A8K9X5	cDNA FLJ7	MASNMDR	2	1	10	1	650	73.9	4.88	1903.3	1282.7	1.483823
Q86U17	Serpin A11	MGPAWLV	2	1	1	1	422	47	7.68	78.9	54	1.461111
V9HWI6	Epididymis	MKRVLVLL	66	32	2121	2	474	52.9	5.54	2818.7	1938.8	1.453837
P07737	Profilin-1 C	MAGWNAY	27	3	12	3	140	15	8.27	356.3	247.6	1.439015
B1AHL2	Fibulin-1 O	MERAAPSR	7	4	8	4	721	78.3	5.39	261.5	181.8	1.438394

E2RVJ0	Anion exch	MEELQDDY	3	2	4	2	912	101.9	5.22	32.8	23.2	1.413793
P06733	Alpha-enol	MSILKIHAR	3	1	2	1	434	47.1	7.39	70	49.7	1.408451
P13645	Keratin, typ	MSVRYSSSI	19	12	89	9	584	58.8	5.21	5879.9	4187.6	1.404122
P10909	Clusterin O	MMKTLLLF	39	18	653	18	449	52.5	6.27	47018.7	33598.7	1.39942
A0A1C9J6F	B cell recep	EVQLVQSG	14	1	2	1	132	14.6	8.02	86.2	61.6	1.399351
P67936	Tropomyos	MAGLNSLE	8	2	4	2	248	28.5	4.69	458.3	328.1	1.39683
Q4LE64	NUMA1 va	LSGITKMTL	1	1	45	1	2121	238.7	5.81	1117.1	802.5	1.392025
V9HW26	ATP syntha	MLSVRVAA	1	1	2	1	553	59.7	9.13	162.5	117.8	1.379457
A8K486	Peptidyl-pr	MVNPTVFF	5	1	1	1	165	18	6.9	13.1	9.5	1.378947
Q9Y5Z9	UbiA preny	MAASQVLC	3	1	1	1	338	36.8	8.15	112	81.4	1.375921
P49746	Thrombosp	METQELRG	3	3	17	2	956	104.1	4.65	854.1	626.2	1.363941
A0A024RC	Peroxisom	MEQPQEA	2	1	5	1	441	49.9	7.59	603.2	446.8	1.350045
E7EQ64	Trypsin-1 C	MNPLLILTF	11	2	20	2	261	28.1	7.25	442.1	329.5	1.34173
A8KAJ3	cDNA FLJ7	MLKALFLTI	24	10	39	10	493	54.6	5.14	1797	1343.2	1.33785
Q6EMK4	Vasorin OS	MCSRVPLL	6	3	13	3	673	71.7	7.39	485.5	363.3	1.336361
X6R8F3	Neutrophil	MPLGLLWL	14	2	6	2	200	22.8	8.5	354	264.9	1.336353
P02533	Keratin, typ	MTTCSRQF	16	8	40	2	472	51.5	5.16	73.8	55.3	1.334539
Q8NBP7	Proprotein	MGTVSSRR	5	3	4	3	692	74.2	6.61	184.5	138.5	1.33213
B2R6V9	cDNA, FLJ9	MSETSRTA	2	1	1	1	732	83.2	6	69.9	52.7	1.326376
P00918	Carbonic a	MSHHWGY	12	3	5	3	260	29.2	7.4	162.7	122.8	1.324919
Q8IY21	Probable A	MERNVLTT	1	1	12	1	1712	197.7	7.59	1047.1	795	1.317107
E5RG27	Casein kina	MASSSGSK	17	1	1	1	78	8.5	9.7	152.4	115.8	1.316062
Q8NGE6	Olfactory r	MGDKGTG	6	1	16	1	254	28.1	7.91	1579.6	1201.7	1.314471
P07359	Platelet gly	MPLLLLLLL	12	6	55	6	652	71.5	6.29	2718.9	2084.5	1.304342
P03950	Angiogenir	MVMGLGV	18	2	3	2	147	16.5	9.64	472.9	363.2	1.302037
A0A068LL6	Ig heavy ch	QVQLVQSG	19	2	16	1	120	13.3	7.93	77.1	59.3	1.300169
B3KSS6	Phosphodi	MESPTKEIE	1	1	3	1	709	80.7	8.82	295.2	385.6	0.76556
A0A1K0GX	Globin C1 (	MSAARPGV	51	7	179	7	177	19.2	9.77	10905.9	14723.1	0.740734
Q4TZM4	Hemoglobi	TPEEKSAVT	89	9	235	2	101	11	6.52	935.1	1332.1	0.701974
A0A1B1CY	Vitamin D I	LAERLKAKL	38	2	104	2	34	3.7	8.56	7147.6	10424.2	0.685674
Q9H804	cDNA FLJ1	MPGPRVW	21	7	38	1	438	48	7.46	105.8	156.4	0.676471

Uniprot Accession Number	protein	Protein amino acid sequence	Protein amino acid sequence matching degree(%)	The number of peptides identified	The total number of times a polypeptide was identified	The number of characteristic peptides identified	Amino acid length	KDa	isoelectric point	relative abundance of normalized protein - sample 118	relative abundance of normalized protein - control sample 121	ratio(118/1
D6W5K2	Thymosin,	GVSSRFPEF	4	1	1	1	169	17.9	10.48	181.3	88	2.060227
E2RVJ0	Anion exch	MEELQDDY	3	2	4	2	912	101.9	5.22	41.9	23.2	1.806034
P98160	Basement	MGWRAAC	1	2	3	2	4391	468.5	6.51	159.1	91.9	1.73123
A0A068B0'	Cis-AB glyc	MAEVLRTL	5	1	1	1	354	40.8	8.87	124.2	74.1	1.676113
B2RDG0	Proteasom	MSRRYDSR	3	1	2	1	261	29.4	7.72	113.5	67.9	1.671576
Q86U17	Serpin A11	MGPAWLW	2	1	1	1	422	47	7.68	89.4	54	1.655556
A0A0R4J2F	Spermatog	MPRAQLLE	1	1	2	1	1361	150	8.81	36.4	22.3	1.632287
A8K9X5	cDNA FLJ7	MASNMDF	2	1	10	1	650	73.9	4.88	2038.2	1282.7	1.588992
P07996	Thrombos	MGLAWGL	37	39	390	1	1170	129.3	4.94	606.1	392.8	1.543024
Q8NF17	FLJ00385 p	LWLWGRE	23	10	100	2	509	56.1	7.59	370.4	242.4	1.528053
Q9H628	Ras-relatec	MSNFLHLK	4	1	3	1	205	23.8	9.51	295.1	196	1.505612
Q12841	Follistatin-	MWKRWLA	3	1	1	1	308	35	5.52	39.1	26.6	1.469925
Q8TCZ8	Apolipopro	RLGADMEI	54	3	161	1	57	6.7	9.98	559.4	380.9	1.468627
A8K486	Peptidyl-pr	MVNPTVFF	5	1	1	1	165	18	6.9	13.8	9.5	1.452632
Q96S96	Phosphatic	MGWTMRI	4	1	2	1	227	25.7	6.54	46.8	32.3	1.448916
P05164	Myelopero	MGVPFFSS	7	5	7	5	745	83.8	8.97	216	150.6	1.434263
P02533	Keratin, ty	MTTCSRQF	16	8	40	2	472	51.5	5.16	79	55.3	1.428571
A0A125U0	GCT-A2 he	EVQLVESG	15	1	1	1	126	13.8	5.45	26.9	18.9	1.42328
Q05639	Elongation	MGKEKTHI	2	1	2	1	463	50.4	9.03	56.2	40.3	1.394541
H3BQD0	Cerebellin-	MLGVLELLI	11	1	2	1	140	14.7	5.48	18.1	13	1.392308
P00738	Haptoglobi	MSALGAVI	57	27	2184	12	406	45.2	6.58	258436.5	185933.7	1.389939
B7ZMD7	Alpha-amy	MKLFWLLF	3	1	1	1	511	57.7	6.93	49	35.3	1.388102
P00918	Carbonic a	MSHHWGY	12	3	5	3	260	29.2	7.4	170	122.8	1.384365
A8K6A6	cDNA FLJ7	MGLPRLVC	11	6	13	6	646	71.6	5.67	878.3	640.8	1.37063
A8K430	Fructose-b	MAHRFPAL	21	5	9	5	364	39.4	8.21	152.2	111.1	1.369937

P07737	Profilin-1 C	MAGWNAY	27	3	12	3	140	15	8.27	337.3	247.6	1.362278
P37802	Transgelin-	MANRGPA	30	5	14	5	199	22.4	8.25	605.5	445.5	1.359147
S6B2A1	IgG L chain	MVLQTQVI	23	3	107	1	184	20.4	5.52	20.7	15.4	1.344156
P04275	von Willeb	MIPARFAG	23	51	243	2	2813	309.1	5.48	13496.9	10196	1.323745
A0A1B0GU	Ig mu chair	GSASAPTLF	38	16	591	3	474	51.9	6.15	55273.6	41758.8	1.32364
P67936	Tropomyos	MAGLNSLE	8	2	4	2	248	28.5	4.69	433.9	328.1	1.322463
D3DPK5	SH3 domai	KQLKPSKAI	4	1	1	1	257	26.8	8.38	83.4	63.5	1.313386
O43866	CD5 antige	MALLFSLIL	33	8	25	8	347	38.1	5.47	1626.1	1243.5	1.30768
A0A060VC	MHC class	SHSMRYFF	5	1	9	1	340	38.5	6.15	1310.8	1005.6	1.3035
Q86TT1	Full-length	MQGTDEH	41	14	575	1	375	41.2	6.79	818	628.3	1.301926
Q8IXL6	Extracellul	MKMMLVF	3	1	1	1	584	66.2	7.74	92.9	121.2	0.766502
A0A140VK	Leukotrien	MPEIVDTC	2	1	1	1	611	69.2	6.18	24	31.4	0.764331
Q6MZX7	Putative ur	MELMCKKI	16	6	73	2	476	52.4	7.77	938.7	1228.3	0.764227
Q8NGE6	Olfactory r	MGDKGTG	6	1	16	1	254	28.1	7.91	898.4	1201.7	0.747608
P02768	Serum albu	MKWVTFIS	62	47	4176	47	609	69.3	6.28	273544.9	374998.6	0.729456
E5RG27	Casein kina	MASSSGSK	17	1	1	1	78	8.5	9.7	80	115.8	0.690846
A0A024QZ	Serine/thre	MELLRTITY	2	1	3	1	685	78.2	8.24	246.8	359.7	0.686127
Q7Z7Q0	APOB prot	MDPPRPAL	58	50	3404	1	825	92.3	7.42	163	369.3	0.441376

Uniprot Accession Number	protein	Protein amino acid sequence	Protein amino acid sequence matching degree(%)	The number of peptides identified	The total number of times a polypeptide was identified	The number of characteristic peptides identified	Amino acid length	KDa	isoelectric point	relative abundance of normalized protein - sample 119	relative abundance of normalized protein - control sample 121	ratio(119/1
P02741	C-reactive	MEKLLCFL\	24	6	38	6	224	25	5.63	4798.1	1082.9	4.430788
A0A125QY	GCT-A9 ligł	DIQMTQSF	32	2	5	2	107	11.6	4.65	270.5	100.7	2.686197
Q05639	Elongation	MGKEKTHI	2	1	2	1	463	50.4	9.03	104.1	40.3	2.583127
G9K388	YWHAE/FA	RADPAGA/	5	2	5	2	384	41.2	4.97	340.3	139.1	2.446441
A0A0R4J2F	Spermatog	MPRAQLLE	1	1	2	1	1361	150	8.81	52.8	22.3	2.367713
P00738	Haptoglobi	MSALGAVI.	57	27	2184	12	406	45.2	6.58	410792.3	185933.7	2.209348
B2R4R0	Histone H4	MSGRGKG(	17	2	10	2	103	11.4	11.36	564.9	266.4	2.120495
P02763	Alpha-1-ac	MALSWVL 	45	9	562	6	201	23.5	5.02	45114	21588.2	2.089753
Q8TCZ8	Apolipoprc	RLGADMEI	54	3	161	1	57	6.7	9.98	698.9	380.9	1.834865
Q9H628	Ras-relatec	MSNFLHLK	4	1	3	1	205	23.8	9.51	349.6	196	1.783673
B2RDG0	Proteasom	MSRRYDSR	3	1	2	1	261	29.4	7.72	120.2	67.9	1.77025
P0DJI8	Serum amy	MKLLTGLV	41	4	22	2	122	13.5	6.79	683.4	389.3	1.755459
P00739	Haptoglobi	MSDLGAVI	53	21	1147	6	348	39	7.09	4403	2635.5	1.670651
P0DJI9	Serum amy	MKLLTGLV	25	4	16	2	122	13.5	9.14	467.6	294.1	1.589935
Q9ULV0	Unconvent	MSVGELYS	0	2	8	2	1848	213.5	7.2	84.6	53.7	1.575419
A0A024R6I	Alpha-1-an	MPSSVSW(	61	31	1672	1	418	46.7	5.59	254797.8	165755.8	1.537188
V9HW31	ATP syntha	MLGFVGR\	4	2	4	2	529	56.5	5.4	122.9	83.6	1.470096
P18428	Lipopolysa	MGALARAI	9	4	26	4	481	53.4	6.7	1446.9	996.7	1.451691
B4DQY7	cDNA FLJ5:	MSWFNAS	1	1	1	1	992	111.7	4.87	22.6	15.6	1.448718
P19652	Alpha-1-ac	MALSWVL	47	10	310	7	201	23.6	5.11	27901.1	19263.5	1.448392
D6W5L6	Pulmonary	MHQAGYP	3	1	2	1	393	43.3	5.74	38.6	27.1	1.424354
Q07954	Prolow-dei	MLTPPLLLL	1	2	4	2	4544	504.3	5.39	31.1	22.3	1.394619
A0A1B0GV	GRAM don	MVQNLQP	3	1	1	1	767	88.6	6.44	46.9	33.8	1.387574
Q9H804	cDNA FLJ1	MPGPRVW	21	7	38	1	438	48	7.46	212.9	156.4	1.361253
A0A068LL6	Ig heavy ch	QVQLVQSC	19	2	16	1	120	13.3	7.93	80.2	59.3	1.352445

D3DPK5	SH3 domai	KQLKPSKAI	4	1	1	1	257	26.8	8.38	85	63.5	1.338583
P02750	Leucine-ric	MSSWSRQ	28	9	294	9	347	38.2	6.95	27178.7	20434.1	1.330066
B3KS79	cDNA FLJ3!	MKIHYSRQ	34	19	1335	19	448	50.6	5.63	132515.7	99858	1.327041
V9HVY1	Epididymis	MKRMVSW	2	1	1	1	491	55.9	8.27	68	51.6	1.317829
Q16853	Membrane	MNQKTILV	9	5	9	5	763	84.6	6.52	76.7	99.9	0.767768
E5RG27	Casein kina	MASSSGSK	17	1	1	1	78	8.5	9.7	88.9	115.8	0.767703
Q4ZG40	Macrophag	MRNKKILKI	3	1	2	1	520	52.6	8.78	131.7	171.9	0.766143
J3KPA1	Cysteine-ri	MKQILHPA	9	3	13	3	276	31	7.61	1279.2	1673.4	0.764432
V9GYM3	Apolipoprc	MCEQHPKI	32	6	446	6	133	14.9	8.27	44102	58257.6	0.757017
A0A0C4DG	Poliovirus r	MARAMAA	2	1	2	1	417	45.3	6.52	253.4	336.2	0.753718
Q13103	Secreted p	MISRMEKN	27	5	11	5	211	24.3	8.32	215.2	286.8	0.750349
J3KMX3	Alpha-feto	MKWVESIF	1	1	3	1	622	70.4	5.55	46.4	61.9	0.749596
P06727	Apolipoprc	MFLKAVVL	70	36	1655	36	396	45.4	5.38	98547	132070.1	0.746172
B2R815	cDNA, FLJ9	MHLIDYLLL	38	15	289	15	427	48.5	7.75	13815.6	18523.7	0.745834
P49908	Selenoprot	MWRSLGL/	14	6	50	6	381	43.2	7.87	2561.1	3441.9	0.744095
P02743	Serum amy	MNKPLLWI	25	6	181	6	223	25.4	6.54	9575.8	12930.8	0.740542
O94818	Nucleolar p	MESERDM	3	1	1	1	638	71.3	5.62	44.9	60.9	0.737274
B3KUE5	Phospholip	MGLSGSDI	9	4	16	4	513	56.6	6.64	662.1	901.5	0.734443
Q66K66	Transmem	MPGTVATL	3	1	5	1	360	39.4	9.92	380.9	522.4	0.729135
P25391	Laminin su	MRGGVLL\	0	1	2	1	3075	336.9	6.35	49.1	67.6	0.726331
A4D2D2	Procollager	MLPAATAS	12	4	7	4	449	47.9	7.43	96.7	134.5	0.718959
Q96JG6	Syndetin O	MQKIKSLM	1	1	4	1	964	111.1	6.2	189.2	264.9	0.714232
Q4LE64	NUMA1 va	LSGITKMTL	1	1	45	1	2121	238.7	5.81	571.4	802.5	0.712025
B2R9V7	Superoxide	MLALLCSCI	12	2	5	2	240	25.9	6.79	73.1	103	0.709709
P23470	Receptor-t	MRRLLEPC	4	4	5	4	1445	161.9	6.42	104.6	147.9	0.707235
Q9Y5Y7	Lymphatic	MARCFSLV	11	4	24	4	322	35.2	8.28	964.9	1364.9	0.706938
B2R6V9	cDNA, FLJ9	MSETSRTA	2	1	1	1	732	83.2	6	37.2	52.7	0.705882
A0A024R1(	Contactin 1	MKMWLLV	1	1	2	1	1018	113.2	5.9	29.5	41.9	0.704057
P35916	Vascular er	MQRGAAL(	2	1	1	1	1363	152.7	6.3	31.2	44.5	0.701124
A0A068LR\	Ig heavy ch	QVTLRESGI	6	1	4	1	127	14	8.15	73.3	104.6	0.700765
A0A140TA:	Compleme	MRLLWGLI	48	76	5311	1	1698	187.5	7.33	7018.6	10069.7	0.697002
E7EQ64	Trypsin-1 (	MNPLLILTF	11	2	20	2	261	28.1	7.25	229.1	329.5	0.695296
A8K9X5	cDNA FLJ7(	MASNMDF	2	1	10	1	650	73.9	4.88	880.6	1282.7	0.686521

A0A024R8(	Glucosidas	MGVRHPP(	1	1	1	1	952	105.3	6	42	61.2	0.686275
Q7Z7Q0	APOB prot	MDPPRPAL	58	50	3404	1	825	92.3	7.42	248.4	369.3	0.672624
Q8NBP7	Proprotein	MGTVSSRR	5	3	4	3	692	74.2	6.61	92.9	138.5	0.670758
B7Z3G0	cDNA FLJ5	MSARRTSH	2	1	16	1	833	94.1	6.87	191.9	295.9	0.64853
Q6PIY7	Poly(A) RN	MFPNSILGI	2	1	1	1	484	56	9.38	43	67.2	0.639881
A0A024QZ	Serine/thre	MELLRTITY	2	1	3	1	685	78.2	8.24	223.6	359.7	0.621629
A0A075B6I	Dynein hea	MEKDAED(	0	1	6	1	4707	538.3	6.13	77	130.9	0.588235
P02768	Serum albu	MKWVTFIS	62	47	4176	47	609	69.3	6.28	216497.6	374998.6	0.577329
Q8IXL6	Extracellul	MKMMLVF	3	1	1	1	584	66.2	7.74	48.6	121.2	0.40099
A0A1B1CY(	Vitamin D I	LAERLKAKL	38	2	104	2	34	3.7	8.56	3808.9	10424.2	0.36539
