# Supplementary material for: LC-MS/MS-Based Quantitative Proteomics Analysis of Different Stages of Non-Small-Cell Lung Cancer
Source: Biomed Res Int. 2021 Feb 26;2021:5561569. doi: 10.1155/2021/5561569 (PMC7937045; doi:10.1155/2021/5561569)
Supplement: Supplementary 2 — Table S2: the results of urine reversed-phase elution test and searching in the SEQUEST database. [file 5561569.f2.pdf]

Uniprot Accession Number	Protein	protein amino acid sequence	protein amino acid sequence matching degree （%）	number of identified polypeptides	total number of identified polypeptides	characteristic polypeptide number identified	ength of amino acid	The molecular weight KDa	isoelectric point	relative abundance of normalized protein - sample 113	relative abundance of normalized protein - sample 114	relative abundance of normalized protein - sample 115	relative abundance of normalized protein - sample 116	relative abundance of normalized protein - sample 117	relative abundance of normalized protein - sample 118	relative abundance of normalized protein - sample 119	relative abundance of normalized protein - sample 121
P02768	Serum albu	MKWVTFIS	73	56	9971	56	609	69.3	6.28	862466.6	593482.1	2220421	846469.3	634302.5	736961.1	842297.5	666397.2
P02760	Protein AM	MRSLGALL	51	16	4825	5	352	39	6.25	294812.3	474165.8	203377.3	349197.6	346271.7	328356.1	411229.3	381627.7
B4E1B2	cDNA FLJ5	MRLAVGAI	62	43	1716	3	678	74.8	7.12	107560.1	85218.8	199640.4	90022.3	73427.3	82722	102491.8	78511.8
A0A0K0K1I	Epididymis	MRLAVGAI	58	42	1721	2	698	77	7.21	4584.8	4234.8	8496.1	4193.7	3507.1	3632.2	4873.3	3358.1
D3DNU8	Kininogen	MKLITILFL(	52	28	3345	2	427	47.8	6.65	294936.6	257956.9	193831.3	290406.7	313453.3	279805.5	228883.9	302648.3
B4E1C2	Kininogen	MKLITILFL(	36	27	3259	1	644	71.9	6.81	229.7	263.2	278	326	265.5	290.3	341.5	167.9
O60494	Cubilin OS=	MMNMSLF	22	61	773	61	3623	398.5	5.35	45037.5	38654.5	34055.8	39708.6	44702.9	45025.7	32701.3	40132.6
P98164	Low-densit	MDRGPAA	19	75	789	75	4655	521.6	5.08	45344.8	35817.7	35721.8	42157.7	46693.4	48624	38847.7	40915.4
A0A024RA	Heparan su	MRWTHSY	17	57	826	31	4346	463.7	6.51	43536.1	49827.7	33964.3	44780.9	46858.6	47703.5	37785.7	46985.3
P01133	Pro-epider	MLLTLIILLF	29	33	1041	33	1207	133.9	5.85	73109.5	66336.1	56680.9	70877.8	76444.3	81110.3	65749.7	75270.8
Q6N093	Putative ur	FFNYAMH\	46	14	2214	7	417	46	7.59	55632.5	48428.3	63601.5	65204.2	59384.5	40148.4	58939.1	47825.3
X6RBG4	Uromoduli	MRHIMTRI	39	23	3450	23	689	75.6	5.87	267652.8	169355	173051.8	249815.6	272871.5	323278.4	237342.9	364524.4
S4R471	Protein AM	HKSKWNIT	51	10	2476	1	193	21.4	7.25	830.2	1013.7	501.8	900.2	865.2	813.1	1077	913.4
Q6MZQ6	Putative ur	MEFGLSWI	48	16	2484	6	475	52	8.06	156851.3	141749.9	138599.9	186246.8	144680.4	140760.2	177689.2	150720
A0A024R4(	Fibronectir	MLRGPGP(	25	46	468	46	2355	259	5.73	23599.9	22486.3	17512.3	26256.4	24031.8	23591	23870.8	23276.7
P04746	Pancreatic	MKFFLLLFT	53	20	829	3	511	57.7	7.05	3567.6	1554.5	3116.9	3014.4	3868.6	3474.2	2469.6	2613.9
P01833	Polymeric i	MLLFVLTCl	37	24	1000	24	764	83.2	5.74	65223.8	51222.8	44831.7	57440.9	84481.8	65065.7	60349.2	75401.1
V9HW34	Epididymis	METPAQLL	49	8	2857	1	235	25.6	6.55	787.8	1077.1	619	873.4	900.6	903.4	875.5	908.7
Q6PIL8	IGK@ prot	METPAQLL	47	8	2890	1	236	25.8	6.55	3026.6	3563	2074.2	2760.8	3139.4	3486.2	3443.1	3364.6
Q6P5S8	IGK@ prot	METPAQLL	47	8	2911	1	236	25.8	6.33	151922.2	222216.3	121290.3	166687	160695.3	198000.3	194547.5	183839.5
Q0KKI6	Immunobl	EIVMTQSP	48	8	2901	1	219	24	8.06	3800.1	4394.4	2855.3	3317	4193.4	4965.8	4898.1	4700.9
Q59E93	Membrane	GSLSRLPTI	33	35	584	15	977	110.5	5.53	34854.5	27418	27084.2	34101	35756.3	28833.9	28075.9	32198.4
B7ZMD7	Alpha-amy	MKLFWLLF	51	20	717	3	511	57.7	6.93	36477.1	19818.9	29799.8	33533.9	37893.7	36147.9	26885.5	26254.5
A0A161I20	Lactoferrin	MKLVFLVL	54	34	466	0	711	78.3	8.18	39936.6	20213.2	13042.9	22989.8	19645.5	23345.6	23066.2	15145.7
A0A024R8(	Glucosidas	MGVRHPP(	25	19	538	7	952	105.3	6	26278.3	20740.9	19305.1	24136.9	25723	25941.7	21714.1	26196.1
P00734	Prothromb	MAHVRGL(	33	17	694	17	622	70	5.9	36023.3	30475.5	25009.6	33369.7	35911.5	37733	35981.5	39265.9
Q96K68	cDNA FLJ1	MELGLRW	38	15	1028	1	494	53.1	6.86	60465.5	47280	46205.5	55632	65877.7	56589.8	57673.3	63862.1
B3VMW0	Lactoferrin	MKLVFLVL	54	34	448	1	711	78.3	8.12								
W8QEY1	Lactoferrin	MKLVFLVL	52	33	446	1	711	78.3	8.18	227.3	174.7	186.3	323.9	207.4	189.2	220.6	203
V9HWA9	Epididymis	MGPTSGPS	35	55	305	55	1663	187	6.4	17774.3	14518.8	19834.7	14532.1	11925	17333.2	16259.6	10679.9
Q5EFE6	Anti-RhD m	MRVPAQLI	40	6	2769	1	234	25.7	8.43	63.8	94.1	46.1	87.4	55.9	65.6	99.8	66
A0A024RD	Secreted p	MRIAVICF(	50	13	1136	2	314	35.4	4.58	58597	41336.7	33620	55794.7	61223.2	73467.6	49830.6	70922.1
Q59EG0	Basement	SVTEGQTLI	15	27	593	1	2331	246.3	7.23	1510.7	1463.9	1070.7	2426.2	2042.3	3369.3	1566.4	1737.4
A0A140TA	Tenascin-X	MMPAQYA	19	46	295	27	4242	458	5.17	15479.7	13776.6	11588.2	14985.1	15236.8	14675.1	12717	15142.2

Q6N092	Putative ur	GEEPSTRSF	34	14	1008	1	519	56.4	6.93	212.3	170.6	187.3	215.6	195.9	161.8	243	292.1
Q8NCL6	cDNA FLJ9(	MQFGLSW	36	14	1007	1	493	53.2	6.52	111.4	103.6	88.7	97	97.6	94.8	97.1	95.9
E9KL23	Epididymis	MPSSVSW(	53	24	660	1	418	46.7	5.59	2619.7	1850.8	4131.1	1940.5	1781.5	1987.8	2198.3	1716
P25311	Zinc-alpha-	MVRMVPV	60	20	1734	20	298	34.2	6.05	103361.5	236874.5	90208.1	112762.6	114953.3	87034.2	132935.6	151729.7
A0A024RD	Secreted p	MRIAVICF(	47	12	1140	1	300	33.8	4.61	7830	6811.9	4818.4	8363.9	8910.8	10063.6	8087.2	9894.7
A0A024R6I	Alpha-1-an	MPSSVSW(	53	24	629	1	418	46.7	5.59	44423.8	33176.1	80233.1	38125.8	36761.6	37927.4	49272.2	30932.8
P35555	Fibrillin-1 (	MRRGRLLE	18	41	372	41	2871	312	4.93	24349.7	20530	19914.2	23308.7	24593.8	22820.3	21604.6	24928.2
P12109	Collagen al	MRAARALL	27	24	633	24	1028	108.5	5.43	33324.9	28063	23704.5	29432.1	35827.7	35265.8	28378.3	31949.7
A8KAJ3	cDNA FLJ7	MLKALFLTI	42	16	411	3	493	54.6	5.14	24208.6	28686.9	17831.4	24959.9	27916.5	24654.5	21521.2	26275.3
A0A0S2Z3Y	Lectin gala	MTPPRLFW	38	20	537	20	585	65.3	5.27	24440.8	21860.8	20279.5	22939.6	26958.2	29734.9	24870.3	27682.3
E7ER45	Maltase-gl	MARKKLKK	21	32	400	32	2753	311.8	5.41	20741	15799.2	15308.5	19737.9	19120.9	20698.3	17321	17395.7
A0A024R8(	Prostaglan	MATHHTLV	41	6	3425	6	190	21	7.8	132959.1	249501.5	92848.1	168444.8	144643.5	167428.8	131771.6	157265.4
P15309	Prostatic a	MRAAPLLL	32	13	827	13	386	44.5	6.24	44629.5	66404	57259.6	45211.6	65647.6	24586.7	22654.6	60390
P02763	Alpha-1-ac	MALSWVLT	40	7	965	5	201	23.5	5.02	35706.4	76375	34617.4	42550.7	30650.9	32510.1	95028.1	48390.7
A8K5A4	cDNA FLJ7(	MKILILGIFL	28	24	284	24	1065	122.1	5.74	16788.8	14281.1	18971.2	15934.6	14697.8	16077.5	20626.8	14909.8
A0A0S2Z3V	EGF contai	MLKALFLTI	46	14	386	1	413	45.7	5.47	164.5	149.8	117.7	158	151.2	239.5	123.5	118.6
P01861	Ig gamma-	ASTKGPSVI	57	12	1024	5	327	35.9	7.36	4652.6	6314.7	6267.9	4620.4	4081.8	4058.3	5555.8	4247.9
A0A024R6I	Serpin pep	MQLFLLLCI	37	16	531	16	406	45.6	9.26	30478.7	25865.8	26139.5	30958.3	33303.9	33812	29852	27414.4
Q16270	Insulin-like	MERPSLRA	58	16	582	16	282	29.1	7.9	38283.2	32001.3	27463.2	32943.9	37954.8	37956.2	29811.2	39312.4
A0A024RD	Secreted p	MRIAVICF(	45	11	554	2	287	32.3	4.55	77.9	67.3	75.4	76.2	78.8	98	74.1	86.6
P04279	Semenogel	MKPNIIFVL	53	23	236	17	462	52.1	9.29	16697.1	19411.5	10585.7	60356.9	13960	9603.9	8685.3	12317.9
P00738	Haptoglobi	MSALGAVI	46	19	454	19	406	45.2	6.58	23271.4	32501.8	26124.5	22935.7	18392	29883.6	86108.9	22466.7
A0A0G2JPF	Compleme	MRLLWGLI	20	34	216	1	1744	192.8	7.03	8787.4	9127.2	7777.3	8628.3	9221.3	9747	9042	7727.2
W0UV60	Ribonuclea	MVPKLFTS	20	3	946	3	161	18.3	8.73	40770.4	50060	31349.3	49333.6	39027.6	51040	35397.7	45540.4
P0C0L5	Compleme	MRLLWGLI	20	34	218	1	1744	192.6	7.27	332.7	299	292.7	364.7	344.8	388.6	450.4	344.9
O75882	Attractin O	MVAAAAAT	24	28	201	28	1429	158.4	7.31	12022.7	10543.3	9668.8	11707.2	12428.8	11456.7	10342.8	12416
A8K3U3	cDNA FLJ7	MQTCPLAF	14	2	618	2	248	27	7.43	29720.3	38789.3	23338.3	31760.1	30231.1	39678.5	27395.3	27654.4
P02790	Hemopexir	MARVLGAI	47	18	503	18	462	51.6	7.02	30288.1	32208	29317.9	26373	27211.1	29882.3	30681.3	27113.3
P10909	Clusterin O	MMKTLLLF	33	16	465	16	449	52.5	6.27	32485.9	31163	27176	31832.6	38559.5	33439.7	32173.1	29940.2
A8K7T4	cDNA FLJ7!	MAAEGWIY	47	16	724	16	356	40.2	6.95	51924.6	60344.1	37340.5	58631.6	57770.6	63919.2	64303.9	57175.3
P54802	Alpha-N-ac	MEAVAVA/	31	18	268	18	743	82.2	6.65	9813.9	7723.2	6953.8	8759.3	9550.4	8998.8	7625.2	8664.6
P63261	Actin, cyto	MEEEIAALY	42	14	229	5	375	41.8	5.48	16761	11559.1	10082.5	12801.4	16140	15279.7	13624	11164.5
E9PGN7	Plasma prc	MGAGPGR	24	13	593	13	543	59.5	6.76	38639.8	32778.5	29434.6	35619.4	43925.7	38370.4	33662.9	37295.4
S6BGD6	IgG L chain	MAGFPLLL	44	8	1430	1	235	24.8	7.24	1291.3	1704.5	1076.6	1469.4	1395.2	1573.3	1729.3	1427.4
P06396	Gelsolin OS	MAPHRPAI	28	19	403	19	782	85.6	6.28	22734.5	33262.8	17664.8	27384.1	28500	26508.5	25927.5	25485.9
P00747	Plasminoge	MEHKEVVL	40	27	256	27	810	90.5	7.24	16945.7	16062.8	14786.9	15645.1	16065.9	14859.2	17672.5	15357.1
B7ZKJ8	ITIH4 prote	MKPPRPVF	20	17	720	17	935	103.8	6.89	36663.7	32179.9	31430.2	38048	39069.7	40363	40159	35519.9
Q8N355	IGL@ prote	MAWTVLLI	43	8	1436	1	234	24.8	6.37	138.3	270.6	104	167.5	126.6	135	176.5	194.2
B4DPH5	cDNA FLJ5!	MVLNDVYI	34	21	324	1	533	61.4	5.33	22.1	22.6	36.2	46.9	25.7	16.7	38.3	31.5
A2NUT2	Lambda-ch	MTCSPLLLT	38	9	1468	3	235	24.6	7.62	86013.1	177227.2	72587.9	126455.5	97513.4	122835.2	118501.6	101417.1
Q6GMV8	Uncharacte	MAWTVLLI	42	8	1402	1	234	24.9	6.67	3551.6	6709.7	3229.9	4419.4	3762	5315.5	4920.8	4273.6

P05062	Fructose-b	MAHRFPAL	43	14	296	13	364	39.4	7.87	14342.3	10885.6	8662.9	10906.9	14848.6	12235.8	10101.6	10140.5
B7Z6Q5	Beta-galact	MPGFLVRI	26	15	197	15	725	81.8	7.94	10000.1	5876.3	6354.8	8239.7	8969.4	9596.4	7496.3	6953.9
Q569I7	Uncharacte	MDMRVPA	27	4	1473	1	189	20.7	5.27	145.7	136.2	114	110.6	119.4	158.6	169.9	134.5
S6B294	IgG L chain	MDMRVPA	28	4	1477	1	185	20.1	8.72	44.7	58	40.4	38.9	48.2	39.1	43.1	43
Q6GMX4	IGL@ prote	MAWSPLLI	38	7	1438	1	236	24.8	6.89	500.4	544.7	460.9	521	464.8	528.8	551	479.1
S6AWD6	IgG L chain	MRLPAQLL	34	5	1475	2	178	19.4	8.46	233.9	216.7	193.5	205.1	189.3	236.3	207.4	194.6
S6AWD3	IgG L chain	MRLPAQLL	23	4	1483	1	183	19.9	8.91	1018	1199.2	751.7	1491.4	928.4	992.7	1199.5	830
Q8NBJ4	Golgi mem	MMGLGNC	43	18	207	18	401	45.3	4.97	9235.2	7610.4	7144.5	9294.5	10319.8	9231.9	7727.5	10165.7
S6BGE9	IgG L chain	MRLPAQLL	23	4	1509	1	183	19.9	8.9	2212.7	2856	1550.1	2192.6	2079.4	2231	2508.7	2152.9
Q6IPQ0	IGL@ prote	MAWALLLI	38	7	1432	1	236	24.8	6.76								
V9HWI3	Cathepsin I	MQPSSLLP	39	13	309	13	412	44.5	6.54	15711.4	12359.9	11091.3	14318	17143.2	16500.5	14170.4	13018.8
Q6PIK1	IGL@ prote	MAWALLLI	35	7	1010	1	235	24.8	7.71	6290.4	8731	5542.3	6796.3	6542	7580.1	7764.1	6906.4
D6RF35	Vitamin D-	MKRVLVLL	53	23	262	23	476	53	5.52	17327.4	13533.7	24489.9	15861.2	15123.2	15500.5	16584.4	14294.4
V9HWD8	Epididymis	MSMLVVFI	33	12	342	12	495	54.2	5.86	21216.9	26602.6	21869.4	21043.9	20167.4	21694.9	31421	24056.7
P02749	Beta-2-glyc	MISPVLILFS	45	11	404	11	345	38.3	7.97	22534.1	19621.2	12893.6	20707.4	27931.5	20500.3	20266.6	26758.6
D9ZGG2	Vitronectir	MAPLRPLL	26	10	344	10	478	54.3	5.8	21742.7	22367.4	14848.5	24088.8	22575.8	22267.7	25547.6	21122.6
Q5CZ94	Putative ur	MAWTPLFI	32	6	971	1	234	25	7.27	135.2	121.7	147.8	161.2	102.2	148.6	136.4	145.8
Q6DHW4	Uncharacte	MAGFPLLL	35	6	969	1	237	25.1	7.99								
B3KS79	cDNA FLJ35	MKIHYSRQ	30	13	289	13	448	50.6	5.63	13162.9	14201.8	12691.8	14632.2	14459.7	13588.8	22531.2	16211.1
P07998	Ribonuclea	MALEKSLV	58	6	309	6	156	17.6	8.79	20596.6	20328.4	13152.6	21895.2	21754.4	19182.2	17946.9	25347.2
C9JF17	Apolipopro	MFKQLSCIS	33	8	1310	8	215	24.1	5.6	116695.1	109434.1	75109.3	109701.3	141500.2	116202.4	121145	125321.7
Q8WZ75	Roundabou	MGSGGDS	14	10	196	10	1007	107.4	6.64	14015.7	12359.8	11027.9	13649.4	14279	16103.8	13958.1	13999.1
M0R1F0	Prostate-sp	XFLTLSVTV	52	10	493	9	262	28.6	7.44	32571.8	40315.5	30526	26632.8	29508.2	16031.4	16496.1	34544.1
Q6N030	Uncharacte	MDWTWR	37	15	1075	6	518	57	8.05	5074.5	4738.1	5540.6	6024.4	6131	5122.6	7072.7	7211.3
Q8NFZ8	Cell adhesi	MGRARRFC	31	9	189	9	388	42.8	6.3	8883.2	8270.9	6499.1	9017.3	8820.9	9361.4	7196.8	9424.4
B7Z9B1	cDNA FLJ52	MKTPPGAS	20	12	469	12	760	83.4	5.12	23981.7	26643.4	16251.4	25265.4	25342.3	24623.6	22206.7	28059.6
O94919	Endonuclea	MGTARWL	29	12	227	12	500	55	5.71	8326.9	11662	6551.2	8645.8	8894.1	8187	8210.1	9024.3
P02753	Retinol-bin	MKWVWA	61	10	470	10	201	23	6.07	28771.5	58084.3	20772.3	29296.9	33777.9	30653.1	22817.5	26946
B7Z5V6	cDNA FLJ57	MGVRHPP	24	14	312	2	644	71.7	6.34	2519.2	1906.2	2285.5	1916.7	2317.2	2148.9	1547.7	2002.3
E9PR17	CD59 glyco	MGIQGGS\	37	5	1278	5	130	14.5	7.77	90140.3	94116.1	66869	98283.8	104681.9	107610.9	86285.3	112175.1
O00391	Sulfhydryl	MRRCNSGS	31	17	139	17	747	82.5	8.92	6795.3	6010	5442	6302.2	6408.7	6427.3	5843.9	5955.7
P27487	Dipeptidyl	MKTPWKV	31	23	143	23	766	88.2	6.04	9130.5	8022.4	7844.8	9113.7	9502.2	8394.4	7506.1	8683.5
H6VRG1	Keratin 1 C	MSRQFSSR	34	20	161	16	645	66.1	8.12	9476	6231.3	7005	5944.3	7754.1	8608.7	8670.3	9226.5
A8K335	cDNA FLJ76	MASPGRLL	35	12	311	12	318	36	7.42	17957	12883	11143.4	15620.3	18357.3	20282.1	15601.1	15333.5
B7Z8Q2	cDNA FLJ55	MFAGCFFF	24	10	409	10	433	46.6	6.28	29115	47211	22248.8	32847	34937.8	31691.9	33256.5	31137.6
B2R582	cDNA, FLJ9	MELWGAY	53	9	211	1	202	22.5	6.04	535	463.1	497.3	429.5	579.2	442.2	445.4	589
B2R888	Monocyte	MERASCLL	35	12	411	12	375	40	6.23	19350.3	34324.4	13265.4	21759.3	24036.9	22120.8	31587.4	22517.1
A8K3K1	cDNA FLJ78	MCDDEETT	30	10	187	1	377	42	5.39	1376.3	972.4	929.4	1205.8	1637.7	1245.8	1169.5	1132.8
B4E1Z4	cDNA FLJ55	MGPLMVL	18	17	125	3	1266	140.9	7.18	5327	7433.3	3756.4	4316	5048.3	4986.3	4293	4174.9
A8K6Z6	cDNA FLJ78	MKPNIIFVL	43	21	144	15	582	65.6	9.07	3808.1	4746.6	2950.2	13370.1	3415.1	3196.1	2643.1	2968.1
D9YZU5	Beta-globir	MVHLTPEE	78	10	328	1	147	16	7.28	44720.1	11673.8	7891.1	10959.3	20029.3	18593.9	20856.2	8175.3

O00468	Agrin OS=H	MAGRSHP	15	22	122	21	2067	217.1	6.39	6556.5	9170.1	5380.9	6510.6	6654.7	6845.8	6884.2	6549.9
E7ETH0	Compleme	MKLLHVFL	32	15	185	4	591	66.6	7.59	7030	6804.8	6327.9	6590.3	7519.4	7774.9	6675.9	7242.1
A0A1B0GU	Ig mu chair	GSASAPTL	30	13	404	13	474	51.9	6.15	23674.6	25076.2	19022.9	23987	25065.2	30377.4	22344.8	20735.1
B2RCQ6	cDNA, FLJ9	MPPVGGK	55	18	112	3	406	44.9	7.24	7840.2	5243.9	5523.3	5621.9	6657.2	6307	4825.4	5058.9
B7ZKY6	Membrane	MGKSESQ	31	21	138	21	750	85.5	5.91	7337.4	6212	6066.5	6723.5	7573.8	6489.3	5345.8	6746.4
A0A0E3XJU	E-cadherin	MGPWSRS	17	14	408	13	882	97.3	4.7	23890.7	28629.9	19631.3	25363	24789.7	30539	31002.3	30186.4
E7ET40	Urokinase-	MVFHLRTR	35	13	190	13	414	46.9	8.44	10043	10868	7972	9409.9	10646.9	10681.4	9738.5	8753.8
A8K6V6	N-acetylglu	MRLLPLAP	25	13	292	13	552	62.1	8.4	14130.6	14474.9	9677.9	15612.1	14594.5	14408.2	14409.1	14806.6
V9HW22	Epididymis	MSKGPAV	32	18	122	13	646	70.9	5.52	6176.3	4915.2	4402.9	5382.5	5784.9	5413.1	5273.7	5169.2
P28799	Granulins (	MWTLVSW	19	7	209	7	593	63.5	6.83	13200.6	8566.9	8117.2	10492.5	10470.4	10819.4	9760.5	8962.9
B0YJC6	Vitamin K-	MAGCVPLL	33	13	247	13	421	46.9	6.04	18393.1	16104.7	13385.9	18155.4	15686.5	19476.2	15480.4	16253.9
A2KBC4	Anti-TN-C s	EVQLLESG	43	7	240	2	238	25.1	8.41	5443.3	4535.9	4904.6	5186.3	5280.6	5058.7	5224.3	4996.3
P78492	Inter-alpha	LWAFDAV	75	3	1658	1	51	5.7	4.89	676.4	874.5	417.2	801.5	703.3	628.9	910.5	820
Q7Z3B1	Neuronal g	MDMMLL	42	11	177	11	354	38.7	6.21	4784.5	5127	3566.5	5021.4	5158.7	5011.7	4610.3	5405.1
S6BAR0	IgG L chain	MAWTPLF	31	6	1319	2	216	23.1	7.69	1661.8	2367.8	1268.6	1681.7	1674.3	3364.4	2416	1988.2
P13645	Keratin, ty	MSVRYSSS	31	18	186	13	584	58.8	5.21	11061.6	7528.5	9154.3	7392.4	10137.9	11772.1	10809.6	11461.9
Q9Y6R7	IgGFc-bind	MGALWSW	11	24	90	24	5405	571.6	5.34	4069	3160.2	2487.8	2981.7	5357.1	2513.9	3199	4182.5
A0A024QZ	Prosaposin	MYALFLLA	27	14	579	14	524	58.1	5.17	51916	36858	28343.4	40853.7	45275.1	43156.2	50370.4	44266.4
P06870	Kallikrein-1	MWFLVLC	23	6	493	6	262	28.9	4.83	29532.8	26696.4	24544.6	30067.2	37889.1	27163.4	19938.5	31904.2
A0A024R9	Serpin pep	MYSNVIGT	36	16	193	16	464	52.6	6.71	7926.4	9396.9	10940.4	6630.6	6582.5	6966.3	7629.3	6197
A0A024R2	C-type lect	MELWGAY	53	9	191	1	202	22.5	5.67	9181.9	7965.2	7486.8	8543.1	10248.6	8645.4	8053.7	10624.9
B2RBF5	cDNA, FLJ9	MSRPQLR	32	10	163	10	385	43.8	6.64	9872.2	10090.3	7463.7	10399.1	9454	10323.2	10729.7	9972.7
X6R868	Bile salt-ac	MLTMGRL	23	16	161	16	756	79.6	5.34	13296.1	8155.6	9041.5	11132.9	10893.4	13786.7	8731.9	10342.8
A0A087W	Tenascin-X	MMPAQYA	17	20	86	1	1545	165.4	6.02								
Q15828	Cystatin-M	MARSNLPL	50	5	278	5	149	16.5	8.09	7456	14974.4	6224.4	8778.4	8816.8	8199.9	6846.3	7481.9
Q5FWF9	IGL@ prote	MAWTPLL	42	8	690	3	232	24.8	5.59	2832.6	5027.3	2277.8	3809.7	3261.9	3448.2	3768.6	3111.9
P22792	Carboxype	MLPGAWL	22	11	195	11	545	60.5	5.99	8418.9	7375.9	6103.8	8306.7	10519.2	10166.9	8366.4	9129.5
B4DJQ8	cDNA FLJ5	MGAGPSC	34	14	177	14	446	50.1	6.99	9491.6	7696.2	7051.4	8465	9227.7	8902.9	7872.9	7815.2
E7ETN3	Uncharacte	MGPLMVL	17	15	92	1	1115	123.9	6.8	47.1	42.5	34.2	44.4	44.1	47.4	62.1	56.9
O75594	Peptidogly	MSRRSMLL	45	6	227	6	196	21.7	8.59	11315.7	10968.1	8389.1	10397.2	11176.1	10271.8	9934.7	10051.6
P60174	Triosephos	MAEDGEE	59	15	89	15	286	30.8	5.92	6322.4	4996.5	3888.3	5335.4	6184.5	5511.8	5547.9	5034.8
A0A075B6	Ig lambda-	XQPKAAPS	48	4	1262	1	106	11.3	8.29	134.1	314.6	119.4	195.9	191.2	150.6	249.4	126.3
P24855	Deoxyribor	MRGMKLL	23	5	348	5	282	31.4	4.91	19120.1	10550.1	14451.1	18482.7	19902.9	20304.1	14070.1	19469.6
A8K2P8	cDNA FLJ7	MEAARPS	17	14	99	14	901	99.9	5.34	4005.2	4797.7	3005.9	4284.3	4544.7	4309.2	4329.2	4611.3
Q6LAM1	Heavy chai	KVTYTSQE	45	12	123	1	321	35.9	7.59	264	172.8	174.9	218.4	264.4	229.1	317	272.9
D9IAI1	Epididymis	MPVDLSKV	80	11	193	11	187	21	7.53	10704.4	8666.2	8083.9	9207.2	11407.9	9249.3	8762.1	10023.5
A8K7G6	cDNA FLJ7	MAQTSSY	55	9	294	7	166	18.7	5.53	20747.4	67081.8	15740.4	21956.8	29098.7	25074.5	21249.8	24072.4
A0A024R3	Apolipopro	MKAAVLTL	60	16	172	16	267	30.8	5.76	14099.8	8339.7	10752.4	7017	6795.4	13017.8	9385.9	5583.4
A0A0K0K1	Cystatin O	MAGPLRA	37	6	302	6	146	15.8	8.75	23609.1	20362.2	17781	20549.9	25344.5	22440.5	20095	22034.6
P06733	Alpha-enol	MSILKIHAR	39	15	140	15	434	47.1	7.39	9197.9	7512.7	6104.5	7530.6	9779.6	8699.9	8416.6	7328.1
A0A024R0	Matrix-rem	MALPSRILL	33	14	184	14	450	49.7	6.92	14135.6	13360.5	9861.6	13081.8	14067.5	13527.6	14201.3	13392.9

P19652	Alpha-1-ac	MALSWVL	47	8	469	6	201	23.6	5.11	28760.9	42980.5	28097.3	30432.2	25362.8	22890.2	54229.9	32592.2
Q7Z379	Putative ur	KHLWFFLL	33	11	357	3	478	51.6	6.52	2416.1	1625.7	1794.5	2023.5	1993	2266	2332.7	2134.7
B0BCY7	Glutamyl a	MNFAERE	21	20	145	20	957	109.2	5.47	6396.6	4871	4526.1	7544.2	7169.7	6138.2	6823.2	6108.5
A0A125QY	GCT-A9 ligl	DIQMTQS	32	2	172	1	107	11.6	4.65	2491.2	3158.7	1686.8	3206.8	2375.6	2437.5	3373.3	2485.4
P02649	Apolipopro	MKVLWAA	48	15	106	15	317	36.1	5.73	6715.5	5542.8	4969	5478	6541.8	6263.6	5132.9	4851
P11117	Lysosomal	MAGKRSG	29	12	195	12	423	48.3	6.74	11102.1	8212.2	7771.6	9738.9	9839.5	11705	8307.8	9940.3
P06727	Apolipopro	MFLKAVVL	54	21	120	21	396	45.4	5.38	8194	8534.3	6329.7	5770.7	6111.2	7110.2	6475	5285.4
O76076	WNT1-indu	MRGTPKT	36	6	81	6	250	26.8	7.88	2354.8	2240.5	1497.1	2188.9	2228.2	1983	1876.3	2026.8
Q14508	WAP four-	MPACRLG	61	4	231	4	124	13	4.84	12289.6	10492.9	8609.3	12185.4	13100.8	13006.5	15482.9	13168.2
P04083	Annexin A1	MAMVSEF	51	16	132	16	346	38.7	7.02	3502.7	3667.8	2825.3	5595.3	4147.6	4064.7	5350.8	3672
Q9UNN8	Endothelia	MLTTLLPIL	29	6	386	6	238	26.7	7.18	19919.3	17402.5	15700	21225.8	18474.6	20438.6	21937.6	21715.2
P18065	Insulin-like	MLPRVGC	49	12	145	12	325	34.8	7.5	6669.4	6714.4	5207	5987.2	6522.1	7067.8	6039.7	5620.8
P14543	Nidogen-1	MLASSSRI	10	12	141	2	1247	136.3	5.29	6662.7	6247.1	6194.6	6379.3	6386.4	6458	6279.2	6487.1
Q7Z5L0	Vitelline m	MERGAGA	40	5	427	5	202	21.5	5.07	14838	11073.3	10759.1	11735	12882.7	15612.3	14948.9	14878.8
B4DM05	cDNA FLJ5	MLASSSRI	17	12	155	2	726	79.3	4.97	3373.5	3211.1	1987.1	3039.5	3779.5	3380.4	2472.9	3873.7
P02671	Fibrinogen	MFSMRIV	17	12	100	12	866	94.9	6.01	4743	3637.4	2688.6	4047.4	4198.3	4620.4	5013.4	4234.1
A0A024R5	Arylsulfata	MSMGAPR	25	8	154	8	509	53.8	6.07	5705.9	4819.6	3916.7	5213.7	4961.3	5588.7	4636.9	4624.8
B4DNW0	cDNA FLJ6	MWGCQA	33	14	98	14	498	56	7.03	6735	4362.2	4447.6	4353.5	5904.8	6104.6	4342.9	4646.2
P16870	Carboxype	MAGRGGS	31	15	130	15	476	53.1	5.14	6963.6	8192.7	6190.8	6365.7	7394.1	5195.9	4811.7	6000.8
P08582	Melanotra	MRGPSGA	33	19	77	19	738	80.2	5.94	3034	2998.4	2663.6	3365	3292.2	2782.2	2661	3211.1
Q99715	Collagen al	MRSRLPPA	6	13	95	13	3063	332.9	5.53	5615.7	4543.5	4483	5307.4	5506.9	5496.1	5446.3	5685.6
P08294	Extracellula	MLALLCSC	37	7	177	7	240	25.8	6.61	6185.7	6487.6	5858.6	6718.8	7256.2	7641.9	6378.4	7946.4
Q6EMK4	Vasorin OS	MCSRVPLL	15	9	222	9	673	71.7	7.39	13637.6	11660.9	11262.7	12179.6	13908.9	12904.8	11381.6	11193
P21333	Filamin-A	MSSSHSRA	8	15	78	15	2647	280.6	6.06	3358.3	3156.8	2544.4	3219.8	3486	3310	3494.3	3522
A0A0X9UW	GCT-A5 ligl	DIVMTQSP	54	5	236	2	113	12.5	6.58	8308.8	11073.3	6619.9	9396.4	8991.8	10450.5	11201.3	10309.4
P02750	Leucine-ric	MSSWSRQ	28	9	294	9	347	38.2	6.95	12613	24606.6	10453.9	15722.9	13511	13859.2	35646.8	19100
P19440	Gamma-gl	MKKKLVVL	22	11	138	11	569	61.4	7.12	6381.6	5268.6	4712	5852.7	6820.6	7100.7	4533.4	5953.7
P30530	Tyrosine-p	MAWRCPR	10	7	212	7	894	98.3	5.43	8354.3	8598.2	6514.2	8759.5	8655.7	8945.3	8113.3	8678.5
O00187	Mannan-bi	MRLLTLLG	10	7	556	7	686	75.7	5.63	28174.7	31325.3	20703.3	27106.5	28729	32659.2	37673	29491.9
Q9NZP8	Compleme	MPGPRVW	25	10	81	2	487	53.5	7.2	4612	4221.7	3471	4781.8	5028.8	4536.8	5447.1	4947.3
Q96FE7	Phosphoin	MLLAWVQ	21	5	638	5	263	28.2	5.01	48791.1	50194.9	33426.8	56833.1	56993.2	54600.6	45056	60639.2
Q4TZM4	Hemoglobi	TPEEKSAV	89	8	135	2	101	11	6.52	799.4	200.7	152.1	169.2	391.1	392.4	398.7	134.5
P16070	CD44 antig	MDKFWW	9	6	582	6	742	81.5	5.33	46121.7	44805.3	32947.2	52623.7	52530	56809.7	50556.8	61721.5
Q96PD5	N-acetylm	MAQGVLW	23	9	99	9	576	62.2	7.55	6845.7	4899.5	4304.9	6124.7	4679	4731.7	5866.3	4574.7
Q59EQ1	Cadherin 1	PKMKENY	25	14	92	14	798	88.1	4.93	5392.5	6145.5	4164.8	5892.6	5570.7	5926.4	6746.7	6154.1
Q59EJ3	Heat shock	LRSCCDSPL	24	15	85	11	709	77.4	6.3	1688.7	1313.5	1123	1448.6	1430.5	1427.3	1312.5	1260.2
B4DPF0	cDNA FLJ5	MPPVGGK	53	16	75	1	365	40.5	6.93								
H7BY55	Compleme	MTVARPS	24	12	331	12	550	58.9	8.78	16870.9	18960.2	11991	17699.9	18951.4	18436.9	20402.1	23131.9
A0A024R5	Folate rece	MAQRMTT	31	7	206	7	257	29.8	7.97	11850.3	12237.3	8645.7	12932.5	13761.3	12888.9	10740.6	13354.9
Q96RW7	Hemicentir	MISWEVV	2	6	115	6	5635	613	6.49	4630.5	4397.2	3690.3	4410.4	4345.8	4438.6	3645.6	4413.3
Q4LE33	TNC varian	SLLCASKSP	7	13	71	13	2233	244.2	4.94	5995.7	4917.8	4043.6	5283.6	5542.5	5062	5917.9	5727.9

P00558	Phosphogly	MSLSNKLT	39	14	75	14	417	44.6	8.1	3378.6	2678.5	2414.9	2926.4	3242.2	3386	2793.2	2517
P05543	Thyroxine-	MSPFLYLVL	31	13	123	13	415	46.3	6.3	8329.4	6472.1	7373.6	8439.7	11253.1	10374.4	12476.3	10225
D3DQU2	Tripeptidyl	MTADPRK(	20	8	82	8	572	62.2	6.65	4449.1	3953.4	3176.4	3856.9	3914.5	5232.1	3194.9	3143.2
Q59EP1	Annexin (F	VKDLTMSY	35	15	108	15	510	54.9	7.27	5871.8	5467.6	4177.8	5309.7	6345.5	5208	5078.5	4902.7
P33908	Mannosyl-	MPVGGLLP	24	15	104	15	653	72.9	6.47	4720.8	4095.7	3316.8	4006.9	4866.9	4740.6	3971.6	4125.6
B7ZW00	COL6A3 pr	MRKHRHLP	6	14	120	1	2570	278	8.15	764	902.1	576.2	961.7	1286.6	832.8	1001.7	862.7
P60022	Beta-defer	MRTSYLLLF	34	1	111	1	68	7.4	8.6	5318.3	7551.5	5017.4	6151.3	4069.6	8211.8	7714.7	5562.9
Q9UL78	Myosin-rea	EIVLTQSPG	60	4	325	1	109	11.6	8.29	5435.1	5646.1	4489.4	5065.1	5431.4	6108.6	5699.8	5804.5
S6BGE0	IgG H chair	MEFGLSWV	30	6	284	0	300	32.2	7.85	619.1	614.4	644.3	620.3	634.2	631.1	726.6	665
Q9HCU0	Endosialin	MLLRLLLAV	23	13	182	13	757	80.8	5.35	12401.7	10911.9	9069.8	13278.6	13421.9	12460.9	10600.7	13413
A0A1K0GX	Globin C1 (	MSAARPGV	50	6	182	6	177	19.2	9.77	29618.2	9508.2	7413.2	8253.1	15153	13904.8	15770.1	6799.3
P43121	Cell surface	MGLPRLVC	22	11	69	11	646	71.6	5.76	3270	3254.3	2493.3	3249.6	3281.8	3803.3	3274.2	3485.7
A0A075B6	Ig alpha-2 (	ASPTSPKVF	39	10	295	3	340	36.6	6.27	692.8	593.2	599.1	790.1	732.1	521.6	551.7	590.3
Q96NY8	Nectin-4 O	MPLSLGAE	25	10	86	10	510	55.4	5.38	4708.4	3999.8	3850	4385.7	4907.4	5536.9	4613.6	4612.5
A0A024RD	SPARC-like	MKTGLFFL(	30	15	76	15	664	75.2	4.81	3793.8	4046.1	3261.7	3774.7	4534	3652.7	3736.7	3687.9
G3V4U0	Fibulin-5 O	MRSGNCR(	18	7	111	5	453	50.8	4.77	5074.9	4457.3	3773.5	4585.5	5020.6	4681	4490.6	4667.9
A0A0A1TT	Lutheran b	MEPPDAPA	29	14	78	14	628	67.3	5.81	4202.2	3335.2	3038.3	3613.9	3773.6	3867.2	3478	3736.7
X6R8A1	Carboxype	MTSSPRAP	20	9	131	9	498	56.2	6.61	9108.3	4903.2	5425.3	7045.1	7273.7	8585.6	6819.7	5613.9
P19320	Vascular ce	MPGKMVV	18	12	99	12	739	81.2	5.22	4789.2	7333.7	4646.3	4838.7	4779.5	4553.1	5918.9	4993.5
A0A024R0	Macrophag	MTAPGAA(	21	10	138	6	554	60.1	5.29	6218.2	6126.4	4574	6227	6485.1	6718.1	7291.7	7324.9
P12111	Collagen al	MRKHRHLP	5	14	113	1	3177	343.5	6.68	7415.6	7912.6	5452.6	7785.7	8009.2	7299.1	7169.4	7162.4
Q6UX06	Olfactome	MRPGLSFL	21	10	125	10	510	57.2	5.69	5003.2	4025.9	3660.8	4811.1	9592.3	4072.6	4205.4	6418.7
A8K2T4	cDNA FLJ7	MKVISLFIL	13	10	107	10	843	93.3	6.51	7452.5	7214.7	5493.9	7679.1	7694	8065.7	7781.9	7732.9
A0A024RC	Cadherin 2	MCRIAGAL	12	7	145	7	906	99.7	4.81	4051.2	3790	2964.5	4320.6	4196.5	4337.4	4671	4857.4
P51884	Lumican O	MSLSAFTLF	36	11	162	11	338	38.4	6.61	7642.1	10154.5	6257	8331.2	9397.3	8790.4	8770.9	7967.7
Q9BYE9	Cadherin-r	MAQLWLS	12	14	86	14	1310	141.5	4.5	3781.4	3640	3191.5	3753.9	3913.1	3706.3	3380.4	3705.1
Q9UKU9	Angiopoiet	MRPLCVTC	23	11	129	11	493	57.1	7.53	7634.6	4390.3	6199.6	7477.6	6215.8	10462.1	5680	6197.8
P04406	Glyceralde	MGKVKVG	30	9	84	9	335	36	8.46	4985.1	4364.4	3597.4	4289.4	5148.7	5397.6	4728.8	4050.2
A4D2D2	Procollage	MLPAATAS	18	6	92	6	449	47.9	7.43	2648.9	2896.3	2079.8	2789.7	2935	2604.4	2635.7	2776.7
Q9BXP8	Pappalysin	MMCLKILR	12	17	76	17	1791	198.4	5.47	3162.2	2628.6	2362.7	2984.5	3081.3	3160.9	2770.2	2528.1
P01591	Immunoglo	MKNHLLFV	42	6	301	6	159	18.1	5.24	21643	17461.8	13951.6	20737.8	23622.7	19619.8	18930	22042.9
A0A024R3	Cathepsin	MWQLWA	27	7	172	7	339	37.8	6.3	11295	8769	7231.1	13199.4	12385.8	11797	8858.7	9181.8
B4DVA7	Beta-hexos	MTSSRLWF	27	14	112	13	540	62	5.26	5384.5	3289.8	3831.4	4917.7	6053.1	5639.6	4600.1	4755.1
Q03403	Trefoil fact	MGRRDAQ	28	4	119	4	129	14.3	5.81	8058.9	7228.5	7331.7	7912	8480.1	10380.9	7841.1	7971.6
A0A140VJ	Testicular t	MLPGLALL	20	12	88	12	770	86.9	4.82	6109.1	5411.3	4451.7	5954.7	6067.9	5151.7	5195	6176.6
Q969P0	Immunoglo	MGALRPTL	14	7	120	7	613	65	8	5478.2	5763.1	4520.6	5536.4	6001.2	6068.8	5037.5	5559.5
Q96SA9	Anti-strept	DIQMTQSP	49	3	130	1	107	11.5	8.85	59	62.1	34.4	54.8	59.8	60.9	70	56.4
A0N071	Delta globi	MVHLTPEE	41	6	228	1	147	16	8.05								
A0A024R9	Plasma glu	MKFLIFAFF	25	9	105	9	472	51.9	6.18	5450.1	4178.2	3922.2	4977.5	5938.4	6070.9	4969.1	4634
P43251	Biotinidase	MAHAHIQ(	18	8	76	8	543	61.1	6.25	4007.9	3433.6	3371.9	3544.7	3927.9	3892.8	3499.8	3739
D3DPK5	SH3 domai	KQLKPSKAI	14	5	147	5	257	26.8	8.38	7301.7	9659	5353	8906.8	8228.3	9216.4	7713.3	8402.9

Q14767	Latent-trar	MRPRTKAF	4	6	32	6	1821	194.9	5.19	2487.2	2812.7	1639.6	2847.3	2505.4	2936.5	2437.9	2717.6
Q92956	Tumor nec	MEPPGDW	19	3	51	3	283	30.4	7.15	2459.8	1976.6	1927.6	2293.4	2205.1	1971.1	2168	2257.6
A0A087W\	Mucin-1 O:	MTPGTQSF	6	6	145	6	1262	122.9	7.56	7146.2	6532	5873.5	7307.5	8981	7333.4	6572.8	8057.4
P07195	L-lactate d	MATLKEKL	30	9	126	8	334	36.6	6.05	10143.2	7229.1	6568.5	8089.9	11867.5	9207.9	7653	7645.5
A2MYE1	A30 (Fragn	DIQMTQSF	47	3	116	1	96	10.4	8.5	4420.3	5418.3	5346.5	4195.9	4712.5	4510.6	5181.3	4963.5
Q4W4Y1	Dopamine	MATFISVQ	26	22	55	22	868	96	6.52	2555.9	2251.8	2095.3	2399	2771.2	2422.2	1895.2	2352.5
D3DSQ1	N-acylsphir	MNCCIGLG	22	13	115	7	546	60.6	8.81	5411	4681.5	3678.5	5312.2	5482.3	4348	4323.6	4530.2
A1A4E9	Keratin 13	MSLRLQSS.	25	14	107	9	458	49.6	4.96	6278.1	2943.1	3674.5	2963.2	3484.1	5475.3	6283.7	3225.2
P07996	Thrombosp	MGLAWGL	11	10	75	1	1170	129.3	4.94	829.5	950.9	603	949.9	973.4	1055	955.2	862.4
Q07507	Dermatopo	MDLSLLW\	38	6	124	6	201	24	4.82	7663.8	8305.1	5661.8	7674.5	8157	8296	6186.1	7932.2
O43278	Kunitz-type	MAPARTM	30	16	65	16	529	58.4	6.29	2618.5	2388.3	1943.2	2441.5	2672.2	2229.9	2272.6	2534.5
B2RCM5	cDNA, FLJ9	MLPCASCL	20	7	70	7	443	49.3	4.94	2778.8	2565.7	2057.3	2724.9	2740.7	2594.7	2391.2	2574.2
B2R815	cDNA, FLJ9	MHLIDYLLI	28	12	77	12	427	48.5	7.75	3952.5	3292.2	2683.9	3455.1	3860.7	4139.3	3346.8	3411.3
Q9UL83	Myosin-rea	EIVMTQSP	36	2	58	1	108	11.8	8.68	1858.1	1701.5	1872.9	1877.1	1697.8	1765.9	1933.1	1795.6
B2R9F2	cDNA, FLJ9	MPLLLYTCI	23	8	127	8	405	45.1	6.04	6586.5	6752	6012.8	6470.5	6705.7	6742.9	7251.2	6301.6
Q65ZC9	Single-chai	QVQLVQSC	25	5	97	2	240	25.6	9.11	3911.8	3290.7	3307.4	3863.8	3751.6	3579.6	3859.5	3455.5
B2R6J2	cDNA, FLJ9	MPKPINVR	26	18	97	10	586	69.4	6.27	4918.4	4153.1	3316.6	4704.9	5856.9	5435.1	4215.5	4708.4
Q6UVK1	Chondroiti	MQSGPRPI	5	9	75	9	2322	250.4	5.47	4253.8	3586.2	3342.5	3778.9	4146.7	4047.6	3405.6	4052.6
A0A024R9	Thrombosp	MGLAWGL	11	10	67	1	1170	129.3	4.94	2782.9	2760.4	1885.2	2612.4	2562.7	2686	2439.8	2992.3
P06312	Immunoglo	MVLQTQV	30	3	150	1	121	13.4	5.25	695.6	596.1	541.4	617.4	633.4	772.7	811.4	765.8
Q6UXG3	CMRF35-lik	MRLLVLLW	13	4	114	4	332	36	5.92	10138.3	8746.7	7962.6	9517.2	10915.3	10986.5	9753.3	12761.2
Q9UL81	Myosin-rea	DIQMTQSF	51	3	104	2	107	11.5	8.48	34.3	62.8	26.6	38.9	30.3	38	34.1	23.2
D3DVW9	Protein tyr	MEPAGPAI	35	14	60	3	504	54.9	6.98	3944.4	3701.6	3299.8	3873.7	4607.8	4428.1	4253.1	3750.6
V9HWB8	Pyruvate k	MSKPHSEA	30	13	72	13	531	57.9	7.84	4612.1	3926.1	3562.7	4358.7	4392.5	4571	5137.3	3511.6
B2R4M6	Protein S1(	MTCKMSQ	52	6	80	6	114	13.2	6.13	6589.9	5338.6	3495.5	6009.2	6572.4	6127.6	9257.9	4812.2
A0A024R2'	Dystroglyca	MRMSVGL	14	9	54	9	895	97.5	8.56	2598.5	2103.5	1831.5	2110.9	2400.7	2450.8	1990	2219.6
G3V3Y2	Fibulin-5 (F	MPGIKRILT	47	3	80	1	91	9.9	6.37	188.9	217.1	121.8	192.6	184.4	179	237.3	255.6
Q9UL70	Myosin-rea	DIQMTQSF	48	3	106	2	108	11.6	9.36	101	108.3	81.7	91.1	95	97.9	89.3	84.6
O00533	Neural cell	MEPLLLGR	20	19	54	19	1208	135	5.76	1722.1	1576.4	1445.9	1956.5	1877.3	1718.9	1643.1	1884.3
X6R8F3	Neutrophil	MPLGLLWI	44	6	125	6	200	22.8	8.5	6977.9	5396.3	3983.1	5926.6	10723.4	5612.7	7023.2	6613.9
A0A0K0K1I	6-phospho	MAAPAPGI	51	10	94	10	258	27.5	6.05	4299.8	4210.3	3104.4	4061.4	4581.4	4552.2	4135	3608.4
A2NB45	Cold agglut	DIVMTQSP	41	3	156	2	113	12.4	8.48	1719.5	2595.5	1320.5	1746.8	1749.3	1796.2	1897.8	1753.5
E9PNQ8	Thy-1 mem	MNLAISIAL	24	4	70	4	165	18.2	9.16	2619	3111.1	2023.6	2671.5	2798.9	2710.3	2412.1	2848.4
P09668	Pro-cathep	MWATLPLI	23	7	69	7	335	37.4	8.07	3818.3	3708.1	3114.7	4093.6	4162.3	4117.3	3927.9	3752.6
A0A109PW	MS-C1 ligh	DIQMTQSF	26	2	109	1	108	11.8	6.57	632.9	643.8	402.3	598.6	540.1	486.2	572.3	534.8
Q06830	Peroxiredo	MSSGNAKI	45	9	94	8	199	22.1	8.13	5109.6	3996.1	3116.1	3967	5034.6	4278.5	3861.5	4003
A0A140VJ\	Sushi doma	MKPALLPW	26	12	56	12	822	90.2	6.28	2316.1	1852.6	1772.3	1980	2065.6	2159.8	1559.9	2019.6
Q6UXB8	Peptidase i	MHGSCSFL	17	7	121	2	463	49.4	5.39	4194.7	6895.6	3273.1	4707.7	3892.8	4161.6	5028.4	5069.6
P26038	Moesin OS	MPKTISVR\	26	17	87	9	577	67.8	6.4	1900.3	1419.9	1609.4	1648.8	1863.5	1982.3	1550.3	1515.3
B4DUV1	Fibulin-1 O	MATHQKD	21	9	52	2	641	70.1	5.26	3808.6	4108.4	3064.4	4002.7	3563	3644	3855.4	4051.8
P14384	Carboxype	MDFPCLW	27	11	91	11	443	50.5	7.36	7591.3	6086.5	5482.5	7051.3	7146.8	6982.5	6406.9	7514.5

A0A0X9TD	MS-D1 ligh	EIVLTQSPA	38	4	217	1	107	11.6	8.51	6922.7	8683.4	6141.7	7193.8	6887.4	8061	7558.7	6873.1
A0A024R9	Nephrobla	MQSVQSTS	26	8	62	8	357	39.1	7.72	3194.3	3252.1	2459.5	3488.8	3041.5	2950.5	2732.1	3228.5
P35908	Keratin, typ	MSCQISCKS	17	11	66	5	639	65.4	8	823.2	575.3	800.4	610.1	771.6	850.1	708.4	884.5
V9HWB4	Epididymis	MKLSLVAA	27	14	63	11	654	72.3	5.16	1085.3	908.9	882.4	970.4	1146.5	1092	995.2	1025.7
P43652	Afamin OS	MKLLKLTG	21	13	95	13	599	69	5.9	6555.5	5167.9	9319.1	6123.9	5821.8	6216.2	6424.9	5833.9
A2NI60	BRE (Fragm	DIQMTQSP	34	2	101	1	108	11.9	4.56								
A0A024R1	Contactin 1	MKMWLLV	19	16	63	16	1018	113.2	5.9	2593.6	2385	2125	2493.1	2662.2	2498.9	2137.7	2524.1
Q9Y5Y7	Lymphatic	MARCFSLV	18	6	249	6	322	35.2	8.28	13497.1	21240	9204.5	20244.2	18007.5	18504.4	26382.3	22274.4
A0A024R5	Annexin OS	MSTVHEIL	37	11	55	11	339	38.6	7.75	2133.6	2179.2	2212.7	2570.7	2318.8	2357.3	2382.2	2178.1
Q7Z7M0	Multiple ep	MALGKVLA	7	16	63	16	2845	302.9	6.87	2286.2	2037.4	1769.5	2318.4	2546.4	2450.9	1908.6	2118.1
A0A0X9UW	MS-B1 ligh	DIQMTQSP	32	2	101	1	107	11.5	7.96	22	19.9	16.8	14.9	11.5	13.4	14.5	11.2
Q92520	Protein FA	MRVAGAA	40	8	65	8	227	24.7	8.29	3680.4	4972.7	3158.2	3505.7	4087.7	3307.6	3179.6	3355.8
P17936	Insulin-like	MQRARPT	29	6	93	6	291	31.7	8.69	3835.1	3423.4	2883.5	3101.8	4083.1	4204.2	3949.8	3871
S6C4Q9	IgG L chain	MAWALLL	32	6	611	2	217	22.8	6.76	521.1	715.4	471.4	665.7	602.4	568.8	596.1	532.7
Q13228	Selenium-b	MATKCGN	35	13	83	13	472	52.4	6.37	3077.2	2877.9	2469.5	3024	3094.8	3213	2905.3	3078.1
Q9UL90	Myosin-rea	EVQLVESG	50	5	99	2	113	12.4	8.44	744.5	577.6	711.5	632.5	880.3	652.9	689.8	667.8
B2RE74	cDNA, FLJ9	MADQAPF	24	6	53	6	338	36.8	6.68	2126.8	1580.9	1895.1	1611.2	1945.1	1668.7	1386.4	1428.4
Q9H804	cDNA FLJ14	MPGPRVW	22	8	58	1	438	48	7.46	307.3	230.2	287.6	290.7	183.2	317.8	358.9	216.2
V9HW53	Dimethylar	MGTPGEG	48	10	55	9	285	29.6	6.01	2501.4	2391.8	1909.8	2285.5	2782.2	2671.6	2377.4	2336.9
Q9UHL4	Dipeptidyl	MGSAPWA	21	8	67	8	492	54.3	6.32	2840.9	2170.5	2404.5	2754.5	2972.2	3067	2389.2	2357.4
O00241	Signal-regu	MPVPASW	29	10	59	7	398	43.2	6.52	3648.6	3977	2785.2	3271.7	3353.4	3901	3816.9	3846.2
Q9HAT2	Sialate O-a	MVAPGLVL	21	10	95	10	523	58.3	7.33	5690.4	5070.3	3857.9	5266.1	6201.3	5169.4	5246.1	5170.6
B7Z9B8	cDNA FLJ56	METSLSLES	6	5	104	5	1210	129.3	4.87	5761.1	6171.9	3949.3	5993.9	5556.2	5967.5	4806.3	5298.8
Q96DA0	Zymogen g	MGAQGAC	30	5	102	5	208	22.7	7.39	5922.7	4720.3	3676.1	5244.9	6937.1	4787.5	4561.2	6621.2
A8K7Q1	cDNA FLJ7	MPPSGPRC	30	12	55	12	461	53.9	5.25	3709.7	2704.6	3207.6	2838.6	3151.2	3331	2708.4	3019.9
V9HWE9	Epididymis	MPPYTVVY	55	10	60	10	210	23.3	5.64	2724.2	2283.9	1889.5	2734.4	2881.9	2660.9	2578.8	2354.8
P00995	Serine prot	MKVTGIFL	54	4	91	4	79	8.5	7.61	3518.1	4646.8	3072.8	3937.2	4284.3	4482	4145.1	4616.6
P23142	Fibulin-1 O	MERAAPSR	16	8	48	1	703	77.2	5.22								
J3KPF3	4F2 cell-su	MELQPPEA	20	11	44	11	631	68.1	5.05	2481.6	1848.5	1686	2022.9	2327	2371.8	1878.3	1964.2
P01023	Alpha-2-ma	MGKNKLLH	14	17	49	17	1474	163.2	6.46	2846.8	1790.8	1615.2	2120.8	1764.6	2447.4	1904.3	1389.4
A0A146E5	Mesenchym	MQELHLLW	18	6	67	6	428	46	5.15	3860.1	3901.6	2476.7	4008.5	4118.3	4203.8	3991.6	3675.7
Q6LES2	Annexin (F	MAMATKG	46	13	47	12	321	36.1	6.13	2260.1	1675.5	1802.2	2131.6	2170.9	2216.3	1804.7	1926.9
V9HW12	Epididymis	MASGNAR	41	10	40	9	198	21.9	5.97	2931.3	2180.4	1703.8	2218.3	2695.6	2416.8	2170.5	2198.6
A0A087WV	Neural cell	MLQTKDLI	11	9	123	9	884	97.3	4.93	6867.7	6201	4723.8	6512.9	7057.6	7053.3	6276.5	7327.9
P05109	Protein S10	MLTELEKA	56	7	131	7	93	10.8	7.03	11368.9	8367.2	5845.8	10957.3	11092.3	8418.2	16271.7	7687.1
A0A0S2Z42	HCG20398	MASTSTTIF	24	14	53	6	564	60	8	2751.2	1418.1	1540.3	1412.6	1874.5	2630.1	3741.2	2019.2
A0A0G2JLV	Leukocyte-	MSPHPTAL	16	5	135	2	287	31.5	5.78	9309.4	8803.8	7112.7	10296	10161.5	10274.9	9720.2	10954.7
M9MML0	Fc of IgG lo	MGGGAGE	18	6	107	2	290	32.7	8.1	2065.5	1762.7	1408.9	2087.8	1902.6	2406.7	1841.5	2128.2
P17900	Ganglioside	MQSLMQA	26	6	366	6	193	20.8	5.31	26291.2	37220.9	17844.8	33707.9	36274.8	30110.7	45241.4	30307.1
Q14766	Latent-tran	MAGAWLR	8	10	32	10	1721	186.7	5.96	1740.2	2345.7	1354.9	1814.4	1769.2	1682.6	1620.1	1726.8
V9HWC9	Superoxide	MATKAVCV	51	5	85	5	154	15.9	6.13	6624.5	7573.1	5875.6	7308.5	7867.9	6568.3	6099.7	7051.2

A8K0E7	cDNA FLJ7	MDSGAGG	10	9	48	9	1060	114.6	5.02	1621.8	1477	1200	1499.5	1658.5	1702.6	1531.7	1642.1
Q8N114	Protein shi	MTAPVPAF	15	4	139	4	240	25.6	6.68	7917.8	7596	5190.8	8312.4	8938	8866.3	8826.3	9621.4
A8K5T0	cDNA FLJ7!	MRLLAKIIC	15	15	41	13	1231	138.9	6.71	2427	2126.8	1682.7	1907.5	1816.1	2171.7	2007.4	1759.1
P61970	Nuclear tra	MGDKPIW	51	4	89	4	127	14.5	5.38	4776.5	3725	3291.9	4216.4	5200.8	5235.9	4608.7	4546.1
Q53HF3	Galactosida	MQLRNPEI	29	11	58	11	429	48.7	5.6	2257.9	1522.3	1554.9	1689.8	2162.2	2305	1481.8	1888
A8K7E0	cDNA FLJ7(	MWPLWRI	26	8	54	7	368	41.6	7.52	2175.2	1777.9	1639.2	2115.3	2503.3	2498	1949.8	1780.1
A0A0X9T7T	MS-D3 hea	EVQLVESG	34	4	88	3	134	15	5.92	1066.4	825.6	970.3	970.9	1053.7	912.5	1027.9	988
P26842	CD27 antig	MARPHPW	22	4	102	4	260	29.1	7.64	7241.3	8781.8	5361	8328	7492	8459.2	8591.4	8475.3
Q9UHG2	ProSAAS O	MAGSPLLV	38	7	52	6	260	27.4	6.62	2006.3	1615.3	1372.2	1626.7	1858.4	1994.7	1628.7	1948.4
P39059	Collagen al	MAPRRNN	4	5	94	5	1388	141.6	5	5465.6	4044.1	4524.8	5523.7	5827.2	5977.5	4145.9	5038.6
D3DX01	Amine oxic	MPALGWA	16	11	55	11	751	85.3	7.09	2049.2	2070.4	1802	1655.5	2233.3	1984.6	1995.2	2004.7
S6BGF5	IgG H chair	MDWTWR	26	5	151	1	240	25.6	8.56	1192.1	845	1179.8	1052	1038.8	1036.4	1099.6	1031.6
Q0ZCJ2	Immunglob	LVQLVESG	46	5	89	1	124	13.6	7.93	38.8	37.4	28.2	41.6	35.7	27.7	26.7	24.3
Q59EP2	Angiotensi	VLQQKGM	16	7	65	7	491	53.7	6.42	2605.1	2571.8	2309.7	2338.5	2834.5	2294.5	2443	2105.4
A6XNE2	Compleme	MHSWERL	30	6	33	6	260	27.8	7.25	781.8	804.9	590.3	636.8	704	786.6	728.6	564.6
A0A068LKI	Ig heavy ch	QVQLVESG	33	3	55	1	125	13.9	9.01	671.9	506.6	675.4	667.3	777.7	516.7	714.8	666.2
Q9HD89	Resistin OS	MKALCLLLI	69	5	54	5	108	11.4	6.86	2217.6	1779.3	1616.5	2079	2087.2	2264.9	2240.8	1785.5
P07900	Heat shock	MPEETQTC	19	12	44	6	732	84.6	5.02	1982.8	1471.1	1355.8	1550	1803.6	1925.8	1429.5	1472.5
Q9HCN6	Platelet gly	MSPSPTAL	19	6	115	6	339	36.8	9.2	5401.9	4653	3841	4876.3	5472.5	4774.4	4989.3	5334.1
P05937	Calbindin C	MAESHLQS	34	8	50	8	261	30	4.83	3120.3	2021.4	2552.3	2504.5	2977.9	2806	3014.6	2335.8
Q13332	Receptor-t	MAPTWGP	9	10	25	9	1948	216.9	6.46	915.3	826.1	720.2	816.5	883.4	844	710.9	855.9
P05164	Myelopero	MGVPFFSS	20	13	48	13	745	83.8	8.97	2474.9	1795.9	1263.4	1544.4	1808.1	2952.7	2311.4	1404.2
P08138	Tumor nec	MGAGATG	9	3	96	3	427	45.2	4.7	6089.6	5018.9	4209.4	6183.3	5837.5	5233.7	5392	6441.7
Q5TFQ8	Signal-regu	MPVPASW	36	11	47	1	398	43.3	7.83								
X5D7A8	Cell adhesi	MASVVLPS	12	5	92	5	471	51.5	4.87	4355.4	3410.7	3144.4	4044	4109.3	4185.9	4041.7	4714.4
P00352	Retinal deh	MSSSGTPD	31	13	38	11	501	54.8	6.73	1665.4	1147	1404.6	1296	1586.3	1485.7	1109.5	1152.8
Q16769	Glutaminyl	MAGGRHR	12	4	106	4	361	40.9	6.61	5273.8	4544.2	3676	4501.6	5319.2	5943	4307.9	6127
A0A024R5I	Prolylcarbc	MGRRALLL	14	6	62	6	496	55.8	7.21	2348.5	1816.3	1623.9	2294.1	2412.9	2636.9	2051.7	2154.2
Q8TDQ0	Hepatitis A	MFSHLPFD	17	6	201	6	301	33.4	5.72	11849.5	10111.4	8585.3	12179.6	12614.3	11397.2	12392.8	12319.8
Q9UIB8	SLAM fami	MAQHHLW	19	5	61	5	345	38.8	7.06	2794.8	2510.8	2060	2804	2646.8	2772.8	2812.1	2814.1
A0A087WZ	Ribonuclea	MRPAALRC	32	8	46	8	306	34.9	7.11	1726.6	1731.7	1292.8	1821.6	1698.9	1554	1615.7	1668.3
A0A024RA	Betaine-ho	MAPAGRP(	31	9	47	5	363	40.3	5.87	337.8	237.5	290.9	222.5	268.4	288.7	217.4	221.3
O94910	Adhesion C	MARLAAVL	6	7	43	7	1474	162.6	6.6	1408.8	1034.8	1152.5	1145.8	1207	1139.3	1024.2	1235.7
Q9Y2E5	Epididymis	MGQLCWL	13	11	51	11	1009	113.9	7.24	2511.5	1880.1	1749.3	2266.8	2665.7	2456.1	1958.5	1984.9
Q6ICS1	Putative ur	MAQTNSFI	24	3	114	1	166	18.7	5.95	29.8	68.7	29.7	27.5	40.9	61.3	41.6	30.7
P07737	Profilin-1 C	MAGWNAY	63	7	73	7	140	15	8.27	4046.8	4969.7	2210.9	3424.1	3815.9	3843.3	3985.6	2994
O96009	Napsin-A C	MSPPPLLQ	13	6	71	6	420	45.4	6.61	7511.5	5588.8	4732.2	6183.3	8213.1	8080.5	6626.3	5563.1
O43707	Alpha-actir	MVDYHAA	16	12	31	7	911	104.8	5.44	1027.5	816.9	685.2	836.7	908.5	865.1	836.4	766.8
B3KQF4	cDNA FLJ9(	MAPFEPLA	45	7	40	7	207	23.2	8.1	1424.4	1623.5	1361.2	1355.5	1601	910.3	1127.4	1549.3
E7EX29	14-3-3 pro	MDKNELV(	31	7	53	5	246	28	4.92	3019.6	2253.7	1805.9	2220.8	2654.9	2262.3	2440.6	2159.2
A0A024RA	Carboxype	MVGAMW	24	9	40	9	476	54.1	5.62	2082.3	1494.3	1678.4	1755.8	1866.3	2239.4	1659.7	1637.3

Q03591	Compleme	MWLLVSVI	20	6	36	2	330	37.6	7.39	1255.8	1348.3	1029.9	1501	1546.7	1320.3	1395	1680.8
Q0ZCI8	Immunglob	LVQLVESG(	46	5	91	1	124	13.7	6.61	3687	2573.4	3131.5	3709.9	3624.2	3036.7	4086.2	3187.9
Q86T13	C-type lect	MRPAFALC	15	6	54	6	490	51.6	6.35	3762.7	3412.6	2841.4	3417.4	3589.2	3429	3131.1	3765.5
B2R5M8	Isocitrate c	MSKKISGG	30	12	48	12	414	46.6	7.01	2637.9	2191.9	1916.5	2227.2	2655.5	2079	1758.9	1994.9
F5H265	Polyubiqui	MQIFVKTL	72	7	126	7	149	16.8	6.58	8762.6	9627.2	6689.7	9272.1	9372.1	9688.2	8035.3	9502.6
P12273	Prolactin-ir	MRLLQLLF	36	5	115	5	146	16.6	8.05	6014.5	5684.5	3471.5	12194.5	6161	4771.9	4172.1	5909.5
A8K4K1	cDNA FLJ7	MTGERPST	22	8	43	8	415	45.3	5.88	2706.7	2104.8	2049.9	2339	2351.3	2887.8	2295.4	2325.6
V9HW43	Epididymis	MTERRVPF	31	5	33	5	205	22.8	6.4	1537.5	1307	1189.3	1315.5	1427	1456.7	1291.5	1213.8
A8K5S3	cDNA FLJ7	MLGSGFKA	18	8	75	8	364	39.7	5.35	5732.7	4725.4	4608.2	5445.7	6607.4	5516.1	4576.7	5655.8
P07686	Beta-hexos	MELCGLGL	21	11	62	10	556	63.1	6.76	4263.5	2996	3163.9	3938.5	4365	4680.2	3690.5	3543.4
P23470	Receptor-t	MRRLLEPC	6	7	42	7	1445	161.9	6.42	1654.2	1650.3	1264.4	1576.3	1615.9	1460.7	1574	1622.6
P35527	Keratin, typ	MSCRQFSS	14	8	74	7	623	62	5.24	5294.4	3524	4589.8	4269.9	3950.4	4250.1	3597.8	4522.6
P55291	Cadherin-1	MDAAFLLV	12	7	25	7	814	88.9	4.98	288	234.6	279.5	229.6	254.8	309.3	265.3	325.8
Q92896	Golgi appa	MAACGRV	15	15	34	15	1179	134.5	6.9	2052.1	1838.1	1529.4	1753.1	1923.1	1985.1	1659.2	1840.1
P19013	Keratin, typ	MIARQQC\	20	10	34	7	534	57.3	6.61	1337.8	812.9	978.6	804.4	941.4	1621.4	1858.7	765.5
A0A0X9T7	GCT-A4 ligh	DIVMTQSP	60	4	152	2	109	11.8	8.47	9768.6	12718.8	6822	10547.4	9206.9	11836.9	10789.2	9483.6
Q9GZX9	Twisted ga	MKLHYVA\	24	4	43	4	223	25	5.34	2504.2	2159.2	1627.6	2503.7	2641.7	2572.4	2643.7	2924.7
A0A024RA	Oxidised lo	MTFDDLKI	17	4	35	4	273	30.9	7.28	2452.2	1716.2	1613.3	2061.5	2389.4	1942.1	1780	1957.6
P02452	Collagen al	MFSFVDLR	7	10	33	5	1464	138.9	5.8	3928.9	3402.1	2538	4811.3	3750.4	4245.3	4631.9	3460.5
P35052	Glypican-1	MELRARG\	21	8	22	8	558	61.6	7.3	1244.5	1141.8	1047.5	1204.3	1242.2	1150	1060	1240.3
Q9UBC9	Small proli	MSSYQQK(	71	8	121	8	169	18.1	8.57	5390.1	12762	9129	10573.1	8360.2	6416	11899.2	9233.8
A0N5G1	Rheumatoi	DIQMTQSF	32	3	56	3	116	12.5	8.46	2360.1	1989.1	1960.5	1873.6	2111.2	2171	2250.6	2204.4
O43490	Prominin-1	MALVLGSL	17	11	32	11	865	97.1	7.27	1222.6	997	966.4	1167.5	1296	1105.4	952.1	1212.4
A2NZ55	Variable im	QVQLQESG	31	3	66	1	131	13.9	8.79	147.8	123.4	193.1	127.9	134.6	110.3	102.6	102.9
Q8IWU5	Extracellul	MGPPSLVL	3	2	151	2	870	100.4	9.17	10334.4	8417.1	7503.2	10138.2	10534.8	12935.1	9686.6	10985.6
P02533	Keratin, typ	MTTCSRQF	18	10	79	1	472	51.5	5.16								
K4DIA0	ICOS ligand	MRLGSPGL	8	3	88	3	473	51.9	8.05	5079.2	4272.8	3998.4	4664.2	4927	5008.9	4414	5031.9
G9K388	YWHAE/FA	RADPAGAA	16	6	54	4	384	41.2	4.97	679.3	470.5	511.8	544.3	687.1	594.4	534.4	553.6
P01036	Cystatin-S (	MARPLCTL	55	6	26	3	141	16.2	5.02	1438.1	1542.3	1180.3	1476.5	1583.9	1129.7	1276.4	1649.6
A0A140VK	Testicular s	MGPPGSP\	16	6	32	6	440	49.5	6.11	1582.4	931.6	1596.7	1267.1	1527.6	1622.4	1341.4	1333
P61626	Lysozyme (	MKALIVLG	45	5	49	5	148	16.5	9.16	3032.5	2727.6	1886.7	2625.8	2860.8	2982.3	2609.5	2357.3
E7EMB3	Calmodulir	MILAHCNL	21	5	45	4	196	21.7	4.56	2441.8	1992	1697.6	2329.9	2483.3	2298	2206.6	2471
G5EA09	Syndecan b	MIKLWTNF	22	4	41	4	318	34.8	8.51	883.9	839.2	687.6	935.9	1056.2	818.4	776.2	873.8
B0YJ88	Radixin OS	MPKPINVR	16	11	55	3	583	68.5	6.37	311.9	233.5	281.2	249.1	280.8	295.1	224.3	241.6
Q5SZK8	FRAS1-rela	MHSAGTP(	4	10	26	10	3169	350.9	5.03	792.6	737.9	594.6	761.9	773.3	804.4	675	719
B7ZLI0	Reticulon 4	MLPGLRRL	20	8	53	8	420	46.1	7.62	2245.9	1658.8	1591.9	1972.1	2026.8	2360.3	1624.4	2004.2
P36980	Compleme	MWLLVSVI	26	6	33	3	270	30.6	6.38	134.1	423.3	103.5	108.3	139.9	112.3	117.5	120.1
P08779	Keratin, typ	MTTCSRQF	18	10	76	2	473	51.2	5.05	654	462.9	554.2	480	472.5	584	484	560.6
A0A0B4J1X	Immunoglo	MEFGLSW\	25	3	59	1	117	12.8	8.66	728.4	496.3	518.8	706.7	598.3	825	743.9	655
Q9NZZ3	Charged m	MNRLFGKA	32	6	39	6	219	24.6	4.83	2103.7	1732.4	1767.8	1974.2	2283.4	2130.3	1763.5	2219.9
P54760	Ephrin type	MELRVLLC	13	9	41	9	987	108.2	6.9	4150.1	3597.8	3661.3	3917.5	4111.6	3935.4	3866.3	3845.3

B5BU24	14-3-3 pro	MTMDKSE	33	7	47	4	246	28.1	4.83	540.2	366.4	401.3	437.2	510.3	484	437.3	417.7
P00918	Carbonic a	MSHHWGY	33	7	35	7	260	29.2	7.4	3153.7	2148.2	2165.8	2471.6	3115.3	3077.6	2633.3	2325.9
Q9NXI0	cDNA FLJ2	MAIHKALV	39	6	71	6	162	17.8	7.43	4281.8	4055.2	3679.7	4597	5375.9	4133.3	3868	4622.8
B4DRC8	cDNA FLJ5	MARFWVC	21	6	38	6	347	38.2	6.43	1742.5	1525.8	1295	1580.4	1695.9	1801.9	1645.9	1667.8
P13727	Bone marr	MKLPLLLAI	29	6	44	6	222	25.2	6.76	1267.2	1601.6	1054.8	1330.5	1262.3	1452.9	979.8	1435.9
A0A024R6	G protein-	MTPQSLLC	11	6	56	6	693	77.7	8.48	1542.9	1553.7	1174.7	1514.9	1606.5	1744.4	1340.7	1492.5
Q1HP67	Lipoprotei	MEHKEVVL	15	4	44	4	2040	226.4	6.07	2174.3	2279.4	2206.5	2303.6	2567.9	2156.5	2616.5	2730.5
P08727	Keratin, ty	MTSYSYRQ	24	11	76	3	400	44.1	5.14	2142	1364.5	1990.4	1636.9	1657.7	1925.6	2072.4	1779.3
Q9UL86	Myosin-rea	EIVLTQSPG	45	3	39	2	109	11.9	7.96	1789.8	1610.9	1129.5	1322.2	1775.7	1837.6	2061.6	1607.2
P08572	Collagen al	MGRDQRA	4	7	29	7	1712	167.4	8.66	2006.7	1716.7	1584.4	1839.5	1975.3	1847.8	1623.4	1789.4
P01037	Cystatin-SN	MAQYLSTL	44	4	23	2	141	16.4	7.21	24.8	28.8	27.4	21.9	22.3	19.6	19.4	21.5
B7ZL91	Meprin A s	MAWIRSTC	5	2	80	2	774	87.8	5.78	2291.6	2357.6	1893.1	1972	2081.8	2006.6	1956.4	2072.7
Q562M3	Actin-like p	KIWHHTFY	28	2	76	1	103	11.5	6.68	74.8	64.7	55.9	55.6	75.9	66.1	64.7	55.1
P08697	Alpha-2-an	MALLWGLL	16	6	36	6	491	54.5	6.29	2105.7	1715.7	1461.5	1904.1	2323.6	2062.6	1827.3	1902.8
O43653	Prostate st	MKAVLLAL	22	2	164	2	123	12.9	5.29	12636	8903	7470.4	12814.1	19102.5	9102	7892.3	14644.6
B1N7B8	Cryocrysta	EIVLTQSPA	28	2	129	1	107	11.8	7.99	317.5	330.5	195.3	310.9	269.9	372.9	295.6	287.4
J3KPS3	Fructose-b	MPYQYPAL	31	10	68	9	368	39.8	8.09	2061.1	1731.8	1659.4	1871	1945.5	1787	1970.7	1637.1
E9PKP4	Macrophag	MIGSGHLC	56	5	40	1	113	13.2	5.25	161	130.3	93.3	156	122.2	153.8	125.3	117
G3V3D1	Epididymal	MRFLAATF	23	6	53	6	221	23.7	6.73	5071.2	4544.1	4043.5	5687.7	5665	5183.7	4749.9	5185.3
M9MML6	Low affinit	MGGGTGE	21	7	64	3	269	29.9	6.92	5733	4810.8	4415.7	5931	5551.6	4437.6	5472.1	5907.2
K7EKE8	Nectin-2 (F	XKPKNQAE	45	8	40	1	210	22.7	4.91	70.9	60.7	61.4	64.3	70.6	67.9	56.4	69.3
P05787	Keratin, ty	MSIRVTQK	18	10	40	6	483	53.7	5.59	585.3	414.8	335.9	397.8	473.5	529	784.1	378.4
V9HW77	Epididymis	MPFSNSHN	29	7	25	7	381	42.6	5.59	884.9	860	777.8	764.6	831.3	647.8	591.7	765.9
Q96KP4	Cytosolic n	MAALTTLF	24	8	29	8	475	52.8	5.97	933.4	723	968.5	723.4	839.6	784.8	687.6	620
A0A140VJI	Dipeptidas	MWSGWW	17	6	43	6	411	45.6	6.15	2516.8	1651.9	1699.5	1992.2	2163.6	2087.6	1612.2	1991.3
Q9HCC1	Single chai	EVQLVESG	37	3	48	1	112	12.2	8.47	55.8	45.9	53.9	44.4	50.4	44	47.9	59.9
P01624	Immunoglo	MEAPAQLL	26	2	132	0	115	12.5	5.19	321.4	341.2	274.6	335.5	342.8	380.3	421.6	394.7
P08238	Heat shock	MPEEVHH	12	8	27	1	724	83.2	5.03	223.9	161.2	144.4	165.2	201.9	220.2	164.8	175.5
A8K486	Peptidyl-pr	MVNPTVFF	53	8	66	8	165	18	6.9	3466	3071.8	2421.9	3017.3	3213.8	2980.3	2738	2486.2
A8K3I0	cDNA FLJ7	MVPDTAC	17	8	28	7	757	82.8	4.59	1635.7	1251.2	1235.1	1517.7	1531.9	1335.8	1377.3	1548.7
H3BUX1	Mesothelir	VEKTACPS	17	6	50	1	398	43.8	6.37	249.1	163.7	158.5	163.8	195.9	179.5	159.4	170.1
A5PLM9	Cathepsin	MNPTLILA	20	6	49	6	333	37.5	5.45	2778.1	2427.9	1997.4	2643	2692.1	2451.3	2328	2498
A0A140VK	Testis tissu	MQALVLLL	20	7	33	7	418	46.3	6.38	712.7	614.6	655.5	545.8	594.5	648.9	610.7	486.1
A0A0C4DG	Poliovirus	MARAMAA	12	6	63	6	417	45.3	6.52	3861.3	3487.2	3365.3	4192.5	4276.3	4492.2	3807.5	4425
A0A0G2JN	Ephrin type	MATEGAA	9	6	26	6	1022	110.7	6.65	1132.9	1158.7	848.8	1042.2	1122.4	1062.7	929	1083.3
Q92692	Nectin-2 O	MARAAALL	16	8	44	1	538	57.7	4.82	2270.3	1939.5	1743.8	2226	2396.9	2911.6	2197.8	2261.7
H9ZYJ2	Thioredoxi	MVKQIESK	40	5	86	5	105	11.7	4.92	5920.6	4943.4	3751.3	4701.9	5871.3	4765.7	5276.5	5069.4
V9HW87	Abhydrolas	MAASVEQI	33	5	33	5	210	22.3	6.4	1439.8	1138.1	739.7	917.8	1029.2	1228.9	1033.4	868.9
A0A0S2Z4F	Secretoglo	MKLAVTLT	20	3	114	3	91	10	5.06	5373.7	10779.9	5773	7533.6	32076.5	6110.8	10733.6	13735
C9JC84	Fibrinogen	MSWSLHPI	28	9	37	9	461	52.3	5.63	1762.4	1127.8	921.8	1226.6	1006.6	1831.3	1206.5	884.6
P09228	Cystatin-SA	MAWPLCT	48	5	22	3	141	16.4	4.93	278.3	284.7	226.7	292.8	253.7	251.4	237.9	244.6

A8K4G7	cDNA FLJ78	MSSTSPNL	18	8	78	5	444	49.2	6.76	638	489.7	518.1	602.5	679.2	581.9	499.3	601.9
C9JKV3	Tissue facto	MIYTMKKV	23	3	36	3	215	24.6	5.19	1181.8	852.8	901.8	942.7	1054.1	869.6	927	1101.7
S6BGD4	IgG H chain	MKHLWFFI	17	3	127	1	234	25	9.48	392.9	315.7	333.3	324.3	320.8	328.6	425.2	351.9
V9HWE0	Annexin OS	MAQVLRGT	23	8	39	7	320	35.9	5.05	1873.1	1360.8	1963.9	1753.5	1865.3	2098.8	1405.2	1625.8
Q14982	Opioid-bin	MGVCGYLF	14	4	49	3	345	38	6.87	1496.7	1676.5	1148.8	1532.7	1632.2	1519.2	1356	1754.3
P13647	Keratin, typ	MSRQSSVS	18	11	34	4	590	62.3	7.74	21.6	9.1	7.1	8.4	16.2	22.9	22.9	11
B4E0X1	Beta-2-mic	MSRSVALA	27	4	110	4	122	13.9	7.44	8072.4	27383.9	6068.4	8892.8	12376.4	9033.9	8006.1	8163.5
Q05707	Collagen al	MKIFQRKM	5	7	20	7	1796	193.4	5.3	783.6	614.6	602.1	688	701.7	696.8	716.8	720.9
Q76LA1	CSTB prote	MMCGAPS	46	3	39	3	98	11.1	7.56	1792.2	2064.4	1233	1965.6	1713.9	1753	1810	1626.1
A8K6A7	Alpha-man	MGAYARAS	7	6	18	6	1011	113.7	7.28	1033.4	998	803.8	1030.8	1014.1	1059.9	973.7	844.5
Q8N2S1	Latent-tran	MPRPGTSC	5	6	27	6	1624	173.3	5.43	768	688	519.9	703.5	698.4	661.8	604	739
V9HVY1	Epididymis	MKRMVSW	25	11	29	11	491	55.9	8.27	2437.8	1557.3	1260.8	1609.1	1335.7	2788.9	1464.8	1138.4
P22352	Glutathion	MARLLQAS	31	6	71	6	226	25.5	8.13	2563.6	1964.2	1772.7	2023.5	2386.6	2515.8	2003.7	2075.2
P11717	Cation-inde	MGAAAGR	6	13	28	13	2491	274.2	5.94	1217.3	1113.5	1042.7	1078	1216.5	1059.4	956.8	1096.8
P39060	Collagen al	MAPYPCGC	5	9	38	9	1754	178.1	6.01	1910.3	1467.7	1426.3	1755.9	1902.5	2016	1518	1567.8
P27348	14-3-3 prot	MEKTELIQI	16	4	42	1	245	27.7	4.78	52.7	37.6	34.9	39	41.4	41.7	39	37.8
B1B5Q3	N-acylsphir	YRGAVPWY	28	7	64	1	219	24.7	7.42	1559	1181	1166.2	1323.7	1705.7	1682.7	1500.7	1108.7
A0A109PSY	MS-A1 ligh	DIQMTQSP	32	2	35	1	106	11.4	8.48	455	431.8	370.7	444.7	431.1	411.4	428.1	411.4
A0A024R39	Chromosor	MACAEFSF	27	7	31	7	315	35.1	6.7	1945.7	1614.2	1283.9	1666.3	1650	2075.1	1584.4	1446.6
Q13421	Mesothelir	MALPTARP	11	6	47	1	630	68.9	6.38	1033	1805.3	751	1180.2	1765.3	1419.5	1448	1462.1
A8K6D3	cDNA FLJ75	MRLHLLLLI	12	9	30	9	879	100.9	5.52	1358.1	1103.2	988	1166.2	1415.7	1275.2	1133.8	1294
Q8NDA2	Hemicentir	MPGAPLLR	2	6	27	6	5059	541.6	5.87	741.1	706.9	610.5	663.3	641.7	672	596.5	679.7
A0A024R1(	N-acetylga	MLLKTVLLI	15	5	49	5	411	46.5	5.19	2261.6	1739.8	1422.2	1984.7	2477.3	2374.5	1700.8	1806.1
A8K6Q6	cDNA FLJ78	MLRRRGSP	14	3	13	3	534	57.3	4.91	775.7	685.6	553	734.5	755.4	737.5	753.9	837.3
P23284	Peptidyl-pr	MLRLSERN	34	7	32	7	216	23.7	9.41	1717.7	1360.3	1310	1516.1	1553.2	1487.8	1248.4	1480
Q14126	Desmoglei	MARSPGRA	9	8	32	8	1118	122.2	5.24	794.2	800	692.7	683.2	726.5	770.9	578.8	704.3
Q59ED3	Intercellula	LIVTCSLQC	11	4	50	4	344	38.4	7.78	1898.1	1659.3	1243.9	1789.1	1746.1	2055.2	2153.6	1945.1
B7Z831	cDNA FLJ55	MNPPWSV	10	4	46	4	429	47.7	8.53	2676.3	2457.3	2803.3	2612.9	2971.4	2351.5	2022.2	2556.4
P01700	Immunoglo	MAGFPLLL	25	2	68	1	117	12.3	5.91	1520.9	1749.6	1154.2	1661.1	1558.9	1592.1	1656.5	1537.5
P29508	Serpin B3 (	MNSLSEAN	23	8	23	8	390	44.5	6.81	561.3	590.2	447.5	790.4	582.3	466.5	684.4	519.3
P98172	Ephrin-B1 (	MARPGQR	16	6	65	6	346	38	8.94	3050.7	3759	2013.2	3383.4	3661.4	3374.9	3188.6	3677.8
E7EX88	Aggrecan c	MTTLLWVI	3	8	30	8	2530	261.2	4.13	2024.7	1575.7	1306.7	1555.7	1556.8	1473.4	1417.3	1450.7
A2JA16	Anti-mucin	DIQMTQSP	32	2	41	1	107	11.6	8.46	702.5	405.7	430.9	485.7	382.2	438	467.6	377.5
Q96J84	Kin of IRRE	MLSLLVWI	9	5	38	5	757	83.5	5.73	1318.9	840.3	986.8	1015.7	1048.9	1137.5	990.5	1097.2
P68104	Elongation	MGKEKTHI	15	7	28	7	462	50.1	9.01	1705.1	1612.9	1168.8	1489.6	1749.6	1777.2	1548.6	1493.6
A0A087WV	Osteoclast-	MALVLILQI	16	3	74	3	286	30.8	6.52	3216.3	3158.9	2454.7	3172.2	2889.2	3037.9	2923.9	2857.4
A8KAM8	Platelet-de	MRLPGAM	7	6	16	2	1106	123.8	4.98	488.6	387.3	391.2	417.2	473.1	453.9	423.8	442.6
O43866	CD5 antige	MALLFSLIL	14	4	41	4	347	38.1	5.47	2915.9	2302.2	2112.3	2813.1	2794.9	2669.5	2457.7	2812.2
Q12841	Follistatin-	MWKRWLA	31	9	25	9	308	35	5.52	2137.1	2142	1770.6	1852	2137.1	1484.7	1429.3	2041.7
P07204	Thrombom	MLGVLVLG	13	4	20	4	575	60.3	4.92	448.8	342.6	303.9	350.7	372.6	385.9	329.5	383.3
I6QTG3	Glypican 3	MAGTVRTA	12	6	37	6	580	65.5	6.37	2226.9	1935.4	1992	2312	2217.1	1851	1851.5	2113.6

P00966	Argininosu	MSSKGSVV	21	10	40	10	412	46.5	8.02	2130.2	1520.8	1479.7	1635.5	2091.1	1963.9	1334.6	1519.8
Q13621	Solute carr	MSLNNSSN	7	8	22	8	1099	121.4	7.39	1528.9	1645.3	1148	1545.9	1657.9	1432.5	1143	1547.5
P55285	Cadherin-6	MRTYRYFL	9	6	19	6	790	88.3	4.93	916.1	913	740.3	936.4	994.5	1120.4	965	1088.4
B0AZL7	cDNA, FLJ7	MALRKGGI	17	9	22	9	605	66	6.79	840.1	715.5	642.2	684.2	780.4	800.9	638.8	753.7
A0A075B7I	Protein IGI	MEFELSWV	19	2	51	1	117	12.9	6.51	1959.5	1475.5	1827.5	1905.3	1850.1	1647.3	1845.8	1917.7
A0A024R3I	Sortilin-rela	MATRSSRR	5	10	26	10	2214	248.3	5.53	993.6	1061.1	784.7	1063.6	1101.1	1075	876.1	1095.4
P31151	Protein S1(	MSNTQAEI	34	3	35	3	101	11.5	6.77	956.5	741.2	659.2	1743.2	2212	1347.3	1983.1	1722.6
M1VKI3	Tyrosine-p	MAPARLFA	7	4	46	4	746	83.1	4.97	2391.1	1882.6	1857.1	2297.2	2672.2	2363.3	2185.3	2867
A0A024R0	Compleme	MSACWSF/	16	8	49	8	559	63.2	5.52	1434.3	1387.5	1429	1288.4	1302.7	1542.2	1810.6	1203
Q5KU26	Collectin-1	MKDDFAEE	7	6	82	6	742	81.5	5.69	5931.4	6072	3325.5	4204.7	4897.4	6084	4796.8	6136.8
Q9UGN4	CMRF35-lik	MWLPWAI	10	2	82	2	299	33.2	5.49	5299.1	5026.6	3620.8	5487.6	5906.4	5248	5819.6	6215.1
B7Z1Z5	cDNA FLJ57	MKTIQPKM	20	5	32	4	355	39.2	8.05	1432.8	1276.1	1208.8	1321.2	1382.2	1366.6	1347.1	1546.6
Q9H8L6	Multimerir	MILSLLFSL(	7	5	51	5	949	104.3	5.86	2527.5	1925.8	1892.7	2305.8	2331.8	2521.4	2189.7	2241.8
A8KAQ3	cDNA FLJ7(	MERPWGA	9	6	18	6	831	92	5.74	660.4	587.1	570.5	630.7	648.9	634.6	557.9	574.4
P40925	Malate deh	MSEPIRVL\	18	5	29	5	334	36.4	7.36	2093.6	1746.1	1809.7	1889.8	2335.3	1978.7	1738.8	1746.5
D3DTX7	Collagen, t	MGFPGPK(	9	6	20	1	885	84.7	6.24								
A0A109PS5	GCT-A8 ligl	DIVMTQSP	33	2	93	1	112	12.5	8.5	204.4	205.8	130.5	173.9	187	207.6	189.8	213.7
P56537	Eukaryotic	MAVRASFE	23	4	21	4	245	26.6	4.68	954.1	753.4	767.9	848.3	864	941.5	893.8	805.9
B2R5M3	cDNA, FLJ9	MQPTLLLS	6	3	29	3	677	78.2	5.1	2417.7	1964.8	2140.9	2074.8	2335	2773.4	2251.9	2743.8
B7Z1F8	cDNA FLJ53	MCPEDSLC	34	8	24	1	270	30.4	7.99								
P08123	Collagen al	MLSFVDTR	7	6	12	6	1366	129.2	8.95	706.4	689.9	526.5	683.7	683.1	830.1	687.6	599.1
P11047	Laminin su	MRGSHRA/	4	6	25	6	1609	177.5	5.12	780.4	625.4	808.8	698.5	754	736.5	647.9	796.9
A0A024RC.	Discoidin d	MGPEALSS	12	9	22	9	919	101.7	6.83	890.6	650	705.5	781.8	851	882.7	712.9	805.3
P14780	Matrix met	MSLWQPL\	15	9	25	9	707	78.4	6.06	2452.9	1530.3	1080.4	1809.8	1992.7	2260.4	2177.8	1798
Q8NCC3	Group XV p	MGLHLRPY	21	7	41	7	412	46.6	6.73	1659.2	1849.4	1093.8	1593.5	1799.2	1720.4	1399.9	1266.8
B3KNB4	cDNA FLJ14	MVDREQL\	16	4	42	1	247	28.2	4.89	141.7	103	143.4	120	108.6	137.7	103.4	102.1
A0A087WT	Transthyre	MASHRLLL	26	4	33	4	185	20.1	5.33	1516.3	1328	1449.7	1274.6	1364.1	1482	1330	1353.3
P19827	Inter-alpha	MDGAMGI	9	6	17	6	911	101.3	6.79	613.5	471.8	573.4	494.3	480.6	616.8	506.3	440.1
Q9UN37	Vacuolar p	MTTSTLQK	23	9	78	6	437	48.9	7.8	2674	2306.8	1958.7	2677.2	3099.7	2625.1	2040	2652.8
A0A0S2Z45	Serpin pep	MAFLGLFS	15	6	36	6	410	46.4	4.91	1521.9	1022.6	1249.1	1268.9	1514.8	1478.5	1156.2	1294.4
P31997	Carcinoem	MGPISAPS(	12	3	101	2	349	38.1	7.39	3511.7	3216.7	2157.7	3216.6	3211.6	2946.1	3237.4	2990.3
Q96JQ0	Protocadh	MQKELGIV	1	3	20	3	3298	346	4.94	699.5	615.2	638.2	665.4	699.8	691.7	572.8	663.9
P18510	Interleukin	MEICRGLR	24	4	11	4	177	20	6.19	246.8	266.9	212.5	317.9	259.6	259.3	311.8	254.6
B3KUI5	Hyaluronid	MAAHLLPI(	18	5	27	5	435	48.3	6.96	879.3	465.3	570.1	764.9	705.2	691.5	570.8	584
Q15375	Ephrin type	MVFQTRYI	8	6	26	5	998	112	5.8	1043.8	781.1	651.9	832.8	827.2	753.2	663	865.6
O15230	Laminin su	MAKRLCA(	2	7	23	7	3695	399.5	7.02	1277.4	1384.1	1257	1455.4	1219.5	1256.7	1030.6	1128.4
Q9ULI3	Protein HE	MASPRASR	7	7	34	7	1381	147.4	6.18	3599.4	3451.9	2855.2	3841.9	3894.5	3781.3	3725.1	4254.6
A6NI73	Leukocyte	MAPWSHP	17	4	19	4	299	32.7	6.99	1419.2	1255.4	1066.2	1303.5	1416.8	1362.8	1342.9	1420.6
Q9Y279	V-set and i	MGILLGLLL	13	3	32	3	399	44	6.35	1502.7	1657.1	914.9	1971.7	1805.8	1450.6	1453.1	1900.6
S6BAQ4	IgG H chair	MEFGLSW\	16	3	127	1	236	25.3	8.44								
B2R4R0	Histone H4	MSGRGKG(	50	5	58	5	103	11.4	11.36	3267.7	2510.2	1327.3	1672.2	1782.4	3202.8	3831.6	1469.8

Q15485	Ficolin-2 O	MELDRAV	16	6	22	6	313	34	6.77	2249.4	1752.2	1371.5	1262.5	1880.5	2102.4	2340.7	2120.4
P00338	L-lactate d	MATLKDQL	27	8	29	7	332	36.7	8.27	828.7	888	543.8	861.6	945.2	993.3	924.8	637.4
Q96S96	Phosphatid	MGWTMR	25	5	56	5	227	25.7	6.54	5705.1	5915.8	2936.1	3959.4	4666.1	4031.1	3204.2	4021.4
K7EPJ4	Cartilage in	MASLLPLL	7	7	30	7	1162	126.8	8.27	1309.1	1192.2	944.6	1219.8	1235.8	1188.1	1081.7	1220.8
Q7KYR7	Butyrophili	MESAAALH	8	6	68	6	527	59.6	6.48	5880.3	4902.5	4426.9	5645.1	5865.5	5946.4	5279.6	6869.3
A0A087WX	Mucosal ad	MDFGLALL	11	3	42	3	406	42.4	5.07	1635	1422.1	1311.7	1740	1698.8	2030.6	1689.1	1956.5
A0A024RD	Lymphocyt	MARGSVSD	14	8	34	5	627	70.2	5.43	1083.2	784	516.2	823.9	836.9	886.5	1142.3	623.7
A0A0A0N0	CNTFR isof	MAAPVPW	11	4	42	4	380	41.5	6.86	3060.4	2938.5	2418.7	2809.6	2998.2	2725.8	2676.7	3175.4
B2R8I2	cDNA, FLJ9	MKALIAALL	12	7	24	7	525	59.5	7.44	1246	1141.8	840.1	1117.5	1001.6	1314.3	1197	932.5
P29323	Ephrin type	MALRRLGA	7	5	15	5	1055	117.4	6.55	229.4	239.8	184.5	236.1	243.8	239.6	195.7	252
P68363	Tubulin alp	MRECISIHV	16	5	12	1	451	50.1	5.06	20.9	19	11.8	18.9	18.5	19.7	16.4	15.9
A8K6K4	cDNA FLJ7	MTLLWCV	11	7	27	7	570	65.4	8.22	1483.1	1504.4	1208.4	1401.7	1489	1408.5	1384.1	1392.7
P51688	N-sulphogl	MSCPVPA	11	5	30	5	502	56.7	6.95	917.1	561.5	525.9	676.5	662.6	827.3	624.4	582.5
B7Z1K5	Tubulin alp	MTAWFNG	13	5	13	1	519	57.7	5.07	465.8	406.6	342.3	353.2	397.9	385.2	328.3	325.4
S6BAP4	IgG H chair	MDWTWR	22	4	123	1	275	29.4	8.51	26.2	36.7	23.2	53.7	20.7	13.6	19.7	14.6
B4DPQ0	Compleme	MSVRFRV	10	6	19	4	719	81.8	6.37	283.8	233.2	191.1	247.3	237.5	244.2	251.2	239.7
B7ZKQ8	PODXL pro	MRCALALS	9	5	43	5	560	58.8	5.49	3202.6	2843.3	2466.7	3141.1	3425.7	3439.7	2693.7	3403.1
O75487	Glypican-4	MARFGLPA	8	4	17	4	556	62.4	6.68	606.7	544	536	593.3	617.9	615.2	507.1	581.4
B2R701	cDNA, FLJ9	MVELHNLY	16	6	45	1	428	45.7	5.38	25.1	30	20.1	25.4	24.7	23.6	23.4	30.3
P52758	Ribonuclea	MSSLIRRVI	27	3	22	3	137	14.5	8.68	669.4	526.4	508	569.9	663.4	621.3	498.3	539.6
G3XAK1	Hepatocyt	MGLWWV	14	8	29	8	725	81.9	7.8	1531.2	1406.8	1271	1426.6	1515.2	1369.1	1451.5	1427.9
V9HWC7	Epididymis	MPGGLLLG	27	6	41	6	224	25	6.38	1758.3	1425.6	1328.4	1458.9	1803.2	1594.8	1296.7	1373.1
Q9H665	IGF-like far	MGPGRCLL	11	3	51	3	355	37.9	7.08	1816.2	1642.3	1422.6	1945.8	1979.3	1855.6	1805.5	1955.6
A0A125U0	MS-F1 hea	EVQLVESG	17	2	32	1	126	13.5	8.16								
B8ZWD9	Diazepam	MGWTSLC	31	3	46	3	144	16.1	5.05	1883	1852.7	1540.4	1782.7	1839.2	1173.8	1657.3	1884.1
P25940	Collagen al	MGNRRDL	2	2	9	2	1745	172	6.87	269	230.1	314.2	250.9	252.9	229.1	235.6	258.1
O75942	Major prio	MANLGCW	10	3	67	3	285	30.7	9	3095	2786.1	2538	2956.3	3476.6	2890.9	2604.5	3584
P0DJD8	Pepsin A-3	MKWLLLLG	9	4	250	4	388	42	4.41	19671	18981.8	15407.6	26908.4	24011.9	34342.5	19145.7	22700.4
Q07954	Prolow-der	MLTPPLLLL	1	7	31	7	4544	504.3	5.39	2617.6	2206.5	1916.2	2396.9	2485.3	2467.2	2086.3	2483.3
O14745	Na(+)/H(+)	MSADAAA	19	6	32	6	358	38.8	5.77	1769.7	1223.1	1195.9	1283	1539.7	1621.2	1203	1481.2
A0A0A0MF	Immunoglo	MEAPAQLL	32	3	90	2	115	12.6	5.29	111.8	105.3	81.6	106.7	108.3	110.6	98.8	103.8
A2NXP8	Heavy chai	QVQLVQSG	18	2	31	1	123	13.8	9.72	93.6	87.4	107.2	87.3	100.2	88.4	97.5	85.1
P05546	Heparin co	MKHSLNAL	9	4	34	4	499	57	6.9	964.9	883.6	856	944.4	953.3	1098.7	921.4	886.9
P34896	Serine hyd	MTMPVNG	19	7	24	7	483	53	7.71	549.9	402.2	369.5	396.3	505.3	520.1	398.3	382.3
A0A024R0	Guanine nu	MSELDQLR	20	6	25	2	340	37.4	6	84.4	96.5	64.2	84.8	85.2	79.6	69.6	80.9
A0A087WV	CD177 anti	MSAVLLLA	15	6	27	6	437	46.4	6.01	2384.2	2654.3	2125.4	2433.4	2768.1	2120.2	1900.9	2340.8
P30044	Peroxiredo	MGLAGVC	36	6	33	6	214	22.1	8.7	982.8	936.2	647.4	893.8	979	982.2	894.2	908.4
P05362	Intercellula	MAPSSPRP	11	5	34	5	532	57.8	7.99	1658.4	1661.3	1164.3	1576.1	1659.3	1764.4	1716.8	1699.8
B2R7S8	cDNA, FLJ9	MVPAWLW	11	7	15	7	829	89.9	4.96	448.1	353.8	398.5	382	440.4	426.9	424.1	426.3
A8K5I6	cDNA FLJ7	MPQLLQN	11	5	24	5	495	53.5	6.1	2198.4	1626.9	1167.8	1735.9	1692.7	1782.4	2625.8	1669.6
P48061	Stromal ce	MNAKVVV	40	4	55	4	93	10.7	9.88	2991.8	3366.1	2422.9	3085.8	3437.8	3411.9	3247.7	2541.3

Q6V0I7	Protocadhe	MDLAPDRA	2	10	19	10	4981	542.4	4.94	736.9	728.6	580.1	732.5	748.9	753.1	594	719.2
Q5U000	Cathepsin Z	MARRGPG	18	5	37	5	303	33.8	7.11	3462.5	4858.2	2648.4	3428.6	3545.8	3525.8	3138.2	3261.1
Q9BRK5	45 kDa calc	MVWPWV	17	6	15	6	362	41.8	4.86	1225.7	966.4	980.6	1020.7	1077.1	1197.8	955.2	1047.6
P55000	Secreted Ly	MASRWAV	61	3	21	3	103	11.2	5.33	419.5	470.6	283.5	441.1	531.6	468.5	399.8	663.4
P54753	Ephrin type	MARARPPP	8	7	13	7	998	110.3	6.32	336	295.4	292.5	336.9	354.3	347.4	331.4	352.1
H3BLU2	Limbic syst	LRLLCLLPT	21	6	17	6	345	38	6.86	599.9	604.2	601.9	570.2	628.9	554.6	551.7	686.6
Q59FR8	Galectin (F	ECFLSRNC	22	5	16	5	258	27.1	8.41	871.6	777.7	636.1	816.7	937.7	857.6	767.2	800.5
P16152	Carbonyl re	MSSGIHVA	18	4	20	4	277	30.4	8.32	462.6	440.1	335.6	405.9	465.1	431	341.6	385.5
Q9UBX7	Kallikrein-1	MQRLRWL	14	4	43	4	282	31	8.94	2886.9	3948.2	2651.8	2340.6	3219.7	1809.4	1938.5	2722.4
A0A0C4DH	Immunoglo	METPAQLL	22	2	60	1	116	12.5	4.59	2440.4	3158.5	1930.8	2450.8	2371.9	2725.7	3150.4	2826.7
A0A024R64	Ceroid-lipo	MRRNLRLG	15	6	38	6	407	46.3	8.38	1759.1	1227	1184.9	1466.2	1856.7	1724.2	1388.2	1477.6
P09237	Matrilysin	MRLTVLCA	26	7	22	7	267	29.7	7.91	1379.4	1155.3	1118.1	1249.5	1364.3	1583.5	1306.9	1214.8
A0A1A7UP	Galactosan	MAAVVAA	11	6	26	6	522	57.9	6.83	1416.8	735.3	965.6	1109.4	970.4	1418.9	1134.1	997.6
Q16849	Receptor-t	MRRPRRP	4	3	23	3	979	105.8	7.11	1406.2	1093	1081.4	1228	1230.5	1692.9	1051.5	1455.7
A0A125U0	MS-C1 hea	EVQLVESG	25	3	31	2	118	13.1	6.07	952	793.7	912.6	934.2	1014.9	876.2	991.6	1014
Q9HB40	Retinoid-in	MELALRRS	6	3	41	3	452	50.8	5.81	1368.6	929.5	855	1095.1	1193.1	1368.5	883.1	904.6
A0A024R0	Phospholip	MKPKLMY	12	6	43	6	490	54.7	6.47	2048.1	1356.2	1394.3	1730.1	1788.3	2098.2	1540	1692.7
A0A0A0MT	Glucose-6-	TSGQRPAK	12	6	17	6	573	64.8	9.04	1311.3	995.2	873.4	1108.3	1193.8	1075.8	975.4	945.3
Q02747	Guanylin O	MNAFLLSA	18	3	55	3	115	12.4	4.59	2596.8	6099.4	2609	2855.8	3234.9	2920.1	2336.8	2797.1
A0A024R5	Tumor nec	MKPSLLCR	11	3	13	3	430	46.1	8.35	818.8	644.6	636.5	707.2	718.3	843.3	755	829.6
A0A024R5	UDP-GlcNA	MQMSYAI	15	5	25	5	415	47.1	7.2	921.3	710	707.1	823.4	950.1	955.7	763.9	751
P09564	T-cell antig	MAGPPRLL	10	2	36	2	240	25.4	7.27	233.9	178.6	345.4	206	250.6	226.9	245.6	271.3
E9PK25	Cofilin-1 O	MVSPGHG	25	5	47	5	204	22.7	8.34	3775.1	2670.2	2332.4	3004.5	3519.9	3036.5	3144.5	2864.2
A0A087WV	Receptor-t	MKPAAREA	4	4	21	4	1342	146.5	5.62	767.1	538.4	734.8	649	717.5	641.8	594	682.1
A4GW21	Programm	MIFLLLMLS	13	3	37	3	273	30.8	7.78	1167.3	955.3	862	974.5	1115.6	1052.4	1021.5	1066.1
O75339	Cartilage in	MVGTKAW	5	6	23	6	1184	132.5	8.41	1246.8	1186.7	939.7	1242.8	1197.2	1059.7	1119.3	1295.6
A2JA19	Anti-mucin	DIQMTQSP	38	3	25	2	107	11.7	8.91	163	168.3	124	141.4	147	144.1	136.8	138.6
Q99497	Protein deg	MASKRALV	46	7	30	7	189	19.9	6.79	2063.6	1872.6	1541.6	1821.2	2230.1	1856.2	1771.2	1867.4
P18827	Syndecan-1	MRRAALW	12	2	58	2	310	32.4	4.63	1616.4	1237.2	1224.3	1457.9	1727.9	1534.3	1402.8	1571.3
Q9NPF0	CD320 anti	MSGGWM	14	4	49	4	282	29	4.75	2926.8	2570	1843.2	2671.6	3013.7	2917.4	2555.1	3112.6
P00915	Carbonic a	MASPDWG	18	4	31	4	261	28.9	7.12	1363.4	1485.8	623.1	738.5	1088.6	923.7	994.8	816.3
P04155	Trefoil fact	MATMENK	30	2	29	2	84	9.1	4.35	2198.7	2084.6	2264.9	2235.2	2342.5	3474.8	3091.6	2289.6
O95998	Interleukin	MTMRHNV	24	5	41	5	194	21.1	7.39	4158.9	3175.2	3770.7	3832.7	3758.2	4524	4016.7	4387.8
Q99969	Retinoic ac	MRRLLIPLA	21	3	21	3	163	18.6	9.09	505.7	508.8	437.5	486.1	541.1	481.2	430.8	449.9
E5RH16	Platelet-de	MRLPGAM	28	5	12	1	239	26.5	4.7	60.5	60.7	50.1	60.9	57.2	59.8	58.9	59.7
P62879	Guanine nu	MSELEQLR	19	6	27	2	340	37.3	6	1616.4	1366.8	1145.1	1487.9	1739.7	1691.8	1196.3	1397.1
Q01469	Fatty acid-	MATVQQL	25	3	34	3	135	15.2	7.01	934.5	1062.7	1055.3	1016.3	918.7	1262.6	1265.8	974.9
V9HWJ5	Nicotinate-	MDAEGLAI	26	7	17	7	297	30.8	6.21	1006	939.3	811.6	825.3	849.6	779.3	745.4	719.5
P23526	Adenosylho	MSDKLPYK	19	8	23	8	432	47.7	6.34	1319.8	1072.6	802	993.8	1252	1151.6	866.3	875
Q8TB96	T-cell immu	MAAAGRLI	12	6	24	6	612	68.1	5.39	442.7	408.2	358.1	417.6	417.3	414.8	370	386.3
P11279	Lysosome-	MAAPGSAI	8	4	74	4	417	44.9	8.75	5279.3	4792	3348.7	5389.9	5921.8	4877.7	5000.5	5517

D3YTC8	Leukocyte-	MSPHPTAL	13	4	83	1	286	31.3	5.95								
Q9BX67	Junctional	MALRRPPF	14	4	18	4	310	35	7.59	440.1	407.4	405.7	460.6	492.1	441.2	451.5	473.9
A0A024RD	EGF-like-do	MPSGCRCL	9	3	15	3	383	40.5	6.54	541.6	467.8	478.3	493	484.3	490.5	492.3	518.3
P19438	Tumor nec	MGLSTVPD	10	3	10	3	455	50.5	6.64	539.5	540.6	434.3	510.2	496.5	524.7	466.3	513.2
Q8NHP8	Putative ph	MVGQMY(	11	6	35	6	589	65.4	6.8	2171.8	1924	1367.5	2235.3	2171.5	2546	2186.1	2249.8
P05023	Sodium/po	MGKGVGR	8	7	11	7	1023	112.8	5.49	417.9	350.6	341.9	392.1	373	482.4	310.2	411.9
P20160	Azurocidin	MTRLTVLA	14	3	27	3	251	26.9	9.5	960.4	888.2	623.3	761.4	742.3	1039.5	905.4	611.6
B7Z5W1	cDNA FLJ54	MLQAPRGI	15	5	29	5	303	32.8	8.85	1509.2	1737.6	1145.9	1702	1928	1726.8	1606.1	1842.3
A0A140VJX	Sulfurtrans	MASPQLCF	22	5	16	5	297	33.2	6.6	549.9	448.9	369.6	470.2	472.9	576	509.9	403.3
A0A120HG	GCT-A10 h	QVQLQESG	25	3	36	1	127	13.6	8.81	1922.5	1638.4	1808	2062.5	1959.5	1575.8	1823.5	1703.1
Q5M8T4	Connective	MTAASMG	15	4	27	4	349	38	7.94	1202.6	1235.1	850.1	1162.3	1233	1104.8	1088.2	1247.4
Q9BRT3	Migration a	MSGEPGQT	32	4	24	4	115	12.4	4.37	1383.7	1089.7	975.1	1201.4	1505.4	1386.6	1229.2	1376.7
P49788	Retinoic ac	MQPRRQR	20	5	28	5	294	33.3	8.51	899.5	705	816.9	719.7	1750.1	767.3	819.1	965.3
P31025	Lipocalin-1	MKPLLLAV	21	4	38	4	176	19.2	5.58	1277.4	1986	1010.4	1643.2	1591	1342.4	1319	1461.4
A0A075B6I	Protein IGK	MEAPAQLL	23	2	146	1	116	12.8	5.25	409.2	411.4	299.4	370.9	404	428.4	433.5	489.9
Q9BZG9	Ly-6/neuro	MTPLLTLIL	18	2	152	2	131	14	6.71	7236.8	6557.9	4311.3	6534.3	7253.8	6545.8	5953.8	8447.9
A2VCQ3	ROR1 prote	RARGCSAR	4	3	16	2	940	104.4	7.23	890.4	802.8	658.5	911.6	893.9	1004.3	840.5	951.7
P36957	Dihydrolipo	MLSRSRCV	7	3	60	3	453	48.7	8.95	5434.4	4096	3714.7	4739.8	5642	5392.6	4462.1	5668.5
A0A1C9J6T	B cell recep	EVQLVQSG	23	2	42	2	132	14.4	8.03								
A0A024R1A	Testicular s	MSSSPLSKI	6	4	8	4	1058	117.8	5.76	235.7	162.6	154.9	138	175.3	166	144.1	133.1
Q5JWF2	Guanine nu	MGVRNCLY	5	5	25	4	1037	111	5.03	945.2	776.6	641.8	896	1042.8	887.2	665.8	907.3
A0A0G2JM	Cadherin-r	MGSWALL	6	4	14	4	845	88.1	4.91	287.3	188.5	181.9	193.4	244.6	218.6	205	249
P13473	Lysosome-	MVCFRLFP	7	3	113	3	410	44.9	5.63	7456.8	5745.4	4694	8648.5	8424.4	8179.5	8798.8	8958.6
A0A024R93	Proteoglyc	MAWKTLP	4	6	46	1	1404	151	9.5	5246.9	6325.3	3913.3	5340.3	5389.9	4946.1	4970.3	5168.8
P12821	Angiotensi	MGAASGR	6	7	21	7	1306	149.6	6.39	740.1	581.7	632.1	680.9	787.1	598.5	535.7	747.9
B7Z4R8	cDNA FLJ53	MAWKTLP	7	6	45	1	853	89.5	8.91	41.2	45.6	32.5	41.7	43	39.1	46.8	47.5
A0A024R99	Copine III,	MAAQCVT	13	6	14	6	537	60.1	5.85	691.8	541.4	644.4	631.9	758.2	614.3	679.3	638.9
Q5U0I6	H.sapiens r	MSSMNPEY	22	4	25	2	205	22.7	6.21	686.2	503.6	422.1	533.1	639.4	578.7	473.3	545.7
A0A068LL6	Ig heavy ch	QVQLVQSC	25	3	28	1	120	13.3	7.93	743.5	507.5	616.8	684.1	647.7	614.2	812.6	689.2
A0A024R84	RAB14, me	MATAPYNY	24	5	21	4	215	23.9	6.21	917.1	801.1	655.2	803.2	925.2	778.1	659.6	825.9
P12814	Alpha-actin	MDHYDSQ	7	6	16	1	892	103	5.41								
Q14393	Growth arr	MAPSLSPG	8	4	8	4	721	79.6	6.21	327.5	255.8	262.6	325.5	352.3	338	295.3	343.2
P05413	Fatty acid-I	MVDAFLGT	53	7	18	7	133	14.8	6.8	1274.9	1111.2	1041.8	1062.1	1301.3	1110.2	1069.6	1140.6
A0A140VJT	Testicular t	MSLDIQSLI	13	5	10	5	461	49.9	4.82	400.6	273.3	353.4	316.9	365.3	342.3	300.4	298.5
A0A0C4DH	Immunoglo	MDMRVPA	40	3	36	1	117	12.7	8.29	715.6	871	621.8	757.6	637.5	602.2	673.1	606.6
Q09666	Neuroblast	MEKEETTR	6	8	15	8	5890	628.7	6.15	663.1	632.7	568.9	614.5	578.4	594.6	610.6	587.5
P14550	Alcohol de	MAASCVLL	18	5	18	4	325	36.6	6.79	410.8	336.3	313.8	345.3	456.9	424.2	442.7	354
Q16832	Discoidin d	MILIPRMLI	3	2	16	2	855	96.7	5.36	459.6	366.7	357.2	390.8	492.4	440.7	357.1	411.7
P16284	Platelet en	MQPRWA(	10	6	18	1	738	82.5	6.99								
Q9HC84	Mucin-5B (	MGAPSACF	2	4	11	4	5762	596	6.64	643.3	529.5	669.4	613.9	811.7	567.5	538.9	821.2
Q92859	Neogenin (	MAAERGAI	6	7	14	7	1461	159.9	6.54	786.5	701.2	594.3	757.9	790.5	746.3	683.4	797.5

Q7Z738	PCDH12 pr	MMQLLQL	5	4	8	4	1187	129.2	5.26	207.9	150	237.3	168.5	164.1	183.6	157.8	177.3
Q05CP7	FABP1 prot	MSFSGKYC	34	4	33	4	134	15.1	9.51	2216	1837.7	1758	1669.3	2070.9	1954.2	1419.3	1692.4
H0Y3Z8	Uncharacte	XDLTDEWF	15	4	24	4	333	38.4	4.84	733.1	563.1	587	656.7	701.9	724.7	661	789
A0A024RA	Glutaredox	MAQEFVN	43	4	35	4	106	11.8	8.09	764.1	984.8	498.7	703.7	803.2	812.2	651.2	717.9
Q99988	Growth/dif	MPGQELRT	8	2	19	2	308	34.1	9.66	1396.2	1660.6	867.2	1524.9	1300.5	1446.9	1648.4	1308.6
A0A087WV	Immunoglo	MRLPAQLL	22	2	78	1	121	13.3	4.61	333.2	383	336.3	385	318.4	302.9	297.2	286.9
Q14315	Filamin-C (	MMNNSGY	3	6	9	6	2725	290.8	5.97	545.7	449	372.6	492.7	499.1	492.8	391.6	464.2
A0A0X9V9	MS-F1 light	DVVMTQSI	33	2	34	2	112	12.1	5.96	1476.4	1846.8	1351	1398.8	1486.8	2127.1	1949	1807.9
A0A125U0	GCT-A1 he	QVQLQESG	25	3	32	1	127	13.7	8.78	106.2	88.3	83.2	106.5	105.8	164.3	97.5	105.3
Q9BVM4	Gamma-glu	MALVFVYG	35	4	20	4	153	17.3	6.87	840.9	684.9	543	658.2	753.6	840	641.6	675.7
Q5IJ48	Protein cru	MALARPGT	6	5	21	5	1285	134.2	5.55	177	176.9	129.6	146.6	163	191.6	151.1	157.7
Q14247	Src substra	MWKASAG	5	2	35	2	550	61.5	5.4	1053.6	1066.9	675.4	1153.7	1152	1409	985.8	1133.4
A0A075B7	Platelet en	MQPRWAC	10	6	19	1	738	82.5	6.99	1254.7	1009.5	957.3	1165.9	1238.1	1242.9	1141.4	1259
P13671	Compleme	MARRSVLY	6	4	16	4	934	104.7	6.76	671.3	485.9	566	606.5	682.6	704.2	721.3	601.5
A0A024R3	Filamin B, l	MPVTEKDL	3	6	21	6	2622	280.3	5.94	528.1	466.7	436.8	526.7	484.2	578.2	464	491.2
Q0IIN1	Keratin 77	MSHQFSSC	6	4	32	1	578	61.8	5.85								
B2R6X2	Beta-glucu	MARGSAVA	9	6	14	6	651	74.7	7.02	799.7	637.9	550.7	661.5	939	651.2	616.5	770.1
P05556	Integrin be	MNLQPIFW	5	5	20	5	798	88.4	5.39	1658.1	1430.4	1255	1600.5	1658	1609	1466.7	1556.3
P55017	Solute carr	MAELPTTE	7	6	24	6	1021	113.1	7.88	602.3	476.9	399.7	580	657.4	615	457.6	580.6
P02461	Collagen al	MMSFVQK	2	3	14	3	1466	138.5	6.61	1504.5	1365.4	890.7	2066.9	1607.6	1983.7	2122.8	1488.3
A0A0S2Z2	Annexin (F	MAKPAQG	10	7	20	7	673	75.8	5.6	1041.6	728	683.4	838	870.3	895.2	700.5	709.9
B1N7B6	Cryocrysta	QVQLVESG	32	3	25	3	120	13.2	8.48	1379.4	1130.3	1393.4	1481.1	1286.5	1308.8	1399.1	1256.3
A2NJV5	Kappa light	MRLPAQLL	20	2	74	1	121	13.2	7.28	86.5	132.6	79.8	100.4	118.7	129.3	135.3	127.9
Q86Y38	Xylosyltran	MVAAPCAI	2	2	6	2	959	107.5	9.22	508.5	548.5	434.3	608.2	512.6	666.5	465.9	547.6
Q96AP7	Endothelia	MISLPGPLV	8	3	15	3	390	41.2	9.32	949.4	1030.7	771.5	983.9	1078.9	1073.7	948.1	1100.2
A0A024RD	G protein-c	MKSPRRTT	5	6	17	6	1346	149.3	6.65	325.9	314.8	275.7	293.6	341.7	312	274	327.9
Q16651	Prostasin C	MAQKGVL	4	1	16	1	343	36.4	5.85	1888.9	2031.5	1920.1	1707.8	2054.7	1905.5	1788.3	2330.8
P12429	Annexin A3	MASIWVGI	22	6	14	6	323	36.4	5.92	175.6	148.9	160	183.5	145.8	149.1	138.5	120.9
A8K0I8	cDNA FLJ7	MQPRRAQ	3	2	12	2	737	78.5	5.21	553.4	460.4	432.1	512.7	562	518.6	462.7	574
P01766	Immunoglo	MELGLSWV	19	2	19	1	116	12.5	7.08	143.9	143.3	137.3	178.7	125.9	133.1	141.5	115.1
O60888	Protein Cu	MSGGRAPA	16	2	36	2	179	19.1	5.5	802.2	671.5	646.8	717.4	852.5	839.7	722	922.3
B2R657	Annexin OS	MSYPGYPP	7	3	7	3	488	52.6	5.52	87.2	56	72.6	72	85.5	75.1	64.4	64.6
Q6FIE5	PHP14 pro	MAVADLAI	36	4	22	4	125	13.8	6.07	2307.3	1936.1	1452.8	2029.7	2186.5	1953.3	2081.4	1824.2
B2RAM2	cDNA, FLJ9	MDATALEF	7	5	16	5	813	92.6	7.65	772.1	718.3	675.4	724.5	780.6	623	528.4	670.9
O00337	Sodium/nu	MENDPSRF	3	1	26	1	649	71.5	7.59	189	148.6	147.2	183.1	184	125.3	147.1	198.9
A8K987	cDNA FLJ7	MAEKPKLH	18	5	71	1	222	25.7	9	4857.3	3701	2361.3	3403.1	5215.5	3920.5	2928.1	3254.9
P08263	Glutathion	MAEKPKLH	18	5	78	1	222	25.6	8.88	515.5	439.4	348.2	386.9	470.7	586.6	350.8	331
A0A024R5	Dihydroxya	MTSKKLVN	11	5	13	5	575	58.9	7.49	448.1	386.8	288.1	368.8	456.2	513	339.9	347.7
P40189	Interleukin	MLTLQTW	6	5	24	5	918	103.5	5.95	1502.2	1535.8	1236.8	1489	1571.1	1537.7	1304.8	1487.4
Q5NV90	V2-17 prot	SYELTQPPS	41	3	20	3	97	10.4	4.59	719.5	731.6	703	678.5	774.5	746.6	714.1	601.5
P22528	Cornifin-B	MSSQQQK	44	3	24	1	89	9.9	8.48	1716.5	2446.7	1832.6	2536.8	2307.6	1763.9	2761.7	2109

Q9UN70	Protocadh	MVPEAWR	6	6	25	6	934	101	5.21	2723.8	2196.7	2021.5	2364.5	2665.5	2478.2	2172.8	2507.6
A8MTF8	Protein FA	MKSHRGD	17	4	16	4	258	28.3	8.73	701.2	620.2	492.3	644.8	757	662	621	692.4
P20336	Ras-relate	MASATDS	20	4	15	1	220	25	5.03	288	323.8	274.1	270.9	263.1	213.9	203.9	264.4
Q8WVQ1	Soluble cal	MPVQLSE	14	4	14	4	401	44.8	6.09	330.6	258.7	273.8	272.7	352.1	284	239.9	308.3
B8PRF2	CD160 anti	MLLEPGRG	15	3	21	3	234	26	8.56	721.9	696	675.2	659.5	763.6	935.9	605	779.3
A0A0A0M	Coagulatio	MFPGCPRL	1	3	30	3	2229	252.1	6.05	300.9	315.4	245	312	361.4	336.1	333.1	358.9
Q7LBR1	Charged m	MSNMEKH	19	5	37	5	199	22.1	8.1	2648.7	2505.2	2824	3025.5	3153.4	2759	2295.7	2977.5
P13797	Plastin-3 O	MDEMATT	9	6	20	3	630	70.8	5.6	72.7	101.6	41.1	42.1	40.3	39.3	40.6	38.2
Q53G35	Phosphogl	MAAYKLVL	16	3	10	3	254	28.8	7.18	281.4	242.1	226.4	255.6	219.9	238.6	228	231.3
Q9NY97	N-acetyllac	MSVGRRR	9	3	14	3	397	46	8.54	919.2	824.8	809.8	820.4	864.8	836.3	737.5	721.8
A0A024R1	LIM and SH	MNPNCAR	16	3	30	3	261	29.7	7.05	403.7	359.7	339.5	378.4	432.5	367.5	354.1	429.2
Q9Y509	VH3 protei	QVHLVESG	16	2	77	1	147	15.8	8.38	906.8	727.1	827.2	808.2	768.7	750.8	821.1	704.9
G8JLH6	Tetraspani	MPVKGGT	7	3	62	3	228	25.4	6.52	6145.2	6435.4	5012.1	7231.1	8554.5	5538.3	4966.4	7477.1
Q9BTY2	Plasma alp	MRPQELP	11	5	18	5	467	54	6.25	746.3	610.9	619.2	687	796.3	708.6	715	625.1
P22732	Solute carr	MEQQDQS	6	3	22	3	501	54.9	6.04	1530	1084.8	1228	1507.9	1666.2	1386.6	903.4	1407.4
P35443	Thrombos	MLAPRGA	9	5	9	4	961	105.8	4.68	119.7	124.8	148.9	135.3	123.5	121.2	138.8	147.1
A0A0A1HA	H.sapiens	MASVTDG	22	4	14	1	219	24.7	5.11								
Q9UL84	Myosin-rea	EVQLVESG	28	3	29	2	122	13.6	8.46	307.4	208	219	216.9	226.7	233	343.2	216.8
Q9NQ36	Signal pept	MGVAGRN	3	2	7	2	999	109.9	6.65	589	696.4	445.5	694	649.5	673.3	556.2	688.2
A8K2Q6	Peptidyl-pr	MGPGPRL	10	2	28	2	212	22.7	8.4	821.1	599.6	531.2	791.4	838.2	931.8	658.8	712.3
Q14894	Ketimine r	MSRVPAFL	12	3	13	3	314	33.8	5.14	566.4	422.1	567.6	444.8	511.4	480.9	432.6	400.1
Q9ULV1	Frizzled-4	MAWRGA	4	2	39	2	537	59.8	7.27	2061.7	2021.7	1398.8	2209.9	2362.7	1983.4	1839.2	2293.8
Q9Y2S2	Lambda-cr	MASSAAG	13	4	25	4	319	35.4	6.18	1726.9	870.9	639.3	725.1	893.1	1034.1	693	653.3
Q6UX71	Plexin dom	MARFPKA	12	6	13	6	529	59.5	6.46	1042.3	812.5	697.6	857.4	847.8	987.5	751.1	879.6
P04066	Tissue alph	MRAPGM	9	4	22	4	466	53.7	6.84	782	578.6	594.6	729.5	854.4	783.6	660.8	592.1
Q9H6X2	Anthrax to	MATAERR	10	5	12	5	564	62.7	7.61	915.6	1105.3	892.6	928.1	932.3	905.1	923.1	962.8
P20138	Myeloid ce	MPLLLLLPL	12	4	13	4	364	39.8	8.38	975.8	822.3	754	875	806.8	915.9	748.2	880.8
E7EQR8	Protein YIP	MATTAAP	3	1	216	1	356	38.9	5.6	21072.3	17617.5	18663	21307.5	22466	21160	20061.3	23740.1
A0A024RE	Prominin 2	MKHTLALL	8	6	20	6	834	91.9	6.24	1009	945.6	802.3	1009.4	1102.6	1014.6	764.5	960.6
P35321	Cornifin-A	MNSQQQK	44	3	27	1	89	9.9	8.48	339.1	372.9	500.2	379.1	361.8	330.9	437.9	378.6
P36896	Activin rec	MAESAGA	6	2	37	2	505	56.8	7.03	1670	1388.4	1232.7	1677.5	1893.2	1729.5	1604.6	2014
Q9H444	Charged m	MSVFGKLF	25	5	11	5	224	24.9	4.82	478.7	411.2	382	419.4	476.3	460.7	348.4	418.7
A6NMH8	Tetraspani	MWCGWS	13	2	10	2	274	29.8	6.92	203.9	202.5	155	194.5	184.8	193.4	159.1	183.4
Q8WV92	MIT domai	MAKSGLR	21	4	6	4	249	29.3	8	385.2	284	348.4	363.2	398.2	463.8	295.9	314.4
A8K4E0	cDNA FLJ7	MDPARKA	15	4	8	4	346	36	7.75	401.7	325.8	258.6	369.2	335.7	380.5	323.5	377.6
A0A0X9T0	GCT-A5 he	EVQLVQSG	26	3	22	3	114	12.7	7.96	1363.3	1109.9	1365	1454.9	1610.6	1234.6	1571.4	1288.3
Q8N7G1	Purine nuc	MENGYTY	19	5	11	5	293	32.5	7.21	184.8	121.1	132.2	134.9	177.7	163.1	132.4	121.9
A8K8G3	cDNA FLJ7	MRAPGCG	12	5	11	5	424	46.8	4.83	1113.3	939	913.6	1008.8	1122.8	1067	898.4	1058.4
D6RAR4	Hepatocyt	MGRWAW	11	6	22	6	662	71.4	7.05	1206.9	1120	1146	1360.3	1370	1166.7	1038.7	1403.6
Q9NP29	Microfibril	MVLQTQV	18	2	67	1	130	14.1	9.64	570.7	720.9	475.9	681.6	792.3	561.8	614.9	648.7
B2R7D2	cDNA, FLJ9	MLRAPGCL	13	5	9	5	487	55.1	7.97	294.3	201.4	207	188.3	223	258.5	184.8	181.1

Q8NC42	E3 ubiquiti	MAWRRRE	13	4	22	4	400	43.1	6.54	1022.1	916.9	837.9	985.8	942.7	1003.4	905	998.1
P27105	Erythrocyt	MAEKRHTF	25	6	16	6	288	31.7	7.88	561.6	422.1	399.2	493.6	496.4	522.5	417.1	432
P81172	Hepcidin O	MALSSQIW	19	1	63	1	84	9.4	8.81	2372.8	2930.4	1814.3	2823.5	2868.3	2697.5	2796.6	2320.3
Q9HBR0	Putative sc	MTAAAASN	5	4	7	4	1119	119.7	5.73	217.9	174.9	171.3	211.2	219.9	230.7	158.7	203.3
Q5VW32	BRO1 dom	MTHWFHR	15	5	13	5	411	46.4	7.65	605.9	544.8	530.8	617	739.6	672.3	546.9	680.8
Q15286	Ras-relatec	MARDYDH	15	3	23	1	201	23	8.29								
A0A075B6.	Immunoglo	MAWALLLL	25	2	14	2	118	12.4	4.82	900.5	1060.4	755.2	1166.2	1020.6	1022	1031.3	1136.9
Q0ZCF6	Immunglob	PLVQLVESC	18	2	21	1	129	14.5	8.78								
P10645	Chromogra	MRSAAVLA	5	2	26	2	457	50.7	4.6	1315.1	1390.3	847.6	1127.3	1153.3	1322.4	899.4	1150
A2IPI6	HRV Fab 02	MAELQMT	20	2	44	1	113	12.4	9.41	275.8	326.1	265.7	311.2	295.8	329.5	338.2	288.1
P01040	Cystatin-A	MIPGGLSE.	38	3	20	3	98	11	5.5	1120.2	1185.4	1183.6	1327.6	1145	1292.5	1320.2	1303.1
Q14019	Coactosin-	MATKIDKE	23	4	32	4	142	15.9	5.67	3304	3709	2098.1	2691.4	3876.4	2990.7	2315.1	2695.4
B0YIW2	Apolipopro	MGTWGAF	29	3	12	3	117	12.8	8.18	689	823	543.1	534.9	585.2	744.4	578.8	535.4
Q9UKU6	Thyrotropi	MGEDDAA	7	6	14	6	1024	116.9	6.99	567.4	457.8	343.5	411.3	412.6	399.1	332.2	353.4
A0A158RFU	RAB7, men	MTSRKKVL	18	3	13	3	207	23.5	6.7	376.1	256.4	293.9	314.5	335.4	336.9	291.1	305.3
Q9GZM7	Tubulointe	MWRCPLG	12	4	12	4	467	52.4	6.99	416.6	336	312.1	391.7	357.4	343.1	346.4	355.7
P55259	Pancreatic	MPHLMER	9	4	23	4	537	59.4	5.24	152.2	187.9	136.9	168	162.6	120.5	134.8	152.2
Q8N289	cDNA FLJ33	METCAGPH	11	3	10	2	295	32.1	5.91	132.1	133.3	127.7	161	140.4	113.5	112.5	130.7
Q8WW52	Protein FAI	MVCREQLS	7	4	17	4	585	64	6.67	989.6	689	567	737.5	830.1	912.5	609.1	851.1
A0A024R1I	RAB5C, me	MAGRGGA	22	4	13	4	216	23.5	8.41	473.8	354.3	363.6	469.5	529.5	408.8	345.6	412.1
A0A090KF\	HLA class I	MRVMAPR	13	4	7	2	366	40.9	6	171.7	135.1	103.3	147.5	142.7	137.3	120.9	138.3
P20062	Transcobal	MRHLGAFL	14	6	10	6	427	47.5	7.01	313.5	235.1	220.3	266.1	274.3	299.1	246.5	241.5
Q5IWS5	Intelectin 1	MNQLSFLL	16	4	13	4	313	35	5.82	662.1	403	407.9	509.9	416.2	593.9	316.1	793.9
P15121	Aldose red	MASRLLLN	12	4	11	3	316	35.8	6.98	178.4	196.8	143.8	186	255.8	232.8	304.2	209.9
O43692	Peptidase i	MIAISAVSS	8	2	33	1	258	29	8.03	2388.7	1711.9	1882.8	2695.6	2144.6	2346.3	2147.8	2345.7
O95460	Matrilin-4	MRGLLCW	4	2	21	2	622	68.4	6.1	769.4	532.9	555	706.2	654.5	616.9	491.1	789.3
B4E1U9	cDNA FLJ54	MQTIKCVV	16	3	7	3	236	26.5	7.59	502.9	394.2	338.7	400.9	534.3	441.2	423.8	373.9
V9HW62	Lactoylglut	MAEPQPPS	22	5	26	5	184	20.7	5.47	1304.7	1214.5	901.8	1117.3	1269.3	1140.9	975.5	1116.1
Q2UY09	Collagen al	MWNRYFV	2	2	30	2	1125	116.6	6.4	1460.8	1358.3	988.9	1457.2	1426.5	1433.7	1260.7	1588.2
Q06481	Amyloid-lik	MAATGTA/	8	5	11	5	763	86.9	4.79	922.6	901.1	712.4	887.7	820.9	885.7	697.6	659.8
Q15746	Myosin ligh	MGDVKLV/	1	2	19	2	1914	210.6	6.15	1140.3	958.7	861.9	1077.8	1058.3	1254.5	1083.5	1162.6
P21399	Cytoplasm	MSNPFAHL	6	4	8	4	889	98.3	6.68	385.3	309.4	258.9	321.4	372.5	330	271.2	281.1
P02792	Ferritin ligh	MSSQIRQN	18	3	7	3	175	20	5.78	159.7	121.1	113.5	119.3	109.9	109.6	129.7	100.4
P04899	Guanine nu	MGCTVSAE	10	3	20	2	355	40.4	5.54	172.2	160.6	149.7	205	221.7	157.2	160.8	147.3
V9HW91	Epididymis	MTSFRLALI	22	4	7	4	276	30.6	7.21	119.5	65	66.2	66.4	86.5	90.5	57.3	65.2
A8K037	cDNA FLJ77	MEGAPPGS	8	3	15	3	330	36.8	4.82	774.5	651.3	508.3	670.9	654.8	785	593.8	651.7
A6NC48	ADP-ribosy	MAAQGCA	10	3	12	3	333	37.5	7.97	279	248.2	202.9	261.6	249.8	246.8	230.9	286.2
Q53GE4	Leucine ric	MKVTGITIL	10	3	6	3	370	42.3	5.12	266.2	221.6	206.3	239.9	239	252.9	195.6	228.6
B4DRY1	cDNA FLJ54	MLLWILLLE	5	3	11	3	696	77.7	6.61	347.9	348.9	265.1	318.1	347.4	332.6	311.7	306
P50053	Ketohexok	MEEKQILC\	16	5	10	5	298	32.5	6.32	505.3	381.4	294.5	366.7	496.3	422.9	352.5	379
A0A024R7I	Solute carr	MGDERPH	8	6	15	6	706	80.1	8.62	989.2	1000.2	753.4	1022.9	1044.3	840.7	773.4	864.2

A0A024R8!	Protein dis	MLRRALLC	12	6	11	6	508	57.1	4.87	282.9	214.3	206.7	228.6	215.5	232.5	214	223.5
K7N7A8	Uncharacte	XETLTLHSL	4	1	3	1	449	48.8	5.22	13.6	13.8	15.8	16.1	18.9	15.1	14.2	12.6
A0A109PP8	MS-C2 ligh	DIRMTQSP	14	1	6	1	107	11.7	8.5	675	642.2	563	554.6	610.9	787.8	625.4	644.8
A0A087WV	Trypsin-1 C	MNPLLILTF	16	3	34	1	247	26.6	5.86	934.1	517	804.9	753	588.5	698.1	612.1	683
B7ZLM6	X-prolyl am	MARAHWC	8	5	11	5	674	75.6	6.04	475.6	392	298.2	425.1	434.6	393.2	281.5	399.9
Q96DR8	Mucin-like	MKFLAVLV	18	2	78	2	90	9	4.64	7509.4	8039.1	6954.1	8901.6	8034.1	4241.8	5934.1	7477.2
P36639	7,8-dihydro	MYWSNQIT	7	1	20	1	197	22.5	5.27	46.7	35.7	27.4	37.7	45	45.8	39.6	46.1
A9UFC0	Caspase 14	MSNPRSLE	14	4	10	4	242	27.6	5.34	215.4	233.5	178.4	257.8	218.9	253.9	274	272.4
Q15952	Agrin (Frag	ETCGDAVC	60	2	5	1	62	6.3	4.64								
V9HW48	SH3 domai	MVIRVYIAS	35	3	13	3	114	12.8	5.25	89	114.7	92.2	70.4	60.5	68	75.6	56.2
P02743	Serum amy	MNKPLLW	16	3	18	3	223	25.4	6.54	912.8	727.3	697.9	704.4	811.7	880.2	977.9	1124.2
Q68D85	Natural cyt	MTWRAAA	9	3	6	3	454	50.8	5.59	193.7	177	164.2	180.1	188.2	189.8	183.7	202.5
D3DRR6	Inter-alpha	MKRLTCFF	5	4	10	4	947	106.5	6.95	446.9	336	459.8	351.5	296.5	432.7	327.5	249.7
Q59FG9	Chondroiti	IFPPNCNKF	1	3	13	2	3410	374.1	4.53	499.4	334.5	388.2	461.9	497.8	485.2	436.1	517.4
P48960	CD97 antig	MGGRVFLA	2	1	6	1	835	91.8	6.87	338.7	308.5	258.8	295.4	365.7	418.6	266.7	338
O43291	Kunitz-type	MAQLCGLF	10	2	15	2	252	28.2	8.29	788.1	788.7	558.5	759.9	858.5	751.6	781.3	935.7
A8K2X4	cDNA FLJ75	MDRGTLPL	6	3	19	3	658	70.5	6.61	254.2	221.6	278.7	245.1	246.7	241.7	214.8	236.8
P21796	Voltage-de	MAVPPTYA	15	4	7	4	283	30.8	8.54	291.5	253.3	237.4	276.9	231.4	286.4	266.2	204.8
A0A140T8X	Lysosomal	MKSCGSM	10	2	10	2	308	34.9	6.33	282.1	214.6	187.1	265.8	266.3	310.6	229.1	225.9
B2R778	cDNA, FLJ9	MILQAHLH	5	3	6	3	832	92.1	5.11	338.4	255.6	273.4	265.3	293.1	386.7	219.8	323.7
B3KWI4	cDNA FLJ43	MPLKHYLL	6	3	9	3	581	64.4	6.71	186.2	141.6	156.4	150.5	144.1	154.9	137.6	120.9
A0A140VK	Aspartate a	MAPPSVFA	14	5	9	5	413	46.2	7.01	572.1	435.2	451.3	490.8	626.6	470.5	402	402.1
O60635	Tetraspani	MQCFSFIKT	5	1	5	1	241	26.3	5.25	181.3	116.9	104.5	126.7	132.7	135.5	103	121.1
Q5T2L0	V-set doma	MVLLTSRM	6	2	18	2	285	31.2	5.99	1386	624.6	758.7	844	832	907.4	811.8	960.5
P24158	Myeloblast	MAHRPPSF	17	3	17	3	256	27.8	8.35	615.9	413.6	295.9	368.9	390.2	623	677.4	289.9
P68371	Tubulin bet	MREIVHLQ	11	4	10	4	445	49.8	4.89	26.1	18.2	35.9	20.9	20.3	22.9	26.6	23.6
A0A075B6I	Immunoglo	MSVPTMA	15	2	29	2	122	12.8	4.55	1770.1	2483	2168.7	2079.2	1873.2	3276.7	2795	2023.3
P53801	Pituitary tu	MAPGVAR	14	2	19	2	180	20.3	8.79	719.7	593.6	498.6	672.6	683.5	692.9	584.8	688.6
B2R907	cDNA, FLJ9	MSDSKEPR	9	3	4	3	404	45.7	5.3	280.5	261.4	223.3	247.2	299.8	352.9	286.9	346.7
A0A024R5T	EH domain	MEQPGTA	5	3	9	2	548	61.9	6.71	230.6	191.1	140	221	275.8	256.4	187.4	231
Q99536	Synaptic ve	MSDEREVA	12	3	5	3	393	41.9	6.29	147.4	135.9	133.5	135.5	144	146.5	129.6	130.3
E7EUF1	Ectonucleo	MTRHADR	1	1	7	1	884	101.5	7.53	382.5	334.8	317.6	321.1	371.3	344.8	278.6	343.1
P20933	N(4)-(beta-	MARKSNLF	14	4	9	4	346	37.2	6.28	461.4	372.8	418.8	441.1	415.2	405.2	402.6	404.3
A0A0S2Z3I	Dopa deca	MNASEFRF	10	5	16	5	480	53.9	7.2	623.3	493	418.8	507	614.7	582.5	496.5	494.9
Q6UX15	Layilin OS=	MRPGTALC	8	4	7	4	382	43.1	4.94	465.8	467.6	353	491.7	476.1	528	409.6	442.6
B4DKN3	cDNA FLJ50	MERAEEPV	9	3	8	3	499	51.1	6.14	248	225.7	183.8	210.3	240.4	248	204.9	210.3
A0A0A0MT	UPF0764 p	MASLGLLLI	8	4	23	4	434	48.8	5.99	947.7	795.9	793.3	743.5	1208.5	899	733.4	879.9
Q66K79	Carboxype	MPPPLPLLI	3	2	29	2	652	73.6	7.97	2090.1	2060.2	1403.2	2560.9	2240.2	2475.9	2134.7	2103.6
B4DR52	Histone H2	MPDPAKSA	20	3	22	3	166	18	10.32	1928	1482.9	826.5	1087.7	1195	1902.3	2601.2	992.5
B3KQS9	cDNA PSEC	MIPLLLAAL	10	3	21	3	360	39.5	8.05	918.8	688.6	633	750.2	900.2	788.1	835.3	773.7
Q08257	Quinone ox	MATGQKLI	12	3	12	3	329	35.2	8.44	477.5	325.6	294.8	327	454.5	355.2	293.3	319.8

Q59G10	Aldehyde d	QSSRTGGD	7	5	7	5	954	104	6.42	215.2	158.9	151.1	161	201.3	219.5	148.9	170.9
B4DLV7	Rab GDP d	MSLGKRKP	10	4	15	3	449	51.1	8.18	621.6	512.2	420.2	524.1	562	568.7	475.1	472.6
P30043	Flavin redu	MAVKKIAI	12	2	5	2	206	22.1	7.65	203.2	171.6	166.2	173	162.3	185.4	150.6	143.4
Q400G7	B and T lym	MKTLPAM	7	1	4	1	289	32.8	6.34	92.5	71	80.2	75.5	90.5	92.8	91.7	106.5
Q9Y3B3	Transmem	MPRPGSA	16	3	9	3	224	25.2	6.89	430	442	374.7	411	450.4	400	435.7	446
P59665	Neutrophil	MRTLAILA	19	2	29	2	94	10.2	6.99	2669.7	2632.3	1359.4	2256.9	2139	4014.5	4344.9	1746.8
A0A0R7FJH	Coagulatio	MRALLLLG	6	2	6	2	615	67.8	7.8	132	114.1	91.6	96.1	89.6	107.7	95.5	83.3
B2RBC8	cDNA, FLJ9	MEKRLGVK	2	2	13	2	1049	115.5	6.11	762.3	686.1	530.6	751.3	847.2	764.2	717	875.9
P16035	Metallopro	MGAAART	13	3	9	3	220	24.4	7.49	808.9	682	925.9	901.6	865.5	873.9	706.7	890.7
P61204	ADP-ribosy	MGNIFGNL	18	3	14	3	181	20.6	7.43	671.3	534.7	486.1	627.4	643.2	608.7	613.1	528.6
A8K6C1	cDNA FLJ7	MLAATVLT	11	5	10	5	493	54.7	6.09	508.1	509	420.8	490	501.5	492.2	482.6	461.4
V9HWH9	Protein S1	MAKISSPTE	28	3	17	3	105	11.7	7.12	1275.4	1048.9	979.6	1149.1	1119	1259.7	1780.4	1224.2
A0A1B0RP	Fibroblast	MWSWKCL	4	3	16	3	822	91.8	6.15	546.6	460.8	379.5	479.6	487.4	484.2	446.5	493.9
O00182	Galectin-9	MAFSGSQ	6	2	11	2	355	39.5	9.17	1045.3	676.1	737.8	930.6	1037.1	1067.8	872.9	878.9
B2R7S7	cDNA, FLJ9	MTSTFNPR	8	4	6	4	519	57	5.49	199.8	176	131.7	186.5	198.7	197.4	149.8	165.1
Q9NZT1	Calmodulin	MAGELTPE	27	3	11	3	146	15.9	4.44	150.4	119.4	135.3	141.9	147.2	146.9	187.8	169.4
A0A0G2JP	Leukocyte-	MSPHLTAL	15	3	38	2	208	23	7.09	358.4	324.8	293.2	266.2	374.8	307.5	300.5	339.7
P36871	Phosphogl	MVKIVTVK	9	5	7	5	562	61.4	6.76	459.6	364.2	431.6	387.2	432.3	432.8	369	380.9
A2N7P4	Immunoglo	AHSQVQLV	28	3	10	1	120	13.3	9.26								
Q96SB0	Anti-strept	QSVLTQPP	22	2	13	1	108	11.6	6.54	103.3	105.1	83.6	124.1	95.9	159.3	128.6	94.1
A0A024R9	EH-domain	MFSWMG	6	3	10	2	541	61.1	6.76	83.8	62.1	80.9	65.4	73.5	75.6	66.8	74.6
X5DR95	Semaphori	MKGTCVIA	3	3	6	3	1074	120.5	7.21	163.8	133.1	125.9	174.9	182.3	167.5	128.8	177
Q99816	Tumor sus	MAVSESQL	7	3	6	3	390	43.9	6.46	528.8	361.9	382.6	413.9	458.6	540.4	350.4	390.7
P20333	Tumor nec	MAPVAVW	6	2	7	1	461	48.3	6.28	737.1	596.5	516.7	657.8	571.3	1127.5	680.3	706.7
Q5U043	S-(hydroxy	MANEVIKC	9	3	8	3	374	39.7	7.49	298	217.4	185.4	220.4	254.7	214.3	197.9	183
Q5JRA6	Melanoma	MAAAPGLL	2	3	12	3	1907	213.6	4.84	285	309.2	227.4	296.8	275.4	256.7	276.2	297.4
I3L4C2	Brain-speci	MSLSRSEE	4	2	5	2	553	61.3	8.94	79.9	66.5	81.2	67.8	88.2	69.2	63.5	77.2
Q9BRA2	Thioredoxi	MARYEEVS	27	3	15	3	123	13.9	5.52	875.4	691.2	566.4	824.8	928.7	889.1	769.6	785.9
Q9UL89	Myosin-rea	VQSGAEVK	16	2	9	2	116	12.6	9.1	104.1	79.9	126.5	115.9	75.1	109.9	102.6	84.9
A0A0A6YY	Trefoil fact	MKRVLSCV	12	1	45	1	94	10.2	6.92	5290.9	6041.5	3228.5	5198.1	5099.7	4859.1	5565.3	4753.6
A8K556	cDNA FLJ78	MATTVPD	6	2	4	2	357	40.3	8.15	58.7	55.2	41.1	54.3	68.5	61.2	78.9	66.2
P41181	Aquaporin-	MWELRSIA	8	2	14	2	271	28.8	6.93	769.4	814.4	541.7	802.1	943.5	759	695.1	738.1
A0A024R7	Interferon,	MTLSPLLL	8	2	10	2	250	27.9	4.88	402.6	352.3	289	434.6	329	447.9	352.1	330.6
Q13508	Ecto-ADP-r	MKTGHFEI	5	2	58	2	389	43.9	6.06	3432.9	3392.8	2121.5	3256.2	3934.9	3277	2456.4	3963.6
Q8N4F0	BPI fold-co	MAWASRL	7	3	15	3	458	49.1	8.72	698.4	482	566.6	533.5	911.4	549	538.1	975
A1L4F5	Receptor t	MARGSALF	4	3	8	2	943	104.7	6.55	674	486.7	482.7	589.5	542.8	616.9	539.6	539.3
Q7Z5F5	Liver-expre	MWHLKLC	25	2	27	2	81	9.3	10.01	2169.4	2019.6	1508.1	2123.8	2118.7	2721.5	2031.8	2352.3
Q6MZK8	Putative ur	MSDLSVIG	1	2	10	1	2060	226.3	4.44	855.3	753.2	602.8	824.9	1052.1	815.5	884.4	1055.5
A0A0G2JN	Leukocyte	MEQPHDE	8	4	11	4	478	52.5	6.57	727.8	569.3	471.4	639.9	650.6	758.7	604.4	506.9
Q9UK41	Vacuolar p	MFHGIPAT	12	2	5	2	221	25.4	5.54	168.4	135.1	131.4	135.1	139.5	134.7	115.1	125.4
B1AKK2	Dimethylar	MAGLGHP	11	3	20	2	285	31.1	5.81	361.4	224.5	236.1	227.3	316.5	262.5	254.1	237.8

Q13201	Multimerir	MKGARLFV	2	2	33	2	1228	138	7.93	2564.2	2383.5	1835.3	2261.2	2741.7	3078.9	2514.8	3084.4
Q6PCB0	von Willeb	MLPWTAL(	9	3	9	3	445	46.8	7.68	686.2	405.7	301.9	381.8	389	484.1	312.9	360.7
Q15904	V-type pro	MMAAMA	5	2	17	2	470	52	6.14	417.9	529.1	297.8	374.5	464	308.2	356.3	385.4
H0Y8X4	2'-deoxynu	AAAMVPG	14	2	9	2	243	25.9	5.5	414.4	324	305.7	351.5	377.2	305	272.2	281.2
P07585	Decorin OS	MKATIILLLI	9	3	7	2	359	39.7	8.54	405	330.1	330.8	366	354.2	363.2	359.3	336.7
B3KPR2	cDNA FLJ3	MGLQTTK\	5	3	9	3	660	73.9	7.66	481.3	551	426.2	475.2	421.6	462.8	373	426.5
Q03405	Urokinase	MGHPPLLF	9	2	9	2	335	37	6.65	127.2	158.8	115.4	127.4	117	122	109.9	107.7
Q32Q12	Nucleoside	MVLLSTLG	22	4	5	4	292	32.6	8.48	543	451.4	400.9	497.6	535.8	501.1	491.2	380.3
Q9ULC0	Endomucir	MELLQVTII	5	1	5	1	261	27.4	7.94	808	759.5	456.2	869.5	842.1	601.8	867.5	772.7
O00322	Uroplakin-	MASAAAAI	15	3	9	3	258	28.9	5.33	488.1	477.2	370.2	501.9	484.3	500.6	421.2	506.4
P24593	Insulin-like	MVLLTAVL	14	3	8	3	272	30.6	8.21	542.3	491	409.2	491.8	463.7	516	480.8	422.2
B1B0D4	ADAMTS-li	MLFEVGEG	4	4	8	4	1060	116.2	6.76	116.7	99.4	82.5	111.7	111.1	113	105.9	111.6
Q13308	Inactive tyı	MGAARGS	3	3	8	3	1070	118.3	7.09	280.4	218.1	203.3	250	240.6	192.5	222.6	238
O43570	Carbonic a	MPRRSLHA	13	3	6	3	354	39.4	7.23	13.3	11.4	8.7	13.7	16.3	12.6	10.4	12.2
A0A0X9UW	IBM-A3 hea	EVQLVQSG	20	2	5	2	127	14.4	8.37	93.6	64.4	53	61	55.5	64.3	52	45
Q9NQ38	Serine prot	MKIATVSV	6	4	20	4	1064	120.6	8.06	1745.1	2618.2	1196.8	2027.1	2058.5	1807.5	1876.7	2121.7
Q9NY25	C-type lect	MNWHMII	17	3	7	3	188	21.5	8.81	351.3	284.6	275	294.5	332.6	334.2	329	366.3
Q15223	Nectin-1 O	MARMGLA	6	3	7	3	517	57.1	6.1	923.9	798	666.1	1008.6	951.5	945.8	900.1	980.7
Q53RD9	Fibulin-7 O	MVPSSPRA	5	2	15	2	439	47.3	7.62	669.2	772.3	512.5	663.2	655.9	708.5	603.7	722
Q16706	Alpha-man	MKLSRQFT	4	4	12	4	1144	131.1	7.58	313.3	279.2	211.3	288.8	318.6	301.1	276	283.9
P48551	Interferon	MLLSQNAF	3	2	13	2	515	57.7	4.5	969.7	966.2	532.1	832.4	832.9	938.9	1155.2	1228.8
A0A068LK(	Ig heavy ch	QLQLQESG	13	1	6	1	120	13.2	9.07	223.1	168	183.1	214.1	275.5	194.4	225.9	241.8
Q14314	Fibroleukir	MKLANWY	8	4	7	4	439	50.2	7.39	907	602.9	714.1	759	756.1	883.9	611.3	676.9
P36405	ADP-ribosy	MGLLSILRK	13	2	9	2	182	20.4	7.24	89.8	76.7	74.6	82.7	84.3	76.4	73.4	74.8
O15240	Neurosecr	MKALRLSA	6	3	11	3	615	67.2	4.78	1246.6	1077.7	1069.5	1201.3	1129.4	1204.7	944.5	1220.8
J3KS40	Secreted a	MQTCPLAF	19	1	8	1	85	9	5.06	105.1	109.4	88.8	105.4	121.7	113.9	86.2	105.4
A0A024R6I	Delta-like 1	MTATEALL	11	3	41	3	383	41.2	5.53	5585.3	4701.3	3555.6	5313.7	5481.2	4643.6	4609.3	7066.9
Q05DB4	HEBP2 pro	MAEPLQPD	12	2	5	2	214	24	5.38	166.8	129.9	121.3	133	147.9	146.2	137.4	131.4
B2R9S4	cDNA, FLJ9	MYTAIPQS	9	3	4	3	348	38.5	6.37	429.5	374.1	304.8	409.4	418.8	414.3	386.5	345.9
Q9UNF0	Protein kin	MSVTYDDS	9	3	9	3	486	55.7	5.2	113.8	88.1	82	86.2	96.6	86.8	88.5	87.7
Q92626	Peroxidasir	MAKRSRGI	2	3	8	3	1479	165.2	7.17	502.6	374.6	450.9	519.3	480.2	484.8	411.6	473.6
A7U7M2	Prion prote	MRKHLSW	21	4	12	4	176	20.3	9.03	491.1	387.7	394.6	557.2	463.6	502.4	445.8	541.8
A0A024R3	Ras homolo	MAAIRKKL	20	3	9	3	193	21.8	6.1	241.7	211.6	151.2	216.2	200.1	211.9	171.5	180.3
A0A140VJH	Testicular t	MGGKQRD	3	2	31	2	710	79.2	8.59	1606.4	1615.9	1322.8	1825.1	2007.2	1269.3	1273.9	1629
B4DNE1	cDNA FLJ5	MAAALFVL	10	4	14	4	385	42.2	5.66	1232.1	1051.4	965.8	1151.8	1223.4	1385.8	1101.1	1315.6
P40199	Carcinoem	MGPPSAPF	10	2	12	2	344	37.2	5.82	349.8	353	250.2	367	327.1	365.5	316.6	325
Q00796	Sorbitol de	MAAAAKPI	15	5	11	5	357	38.3	7.97	686.6	565.3	402	526.4	544.3	482.9	356.4	446.2
Q16610	Extracellula	MGTTARA	10	4	5	4	540	60.6	6.71	138.6	99.2	111.4	123.6	109.2	115.1	108.8	111.2
A0A193CH	10E8 heavy	EVRLVESG(	18	2	15	1	131	14.6	4.84	323	277.2	277.4	324.6	344.5	356.2	334.3	299.2
B2R597	cDNA, FLJ9	MNVLLGSV	14	2	78	2	114	12.8	5.5	9325.5	9386.4	7418.6	9548.3	9352.6	2737.9	4333.9	12181.9
P20151	Kallikrein-2	MWDLVLSI	8	2	45	1	261	28.7	6.92								

Q9Y376	Calcium-bi	MPFPFGKS	12	4	7	4	341	39.8	6.89	241	199.4	144.7	218.1	224.5	205.7	147.1	197
A0A140VJK	Testicular t	MAQKEGG	5	2	8	2	417	45	7.62	169.6	136.7	123.7	148.1	149.7	139.7	130.9	138.2
Q0ZCH6	Immunglob	LVQLVESG	15	2	7	2	131	14.3	6.55	587.5	508.3	538.1	771.4	534.1	589.1	544.5	522
Q9BXN2	C-type lect	MEYHPDLE	9	2	13	2	247	27.6	5.36	416.2	382.7	326.9	393.8	385	526.4	402.3	420.9
Q8TCD5	5'(3')-deox	MARSVRVL	17	3	9	3	201	23.4	6.64	586.3	496.2	436	541.1	570.5	585.2	467.6	528.8
B4DMS4	cDNA FLJ5	MGKLSRCS	2	2	4	2	812	88.4	4.4	558.8	527	395.8	590.7	608.2	594.4	495.7	369.1
Q9NP79	Vacuolar p	MAALAPLP	18	3	6	3	307	33.9	6.29	46.8	30.5	34.5	36.9	42.8	39.2	46.4	37.1
Q9UGM3	Deleted in	MGISTVILE	7	2	4	2	2413	260.6	5.44	195.1	201.8	178.1	171.8	160.3	202.5	200.3	214.2
A8K0T9	cDNA FLJ7	MADFDDR	17	3	6	2	286	32.9	5.69	370.4	374.5	216.2	349	362.7	369.4	279.8	238.9
P23468	Receptor-t	MVHVARLI	2	3	3	2	1912	214.6	6.57	54.4	39.6	39.9	32.9	42.9	82.3	31	48.4
P24592	Insulin-like	MTPHRLLP	10	2	7	2	240	25.3	7.81	865.4	908.8	760.7	865	812.3	844.8	829.2	785
P20827	Ephrin-A1 (	MEFLWAPI	13	3	16	3	205	23.8	6.83	1424.5	1376.3	976.8	1436	1513.2	1470.8	1186.4	1456.4
Q9UIU0	Dihydropyr	MAAGCLLA	5	5	8	5	1110	125.2	5.36	442.2	448.9	434.8	458.6	534.3	450.8	378.3	439
P01210	Proenkeph	MARFLTLC	9	2	10	2	267	30.8	5.53	888.3	775	1029.9	853.9	785.3	764.7	703.1	752.9
P06703	Protein S1(	MACPLDQ	24	3	32	3	90	10.2	5.48	2621.2	2536.2	1732.2	2589.1	3375.7	3079.8	3340.9	2629.1
A0A024RB(	Tetraspani	MAVEGGM	4	1	24	1	238	25.6	7.81	1531.3	1467.4	1195.2	1469.9	1382.3	1209.3	1181.8	1265.1
Q9H0B8	Cysteine-ri	MSCVLGG	9	4	8	3	497	55.9	8.02	627.2	623.6	559.7	583.5	584.2	467.9	479.4	601.1
P08729	Keratin, ty	MSIHFSSP	9	5	14	2	469	51.4	5.48	137.6	73.7	60.5	69.4	74.3	109.6	151.1	78.3
P25815	Protein S1(	MTELETAM	24	2	14	2	95	10.4	4.88	1048.1	841.3	548.2	869.6	1204.9	1073.2	1676.1	900.9
P14625	Endoplasm	MRALWVL	5	3	6	2	803	92.4	4.84								
A0A024R1	Glycerol-3-	MASKKVCI	9	3	10	3	349	37.5	6.18	871.9	478.6	510.2	500.7	553.8	512.5	409.5	437.7
B1PS43	Myosin hea	MAQKGQL	1	2	7	2	2029	234.1	5.8	277.4	174.6	171.4	191.6	185.1	211.5	207.8	156.6
A0A087WY	Seizure 6-li	MGTPRAQ	4	3	7	3	923	99.1	4.87	178	98.7	99.9	105.3	110.5	109.3	104.6	107.6
P01709	Immunogl(	MAWALLLI	7	1	27	1	118	12.4	5.91	1762.5	2050.9	1622.8	1830.7	1804.7	1761.2	1809	1720.5
B2R983	cDNA, FLJ9	MSGESARS	15	3	9	3	241	27.5	6.6	497.6	350.4	404.1	417.9	450	461.9	372.9	356.1
A0A1C3PH	MHC class	MRVMAPR	10	3	4	2	366	40.6	6.2								
A0A024RD	Scavenger	MGRCCFYT	4	2	4	2	478	54.3	5.14	268.1	209.6	200.4	251.5	248.5	257	241.1	254.2
P13640	Metallothi(	MDPNCSC	32	2	4	1	62	6.1	7.96	642.5	672.2	426.7	749.1	744.3	866.3	768.9	559.1
B4DJQ5	cDNA FLJ5	MLLPLLLLL	7	3	10	3	535	60.1	4.42	503.5	471.9	357	544.3	507.3	479.7	412	436.2
Q16661	Guanylate	MGCRAAS	12	1	9	1	112	12.1	6.48	77.1	255.4	55.6	74.8	74.3	83	64.4	58.1
Q96C23	Aldose 1-e	MASVTRA	11	3	9	3	342	37.7	6.65	40.1	37.1	34.5	45.4	42.7	51.1	39.9	33.6
J3KNB4	Cathelicidi	MGTMKTC	18	3	7	3	173	19.6	9.41	824.5	776.3	687.1	854.3	778.5	945.5	955.3	767.1
Q86UD1	Out at first	MRLPGVPL	11	3	14	3	273	30.7	6.84	741.8	579.9	648.7	639.2	774.1	816.5	652.4	662.9
J3KPA1	Cysteine-ri	MKQILHPA	13	2	12	2	276	31	7.61	846.6	678.2	686.2	815.2	1100.2	768.3	908.6	1153.4
A0A1C9J6(	B cell rece	DIRMTQAF	11	1	8	1	107	11.7	7.99	562	568.8	540.6	476.3	525	524.9	483.8	475.4
G3XAI2	Laminin su	MDCPLSAP	3	3	6	3	1810	200.3	4.96	451.9	413.4	392.9	399.7	394.8	492.2	361	400.8
Q9H6B4	CXADR-like	MSLLLLLLL	7	3	14	3	373	41.3	7.99	885.2	1107.5	697.3	940.9	968.6	931.8	1076.6	1006.1
M0R1T5	Charged m	XEKKIIADIK	11	3	9	3	227	24.9	5.27	980.7	828.5	748.1	1024	1025.2	991.9	771.8	898.2
B2RD36	cDNA, FLJ9	MANEAYP(	15	4	17	4	245	28	6.79	1492.3	900.7	954.5	1036.2	1323.4	1136.8	869.9	855.7
A8K2T6	cDNA FLJ7(	MERRWPL	3	3	8	3	976	108	6.64	703.3	624	587.6	715.9	667.7	722.9	635.5	665.8
V9HW90	Epididymis	MALLPRAL	5	2	11	2	522	56.2	8.5	295.1	211.7	192.2	232.4	238.6	224.1	218.7	185.3

A2N4P8	IL2R protei	ELCDDDPP	11	2	7	2	251	28.4	6.52	253.3	187.2	203.7	229	207.5	218.4	244	217.5
A2NW97	Rheumatoi	MDWTWR	13	2	8	1	134	14.9	8.85	438.5	385.4	497.1	397.1	387.4	383.6	441.3	395.2
H7C2F2	CD99 antig	RRWGRPA	8	1	10	1	201	20.6	5.27	154.8	175.9	131.9	149.6	136.9	168.1	129.4	165.1
V9GYM3	Apolipopro	MCEQHPKI	14	2	10	2	133	14.9	8.27	521.7	362.2	408.4	414.5	356.9	635.1	438.5	315.6
B2R7Z2	cDNA, FLJ9	MPRQLSAA	5	2	3	2	416	44.3	8.41	183.6	453.3	131	307.3	240.1	294.5	324.2	286.3
P12724	Eosinophil	MVPKLFTS	17	2	5	2	160	18.4	10.02	120.6	114.8	119.5	103	122.3	103.4	136.5	77.9
A0A0A0MC	Laminin su	MALSSAWI	2	3	7	3	1823	202.4	6.25	576.6	592.8	437.2	740.9	557	567.9	490.8	554.8
O00526	Uroplakin-1	MAPLLPIRT	16	2	4	2	184	19.4	10.4	148	145	112	145.4	154.1	151.4	111.1	169.9
A0A0C4DH	Immunoglo	MRLLAQLL	26	2	11	2	120	13.1	8.53	330.6	334	352.9	300.3	293.5	359.9	314.8	320.2
A0N7J6	REV25-2 (F	METPAQLL	22	2	52	2	134	14.5	6.57	2349.9	3333.3	1832.2	2334.3	2874	2715.3	3016.2	2623.9
Q8N3J6	Cell adhesi	MIWKRSAV	8	2	5	2	435	47.5	5.33	220.8	222.4	135.6	198.5	202.8	203.5	193.6	226.2
P27482	Calmodulin	MADQLTEE	17	2	8	1	149	16.9	4.42	84.2	86.7	68.6	92.8	81.6	95.7	76.1	76.5
P04732	Metallothi	MDPNCSCA	33	2	3	1	61	6	7.96	254	251.7	158	264	223.3	246.3	267.3	191.4
Q53FE8	cDNA FLJ36	MAAERQEA	3	1	24	1	370	40.5	5.14	1238.4	960.9	1061.7	1097.3	1221.9	1089.9	1157.5	1131.9
B2RA03	cDNA, FLJ9	MSFTTRSTI	7	3	20	1	430	48	5.38	55.1	40.8	37.9	44.4	46.9	49.8	84.5	40.2
O94772	Lymphocyt	MLPAAMK	20	3	7	3	140	14.7	7.25	610.4	724.7	443.8	673.8	688.9	882.3	634.8	647.1
C3VMY8	Alpha B cry	MDIAIHHP	17	3	4	3	175	20.1	7.08	670.4	480.8	514.7	530.1	657.6	546.5	531.7	449.9
A0A024RD	3-hydroxyb	MGRLDGKV	9	2	5	2	245	26.7	7.65	124.4	92.5	119.7	93.9	95.3	90.5	79.4	86.1
A0A075B6I	Immunoglo	MAWTPLFI	15	2	18	2	117	12.5	7.2	1561.4	2106.3	1329.5	1787	1749.1	2090.8	2051.3	1686
Q969H8	Myeloid-de	MAAPSGGV	18	3	15	3	173	18.8	6.68	598.6	813.4	547	611.4	608.5	600.9	695.2	628.9
A0A024RD	Tumor nec	MALKVLLE	7	2	2	2	423	46	5.47	241.4	262.3	209.8	237.7	239.2	242.4	204.1	233.8
P81605	Dermcidin	MRFMTLLF	10	1	10	1	110	11.3	6.54	618	1243.9	520.9	708.1	690.7	658.5	818.3	563.4
Q02413	Desmoglei	MDWSFFRY	5	3	5	3	1049	113.7	5.03	138.6	120.1	132.3	120.5	118.2	122.6	112	117.5
Q9Y6Q6	Tumor nec	MAPRARRI	3	1	6	1	616	66	5.34	169.1	162.6	104.8	158.9	145.6	149.7	134.6	139.8
P15291	Beta-1,4-ga	MRLREPLLS	5	2	18	2	398	43.9	8.65	783	466.4	567	648.5	951	997.2	484.1	589.3
A8K2U0	Alpha-2-ma	MWAQLLLC	3	4	5	4	1454	161	5.73	366.3	309.3	284.2	315.2	295.3	282.6	361.2	313.5
Q9HC38	Glyoxalase	MAARRALH	6	2	9	2	313	34.8	5.6	498.6	388.2	319	357.1	408	360.4	321.7	331.6
B1AH90	Signal pept	MGAAAVR	9	2	5	2	347	37.3	5.71	100.6	170.1	79.4	116.6	101.8	107	116	118.8
C0JYY2	Apolipopro	MDPPRPAL	1	3	5	3	4563	515.2	7.05	99.2	64	64.4	61.9	50.3	95.9	49.6	40.5
Q53SS8	Epididymis	MDAGVTES	8	2	4	2	356	37.5	7.09	62.8	59.6	37.2	56.6	63.6	56.5	58.3	44.4
P07307	Asialoglyco	MAKDFQD	12	2	6	2	311	35.1	6.25	42.3	43	57.1	41.9	47.7	40	35.8	40.5
Q8NES3	Beta-1,3-N	MLKRCGRF	7	1	2	1	379	41.7	9.17	40.4	40.6	31.8	38.6	42	35.1	42.7	47.5
P19021	Peptidyl-gly	MAGRVPSL	3	3	6	3	973	108.3	6.42	98.6	88.9	75.9	92.7	100.1	129.3	83.3	96.2
Q9UEF7	Klotho OS=	MPASAPPF	6	4	4	4	1012	116.1	7.97	279.8	257.9	230.4	264.9	250	258.7	200.7	214.6
A8K865	cDNA FLJ75	MAPNIYLV	4	1	8	1	259	28.5	7.99	345.7	270.3	197.2	341.9	299.2	283.5	354	271.8
O75340	Programme	MAAYSYRP	6	1	5	1	191	21.9	5.4	189	153.1	173.9	182.4	212.6	186.2	181.1	205
P21266	Glutathion	MSCESSMV	13	3	11	3	225	26.5	5.54	66	50.1	39.2	48.3	72.2	63.9	48.8	61.6
A0A0B4J2D	Immunoglo	MDMRVPA	14	1	7	1	117	12.6	7.84	89.8	85.7	85.5	77.9	82.1	94.4	67.6	101.1
Q6IB39	RNASE6 pr	MVLCFPLLI	19	2	12	2	150	17.2	8.76	351.3	500.5	279.2	465.7	368.4	370.8	347.1	310.4
B2RCJ5	cDNA, FLJ9	MSSSSWLL	3	2	3	2	805	92.4	5.54	125	90.1	115.3	103.1	104.1	107.2	95	89
Q59F30	Fibroblast g	PCMWHQC	1	2	4	2	1034	112.1	7.88	36.9	33.4	27.5	27.7	31.3	38.8	37.8	33.7

C7FDU7	MHC class	SHSMRYFS	12	2	4	1	181	21.2	7.3								
A0A024RB	Vacuolar p	MAGAGSE	6	1	2	1	285	31.3	7.34								
H7C2N1	Prothymos	XSPGLRPP	7	1	8	1	148	15.8	4.23	569.2	522.1	314.9	576.9	527.6	654.5	549.3	488.7
Q495M3	Proton-cou	MSVTKSTE	5	2	9	2	483	53.2	8.12	221.4	214.9	152.7	203.2	194.1	232.3	167.2	204.8
Q04721	Neurogeni	MPALRPAL	1	2	4	2	2471	265.2	5.14	481.2	394.6	439	462.4	423.5	417.2	442.6	466.6
A0A087WT	Neuroblast	MCSLALQC	12	3	17	3	215	23.2	5.21	1931.4	2167	1555.1	2070.6	2151.1	1769.4	1990.9	2274.8
Q9NZU0	Leucine-ric	MISAAWSI	6	3	4	3	649	73	7.64	20	17.8	13.4	18.3	26	19.6	18.1	23.7
A0A0A0MT	Titin OS=H	(content is	0	4	5	4	35991	3992.2	6.39	181	143.9	131.7	172.5	184.8	256	195.2	154.4
E7ERL8	Neurexin-1	MGTALLQF	1	2	11	2	1507	165.3	5.96	1014.5	785.4	812.8	993.7	949.7	900.2	846.1	982.7
O96033	Molybdopt	MVPLCQVI	22	2	7	2	88	9.7	4.72	326.8	303.4	280.7	312.9	361.7	331.1	305.8	328.1
Q9NP85	Podocin OS	MERRARSS	8	3	5	3	383	42.2	8.75	101.3	102.8	92.9	95.8	99.4	113.6	80.7	105.2
P04430	Immunogl	MDMRVLA	14	1	5	1	117	12.6	8.16								
A0A140VK	Testis secr	MDPRKVN	8	3	7	3	369	41.3	5.27	306	265.6	193.6	267	278.8	245.7	218.2	201.6
Q59EA3	Cadherin 5	TDRQSNGT	4	3	3	3	807	89.9	5.48	27	43.2	24.1	36.3	37.2	26.3	45.4	31.7
B3KQT8	cDNA PSEC	MRGANAW	8	3	13	3	415	45.7	8.47	384.5	386.2	320.4	383.5	415.3	464.9	350.9	391.1
J3QQX2	Rho GDP-d	MAEQEPTA	13	2	14	2	235	25.8	7.44	418.3	384.4	291.8	383.7	406.8	341.3	324.2	379.3
Q13145	BMP and a	MDRHSSYI	15	2	11	2	260	29.1	7.75	721.2	716.1	499.1	686.7	651.3	736.3	548.2	609.5
P52209	6-phospho	MAQADIAL	7	3	7	3	483	53.1	7.23	65.6	44.4	28.9	75.7	59.6	63.6	66.1	38.5
E1U340	ZNF511/PF	MQVADVP	8	2	3	2	310	35.2	5.94	165.1	180.6	109.3	108.7	149.5	140.2	120.8	132.7
A1L3A3	Contactin 2	MGTATRRI	4	2	3	2	1040	113.4	7.83	100.3	77.4	95	74.3	85	89.4	76.8	90.3
Q6FHG5	Gamma-sy	MDVFKKGI	13	1	7	1	127	13.3	4.86	125.4	98.2	99.8	88.4	109.2	168.6	120	107.4
A8K4A5	cDNA FLJ7	MPSLLVLTI	5	3	9	3	541	59.8	6.27	453.2	345.1	446.9	402.3	466.4	436.6	293.7	364.1
Q59FB9	Toll interac	QLSPSQAP	4	1	3	1	352	38.9	8.46	40.8	33.5	34.7	27.8	34.3	43.9	27	32.7
Q9H6S3	Epidermal	MSQSGAVS	3	3	4	3	715	80.6	6.84	262.5	249.9	190.6	271.4	313.3	238.5	248.1	260.1
A0A0B4J1F	4-hydroxyp	MTTYSDKG	12	4	4	4	393	44.9	7.01	135.8	93.1	99.6	99.8	124.5	113.8	77.7	90
A0A024R3	RAB4A, me	MSQTAMS	10	2	10	1	218	24.4	6.07	28.9	39.9	24.3	33.8	36.2	32.5	26.2	32.2
P03973	Antileukop	MKSSGLFP	9	1	5	1	132	14.3	8.75	423.3	599.4	265.8	552.7	486.9	358.9	398.8	431.3
P51170	Amiloride-	MAPGEKIK	4	2	8	2	649	74.2	7.5	377.2	270.6	256.5	322.6	414.9	372	303	446.9
Q9H461	Frizzled-8	MEWGYLLI	3	2	9	2	694	73.3	8.19	660.3	701	481.4	692.9	680.7	536.9	744.7	671.3
Q9UIH2	Tumor nec	VAFTPYAPI	85	2	4	1	33	3.7	6.27								
Q8IX04	Ubiquitin-c	MEFDCEGL	6	3	5	3	471	52.2	7.09	196.9	171.8	165.5	201.4	198.6	192.3	143.1	171
E0D851	Platelet gly	MPLLLLLLL	3	2	9	2	665	72.8	6.09	303.1	480.4	241.3	306.7	289.2	319.5	387.8	339.9
Q08629	Testican-1	MPAIAVLA	9	3	10	3	439	49.1	6.1	178.7	123.5	137.4	123.8	178	244.7	161.4	171.3
Q6FHJ7	Secreted fr	MFLSILVAL	5	1	2	1	346	39.8	8.82								
M0R2A0	ER membra	MAAASAG	7	2	4	2	371	39	6.9	434.3	403.2	333.2	392.1	403.6	418	380.2	363.1
P41217	OX-2 mem	MERLVIRM	9	2	4	2	278	31.2	8.51	281.4	163.2	156	177.1	208.6	198.4	190.4	206.1
P04040	Catalase O	MADSRDP	9	4	4	4	527	59.7	7.39	208.2	118.8	176.6	136.9	122.1	150.9	142.6	121
Q5T9B7	Adenylate	MGCCSSSD	6	1	7	1	210	23.4	8.6	220.3	237.8	160.4	199.6	214.7	222.1	186	184.3
Q6DKI7	Transmem	MRTEAQVI	4	1	3	1	326	34.3	8.91	32.6	37.4	31.4	29.6	37.1	37	36.6	38
Q5TBU5	HCG17736	MASKGLQI	29	2	4	2	76	7.9	5.31	357.7	354.9	235.1	329.4	387.6	376.1	334.3	329.8
P05091	Aldehyde c	MLRAAARI	4	3	17	1	517	56.3	7.05	405.9	337.3	286.8	386.3	392	498	460.4	445.7

P30047	GTP cycloh	MPYLLISTC	40	2	23	2	84	9.7	6.54	271.7	211.3	400.7	226.7	227.2	255.3	256.1	235.6
H0YBZ2	HLA class I	XRSCREDQ	6	1	4	1	316	35.1	7.72	269.2	297.7	207.8	261.5	267.9	251	279.4	274.6
Q9NX12	cDNA FLJ2(	MVKVTFNS	15	3	5	3	266	30.3	5.21	22.1	17	19.4	16.7	17.3	18.2	20.1	15.3
P04275	von Willeb	MIPARFAG	1	3	8	3	2813	309.1	5.48	105	110	70.9	112.1	100.8	128.3	91.8	93.5
A4D0V4	Capping pr	MADLEEQL	9	2	5	1	286	32.9	5.85	38	24.1	19.3	27.7	28.4	25.3	23.7	20.4
A0A024R4/	Alkaline ph	MQGPWVL	3	2	4	2	528	56.8	5.86	95.8	84	42.8	64.5	71.3	54.8	77.3	60.2
A0A0B4J1Y	Immunoglo	MEFGLSW	15	2	10	1	119	13.2	7.85	866.3	781.2	845	956.7	917	770.6	938.3	818.1
A0A0C4DH	Protein IGH	MQFVLSW	9	1	4	1	116	12.8	9.25	329.9	264.4	234.3	265.1	232	232.8	197.9	198.3
A0A140VJF	Testicular t	MAAATGPS	3	1	3	1	493	54.5	6	62.9	44.8	45	42.9	48.2	48.5	44.2	45.8
P38606	V-type pro	MDFSKLPK	5	3	7	3	617	68.3	5.52	579.3	472.7	392.9	491.1	551.3	557.4	412.8	392.4
Q13443	Disintegrin	MGSGARFF	3	2	2	2	819	90.5	7.52	138.9	101.6	111.1	119.6	137.6	125.2	124.1	139.8
P03951	Coagulatio	MIFLYQVV	6	3	6	3	625	70.1	8.1	89.1	92.9	65.7	73	80.2	71.8	81	77.2
P13688	Carcinoem	MGHLSAPL	5	2	27	1	526	57.5	5.97	101.9	81.2	145	97.9	93.1	90.4	64.8	77.3
F1T0L0	LY6/PLAUF	MRGTRLAL	11	1	8	1	125	13.1	5.96	254.5	198.8	474.9	235	245.6	197.6	199.8	291.3
A0A0F7TA\	IGHV5-51 p	GAEVKKPG	22	2	9	2	110	12.1	8.38	341.1	267.2	284.3	304.6	319.3	352.1	334.6	298.3
Q68CJ9	Cyclic AMP	MNTDLAA(	7	2	39	2	461	49	5.15	1786.2	1292.7	1177.1	1312.2	1536.9	1710.6	1563.2	1983.8
P48740	Mannan-bi	MRWLLLYY	6	3	4	3	699	79.2	5.49	95.1	87	77.2	83.6	78.7	79.9	65.6	79.2
H0YMD1	Low-densit	MGPWGW	2	2	13	2	948	104.7	5.5	1161.4	1156.6	819.6	1033.7	1101.8	1176.6	994.7	1237.7
A0A140VJE	Guanylate	MPGPRRP/	2	2	4	2	1061	118.8	6.64	122.6	80.2	107.8	76.9	87.7	104.5	68.1	82.9
Q8N474	Secreted fr	MGIGRSEG	9	2	4	2	314	35.4	8.85	69.1	72.5	46.7	66.6	78.1	97.4	53.2	63.3
B2RDI5	cDNA, FLJ9	MSEEIITPV	4	2	3	2	714	81.8	5.54	177.4	176.8	133.6	178	171	153.7	156.2	146.3
A0A024R2(	Cysteine-ri	MAPWPPK	5	2	4	2	420	45.4	4.87	54.2	33.9	42.3	40.8	50.5	55.8	52.7	54.1
P01601	Immunoglo	MDMRVLA	14	1	3	1	117	12.7	7.74	36.6	46.7	35.4	38.1	42.3	46	41.2	45.5
P08246	Neutrophil	MTLGRRLA	6	2	11	2	267	28.5	9.35	698	435.3	307.3	476.7	467.3	783.7	579	343.6
Q9NP84	Tumor nec	MARGSLRF	8	1	9	1	129	13.9	8.95	783	972.6	539.2	805.6	815.5	1053.8	932.4	882.1
P61604	10 kDa hea	MAGQAFR	30	3	3	3	102	10.9	8.92	81.8	68.9	45.4	67	89.2	94.9	63.5	96.8
Q06828	Fibromodu	MQWTSLLI	5	2	5	2	376	43.2	6.04	329.9	449.2	272.6	305.2	322.7	329.6	269.3	345.6
Q86SQ8	Beta-defer	GNFLTGLG	21	1	13	1	47	5.1	8.73	304.5	470.9	245.3	347.5	277.7	309.6	583.1	268.5
A6XMV9	Protease s	MNLLLILTF	15	3	5	1	261	28	5.14	103.6	144	75.1	86.9	108.9	87	97.4	95.5
A8K669	cDNA FLJ7	MVWKVAV	4	1	3	1	433	49.4	6.55	48.8	35.1	69.4	29.6	27	40.1	34.2	30.4
P09529	Inhibin bet	MDGLPGR/	2	1	6	1	407	45.1	8.03	193.1	181.7	158.5	180.3	203	216.8	175.3	189.4
P29966	Myristoyla	MGAQFSK	5	1	4	1	332	31.5	4.45	286.4	232.1	274.9	281.1	333.6	319.7	267.5	270.4
A2J1M8	Rheumatoi	LVESGGGV	21	2	10	1	95	10.7	7.99								
A0A0K0K1I	Epididymis	MAMLRVC	13	3	8	3	249	28.7	6.02	527.2	321.9	331.9	393.6	454.7	466.8	389.6	331.6
B4DQD2	cDNA FLJ5.	MPLLTLYLL	7	2	3	2	305	33.8	5.88	162.5	163.7	120.1	147.7	148.9	178.4	182.9	141
P01706	Immunoglo	MAWALLLI	7	1	8	1	119	12.6	7.24	595.3	572.2	482.2	505.5	498.8	632.5	881.9	669.4
Q8NBS9	Thioredoxi	MPARPGRI	7	3	9	3	432	47.6	5.97	455	427.6	373.8	415.5	450.1	418.8	437.5	401.7
D3DRD5	Fibroblast g	MGLTSTW	1	1	7	1	838	93.8	6.04	443.1	377.3	337.9	395.2	419.3	410.9	350.7	462.5
Q8TCT8	Signal pept	MGPQRRLS	3	1	8	1	520	58.1	8.32	324.4	255.8	225.6	298.9	321.8	252.6	300.1	348.3
J3KQ18	D-dopachr	MQLFLLAV	23	3	7	3	132	14.2	7.3	368.8	226.4	211	209.5	294.9	265.2	220.2	220.3
Q53GD1	Guanine nu	MSSKTAST	22	1	3	1	72	8	8.97	123.9	102	115.1	94.5	103.4	99.5	98	93.5

A0A087WY	Glycoprote	MDYYRKYA	5	1	8	1	147	16.3	8.47	439.2	500.6	302.8	385.9	442.7	516.7	398	415.7
P14174	Macrophag	MPMFIVNT	8	1	30	1	115	12.5	7.88	2360.8	1882.7	1457.4	1941.2	2284.4	2061.6	1939.5	1538
P50897	Palmitoyl-p	MASPGCLV	5	1	5	1	306	34.2	6.52	178.1	142.9	153.5	153.4	175.5	155.9	136.5	155.9
A0A024R8	L-selectin (	MGCRRTRE	7	2	5	2	385	43.6	7.15	249.1	228.8	238.7	271.7	235.9	240.4	289.1	313
Q7L0X0	TLR4 intera	MEAARALF	1	1	4	1	811	88.7	9.6	187.3	173.1	115.2	164.8	187.9	186.2	147.4	179.7
Q53H93	Diphtheria	MKLLPSVV	5	1	5	1	208	23.1	9.38								
P05387	60S acidic r	MRYVASYL	10	1	7	1	115	11.7	4.54								
A0A0J9YX3	Uncharacte	MEFWLSW	19	2	6	2	117	12.8	7.85	259.7	205.1	225.1	222.3	237.7	210.6	210.6	201.9
J3QL71	Secernin-2	MERPASPC	4	1	1	1	433	47.5	5.68	64.9	53	89.4	62.7	69	68.1	62.9	68.1
P30039	Phenazine	MKLPIFIAD	10	2	5	2	288	31.8	6.52	187.9	134.4	286.2	158.6	167.2	157.2	147.8	152.6
A2NH53	Immunogo	THGEIVMT	19	1	1	1	112	12.2	8.51	71	95	94.4	82.9	84.2	76.1	72.6	76.9
P83110	Serine prot	MQARALLL	5	2	4	2	453	48.6	7.09	68.8	85.8	54.8	74	81.9	67.3	71.2	63.3
Q9UN73	Protocadh	MVFTPEDF	3	2	2	2	950	102.7	5.06	31.1	31.4	29.3	36.9	43.9	29.8	37	40.4
O75223	Gamma-glu	MANSGCKI	12	2	8	2	188	21	5.14	224.9	183.1	139.5	198.5	196.5	192.5	198.6	195.6
Q86V85	Integral me	MGGLRLLA	2	1	5	1	440	49.4	7.39	176.3	212.7	185.7	190.2	191.9	186	184.9	196.6
Q9UBX1	Cathepsin	MAPWLQL	4	2	6	2	484	53.3	8.22	122.2	112.4	74.8	106.5	117.2	117	107.9	111.4
P54727	UV excision	MQVTLKTL	2	1	2	1	409	43.1	4.84	245.5	263.1	184.6	226.1	247.5	243.2	225.8	221.8
A0A0U1RR	Histone H2	MSGRGKQ	14	3	13	3	170	18.5	11.52	1265.2	1004.6	657	736.5	768.7	1312.5	1501.5	601.7
A0A024RA	Arylsulfata	MGPRGAA	3	2	5	2	533	59.6	8.21	348.4	208.9	177.6	252.2	249	377.4	280.4	236.9
C6SUN5	Agouti rela	MLTAAVLS	12	1	8	1	132	14.4	7.44	57.6	51.7	45.4	57.9	59.5	56.1	44.4	40.8
P01258	Calcitonin	MGFQKFSP	13	2	2	2	141	15.5	6.52	337.3	256.4	244.8	394.7	377.9	484.4	337.8	403.3
A8K9M5	cDNA FLJ7	MKNSRTW	4	2	5	2	591	67	8.18	266.1	179	218.4	235.1	237.7	231.4	297.9	227.6
C9JJE7	Mucin-20 (	MGCLWGL	4	2	5	2	723	73.1	4.94	231.2	183.3	172.6	174.9	230.8	225.1	214	232.7
B2R880	cDNA, FLJ9	MANCEFSP	4	1	3	1	300	34.3	7.47	59.7	50.9	44.9	37.9	43.2	25.8	28.1	44.1
B4E3D4	cDNA FLJ5	MILPEALYL	1	1	3	1	607	68	6.19	247.5	237.5	207.8	288.2	251.5	268.3	235.3	227.7
B4E0V9	cDNA FLJ6	MSENTKET	6	1	5	1	246	27.7	8.25	321.7	188.6	267.3	234.5	234.7	313.8	269.9	247.7
A0A024RA	Bridging in	MAEMGSK	5	2	3	2	593	64.7	5.06	102.2	99.4	80.5	99.3	100.3	93.2	104.4	97.9
P29401	Transketola	MESYHKPD	5	2	3	2	623	67.8	7.66	169.1	153.2	86.8	120.2	135.8	144.1	143	98.4
A0A0K0K1	Proteasom	MSYDRAIT	9	2	3	2	248	27.9	8.46	193.9	139.1	136.1	143.2	168	156.9	135.8	153.3
A8K2N0	cDNA FLJ7	MWCIVLFS	4	2	7	2	688	76.6	4.98	248.6	174.1	212.7	218.5	219.3	215.7	198.7	191.2
P16402	Histone H1	MSETAPLA	5	1	4	1	221	22.3	11.02	425.3	325.7	311.8	319.2	351.6	338.8	357.1	316
Q92484	Acid sphing	MALVRALV	5	2	3	2	453	51.2	6.33	64.5	69.1	58.4	76.4	59.9	50.5	56.8	52
Q4G0I0	Protein CC	MNRVLCAF	17	2	2	2	132	15	7.02	46.9	48.4	40	40.9	38.6	52.8	42	42
B4DHQ3	Phosphose	MGRRLGA	5	2	7	2	415	45.3	9.03	68.4	66.7	45.6	56.6	71.6	78	38.4	55.9
A0A0F7RQ	Luteinizing	MEMLQGL	11	1	6	1	141	15.3	7.74	203.3	177.7	116.7	157.6	162.8	197.7	141.3	148.7
Q9H1C7	Cysteine-ri	MNQENPP	10	1	2	1	97	10.6	4.32	66.4	79.3	49.4	76.9	75.7	72.3	81.3	70
Q02388	Collagen al	MTLRLLVA	0	1	4	1	2944	295	6.27	200	209.7	150.4	208.5	191.1	184.6	152.2	159.4
A0A0A1HA	H.sapiens r	MAYAYLFK	14	2	5	2	212	23.6	6.54	208.1	93.3	122.1	95.6	100.7	107.1	91	92.1
Q6E0U4	Dermokine	MKFQGPLA	4	2	5	2	476	47.1	7.28	434.7	622	343	499.8	432.9	610.5	569.4	502.4
P30626	Sorcin OS=	MAYPGHP	17	3	7	3	198	21.7	5.59	208.4	190.1	185.6	186.5	193.4	193.2	175.9	172.9
B4DMN1	cDNA FLJ6	MAQRQGG	9	2	2	2	261	28.8	8.38	101.6	87.7	83.3	97	102.7	99.1	92.1	97.6

A0A024R8	Ubiquitin-r	MAAPLSVE	13	1	8	1	101	11.4	4.67	94.2	79.6	89.2	85.5	91.8	83.7	80.4	75.6
P24298	Alanine am	MASSTGDF	4	2	2	2	496	54.6	7.18	107.8	66.1	60.3	75.7	80.6	75	58	57.6
V9HW88	Calreticulin	MLLSVPLLL	10	4	6	4	417	48.1	4.44	81.1	58.9	57.1	59.9	74.5	70.8	75.2	75
Q6UY14	ADAMTS-li	MENWTGR	2	2	3	2	1074	116.5	8.34	196.7	182.4	159.1	186.5	216.3	223.5	230.7	255.7
P07360	Compleme	MLPPGTAT	7	1	1	1	202	22.3	8.31	56.8	57.3	47.2	45.5	54.2	59.1	41.9	45.6
E7EMK3	Flotillin-2 (	MGNCHTV	5	2	3	2	483	53.1	5.24	47.2	32.1	29.1	33	27.2	29.6	23.8	27.6
P49773	Histidine tr	MADEIAKA	21	2	6	2	126	13.8	6.95	303.5	250.6	213.6	265	317.5	283.1	231.7	222.4
Q6PK18	2-oxogluta	MAPQRRA	10	3	4	3	319	35.6	8.18	22.6	16.8	10.6	16.8	17.5	22.5	17.3	16.9
B2R6U9	Delta-like p	MRSPRTRG	3	3	5	3	1218	133.8	6.06	760.5	673	681.9	773.3	799	730.2	685.2	856.5
O94985	Calsyntenin	MLRRPAPA	2	2	5	2	981	109.7	4.91	369.1	348.3	362.2	317.9	351.9	320.1	299.6	316.3
O95196	Chondroiti	MGRAGGG	2	1	2	1	566	60	4.5	97.4	99.4	71.7	97.3	87.8	100.5	91	103.8
B4DJ06	cDNA FLJ5(	MTAEGPSF	3	2	6	2	629	67.9	4.91	625	596.7	514.3	607.2	615.8	661.2	602.6	626.6
Q9UM47	Neurogenic	MGPGARG	1	1	1	1	2321	243.5	5.39	77.9	56.7	56.7	63.1	64.2	55.7	59.5	65.6
Q9UL82	Myosin-rea	SYELTQPSS	17	2	15	2	107	11.4	6.6	743.1	1333.8	663.3	916.6	800	1182.3	1116.4	916.9
Q12929	Epidermal	MNGHISNH	4	3	4	3	822	91.8	7.5	155.1	115.3	95.4	125	132.6	137.4	107	99.3
Q86VB7	Scavenger	MSKLRMVI	2	2	5	2	1156	125.4	5.95	412.1	419	271.5	341.3	329.9	317.9	385.1	313
A0A024RC	Desmocolli	MAAAGPR	2	1	3	1	896	99.9	6.1	188.6	189.1	144.7	204	213.5	188.5	171.5	207.8
A8K144	cDNA FLJ7(	MAQLFLPL	1	1	7	1	911	99.1	4.68	930.9	848.6	1135.9	842.5	861.5	991.5	634.4	882.3
A0A125U0	GCT-A2 hea	EVQLVESG	15	1	18	1	126	13.8	5.45	140.8	103.1	176.5	110.8	113.3	132	112.7	115.8
A0A024RC	CD99 antig	MVAWRSA	6	1	4	1	262	28	5.14	161.6	144.2	84.6	120.6	104.1	107.1	112	94.6
O15144	Actin-relat	MILLEVNN	8	2	2	2	300	34.3	7.36	96.8	118.4	58.1	97.7	91.1	119.6	70.8	87
Q6UWI4	Protein shi	MWGARRS	5	1	3	1	295	31.4	7.68	202.1	167.2	173.4	150.2	163	165.9	158.3	160.1
Q9UL75	Myosin-rea	QVQLQQS(	13	2	22	1	122	13.7	8.46	204	157.4	158.5	196.9	212.6	186.8	210.6	216.4
Q6UXB4	C-type lect	MDTTRYSK	5	2	35	2	293	32.5	6.61	2538.5	2606.1	2702.7	2706.2	2743.9	2656.2	2493.7	2672
P06734	Low affinit	MEEGQYSE	9	3	5	3	321	36.4	5.57	267.8	227.4	167.2	234.7	236.7	250.8	192.1	203.8
P99999	Cytochrom	MGDVEKG	18	2	6	2	105	11.7	9.57	201.5	149.1	130.2	175.2	218.2	205	198.7	193.2
Q59EB6	Compleme	KLLSPLGFS	3	2	18	1	671	70.4	5.44	109	111.3	89.9	107.9	112.1	108.5	107.6	115.4
A0A087WY	Proline-ricl	MWRGIDSI	10	1	4	1	147	16.9	7.52	479.1	437.1	306.2	521.4	446.1	479.8	579.7	379.1
P55786	Puromycin	MWLAAAA	4	3	3	3	919	103.2	5.72	36.4	23	20.8	29.7	24.1	32.9	19.7	23.7
P08253	72 kDa typ	MEALMAR	2	1	4	1	660	73.8	5.47	450.4	374.4	319.4	455.1	467.9	427.8	440.5	397.7
A0A0X9US	MS-A6 hea	QVQLVQS(	16	2	2	1	118	12.7	8.44	112.7	93.7	128	95.1	93.6	99.8	83.3	89
P05026	Sodium/pc	MARGKAKI	9	2	8	2	303	35	8.53	356.5	377.7	234.5	333.6	337	348.8	260.5	330.7
A0A0C4DH	Protein IGH	MEFGLSWV	9	1	7	1	117	12.8	7.88	675.8	478.8	693.6	631.1	625.7	519.5	629.1	655.5
P49908	Selenoprot	MWRSLGL/	4	2	12	2	381	43.2	7.87	877.6	810.3	674.7	816.2	890.8	881.1	832.8	1007.4
A0A0A0MT	Protein IGK	LTFGGGTKV	67	1	6	1	12	1.2	8.84	890.9	947.2	637.4	760	1080	920.6	710.9	836.2
Q14210	Lymphocyt	MRTALLLL/	7	1	5	1	128	13.3	8.21	892.2	1131.4	695.9	1037.5	1030.1	1101.4	710.4	834.8
Q59ER5	WD repeat	RAGPGGSR	2	1	2	1	624	68.1	7.23	103.8	59.3	55.6	64.5	74.1	90.3	65.6	59.9
B3KTG0	cDNA FLJ38	MCSTMSAI	4	1	2	1	342	36.9	5.85	41.2	32.3	46.7	36.6	44.9	47	32.5	36.2
A2N2F4	VK3 protei	LTQSPGTLS	8	1	15	1	108	11.9	9.51	254.5	491.3	198.9	269.7	305.8	289.6	279.5	285.9
A0A193CH	10E8 light (	SSELTQDP/	17	2	5	2	109	11.6	7.96	372.6	422.7	502.2	415.8	365.1	544.3	476.1	444
Q9Y4L1	Hypoxia up	MADKVRR(	2	1	2	1	999	111.3	5.22	22.8	15.6	24.1	15.4	20.8	19.8	16.2	20.5

P27930	Interleukin	MLRLYVLV	5	2	2	2	398	45.4	7.9								
A8K6C9	cDNA FLJ7	MGIPMGK	9	2	8	2	180	20.2	9.32	240.9	365.4	149	244.8	221.6	240.3	301	272.2
A0A140VK	Transaldol	MSSSPVKR	6	2	5	2	337	37.5	6.81	292.6	233.8	171.3	269.6	253	318.9	364.6	211
P37802	Transgelin-	MANRGPA	19	3	5	3	199	22.4	8.25	141.4	104.2	169.7	130.8	138.6	125.8	143.9	116.5
Q9UQN3	Charged m	MASLFKKK	13	3	6	3	213	23.9	8.76	234.2	189.7	168.8	214.4	206.1	235.6	156.6	188.2
B2R4F3	cDNA, FLJ9	MTEKAPEP	6	1	2	1	201	22.9	5.3	168.2	99.1	95.3	132.3	118.3	126.8	143.1	117.3
J3QRS3	Myosin reg	MDLTTTM	12	2	2	2	177	20.4	4.75	42.5	39.1	23.9	31	30.9	40.9	39.6	35.4
Q96QS0	Putative m	MDWTWR	11	2	3	1	159	17.5	9.29	120.8	106.1	111.5	106.1	125	105.8	131	126.5
P18428	Lipopolysa	MGALARA	5	2	9	2	481	53.4	6.7	352	289.9	263	288.8	340.7	328	428.6	258
P98161	Polycystin-	MPPAAPA	0	1	4	1	4303	462.2	6.73	180.8	161.5	151.3	154.8	161.3	156.1	133.4	161.1
Q14112	Nidogen-2	MEGDRVA	2	2	3	2	1375	151.2	5.29	136.9	111	115.9	117.9	105	110.9	102	113.6
A0A1B1CY	Vitamin D	LAERLKAKL	32	1	8	1	34	3.7	8.56	504.5	309.5	867.1	484.5	320.7	325.4	236.3	346.4
I3L504	Eukaryotic	MADDLDF	6	1	7	1	186	20.5	5.25	246.8	234.1	145.1	217.5	209.8	214.1	171.6	170.1
Q53HU8	Vimentin v	MSTRSVSS	6	3	4	2	466	53.7	5.12	52.8	36.1	30.6	31.6	31.1	40.4	39.4	35.3
Q8TBP5	Membrane	MKASQCC	5	1	3	1	190	19.9	6.33	147.6	111	106.1	153.3	148.7	138.3	127.4	157.3
B2R6W4	cDNA, FLJ9	MVCGSPG	3	1	5	1	325	36.2	8.37	389.3	514.4	297.3	432.2	419.4	394.1	333.2	394.5
V6A6E5	MHC class	MRVMAPR	4	1	2	1	366	40.9	6.3	74.1	88.9	64.2	84.8	76.4	82.1	65.1	78.4
P82980	Retinol-bin	MPPNLTGY	7	1	9	1	135	15.9	6.54	757.5	632	453.8	676.4	864.8	746.1	595.4	701.1
A0A0X9T7	MS-A6 ligh	DIQLTQSP	15	1	5	1	107	11.4	8.47	72.9	60.7	55.9	58.5	52.7	68.7	54.3	54.3
F8RHH9	MHC class	SHSMRYFS	13	2	3	1	181	21.2	7.3								
Q14117	Dihydropy	MAAPSRLL	3	2	6	2	519	56.6	7.27	201.8	141.6	111	138.3	209.1	172.8	124.8	130.9
P19256	Lymphocyt	MVAGSDA	4	1	11	1	250	28.1	6.76	776.7	626.6	595.5	697.1	721.6	753.2	653.8	773.2
Q86XT2	Vacuolar p	MYRARAA	10	2	4	2	251	27.7	9.01	58.7	62.4	53.8	65.6	69.1	61.2	66	66
D2IYL2	Corneodes	MGSSRAP	7	3	3	3	529	51.5	8.35	520.3	544.5	442.9	475.2	444.8	430.4	364.4	415
Q5HYL0	Putative u	MQRAVPE	1	1	2	1	967	105.3	6.83	51.4	49.3	38.6	41	49.9	28.4	32.2	48.9
Q6UWV6	Ectonucle	MRGLAVLL	5	2	3	2	458	51.5	6.89	219.4	157.8	192.7	178.4	197.1	172.8	150.7	193.3
K7ES00	Histone H3	MARTKQT	9	2	8	2	151	16.6	11.84	1428.3	1016.6	681.7	754.7	827.1	1406.8	1900.6	602.6
Q8NC54	Keratinocy	MAAAVPK	4	1	11	1	265	29.2	5.08	490.9	422.4	421.1	420.2	493.8	456.3	497.4	485.1
P15907	Beta-galac	MIHTNLKK	2	1	2	1	406	46.6	9.01	91.2	67.6	79.4	77.6	75.9	104.5	67.3	75.5
P22223	Cadherin-3	MGLPRGPL	3	2	54	1	829	91.4	4.75								
A0A0X9TD	GCT-A3 he	QVQLQESG	13	1	1	1	127	13.8	8.46								
Q96MM7	Heparan-s	MALPACA	3	2	3	2	605	69.1	9.73	138.9	122	121	106.4	117.8	127.9	89.3	94.5
P25774	Cathepsin	MKRLVCVL	8	2	2	2	331	37.5	8.34	108.5	98.8	80.5	87.4	102.4	121.4	97.5	115.9
P19075	Tetraspani	MAGVSAC	4	1	1	1	237	26	5.6	207.2	165.4	218	182.1	194	130.4	138.4	182
B3KT21	cDNA FLJ3	MCSGAGV	5	1	2	1	241	26.8	7.08	92.6	74.3	70.6	75.1	87.6	100.3	74.6	99.1
V9HW80	Epididymis	MASGADS	3	2	2	2	806	89.3	5.26								
Q9BS26	Endoplasm	MHPAVFLS	5	2	7	2	406	46.9	5.26	243	228.2	158.4	204.3	229.6	239.3	208.1	229.6
Q6FGQ5	ASGR1 pro	MTKEYQDL	4	1	1	1	291	33.2	5.5	229.7	206.2	158.4	233.7	247.8	269.9	231.5	266.7
A8K243	cDNA FLJ7	MACTIQKA	11	3	8	3	387	44.7	6.74	78.2	45.5	62.4	50.4	55	57.2	40.8	55
P22748	Carbonic a	MRMLLALL	5	2	5	2	312	35	7.83	335.3	298.2	233.2	357.8	421.7	312.2	296.8	300.4
B4DW34	cDNA FLJ5	MRLLAWL	6	2	2	2	455	50.8	5.64	49.7	38.2	44.3	47.3	42	52	40.8	44.2

Q9UN66	Protocadh	MEASGKLI	2	1	2	1	801	87.6	4.88	45	31	49.9	33.1	40.7	41.5	31.4	45.3
A4D1W8	Ependymir	MPPRMLV	7	2	8	2	344	38.1	9.58	406.2	412.1	301.2	360.1	393.5	365.2	408.3	403.7
O43488	Aflatoxin B	MLSAASRV	4	1	1	1	359	39.6	7.17	24.5	21.3	15.4	15.7	27	23.8	15.2	18.6
Q8WWZ8	Oncoprote	MPPFLLLT	2	1	5	1	545	60	5.58	130.7	103.9	78.8	104.1	90.5	106.3	102.2	90.4
B2R7D1	cDNA, FLJ9	MMDASKE	2	2	7	2	684	77.1	6.76	55.5	20.2	47.3	39.5	47.6	23	17.2	39.4
Q9BUD6	Spondin-2	MENPSPA	5	2	2	2	331	35.8	5.52	84.6	153	94.8	94.6	108.7	72.1	73	86.9
B5MCA4	Epithelial c	METKHLGF	8	2	2	2	342	37.9	8.47								
A0AVF1	Intraflagell	MMLSRAK	1	1	17	1	554	64.1	6.93	989.9	1068.8	738.7	1308.1	1250	1127.4	850.5	1223.2
Q9NZV1	Cysteine-ri	MYLVAGD	3	2	3	2	1036	113.7	5.21	75.1	85.6	67.2	89.7	82.2	96.3	66.9	79.9
Q86X29	Lipolysis-st	MQQDGLG	2	1	1	1	649	71.4	7.97	69.9	72.6	58.2	69	77	56	62.5	57.1
A0A024R8	ATPase, H+	MASQSQG	9	1	2	1	118	13.7	8.79	99.1	83.5	70.3	98.9	90.4	82.9	71.4	71.8
P49767	Vascular er	MHLLGFFS	3	1	2	1	419	46.9	7.52								
A0A024R1	Leukotrien	MVRTKTW	4	1	2	1	329	35.9	8.29	77.5	61.8	51.5	61.2	64.7	58.9	63.2	62.7
A1L471	ATP-bindin	MDLEGDR	2	2	3	2	1280	141.4	9	125.8	86.1	96.3	96.5	119.6	123.2	74	121
A8K2T7	Receptor p	MRPSGTA	1	1	4	1	1210	134.1	6.7								
V9HW55	Proteasom	MQLSKVKF	7	2	4	2	269	30.2	6.99	260.2	296.6	189.1	259.1	262.2	247.5	244	211.7
P03950	Angiogenir	MVMGLGV	13	2	6	2	147	16.5	9.64	404.3	446.7	333	406.2	389.5	423.6	441.8	422.6
A0A0S2Z4	Solute carr	MAEDKSKF	4	2	2	2	685	78.8	5.96	80.7	62.9	44.2	61.5	64	57.6	55.3	54.8
Q03167	Transformi	MTSHYVIA	2	1	3	1	851	93.4	5.71	97.9	80.6	58.7	84.1	80.2	93.4	88.1	66
C9IYI1	Dermokine	MLGITSCS	4	1	2	1	253	26.7	9.09								
H7C215	Reticulon-4	XVEEGAG	6	2	2	2	559	59.5	8.43	93.9	111	89.7	97.2	106.2	102.9	87	96.5
P55107	Growth/dif	MAHVPAR	3	1	2	1	478	53.1	9.51	219.7	161.8	160.6	172.8	181.2	183.7	155	234.2
P14784	Interleukin	MAAPALSV	5	2	3	2	551	61.1	5.05	179.6	170.7	131.8	161.9	159.9	177.7	149.1	188.2
V9HWH1	Epididymis	MEQLSSAN	6	2	2	2	379	42.7	6.28	86.5	65.8	58.1	85.4	68.1	78.1	76.8	59.9
O43405	Cochlin OS	MSAAWIPA	2	1	6	1	550	59.4	7.96	346.4	511.1	275.7	415	442.1	351.6	386.2	452.5
A0A1B0GW	Alpha-amir	MWRLPRA	3	1	1	1	554	60	8.18	31	29.2	24.1	23.1	24.2	34.6	25.3	23.3
F2RM35	Serine prot	MQRVNMI	2	1	2	1	461	51.7	5.39								
Q9NR34	Mannosyl-	MLMRKVP	3	2	2	2	630	70.9	7.46								
O00299	Chloride in	MAEEQPQ	11	2	9	2	241	26.9	5.17	104.1	95.7	71.5	94.6	102.6	87.8	80.7	80
A0A024R9	Mal, T-cell	MSAGGAS	6	1	6	1	176	19.1	6.24	132	109.8	84.5	94	101.7	107.9	88.7	104
P31150	Rab GDP d	MDEEYDVI	4	2	7	1	447	50.6	5.14	114.8	83.7	86.9	103.1	99.2	109	78	91.8
A0A024R3	Laminin, be	MELTSRER	2	2	3	2	1798	195.9	6.52	134.8	142.9	111	114.4	122.6	150.8	107.1	122.5
B2R7N9	cDNA, FLJ9	MGFLSPIY	4	2	4	2	421	49.4	5.52	338.1	349.5	238.1	343.8	309.6	357.4	319.5	309.7
P01704	Immunogl	MAWALLLI	13	1	5	1	120	12.6	6.49	304.8	454.4	233	303.9	281.4	342.4	229.9	246.2
Q8N126	Cell adhesi	MGAPAASI	5	1	5	1	398	43.3	6.09	73.7	95.3	77.6	79.7	78.2	95.7	96.6	80.7
A0A0X9V9	IBM-B2 ligh	EIVMTQSP	8	1	6	1	106	11.4	6.51	266.4	237.5	191.9	303.4	357.1	284.3	328.7	293.4
Q9HBJ8	Collectrin (	MLWLLFFL	6	1	7	1	222	25.2	5.63	109.6	79.1	82.2	100.7	125.9	115.8	83.4	94
Q86UZ8	FZD2 prote	ARGVSGCS	1	1	8	1	641	71.4	9	550.6	579.5	371.6	612.1	620.5	612.7	574.2	609.8
H3BQD0	Cerebellin-	MLGVLELL	11	1	8	1	140	14.7	5.48	317.7	722.6	197	399.5	338.3	205.1	329.1	333
Q96MU8	Kremen pr	MAPPAARI	3	1	1	1	473	51.7	7.11	48.9	33.9	29.8	33.6	29	33.5	27.3	27.5
P58546	Myotrophi	MCDKEFM	14	1	5	1	118	12.9	5.52	53.1	55.5	41.4	55.5	60.5	59.3	66.4	58.6

P22304	Iduronate	MPPPRTGF	2	1	5	1	550	61.8	5.45	142.9	111.3	112.3	119.5	122.3	122.3	114.7	123.9
B3KNV8	Polypeptid	MVCTRKTK	3	2	2	2	607	69.5	6.49	157.5	140.2	128.1	159.4	206.1	178	126.5	143.7
O75787	Renin rece	MAVFVVLL	5	2	5	2	350	39	6.1	973.9	474.6	387.6	490.8	579.7	599.7	413.1	450.3
Q5T7S2	Receptor p	MEAAVAAI	2	1	1	1	503	55.9	7.55								
O75071	EF-hand ca	MKKRKELN	4	2	8	2	495	55	6.32	330.6	300.4	204.3	311.9	342.7	292.9	321.9	337.1
D2JYI1	TGF-beta r	MGRGLLRC	3	1	3	1	592	67.4	5.88	75.2	79.8	66	58	57.9	57.7	60.7	55.8
P04118	Colipase O	MEKILILLLV	7	1	9	1	112	11.9	7.5	838.9	902.8	513.9	804.5	1277.9	865.7	738.1	777.9
O43567	E3 ubiquiti	MLLSIGML	4	2	6	2	381	42.8	4.98	649.5	599.2	425.8	697.7	681.4	632.1	715.8	675
A0A0G2JN	Mucin-6 OS	MVQRWLL	1	2	2	2	2439	256.9	7.39								
Q13790	Apolipopro	MTGLCGYS	4	1	6	1	326	35.4	5.64	161	116.9	111	115	175.3	183	196.6	235.1
Q9H9P2	Chondrole	MSRVVSLL	11	2	2	2	273	30.4	6.83	13.6	10.7	10.2	9.1	12.1	13.9	13.3	15.2
P08887	Interleukin	MLAVGCAI	3	1	2	1	468	51.5	8.22	65.3	66.6	55.5	67.5	58.2	74.7	61.2	57.9
A0A024RA	Calpastatir	MSQPGQK	2	1	1	1	803	86.6	5.14								
Q6UWH4	Protein FAI	MTCPDKPC	2	1	2	1	519	57.5	9.7	323.8	311.7	325.3	464.7	424.6	365.6	310.8	373.9
B2RDY9	Adenylyl cy	MADMQNI	4	2	2	2	475	51.6	8.22	218	156.3	174.4	161.1	153.9	175.6	157.4	273.4
A0A087X0	Growth ho	MNWGPTC	2	1	5	1	645	72.2	4.88	111.5	87.9	75	90.5	99.4	101.9	99	105.8
P51606	N-acylgluc	MSKGLPAR	3	1	2	1	427	48.8	6.37	88.6	67.2	88.8	73.3	70.8	73.7	51.2	55.8
P27824	Calnexin O	MEGKWLLC	4	2	3	2	592	67.5	4.6	30.4	34.9	38.5	26.9	26.2	31.8	29.9	29.3
B2RAN2	cDNA, FLJ9	MTTQLPAY	4	2	5	2	513	57	5.57	354.5	285.9	320.7	249.9	274.4	243.3	262.6	235.9
Q9UGM5	Fetuin-B O	MGLLLPLA	2	1	5	1	382	42	6.83	140	114.8	112.9	118.9	111.8	131.1	125.2	93.5
B9EKV4	Aldehyde c	MFLRAGLA	3	1	5	1	518	56.3	6.57	20.2	9.8	16.8	12.6	16.9	13.9	10.9	11.7
P78310	Coxsackiev	MALLLCFV	3	1	1	1	365	40	7.56	47.1	37.6	35.6	40	38.2	27.4	31.1	31.6
O75074	Low-densit	MEKRAAAC	2	1	3	1	770	82.8	6.21	189.3	150.2	159.1	185.1	210.7	203.7	194.8	218.8
Q0VDC6	Peptidylpro	MGRQRAE	10	1	1	1	145	15.7	9.17	35.3	33.8	40.5	34.5	35	37.7	38.2	33.7
A8K5Y2	cDNA FLJ7	MADIKTGII	1	1	4	1	695	76.2	4.64	405.2	432	309.4	414.4	409.5	361.2	330.4	420.4
Q96FE5	Leucine-ric	MQVSKRM	2	1	17	1	620	69.8	8.56	521.7	476.9	443.6	523.5	515	537.2	379.8	587.4
Q96MG2	Junctional	MSMTTRA	6	2	4	2	331	36.3	9.38	258.5	272.2	176.9	234.6	310.4	225	192.1	262.2
A8K9U9	cDNA FLJ7	MATLVVNI	2	1	4	1	574	64	6.43								
D6W5K2	Thymosin,	GVSSRFPEF	8	2	7	2	169	17.9	10.48	1247	1089.4	536.4	1030.5	1004.3	1341.3	1170.9	878.5
A0A0B5HR	Serine/thre	MVDGVMI	4	1	2	1	509	57.1	7.33	117.6	109.8	106.5	123.8	133	114.8	113.3	128.2
Q9NWV4	UPF0587 p	MGKIALQL	16	2	3	2	160	18	5.01	259.2	373.2	182.5	221.3	246.3	177.1	199.5	206.9
P02545	Prelamin-A	METPSQRF	2	1	1	1	664	74.1	7.02	173.5	98.4	115.4	86	73	89.4	66.8	64.1
A4D2P0	Ras-relatec	MQAIKCVV	10	2	3	2	211	23.5	8.63	84.6	71	71.3	84.1	105.5	82.4	93.3	78.2
Q86YT9	Junctional	MFCPLKLIL	5	2	3	2	394	44.3	7.12	124.7	180	88.4	138.5	122.5	103.1	101.5	101.9
P15529	Membrane	MEPPGRRE	4	1	4	1	392	43.7	6.74	35.9	45.1	31.2	44.7	39.4	33.8	35.4	40.8
Q9BYE4	Small proli	MSYQQQC	18	1	2	1	73	8.2	7.96	225.5	254.8	318.1	255.2	243.8	263.5	244.6	217.1
H7BZJ3	Protein dis	SDVLELTDI	11	1	2	1	123	13.5	7.3								
B2RC95	cDNA, FLJ9	MAEEEAPK	2	1	7	1	647	71.1	7.99	144.5	134.2	114.9	142.2	146.9	130	120.5	122.9
Q9BYJ0	Fibroblast	MKFVPCLL	5	1	1	1	223	24.6	8.87	58	64	38.3	57.8	70.6	49.3	57.2	64.1
Q96PC5	Melanoma	MAKFGVHI	1	2	3	2	1412	159.7	4.69	112.2	94.7	98.6	106.7	106.4	110.9	102.6	116.3
Q9UMX5	Neudesin C	MVGPAPRI	5	1	10	1	172	18.8	5.69	229.2	209.7	167.5	226.5	244.1	222.8	206.9	235.1

Q9Y4I9	Tyrosine pl	MGPPLPLL	1	1	2	1	986	108	5.91	155.4	147.2	121.1	139.4	156.3	131.5	109	150.1
P11234	Ras-relatec	MAANKSK(	7	1	2	1	206	23.4	6.62	29.3	22.5	20.2	20.2	25.2	39.8	17.4	19.1
Q16581	C3a anaph	MASFSAET	3	1	1	1	482	53.8	6.67	70.9	63.9	67.8	64.7	70.1	65.3	63.1	63.4
B2R6S5	UMP-CMP	MLSRCRSG	4	1	6	1	228	25.8	7.97	406.5	384.8	240.2	339.2	404	438.8	308.5	319.9
A0A1B1RV	Lipoprotei	MESKALLV	6	2	2	2	475	53.1	8.15	80.3	60.9	70.8	74.6	80.2	69.5	64.4	56.3
Q59ED5	Tetraspani	PFSVVDAR	5	1	2	1	285	31.7	8.37	62.9	84.1	55.4	67.3	65.1	51.7	63.2	67.4
A8K139	cDNA FLJ7(	MVVALRY\	2	2	5	2	1257	139.9	6.24	96.3	73.4	67.1	72.2	85.9	68.7	69	68
G3V5Z7	Proteasom	MSRGSSAG	8	2	2	2	252	28.1	6.76	170.1	150.6	125.1	142.8	125.9	146.2	113.5	106.8
Q8NFU3	Thiosulfate	MAGAPTV!	8	1	1	1	115	12.5	6.07								
P32241	Vasoactive	MRPPSPLP	3	1	1	1	457	51.5	8.18	40.8	43	32.2	42.1	34.4	35	35.3	35.1
Q8IYJ0	PILR alpha-	MESRMWI	2	1	3	1	282	30.1	9.1	128.5	124.5	84.4	124.2	117.3	122.3	89.9	120.9
A0A075B6!	Protein IGI	MDMRVPA	14	1	2	1	117	12.7	9.25	36.2	41.8	32.6	23.3	32	39.4	25.9	20.1
A0A087WY	Tumor pro	MDSAGQD	4	1	4	1	222	23.8	6.55	266	228.2	169.7	242.9	214.3	266.8	244.4	215.4
Q03164	Histone-lys	MAHSCRW	0	1	10	1	3969	431.5	9.09	472.5	2094.7	378.3	540	729.5	922.7	521.2	606.1
P13639	Elongation	MVNFTVD(	2	1	1	1	858	95.3	6.83								
O95502	Neuronal p	MKFLAVLL	2	2	2	2	500	52.8	6.21								
X5D2G8	Fibroblast g	MGAPACA	2	2	3	2	807	87.8	5.9	316.4	228.2	256.8	255.9	249.8	294.5	206.5	237.9
A0A024R3	Eukaryotic	MGFGDLK!	7	1	1	1	225	24.7	4.67								
Q8WY15	Sodium/glu	MEEHTEA(	2	1	1	1	698	75.6	7.46								
C5IX07	Tumor-ass	MARGPGL	4	1	1	1	323	35.7	8.87	130.5	115.3	103.3	129.5	127.1	125.5	120.7	127.5
Q9H2A7	C-X-C moti	MGRDLRP(	6	1	1	1	254	27.6	8.78								
A0A075B7	Protein IGI	MDWTWR	16	2	3	1	117	13	9.13								
I3L0A0	HCG20447	MAGAENW	5	2	7	2	370	42.2	6.71	377.8	175.7	137.8	193	212.5	188.3	177.6	148.5
Q7L5L3	Glyceroph	MSLLLYYAI	4	1	1	1	318	36.6	7.97	18.2	12.9	10.4	9.7	12.6	16.3	15.3	11.7
Q9GZP0	Platelet-de	MHRLIFVY	2	1	3	1	370	42.8	7.97								
A8K061	cDNA FLJ7	MFTIKLLLF	2	1	3	1	460	53.6	6.6	64.3	67.7	76.5	67	89.8	81.7	73.2	74.5
O00622	Protein CYI	MSSRIARA	3	1	1	1	381	42	8.21	41.8	41	33.5	39.3	44.8	42.5	38	39.4
A0A1K0FU	Globin E1 (	MLSAQERA	11	1	1	1	141	15.6	6.62	18.4	13.1	13.3	9.4	11	19	11.2	7.8
P10721	Mast/stem	MRGARGA	1	1	1	1	976	109.8	6.98	59	57.5	50.6	62	59.2	53.7	51.9	56.5
Q9ULK6	RING finge	MAMSLIQ/	2	1	11	1	438	48	5.27	261.6	235	217	266.4	292.7	242.5	220.3	263.9
Q8NG20	Plasminoge	MSEGNSD(	14	2	6	2	90	9.8	7.14	122.3	103.2	93.3	119.2	128.8	136.6	211.5	180.8
Q8N2G4	Ly6/PLAUR	MWVLGIA	9	1	1	1	141	15.2	8.02								
A0A0G2JH	Myelin-olig	MASLSRPS	4	1	2	1	295	33.5	9.14	99.9	90.3	80.3	99.6	104.5	91.2	80.9	99.1
Q53Y06	ATPase, H+	MALSDAD\	4	1	4	1	226	26.1	8	222.5	215.2	198.8	192.5	212.5	224.7	172.8	145.2
B5TMG5	Histidine ri	MGHHRPW	1	1	5	1	750	86.2	4.75	603	401.1	373.4	572.2	635.3	520.7	435.4	636.8
B7Z6S9	Glucosylce	MSDRLFSN	1	1	4	1	621	68.7	7.94	122.6	87.3	87.1	96	128	102.8	80.7	86.2
Q9H6Y7	E3 ubiquiti	MHPAAFPL	3	1	3	1	350	38.3	5.63	101.6	68.8	62.8	85	78.9	83.8	78.9	93.1
Q6ZSJ9	Protein shi	MALRRLLLI	2	1	2	1	500	55.7	9.35	116.6	120	86.5	107.4	104.5	123	91.1	109.9
G9FP35	Guanine nu	MTLESIMA	3	1	5	1	359	42.1	5.68	134.5	134.8	103.7	151	151.6	153.1	124.5	122.5
O14786	Neuropilin-	MERGLPLL	2	1	4	1	923	103.1	5.88	17	13.9	10.9	9.3	8.1	13.4	12.5	11.3
Q7L9L4	MOB kinas	MSFLFGSR	5	1	1	1	216	25.1	6.73	20.6	9.6	11.8	11.5	18.8	15.3	12	10.2

Q59FM9	TYRO3 pro	RRRPRPEA	2	1	3	1	951	102.8	6.42								
B2RB70	Neurocalci	MGKQNSK	8	2	2	2	193	22.2	5.35	193.8	147.6	126.8	158.4	186.1	177.7	138.9	155
A0A024R2	Tyrosine-p	MSSWIRW	2	1	2	1	838	93.8	6.57	24.4	28.1	13.5	27.7	22.5	22.9	22.5	25.5
Q53TN4	Cytochrom	MAMEGYW	3	1	1	1	286	31.6	8.76	212.3	168.5	174.6	222	237.1	180.6	148.3	186.2
Q15043	Zinc transp	MKLLLLHP	2	1	3	1	492	54.2	5.33	163.4	128.1	122.7	168	153	257.6	128.8	133
Q08708	CMRF35-lil	MTARAWA	4	1	8	1	224	24.8	9.25	433.4	357.3	289.4	479.1	520.1	507.8	424.8	425.2
A0A0B4J1S	High affinit	MDGEATV	1	1	3	1	577	62.1	9.47	321.4	269	236	340.9	291.6	368.7	347.6	380.5
P0DKV0	Spermatog	MENLPFPL	2	1	7	1	1188	130.4	9.42	605.1	512.5	762.5	501.2	473	581.2	639.1	633.5
A0A087X1	Serpin B6 (	MSSRQRGI	5	1	1	1	395	44.8	5.68	63.8	65.1	50	56.8	64.3	59.2	57.3	59.1
V9GYJ8	Apolipopro	MGTRLLPA	13	1	1	1	87	9.6	4.53								
Q7Z7G8	Vacuolar p	MLESYVTP	0	1	4	1	4022	448.4	6.46	192.1	268	132.2	204.4	219.2	153.4	185.6	184
A0A0X9US	MS-D2 ligh	DIVMTQSP	17	1	2	1	107	11.4	7.96								
A8K9D8	cDNA FLJ7	MATVRASL	1	1	2	1	971	106.4	6.68	103.8	78.4	76.9	75	75.2	77.5	73.2	79.3
A0A087W1	Protein sid	MARGARP	1	1	1	1	2213	241.9	6.49								
P61457	Pterin-4-al	MAGKAHR	10	1	4	1	104	12	6.8	247.2	161.1	146.9	164.6	199	176.3	161.5	182.4
P80511	Protein S1(	MTKLEEHL	8	1	5	1	92	10.6	6.25	1210	875.6	607.9	1073.4	1010.6	1011.6	1638.1	750.5
Q8WWQ8	Stabilin-2 (	MMLQHLV	0	1	1	1	2551	276.8	6.4	26.1	15.4	19.7	16	21.3	17.4	17	11.6
A0A087WS	Nucleobind	MRWRTILL	2	1	2	1	420	50.2	5.12	86.5	56.8	43.7	72.2	64.8	62.8	53.6	49.3
A0A125U0	MS-C3 ligh	DIQMTQSF	17	1	1	1	106	11.4	4.83	117.6	79.2	155.7	107.3	114.3	84.8	93	98.8
Q6UW88	Epigen OS=	MALGVPIS	8	1	1	1	154	17.1	6.95	84.1	51.8	54.8	63.1	64.6	60.6	54.9	50.2
P09382	Galectin-1	MACGLVAS	9	1	2	1	135	14.7	5.5	78.6	83.7	69.6	86	77.4	71.7	53.6	68.1
P62070	Ras-relatec	MAAAGWF	6	1	1	1	204	23.4	6.01	83	54.3	65.6	75.1	77.2	81.2	54.4	61.1
B2RCC8	cDNA, FLJ9	MFLWLFLII	2	1	1	1	837	94.4	7.97	34.1	21.8	17.4	21	22.2	30.9	18.9	17.6
Q96RZ2	N-acetylglu	MAAGLARI	3	1	2	1	331	36.8	6.79	63.6	47.3	40.9	49	66.4	53	47.9	59.5
P01127	Platelet-de	MNRCWAL	5	1	1	1	241	27.3	9.16	74.1	50.8	50.1	53.7	54.8	62.9	49.5	56.2
A0A0C4DH	Protein IGK	MVSPLQFL	10	1	1	1	115	12.6	7.12	185.7	152.5	136.9	154.5	154.7	161	193.7	163.8
B2R9K8	cDNA, FLJ9	MAAVKTLN	2	1	1	1	531	57.9	6.8	45.5	22.9	21.2	23.8	29	38.9	27.8	17.3
Q9Y6U3	Adseverin (	MARELYHE	1	1	3	1	715	80.4	5.71	92.6	92.8	64	79.2	73	84.9	85.4	70.8
O43493	Trans-Golg	MRFVVALV	2	1	1	1	480	51.1	5.73	163	121.1	104.1	144.2	141.3	178.1	128.6	164.8
Q9HCM3	UPF0606 p	MPGARRR	1	1	1	1	1950	210.6	6.11	26.1	16.9	15.7	19.2	21.6	21.3	18.8	22.7
P13521	Secretogra	MAEAKTH\	2	1	2	1	617	70.9	4.75	186.6	232.1	131.2	217.4	159.4	226.3	149.7	132.3
P16234	Platelet-de	MGTSHPAF	2	1	2	1	1089	122.6	5.17	33.2	26	30.5	26.2	29.4	22.1	27.9	29.7
Q96AX9	E3 ubiquiti	MGWKPSE	1	1	4	1	1013	109.9	8.44	84.1	28.5	30.2	59.4	52.6	78	45.5	88.1
Q86YD5	Low-densit	MWLLGPL(	5	1	1	1	345	37.4	4.87	14.4	17.7	9.9	16.5	11.8	14.2	11.6	10.8
A6NL88	Protein shi	MPALLLLV	2	1	1	1	538	56.2	10.02	80	57	133	76.5	74.3	73.3	62.3	75.4
E5RIM7	Copper tra	MKAESPGK	23	1	1	1	73	7.9	7.24	31.9	34.8	32.1	25.7	27.2	34.5	35	29.3
Q9NQ79	Cartilage a	MAPSADP(	2	1	1	1	661	71.4	5.12	91.6	71.7	85	85.4	74.4	96	73.4	82.3
O94991	SLIT and N	MHTCCPP\	1	1	4	1	958	107.4	6.95	135.1	156.6	100.1	122.8	109.4	155.5	94.3	98.7
B4DZM1	cDNA FLJ5	MTKLPDDF	2	1	3	1	872	100.5	5.02	243	208.8	295.4	222.6	225.1	244.1	219.2	261.6
B7Z6Z4	Myosin ligh	MVGPRSW	3	1	1	1	238	26.7	5.08	80.6	56.1	37.9	54.2	63.5	67.3	69.6	66.5
O14494	Phospholip	MFDKTRLP	4	1	1	1	284	32.1	7.97	196.6	151.5	147.6	149.9	176.9	126.3	151.3	152.4

Q8N386	Leucine-ric	MGGTLAW	3	1	4	1	305	33.2	5.05								
Q9UHY7	Enolase-ph	MVVLSVPA	5	1	1	1	261	28.9	4.78	17.8	13.5	11	10.6	11.3	13.5	10.1	11.8
P17927	Compleme	MGASSPRS	1	1	2	1	2039	223.5	6.95	52	53.3	36.8	37.4	47.2	55.1	35.7	48.3
P29317	Ephrin type	MELQAARA	1	1	1	1	976	108.2	6.23								
Q96S16	JmjC doma	MVTGGAH	3	1	1	1	334	36.8	9.72								
P56159	GDNF fami	MFLATLYFA	3	1	1	1	465	51.4	7.91	412.4	328.2	386.6	351.5	317.1	499	312.1	334.3
Q9Y490	Talin-1 OS=	MVALSLKIS	0	1	1	1	2541	269.6	6.07	49.7	55.6	32.6	40.9	45.8	57.4	49.1	46.5
Q59GD9	Myelin ass	RSRGAEAT	2	1	1	1	661	72.7	5.16								
A0A024R6	Galactosylc	MAEWLLSA	1	1	2	1	685	77	6.64	99.9	78.5	61.6	82.6	77.4	78.2	78.9	63.7
A0A024R1	Parvalbum	MSMTDLLN	12	1	1	1	110	12.1	5.19								
A0A024RB	Solute carr	MMGSPVS	2	1	2	1	540	56.4	6.83	103.6	57.4	45	58.4	77	79.3	42.8	65.3
Q5T7Y6	Protein S1	MEEPQTKT	10	1	1	1	147	15.9	4.48								
Q59EU6	EF hand do	CEERAARQ	4	1	1	1	265	29.8	6.21	22.4	12.7	13.6	12.2	18.2	17.3	13.4	10.2
Q7Z4W1	L-xylulose	MELFLAGR	4	1	3	1	244	25.9	8.1	245.2	167.9	190.3	178.1	206.6	172.8	148.9	161
Q6I929	HCG19786	MDGTRTSL	5	1	1	1	246	28.4	8.21	34.1	22.8	32	28.4	29.2	25.4	25.8	27.4
A0A0F7RQ	Follicle stir	MKTLQFFF	6	1	6	1	129	14.7	7.06	306.7	243.5	193.2	218.8	312.8	311	291.6	303.2
V9HWJ1	Glutathion	MATNWGS	2	1	2	1	474	52.4	5.92								
S4R347	Formin-bin	MSWGTEL	2	1	1	1	609	70.5	6.64								
A0A1B0GU	Characte	MYGDIFNA	13	1	1	1	103	10.9	8.92	25.5	14.2	22.1	17.1	16.8	23.3	22.6	27.8
A0A087WU	Protein SYN	MNGRVDY	6	1	1	1	186	20.8	5.19								
Q9H741	UPF0454 p	MVNLAAM	6	1	1	1	205	23.6	8.1	30.5	34.9	27.2	29.7	28	26.2	23.4	29.4
P30101	Protein dis	MRLRRLAL	2	1	2	1	505	56.7	6.35	45.8	50	38.6	41.1	37.8	48.8	39.5	33
P22692	Insulin-like	MLPLCLVA	5	1	2	1	258	27.9	7.15	149.9	148.4	158.8	128.6	133.5	145.8	128.8	130.8
Q9H0X4	Protein FAI	MLDHKDLE	2	1	2	1	552	59.6	6.28	61.4	44.4	48.8	50.7	53.1	49.7	37	42.8
Q6ZMU0	Delta-amin	MPPTSSTP	3	1	2	1	359	39	7.65								
Q8TBD0	Uncharacte	RTQHCSSS	8	1	1	1	132	14	7.9	84.6	76.7	96	96.2	84.9	80.6	110.1	77.9
X6R7Y7	Intraflagell	MRKIDLCLS	5	1	1	1	214	24.3	8.1	34.8	34.4	22.2	36	28	31.7	30.1	26.7
B4DU44	Involucrin	MSQQHTLI	2	1	1	1	595	69.6	4.6	43.4	25.7	34.3	28.3	30.1	26.8	39.5	29.9
Q8TER0	Sushi, nido	MRHGVAW	1	1	1	1	1413	152.1	6.98								
Q9Y3R5	Protein do	MDPEEQEL	0	1	1	1	2298	258.1	6.29	33.4	52.7	33.8	23.2	25.8	24.2	17.6	18.7
B2R8C2	Tyrosine-p	MSNICQRL	2	1	1	1	505	58.3	6.8								
P09543	2',3'-cyclic-	MNRGFSRH	3	1	1	1	421	47.5	9.07								
A0A024QZ	NAD(P)H d	MAGKKVLI	4	1	1	1	231	25.9	6.29	99.8	61.1	137.6	84.7	91.9	87.9	77.4	66.2
P51159	Ras-related	MSDGDYD	4	1	2	1	221	24.9	5.22	207.2	259.3	193.5	185.5	183	126.7	123.1	190.9
P55010	Eukaryotic	MSVNVNR	2	1	4	1	431	49.2	5.58	330.4	250.3	242.2	292.2	297.8	333.6	296.7	346.4
P48509	CD151 anti	MGEFNEKK	4	1	2	1	253	28.3	7.47	43.6	32.2	27.4	33.7	34.8	40.1	27.9	30.4
P18085	ADP-ribosy	MGLTISSLF	6	1	1	1	180	20.5	7.14	36.6	24.6	32.1	20.4	23.7	18.6	18.6	20.6
Q53H88	Dynactin 2	MADPKYAI	2	1	4	1	406	44.8	5.12	92.2	90.7	67.6	89.7	97.4	83.6	99.5	101.2
A8K8U1	cDNA FLJ7	MASASYHI	1	1	1	1	1230	136.2	5.83	36.4	29.7	24.4	26.6	34.4	30.3	28.7	26
B3KR33	cDNA FLJ3	MNPIVVVH	3	1	1	1	308	32.1	6.1	195.3	159.1	118.3	162.6	152.5	146.7	135.4	155.8
P54826	Growth arr	MVAALLGC	3	1	10	1	345	35.7	5.55	955.2	877.9	660.9	943.3	941.9	946.1	817.2	887

B4DWA6	cDNA FLJ6(	MGGPQDK	3	1	2	1	335	37.5	6.14	188.6	155.8	147.6	140.3	151.8	157	157.1	153.1
D6W551	Chromosor	MKTSAELH	2	1	1	1	284	30.3	8.38	62.1	49.9	45.2	56.3	53.7	93.7	61.1	73.6
A0A140VJJ	S-formylglu	MALKQISSI	6	1	1	1	282	31.4	7.02								
A0A024R3I	Sodium cha	MPAFNRLF	4	1	3	1	215	24.7	4.68	169	131.3	121.3	136	165.8	129.4	130.2	167.9
A0A0A7E7	Endoplasm	MVFLPLKW	1	1	1	1	941	107.2	6.4								
O95861	3'(2'),5'-bis	MASSNTVL	3	1	1	1	308	33.4	5.69	35.5	23.6	20.7	26.8	33.3	31.7	25.7	21.3
A2NW98	Rheumatoi	MVLQTQV	7	1	5	1	134	14.8	7.12	184.1	175.2	115.5	138.6	151.4	138	204.9	186.7
Q9HD42	Charged m	MDDTLFQL	4	1	2	1	196	21.7	8.06	131	102.8	87.8	109.3	136.5	115.9	107	126.2
B7Z6C9	Transmem	MSGLGRLF	2	1	1	1	815	92.4	6.29	34.2	23.2	32.1	28.8	36.5	27	24.3	32.7
B2RAF9	Suppressor	MGSDRARI	2	1	1	1	855	94.7	6.52	48.5	61.9	42.9	45.8	50.1	48.5	43.1	33.1
P80723	Brain acid s	MGGKLSKK	4	1	1	1	227	22.7	4.63	314.6	286.2	207.2	300.1	245.8	255.1	209	268
Q92743	Serine prot	MQIPRAAL	3	1	1	1	480	51.3	7.83	299.2	82	52.3	72.8	66.7	123.9	70.4	74.4
Q8WU39	Marginal zo	MRLSLPLLL	6	1	2	1	189	20.7	5.57	39.7	45.7	28.3	46.6	36.5	35.9	39	35
O15031	Plexin-B2 (	MALQLWA	1	1	1	1	1838	205	6.24								
Q9UBV8	Peflin OS=H	MASYPYRC	3	1	1	1	284	30.4	6.54	153.7	104.5	114.8	123.8	160	110.4	104.2	99.1
Q13740	CD166 anti	MESKGASS	2	1	1	1	583	65.1	6.25	22.8	24.7	23	22.7	23.7	21	19.2	24.8
C9JIF9	Acylamino-	MERQVLLS	2	1	1	1	737	81.6	5.54	50.4	48.8	39.8	45.2	45	55.3	33.9	37.4
A0N5G7	Rheumatoi	GGGVVQP(	9	1	1	1	119	13	8.94	107.1	80.3	141.9	77.7	82.6	93.1	102	88.1
U3KQ56	Glyoxylate	MRPVRLM	5	1	1	1	358	38.7	8.02	18.4	9.6	14.1	9.9	14.3	12.3	10.1	11.8
P46952	3-hydroxya	MERRLGVF	4	1	2	1	286	32.5	5.88	85.5	71.6	66.2	73.7	85.9	76.9	59.6	70.8
Q9BQR3	Serine prot	MRRPAAVI	3	1	1	1	290	31.9	8.13	65.2	64.6	51.4	74.6	71.8	76	74.8	62.1
Q13277	Syntaxin-3	MKDRLEQL	3	1	1	1	289	33.1	5.44	140.8	89	118.1	125.9	121	118.4	103	106
Q14764	Major vaul	MATEEFIIR	2	1	1	1	893	99.3	5.48								
D6R956	Ubiquitin c	MQLKPME	4	1	3	1	241	26.8	5.81	169.8	108.5	139.3	134.8	165.9	171.8	143.1	147.3
P16930	Fumarylace	MSFIPVAEI	2	1	1	1	419	46.3	6.95								
A2TDC0	Titin-cap (T	MATSELSC	7	1	1	1	167	19	5.25								
Q99584	Protein S1(	MAAEPLTE	11	1	1	1	98	11.5	6.16	108.2	85.2	96.5	90.9	113.3	121.5	98.4	100
A0A0S2Z61	Transmem	MAVWSLLS	8	1	2	1	219	23.9	6.15								
Q15366	Poly(rC)-bi	MDTGVIEG	4	1	2	1	365	38.6	6.79								
L7RSM2	Mitogen-ac	MSQERPTF	2	1	1	1	360	41.5	5.88	121.3	89	86.5	128.3	96.7	114.6	131.8	120.6
Q86SQ4	Adhesion G	MMFRSDR	1	1	1	1	1221	136.6	7.87								
P29373	Cellular ret	MPNFSGN	7	1	4	1	138	15.7	5.4	47.2	40.6	28.7	39.7	40.1	44.5	50.4	32.7
C9JVC9	Voltage-de	MAVPART(	1	1	2	1	1153	130	5.8	212	192.2	201.4	215.3	217	205.6	203.3	225.8
A0A024R6	Endothelin	MNKSTCLN	2	1	1	1	532	59.4	9.25	227.3	195.1	217.3	220.6	229.2	234.7	194.8	216.1
Q9NX62	Inositol mo	MAPMGIRI	3	1	2	1	359	38.7	6.86	22.3	18.3	15	17	23	24	17.2	13.2
Q9UBG0	C-type mar	MGPGRPA	1	1	1	1	1479	166.6	5.83								
P28072	Proteasom	MAATLLAA	5	1	1	1	239	25.3	4.92	61.9	39	53.2	40.9	49.1	47.2	43.9	55.8
P40197	Platelet gly	MLRGTLLC	2	1	1	1	560	60.9	9.63								
P46781	40S ribosor	MPVARSW	5	1	1	1	194	22.6	10.65	156.7	139.9	111.5	155.4	134.7	90.5	199.3	93.2
A0A024RA	HCG20096	MGWIRGR	5	1	1	1	216	25.3	8.21	93.7	58.4	58.6	48.5	57.5	67.9	59.5	63
Q5R3I4	Tetratricop	MAAASPLR	2	1	1	1	469	52.8	5.99								

A0PJK1	Sodium/glu	MAANSTSD	2	1	1	1	596	64.3	7.61								
A0A140VK	Leukotrien	MPEIVDTC	2	1	1	1	611	69.2	6.18	49.7	34.5	30	42.1	50	29.8	38.7	28.3
P23434	Glycine cle	MALRVVRS	6	1	4	1	173	18.9	4.88	101.3	94.3	100.8	94.9	95.6	102.4	86.1	81.5
Q96HA4	Uncharacte	MALRHLAL	3	1	1	1	380	40.3	10.01	186.6	156.9	179.8	192.1	167.4	158	185.4	194.7
Q96ID5	Immunoglo	MRTAPSLR	2	1	1	1	467	51.8	6.93	28.9	32	17.1	31.9	26.6	25.8	22.6	35.5
J3QRU1	Tyrosine-p	MLDLIMGC	1	1	1	1	548	61.3	6.57	98.1	69.7	95.8	90.3	92.8	94.3	70.2	81.5
A0A0U1RC	Neurexin-3	MSSTLHSV	0	1	4	1	1664	183.2	5.74								
D0AB04	Rhesus blo	GLLLNLKIW	28	1	4	1	25	3	9.42								
D3DSM8	Formimino	MRSRWGG	2	1	1	1	581	63.3	6.3	46.7	34.5	25.4	26.1	42	36	20.6	26.4
A0A024R5	ADAM met	MVLLRVLIL	1	1	1	1	748	84.1	7.77	371.3	122.3	98.8	113.3	106.5	113.2	85.4	93.2
A0A024R1	Tyrosylpro	MRLSVRRV	3	1	1	1	377	41.9	9.09	77.1	96.7	65.4	77.8	80.1	60.1	81	88.3
P28827	Receptor-t	MRGLGTCL	1	1	1	1	1452	163.6	6.65								
G3V4P8	Glia matura	MNKFVGLS	7	1	1	1	150	17.5	5.31	36.3	31.5	22.9	29.2	34.5	30.5	28.9	28.9
Q8IWX7	Protein unc	MAEVEAVC	1	1	1	1	931	103.7	7.81								
Q9Y639	Neuroplast	MSGSSLPSA	2	1	1	1	398	44.4	7.99	273.7	220.7	233.1	240.6	234.9	281.1	209.7	263.4
Q8IY95	Transmem	MAAGGRM	4	1	1	1	271	30.9	7.99								
P14778	Interleukin	MKVLLRLIC	1	1	2	1	569	65.4	7.83	98.9	109.7	77.3	98.7	132	90	76.5	90.2
P46108	Adapter m	MAGNFDSI	2	1	1	1	304	33.8	5.55	170.4	145.4	93.8	128.3	163.7	186.5	125.6	139.5
Q9Y546	Leucine-ric	MSYYLSSEI	3	1	6	1	428	48.5	7.46	580	771.1	389.3	745.5	575.7	444.1	517.5	408
B2R6Q2	Alkaline ph	MISPFLVLA	2	1	2	1	524	57.2	6.57	20.5	12.8	30.9	18.2	16.3	17.3	16	12
Q9H772	Gremlin-2 (	MFWKLSLS	7	1	1	1	168	19.3	9.17	72.7	99.2	63.1	96	89.2	87	77.2	86.7
P10109	Adrenodox	MAAAGGA	4	1	3	1	184	19.4	5.83	282.1	287.3	212.5	228.2	224.3	267.8	212.3	205.4
P23458	Tyrosine-p	MQYLNIKE	1	1	2	1	1154	133.2	7.55								
Q8N353	TMEM106	PRAPGRSLI	3	1	2	1	314	35.1	8.22	249.7	232	285.2	289.5	240.2	269.5	296.3	215.6
Q8NF12	FLJ00390 p	SQRGKGTC	5	1	1	1	322	35	6.68								
Q7RU04	Aminopept	MASGEHSF	2	1	1	1	658	73.5	6.11								
P0C7U0	Protein ELF	MAGRGWC	1	1	1	1	828	90.4	8.37								
Q6ZUY8	Lipase OS=	MACLEFVP	4	1	1	1	401	45.4	7.11	45.1	34.9	57.1	45.1	45.9	45	37.5	40.4
Q6LEU6	HLA-DMB p	MITFLPLLL	5	1	1	1	263	28.9	7.44	37.6	28.8	35.7	28.2	25.5	29.5	24.1	30.4
P10586	Receptor-t	MAPEPAPC	0	1	2	1	1907	212.7	6.3	109.7	122.4	119.4	110.8	116.3	122.4	95.8	121.8
A8K1K1	cDNA FLJ7	MDPKLGRI	2	1	1	1	507	56.7	5.36	83.8	61.6	94.7	56.6	65.2	67.2	60.9	63.4
Q8TDY8	Immunoglo	MARGDAG	1	1	1	1	1250	134.1	6.2								
A0A087WU	ADP-ribosy	MSDLRITEA	6	1	1	1	204	22.9	5.63	33.6	58.9	30.3	37.4	33.8	31.1	29.3	32
Q99835	Smoothene	MAAARPAI	1	1	4	1	787	86.3	8.34	292.2	222.3	203.6	298.2	367.2	328.6	232.8	361.7
B3KQQ9	cDNA PSEC	MAGIPGLL	3	1	1	1	383	43	9.36	97.5	62.7	88.2	82.1	80.9	84.8	69.6	79
Q6ZP37	cDNA FLJ2	MAALRQPC	3	1	1	1	396	42.9	7.24								
O15393	Transmem	MALNSGSF	2	1	4	1	492	53.8	7.81								
O00159	Unconvent	MALQVELV	2	1	1	1	1063	121.6	9.41								
A0A024RD	Tyrosylpro	MVGKLKQI	3	1	1	1	370	42.2	9.09								
P19957	Elafin OS=H	MRASSFLIV	14	1	4	1	117	12.3	8.82	204	327.2	133.1	251.3	208.2	235.6	373.9	296.5
Q5T1S8	Noncompa	MTTATPLG	7	1	16	1	102	11.1	9.86	2252	1819.3	1528.2	2147.8	2612.8	2270.6	1851.4	2795.3

B2RAK1	cDNA, FLJ9	MEPFTNDF	1	1	2	1	1025	117.3	5.73								
A6NH11	Glycolipid t	MGVAARP	3	1	1	1	291	31.6	10.15								
P08833	Insulin-like	MSEVPVAF	5	1	1	1	259	27.9	5.19								
Q99795	Cell surface	MVGKMW	3	1	1	1	319	35.6	4.93								
Q96TA2	ATP-depen	MFSLSSTV(	1	1	1	1	773	86.4	8.76	108.6	85.8	72.9	123.8	115.8	164.1	164.7	165.7
F6SYP7	Integral me	MVPKADS(	2	1	1	1	550	60.8	5.36	262.6	279	195.5	282.6	250.6	267	259	280.9
B2R6I6	cDNA, FLJ9	MLQNSAVI	8	1	1	1	247	27.6	7.99								
E9PGC5	Receptor-t	MDTTAAA/	1	1	1	1	1472	165.9	5.91								
Q8TE68	Epidermal	MSTATGPE	1	1	1	1	723	80.2	6.04	47.8	44.8	34.9	46.7	42.8	47.2	36.8	39.2
Q14165	Malectin O	MLGAWAV	4	1	1	1	292	32.2	5.41	67.4	64.6	55	59.6	62.8	56.9	56.1	67.8
A2KUC4	UGa8L (Fra	ELTQPHSV!	7	1	2	1	112	12.2	5.1	375.7	319	316.4	301.3	390.5	367.6	435.5	372.5
M1VPF4	Tyrosine-p	MMEAIKKK	2	1	1	1	725	82.4	4.97	81.7	71.7	61.5	73.3	52.8	53.8	55.3	49
D3DQ48	Transmem	MAAPKGSI	3	1	3	1	324	36.2	5.05	178.8	126	154.3	153.8	119.6	169	123.9	123.5
B4DT57	cDNA FLJ61	MGRDQRA	2	1	3	1	450	50	5.6	60.3	54	42.4	62.1	66.2	57.4	44.3	54.3
Q9ULL1	Pleckstrin I	MELSDSDR	1	1	8	1	1385	155.3	6.24	520.4	833.4	440.1	566.1	484.9	464.1	457.1	396.8
A0A0B4J1S	Protein SEI	MVAMAAG	8	1	1	1	165	18	5.03								
Q8N729	Neuropept	MAWRPGE	7	1	1	1	165	18	11.62	21	19.1	14.8	17.4	23.6	19.7	15.6	17.8
B3KPC7	Actin-relat	MARNTLSS	8	1	2	1	153	17	6.02								
B4DH02	cDNA FLJ50	MSVVGIDL	1	1	1	1	840	94.3	5.19								
A0A024R2	Guanine de	MCAAQMF	3	1	1	1	454	51	5.68								
A0A068LRV	Ig heavy ch	QVTLRESG	6	1	9	1	127	14	8.15	446.9	414	578	631.1	520.9	521.7	561.7	495.7
O94967	WD repeat	MTAEETVN	1	1	3	1	919	101.9	5.94	140.5	384.9	106.5	191.3	119.3	112.5	111.5	112.6
A8K3H0	cDNA FLJ75	MSLLGPKV	5	1	1	1	173	19.6	5.94								
J3KR56	Receptor a	METGALRF	4	1	4	1	151	16.8	5.48	329.3	439.9	193.7	410.1	402.2	500	457.5	510.1
O75095	Multiple ep	MSFLEEAR	1	1	1	1	1541	161.1	6.38	188.7	164.9	178.7	187.4	194.9	219.6	160.7	178.7
Q06278	Aldehyde c	MDRASELL	1	1	1	1	1338	147.8	7.17	43.4	20.8	15.3	17.6	20.7	21.5	15.2	16.3
A0A0G2JS6	Mucin-4 O!	MKGARWF	0	1	1	1	7418	734.2	5.24								
P35606	Coatomer s	MPLRLDIKI	1	1	1	1	906	102.4	5.27	90	81.5	62.5	73.5	80.8	93.2	72.6	66.5
P16401	Histone H1	MSETAPAE	5	1	1	1	226	22.6	10.92								
A8K8R5	Receptor p	MTSSLQRP	1	1	1	1	1038	115.1	6.2								
Q86SF2	N-acetylga	MRLKIGFIL	1	1	1	1	657	75.3	7.11								
Q96JF0	Beta-galact	MKPHLKQ\	1	1	1	1	529	60.1	9.74	105.2	120.9	73.2	126.2	123.8	134.8	99.8	148
Q9Y3E7	Charged m	MGLFGKTC	4	1	1	1	222	25.1	5.12								
B3KUE5	Phospholip	MGLSGSDN	2	1	4	1	513	56.6	6.64	221.8	269.5	156.5	250.5	233.3	235.6	237.2	232
Q68DC2	Ankyrin rep	MGEGGLPI	1	1	8	1	871	92.2	7.39	910.8	609.8	706.6	1120.5	1042.4	634.6	748.4	647
O60437	Periplakin (	MNSLFRKR	1	1	2	1	1756	204.6	5.6	15.7	15.8	9.9	9	13.1	14.6	14.4	13.4
Q96IY4	Carboxype	MKLCSLAV	2	1	2	1	423	48.4	7.71	46.5	32.7	42	40.9	34.9	53.3	41	27.9
O60241	Adhesion G	MENTGWN	1	1	1	1	1585	172.5	7.42								
P13686	Tartrate-re	MDMWTA	6	1	1	1	325	36.6	8.7								
Q93045	Stathmin-2	MAKTAMA	5	1	1	1	179	20.8	8.32								
Q6IPH7	RPL14 prot	MVFRRFVE	8	1	1	1	220	23.8	10.93								

Q8N0V5	N-acetyllac	MMGSWKI	3	1	1	1	402	45.8	7.18	23	22.7	17.1	23.8	25.7	19.9	16.4	15.5
Q9UK23	N-acetylglu	MATSTGRV	2	1	2	1	515	56	6.6	164.9	155.5	155.1	158.2	151.2	159.5	132.9	148.4
Q05D71	TNFRSF10C	MARIPKTLI	3	1	2	1	277	29.7	8.4	463.1	402.3	316.9	560.5	547.4	541.1	565.9	615.2
A0A096LNU	Uncharacte	MALTKMLI	6	1	6	1	201	23.3	9.38	257.3	160.6	152.7	267	198.8	225.7	151	272.3
Q9UHG3	Prenylcyste	MGRVVAEI	2	1	1	1	505	56.6	6.18	93.5	91.2	76.7	88	86.7	88.3	85.8	96.8
Q96D15	Reticulocal	MMWRPSV	2	1	4	1	328	37.5	4.89	439.9	329.7	295.4	381.3	436.7	413.3	362.4	394.9
A0A0X9T0I	IBM-A2 ligl	DIVMTQSP	8	1	2	1	113	12.2	5.01	70.5	67.8	53.6	55.9	64.1	60.7	92.6	68
Q9H477	Ribokinase	MAASGEPC	2	1	2	1	322	34.1	5.05	31.2	15.1	13	16	24	22.8	16.4	22.4
B3KV71	cDNA FLJ16	MLQLSEFD	1	1	1	1	831	92.6	7.42	79.2	54	137.5	60.9	44.3	55.2	67.7	45.8
Q6UWP8	Suprabasin	MHLARLVC	2	1	1	1	590	60.5	7.01	322.1	431.8	235.8	493.5	343.8	357.5	311.2	321.2
B2R736	cDNA, FLJ9	MATMVPS	2	1	2	1	547	59.5	5.43	100.6	86.3	53.9	74.3	80.8	103.4	78.4	75
Q9P2B2	Prostaglan	MGRLASRF	1	1	1	1	879	98.5	6.61	151.1	136.9	192.4	141.8	142.8	165	119.9	139.1
A2J1N7	Rheumatoi	VESGGGLV	7	1	4	1	96	10.5	9.36	244.2	264.7	236	253	250.6	244.8	276.4	266.9
P12319	High affinit	MAPAMES	3	1	1	1	257	29.6	6.68	107.2	73.3	94.3	99.6	98.7	104.8	95.3	127.2
B3KQP7	cDNA FLJ90	MMTKVLG	2	1	1	1	450	49.7	6.61	94	94.9	122	95.6	94.1	166.6	113.1	105.3
A0A193CH	10E8 light c	SSELTQETG	8	1	1	1	109	11.6	9.2								
Q5JU67	Cilia- and f	MAPKKSVS	4	1	3	1	520	60.5	8.81	41.2	40	36.5	42.3	36	39.4	42.7	31.8
B1PZN7	MHC class	MRVTAPRT	3	1	1	1	362	40.4	6.46								
P09958	Furin OS=H	MELRPWLI	1	1	3	1	794	86.6	6.47								
Q6YHK3	CD109 anti	MQGPPLLT	1	1	1	1	1445	161.6	5.85								
B0LPF3	Growth fac	MEAIAKYD	3	1	1	1	217	25.2	6.32								
Q9BZZ2	Sialoadhes	MGFLPKLL	1	1	1	1	1709	182.5	6.62	101.5	120.2	93.5	101.2	107.5	104.6	89.1	111.3
Q53FP0	Pyridoxine	MTCWLRG	6	1	1	1	261	29.9	7.4	35.5	30.2	26.9	27.5	41.4	30.4	33.1	24.6
A0A024R0T	Apolipopro	MRLFLSLPV	11	1	4	1	83	9.3	8.47	95.6	75.6	74	57.9	71.5	95.9	71.2	52
A0A024RA	Endothelin	MRGVWPP	2	1	1	1	770	87.1	5.88	166.3	175.3	120.2	174.5	180.5	154.8	182.9	207.5
P04424	Argininosu	MASESGKL	2	1	1	1	464	51.6	6.48	125.4	75.4	121.3	103.6	133.6	87.1	110	106.8
Q6ZWJ1	Syntaxin-bi	MNKNTSTV	3	1	2	1	553	61.6	5.2								
A0A024RD	Integrin-bi	MKTALILLS	3	1	1	1	317	35.1	4.18								
P20142	Gastricsin (	MKWMVVV	2	1	2	1	388	42.4	4.46	107.5	137	62.2	150.6	96.6	71	91.8	78.6
Q92542	Nicastrin O	MATAGGG	2	1	1	1	709	78.4	5.99	50.6	32.3	29.2	37.5	37.2	37.7	31	35.1
Q9Y2G1	Myelin reg	MEVVDETE	1	1	1	1	1151	124.3	7.44	108.2	99	77	81.1	90.1	85.7	74.5	91.9
B3KTQ4	Hexosyltra	MASALWTY	3	1	1	1	331	39.4	7.97	184.1	153.1	210.4	144.9	125.8	147.3	122.5	107.7
B6V6K6	Mutant my	MRFFCARC	2	1	2	1	504	57	5.54	273.1	434.3	230.1	302.4	342.3	270.8	270.1	251.9
B2R960	cDNA, FLJ9	MVGVKPVC	2	1	3	1	289	32.2	4.96	162.8	186.9	112.4	160.4	164.5	183.7	151	184.8
B4E3I3	cDNA FLJ59	MFSFNMFI	3	1	1	1	315	35.4	5.72	91.9	67.7	66.8	61.4	62.7	64.8	53.7	61.1
Q14703	Membrane	MKLVNIWI	1	1	1	1	1052	117.7	8.75								
P23471	Receptor-t	MRILKRFLA	1	1	1	1	2315	254.4	4.88	33.9	26.3	35.7	35.2	29.6	28.6	24.3	29.3
Q6P4E1	Protein CA	MVGFGAN	2	1	2	1	433	48.8	5.82								
A0A087WX	ST3 beta-g	MWQAPRE	2	1	1	1	384	43.4	8.92	62.4	35.8	72.8	40.3	56.4	47.6	42.7	45.8
E7EV01	Calpain-5 (	MFSCVKPY	1	1	1	1	680	77.4	7.94								
J3QR18	DNA topois	MIFPVARY	6	1	1	1	139	16.1	10.48								

P05162	Galectin-2	MTGELEVK	6	1	1	1	132	14.6	6.38								
B4DI63	cDNA FLJ5	MLAKISTT	3	1	1	1	356	40.5	7.99	163.4	242.1	171.3	178.4	172.3	186.1	162.8	151.7
P15428	15-hydrox	MHVNGKV	3	1	1	1	266	29	5.86	20	20.2	13.2	18.4	20.9	23.6	18	22.7
P35625	Metallopro	MTPWLGL	3	1	1	1	211	24.1	8.72	280.1	280.9	314	325.4	298.7	320.3	290	269.6
A0A024R1	Follistatin-	MRPGAPG	4	1	1	1	263	27.6	6.77								
Q96FN5	Kinesin-like	MEERGSP	1	1	1	1	646	70.6	8.85								
P62701	40S riboso	MARGPKK	3	1	1	1	263	29.6	10.15	93.9	84.3	66.1	83.4	87.5	96	82.7	83.2
J3QSE8	Fanconi an	MSSMWSE	1	1	1	1	994	112.4	8.21								
P15104	Glutamine	MTTSASSH	2	1	1	1	373	42	6.89	40.6	31.8	16.7	44.4	34.3	39.4	58.3	26.4
K7EJ20	Glutathion	MCASRDD	5	1	3	1	242	26.8	9.31	102.6	88	73.2	81.5	84.1	74.9	62.5	60.1
Q8N5I2	Arrestin do	MGRVQLF	2	1	1	1	433	46	7.02								
F5H056	4F2 cell-su	MLFTLPGT	18	1	2	1	67	7	3.69	70.2	62.3	58.1	69.9	61.8	65.6	50.8	61.4
Q13183	Solute carr	MATCWQA	2	1	1	1	592	64.4	7.01	89.1	64.2	67.7	74.1	85.8	78.8	70.6	82.4
Q9BYW2	Histone-lys	MKQLQPQ	0	1	1	1	2564	287.4	6.14								
B3KUZ7	cDNA FLJ4	MTKMDIR	1	1	3	1	483	55.9	6.48	118.1	102.8	83.6	90.2	122.5	128.6	71.3	86.5
Q9BZJ0	Crooked ne	MTATVENL	1	1	1	1	848	100.4	8								
Q5FWE3	Proline-rich	MASSPWG	1	1	1	1	981	102.1	7.78								
P35318	ADM OS=H	MKLVSVAL	7	1	1	1	185	20.4	10.84								
Q86TD4	Sarcalume	MRALVLLG	1	1	1	1	932	100.7	4.4	99.6	93.1	59.7	76.3	69.1	88	81.3	61.5
Q09328	Alpha-1,6-	MALFTPW	1	1	2	1	741	84.5	8.12	129.8	117.4	108	112.7	140.3	136.2	102.3	100.2
B3KM35	cDNA FLJ1	MGFNLTFH	3	1	1	1	344	40	9.07								
O60637	Tetraspani	MGQCGITS	3	1	2	1	253	28	5.81	395.2	268.3	289	348.9	353.3	333.7	300.9	341.4
A0A024RB	Kit ligand	MKKTQTW	3	1	1	1	273	30.9	6.14	39.5	44.8	29.4	36.3	38.1	44.1	41.1	39.5
A0A140VJL	Testicular t	MGQRPGP	3	1	3	1	336	36.7	7.91	17.9	12.6	15.9	13.5	17.9	19.4	14.6	15.8
Q9H9V9	JmjC doma	MRAGPEP	2	1	2	1	463	52.5	6.21								
A8K690	cDNA FLJ7	MEQVNEL	1	1	2	1	543	62.6	6.8	172.5	157.4	139.5	157.4	163.5	170.8	138.2	142.3
B7Z7X3	cDNA FLJ5	MEERGGV	2	1	6	1	921	102.5	8.29	484.1	715.3	269	437	486.7	602.3	547.3	538.2
A0A0G2JN	Leukocyte	MTLTLSVL	3	1	1	1	643	70.1	6.73								
B7ZLW2	HELZ prote	MEDRRAE	1	1	2	1	1943	219	7.42	15.4	18.6	16.7	23.1	18.1	14.5	12.8	24.3
P48547	Potassium	MGQGDES	3	1	2	1	511	57.9	6.58	25.6	21.6	17.5	20	26.7	30	31	19
O14618	Copper cha	MASDSGN	3	1	1	1	274	29	5.58								
H3BSR6	Fractalkine	MAPISLSW	3	1	1	1	403	42.7	6.52	101.7	88.8	178.5	92.8	99.7	83.8	90.2	81.4
A6NKB5	Pecanex-lik	MVSQVLQ	1	1	1	1	2137	237.1	6.76								
Q8N436	Inactive ca	MSRPGTAT	2	1	1	1	756	85.8	6.87								
Q6UWL6	Kin of IRRE	MLRMRVP	2	1	1	1	708	75	6.93	17	13.8	12.7	12.9	12.6	14.5	12.9	13.9
Q92618	Zinc finger	MDRNREA	1	1	1	1	1163	124.2	8.72								
H9STE0	Cytochrom	MAHAAQV	3	1	1	1	231	25.9	4.82	198.6	183.3	146.3	207.7	168.9	233.6	183.2	204.9
Q96NL6	Sodium cha	MAAEIDFL	1	1	3	1	688	80.9	6.07	297.6	364.1	401.2	291.1	255.7	212.8	257.4	199.9
B2RDG0	Proteasom	MSRRYDSR	3	1	1	1	261	29.4	7.72								
B4DPQ3	cDNA FLJ5	MAAGRRT	2	1	1	1	517	57.4	7.53	28.9	20.1	16.1	24.1	23	24.7	21.3	21.5
O43175	D-3-phosp	MAFANLRK	2	1	1	1	533	56.6	6.71								

H9KV90	SH3 and m	MTHSPATS	0	1	1	1	2169	225.8	8.34	32.4	59.1	19.8	34.2	26.4	25.5	25.7	24.1
Q6ZMJ2	Scavenger	MENKAMY	2	1	1	1	495	54	7.09	80	55.5	57.2	59.4	60.6	63.7	55.6	63.4
Q9BV40	Vesicle-ass	MEEASEGG	8	1	1	1	100	11.4	7.34								
A0A0A0MT	Coiled-coil	MALSPWTI	1	1	2	1	828	97.4	5.99	281.2	262.2	280.7	293.6	264.5	208	192.8	202.2
A0A024R8	Ubiquitinyl	MARMGDS	1	1	2	1	916	102.2	6.21	112.1	107.2	78.8	69.7	85.9	42.1	105.9	41.4
O15162	Phospholip	MDKQNSQ	2	1	1	1	318	35	4.94	205.7	163.4	106.6	181.5	210.2	132.2	135	154.9
A0A0A0MT	TATA-bindi	MGVFRLDF	0	1	2	1	1849	206.8	6.52	150.2	175.6	91.6	143.8	122.6	121.4	128.8	119.7
A6NFX8	ADP-sugar	MESQEPTE	4	1	1	1	232	25.9	5.19	46.1	39.3	35.6	37.6	40.7	36.5	34.2	29.5
Q2M204	HCG16428	MAWQVSL	3	1	1	1	487	54.7	7.61								
H7BXI7	Ly6/PLAUR	MRYKSSDR	8	1	1	1	183	20.7	7.94	25.9	23.7	18.6	31.7	27.9	23.4	30.1	26.6
Q9UKR3	Kallikrein-1	MWPLALV	4	1	1	1	277	30.6	8.46								
D6RH31	Nephronec	MDFLLALV	1	1	3	1	609	66.9	8.4	184	165.7	166	194.7	211.6	215.6	190.8	231.7
O60662	Kelch-like p	MDSQRELA	1	1	2	1	606	68	5.29	206.4	201.9	144.9	184.8	165.7	83.2	100.6	188
A0A087WU	C-C motif c	MKVSEAAL	21	1	3	1	53	5.7	11.55								
Q5HYA8	Meckelin C	MATRGGA	2	1	3	1	995	111.7	6.77	32.6	60.4	41.1	38.3	27.6	23.4	20.2	21.4
Q96AM1	Mas-relate	MAGNCSW	4	1	1	1	343	38.1	8.56	27.7	26.4	25.6	31.8	32.9	30.5	29.3	34.8
B3KQQ3	cDNA PSEC	MTSSGPGP	1	1	1	1	738	84.6	6.2	71.9	51.7	60.1	64.1	70.2	82	62.3	59.6
Q9BQG1	Synaptotag	MSGDYEDI	2	1	1	1	590	63.3	6.62								
O95445	Apolipopro	MFHQIWA	4	1	8	1	188	21.2	6.01	331.8	428.4	231.7	343.3	397.3	419.1	288	354.3

Uniprot Accession Number	protein	Protein amino acid sequence	Protein amino acid sequence matching degree(%)	The number of peptides identified	The total number of times a polypeptide was identified	The number of characteristic peptides identified	Amino acid length	KDa	isoelectric point	relative abundance of normalized protein - sample 113	relative abundance of normalized protein - control sample 121	ratio(113/1
Q4TZM4	Hemoglobi	TPEEKSAVT	89	8	135	2	101	11	6.52	799.4	134.5	5.943494
D9YZU5	Beta-globir	MVHLTPEE	78	10	328	1	147	16	7.28	44720.1	8175.3	5.470148
A0A1K0GX	Globin C1 (	MSAARPG\	50	6	182	6	177	19.2	9.77	29618.2	6799.3	4.356066
Q92743	Serine prot	MQIPRAAL	3	1	1	1	480	51.3	7.83	299.2	74.4	4.021505
A0A024R5I	ADAM met	MVLLRVLIL	1	1	1	1	748	84.1	7.77	371.3	93.2	3.983906
A0A024R8/	Ubiquitinyl	MARMGDS	1	1	2	1	916	102.2	6.21	112.1	41.4	2.707729
P02545	Prelamin-A	METPSQRF	2	1	1	1	664	74.1	7.02	173.5	64.1	2.706708
Q06278	Aldehyde c	MDRASELL	1	1	1	1	1338	147.8	7.17	43.4	16.3	2.662577
Q9Y2S2	Lambda-cr	MASSAAG(	13	4	25	4	319	35.4	6.18	1726.9	653.3	2.643349
A0A161I20	Lactoferrin	MKLVFLVLI	54	34	466	0	711	78.3	8.18	39936.6	15145.7	2.636828
B2R9K8	cDNA, FLJ9	MAAVKTLN	2	1	1	1	531	57.9	6.8	45.5	17.3	2.630058
I3L0A0	HCG20447	MAGAENW	5	2	7	2	370	42.2	6.71	377.8	148.5	2.544108
A0A024R3I	Apolipoprc	MKAAVLTL	60	16	172	16	267	30.8	5.76	14099.8	5583.4	2.525307
C0JYY2	Apolipoprc	MDPPRPAL	1	3	5	3	4563	515.2	7.05	99.2	40.5	2.449383
K7ES00	Histone H3	MARTKQT/	9	2	8	2	151	16.6	11.84	1428.3	602.6	2.370229
A0A1K0FU	Globin E1 (	MLSAQER/	11	1	1	1	141	15.6	6.62	18.4	7.8	2.358974
A0A0A1HA	H.sapiens r	MAYAYLFK	14	2	5	2	212	23.6	6.54	208.1	92.1	2.259501
Q8WWQ8	Stabilin-2 (	MMLQHLV	0	1	1	1	2551	276.8	6.4	26.1	11.6	2.25
B2R4R0	Histone H4	MSGRGKG	50	5	58	5	103	11.4	11.36	3267.7	1469.8	2.223228
Q59EU6	EF hand dc	CEERAARQ	4	1	1	1	265	29.8	6.21	22.4	10.2	2.196078
O75787	Renin rece	MAVFVVLL	5	2	5	2	350	39	6.1	973.9	450.3	2.16278
V9HVY1	Epididymis	MKRMVSW	25	11	29	11	491	55.9	8.27	2437.8	1138.4	2.141427
P24158	Myeloblast	MAHRPPSF	17	3	17	3	256	27.8	8.35	615.9	289.9	2.124526
A0A0U1RR	Histone H2	MSGRGKQ	14	3	13	3	170	18.5	11.52	1265.2	601.7	2.102709
A0A0X9UW	IBM-A3 he	EVQLVQSG	20	2	5	2	127	14.4	8.37	93.6	45	2.08

P01023	Alpha-2-ma	MGKNKLLH	14	17	49	17	1474	163.2	6.46	2846.8	1389.4	2.048942
P08246	Neutrophil	MTLGRRLA	6	2	11	2	267	28.5	9.35	698	343.6	2.031432
Q7L9L4	MOB kinas	MSFLFGSRS	5	1	1	1	216	25.1	6.73	20.6	10.2	2.019608
C9JC84	Fibrinogen	MSWSLHPI	28	9	37	9	461	52.3	5.63	1762.4	884.6	1.992313
A0A024R1:	Glycerol-3-	MASKKVCI	9	3	10	3	349	37.5	6.18	871.9	437.7	1.992004
A0A0X9UW	MS-B1 ligh	DIQMTQSP	32	2	101	1	107	11.5	7.96	22	11.2	1.964286
P13647	Keratin, typ	MSRQSSVS	18	11	34	4	590	62.3	7.74	21.6	11	1.963636
A1A4E9	Keratin 13	MSLRLQSS:	25	14	107	9	458	49.6	4.96	6278.1	3225.2	1.946577
B4DR52	Histone H2	MPDPAKSA	20	3	22	3	166	18	10.32	1928	992.5	1.942569
B2RCC8	cDNA, FLJ9	MFLWLFLII	2	1	1	1	837	94.4	7.97	34.1	17.6	1.9375
P13797	Plastin-3 O	MDEMATT	9	6	20	3	630	70.8	5.6	72.7	38.2	1.903141
Q6PCB0	von Willeb	MLPWTALC	9	3	9	3	445	46.8	7.68	686.2	360.7	1.902412
P24298	Alanine am	MASSTGDF	4	2	2	2	496	54.6	7.18	107.8	57.6	1.871528
A4D0V4	Capping pr	MADLEEQL	9	2	5	1	286	32.9	5.85	38	20.4	1.862745
A2JA16	Anti-mucin	DIQMTQSP	32	2	41	1	107	11.6	8.46	702.5	377.5	1.860927
A0A024R0	Apolipoprc	MRLFLSLPV	11	1	4	1	83	9.3	8.47	95.6	52	1.838462
V9HW91	Epididymis	MTSFRLALI	22	4	7	4	276	30.6	7.21	119.5	65.2	1.832822
A0A075B6:	Protein IGK	MDMRVPA	14	1	2	1	117	12.7	9.25	36.2	20.1	1.800995
S6BAP4	IgG H chair	MDWTWRI	22	4	123	1	275	29.4	8.51	26.2	14.6	1.794521
D3DRR6	Inter-alpha	MKRLTCFFI	5	4	10	4	947	106.5	6.95	446.9	249.7	1.789748
Q9Y3R5	Protein do	MDPEEQEL	0	1	1	1	2298	258.1	6.29	33.4	18.7	1.786096
Q96MU8	Kremen pr	MAPPAARL	3	1	1	1	473	51.7	7.11	48.9	27.5	1.778182
P18085	ADP-ribosy	MGLTISSLF	6	1	1	1	180	20.5	7.14	36.6	20.6	1.776699
B1PS43	Myosin hea	MAQKGQL	1	2	7	2	2029	234.1	5.8	277.4	156.6	1.771392
A0A024R1,	Testicular s	MSSSPLSKK	6	4	8	4	1058	117.8	5.76	235.7	133.1	1.770849
D3DSM8	Formimino	MRSRWGG	2	1	1	1	581	63.3	6.3	46.7	26.4	1.768939
P05164	Myelopero	MGVPFFSS	20	13	48	13	745	83.8	8.97	2474.9	1404.2	1.762498
P08729	Keratin, typ	MSIHFSSPV	9	5	14	2	469	51.4	5.48	137.6	78.3	1.757344
A0A140VK:	Leukotrien	MPEIVDTC:	2	1	1	1	611	69.2	6.18	49.7	28.3	1.756184
A0A087WS	Nucleobinc	MRWRTILL	2	1	2	1	420	50.2	5.12	86.5	49.3	1.754564
P19013	Keratin, typ	MIARQQCV	20	10	34	7	534	57.3	6.61	1337.8	765.5	1.747616
B2RD36	cDNA, FLJ9	MANEAYPC	15	4	17	4	245	28	6.79	1492.3	855.7	1.743952
A0A024RD	Lymphocyt	MARGSVSD	14	8	34	5	627	70.2	5.43	1083.2	623.7	1.736732

Q59ER5	WD repeat	RAGPGGSR	2	1	2	1	624	68.1	7.23	103.8	59.9	1.732888
B3KV71	cDNA FLJ16	MLQLSEFD	1	1	1	1	831	92.6	7.42	79.2	45.8	1.729258
B9EKV4	Aldehyde c	MFLRAGLA	3	1	5	1	518	56.3	6.57	20.2	11.7	1.726496
P04040	Catalase O:	MADSRDPA	9	4	4	4	527	59.7	7.39	208.2	121	1.720661
P29401	Transketola	MESYHKPD	5	2	3	2	623	67.8	7.66	169.1	98.4	1.718496
E7EMK3	Flotillin-2 (	MGNCHTV	5	2	3	2	483	53.1	5.24	47.2	27.6	1.710145
B3KTQ4	Hexosyltra	MASALWT	3	1	1	1	331	39.4	7.97	184.1	107.7	1.709378
B2R6Q2	Alkaline ph	MISPFLVLA	2	1	2	1	524	57.2	6.57	20.5	12	1.708333
A0A024RC	CD99 antig	MVAWRSA	6	1	4	1	262	28	5.14	161.6	94.6	1.708245
K7EJ20	Glutathion	MCASRDD	5	1	3	1	242	26.8	9.31	102.6	60.1	1.707155
P52209	6-phospho	MAQADIAL	7	3	7	3	483	53.1	7.23	65.6	38.5	1.703896
Q9NX62	Inositol mc	MAPMGIRI	3	1	2	1	359	38.7	6.86	22.3	13.2	1.689394
P46781	40S riboso	MPVARSW	5	1	1	1	194	22.6	10.65	156.7	93.2	1.68133
Q6UW88	Epigen OS=	MALGVPIS	8	1	1	1	154	17.1	6.95	84.1	50.2	1.675299
J3KQ18	D-dopachr	MQLFLLAV	23	3	7	3	132	14.2	7.3	368.8	220.3	1.674081
P00915	Carbonic a	MASPDWG	18	4	31	4	261	28.9	7.12	1363.4	816.3	1.670219
M1VPF4	Tyrosine-p	MMEAIKKK	2	1	1	1	725	82.4	4.97	81.7	49	1.667347
Q96IY4	Carboxype	MKLCSLAV	2	1	2	1	423	48.4	7.71	46.5	27.9	1.666667
O95861	3'(2'),5'-bis	MASSNTVL	3	1	1	1	308	33.4	5.69	35.5	21.3	1.666667
V9HWA9	Epididymis	MGPTSGPS	35	55	305	55	1663	187	6.4	17774.3	10679.9	1.664276
A0A0C4DH	Protein IGH	MQFVLSW	9	1	4	1	116	12.8	9.25	329.9	198.3	1.663641
V9HW87	Abhydrolas	MAASVEQI	33	5	33	5	210	22.3	6.4	1439.8	868.9	1.657038
A0A087WY	Seizure 6-li	MGTPRAQI	4	3	7	3	923	99.1	4.87	178	107.6	1.654275
V9GYM3	Apolipoprc	MCEQHPKI	14	2	10	2	133	14.9	8.27	521.7	315.6	1.653042
Q5U043	S-(hydroxy	MANEVIKC	9	3	8	3	374	39.7	7.49	298	183	1.628415
B2R7D2	cDNA, FLJ9	MLRAPGCL	13	5	9	5	487	55.1	7.97	294.3	181.1	1.625069
X6R8A1	Carboxype	MTSSPRAP	20	9	131	9	498	56.2	6.61	9108.3	5613.9	1.622455
Q86TD4	Sarcalumei	MRALVLLG	1	1	1	1	932	100.7	4.4	99.6	61.5	1.619512
P80511	Protein S1(	MTKLEEHL	8	1	5	1	92	10.6	6.25	1210	750.5	1.612258
Q9UKU6	Thyrotropi	MGEDDAA	7	6	14	6	1024	116.9	6.99	567.4	353.4	1.605546
A8K669	cDNA FLJ78	MVWKVAV	4	1	3	1	433	49.4	6.55	48.8	30.4	1.605263
Q0ZCJ2	Immunglob	LVQLVESG	46	5	89	1	124	13.6	7.93	38.8	24.3	1.596708
G3V5Z7	Proteasom	MSRGSSAG	8	2	2	2	252	28.1	6.76	170.1	106.8	1.592697

V9HW90	Epididymis	MALLPRAL	5	2	11	2	522	56.2	8.5	295.1	185.3	1.592553
A0A024R4	Alkaline ph	MQGPWVL	3	2	4	2	528	56.8	5.86	95.8	60.2	1.591362
P02792	Ferritin ligh	MSSQIRQN	18	3	7	3	175	20	5.78	159.7	100.4	1.590637
A0A0K0K1	Epididymis	MAMLRVQ	13	3	8	3	249	28.7	6.02	527.2	331.6	1.589867
P51606	N-acylgluc	MSKGLPAR	3	1	2	1	427	48.8	6.37	88.6	55.8	1.587814
A0A024RB	Solute carr	MMGSPVS	2	1	2	1	540	56.4	6.83	103.6	65.3	1.586524
A0A0R7FJH	Coagulatio	MRALLLLG	6	2	6	2	615	67.8	7.8	132	83.3	1.584634
V9HW48	SH3 domai	MVIRVYIAS	35	3	13	3	114	12.8	5.25	89	56.2	1.58363
P51688	N-sulphogl	MSCPVPAC	11	5	30	5	502	56.7	6.95	917.1	582.5	1.574421
P20160	Azurocidin	MTRLTVLA	14	3	27	3	251	26.9	9.5	960.4	611.6	1.570307
A0A024R6	Galactosyl	MAEWLLSA	1	1	2	1	685	77	6.64	99.9	63.7	1.568289
A6NFX8	ADP-sugar	MESQEPTE	4	1	1	1	232	25.9	5.19	46.1	29.5	1.562712
Q12929	Epidermal	MNGHISNH	4	3	4	3	822	91.8	7.5	155.1	99.3	1.561934
U3KQ56	Glyoxylate	MRPVRLM	5	1	1	1	358	38.7	8.02	18.4	11.8	1.559322
P08263	Glutathion	MAEKPKLH	18	5	78	1	222	25.6	8.88	515.5	331	1.557402
Q7L5L3	Glyceroph	MSLLLYYAL	4	1	1	1	318	36.6	7.97	18.2	11.7	1.555556
Q9UBV8	Peflin OS=H	MASYPYRC	3	1	1	1	284	30.4	6.54	153.7	99.1	1.550959
A8K0T9	cDNA FLJ7	MADFDDRY	17	3	6	2	286	32.9	5.69	370.4	238.9	1.55044
P06727	Apolipopro	MFLKAVVL	54	21	120	21	396	45.4	5.38	8194	5285.4	1.550308
B2RCQ6	cDNA, FLJ9	MPPVGGKI	55	18	112	3	406	44.9	7.24	7840.2	5058.9	1.549784
P12724	Eosinophil	MVPKLFTS	17	2	5	2	160	18.4	10.02	120.6	77.9	1.548139
P05787	Keratin, ty	MSIRVTQK	18	10	40	6	483	53.7	5.59	585.3	378.4	1.546776
Q14117	Dihydropy	MAAPSRLL	3	2	6	2	519	56.6	7.27	201.8	130.9	1.541635
B3KWI4	cDNA FLJ4	MPLKHYLL	6	3	9	3	581	64.4	6.71	186.2	120.9	1.540116
Q00796	Sorbitol de	MAAAAKPI	15	5	11	5	357	38.3	7.97	686.6	446.2	1.538772
P15104	Glutamine	MTTSASSH	2	1	1	1	373	42	6.89	40.6	26.4	1.537879
P55786	Puromycin	MWLAAAA	4	3	3	3	919	103.2	5.72	36.4	23.7	1.535865
P14174	Macrophag	MPMFIVNT	8	1	30	1	115	12.5	7.88	2360.8	1538	1.53498
P11234	Ras-relatec	MAANKSK	7	1	2	1	206	23.4	6.62	29.3	19.1	1.534031
Q53Y06	ATPase, H+	MALSDAD	4	1	4	1	226	26.1	8	222.5	145.2	1.532369
P59665	Neutrophil	MRTLAILA	19	2	29	2	94	10.2	6.99	2669.7	1746.8	1.528338
E9KL23	Epididymis	MPSSVSW	53	24	660	1	418	46.7	5.59	2619.7	1716	1.526632
A0A024RA	Betaine-ho	MAPAGRP	31	9	47	5	363	40.3	5.87	337.8	221.3	1.526435

Q5HYA8	Meckelin C	MATRGGA(	2	1	3	1	995	111.7	6.77	32.6	21.4	1.523364
Q7Z4W1	L-xylulose ı	MELFLAGR	4	1	3	1	244	25.9	8.1	245.2	161	1.522981
B1AKK2	Dimethylar	MAGLGHP/	11	3	20	2	285	31.1	5.81	361.4	237.8	1.519765
A0A140VK/	Testis secr	MDPRKVNI	8	3	7	3	369	41.3	5.27	306	201.6	1.517857
Q8N7G1	Purine nuc	MENGYTYE	19	5	11	5	293	32.5	7.21	184.8	121.9	1.515997
B4DMS4	cDNA FLJ5!	MGKLSRCS	2	2	4	2	812	88.4	4.4	558.8	369.1	1.513953
Q9HB40	Retinoid-in	MELALRRS	6	3	41	3	452	50.8	5.81	1368.6	904.6	1.512934
A0A0B4J1F	4-hydroxyp	MTTYSDKG	12	4	4	4	393	44.9	7.01	135.8	90	1.508889
Q9UHY7	Enolase-ph	MVVLSVPA	5	1	1	1	261	28.9	4.78	17.8	11.8	1.508475
P23526	Adenosylh	MSDKLPYK	19	8	23	8	432	47.7	6.34	1319.8	875	1.508343
A0A024QZ	NAD(P)H d	MAGKKVLI	4	1	1	1	231	25.9	6.29	99.8	66.2	1.507553
B3KUI5	Hyaluronid	MAAHLLPI(	18	5	27	5	435	48.3	6.96	879.3	584	1.505651
Q96KP4	Cytosolic n	MAALTTLFI	24	8	29	8	475	52.8	5.97	933.4	620	1.505484
O14786	Neuropilin-	MERGLPLL(	2	1	4	1	923	103.1	5.88	17	11.3	1.504425
B4E3I3	cDNA FLJ5!	MFSFNMFI	3	1	1	1	315	35.4	5.72	91.9	61.1	1.504092
Q9HC38	Glyoxalase	MAARRALH	6	2	9	2	313	34.8	5.6	498.6	331.6	1.503619
B2RAN2	cDNA, FLJ9	MTTQLPAY	4	2	5	2	513	57	5.57	354.5	235.9	1.502755
P63261	Actin, cyto	MEEEIAAL\	42	14	229	5	375	41.8	5.48	16761	11164.5	1.501276
P02750	Leucine-ric	MSSWSRQ	28	9	294	9	347	38.2	6.95	12613	19100	0.660366
Q96TA2	ATP-depen	MFSLSSTV(	1	1	1	1	773	86.4	8.76	108.6	165.7	0.655401
J3KR56	Receptor a	METGALRF	4	1	4	1	151	16.8	5.48	329.3	510.1	0.64556
B2R7Z2	cDNA, FLJ9	MPRQLSA/	5	2	3	2	416	44.3	8.41	183.6	286.3	0.641285
B7ZLW2	HELZ prote	MEDRRAEI	1	1	2	1	1943	219	7.42	15.4	24.3	0.633745
P55000	Secreted L	MASRWAV	61	3	21	3	103	11.2	5.33	419.5	663.4	0.632349
Q9Y5Y7	Lymphatic	MARCFSLV	18	6	249	6	322	35.2	8.28	13497.1	22274.4	0.605947
Q9UBC9	Small proli	MSSYQQK(	71	8	121	8	169	18.1	8.57	5390.1	9233.8	0.583736
P31151	Protein S1(	MSNTQAEI	34	3	35	3	101	11.5	6.77	956.5	1722.6	0.555265
A0A0S2Z4I	Secretoglo	MKLAVTLT	20	3	114	3	91	10	5.06	5373.7	13735	0.391241

Uniprot Accession Number	protein	Protein amino acid sequence	Protein amino acid sequence matching degree(%)	The number of peptides identified	The total number of times a polypeptide was identified	The number of characteristic peptides identified	Amino acid length	KDa	isoelectric point	relative abundance of normalized protein - sample 114	relative abundance of normalized protein - control sample 121	ratio(114/1
Q16661	Guanylate	MGCRAAS	12	1	9	1	112	12.1	6.48	255.4	58.1	4.395869
P36980	Compleme	MWLLVSVI	26	6	33	3	270	30.6	6.38	423.3	120.1	3.524563
Q03164	Histone-ly	MAHSCRW	0	1	10	1	3969	431.5	9.09	2094.7	606.1	3.45603
O94967	WD repeat	MTAEETV	1	1	3	1	919	101.9	5.94	384.9	112.6	3.418295
B4E0X1	Beta-2-mic	MSRSVALA	27	4	110	4	122	13.9	7.44	27383.9	8163.5	3.354431
Q5HYA8	Meckelin C	MATRGGA	2	1	3	1	995	111.7	6.77	60.4	21.4	2.82243
Q9Y3R5	Protein do	MDPEEQEL	0	1	1	1	2298	258.1	6.29	52.7	18.7	2.818182
A8K7G6	cDNA FLJ7	MAQTSSYF	55	9	294	7	166	18.7	5.53	67081.8	24072.4	2.786669
Q9UL81	Myosin-re	DIQMTQSP	51	3	104	2	107	11.5	8.48	62.8	23.2	2.706897
P13797	Plastin-3 O	MDEMATT	9	6	20	3	630	70.8	5.6	101.6	38.2	2.659686
A0A024R8	Ubiquitinyl	MARMGDS	1	1	2	1	916	102.2	6.21	107.2	41.4	2.589372
S6BAP4	IgG H chair	MDWTWR	22	4	123	1	275	29.4	8.51	36.7	14.6	2.513699
A0A075B6	Ig lambda-	XQPKAAPS	48	4	1262	1	106	11.3	8.29	314.6	126.3	2.490895
H9KV90	SH3 and m	MTHSPATS	0	1	1	1	2169	225.8	8.34	59.1	24.1	2.452282
Q6ICS1	Putative ur	MAQTNSF	24	3	114	1	166	18.7	5.95	68.7	30.7	2.237785
P81605	Dermcidin	MRFMTLLF	10	1	10	1	110	11.3	6.54	1243.9	563.4	2.207845
Q02747	Guanylin O	MNAFLLSA	18	3	55	3	115	12.4	4.59	6099.4	2797.1	2.180616
H3BQD0	Cerebellin-	MLGVLELL	11	1	8	1	140	14.7	5.48	722.6	333	2.16997
P02753	Retinol-bin	MKWVWA	61	10	470	10	201	23	6.07	58084.3	26946	2.155582
Q9ULL1	Pleckstrin	MELSDSDR	1	1	8	1	1385	155.3	6.24	833.4	396.8	2.100302
A0A075B6	Protein IG	MDMRVPA	14	1	2	1	117	12.7	9.25	41.8	20.1	2.079602
V9HW48	SH3 domai	MVIRVYIA	35	3	13	3	114	12.8	5.25	114.7	56.2	2.040925
Q15828	Cystatin-M	MARSNLPL	50	5	278	5	149	16.5	8.09	14974.4	7481.9	2.001417
Q9Y546	Leucine-ric	MSYYLSSE	3	1	6	1	428	48.5	7.46	771.1	408	1.889951
B2RAF9	Suppresso	MGSDRAR	2	1	1	1	855	94.7	6.52	61.9	33.1	1.870091

P01704	Immunogl	MAWALLLI	13	1	5	1	120	12.6	6.49	454.4	246.2	1.845654
A0A087WU	ADP-ribosy	MSDLRITEA	6	1	1	1	204	22.9	5.63	58.9	32	1.840625
Q96NL6	Sodium ch	MAAEIDFLI	1	1	3	1	688	80.9	6.07	364.1	199.9	1.821411
P00915	Carbonic a	MASPDWG	18	4	31	4	261	28.9	7.12	1485.8	816.3	1.820164
Q9NWV4	UPF0587 p	MGKIALQL	16	2	3	2	160	18	5.01	373.2	206.9	1.80377
B4E1Z4	cDNA FLJ5	MGPLMVLI	18	17	125	3	1266	140.9	7.18	7433.3	4174.9	1.780474
A0A0X9UW	MS-B1 ligh	DIQMTQSF	32	2	101	1	107	11.5	7.96	19.9	11.2	1.776786
Q86YT9	Junctional	MFCPLKLIL	5	2	3	2	394	44.3	7.12	180	101.9	1.766438
Q9BUD6	Spondin-2	MENPSPAA	5	2	2	2	331	35.8	5.52	153	86.9	1.760644
P13521	Secretogra	MAEAKTHV	2	1	2	1	617	70.9	4.75	232.1	132.3	1.754346
Q86SQ8	Beta-defen	GNFLTGLG	21	1	13	1	47	5.1	8.73	470.9	268.5	1.753818
A2NUT2	Lambda-ch	MTCSPLLLT	38	9	1468	3	235	24.6	7.62	177227.2	101417.1	1.747508
P20142	Gastricsin (	MKWMVV	2	1	2	1	388	42.4	4.46	137	78.6	1.743003
B6V6K6	Mutant my	MRFFCARC	2	1	2	1	504	57	5.54	434.3	251.9	1.724097
A2N2F4	VK3 protei	LTQSPGTLS	8	1	15	1	108	11.9	9.51	491.3	285.9	1.718433
B2R4R0	Histone H4	MSGRGKG	50	5	58	5	103	11.4	11.36	2510.2	1469.8	1.707851
K7ES00	Histone H3	MARTKQTA	9	2	8	2	151	16.6	11.84	1016.6	602.6	1.687023
A0A1K0FU	Globin E1 (	MLSAQERA	11	1	1	1	141	15.6	6.62	13.1	7.8	1.679487
A0A0U1RR	Histone H2	MSGRGKQ	14	3	13	3	170	18.5	11.52	1004.6	601.7	1.669603
P07737	Profilin-1 C	MAGWNAY	63	7	73	7	140	15	8.27	4969.7	2994	1.659886
Q86YD5	Low-densit	MWLLGPL(	5	1	1	1	345	37.4	4.87	17.7	10.8	1.638889
Q5FWF9	IGL@ prote	MAWTPLLI	42	8	690	3	232	24.8	5.59	5027.3	3111.9	1.615508
P06727	Apolipopro	MFLKAVVL	54	21	120	21	396	45.4	5.38	8534.3	5285.4	1.614693
Q6IB39	RNASE6 pr	MVLCFPLLI	19	2	12	2	150	17.2	8.76	500.5	310.4	1.612436
A8K6Z6	cDNA FLJ7	MKPNIIFVL	43	21	144	15	582	65.6	9.07	4746.6	2968.1	1.599205
B4DI63	cDNA FLJ5	MLAKISTTV	3	1	1	1	356	40.5	7.99	242.1	151.7	1.595913
O94991	SLIT and N	MHTCCPPV	1	1	4	1	958	107.4	6.95	156.6	98.7	1.586626
A0A024R8	Prostaglan	MATHHTLV	41	6	3425	6	190	21	7.8	249501.5	157265.4	1.5865
B2R7Z2	cDNA, FLJ9	MPRQLSAA	5	2	3	2	416	44.3	8.41	453.3	286.3	1.583304
C0JYY2	Apolipopro	MDPPRPAL	1	3	5	3	4563	515.2	7.05	64	40.5	1.580247
P02763	Alpha-1-ac	MALSWVLT	40	7	965	5	201	23.5	5.02	76375	48390.7	1.578299
P04279	Semenogel	MKPNIIFVL	53	23	236	17	462	52.1	9.29	19411.5	12317.9	1.575877
Q6GMV8	Uncharacte	MAWTVLLI	42	8	1402	1	234	24.9	6.67	6709.7	4273.6	1.570035

A8K0T9	cDNA FLJ7!	MADFDDR'	17	3	6	2	286	32.9	5.69	374.5	238.9	1.567602
B4E1C2	Kininogen :	MKLITILFL(	36	27	3259	1	644	71.9	6.81	263.2	167.9	1.5676
P25311	Zinc-alpha-	MVRMVPV	60	20	1734	20	298	34.2	6.05	236874.5	151729.7	1.561161
P29401	Transketol:	MESYHKPD	5	2	3	2	623	67.8	7.66	153.2	98.4	1.556911
Q0ZCJ2	Immunglol	LVQLVESG(	46	5	89	1	124	13.6	7.93	37.4	24.3	1.539095
B0YIW2	Apolipoprc	MGTWGAF	29	3	12	3	117	12.8	8.18	823	535.4	1.537168
P02545	Prelamin-A	METPSQRF	2	1	1	1	664	74.1	7.02	98.4	64.1	1.535101
B2R888	Monocyte	MERASCLL	35	12	411	12	375	40	6.23	34324.4	22517.1	1.52437
A0A024RC:	CD99 antig	MVAWRSA	6	1	4	1	262	28	5.14	144.2	94.6	1.524313
A0A024R9.	Serpin pep	MYSNVIGT	36	16	193	16	464	52.6	6.71	9396.9	6197	1.516363
B7Z8Q2	cDNA FLJ5!	MFAGCFFF	24	10	409	10	433	46.6	6.28	47211	31137.6	1.516205
P30101	Protein dis	MRLRRLAL	2	1	2	1	505	56.7	6.35	50	33	1.515152
Q86TD4	Sarcalumei	MRALVLLG	1	1	1	1	932	100.7	4.4	93.1	61.5	1.513821
A6XMV9	Protease s	MNLLLILTF	15	3	5	1	261	28	5.14	144	95.5	1.507853
P59665	Neutrophil	MRTLAILA/	19	2	29	2	94	10.2	6.99	2632.3	1746.8	1.506927
P46781	40S ribosoi	MPVARSW	5	1	1	1	194	22.6	10.65	139.9	93.2	1.501073
Q400G7	B and T lyn	MKTLPAMI	7	1	4	1	289	32.8	6.34	71	106.5	0.666667
A0A024R6I	Delta-like 1	MTATEALL	11	3	41	3	383	41.2	5.53	4701.3	7066.9	0.665256
P09564	T-cell antig	MAGPPRLL	10	2	36	2	240	25.4	7.27	178.6	271.3	0.658312
Q6N030	Uncharact	MDWTWRI	37	15	1075	6	518	57	8.05	4738.1	7211.3	0.657038
P13645	Keratin, ty	MSVRYSSI	31	18	186	13	584	58.8	5.21	7528.5	11461.9	0.656828
M1VKI3	Tyrosine-p	MAPARLFA	7	4	46	4	746	83.1	4.97	1882.6	2867	0.656645
Q05D71	TNFRSF10(	MARIPKTLI	3	1	2	1	277	29.7	8.4	402.3	615.2	0.653934
Q68CJ9	Cyclic AMP	MNTDLAA(	7	2	39	2	461	49	5.15	1292.7	1983.8	0.651628
Q5T1S8	Noncompa	MTTATPLG	7	1	16	1	102	11.1	9.86	1819.3	2795.3	0.650842
P35908	Keratin, ty	MSCQISCK!	17	11	66	5	639	65.4	8	575.3	884.5	0.650424
Q5T2L0	V-set dom:	MVLLTSRN	6	2	18	2	285	31.2	5.99	624.6	960.5	0.650286
P02743	Serum amy	MNKPLLWI	16	3	18	3	223	25.4	6.54	727.3	1124.2	0.646949
Q59FG9	Chondroiti	IFPPNCNKF	1	3	13	2	3410	374.1	4.53	334.5	517.4	0.646502
Q9HC84	Mucin-5B (	MGAPSACF	2	4	11	4	5762	596	6.64	529.5	821.2	0.644788
P13473	Lysosome-	MVCFRLFP	7	3	113	3	410	44.9	5.63	5745.4	8958.6	0.641328
P01258	Calcitonin (	MGFQKFSF	13	2	2	2	141	15.5	6.52	256.4	403.3	0.635755
Q6LAM1	Heavy chai	KVTYTSQEI	45	12	123	1	321	35.9	7.59	172.8	272.9	0.633199

P05543	Thyroxine-	MSPFLYLVI	31	13	123	13	415	46.3	6.3	6472.1	10225	0.632968
B5TMG5	Histidine ri	MGHHRPW	1	1	5	1	750	86.2	4.75	401.1	636.8	0.629868
Q6UX06	Olfactome	MRPGLSFL	21	10	125	10	510	57.2	5.69	4025.9	6418.7	0.627214
A0A024R2	Cysteine-ri	MAPWPPK	5	2	4	2	420	45.4	4.87	33.9	54.1	0.626617
Q99835	Smoothen	MAAARPAI	1	1	4	1	787	86.3	8.34	222.3	361.7	0.614598
O43653	Prostate st	MKAVLLAL	22	2	164	2	123	12.9	5.29	8903	14644.6	0.607937
P51170	Amiloride-	MAPGEKIK	4	2	8	2	649	74.2	7.5	270.6	446.9	0.605505
P04746	Pancreatic	MKFFLLLFT	53	20	829	3	511	57.7	7.05	1554.5	2613.9	0.594705
A0A096LN	Uncharacte	MALTKMLI	6	1	6	1	201	23.3	9.38	160.6	272.3	0.589791
J3KPA1	Cysteine-ri	MKQILHPA	13	2	12	2	276	31	7.61	678.2	1153.4	0.588001
Q6N092	Putative ur	GEEPSTRSF	34	14	1008	1	519	56.4	6.93	170.6	292.1	0.584047
A0A024RD	Secreted p	MRIAVICF	50	13	1136	2	314	35.4	4.58	41336.7	70922.1	0.582847
P12319	High affinit	MAPAMES	3	1	1	1	257	29.6	6.68	73.3	127.2	0.576258
B2RDY9	Adenylyl c	MADMQNI	4	2	2	2	475	51.6	8.22	156.3	273.4	0.57169
Q8NG20	Plasminog	MSEGNSD	14	2	6	2	90	9.8	7.14	103.2	180.8	0.570796
P24855	Deoxyribor	MRGMKLL	23	5	348	5	282	31.4	4.91	10550.1	19469.6	0.541876
Q96TA2	ATP-depen	MFSLSSTV	1	1	1	1	773	86.4	8.76	85.8	165.7	0.517803
B2R7D1	cDNA, FLJ9	MMDASKE	2	2	7	2	684	77.1	6.76	20.2	39.4	0.51269
A0A1B0GU	Uncharacte	MYGDIFNA	13	1	1	1	103	10.9	8.92	14.2	27.8	0.510791
Q5IWS5	Intelectin 1	MNQLSFLL	16	4	13	4	313	35	5.82	403	793.9	0.507621
Q13790	Apolipopro	MTGLCGYS	4	1	6	1	326	35.4	5.64	116.9	235.1	0.497235
Q8N4F0	BPI fold-co	MAWASRL	7	3	15	3	458	49.1	8.72	482	975	0.494359
X6RBG4	Uromoduli	MRHIMTRI	39	23	3450	23	689	75.6	5.87	169355	364524.4	0.464592
P31151	Protein S1	MSNTQAE	34	3	35	3	101	11.5	6.77	741.2	1722.6	0.43028
Q96AX9	E3 ubiquiti	MGWKPSE	1	1	4	1	1013	109.9	8.44	28.5	88.1	0.323496

Uniprot Accession Number	protein	Protein amino acid sequence	Protein amino acid sequence matching degree(%)	The number of peptides identified	The total number of times a polypeptide was identified	The number of characteristic peptides identified	Amino acid length	KDa	isoelectric point	relative abundance of normalized protein - sample 115	relative abundance of normalized protein - control sample 121	ratio(115/1
P02768	Serum albu	MKWVTFIS	73	56	9971	56	609	69.3	6.28	2220421	666397.2	3.331978
B3KV71	cDNA FLJ1(	MLQLSEFD	1	1	1	1	831	92.6	7.42	137.5	45.8	3.002183
A0A024R6I	Alpha-1-an	MPSSVSW(	53	24	629	1	418	46.7	5.59	80233.1	30932.8	2.593787
B2R6Q2	Alkaline ph	MISPFLVLA	2	1	2	1	524	57.2	6.57	30.9	12	2.575
B4E1B2	cDNA FLJ5:	MRLAVGAL	62	43	1716	3	678	74.8	7.12	199640.4	78511.8	2.542808
A0A0K0K1I	Epididymis	MRLAVGAL	58	42	1721	2	698	77	7.21	8496.1	3358.1	2.530032
A0A1B1CY(	Vitamin D I	LAERLKAKL	32	1	8	1	34	3.7	8.56	867.1	346.4	2.503176
E9KL23	Epididymis	MPSSVSW(	53	24	660	1	418	46.7	5.59	4131.1	1716	2.407401
A8K669	cDNA FLJ78	MVWKVAV	4	1	3	1	433	49.4	6.55	69.4	30.4	2.282895
H3BSR6	Fractalkine	MAPISLSW	3	1	1	1	403	42.7	6.52	178.5	81.4	2.192875
A0A024QZ	NAD(P)H d	MAGKKVLI	4	1	1	1	231	25.9	6.29	137.6	66.2	2.07855
Q96NL6	Sodium cha	MAAEIDFLI	1	1	3	1	688	80.9	6.07	401.2	199.9	2.007004
B3KTQ4	Hexosyltra	MASALWT	3	1	1	1	331	39.4	7.97	210.4	107.7	1.953575
A0A024R3I	Apolipoprc	MKAAVLTL	60	16	172	16	267	30.8	5.76	10752.4	5583.4	1.92578
Q5HYA8	Meckelin C	MATRGGA(	2	1	3	1	995	111.7	6.77	41.1	21.4	1.920561
A0A024R8/	Ubiquitinyl	MARMGDS	1	1	2	1	916	102.2	6.21	78.8	41.4	1.903382
A2NZ55	Variable im	QVQLQESG	31	3	66	1	131	13.9	8.79	193.1	102.9	1.876579
P13688	Carcinoem	MGHLSAPL	5	2	27	1	526	57.5	5.97	145	77.3	1.875809
P30039	Phenazine	MKLPIFIAD	10	2	5	2	288	31.8	6.52	286.2	152.6	1.875491
V9HWA9	Epididymis	MGPTSGPS	35	55	305	55	1663	187	6.4	19834.7	10679.9	1.857199
D3DRR6	Inter-alpha	MKRLTCFFI	5	4	10	4	947	106.5	6.95	459.8	249.7	1.84141
Q9Y3R5	Protein do	MDPEEQEL	0	1	1	1	2298	258.1	6.29	33.8	18.7	1.807487
P02545	Prelamin-A	METPSQRF	2	1	1	1	664	74.1	7.02	115.4	64.1	1.800312
A0A024R9	Serpin pep	MYSNVIGT	36	16	193	16	464	52.6	6.71	10940.4	6197	1.765435
A6NL88	Protein shi	MPALLLLVI	2	1	1	1	538	56.2	10.02	133	75.4	1.763926

D6RF35	Vitamin D-	MKRVLVLL	53	23	262	23	476	53	5.52	24489.9	14294.4	1.713251
A0A1K0FU	Globin E1 (	MLSAQERA	11	1	1	1	141	15.6	6.62	13.3	7.8	1.705128
P30047	GTP cycloh	MPYLLISTC	40	2	23	2	84	9.7	6.54	400.7	235.6	1.700764
Q8WWQ8	Stabilin-2 (	MMLQHLV	0	1	1	1	2551	276.8	6.4	19.7	11.6	1.698276
B4E1C2	Kininogen :	MKLITILFL(	36	27	3259	1	644	71.9	6.81	278	167.9	1.655747
V9HW48	SH3 domai	MVIRVYIAS	35	3	13	3	114	12.8	5.25	92.2	56.2	1.640569
F1T0L0	LY6/PLAUR	MRGTRLAL	11	1	8	1	125	13.1	5.96	474.9	291.3	1.630278
A0A075B6	Protein IGk	MDMRVPA	14	1	2	1	117	12.7	9.25	32.6	20.1	1.621891
A0N5G7	Rheumatoi	GGGVVQP(	9	1	1	1	119	13	8.94	141.9	88.1	1.61067
P43652	Afamin OS=	MKLLKLTGI	21	13	95	13	599	69	5.9	9319.1	5833.9	1.597405
P51606	N-acylgluc	MSKGLPAR	3	1	2	1	427	48.8	6.37	88.8	55.8	1.591398
C0JYY2	Apolipopro	MDPPRPAL	1	3	5	3	4563	515.2	7.05	64.4	40.5	1.590123
A0A087W	ST3 beta-g	MWQAPRE	2	1	1	1	384	43.4	8.92	72.8	45.8	1.58952
S6BAP4	IgG H chair	MDWTWRI	22	4	123	1	275	29.4	8.51	23.2	14.6	1.589041
A0A125U0	MS-C3 ligh	DIQMTQSP	17	1	1	1	106	11.4	4.83	155.7	98.8	1.575911
Q96KP4	Cytosolic n	MAALTTLFI	24	8	29	8	475	52.8	5.97	968.5	620	1.562097
P18085	ADP-ribosy	MGLTISSLF	6	1	1	1	180	20.5	7.14	32.1	20.6	1.558252
P12724	Eosinophil	MVPKLFTS(	17	2	5	2	160	18.4	10.02	119.5	77.9	1.534018
A0A125U0	GCT-A2 hea	EVQLVESG(	15	1	18	1	126	13.8	5.45	176.5	115.8	1.52418
P68371	Tubulin bet	MREIVHLQ	11	4	10	4	445	49.8	4.89	35.9	23.6	1.521186
Q96IY4	Carboxype	MKLCSLAV	2	1	2	1	423	48.4	7.71	42	27.9	1.505376
A0A0X9UV	MS-B1 ligh	DIQMTQSP	32	2	101	1	107	11.5	7.96	16.8	11.2	1.5
P49908	Selenoprot	MWRSLGL/	4	2	12	2	381	43.2	7.87	674.7	1007.4	0.669744
Q8NES3	Beta-1,3-N	MLKRCGRF	7	1	2	1	379	41.7	9.17	31.8	47.5	0.669474
P22792	Carboxype	MLPGAWL	22	11	195	11	545	60.5	5.99	6103.8	9129.5	0.66858
B1AH90	Signal pept	MGAAAVR	9	2	5	2	347	37.3	5.71	79.4	118.8	0.66835
Q53H88	Dynactin 2	MADPKYAI	2	1	4	1	406	44.8	5.12	67.6	101.2	0.667984
B7ZW00	COL6A3 pr	MRKHRHLF	6	14	120	1	2570	278	8.15	576.2	862.7	0.667903
O15144	Actin-relat	MILLEVNNI	8	2	2	2	300	34.3	7.36	58.1	87	0.667816
Q66K79	Carboxype	MPPPLPLLI	3	2	29	2	652	73.6	7.97	1403.2	2103.6	0.667047
X5D7A8	Cell adhesi	MASVVLPS	12	5	92	5	471	51.5	4.87	3144.4	4714.4	0.666978
Q8WW52	Protein FAI	MVCREQLS	7	4	17	4	585	64	6.67	567	851.1	0.666197
P00995	Serine prot	MKVTGIFLI	54	4	91	4	79	8.5	7.61	3072.8	4616.6	0.665598

A0A075B6.	Immunoglo	MAWALLLL	25	2	14	2	118	12.4	4.82	755.2	1136.9	0.664262
B2R701	cDNA, FLJ9	MVELHNLY	16	6	45	1	428	45.7	5.38	20.1	30.3	0.663366
Q99988	Growth/dif	MPGQELRT	8	2	19	2	308	34.1	9.66	867.2	1308.6	0.662693
H0YMD1	Low-densit	MGPWGW	2	2	13	2	948	104.7	5.5	819.6	1237.7	0.662196
M9MML0	Fc of IgG lo	MGGGAGE	18	6	107	2	290	32.7	8.1	1408.9	2128.2	0.662015
P04118	Colipase O:	MEKILILLLV	7	1	9	1	112	11.9	7.5	513.9	777.9	0.660625
A8K6Q6	cDNA FLJ78	MLRRRGSP	14	3	13	3	534	57.3	4.91	553	837.3	0.660456
Q7Z3B1	Neuronal g	MDMMLLV	42	11	177	11	354	38.7	6.21	3566.5	5405.1	0.65984
Q6P5S8	IGK@ prote	METPAQLL	47	8	2911	1	236	25.8	6.33	121290.3	183839.5	0.659762
O00526	Uroplakin-2	MAPLLPIRT	16	2	4	2	184	19.4	10.4	112	169.9	0.659211
P06703	Protein S10	MACPLDQA	24	3	32	3	90	10.2	5.48	1732.2	2629.1	0.658857
P36957	Dihydrolipo	MLSRSRCV	7	3	60	3	453	48.7	8.95	3714.7	5668.5	0.655323
A9UFC0	Caspase 14	MSNPRSLE	14	4	10	4	242	27.6	5.34	178.4	272.4	0.654919
Q14982	Opioid-bin	MGVCGYLF	14	4	49	3	345	38	6.87	1148.8	1754.3	0.654848
A0A0X9V9	IBM-B2 ligh	EIVMTQSPA	8	1	6	1	106	11.4	6.51	191.9	293.4	0.654056
O95445	Apolipopro	MFHQIWA	4	1	8	1	188	21.2	6.01	231.7	354.3	0.653966
A8K7G6	cDNA FLJ75	MAQTSSYF	55	9	294	7	166	18.7	5.53	15740.4	24072.4	0.653877
Q14508	WAP four-d	MPACRLGP	61	4	231	4	124	13	4.84	8609.3	13168.2	0.653795
A8K6V6	N-acetylglu	MRLLPLAP	25	13	292	13	552	62.1	8.4	9677.9	14806.6	0.653621
P08138	Tumor nec	MGAGATG	9	3	96	3	427	45.2	4.7	4209.4	6441.7	0.653461
A8K7T4	cDNA FLJ75	MAAEGWI	47	16	724	16	356	40.2	6.95	37340.5	57175.3	0.653088
A8K2P8	cDNA FLJ76	MEAARPSG	17	14	99	14	901	99.9	5.34	3005.9	4611.3	0.651855
A0A0E3XJU	E-cadherin	MGPWSRS	17	14	408	13	882	97.3	4.7	19631.3	30186.4	0.650336
A0A0G2JLV	Leukocyte-	MSPHPTAL	16	5	135	2	287	31.5	5.78	7112.7	10954.7	0.649283
M1VKI3	Tyrosine-pr	MAPARLFA	7	4	46	4	746	83.1	4.97	1857.1	2867	0.64775
Q8TCT8	Signal pept	MGPQRRLS	3	1	8	1	520	58.1	8.32	225.6	348.3	0.647717
A0A024R5I	Folate rece	MAQRMTT	31	7	206	7	257	29.8	7.97	8645.7	13354.9	0.64738
Q9NQ36	Signal pept	MGVAGRN	3	2	7	2	999	109.9	6.65	445.5	688.2	0.647341
P82980	Retinol-bin	MPPNLTGY	7	1	9	1	135	15.9	6.54	453.8	701.1	0.647269
Q15485	Ficolin-2 O	MELDRAVG	16	6	22	6	313	34	6.77	1371.5	2120.4	0.646812
Q6UXB8	Peptidase i	MHGSCSFL	17	7	121	2	463	49.4	5.39	3273.1	5069.6	0.645633
P13647	Keratin, typ	MSRQSSVS	18	11	34	4	590	62.3	7.74	7.1	11	0.645455
A0A087WV	Neural cell	MLQTKDLI	11	9	123	9	884	97.3	4.93	4723.8	7327.9	0.644632

Q7KYR7	Butyrophili	MESAAALH	8	6	68	6	527	59.6	6.48	4426.9	6869.3	0.644447
H7C2N1	Prothymos	XSPGLRPP(	7	1	8	1	148	15.8	4.23	314.9	488.7	0.644363
B2R907	cDNA, FLJ9	MSDSKEPR	9	3	4	3	404	45.7	5.3	223.3	346.7	0.644073
P05091	Aldehyde c	MLRAAARF	4	3	17	1	517	56.3	7.05	286.8	445.7	0.643482
A0A0X9UW	GCT-A5 ligl	DIVMTQSP	54	5	236	2	113	12.5	6.58	6619.9	10309.4	0.642123
Q6N092	Putative ur	GEEPSTRSF	34	14	1008	1	519	56.4	6.93	187.3	292.1	0.641219
Q7Z5F5	Liver-expre	MWHLKLC/	25	2	27	2	81	9.3	10.01	1508.1	2352.3	0.641117
Q7L0X0	TLR4 intera	MEAARALF	1	1	4	1	811	88.7	9.6	115.2	179.7	0.641068
Q6LAM1	Heavy chai	KVTYTSQEI	45	12	123	1	321	35.9	7.59	174.9	272.9	0.640894
D3DNU8	Kininogen :	MKLITILFL(	52	28	3345	2	427	47.8	6.65	193831.3	302648.3	0.640451
A0A024QZ	Prosaposin	MYALFLLA!	27	14	579	14	524	58.1	5.17	28343.4	44266.4	0.640292
Q59ED3	Intercellulɑ	LIVTCSLQQ	11	4	50	4	344	38.4	7.78	1243.9	1945.1	0.639504
S6BAR0	IgG L chain	MAWTPLFI	31	6	1319	2	216	23.1	7.69	1268.6	1988.2	0.638065
B2RDY9	Adenylyl cy	MADMQNI	4	2	2	2	475	51.6	8.22	174.4	273.4	0.637893
A0A0F7RQ	Follicle stir	MKTLQFFF	6	1	6	1	129	14.7	7.06	193.2	303.2	0.637203
D3DPK5	SH3 domai	KQLKPSKAI	14	5	147	5	257	26.8	8.38	5353	8402.9	0.637042
P00734	Prothromb	MAHVRGL(	33	17	694	17	622	70	5.9	25009.6	39265.9	0.636929
P21266	Glutathion	MSCESSM\	13	3	11	3	225	26.5	5.54	39.2	61.6	0.636364
P02671	Fibrinogen	MFSMRIVC	17	12	100	12	866	94.9	6.01	2688.6	4234.1	0.634987
P01591	Immunoglc	MKNHLLFV	42	6	301	6	159	18.1	5.24	13951.6	22042.9	0.632929
P15104	Glutamine	MTTSASSH	2	1	1	1	373	42	6.89	16.7	26.4	0.632576
P26842	CD27 antig	MARPHPW	22	4	102	4	260	29.1	7.64	5361	8475.3	0.632544
O43493	Trans-Golg	MRFVVALV	2	1	1	1	480	51.1	5.73	104.1	164.8	0.631675
O43567	E3 ubiquiti	MLLSIGML	4	2	6	2	381	42.8	4.98	425.8	675	0.630815
A0A024R9(	Thrombos|	MGLAWGL	11	10	67	1	1170	129.3	4.94	1885.2	2992.3	0.630017
Q6PK18	2-oxogluta	MAPQRRA/	10	3	4	3	319	35.6	8.18	10.6	16.9	0.627219
A0A024R0/	Macrophag	MTAPGAA(	21	10	138	6	554	60.1	5.29	4574	7324.9	0.624445
Q03164	Histone-lys	MAHSCRW	0	1	10	1	3969	431.5	9.09	378.3	606.1	0.624154
Q6UXG3	CMRF35-lik	MRLLVLLW	13	4	114	4	332	36	5.92	7962.6	12761.2	0.62397
A2NJV5	Kappa light	MRLPAQLL	20	2	74	1	121	13.2	7.28	79.8	127.9	0.623925
Q2UY09	Collagen al	MWNRYFV	2	2	30	2	1125	116.6	6.4	988.9	1588.2	0.622655
Q6UY14	ADAMTS-li	MENWTGR	2	2	3	2	1074	116.5	8.34	159.1	255.7	0.622214
B7Z5W1	cDNA FLJ5⁴	MLQAPRGI	15	5	29	5	303	32.8	8.85	1145.9	1842.3	0.621994

A8K556	cDNA FLJ78	MATTVPDC	6	2	4	2	357	40.3	8.15	41.1	66.2	0.620846
P02743	Serum amy	MNKPLLWI	16	3	18	3	223	25.4	6.54	697.9	1124.2	0.620797
A0A0B4J1S	High affinit	MDGEATVI	1	1	3	1	577	62.1	9.47	236	380.5	0.620237
A2NW98	Rheumatoi	MVLQTQVI	7	1	5	1	134	14.8	7.12	115.5	186.7	0.61864
Q6PIL8	IGK@ prot	METPAQLL	47	8	2890	1	236	25.8	6.55	2074.2	3364.6	0.616477
P03973	Antileukop	MKSSGLFP	9	1	5	1	132	14.3	8.75	265.8	431.3	0.616276
Q59EG0	Basement	SVTEGQTLI	15	27	593	1	2331	246.3	7.23	1070.7	1737.4	0.616266
D6W551	Chromosor	MKTSAELH	2	1	1	1	284	30.3	8.38	45.2	73.6	0.61413
Q03591	Compleme	MWLLVSVI	20	6	36	2	330	37.6	7.39	1029.9	1680.8	0.612744
P36896	Activin rec	MAESAGAS	6	2	37	2	505	56.8	7.03	1232.7	2014	0.612066
Q9NP84	Tumor nec	MARGSLRF	8	1	9	1	129	13.9	8.95	539.2	882.1	0.611269
A0A075B6I	Protein IGK	MEAPAQLL	23	2	146	1	116	12.8	5.25	299.4	489.9	0.611145
A0A109PS5	GCT-A8 ligh	DIVMTQSP	33	2	93	1	112	12.5	8.5	130.5	213.7	0.610669
D6W5K2	Thymosin,	GVSSRFPEF	8	2	7	2	169	17.9	10.48	536.4	878.5	0.610586
A0A024RC	Cadherin 2	MCRIAGAL	12	7	145	7	906	99.7	4.81	2964.5	4857.4	0.610306
Q96SA9	Anti-strept	DIQMTQSF	49	3	130	1	107	11.5	8.85	34.4	56.4	0.609929
Q9ULV1	Frizzled-4 (	MAWRGAC	4	2	39	2	537	59.8	7.27	1398.8	2293.8	0.609818
Q86UZ8	FZD2 prote	ARGVSGCS	1	1	8	1	641	71.4	9	371.6	609.8	0.60938
O43405	Cochlin OS	MSAAWIPA	2	1	6	1	550	59.4	7.96	275.7	452.5	0.609282
B2R597	cDNA, FLJ9	MNVLLGSV	14	2	78	2	114	12.8	5.5	7418.6	12181.9	0.608985
P25815	Protein S1(	MTELETAN	24	2	14	2	95	10.4	4.88	548.2	900.9	0.608503
B2R960	cDNA, FLJ9	MVGVKPV(	2	1	3	1	289	32.2	4.96	112.4	184.8	0.608225
Q8NHP8	Putative ph	MVGQMYC	11	6	35	6	589	65.4	6.8	1367.5	2249.8	0.607832
Q0KKI6	Immunobl(	EIVMTQSP	48	8	2901	1	219	24	8.06	2855.3	4700.9	0.607394
P01258	Calcitonin (	MGFQKFSF	13	2	2	2	141	15.5	6.52	244.8	403.3	0.606992
P11279	Lysosome-	MAAPGSAI	8	4	74	4	417	44.9	8.75	3348.7	5517	0.606978
A0A024R5	EH domain	MEQPGTA	5	3	9	2	548	61.9	6.71	140	231	0.606061
O75071	EF-hand ca	MKKRKELN	4	2	8	2	495	55	6.32	204.3	337.1	0.606052
B2RBC8	cDNA, FLJ9	MEKRLGVK	2	2	13	2	1049	115.5	6.11	530.6	875.9	0.605777
A0AVF1	Intraflagell	MMLSRAKI	1	1	17	1	554	64.1	6.93	738.7	1223.2	0.603908
Q14767	Latent-trar	MRPRTKAF	4	6	32	6	1821	194.9	5.19	1639.6	2717.6	0.603326
X6R8F3	Neutrophil	MPLGLLWI	44	6	125	6	200	22.8	8.5	3983.1	6613.9	0.602232
E7ETN3	Uncharacte	MGPLMVLI	17	15	92	1	1115	123.9	6.8	34.2	56.9	0.601054

P14780	Matrix met	MSLWQPL\	15	9	25	9	707	78.4	6.06	1080.4	1798	0.60089
Q16769	Glutaminyl	MAGGRHR	12	4	106	4	361	40.9	6.61	3676	6127	0.599967
Q8N3J6	Cell adhesi	MIWKRSA\	8	2	5	2	435	47.5	5.33	135.6	226.2	0.599469
C9JF17	Apolipoprc	MFKQLSCIS	33	8	1310	8	215	24.1	5.6	75109.3	125321.7	0.599332
P02461	Collagen al	MMSFVQK	2	3	14	3	1466	138.5	6.61	890.7	1488.3	0.598468
Q9BYJ0	Fibroblast ;	MKFVPCLL	5	1	1	1	223	24.6	8.87	38.3	64.1	0.597504
O43291	Kunitz-type	MAQLCGLF	10	2	15	2	252	28.2	8.29	558.5	935.7	0.596879
E9PR17	CD59 glyco	MGIQGGS\	37	5	1278	5	130	14.5	7.77	66869	112175.1	0.596113
Q14247	Src substra	MWKASAG	5	2	35	2	550	61.5	5.4	675.4	1133.4	0.595906
Q13201	Multimerir	MKGARLFV	2	2	33	2	1228	138	7.93	1835.3	3084.4	0.595027
J3KPA1	Cysteine-ri	MKQILHPA	13	2	12	2	276	31	7.61	686.2	1153.4	0.594937
Q9Y6R7	IgGFc-bind	MGALWSW	11	24	90	24	5405	571.6	5.34	2487.8	4182.5	0.594812
P01833	Polymeric i	MLLFVLTCL	37	24	1000	24	764	83.2	5.74	44831.7	75401.1	0.594576
P25311	Zinc-alpha-	MVRMVPV	60	20	1734	20	298	34.2	6.05	90208.1	151729.7	0.594532
P36639	7,8-dihydrc	MYWSNQI	7	1	20	1	197	22.5	5.27	27.4	46.1	0.59436
Q6FGQ5	ASGR1 pro	MTKEYQDL	4	1	1	1	291	33.2	5.5	158.4	266.7	0.593926
Q68CJ9	Cyclic AMP	MNTDLAA(	7	2	39	2	461	49	5.15	1177.1	1983.8	0.593356
Q9NPF0	CD320 anti	MSGGWM.	14	4	49	4	282	29	4.75	1843.2	3112.6	0.592174
H3BQD0	Cerebellin-	MLGVLELLI	11	1	8	1	140	14.7	5.48	197	333	0.591592
Q9ULC0	Endomucir	MELLQVTIL	5	1	5	1	261	27.4	7.94	456.2	772.7	0.590397
A0A024R8(	Prostaglan	MATHHTLV	41	6	3425	6	190	21	7.8	92848.1	157265.4	0.590391
B2R888	Monocyte	MERASCLL	35	12	411	12	375	40	6.23	13265.4	22517.1	0.589126
P17900	Gangliosid	MQSLMQA	26	6	366	6	193	20.8	5.31	17844.8	30307.1	0.588799
P12273	Prolactin-ir	MRLLQLLFI	36	5	115	5	146	16.6	8.05	3471.5	5909.5	0.587444
B5TMG5	Histidine ri	MGHHRPW	1	1	5	1	750	86.2	4.75	373.4	636.8	0.586369
Q9UGN4	CMRF35-lik	MWLPWAL	10	2	82	2	299	33.2	5.49	3620.8	6215.1	0.582581
P15428	15-hydroxy	MHVNGKV	3	1	1	1	266	29	5.86	13.2	22.7	0.581498
Q8N4F0	BPI fold-co	MAWASRL(	7	3	15	3	458	49.1	8.72	566.6	975	0.581128
Q9H477	Ribokinase	MAASGEP(	2	1	2	1	322	34.1	5.05	13	22.4	0.580357
A0A024RA	Endothelin	MRGVWPF	2	1	1	1	770	87.1	5.88	120.2	207.5	0.579277
B7Z9B1	cDNA FLJ5;	MKTPPGAS	20	12	469	12	760	83.4	5.12	16251.4	28059.6	0.579174
P51170	Amiloride-:	MAPGEKIK	4	2	8	2	649	74.2	7.5	256.5	446.9	0.573954
Q6MZK8	Putative ur	MSDLSVIGI	1	2	10	1	2060	226.3	4.44	602.8	1055.5	0.571104

Q6UX06	Olfactome	MRPGLSFL	21	10	125	10	510	57.2	5.69	3660.8	6418.7	0.570334
B7Z6Z4	Myosin ligh	MVGPRSW	3	1	1	1	238	26.7	5.08	37.9	66.5	0.569925
Q9NZU0	Leucine-ric	MISAAWSI	6	3	4	3	649	73	7.64	13.4	23.7	0.565401
Q9NQ38	Serine prot	MKIATVSVI	6	4	20	4	1064	120.6	8.06	1196.8	2121.7	0.564076
Q99835	Smoothene	MAAARPAI	1	1	4	1	787	86.3	8.34	203.6	361.7	0.562897
A0A096LN	Uncharacte	MALTKMLI	6	1	6	1	201	23.3	9.38	152.7	272.3	0.560779
Q9GZX9	Twisted ga	MKLHYVAV	24	4	43	4	223	25	5.34	1627.6	2924.7	0.556502
Q96DA0	Zymogen g	MGAQGAC	30	5	102	5	208	22.7	7.39	3676.1	6621.2	0.555201
Q96FE7	Phosphoin	MLLAWVQ	21	5	638	5	263	28.2	5.01	33426.8	60639.2	0.551241
S4R471	Protein AM	HKSKWNIT	51	10	2476	1	193	21.4	7.25	501.8	913.4	0.549376
P98172	Ephrin-B1 (	MARPGQR	16	6	65	6	346	38	8.94	2013.2	3677.8	0.547392
A8K6C9	cDNA FLJ7	MGIPMGK	9	2	8	2	180	20.2	9.32	149	272.2	0.547392
P02750	Leucine-ric	MSSWSRQ	28	9	294	9	347	38.2	6.95	10453.9	19100	0.547325
Q5T1S8	Noncompa	MTTATPLG	7	1	16	1	102	11.1	9.86	1528.2	2795.3	0.546703
Q5KU26	Collectin-1	MKDDFAEE	7	6	82	6	742	81.5	5.69	3325.5	6136.8	0.541895
Q8N114	Protein shi	MTAPVPAF	15	4	139	4	240	25.6	6.68	5190.8	9621.4	0.539506
Q8N355	IGL@ prote	MAWTVLLI	43	8	1436	1	234	24.8	6.37	104	194.2	0.53553
Q13508	Ecto-ADP-r	MKTGHFEI	5	2	58	2	389	43.9	6.06	2121.5	3963.6	0.535246
P16070	CD44 antig	MDKFWWI	9	6	582	6	742	81.5	5.33	32947.2	61721.5	0.533804
P02760	Protein AM	MRSLGALL	51	16	4825	5	352	39	6.25	203377.3	381627.7	0.532921
A0A024R2	Tyrosine-p	MSSWIRW	2	1	2	1	838	93.8	6.57	13.5	25.5	0.529412
P13473	Lysosome-	MVCFRLFP	7	3	113	3	410	44.9	5.63	4694	8958.6	0.523966
P07998	Ribonuclea	MALEKSLV	58	6	309	6	156	17.6	8.79	13152.6	25347.2	0.518898
H7BY55	Compleme	MTVARPSV	24	12	331	12	550	58.9	8.78	11991	23131.9	0.518375
Q8NG20	Plasminoge	MSEGNSD(	14	2	6	2	90	9.8	7.14	93.3	180.8	0.51604
Q05D71	TNFRSF10(	MARIPKTLI	3	1	2	1	277	29.7	8.4	316.9	615.2	0.515117
Q5IWS5	Intelectin 1	MNQLSFLL	16	4	13	4	313	35	5.82	407.9	793.9	0.513793
Q13421	Mesothelir	MALPTARP	11	6	47	1	630	68.9	6.38	751	1462.1	0.513645
B4DM05	cDNA FLJ5	MLASSSRIF	17	12	155	2	726	79.3	4.97	1987.1	3873.7	0.512972
Q9BZG9	Ly-6/neuro	MTPLLTLIL	18	2	152	2	131	14	6.71	4311.3	8447.9	0.51034
O43653	Prostate st	MKAVLLAL	22	2	164	2	123	12.9	5.29	7470.4	14644.6	0.510113
P78492	Inter-alpha	LWAFDAVK	75	3	1658	1	51	5.7	4.89	417.2	820	0.50878
A0A024R6	Delta-like 1	MTATEALL	11	3	41	3	383	41.2	5.53	3555.6	7066.9	0.503134

B7Z7X3	cDNA FLJ5:	MEERGGV'	2	1	6	1	921	102.5	8.29	269	538.2	0.499814
Q96JF0	Beta-galact	MKPHLKQ\	1	1	1	1	529	60.1	9.74	73.2	148	0.494595
A0A024RD	Secreted p	MRIAVICF(	47	12	1140	1	300	33.8	4.61	4818.4	9894.7	0.486968
P02749	Beta-2-glyc	MISPVLILF!	45	11	404	11	345	38.3	7.97	12893.6	26758.6	0.481849
Q96ID5	Immunogl(	MRTAPSLR	2	1	1	1	467	51.8	6.93	17.1	35.5	0.48169
Q9Y279	V-set and i	MGILLGLLL	13	3	32	3	399	44	6.35	914.9	1900.6	0.481374
G3V3Y2	Fibulin-5 (F	MPGIKRILT	47	3	80	1	91	9.9	6.37	121.8	255.6	0.476526
X6RBG4	Uromoduli	MRHIMTRL	39	23	3450	23	689	75.6	5.87	173051.8	364524.4	0.474733
A0A024RD	Secreted p	MRIAVICF(	50	13	1136	2	314	35.4	4.58	33620	70922.1	0.474041
Q13790	Apolipoprc	MTGLCGYS	4	1	6	1	326	35.4	5.64	111	235.1	0.47214
P61604	10 kDa hea	MAGQAFR	30	3	3	3	102	10.9	8.92	45.4	96.8	0.469008
B2R7Z2	cDNA, FLJ9	MPRQLSA/	5	2	3	2	416	44.3	8.41	131	286.3	0.457562
P19957	Elafin OS=I	MRASSFLIV	14	1	4	1	117	12.3	8.82	133.1	296.5	0.448904
Q96TA2	ATP-depen	MFSLSSTV(	1	1	1	1	773	86.4	8.76	72.9	165.7	0.439952
P48551	Interferon	MLLSQNAF	3	2	13	2	515	57.7	4.5	532.1	1228.8	0.433024
P55000	Secreted L	MASRWAV	61	3	21	3	103	11.2	5.33	283.5	663.4	0.427344
A0A0S2Z4I	Secretoglo	MKLAVTLT	20	3	114	3	91	10	5.06	5773	13735	0.420313
Q9Y5Y7	Lymphatic	MARCFSLV	18	6	249	6	322	35.2	8.28	9204.5	22274.4	0.413232
P31151	Protein S1(	MSNTQAEI	34	3	35	3	101	11.5	6.77	659.2	1722.6	0.382677
J3KR56	Receptor a	METGALRF	4	1	4	1	151	16.8	5.48	193.7	510.1	0.379729
Q96AX9	E3 ubiquiti	MGWKPSE	1	1	4	1	1013	109.9	8.44	30.2	88.1	0.342792

Uniprot Accession Number	protein	Protein amino acid sequence	Protein amino acid sequence matching degree(%)	The number of peptides identified	The total number of times a polypeptide was identified	The number of characteristic peptides identified	Amino acid length	KDa	isoelectric point	relative abundance of normalized protein - sample 116	relative abundance of normalized protein - control sample 121	ratio(116/1
P04279	Semenogel	MKPNIIFVL	53	23	236	17	462	52.1	9.29	60356.9	12317.9	4.899934
A8K6Z6	cDNA FLJ7	MKPNIIFVL	43	21	144	15	582	65.6	9.07	13370.1	2968.1	4.504599
S6BAP4	IgG H chain	MDWTWR	22	4	123	1	275	29.4	8.51	53.7	14.6	3.678082
P12273	Prolactin-in	MRLLQLLFI	36	5	115	5	146	16.6	8.05	12194.5	5909.5	2.063542
P52209	6-phospho	MAQADIAL	7	3	7	3	483	53.1	7.23	75.7	38.5	1.966234
B4E1C2	Kininogen	MKLITILFL	36	27	3259	1	644	71.9	6.81	326	167.9	1.941632
P20142	Gastricsin	MKWMVV	2	1	2	1	388	42.4	4.46	150.6	78.6	1.916031
Q9Y546	Leucine-ric	MSYYLSSE	3	1	6	1	428	48.5	7.46	745.5	408	1.827206
S6AWD3	IgG L chain	MRLPAQLL	23	4	1483	1	183	19.9	8.91	1491.4	830	1.796867
Q5HYA8	Meckelin C	MATRGGA	2	1	3	1	995	111.7	6.77	38.3	21.4	1.78972
Q68DC2	Ankyrin re	MGEGGLP	1	1	8	1	871	92.2	7.39	1120.5	647	1.731839
Q0ZCJ2	Immunglob	LVQLVESG	46	5	89	1	124	13.6	7.93	41.6	24.3	1.711934
O94967	WD repeat	MTAEETVN	1	1	3	1	919	101.9	5.94	191.3	112.6	1.698934
A0A024R8	Ubiquitinyl	MARMGDS	1	1	2	1	916	102.2	6.21	69.7	41.4	1.683575
P15104	Glutamine	MTTSASSH	2	1	1	1	373	42	6.89	44.4	26.4	1.681818
Q9UL81	Myosin-rea	DIQMTQSP	51	3	104	2	107	11.5	8.48	38.9	23.2	1.676724
P46781	40S riboso	MPVARSW	5	1	1	1	194	22.6	10.65	155.4	93.2	1.667382
P13521	Secretogra	MAEAKTH	2	1	2	1	617	70.9	4.75	217.4	132.3	1.643235
B4DMS4	cDNA FLJ5	MGKLSRCS	2	2	4	2	812	88.4	4.4	590.7	369.1	1.600379
W8QEY1	Lactoferrin	MKLVFLVL	52	33	446	1	711	78.3	8.18	323.9	203	1.595567
P01766	Immunogl	MELGLSW	19	2	19	1	116	12.5	7.08	178.7	115.1	1.552563
A0A075B6	Ig lambda-	XQPKAAPS	48	4	1262	1	106	11.3	8.29	195.9	126.3	1.551069
Q6UWP8	Suprabasin	MHLARLV	2	1	1	1	590	60.5	7.01	493.5	321.2	1.536426
Q8N0V5	N-acetyllac	MMGSWKI	3	1	1	1	402	45.8	7.18	23.8	15.5	1.535484
C0JYY2	Apolipopro	MDPPRPAI	1	3	5	3	4563	515.2	7.05	61.9	40.5	1.528395

Q86YD5	Low-densit	MWLLGPL(	5	1	1	1	345	37.4	4.87	16.5	10.8	1.527778
P01023	Alpha-2-ma	MGKNKLLH	14	17	49	17	1474	163.2	6.46	2120.8	1389.4	1.526414
P04083	Annexin A1	MAMVSEFI	51	16	132	16	346	38.7	7.02	5595.3	3672	1.523775
P29508	Serpin B3 (	MNSLSEAN	23	8	23	8	390	44.5	6.81	790.4	519.3	1.522049
A0A161I20	Lactoferrin	MKLVFLVLI	54	34	466	0	711	78.3	8.18	22989.8	15145.7	1.517909
P12429	Annexin A3	MASIWVGI	22	6	14	6	323	36.4	5.92	183.5	120.9	1.517783
B2R6Q2	Alkaline ph	MISPFLVLA	2	1	2	1	524	57.2	6.57	18.2	12	1.516667
Q6IB39	RNASE6 pr	MVLCFPLLI	19	2	12	2	150	17.2	8.76	465.7	310.4	1.500322
P55000	Secreted Ly	MASRWAV	61	3	21	3	103	11.2	5.33	441.1	663.4	0.664908
Q68CJ9	Cyclic AMP	MNTDLAA(	7	2	39	2	461	49	5.15	1312.2	1983.8	0.661458
Q8NG20	Plasminoge	MSEGNSD(	14	2	6	2	90	9.8	7.14	119.2	180.8	0.659292
P13645	Keratin, typ	MSVRYSSSI	31	18	186	13	584	58.8	5.21	7392.4	11461.9	0.644954
H6VRG1	Keratin 1 C	MSRQFSSR	34	20	161	16	645	66.1	8.12	5944.3	9226.5	0.644264
Q5IWS5	Intelectin 1	MNQLSFLL	16	4	13	4	313	35	5.82	509.9	793.9	0.642272
P02743	Serum amy	MNKPLLWI	16	3	18	3	223	25.4	6.54	704.4	1124.2	0.626579
A0A1B0GU	Uncharacte	MYGDIFNA	13	1	1	1	103	10.9	8.92	17.1	27.8	0.615108
Q9H9P2	Chondrole	MSRVVSLLI	11	2	2	2	273	30.4	6.83	9.1	15.2	0.598684
Q15485	Ficolin-2 O	MELDRAV(	16	6	22	6	313	34	6.77	1262.5	2120.4	0.595407
B2RDY9	Adenylyl cy	MADMQNI	4	2	2	2	475	51.6	8.22	161.1	273.4	0.589247
A0A0S2Z4F	Secretoglo	MKLAVTLT	20	3	114	3	91	10	5.06	7533.6	13735	0.548497
Q8N4F0	BPI fold-co	MAWASRL	7	3	15	3	458	49.1	8.72	533.5	975	0.547179
Q13790	Apolipopro	MTGLCGYS	4	1	6	1	326	35.4	5.64	115	235.1	0.489154

Uniprot Accession Number	protein	Protein amino acid sequence	Protein amino acid sequence matching degree(%)	The number of peptides identified	The total number of times a polypeptide was identified	The number of characteristic peptides identified	Amino acid length	KDa	isoelectric point	relative abundance of normalized protein - sample 117	relative abundance of normalized protein - control sample 121	ratio(117/1
Q4TZM4	Hemoglobi	TPEEKSAVT	89	8	135	2	101	11	6.52	391.1	134.5	2.907807
D9YZU5	Beta-globir	MVHLTPEE	78	10	328	1	147	16	7.28	20029.3	8175.3	2.449977
A0A0S2Z4F	Secretoglo	MKLAVTLT	20	3	114	3	91	10	5.06	32076.5	13735	2.335384
A0A1K0GX	Globin C1 (	MSAARPG\	50	6	182	6	177	19.2	9.77	15153	6799.3	2.228612
A0A024R8/	Ubiquitinyl	MARMGDS	1	1	2	1	916	102.2	6.21	85.9	41.4	2.074879
Q7L9L4	MOB kinas	MSFLFGSR:	5	1	1	1	216	25.1	6.73	18.8	10.2	1.843137
Q8WWQ8	Stabilin-2 (	MMLQHLV	0	1	1	1	2551	276.8	6.4	21.3	11.6	1.836207
P49788	Retinoic ac	MQPRRQR	20	5	28	5	294	33.3	8.51	1750.1	965.3	1.813011
Q59EU6	EF hand do	CEERAARQ	4	1	1	1	265	29.8	6.21	18.2	10.2	1.784314
A0A140VK:	Leukotrien	MPEIVDTC:	2	1	1	1	611	69.2	6.18	50	28.3	1.766784
Q9NX62	Inositol mc	MAPMGIRI	3	1	2	1	359	38.7	6.86	23	13.2	1.742424
Q53FP0	Pyridoxine	MTCWLRG	6	1	1	1	261	29.9	7.4	41.4	24.6	1.682927
B2R9K8	cDNA, FLJ9	MAAVKTLN	2	1	1	1	531	57.9	6.8	29	17.3	1.676301
Q8N0V5	N-acetyllac	MMGSWKI	3	1	1	1	402	45.8	7.18	25.7	15.5	1.658065
B4DMS4	cDNA FLJ5	MGKLSRCS	2	2	4	2	812	88.4	4.4	608.2	369.1	1.647792
P04118	Colipase O:	MEKILILLL\	7	1	9	1	112	11.9	7.5	1277.9	777.9	1.642756
X6R8F3	Neutrophil	MPLGLLWI	44	6	125	6	200	22.8	8.5	10723.4	6613.9	1.621343
Q9UBV8	Peflin OS=I	MASYPYRC	3	1	1	1	284	30.4	6.54	160	99.1	1.614531
P15291	Beta-1,4-ga	MRLREPLL:	5	2	18	2	398	43.9	8.65	951	589.3	1.613779
Q68DC2	Ankyrin rep	MGEGGLPI	1	1	8	1	871	92.2	7.39	1042.4	647	1.611128
A8K987	cDNA FLJ7:	MAEKPKLH	18	5	71	1	222	25.7	9	5215.5	3254.9	1.602353
Q14117	Dihydropyı	MAAPSRLL	3	2	6	2	519	56.6	7.27	209.1	130.9	1.597403
A0A075B6	Protein IGI	MDMRVPA	14	1	2	1	117	12.7	9.25	32	20.1	1.59204
D3DSM8	Formimino	MRSRWGG	2	1	1	1	581	63.3	6.3	42	26.4	1.590909
B4E1C2	Kininogen :	MKLITILFLC	36	27	3259	1	644	71.9	6.81	265.5	167.9	1.581298

P12724	Eosinophil	MVPKLFTS(	17	2	5	2	160	18.4	10.02	122.3	77.9	1.569961
O95861	3'(2'),5'-bis	MASSNTVL	3	1	1	1	308	33.4	5.69	33.3	21.3	1.56338
A0A140VK(	Aspartate a	MAPPSVFA	14	5	9	5	413	46.2	7.01	626.6	402.1	1.558319
P07195	L-lactate d(	MATLKEKL	30	9	126	8	334	36.6	6.05	11867.5	7645.5	1.55222
P52209	6-phospho	MAQADIAL	7	3	7	3	483	53.1	7.23	59.6	38.5	1.548052
B2RD36	cDNA, FLJ9	MANEAYP(	15	4	17	4	245	28	6.79	1323.4	855.7	1.54657
B1B5Q3	N-acylsphir	YRGAVPW'	28	7	64	1	219	24.7	7.42	1705.7	1108.7	1.538468
A8K0T9	cDNA FLJ7!	MADFDDR'	17	3	6	2	286	32.9	5.69	362.7	238.9	1.518208
B4E0X1	Beta-2-mic	MSRSVALA	27	4	110	4	122	13.9	7.44	12376.4	8163.5	1.516065
A0A075B6I	Ig lambda-	XQPKAAPS	48	4	1262	1	106	11.3	8.29	191.2	126.3	1.513856
B2RAF9	Suppressor	MGSDRARI	2	1	1	1	855	94.7	6.52	50.1	33.1	1.513595
P04899	Guanine nu	MGCTVSAE	10	3	20	2	355	40.4	5.54	221.7	147.3	1.505092
K7N7A8	Uncharacte	XETLTLHSL	4	1	3	1	449	48.8	5.22	18.9	12.6	1.5
Q8N355	IGL@ prote	MAWTVLLI	43	8	1436	1	234	24.8	6.37	126.6	194.2	0.651905
P02763	Alpha-1-ac	MALSWVL'	40	7	965	5	201	23.5	5.02	30650.9	48390.7	0.633405
A0A1B0GU	Uncharacte	MYGDIFNA	13	1	1	1	103	10.9	8.92	16.8	27.8	0.604317
Q96AX9	E3 ubiquiti	MGWKPSE	1	1	4	1	1013	109.9	8.44	52.6	88.1	0.597049
B2RDY9	Adenylyl cy	MADMQNI	4	2	2	2	475	51.6	8.22	153.9	273.4	0.562911
Q5IWS5	Intelectin 1	MNQLSFLL	16	4	13	4	313	35	5.82	416.2	793.9	0.524247

Uniprot Accession Number	protein	Protein amino acid sequence	Protein amino acid sequence matching degree(%)	The number of peptides identified	The total number of times a polypeptide was identified	The number of characteristic peptides identified	Amino acid length	KDa	isoelectric point	relative abundance of normalized protein - sample 118	relative abundance of normalized protein - control sample 121	ratio(118/1
Q4TZM4	Hemoglobi	TPEEKSAVT	89	8	135	2	101	11	6.52	392.4	134.5	2.917472
V9HVY1	Epididymis	MKRMVSW	25	11	29	11	491	55.9	8.27	2788.9	1138.4	2.449842
A0A1K0FU	Globin E1 (	MLSAQERA	11	1	1	1	141	15.6	6.62	19	7.8	2.435897
C0JYY2	Apolipopro	MDPPRPAL	1	3	5	3	4563	515.2	7.05	95.9	40.5	2.367901
K7ES00	Histone H3	MARTKQTA	9	2	8	2	151	16.6	11.84	1406.8	602.6	2.33455
A0A024R3I	Apolipopro	MKAAVLTL	60	16	172	16	267	30.8	5.76	13017.8	5583.4	2.331518
P59665	Neutrophil	MRTLAILAA	19	2	29	2	94	10.2	6.99	4014.5	1746.8	2.298202
P08246	Neutrophil	MTLGRRLA	6	2	11	2	267	28.5	9.35	783.7	343.6	2.28085
D9YZU5	Beta-globir	MVHLTPEE	78	10	328	1	147	16	7.28	18593.9	8175.3	2.2744
B2R9K8	cDNA, FLJ9	MAAVKTLN	2	1	1	1	531	57.9	6.8	38.9	17.3	2.248555
A0A0U1RR	Histone H2	MSGRGKQ	14	3	13	3	170	18.5	11.52	1312.5	601.7	2.18132
B2R4R0	Histone H4	MSGRGKG	50	5	58	5	103	11.4	11.36	3202.8	1469.8	2.179072
P24158	Myeloblast	MAHRPPSF	17	3	17	3	256	27.8	8.35	623	289.9	2.149017
P19013	Keratin, typ	MIARQQCV	20	10	34	7	534	57.3	6.61	1621.4	765.5	2.118093
P05164	Myelopero	MGVPFFSS	20	13	48	13	745	83.8	8.97	2952.7	1404.2	2.102763
P11234	Ras-related	MAANKSK(	7	1	2	1	206	23.4	6.62	39.8	19.1	2.08377
P13647	Keratin, typ	MSRQSSVS	18	11	34	4	590	62.3	7.74	22.9	11	2.081818
C9JC84	Fibrinogen	MSWSLHPI	28	9	37	9	461	52.3	5.63	1831.3	884.6	2.070201
A0A1K0GX	Globin C1 (	MSAARPGV	50	6	182	6	177	19.2	9.77	13904.8	6799.3	2.045034
A0A0S2Z3V	EGF contai	MLKALFLTI	46	14	386	1	413	45.7	5.47	239.5	118.6	2.019393
V9GYM3	Apolipopro	MCEQHPKI	14	2	10	2	133	14.9	8.27	635.1	315.6	2.012357
Q6ICS1	Putative ur	MAQTNSFF	24	3	114	1	166	18.7	5.95	61.3	30.7	1.996743
A0A075B6S	Protein IGK	MDMRVPA	14	1	2	1	117	12.7	9.25	39.4	20.1	1.960199
Q59EG0	Basement	SVTEGQTLI	15	27	593	1	2331	246.3	7.23	3369.3	1737.4	1.939277
Q15043	Zinc transp	MKLLLLHPA	2	1	3	1	492	54.2	5.33	257.6	133	1.936842

B4DR52	Histone H2	MPDPAKSA	20	3	22	3	166	18	10.32	1902.3	992.5	1.916675
Q96IY4	Carboxype	MKLCSLAV	2	1	2	1	423	48.4	7.71	53.3	27.9	1.910394
A0A024R0	Apolipopro	MRLFLSLPV	11	1	4	1	83	9.3	8.47	95.9	52	1.844231
Q9NX62	Inositol mo	MAPMGIRI	3	1	2	1	359	38.7	6.86	24	13.2	1.818182
P08263	Glutathion	MAEKPKLH	18	5	78	1	222	25.6	8.88	586.6	331	1.772205
P01023	Alpha-2-ma	MGKNKLLH	14	17	49	17	1474	163.2	6.46	2447.4	1389.4	1.76148
B2RCC8	cDNA, FLJ9	MFLWLFLII	2	1	1	1	837	94.4	7.97	30.9	17.6	1.755682
D3DRR6	Inter-alpha	MKRLTCFFI	5	4	10	4	947	106.5	6.95	432.7	249.7	1.732879
B4E1C2	Kininogen	MKLITILFLC	36	27	3259	1	644	71.9	6.81	290.3	167.9	1.729005
P13521	Secretogra	MAEAKTHV	2	1	2	1	617	70.9	4.75	226.3	132.3	1.710506
P23468	Receptor-t	MVHVARLL	2	3	3	2	1912	214.6	6.57	82.3	48.4	1.700413
P20160	Azurocidin	MTRLTVLA	14	3	27	3	251	26.9	9.5	1039.5	611.6	1.69964
A1A4E9	Keratin 13	MSLRLQSS	25	14	107	9	458	49.6	4.96	5475.3	3225.2	1.697662
Q59EU6	EF hand do	CEERAARQ	4	1	1	1	265	29.8	6.21	17.3	10.2	1.696078
Q96SB0	Anti-strept	QSVLTQPP	22	2	13	1	108	11.6	6.54	159.3	94.1	1.69288
S6BAR0	IgG L chain	MAWTPLFI	31	6	1319	2	216	23.1	7.69	3364.4	1988.2	1.692184
P15291	Beta-1,4-ga	MRLREPLLS	5	2	18	2	398	43.9	8.65	997.2	589.3	1.692177
Q9UKU9	Angiopoiet	MRPLCVTC	23	11	129	11	493	57.1	7.53	10462.1	6197.8	1.688034
Q92743	Serine prot	MQIPRAAL	3	1	1	1	480	51.3	7.83	123.9	74.4	1.665323
D3DQU2	Tripeptidyl	MTADPRKC	20	8	82	8	572	62.2	6.65	5232.1	3143.2	1.664578
A0A0A0MT	Titin OS=H	(content is	0	4	5	4	35991	3992.2	6.39	256	154.4	1.658031
P52209	6-phospho	MAQADIAL	7	3	7	3	483	53.1	7.23	63.6	38.5	1.651948
Q9UL81	Myosin-rea	DIQMTQSP	51	3	104	2	107	11.5	8.48	38	23.2	1.637931
V9HWA9	Epididymis	MGPTSGPS	35	55	305	55	1663	187	6.4	17333.2	10679.9	1.622974
A0A075B6I	Immunoglo	MSVPTMA	15	2	29	2	122	12.8	4.55	3276.7	2023.3	1.619483
B4DMS4	cDNA FLJ59	MGKLSRCS	2	2	4	2	812	88.4	4.4	594.4	369.1	1.610404
P20333	Tumor nec	MAPVAVW	6	2	7	1	461	48.3	6.28	1127.5	706.7	1.595444
A0A024RA	Arylsulfata	MGPRGAA	3	2	5	2	533	59.6	8.21	377.4	236.9	1.593077
Q9Y2S2	Lambda-cry	MASSAAGC	13	4	25	4	319	35.4	6.18	1034.1	653.3	1.582887
B3KQP7	cDNA FLJ90	MMTKVLG	2	1	1	1	450	49.7	6.61	166.6	105.3	1.582146
P48547	Potassium	MGQGDES	3	1	2	1	511	57.9	6.58	30	19	1.578947
O94991	SLIT and NT	MHTCCPPV	1	1	4	1	958	107.4	6.95	155.5	98.7	1.575481
Q6FHG5	Gamma-sy	MDVFKKGF	13	1	7	1	127	13.3	4.86	168.6	107.4	1.569832

A0A125U0	GCT-A1 he	QVQLQESG	25	3	32	1	127	13.7	8.78	164.3	105.3	1.560304
P00338	L-lactate d	MATLKDQL	27	8	29	7	332	36.7	8.27	993.3	637.4	1.558362
P13640	Metallothi	MDPNCSCA	32	2	4	1	62	6.1	7.96	866.3	559.1	1.549454
Q53Y06	ATPase, H+	MALSDADV	4	1	4	1	226	26.1	8	224.7	145.2	1.547521
A8K0T9	cDNA FLJ7	MADFDDR	17	3	6	2	286	32.9	5.69	369.4	238.9	1.546254
A0A161I20	Lactoferrin	MKLVFLVLI	54	34	466	0	711	78.3	8.18	23345.6	15145.7	1.541401
Q8N474	Secreted fr	MGIGRSEG	9	2	4	2	314	35.4	8.85	97.4	63.3	1.538705
X6R8A1	Carboxype	MTSSPRAP	20	9	131	9	498	56.2	6.61	8585.6	5613.9	1.529347
D6W5K2	Thymosin,	GVSSRFPEF	8	2	7	2	169	17.9	10.48	1341.3	878.5	1.526807
Q03164	Histone-lys	MAHSCRW	0	1	10	1	3969	431.5	9.09	922.7	606.1	1.522356
Q96C23	Aldose 1-e	MASVTRA	11	3	9	3	342	37.7	6.65	51.1	33.6	1.520833
B1B5Q3	N-acylsphi	YRGAVPW	28	7	64	1	219	24.7	7.42	1682.7	1108.7	1.517723
P04155	Trefoil fact	MATMENK	30	2	29	2	84	9.1	4.35	3474.8	2289.6	1.517645
P0DJD8	Pepsin A-3	MKWLLLLG	9	4	250	4	388	42	4.41	34342.5	22700.4	1.512859
Q9HB40	Retinoid-in	MELALRRS	6	3	41	3	452	50.8	5.81	1368.5	904.6	1.512823
A0A140VK	Transaldol	MSSSPVKR	6	2	5	2	337	37.5	6.81	318.9	211	1.511374
Q59ER5	WD repeat	RAGPGGSR	2	1	2	1	624	68.1	7.23	90.3	59.9	1.507513
Q7L9L4	MOB kinas	MSFLFGSR	5	1	1	1	216	25.1	6.73	15.3	10.2	1.5
Q8WWQ8	Stabilin-2 (	MMLQHLV	0	1	1	1	2551	276.8	6.4	17.4	11.6	1.5
J3KPA1	Cysteine-ri	MKQILHPA	13	2	12	2	276	31	7.61	768.3	1153.4	0.666118
Q9UBX7	Kallikrein-1	MQRLRWL	14	4	43	4	282	31	8.94	1809.4	2722.4	0.664634
P51159	Ras-relatec	MSDGDYD	4	1	2	1	221	24.9	5.22	126.7	190.9	0.663698
A0A024R6	Delta-like 1	MTATEALL	11	3	41	3	383	41.2	5.53	4643.6	7066.9	0.657092
B2RDY9	Adenylyl cy	MADMQNI	4	2	2	2	475	51.6	8.22	175.6	273.4	0.642282
Q6UX06	Olfactome	MRPGLSFL	21	10	125	10	510	57.2	5.69	4072.6	6418.7	0.63449
O00337	Sodium/nu	MENDPSRF	3	1	26	1	649	71.5	7.59	125.3	198.9	0.629965
B8ZWD9	Diazepam I	MGWTSLC	31	3	46	3	144	16.1	5.05	1173.8	1884.1	0.623003
O43653	Prostate st	MKAVLLAL	22	2	164	2	123	12.9	5.29	9102	14644.6	0.621526
H3BQD0	Cerebellin-	MLGVLELLI	11	1	8	1	140	14.7	5.48	205.1	333	0.615916
Q9Y6R7	IgGFc-bind	MGALWSW	11	24	90	24	5405	571.6	5.34	2513.9	4182.5	0.601052
B7ZLW2	HELZ prote	MEDRRAEK	1	1	2	1	1943	219	7.42	14.5	24.3	0.596708
B3KQF4	cDNA FLJ9	MAPFEPLA	45	7	40	7	207	23.2	8.1	910.3	1549.3	0.587556
B2R880	cDNA, FLJ9	MANCEFSP	4	1	3	1	300	34.3	7.47	25.8	44.1	0.585034

B2R7D1	cDNA, FLJ9	MMDASKE	2	2	7	2	684	77.1	6.76	23	39.4	0.583756
Q5HYL0	Putative ur	MQRAVPE(	1	1	2	1	967	105.3	6.83	28.4	48.9	0.580777
P25311	Zinc-alpha-	MVRMVPV	60	20	1734	20	298	34.2	6.05	87034.2	151729.7	0.573613
Q96DR8	Mucin-like	MKFLAVLV	18	2	78	2	90	9	4.64	4241.8	7477.2	0.567298
Q8N4F0	BPI fold-co	MAWASRL	7	3	15	3	458	49.1	8.72	549	975	0.563077
Q6N092	Putative ur	GEEPSTRSF	34	14	1008	1	519	56.4	6.93	161.8	292.1	0.55392
B4DPH5	cDNA FLJ5!	MVLNDVYF	34	21	324	1	533	61.4	5.33	16.7	31.5	0.530159
M0R1F0	Prostate-sp	XFLTLSVTW	52	10	493	9	262	28.6	7.44	16031.4	34544.1	0.464085
A0A0S2Z4F	Secretoglo	MKLAVTLT	20	3	114	3	91	10	5.06	6110.8	13735	0.444907
O60662	Kelch-like p	MDSQRELA	1	1	2	1	606	68	5.29	83.2	188	0.442553
P15309	Prostatic a	MRAAPLLL	32	13	827	13	386	44.5	6.24	24586.7	60390	0.407132
B2R597	cDNA, FLJ9	MNVLLGSV	14	2	78	2	114	12.8	5.5	2737.9	12181.9	0.224751

Uniprot Accession Number	protein	Protein amino acid sequence	Protein amino acid sequence matching degree(%)	The number of peptides identified	The total number of times a polypeptide was identified	The number of characteristic peptides identified	Amino acid length	KDa	isoelectric point	relative abundance of normalized protein - sample 119	relative abundance of normalized protein - control sample 121	ratio(119/1
P00738	Haptoglobi	MSALGAVI	46	19	454	19	406	45.2	6.58	86108.9	22466.7	3.832735
K7ES00	Histone H3	MARTKQT/	9	2	8	2	151	16.6	11.84	1900.6	602.6	3.153999
Q4TZM4	Hemoglobi	TPEEKSAVT	89	8	135	2	101	11	6.52	398.7	134.5	2.964312
B4DR52	Histone H2	MPDPAKS/	20	3	22	3	166	18	10.32	2601.2	992.5	2.620856
B2R4R0	Histone H4	MSGRGKG	50	5	58	5	103	11.4	11.36	3831.6	1469.8	2.606885
A0A024R8/	Ubiquitinyl	MARMGDS	1	1	2	1	916	102.2	6.21	105.9	41.4	2.557971
D9YZU5	Beta-globir	MVHLTPEE	78	10	328	1	147	16	7.28	20856.2	8175.3	2.551124
A0A0U1RR	Histone H2	MSGRGKQ	14	3	13	3	170	18.5	11.52	1501.5	601.7	2.49543
P59665	Neutrophil	MRTLAILA/	19	2	29	2	94	10.2	6.99	4344.9	1746.8	2.487348
P19013	Keratin, ty	MIARQQC\	20	10	34	7	534	57.3	6.61	1858.7	765.5	2.428086
P24158	Myeloblast	MAHRPPSF	17	3	17	3	256	27.8	8.35	677.4	289.9	2.336668
A0A1K0GX	Globin C1 (	MSAARPG\	50	6	182	6	177	19.2	9.77	15770.1	6799.3	2.319371
P15104	Glutamine	MTTSASSH	2	1	1	1	373	42	6.89	58.3	26.4	2.208333
P80511	Protein S1(	MTKLEEHL	8	1	5	1	92	10.6	6.25	1638.1	750.5	2.182678
Q86SQ8	Beta-defer	GNFLTGLG	21	1	13	1	47	5.1	8.73	583.1	268.5	2.171695
P46781	40S riboso	MPVARSW	5	1	1	1	194	22.6	10.65	199.3	93.2	2.138412
P05109	Protein S1(	MLTELEKA	56	7	131	7	93	10.8	7.03	16271.7	7687.1	2.116754
B2RA03	cDNA, FLJ9	MSFTTRSTI	7	3	20	1	430	48	5.38	84.5	40.2	2.10199
P13647	Keratin, ty	MSRQSSVS	18	11	34	4	590	62.3	7.74	22.9	11	2.081818
P05787	Keratin, ty	MSIRVTQK	18	10	40	6	483	53.7	5.59	784.1	378.4	2.072146
B4E1C2	Kininogen	MKLITILFL(	36	27	3259	1	644	71.9	6.81	341.5	167.9	2.033949
A0A075B6I	Ig lambda-	XQPKAAPS	48	4	1262	1	106	11.3	8.29	249.4	126.3	1.974663
P02763	Alpha-1-ac	MALSWVL	40	7	965	5	201	23.5	5.02	95028.1	48390.7	1.963768
A1A4E9	Keratin 13	MSLRLQSS	25	14	107	9	458	49.6	4.96	6283.7	3225.2	1.948313
P08729	Keratin, ty	MSIHFSSP\	9	5	14	2	469	51.4	5.48	151.1	78.3	1.929757

B2R4M6	Protein S1(	MTCKMSQ	52	6	80	6	114	13.2	6.13	9257.9	4812.2	1.923839
P02750	Leucine-ric	MSSWSRQ	28	9	294	9	347	38.2	6.95	35646.8	19100	1.866325
P25815	Protein S1(	MTELETAM	24	2	14	2	95	10.4	4.88	1676.1	900.9	1.860473
A0A0S2Z42	HCG20398	MASTSTTIF	24	14	53	6	564	60	8	3741.2	2019.2	1.852813
A0A024RD	Lymphocyt	MARGSVSD	14	8	34	5	627	70.2	5.43	1142.3	623.7	1.831489
P12724	Eosinophil	MVPKLFTS	17	2	5	2	160	18.4	10.02	136.5	77.9	1.752246
A0A140VK	Transaldol	MSSSPVKR	6	2	5	2	337	37.5	6.81	364.6	211	1.727962
P52209	6-phospho	MAQADIAL	7	3	7	3	483	53.1	7.23	66.1	38.5	1.716883
P08246	Neutrophil	MTLGRRLA	6	2	11	2	267	28.5	9.35	579	343.6	1.685099
A0A024R3	Apolipopro	MKAAVLTL	60	16	172	16	267	30.8	5.76	9385.9	5583.4	1.681037
P19652	Alpha-1-ac	MALSWVLT	47	8	469	6	201	23.6	5.11	54229.9	32592.2	1.663892
P18428	Lipopolysa	MGALARAL	5	2	9	2	481	53.4	6.7	428.6	258	1.66124
Q9H804	cDNA FLJ1	MPGPRVW	22	8	58	1	438	48	7.46	358.9	216.2	1.660037
P05164	Myelopero	MGVPFFSS	20	13	48	13	745	83.8	8.97	2311.4	1404.2	1.646062
P48547	Potassium	MGQGDES	3	1	2	1	511	57.9	6.58	31	19	1.631579
B2R9K8	cDNA, FLJ9	MAAVKTLN	2	1	1	1	531	57.9	6.8	27.8	17.3	1.606936
A0A024R6	Alpha-1-an	MPSSVSW	53	24	629	1	418	46.7	5.59	49272.2	30932.8	1.592879
Q9UL84	Myosin-rea	EVQLVESG	28	3	29	2	122	13.6	8.46	343.2	216.8	1.583026
A8K5I6	cDNA FLJ7	MPQLLQNI	11	5	24	5	495	53.5	6.1	2625.8	1669.6	1.572712
P29373	Cellular ret	MPNFSGN	7	1	4	1	138	15.7	5.4	50.4	32.7	1.541284
A0A087WY	Proline-rich	MWRGIDSI	10	1	4	1	147	16.9	7.52	579.7	379.1	1.529148
A0A161I20	Lactoferrin	MKLVFLVLI	54	34	466	0	711	78.3	8.18	23066.2	15145.7	1.522954
V9HWA9	Epididymis	MGPTSGPS	35	55	305	55	1663	187	6.4	16259.6	10679.9	1.522449
Q5EFE6	Anti-RhD m	MRVPAQLL	40	6	2769	1	234	25.7	8.43	99.8	66	1.512121
A0A024R0	Compleme	MSACWSF	16	8	49	8	559	63.2	5.52	1810.6	1203	1.505071
A0A0B4J2D	Immunoglo	MDMRVPA	14	1	7	1	117	12.6	7.84	67.6	101.1	0.668645
G8JLH6	Tetraspani	MPVKGGTI	7	3	62	3	228	25.4	6.52	4966.4	7477.1	0.664215
Q5T1S8	Noncompa	MTTATPLG	7	1	16	1	102	11.1	9.86	1851.4	2795.3	0.662326
P55107	Growth/dif	MAHVPART	3	1	2	1	478	53.1	9.51	155	234.2	0.661827
Q5HYL0	Putative ur	MQRAVPE	1	1	2	1	967	105.3	6.83	32.2	48.9	0.658487
Q9HC84	Mucin-5B (	MGAPSACF	2	4	11	4	5762	596	6.64	538.9	821.2	0.656235
P61604	10 kDa hea	MAGQAFR	30	3	3	3	102	10.9	8.92	63.5	96.8	0.655992
A0A024RB	Solute carr	MMGSPVS	2	1	2	1	540	56.4	6.83	42.8	65.3	0.655436

Q6UX06	Olfactome	MRPGLSFL	21	10	125	10	510	57.2	5.69	4205.4	6418.7	0.655179
O00526	Uroplakin-:	MAPLLPIRT	16	2	4	2	184	19.4	10.4	111.1	169.9	0.653914
A0A024R6I	Delta-like 1	MTATEALL	11	3	41	3	383	41.2	5.53	4609.3	7066.9	0.652238
X6RBG4	Uromoduli	MRHIMTRI	39	23	3450	23	689	75.6	5.87	237342.9	364524.4	0.651103
Q96FE5	Leucine-ric	MQVSKRM	2	1	17	1	620	69.8	8.56	379.8	587.4	0.646578
P51159	Ras-relatec	MSDGDYD	4	1	2	1	221	24.9	5.22	123.1	190.9	0.64484
Q99835	Smoothene	MAAARPAI	1	1	4	1	787	86.3	8.34	232.8	361.7	0.643627
P22732	Solute carr	MEQQDQS	6	3	22	3	501	54.9	6.04	903.4	1407.4	0.641893
P23468	Receptor-t	MVHVARLL	2	3	3	2	1912	214.6	6.57	31	48.4	0.640496
B4DM05	cDNA FLJ5:	MLASSSRIF	17	12	155	2	726	79.3	4.97	2472.9	3873.7	0.638382
B2R880	cDNA, FLJ9	MANCEFSF	4	1	3	1	300	34.3	7.47	28.1	44.1	0.637188
Q96ID5	Immunogl	MRTAPSLR	2	1	1	1	467	51.8	6.93	22.6	35.5	0.63662
P06870	Kallikrein-1	MWFLVLCL	23	6	493	6	262	28.9	4.83	19938.5	31904.2	0.624949
O95460	Matrilin-4 (	MRGLLCW	4	2	21	2	622	68.4	6.1	491.1	789.3	0.622197
Q13508	Ecto-ADP-r	MKTGHFEI	5	2	58	2	389	43.9	6.06	2456.4	3963.6	0.61974
A1L471	ATP-bindin	MDLEGDRI	2	2	3	2	1280	141.4	9	74	121	0.61157
P55000	Secreted L	MASRWAV	61	3	21	3	103	11.2	5.33	399.8	663.4	0.602653
B2RDY9	Adenylyl cy	MADMQNI	4	2	2	2	475	51.6	8.22	157.4	273.4	0.575713
A0A096LN	Uncharacte	MALTKMLI	6	1	6	1	201	23.3	9.38	151	272.3	0.554535
Q8N4F0	BPI fold-co	MAWASRL	7	3	15	3	458	49.1	8.72	538.1	975	0.551897
O43653	Prostate st	MKAVLLAL	22	2	164	2	123	12.9	5.29	7892.3	14644.6	0.538922
O60662	Kelch-like p	MDSQRELA	1	1	2	1	606	68	5.29	100.6	188	0.535106
B7ZLW2	HELZ prote	MEDRRAEK	1	1	2	1	1943	219	7.42	12.8	24.3	0.526749
Q96AX9	E3 ubiquiti	MGWKPSE	1	1	4	1	1013	109.9	8.44	45.5	88.1	0.516459
M0R1F0	Prostate-sp	XFLTLSVTW	52	10	493	9	262	28.6	7.44	16496.1	34544.1	0.477537
B2R7D1	cDNA, FLJ9	MMDASKE	2	2	7	2	684	77.1	6.76	17.2	39.4	0.436548
Q5IWS5	Intelectin 1	MNQLSFLL	16	4	13	4	313	35	5.82	316.1	793.9	0.398161
P15309	Prostatic a	MRAAPLLL	32	13	827	13	386	44.5	6.24	22654.6	60390	0.375138
B2R597	cDNA, FLJ9	MNVLLGSV	14	2	78	2	114	12.8	5.5	4333.9	12181.9	0.355766
