# Supplementary material for: LC-MS/MS-Based Quantitative Proteomics Analysis of Different Stages of Non-Small-Cell Lung Cancer
Source: Biomed Res Int. 2021 Feb 26;2021:5561569. doi: 10.1155/2021/5561569 (PMC7937045; doi:10.1155/2021/5561569)
Supplement: Supplementary 3 — Table S3: a further study on the LC-MS analysis results of urine in Table S2. Urine samples from healthy controls were compared to urine samples from different lung adenocarcinoma groups to obtain differential protein levels. [file 5561569.f3.pdf]

Uniprot Acc	protiens	121	113	ratio(113/12	114	ratio(114/12	115	ratio(115/12	116	ratio(116/12	117	ratio(117/12	118	ratio(118/12	119	ratio(119/121)
P02768	Serum albumin OS=Homo s	666397.2	862466.6	1.29	593482.1	0.89	2220420.5	3.33	846469.3	1.27	634302.5	0.95	736961.1	1.11	842297.5	1.26
P02760	Protein AMBP OS=Homo s	381627.7	294812.3	0.77	474165.8	1.24	203377.3	0.53	349197.6	0.92	346271.7	0.91	328356.1	0.86	411229.3	1.08
B4E1B2	cDNA FLJ53691, highly sin	78511.8	107560.1	1.37	85218.8	1.09	199640.4	2.54	90022.3	1.15	73427.3	0.94	82722	1.05	102491.8	1.31
A0A0K0K1	Epididymis secretory sperm	3358.1	4584.8	1.37	4234.8	1.26	8496.1	2.53	4193.7	1.25	3507.1	1.04	3632.2	1.08	4873.3	1.45
D3DNU8	Kininogen 1, isoform CRA_	302648.3	294936.6	0.97	257956.9	0.85	193831.3	0.64	290406.7	0.96	313453.3	1.04	279805.5	0.92	228883.9	0.76
B4E1C2	Kininogen 1, isoform CRA_	167.9	229.7	1.37	263.2	1.57	278	1.66	326	1.94	265.5	1.58	290.3	1.73	341.5	2.03
O60494	Cubilin OS=Homo sapiens (	40132.6	45037.5	1.12	38654.5	0.96	34055.8	0.85	39708.6	0.99	44702.9	1.11	45025.7	1.12	32701.3	0.81
P98164	Low-density lipoprotein rec	40915.4	45344.8	1.11	35817.7	0.88	35721.8	0.87	42157.7	1.03	46693.4	1.14	48624	1.19	38847.7	0.95
A0A024RA	Heparan sulfate proteoglyca	46985.3	43536.1	0.93	49827.7	1.06	33964.3	0.72	44780.9	0.95	46858.6	1.00	47703.5	1.02	37785.7	0.80
P01133	Pro-epidermal growth factor	75270.8	73109.5	0.97	66336.1	0.88	56680.9	0.75	70877.8	0.94	76444.3	1.02	81110.3	1.08	65749.7	0.87
Q6N093	Putative uncharacterized pro	47825.3	55632.5	1.16	48428.3	1.01	63601.5	1.33	65204.2	1.36	59384.5	1.24	40148.4	0.84	58939.1	1.23
X6RBG4	Uromodulin OS=Homo sapi	364524.4	267652.8	0.73	169355	0.46	173051.8	0.47	249815.6	0.69	272871.5	0.75	323278.4	0.89	237342.9	0.65
S4R471	Protein AMBP (Fragment) (	913.4	830.2	0.91	1013.7	1.11	501.8	0.55	900.2	0.99	865.2	0.95	813.1	0.89	1077	1.18
Q6MZQ6	Putative uncharacterized pro	150720	156851.3	1.04	141749.9	0.94	138599.9	0.92	186246.8	1.24	144680.4	0.96	140760.2	0.93	177689.2	1.18
A0A024R46	Fibronectin 1, isoform CRA	23276.7	23599.9	1.01	22486.3	0.97	17512.3	0.75	26256.4	1.13	24031.8	1.03	23591	1.01	23870.8	1.03
P04746	Pancreatic alpha-amylase OS	2613.9	3567.6	1.36	1554.5	0.59	3116.9	1.19	3014.4	1.15	3868.6	1.48	3474.2	1.33	2469.6	0.94
P01833	Polymeric immunoglobulin r	75401.1	65223.8	0.87	51222.8	0.68	44831.7	0.59	57440.9	0.76	84481.8	1.12	65065.7	0.86	60349.2	0.80
V9HW34	Epididymis luminal protein	908.7	787.8	0.87	1077.1	1.19	619	0.68	873.4	0.96	900.6	0.99	903.4	0.99	875.5	0.96
Q6PIL8	IGK@ protein OS=Homo sa	3364.6	3026.6	0.90	3563	1.06	2074.2	0.62	2760.8	0.82	3139.4	0.93	3486.2	1.04	3443.1	1.02
Q6P5S8	IGK@ protein OS=Homo sa	183839.5	151922.2	0.83	222216.3	1.21	121290.3	0.66	166687	0.91	160695.3	0.87	198000.3	1.08	194547.5	1.06
Q0KKI6	Immunoblobulin light chain	4700.9	3800.1	0.81	4394.4	0.93	2855.3	0.61	3317	0.71	4193.4	0.89	4965.8	1.06	4898.1	1.04
Q59E93	Membrane alanine aminope	32198.4	34854.5	1.08	27418	0.85	27084.2	0.84	34101	1.06	35756.3	1.11	28833.9	0.90	28075.9	0.87
B7ZMD7	Alpha-amylase OS=Homo s	26254.5	36477.1	1.39	19818.9	0.75	29799.8	1.14	33533.9	1.28	37893.7	1.44	36147.9	1.38	26885.5	1.02
A0A161I20	Lactoferrin OS=Homo sapie	15145.7	39936.6	2.64	20213.2	1.33	13042.9	0.86	22989.8	1.52	19645.5	1.30	23345.6	1.54	23066.2	1.52
A0A024R8	Glucosidase, alpha acid (Po	26196.1	26278.3	1.00	20740.9	0.79	19305.1	0.74	24136.9	0.92	25723	0.98	25941.7	0.99	21714.1	0.83
P00734	Prothrombin OS=Homo sap	39265.9	36023.3	0.92	30475.5	0.78	25009.6	0.64	33369.7	0.85	35911.5	0.91	37733	0.96	35981.5	0.92
Q96K68	cDNA FLJ14473 fis, clone	63862.1	60465.5	0.95	47280	0.74	46205.5	0.72	55632	0.87	65877.7	1.03	56589.8	0.89	57673.3	0.90
B3VMW0	Lactoferrin OS=Homo sapie															
W8QEY1	Lactoferrin OS=Homo sapie	203	227.3	1.12	174.7	0.86	186.3	0.92	323.9	1.60	207.4	1.02	189.2	0.93	220.6	1.09
V9HWA9	Epididymis secretory sperm	10679.9	17774.3	1.66	14518.8	1.36	19834.7	1.86	14532.1	1.36	11925	1.12	17333.2	1.62	16259.6	1.52
Q5EFE6	Anti-RhD monoclonal T125	66	63.8	0.97	94.1	1.43	46.1	0.70	87.4	1.32	55.9	0.85	65.6	0.99	99.8	1.51
A0A024RD	Secreted phosphoprotein 1 (	70922.1	58597	0.83	41336.7	0.58	33620	0.47	55794.7	0.79	61223.2	0.86	73467.6	1.04	49830.6	0.70
Q59EG0	Basement membrane-specifi	1737.4	1510.7	0.87	1463.9	0.84	1070.7	0.62	2426.2	1.40	2042.3	1.18	3369.3	1.94	1566.4	0.90
A0A140TA	Tenascin-X OS=Homo sapi	15142.2	15479.7	1.02	13776.6	0.91	11588.2	0.77	14985.1	0.99	15236.8	1.01	14675.1	0.97	12717	0.84
Q6N092	Putative uncharacterized pro	292.1	212.3	0.73	170.6	0.58	187.3	0.64	215.6	0.74	195.9	0.67	161.8	0.55	243	0.83
Q8NCL6	cDNA FLJ90170 fis, clone	95.9	111.4	1.16	103.6	1.08	88.7	0.92	97	1.01	97.6	1.02	94.8	0.99	97.1	1.01
E9KL23	Epididymis secretory sperm	1716	2619.7	1.53	1850.8	1.08	4131.1	2.41	1940.5	1.13	1781.5	1.04	1987.8	1.16	2198.3	1.28
P25311	Zinc-alpha-2-glycoprotein C	151729.7	103361.5	0.68	236874.5	1.56	90208.1	0.59	112762.6	0.74	114953.3	0.76	87034.2	0.57	132935.6	0.88
A0A024RD	Secreted phosphoprotein 1 (	9894.7	7830	0.79	6811.9	0.69	4818.4	0.49	8363.9	0.85	8910.8	0.90	10063.6	1.02	8087.2	0.82
A0A024R6I	Alpha-1-antitrypsin OS=Ho	30932.8	44423.8	1.44	33176.1	1.07	80233.1	2.59	38125.8	1.23	36761.6	1.19	37927.4	1.23	49272.2	1.59
P35555	Fibrillin-1 OS=Homo sapier	24928.2	24349.7	0.98	20530	0.82	19914.2	0.80	23308.7	0.94	24593.8	0.99	22820.3	0.92	21604.6	0.87
P12109	Collagen alpha-1(VI) chain	31949.7	33324.9	1.04	28063	0.88	23704.5	0.74	29432.1	0.92	35827.7	1.12	35265.8	1.10	28378.3	0.89
A8KAJ3	cDNA FLJ77823, highly sin	26275.3	24208.6	0.92	28686.9	1.09	17831.4	0.68	24959.9	0.95	27916.5	1.06	24654.5	0.94	21521.2	0.82
A0A0S2Z3Y	Lectin galactoside-binding s	27682.3	24440.8	0.88	21860.8	0.79	20279.5	0.73	22939.6	0.83	26958.2	0.97	29734.9	1.07	24870.3	0.90
E7ER45	Maltase-glucoamylase, intes	17395.7	20741	1.19	15799.2	0.91	15308.5	0.88	19737.9	1.13	19120.9	1.10	20698.3	1.19	17321	1.00
A0A024R8	Prostaglandin D2 synthase 2	157265.4	132959.1	0.85	249501.5	1.59	92848.1	0.59	168444.8	1.07	144643.5	0.92	167428.8	1.06	131771.6	0.84
P15309	Prostatic acid phosphatase (	60390	44629.5	0.74	66404	1.10	57259.6	0.95	45211.6	0.75	65647.6	1.09	24586.7	0.41	22654.6	0.38
P02763	Alpha-1-acid glycoprotein 1	48390.7	35706.4	0.74	76375	1.58	34617.4	0.72	42550.7	0.88	30650.9	0.63	32510.1	0.67	95028.1	1.96
A8K5A4	cDNA FLJ76826, highly sin	14909.8	16788.8	1.13	14281.1	0.96	18971.2	1.27	15934.6	1.07	14697.8	0.99	16077.5	1.08	20626.8	1.38
A0A0S2Z3V	EGF containing fibulin-like	118.6	164.5	1.39	149.8	1.26	117.7	0.99	158	1.33	151.2	1.27	239.5	2.02	123.5	1.04
P01861	Ig gamma-4 chain C region	4247.9	4652.6	1.10	6314.7	1.49	6267.9	1.48	4620.4	1.09	4081.8	0.96	4058.3	0.96	5555.8	1.31
A0A024R6N	Serpin peptidase inhibitor, c	27414.4	30478.7	1.11	25865.8	0.94	26139.5	0.95	30958.3	1.13	33303.9	1.21	33812	1.23	29852	1.09
Q16270	Insulin-like growth factor-bi	39312.4	38283.2	0.97	32001.3	0.81	27463.2	0.70	32943.9	0.84	37954.8	0.97	37956.2	0.97	29811.2	0.76
A0A024RD.	Secreted phosphoprotein 1 (	86.6	77.9	0.90	67.3	0.78	75.4	0.87	76.2	0.88	78.8	0.91	98	1.13	74.1	0.86
P04279	Semenogelin-1 OS=Homo s	12317.9	16697.1	1.36	19411.5	1.58	10585.7	0.86	60356.9	4.90	13960	1.13	9603.9	0.78	8685.3	0.71
P00738	Haptoglobin OS=Homo sap	22466.7	23271.4	1.04	32501.8	1.45	26124.5	1.16	22935.7	1.02	18392	0.82	29883.6	1.33	86108.9	3.83
A0A0G2JPF	Complement C4-A OS=Hor	7727.2	8787.4	1.14	9127.2	1.18	7777.3	1.01	8628.3	1.12	9221.3	1.19	9747	1.26	9042	1.17

W0UV60	Ribonuclease A F3 OS=Hor	45540.4	40770.4	0.90	50060	1.10	31349.3	0.69	49333.6	1.08	39027.6	0.86	51040	1.12	35397.7	0.78
P0C0L5	Complement C4-B OS=Hor	344.9	332.7	0.96	299	0.87	292.7	0.85	364.7	1.06	344.8	1.00	388.6	1.13	450.4	1.31
O75882	Attractin OS=Homo sapiens	12416	12022.7	0.97	10543.3	0.85	9668.8	0.78	11707.2	0.94	12428.8	1.00	11456.7	0.92	10342.8	0.83
A8K3U3	cDNA FLJ77863, highly sin	27654.4	29720.3	1.07	38789.3	1.40	23338.3	0.84	31760.1	1.15	30231.1	1.09	39678.5	1.43	27395.3	0.99
P02790	Hemopexin OS=Homo sapie	27113.3	30288.1	1.12	32208	1.19	29317.9	1.08	26373	0.97	27211.1	1.00	29882.3	1.10	30681.3	1.13
P10909	Clusterin OS=Homo sapiens	29940.2	32485.9	1.09	31163	1.04	27176	0.91	31832.6	1.06	38559.5	1.29	33439.7	1.12	32173.1	1.07
A8K7T4	cDNA FLJ75774, highly sin	57175.3	51924.6	0.91	60344.1	1.06	37340.5	0.65	58631.6	1.03	57770.6	1.01	63919.2	1.12	64303.9	1.12
P54802	Alpha-N-acetylglucosaminic	8664.6	9813.9	1.13	7723.2	0.89	6953.8	0.80	8759.3	1.01	9550.4	1.10	8998.8	1.04	7625.2	0.88
P63261	Actin, cytoplasmic 2 OS=Ho	11164.5	16761	1.50	11559.1	1.04	10082.5	0.90	12801.4	1.15	16140	1.45	15279.7	1.37	13624	1.22
E9PGN7	Plasma protease C1 inhibito	37295.4	38639.8	1.04	32778.5	0.88	29434.6	0.79	35619.4	0.96	43925.7	1.18	38370.4	1.03	33662.9	0.90
S6BGD6	IgG L chain OS=Homo sapi	1427.4	1291.3	0.90	1704.5	1.19	1076.6	0.75	1469.4	1.03	1395.2	0.98	1573.3	1.10	1729.3	1.21
P06396	Gelsolin OS=Homo sapiens	25485.9	22734.5	0.89	33262.8	1.31	17664.8	0.69	27384.1	1.07	28500	1.12	26508.5	1.04	25927.5	1.02
P00747	Plasminogen OS=Homo sap	15357.1	16945.7	1.10	16062.8	1.05	14786.9	0.96	15645.1	1.02	16065.9	1.05	14859.2	0.97	17672.5	1.15
B7ZKJ8	ITIH4 protein OS=Homo sa	35519.9	36663.7	1.03	32179.9	0.91	31430.2	0.88	38048	1.07	39069.7	1.10	40363	1.14	40159	1.13
Q8N355	IGL@ protein OS=Homo sa	194.2	138.3	0.71	270.6	1.39	104	0.54	167.5	0.86	126.6	0.65	135	0.70	176.5	0.91
B4DPH5	cDNA FLJ55496, highly sin	31.5	22.1	0.70	22.6	0.72	36.2	1.15	46.9	1.49	25.7	0.82	16.7	0.53	38.3	1.22
A2NUT2	Lambda-chain (AA -20 to 2	101417.1	86013.1	0.85	177227.2	1.75	72587.9	0.72	126455.5	1.25	97513.4	0.96	122835.2	1.21	118501.6	1.17
Q6GMV8	Uncharacterized protein OS	4273.6	3551.6	0.83	6709.7	1.57	3229.9	0.76	4419.4	1.03	3762	0.88	5315.5	1.24	4920.8	1.15
P05062	Fructose-bisphosphate aldol	10140.5	14342.3	1.41	10885.6	1.07	8662.9	0.85	10906.9	1.08	14848.6	1.46	12235.8	1.21	10101.6	1.00
B7Z6Q5	Beta-galactosidase OS=Hon	6953.9	10000.1	1.44	5876.3	0.85	6354.8	0.91	8239.7	1.18	8969.4	1.29	9596.4	1.38	7496.3	1.08
Q569I7	Uncharacterized protein OS	134.5	145.7	1.08	136.2	1.01	114	0.85	110.6	0.82	119.4	0.89	158.6	1.18	169.9	1.26
S6B294	IgG L chain OS=Homo sapi	43	44.7	1.04	58	1.35	40.4	0.94	38.9	0.90	48.2	1.12	39.1	0.91	43.1	1.00
Q6GMX4	IGL@ protein OS=Homo sa	479.1	500.4	1.04	544.7	1.14	460.9	0.96	521	1.09	464.8	0.97	528.8	1.10	551	1.15
S6AWD6	IgG L chain OS=Homo sapi	194.6	233.9	1.20	216.7	1.11	193.5	0.99	205.1	1.05	189.3	0.97	236.3	1.21	207.4	1.07
S6AWD3	IgG L chain OS=Homo sapi	830	1018	1.23	1199.2	1.44	751.7	0.91	1491.4	1.80	928.4	1.12	992.7	1.20	1199.5	1.45
Q8NBJ4	Golgi membrane protein 1 C	10165.7	9235.2	0.91	7610.4	0.75	7144.5	0.70	9294.5	0.91	10319.8	1.02	9231.9	0.91	7727.5	0.76
S6BGE9	IgG L chain OS=Homo sapi	2152.9	2212.7	1.03	2856	1.33	1550.1	0.72	2192.6	1.02	2079.4	0.97	2231	1.04	2508.7	1.17
Q6IPQ0	IGL@ protein OS=Homo sa															
V9HWI3	Cathepsin D (Lysosomal asp	13018.8	15711.4	1.21	12359.9	0.95	11091.3	0.85	14318	1.10	17143.2	1.32	16500.5	1.27	14170.4	1.09
Q6PIK1	IGL@ protein OS=Homo sa	6906.4	6290.4	0.91	8731	1.26	5542.3	0.80	6796.3	0.98	6542	0.95	7580.1	1.10	7764.1	1.12
D6RF35	Vitamin D-binding protein (	14294.4	17327.4	1.21	13533.7	0.95	24489.9	1.71	15861.2	1.11	15123.2	1.06	15500.5	1.08	16584.4	1.16
V9HWD8	Epididymis secretory sperm	24056.7	21216.9	0.88	26602.6	1.11	21869.4	0.91	21043.9	0.87	20167.4	0.84	21694.9	0.90	31421	1.31
P02749	Beta-2-glycoprotein 1 OS=H	26758.6	22534.1	0.84	19621.2	0.73	12893.6	0.48	20707.4	0.77	27931.5	1.04	20500.3	0.77	20266.6	0.76
D9ZGG2	Vitronectin OS=Homo sapie	21122.6	21742.7	1.03	22367.4	1.06	14848.5	0.70	24088.8	1.14	22575.8	1.07	22267.7	1.05	25547.6	1.21
Q5CZ94	Putative uncharacterized pro	145.8	135.2	0.93	121.7	0.83	147.8	1.01	161.2	1.11	102.2	0.70	148.6	1.02	136.4	0.94
Q6DHW4	Uncharacterized protein OS															
B3KS79	cDNA FLJ35730 fis, clone T	16211.1	13162.9	0.81	14201.8	0.88	12691.8	0.78	14632.2	0.90	14459.7	0.89	13588.8	0.84	22531.2	1.39
P07998	Ribonuclease pancreatic OS	25347.2	20596.6	0.81	20328.4	0.80	13152.6	0.52	21895.2	0.86	21754.4	0.86	19182.2	0.76	17946.9	0.71
C9JF17	Apolipoprotein D (Fragmen	125321.7	116695.1	0.93	109434.1	0.87	75109.3	0.60	109701.3	0.88	141500.2	1.13	116202.4	0.93	121145	0.97
Q8WZ75	Roundabout homolog 4 OS=	13999.1	14015.7	1.00	12359.8	0.88	11027.9	0.79	13649.4	0.98	14279	1.02	16103.8	1.15	13958.1	1.00
M0R1F0	Prostate-specific antigen (Fr	34544.1	32571.8	0.94	40315.5	1.17	30526	0.88	26632.8	0.77	29508.2	0.85	16031.4	0.46	16496.1	0.48
Q6N030	Uncharacterized protein OS	7211.3	5074.5	0.70	4738.1	0.66	5540.6	0.77	6024.4	0.84	6131	0.85	5122.6	0.71	7072.7	0.98
Q8NFZ8	Cell adhesion molecule 4 OS	9424.4	8883.2	0.94	8270.9	0.88	6499.1	0.69	9017.3	0.96	8820.9	0.94	9361.4	0.99	7196.8	0.76
B7Z9B1	cDNA FLJ52398, highly sin	28059.6	23981.7	0.85	26643.4	0.95	16251.4	0.58	25265.4	0.90	25342.3	0.90	24623.6	0.88	22206.7	0.79
O94919	Endonuclease domain-conta	9024.3	8326.9	0.92	11662	1.29	6551.2	0.73	8645.8	0.96	8894.1	0.99	8187	0.91	8210.1	0.91
P02753	Retinol-binding protein 4 OS	26946	28771.5	1.07	58084.3	2.16	20772.3	0.77	29296.9	1.09	33777.9	1.25	30653.1	1.14	22817.5	0.85
B7Z5V6	cDNA FLJ57046, highly sin	2002.3	2519.2	1.26	1906.2	0.95	2285.5	1.14	1916.7	0.96	2317.2	1.16	2148.9	1.07	1547.7	0.77
E9PR17	CD59 glycoprotein OS=Hor	112175.1	90140.3	0.80	94116.1	0.84	66869	0.60	98283.8	0.88	104681.9	0.93	107610.9	0.96	86285.3	0.77
O00391	Sulfhydryl oxidase 1 OS=Ho	5955.7	6795.3	1.14	6010	1.01	5442	0.91	6302.2	1.06	6408.7	1.08	6427.3	1.08	5843.9	0.98
P27487	Dipeptidyl peptidase 4 OS=	8683.5	9130.5	1.05	8022.4	0.92	7844.8	0.90	9113.7	1.05	9502.2	1.09	8394.4	0.97	7506.1	0.86
H6VRG1	Keratin 1 OS=Homo sapiens	9226.5	9476	1.03	6231.3	0.68	7005	0.76	5944.3	0.64	7754.1	0.84	8608.7	0.93	8670.3	0.94
A8K335	cDNA FLJ76254, highly sin	15333.5	17957	1.17	12883	0.84	11143.4	0.73	15620.3	1.02	18357.3	1.20	20282.1	1.32	15601.1	1.02
B7Z8Q2	cDNA FLJ55606, highly sin	31137.6	29115	0.94	47211	1.52	22248.8	0.71	32847	1.05	34937.8	1.12	31691.9	1.02	33256.5	1.07
B2R582	cDNA, FLJ92374, highly si	589	535	0.91	463.1	0.79	497.3	0.84	429.5	0.73	579.2	0.98	442.2	0.75	445.4	0.76
B2R888	Monocyte differentiation an	22517.1	19350.3	0.86	34324.4	1.52	13265.4	0.59	21759.3	0.97	24036.9	1.07	22120.8	0.98	31587.4	1.40
A8K3K1	cDNA FLJ78096, highly sin	1132.8	1376.3	1.21	972.4	0.86	929.4	0.82	1205.8	1.06	1637.7	1.45	1245.8	1.10	1169.5	1.03
B4E1Z4	cDNA FLJ55673, highly sin	4174.9	5327	1.28	7433.3	1.78	3756.4	0.90	4316	1.03	5048.3	1.21	4986.3	1.19	4293	1.03
A8K6Z6	cDNA FLJ78262, highly sin	2968.1	3808.1	1.28	4746.6	1.60	2950.2	0.99	13370.1	4.50	3415.1	1.15	3196.1	1.08	2643.1	0.89

D9YZU5	Beta-globin OS=Homo sapi	8175.3	44720.1	5.47	11673.8	1.43	7891.1	0.97	10959.3	1.34	20029.3	2.45	18593.9	2.27	20856.2	2.55
O00468	Agrin OS=Homo sapiens Gl	6549.9	6556.5	1.00	9170.1	1.40	5380.9	0.82	6510.6	0.99	6654.7	1.02	6845.8	1.05	6884.2	1.05
E7ETH0	Complement factor I OS=H	7242.1	7030	0.97	6804.8	0.94	6327.9	0.87	6590.3	0.91	7519.4	1.04	7774.9	1.07	6675.9	0.92
A0A1B0GU	Ig mu chain C region (Fragn	20735.1	23674.6	1.14	25076.2	1.21	19022.9	0.92	23987	1.16	25065.2	1.21	30377.4	1.47	22344.8	1.08
B2RCQ6	cDNA, FLJ96222, highly si	5058.9	7840.2	1.55	5243.9	1.04	5523.3	1.09	5621.9	1.11	6657.2	1.32	6307	1.25	4825.4	0.95
B7ZKY6	Membrane metallo-endopep	6746.4	7337.4	1.09	6212	0.92	6066.5	0.90	6723.5	1.00	7573.8	1.12	6489.3	0.96	5345.8	0.79
A0A0E3XJU	E-cadherin 1 OS=Homo sap	30186.4	23890.7	0.79	28629.9	0.95	19631.3	0.65	25363	0.84	24789.7	0.82	30539	1.01	31002.3	1.03
E7ET40	Urokinase-type plasminogen	8753.8	10043	1.15	10868	1.24	7972	0.91	9409.9	1.07	10646.9	1.22	10681.4	1.22	9738.5	1.11
A8K6V6	N-acetylglucosamine-6-sulfa	14806.6	14130.6	0.95	14474.9	0.98	9677.9	0.65	15612.1	1.05	14594.5	0.99	14408.2	0.97	14409.1	0.97
V9HW22	Epididymis luminal protein	5169.2	6176.3	1.19	4915.2	0.95	4402.9	0.85	5382.5	1.04	5784.9	1.12	5413.1	1.05	5273.7	1.02
P28799	Granulins OS=Homo sapien	8962.9	13200.6	1.47	8566.9	0.96	8117.2	0.91	10492.5	1.17	10470.4	1.17	10819.4	1.21	9760.5	1.09
B0YJC6	Vitamin K-dependent protei	16253.9	18393.1	1.13	16104.7	0.99	13385.9	0.82	18155.4	1.12	15686.5	0.97	19476.2	1.20	15480.4	0.95
A2KBC4	Anti-TN-C scFv (Fragment)	4996.3	5443.3	1.09	4535.9	0.91	4904.6	0.98	5186.3	1.04	5280.6	1.06	5058.7	1.01	5224.3	1.05
P78492	Inter-alpha-trypsin inhibitor	820	676.4	0.82	874.5	1.07	417.2	0.51	801.5	0.98	703.3	0.86	628.9	0.77	910.5	1.11
Q7Z3B1	Neuronal growth regulator 1	5405.1	4784.5	0.89	5127	0.95	3566.5	0.66	5021.4	0.93	5158.7	0.95	5011.7	0.93	4610.3	0.85
S6BAR0	IgG L chain OS=Homo sapi	1988.2	1661.8	0.84	2367.8	1.19	1268.6	0.64	1681.7	0.85	1674.3	0.84	3364.4	1.69	2416	1.22
P13645	Keratin, type I cytoskeletal 1	11461.9	11061.6	0.97	7528.5	0.66	9154.3	0.80	7392.4	0.64	10137.9	0.88	11772.1	1.03	10809.6	0.94
Q9Y6R7	IgGFc-binding protein OS=l	4182.5	4069	0.97	3160.2	0.76	2487.8	0.59	2981.7	0.71	5357.1	1.28	2513.9	0.60	3199	0.76
A0A024QZ(	Prosaposin (Variant Gauche	44266.4	51916	1.17	36858	0.83	28343.4	0.64	40853.7	0.92	45275.1	1.02	43156.2	0.97	50370.4	1.14
P06870	Kallikrein-1 OS=Homo sapi	31904.2	29532.8	0.93	26696.4	0.84	24544.6	0.77	30067.2	0.94	37889.1	1.19	27163.4	0.85	19938.5	0.62
A0A024R94	Serpin peptidase inhibitor, c	6197	7926.4	1.28	9396.9	1.52	10940.4	1.77	6630.6	1.07	6582.5	1.06	6966.3	1.12	7629.3	1.23
A0A024R2(	C-type lectin domain family	10624.9	9181.9	0.86	7965.2	0.75	7486.8	0.70	8543.1	0.80	10248.6	0.96	8645.4	0.81	8053.7	0.76
B2RBF5	cDNA, FLJ95483, highly si	9972.7	9872.2	0.99	10090.3	1.01	7463.7	0.75	10399.1	1.04	9454	0.95	10323.2	1.04	10729.7	1.08
X6R868	Bile salt-activated lipase OS	10342.8	13296.1	1.29	8155.6	0.79	9041.5	0.87	11132.9	1.08	10893.4	1.05	13786.7	1.33	8731.9	0.84
A0A087WW	Tenascin-X OS=Homo sapie															
Q15828	Cystatin-M OS=Homo sapie	7481.9	7456	1.00	14974.4	2.00	6224.4	0.83	8778.4	1.17	8816.8	1.18	8199.9	1.10	6846.3	0.92
Q5FWF9	IGL@ protein OS=Homo sa	3111.9	2832.6	0.91	5027.3	1.62	2277.8	0.73	3809.7	1.22	3261.9	1.05	3448.2	1.11	3768.6	1.21
P22792	Carboxypeptidase N subunit	9129.5	8418.9	0.92	7375.9	0.81	6103.8	0.67	8306.7	0.91	10519.2	1.15	10166.9	1.11	8366.4	0.92
B4DJQ8	cDNA FLJ55694, highly sin	7815.2	9491.6	1.21	7696.2	0.98	7051.4	0.90	8465	1.08	9227.7	1.18	8902.9	1.14	7872.9	1.01
E7ETN3	Uncharacterized protein OS	56.9	47.1	0.83	42.5	0.75	34.2	0.60	44.4	0.78	44.1	0.78	47.4	0.83	62.1	1.09
O75594	Peptidoglycan recognition p	10051.6	11315.7	1.13	10968.1	1.09	8389.1	0.83	10397.2	1.03	11176.1	1.11	10271.8	1.02	9934.7	0.99
P60174	Triosephosphate isomerase (	5034.8	6322.4	1.26	4996.5	0.99	3888.3	0.77	5335.4	1.06	6184.5	1.23	5511.8	1.09	5547.9	1.10
A0A075B6I	Ig lambda-7 chain C region	126.3	134.1	1.06	314.6	2.49	119.4	0.95	195.9	1.55	191.2	1.51	150.6	1.19	249.4	1.97
P24855	Deoxyribonuclease-1 OS=H	19469.6	19120.1	0.98	10550.1	0.54	14451.1	0.74	18482.7	0.95	19902.9	1.02	20304.1	1.04	14070.1	0.72
A8K2P8	cDNA FLJ76245, highly sin	4611.3	4005.2	0.87	4797.7	1.04	3005.9	0.65	4284.3	0.93	4544.7	0.99	4309.2	0.93	4329.2	0.94
Q6LAM1	Heavy chain of factor I (Fra	272.9	264	0.97	172.8	0.63	174.9	0.64	218.4	0.80	264.4	0.97	229.1	0.84	317	1.16
D9IAI1	Epididymis secretory protei	10023.5	10704.4	1.07	8666.2	0.86	8083.9	0.81	9207.2	0.92	11407.9	1.14	9249.3	0.92	8762.1	0.87
A8K7G6	cDNA FLJ75763, highly sin	24072.4	20747.4	0.86	67081.8	2.79	15740.4	0.65	21956.8	0.91	29098.7	1.21	25074.5	1.04	21249.8	0.88
A0A024R3E	Apolipoprotein A-I, isoform	5583.4	14099.8	2.53	8339.7	1.49	10752.4	1.93	7017	1.26	6795.4	1.22	13017.8	2.33	9385.9	1.68
A0A0K0K1.	Cystatin OS=Homo sapiens	22034.6	23609.1	1.07	20362.2	0.92	17781	0.81	20549.9	0.93	25344.5	1.15	22440.5	1.02	20095	0.91
P06733	Alpha-enolase OS=Homo sa	7328.1	9197.9	1.26	7512.7	1.03	6104.5	0.83	7530.6	1.03	9779.6	1.33	8699.9	1.19	8416.6	1.15
A0A024R0A	Matrix-remodelling associat	13392.9	14135.6	1.06	13360.5	1.00	9861.6	0.74	13081.8	0.98	14067.5	1.05	13527.6	1.01	14201.3	1.06
P19652	Alpha-1-acid glycoprotein 2	32592.2	28760.9	0.88	42980.5	1.32	28097.3	0.86	30432.2	0.93	25362.8	0.78	22890.2	0.70	54229.9	1.66
Q7Z379	Putative uncharacterized pro	2134.7	2416.1	1.13	1625.7	0.76	1794.5	0.84	2023.5	0.95	1993	0.93	2266	1.06	2332.7	1.09
B0BCY7	Glutamyl aminopeptidase (A	6108.5	6396.6	1.05	4871	0.80	4526.1	0.74	7544.2	1.24	7169.7	1.17	6138.2	1.00	6823.2	1.12
A0A125QY	GCT-A9 light chain variable	2485.4	2491.2	1.00	3158.7	1.27	1686.8	0.68	3206.8	1.29	2375.6	0.96	2437.5	0.98	3373.3	1.36
P02649	Apolipoprotein E OS=Homo	4851	6715.5	1.38	5542.8	1.14	4969	1.02	5478	1.13	6541.8	1.35	6263.6	1.29	5132.9	1.06
P11117	Lysosomal acid phosphatase	9940.3	11102.1	1.12	8212.2	0.83	7771.6	0.78	9738.9	0.98	9839.5	0.99	11705	1.18	8307.8	0.84
P06727	Apolipoprotein A-IV OS=H	5285.4	8194	1.55	8534.3	1.61	6329.7	1.20	5770.7	1.09	6111.2	1.16	7110.2	1.35	6475	1.23
O76076	WNT1-inducible-signaling p	2026.8	2354.8	1.16	2240.5	1.11	1497.1	0.74	2188.9	1.08	2228.2	1.10	1983	0.98	1876.3	0.93
Q14508	WAP four-disulfide core do	13168.2	12289.6	0.93	10492.9	0.80	8609.3	0.65	12185.4	0.93	13100.8	0.99	13006.5	0.99	15482.9	1.18
P04083	Annexin A1 OS=Homo sapi	3672	3502.7	0.95	3667.8	1.00	2825.3	0.77	5595.3	1.52	4147.6	1.13	4064.7	1.11	5350.8	1.46
Q9UNN8	Endothelial protein C recept	21715.2	19919.3	0.92	17402.5	0.80	15700	0.72	21225.8	0.98	18474.6	0.85	20438.6	0.94	21937.6	1.01
P18065	Insulin-like growth factor-bi	5620.8	6669.4	1.19	6714.4	1.19	5207	0.93	5987.2	1.07	6522.1	1.16	7067.8	1.26	6039.7	1.07
P14543	Nidogen-1 OS=Homo sapie	6487.1	6662.7	1.03	6247.1	0.96	6194.6	0.95	6379.3	0.98	6386.4	0.98	6458	1.00	6279.2	0.97
Q7Z5L0	Vitelline membrane outer la	14878.8	14838	1.00	11073.3	0.74	10759.1	0.72	11735	0.79	12882.7	0.87	15612.3	1.05	14948.9	1.00
B4DM05	cDNA FLJ51241, highly sin	3873.7	3373.5	0.87	3211.1	0.83	1987.1	0.51	3039.5	0.78	3779.5	0.98	3380.4	0.87	2472.9	0.64
P02671	Fibrinogen alpha chain OS=	4234.1	4743	1.12	3637.4	0.86	2688.6	0.63	4047.4	0.96	4198.3	0.99	4620.4	1.09	5013.4	1.18

A0A024R50	Arylsulfatase A, isoform CR	4624.8	5705.9	1.23	4819.6	1.04	3916.7	0.85	5213.7	1.13	4961.3	1.07	5588.7	1.21	4636.9	1.00
B4DNW0	cDNA FLJ60317, highly sin	4646.2	6735	1.45	4362.2	0.94	4447.6	0.96	4353.5	0.94	5904.8	1.27	6104.6	1.31	4342.9	0.93
P16870	Carboxypeptidase E OS=Ho	6000.8	6963.6	1.16	8192.7	1.37	6190.8	1.03	6365.7	1.06	7394.1	1.23	5195.9	0.87	4811.7	0.80
P08582	Melanotransferrin OS=Hom	3211.1	3034	0.94	2998.4	0.93	2663.6	0.83	3365	1.05	3292.2	1.03	2782.2	0.87	2661	0.83
Q99715	Collagen alpha-1(XII) chain	5685.6	5615.7	0.99	4543.5	0.80	4483	0.79	5307.4	0.93	5506.9	0.97	5496.1	0.97	5446.3	0.96
P08294	Extracellular superoxide dis	7946.4	6185.7	0.78	6487.6	0.82	5858.6	0.74	6718.8	0.85	7256.2	0.91	7641.9	0.96	6378.4	0.80
Q6EMK4	Vasorin OS=Homo sapiens	11193	13637.6	1.22	11660.9	1.04	11262.7	1.01	12179.6	1.09	13908.9	1.24	12904.8	1.15	11381.6	1.02
P21333	Filamin-A OS=Homo sapien	3522	3358.3	0.95	3156.8	0.90	2544.4	0.72	3219.8	0.91	3486	0.99	3310	0.94	3494.3	0.99
A0A0X9UW	GCT-A5 light chain variable	10309.4	8308.8	0.81	11073.3	1.07	6619.9	0.64	9396.4	0.91	8991.8	0.87	10450.5	1.01	11201.3	1.09
P02750	Leucine-rich alpha-2-glycop	19100	12613	0.66	24606.6	1.29	10453.9	0.55	15722.9	0.82	13511	0.71	13859.2	0.73	35646.8	1.87
P19440	Gamma-glutamyltranspeptid	5953.7	6381.6	1.07	5268.6	0.88	4712	0.79	5852.7	0.98	6820.6	1.15	7100.7	1.19	4533.4	0.76
P30530	Tyrosine-protein kinase rec	8678.5	8354.3	0.96	8598.2	0.99	6514.2	0.75	8759.5	1.01	8655.7	1.00	8945.3	1.03	8113.3	0.93
O00187	Mannan-binding lectin serin	29491.9	28174.7	0.96	31325.3	1.06	20703.3	0.70	27106.5	0.92	28729	0.97	32659.2	1.11	37673	1.28
Q9NZP8	Complement C1r subcompo	4947.3	4612	0.93	4221.7	0.85	3471	0.70	4781.8	0.97	5028.8	1.02	4536.8	0.92	5447.1	1.10
Q96FE7	Phosphoinositide-3-kinase-i	60639.2	48791.1	0.80	50194.9	0.83	33426.8	0.55	56833.1	0.94	56993.2	0.94	54600.6	0.90	45056	0.74
Q4TZM4	Hemoglobin beta chain (Fra	134.5	799.4	5.94	200.7	1.49	152.1	1.13	169.2	1.26	391.1	2.91	392.4	2.92	398.7	2.96
P16070	CD44 antigen OS=Homo sa	61721.5	46121.7	0.75	44805.3	0.73	32947.2	0.53	52623.7	0.85	52530	0.85	56809.7	0.92	50556.8	0.82
Q96PD5	N-acetylmuramoyl-L-alanin	4574.7	6845.7	1.50	4899.5	1.07	4304.9	0.94	6124.7	1.34	4679	1.02	4731.7	1.03	5866.3	1.28
Q59EQ1	Cadherin 11, type 2 isoform	6154.1	5392.5	0.88	6145.5	1.00	4164.8	0.68	5892.6	0.96	5570.7	0.91	5926.4	0.96	6746.7	1.10
Q59EJ3	Heat shock 70kDa protein 1	1260.2	1688.7	1.34	1313.5	1.04	1123	0.89	1448.6	1.15	1430.5	1.14	1427.3	1.13	1312.5	1.04
B4DPF0	cDNA FLJ54604, highly sin															
H7BY55	Complement decay-accelera	23131.9	16870.9	0.73	18960.2	0.82	11991	0.52	17699.9	0.77	18951.4	0.82	18436.9	0.80	20402.1	0.88
A0A024R5H	Folate receptor 1 (Adult), is	13354.9	11850.3	0.89	12237.3	0.92	8645.7	0.65	12932.5	0.97	13761.3	1.03	12888.9	0.97	10740.6	0.80
Q96RW7	Hemicentin-1 OS=Homo sa	4413.3	4630.5	1.05	4397.2	1.00	3690.3	0.84	4410.4	1.00	4345.8	0.98	4438.6	1.01	3645.6	0.83
Q4LE33	TNC variant protein (Fragm	5727.9	5995.7	1.05	4917.8	0.86	4043.6	0.71	5283.6	0.92	5542.5	0.97	5062	0.88	5917.9	1.03
P00558	Phosphoglycerate kinase 1 O	2517	3378.6	1.34	2678.5	1.06	2414.9	0.96	2926.4	1.16	3242.2	1.29	3386	1.35	2793.2	1.11
P05543	Thyroxine-binding globulin	10225	8329.4	0.81	6472.1	0.63	7373.6	0.72	8439.7	0.83	11253.1	1.10	10374.4	1.01	12476.3	1.22
D3DQU2	Tripeptidyl peptidase I, isof	3143.2	4449.1	1.42	3953.4	1.26	3176.4	1.01	3856.9	1.23	3914.5	1.25	5232.1	1.66	3194.9	1.02
Q59EP1	Annexin (Fragment) OS=Ho	4902.7	5871.8	1.20	5467.6	1.12	4177.8	0.85	5309.7	1.08	6345.5	1.29	5208	1.06	5078.5	1.04
P33908	Mannosyl-oligosaccharide 1	4125.6	4720.8	1.14	4095.7	0.99	3316.8	0.80	4006.9	0.97	4866.9	1.18	4740.6	1.15	3971.6	0.96
B7ZW00	COL6A3 protein OS=Homo	862.7	764	0.89	902.1	1.05	576.2	0.67	961.7	1.11	1286.6	1.49	832.8	0.97	1001.7	1.16
P60022	Beta-defensin 1 OS=Homo	5562.9	5318.3	0.96	7551.5	1.36	5017.4	0.90	6151.3	1.11	4069.6	0.73	8211.8	1.48	7714.7	1.39
Q9UL78	Myosin-reactive immunoglo	5804.5	5435.1	0.94	5646.1	0.97	4489.4	0.77	5065.1	0.87	5431.4	0.94	6108.6	1.05	5699.8	0.98
S6BGE0	IgG H chain OS=Homo sap	665	619.1	0.93	614.4	0.92	644.3	0.97	620.3	0.93	634.2	0.95	631.1	0.95	726.6	1.09
Q9HCU0	Endosialin OS=Homo sapie	13413	12401.7	0.92	10911.9	0.81	9069.8	0.68	13278.6	0.99	13421.9	1.00	12460.9	0.93	10600.7	0.79
A0A1K0GX	Globin C1 OS=Homo sapien	6799.3	29618.2	4.36	9508.2	1.40	7413.2	1.09	8253.1	1.21	15153	2.23	13904.8	2.05	15770.1	2.32
P43121	Cell surface glycoprotein M	3485.7	3270	0.94	3254.3	0.93	2493.3	0.72	3249.6	0.93	3281.8	0.94	3803.3	1.09	3274.2	0.94
A0A075B6N	Ig alpha-2 chain C region (F	590.3	692.8	1.17	593.2	1.00	599.1	1.01	790.1	1.34	732.1	1.24	521.6	0.88	551.7	0.93
Q96NY8	Nectin-4 OS=Homo sapiens	4612.5	4708.4	1.02	3999.8	0.87	3850	0.83	4385.7	0.95	4907.4	1.06	5536.9	1.20	4613.6	1.00
A0A024RDI	SPARC-like 1 (Mast9, hevin	3687.9	3793.8	1.03	4046.1	1.10	3261.7	0.88	3774.7	1.02	4534	1.23	3652.7	0.99	3736.7	1.01
G3V4U0	Fibulin-5 OS=Homo sapiens	4667.9	5074.9	1.09	4457.3	0.95	3773.5	0.81	4585.5	0.98	5020.6	1.08	4681	1.00	4490.6	0.96
A0A0A1TT	Lutheran blood group glyco	3736.7	4202.2	1.12	3335.2	0.89	3038.3	0.81	3613.9	0.97	3773.6	1.01	3867.2	1.03	3478	0.93
X6R8A1	Carboxypeptidase OS=Hom	5613.9	9108.3	1.62	4903.2	0.87	5425.3	0.97	7045.1	1.25	7273.7	1.30	8585.6	1.53	6819.7	1.21
P19320	Vascular cell adhesion prote	4993.5	4789.2	0.96	7333.7	1.47	4646.3	0.93	4838.7	0.97	4779.5	0.96	4553.1	0.91	5918.9	1.19
A0A024R0A	Macrophage colony-stimula	7324.9	6218.2	0.85	6126.4	0.84	4574	0.62	6227	0.85	6485.1	0.89	6718.1	0.92	7291.7	1.00
P12111	Collagen alpha-3(VI) chain	7162.4	7415.6	1.04	7912.6	1.10	5452.6	0.76	7785.7	1.09	8009.2	1.12	7299.1	1.02	7169.4	1.00
Q6UX06	Olfactomedin-4 OS=Homo	6418.7	5003.2	0.78	4025.9	0.63	3660.8	0.57	4811.1	0.75	9592.3	1.49	4072.6	0.63	4205.4	0.66
A8K2T4	cDNA FLJ78207, highly sin	7732.9	7452.5	0.96	7214.7	0.93	5493.9	0.71	7679.1	0.99	7694	0.99	8065.7	1.04	7781.9	1.01
A0A024RC4	Cadherin 2, type 1, N-cadhe	4857.4	4051.2	0.83	3790	0.78	2964.5	0.61	4320.6	0.89	4196.5	0.86	4337.4	0.89	4671	0.96
P51884	Lumican OS=Homo sapiens	7967.7	7642.1	0.96	10154.5	1.27	6257	0.79	8331.2	1.05	9397.3	1.18	8790.4	1.10	8770.9	1.10
Q9BYE9	Cadherin-related family mem	3705.1	3781.4	1.02	3640	0.98	3191.5	0.86	3753.9	1.01	3913.1	1.06	3706.3	1.00	3380.4	0.91
Q9UKU9	Angiopoietin-related protein	6197.8	7634.6	1.23	4390.3	0.71	6199.6	1.00	7477.6	1.21	6215.8	1.00	10462.1	1.69	5680	0.92
P04406	Glyceraldehyde-3-phosphate	4050.2	4985.1	1.23	4364.4	1.08	3597.4	0.89	4289.4	1.06	5148.7	1.27	5397.6	1.33	4728.8	1.17
A4D2D2	Procollagen C-endopeptidas	2776.7	2648.9	0.95	2896.3	1.04	2079.8	0.75	2789.7	1.00	2935	1.06	2604.4	0.94	2635.7	0.95
Q9BXP8	Pappalysin-2 OS=Homo sap	2528.1	3162.2	1.25	2628.6	1.04	2362.7	0.93	2984.5	1.18	3081.3	1.22	3160.9	1.25	2770.2	1.10
P01591	Immunoglobulin J chain OS	22042.9	21643	0.98	17461.8	0.79	13951.6	0.63	20737.8	0.94	23622.7	1.07	19619.8	0.89	18930	0.86
A0A024R37	Cathepsin B, isoform CRA_	9181.8	11295	1.23	8769	0.96	7231.1	0.79	13199.4	1.44	12385.8	1.35	11797	1.28	8858.7	0.96
B4DVA7	Beta-hexosaminidase OS=H	4755.1	5384.5	1.13	3289.8	0.69	3831.4	0.81	4917.7	1.03	6053.1	1.27	5639.6	1.19	4600.1	0.97

Q03403	Trefoil factor 2 OS=Homo s	7971.6	8058.9	1.01	7228.5	0.91	7331.7	0.92	7912	0.99	8480.1	1.06	10380.9	1.30	7841.1	0.98
A0A140VJC	Testicular tissue protein Li 2	6176.6	6109.1	0.99	5411.3	0.88	4451.7	0.72	5954.7	0.96	6067.9	0.98	5151.7	0.83	5195	0.84
Q969P0	Immunoglobulin superfamily	5559.5	5478.2	0.99	5763.1	1.04	4520.6	0.81	5536.4	1.00	6001.2	1.08	6068.8	1.09	5037.5	0.91
Q96SA9	Anti-streptococcal/anti-myo	56.4	59	1.05	62.1	1.10	34.4	0.61	54.8	0.97	59.8	1.06	60.9	1.08	70	1.24
A0N071	Delta globin OS=Homo sap															
A0A024R9E	Plasma glutamate carboxype	4634	5450.1	1.18	4178.2	0.90	3922.2	0.85	4977.5	1.07	5938.4	1.28	6070.9	1.31	4969.1	1.07
P43251	Biotinidase OS=Homo sapie	3739	4007.9	1.07	3433.6	0.92	3371.9	0.90	3544.7	0.95	3927.9	1.05	3892.8	1.04	3499.8	0.94
D3DPK5	SH3 domain binding glutam	8402.9	7301.7	0.87	9659	1.15	5353	0.64	8906.8	1.06	8228.3	0.98	9216.4	1.10	7713.3	0.92
Q14767	Latent-transforming growth	2717.6	2487.2	0.92	2812.7	1.03	1639.6	0.60	2847.3	1.05	2505.4	0.92	2936.5	1.08	2437.9	0.90
Q92956	Tumor necrosis factor recep	2257.6	2459.8	1.09	1976.6	0.88	1927.6	0.85	2293.4	1.02	2205.1	0.98	1971.1	0.87	2168	0.96
A0A087WW	Mucin-1 OS=Homo sapiens	8057.4	7146.2	0.89	6532	0.81	5873.5	0.73	7307.5	0.91	8981	1.11	7333.4	0.91	6572.8	0.82
P07195	L-lactate dehydrogenase B c	7645.5	10143.2	1.33	7229.1	0.95	6568.5	0.86	8089.9	1.06	11867.5	1.55	9207.9	1.20	7653	1.00
A2MYE1	A30 (Fragment) OS=Homo	4963.5	4420.3	0.89	5418.3	1.09	5346.5	1.08	4195.9	0.85	4712.5	0.95	4510.6	0.91	5181.3	1.04
Q4W4Y1	Dopamine receptor interacti	2352.5	2555.9	1.09	2251.8	0.96	2095.3	0.89	2399	1.02	2771.2	1.18	2422.2	1.03	1895.2	0.81
D3DSQ1	N-acylsphingosine amidohy	4530.2	5411	1.19	4681.5	1.03	3678.5	0.81	5312.2	1.17	5482.3	1.21	4348	0.96	4323.6	0.95
A1A4E9	Keratin 13 OS=Homo sapie	3225.2	6278.1	1.95	2943.1	0.91	3674.5	1.14	2963.2	0.92	3484.1	1.08	5475.3	1.70	6283.7	1.95
P07996	Thrombospondin-1 OS=Hor	862.4	829.5	0.96	950.9	1.10	603	0.70	949.9	1.10	973.4	1.13	1055	1.22	955.2	1.11
Q07507	Dermatopontin OS=Homo s	7932.2	7663.8	0.97	8305.1	1.05	5661.8	0.71	7674.5	0.97	8157	1.03	8296	1.05	6186.1	0.78
O43278	Kunitz-type protease inhibit	2534.5	2618.5	1.03	2388.3	0.94	1943.2	0.77	2441.5	0.96	2672.2	1.05	2229.9	0.88	2272.6	0.90
B2RCM5	cDNA, FLJ96160, highly si	2574.2	2778.8	1.08	2565.7	1.00	2057.3	0.80	2724.9	1.06	2740.7	1.06	2594.7	1.01	2391.2	0.93
B2R815	cDNA, FLJ93695, highly si	3411.3	3952.5	1.16	3292.2	0.97	2683.9	0.79	3455.1	1.01	3860.7	1.13	4139.3	1.21	3346.8	0.98
Q9UL83	Myosin-reactive immunoglo	1795.6	1858.1	1.03	1701.5	0.95	1872.9	1.04	1877.1	1.05	1697.8	0.95	1765.9	0.98	1933.1	1.08
B2R9F2	cDNA, FLJ94361, highly si	6301.6	6586.5	1.05	6752	1.07	6012.8	0.95	6470.5	1.03	6705.7	1.06	6742.9	1.07	7251.2	1.15
Q65ZC9	Single-chain Fv (Fragment)	3455.5	3911.8	1.13	3290.7	0.95	3307.4	0.96	3863.8	1.12	3751.6	1.09	3579.6	1.04	3859.5	1.12
B2R6J2	cDNA, FLJ92973, highly si	4708.4	4918.4	1.04	4153.1	0.88	3316.6	0.70	4704.9	1.00	5856.9	1.24	5435.1	1.15	4215.5	0.90
Q6UVK1	Chondroitin sulfate proteogl	4052.6	4253.8	1.05	3586.2	0.88	3342.5	0.82	3778.9	0.93	4146.7	1.02	4047.6	1.00	3405.6	0.84
A0A024R9C	Thrombospondin 1, isoform	2992.3	2782.9	0.93	2760.4	0.92	1885.2	0.63	2612.4	0.87	2562.7	0.86	2686	0.90	2439.8	0.82
P06312	Immunoglobulin kappa varia	765.8	695.6	0.91	596.1	0.78	541.4	0.71	617.4	0.81	633.4	0.83	772.7	1.01	811.4	1.06
Q6UXG3	CMRF35-like molecule 9 O	12761.2	10138.3	0.79	8746.7	0.69	7962.6	0.62	9517.2	0.75	10915.3	0.86	10986.5	0.86	9753.3	0.76
Q9UL81	Myosin-reactive immunoglo	23.2	34.3	1.48	62.8	2.71	26.6	1.15	38.9	1.68	30.3	1.31	38	1.64	34.1	1.47
D3DVW9	Protein tyrosine phosphatase	3750.6	3944.4	1.05	3701.6	0.99	3299.8	0.88	3873.7	1.03	4607.8	1.23	4428.1	1.18	4253.1	1.13
V9HWB8	Pyruvate kinase OS=Homo	3511.6	4612.1	1.31	3926.1	1.12	3562.7	1.01	4358.7	1.24	4392.5	1.25	4571	1.30	5137.3	1.46
B2R4M6	Protein S100 OS=Homo sap	4812.2	6589.9	1.37	5338.6	1.11	3495.5	0.73	6009.2	1.25	6572.4	1.37	6127.6	1.27	9257.9	1.92
A0A024R2V	Dystroglycan 1 (Dystrophin	2219.6	2598.5	1.17	2103.5	0.95	1831.5	0.83	2110.9	0.95	2400.7	1.08	2450.8	1.10	1990	0.90
G3V3Y2	Fibulin-5 (Fragment) OS=H	255.6	188.9	0.74	217.1	0.85	121.8	0.48	192.6	0.75	184.4	0.72	179	0.70	237.3	0.93
Q9UL70	Myosin-reactive immunoglo	84.6	101	1.19	108.3	1.28	81.7	0.97	91.1	1.08	95	1.12	97.9	1.16	89.3	1.06
O00533	Neural cell adhesion molecu	1884.3	1722.1	0.91	1576.4	0.84	1445.9	0.77	1956.5	1.04	1877.3	1.00	1718.9	0.91	1643.1	0.87
X6R8F3	Neutrophil gelatinase-associ	6613.9	6977.9	1.06	5396.3	0.82	3983.1	0.60	5926.6	0.90	10723.4	1.62	5612.7	0.85	7023.2	1.06
A0A0K0K1	6-phosphogluconolactonase	3608.4	4299.8	1.19	4210.3	1.17	3104.4	0.86	4061.4	1.13	4581.4	1.27	4552.2	1.26	4135	1.15
A2NB45	Cold agglutinin FS-1 L-chai	1753.5	1719.5	0.98	2595.5	1.48	1320.5	0.75	1746.8	1.00	1749.3	1.00	1796.2	1.02	1897.8	1.08
E9PNQ8	Thy-1 membrane glycoprote	2848.4	2619	0.92	3111.1	1.09	2023.6	0.71	2671.5	0.94	2798.9	0.98	2710.3	0.95	2412.1	0.85
P09668	Pro-cathepsin H OS=Homo	3752.6	3818.3	1.02	3708.1	0.99	3114.7	0.83	4093.6	1.09	4162.3	1.11	4117.3	1.10	3927.9	1.05
A0A109PW	MS-C1 light chain variable	534.8	632.9	1.18	643.8	1.20	402.3	0.75	598.6	1.12	540.1	1.01	486.2	0.91	572.3	1.07
Q06830	Peroxiredoxin-1 OS=Homo	4003	5109.6	1.28	3996.1	1.00	3116.1	0.78	3967	0.99	5034.6	1.26	4278.5	1.07	3861.5	0.96
A0A140VJV	Sushi domain containing 2,	2019.6	2316.1	1.15	1852.6	0.92	1772.3	0.88	1980	0.98	2065.6	1.02	2159.8	1.07	1559.9	0.77
Q6UXB8	Peptidase inhibitor 16 OS=H	5069.6	4194.7	0.83	6895.6	1.36	3273.1	0.65	4707.7	0.93	3892.8	0.77	4161.6	0.82	5028.4	0.99
P26038	Moesin OS=Homo sapiens (	1515.3	1900.3	1.25	1419.9	0.94	1609.4	1.06	1648.8	1.09	1863.5	1.23	1982.3	1.31	1550.3	1.02
B4DUV1	Fibulin-1 OS=Homo sapiens	4051.8	3808.6	0.94	4108.4	1.01	3064.4	0.76	4002.7	0.99	3563	0.88	3644	0.90	3855.4	0.95
P14384	Carboxypeptidase M OS=H	7514.5	7591.3	1.01	6086.5	0.81	5482.5	0.73	7051.3	0.94	7146.8	0.95	6982.5	0.93	6406.9	0.85
A0A0X9TD	MS-D1 light chain variable	6873.1	6922.7	1.01	8683.4	1.26	6141.7	0.89	7193.8	1.05	6887.4	1.00	8061	1.17	7558.7	1.10
A0A024R9J	Nephroblastoma overexpres	3228.5	3194.3	0.99	3252.1	1.01	2459.5	0.76	3488.8	1.08	3041.5	0.94	2950.5	0.91	2732.1	0.85
P35908	Keratin, type II cytoskeletal	884.5	823.2	0.93	575.3	0.65	800.4	0.90	610.1	0.69	771.6	0.87	850.1	0.96	708.4	0.80
V9HWB4	Epididymis secretory sperm	1025.7	1085.3	1.06	908.9	0.89	882.4	0.86	970.4	0.95	1146.5	1.12	1092	1.06	995.2	0.97
P43652	Afamin OS=Homo sapiens (	5833.9	6555.5	1.12	5167.9	0.89	9319.1	1.60	6123.9	1.05	5821.8	1.00	6216.2	1.07	6424.9	1.10
A2NI60	BRE (Fragment) OS=Homo															
A0A024R10	Contactin 1, isoform CRA_a	2524.1	2593.6	1.03	2385	0.94	2125	0.84	2493.1	0.99	2662.2	1.05	2498.9	0.99	2137.7	0.85
Q9Y5Y7	Lymphatic vessel endothelia	22274.4	13497.1	0.61	21240	0.95	9204.5	0.41	20244.2	0.91	18007.5	0.81	18504.4	0.83	26382.3	1.18
A0A024R5Z	Annexin OS=Homo sapiens	2178.1	2133.6	0.98	2179.2	1.00	2212.7	1.02	2570.7	1.18	2318.8	1.06	2357.3	1.08	2382.2	1.09

Q7Z7M0	Multiple epidermal growth f	2118.1	2286.2	1.08	2037.4	0.96	1769.5	0.84	2318.4	1.09	2546.4	1.20	2450.9	1.16	1908.6	0.90
A0A0X9UW	MS-B1 light chain variable r	11.2	22	1.96	19.9	1.78	16.8	1.50	14.9	1.33	11.5	1.03	13.4	1.20	14.5	1.29
Q92520	Protein FAM3C OS=Homo	3355.8	3680.4	1.10	4972.7	1.48	3158.2	0.94	3505.7	1.04	4087.7	1.22	3307.6	0.99	3179.6	0.95
P17936	Insulin-like growth factor-bi	3871	3835.1	0.99	3423.4	0.88	2883.5	0.74	3101.8	0.80	4083.1	1.05	4204.2	1.09	3949.8	1.02
S6C4Q9	IgG L chain OS=Homo sapi	532.7	521.1	0.98	715.4	1.34	471.4	0.88	665.7	1.25	602.4	1.13	568.8	1.07	596.1	1.12
Q13228	Selenium-binding protein 1 (	3078.1	3077.2	1.00	2877.9	0.93	2469.5	0.80	3024	0.98	3094.8	1.01	3213	1.04	2905.3	0.94
Q9UL90	Myosin-reactive immunoglo	667.8	744.5	1.11	577.6	0.86	711.5	1.07	632.5	0.95	880.3	1.32	652.9	0.98	689.8	1.03
B2RE74	cDNA, FLJ94876, highly si	1428.4	2126.8	1.49	1580.9	1.11	1895.1	1.33	1611.2	1.13	1945.1	1.36	1668.7	1.17	1386.4	0.97
Q9H804	cDNA FLJ14022 fis, clone I	216.2	307.3	1.42	230.2	1.06	287.6	1.33	290.7	1.34	183.2	0.85	317.8	1.47	358.9	1.66
V9HW53	Dimethylarginine dimethyla	2336.9	2501.4	1.07	2391.8	1.02	1909.8	0.82	2285.5	0.98	2782.2	1.19	2671.6	1.14	2377.4	1.02
Q9UHL4	Dipeptidyl peptidase 2 OS=I	2357.4	2840.9	1.21	2170.5	0.92	2404.5	1.02	2754.5	1.17	2972.2	1.26	3067	1.30	2389.2	1.01
O00241	Signal-regulatory protein be	3846.2	3648.6	0.95	3977	1.03	2785.2	0.72	3271.7	0.85	3353.4	0.87	3901	1.01	3816.9	0.99
Q9HAT2	Sialate O-acetylesterase OS=	5170.6	5690.4	1.10	5070.3	0.98	3857.9	0.75	5266.1	1.02	6201.3	1.20	5169.4	1.00	5246.1	1.01
B7Z9B8	cDNA FLJ56912, highly sin	5298.8	5761.1	1.09	6171.9	1.16	3949.3	0.75	5993.9	1.13	5556.2	1.05	5967.5	1.13	4806.3	0.91
Q96DA0	Zymogen granule protein 16	6621.2	5922.7	0.89	4720.3	0.71	3676.1	0.56	5244.9	0.79	6937.1	1.05	4787.5	0.72	4561.2	0.69
A8K7Q1	cDNA FLJ77770, highly sin	3019.9	3709.7	1.23	2704.6	0.90	3207.6	1.06	2838.6	0.94	3151.2	1.04	3331	1.10	2708.4	0.90
V9HWE9	Epididymis secretory protei	2354.8	2724.2	1.16	2283.9	0.97	1889.5	0.80	2734.4	1.16	2881.9	1.22	2660.9	1.13	2578.8	1.10
P00995	Serine protease inhibitor Ka	4616.6	3518.1	0.76	4646.8	1.01	3072.8	0.67	3937.2	0.85	4284.3	0.93	4482	0.97	4145.1	0.90
P23142	Fibulin-1 OS=Homo sapiens															
J3KPF3	4F2 cell-surface antigen hea	1964.2	2481.6	1.26	1848.5	0.94	1686	0.86	2022.9	1.03	2327	1.18	2371.8	1.21	1878.3	0.96
P01023	Alpha-2-macroglobulin OS=	1389.4	2846.8	2.05	1790.8	1.29	1615.2	1.16	2120.8	1.53	1764.6	1.27	2447.4	1.76	1904.3	1.37
A0A146E5L	Mesenchymal stromal cell-a	3675.7	3860.1	1.05	3901.6	1.06	2476.7	0.67	4008.5	1.09	4118.3	1.12	4203.8	1.14	3991.6	1.09
Q6LES2	Annexin (Fragment) OS=Ho	1926.9	2260.1	1.17	1675.5	0.87	1802.2	0.94	2131.6	1.11	2170.9	1.13	2216.3	1.15	1804.7	0.94
V9HW12	Epididymis secretory sperm	2198.6	2931.3	1.33	2180.4	0.99	1703.8	0.77	2218.3	1.01	2695.6	1.23	2416.8	1.10	2170.5	0.99
A0A087WV	Neural cell adhesion molecu	7327.9	6867.7	0.94	6201	0.85	4723.8	0.64	6512.9	0.89	7057.6	0.96	7053.3	0.96	6276.5	0.86
P05109	Protein S100-A8 OS=Homo	7687.1	11368.9	1.48	8367.2	1.09	5845.8	0.76	10957.3	1.43	11092.3	1.44	8418.2	1.10	16271.7	2.12
A0A0S2Z42	HCG2039812, isoform CRA	2019.2	2751.2	1.36	1418.1	0.70	1540.3	0.76	1412.6	0.70	1874.5	0.93	2630.1	1.30	3741.2	1.85
A0A0G2JLV	Leukocyte-associated immu	10954.7	9309.4	0.85	8803.8	0.80	7112.7	0.65	10296	0.94	10161.5	0.93	10274.9	0.94	9720.2	0.89
M9MML0	Fc of IgG low affinity IIIa re	2128.2	2065.5	0.97	1762.7	0.83	1408.9	0.66	2087.8	0.98	1902.6	0.89	2406.7	1.13	1841.5	0.87
P17900	Ganglioside GM2 activator (	30307.1	26291.2	0.87	37220.9	1.23	17844.8	0.59	33707.9	1.11	36274.8	1.20	30110.7	0.99	45241.4	1.49
Q14766	Latent-transforming growth	1726.8	1740.2	1.01	2345.7	1.36	1354.9	0.78	1814.4	1.05	1769.2	1.02	1682.6	0.97	1620.1	0.94
V9HWC9	Superoxide dismutase [Cu-Z	7051.2	6624.5	0.94	7573.1	1.07	5875.6	0.83	7308.5	1.04	7867.9	1.12	6568.3	0.93	6099.7	0.87
A8K0E7	cDNA FLJ77464, highly sin	1642.1	1621.8	0.99	1477	0.90	1200	0.73	1499.5	0.91	1658.5	1.01	1702.6	1.04	1531.7	0.93
Q8N114	Protein shisa-5 OS=Homo s	9621.4	7917.8	0.82	7596	0.79	5190.8	0.54	8312.4	0.86	8938	0.93	8866.3	0.92	8826.3	0.92
A8K5T0	cDNA FLJ75416, highly sin	1759.1	2427	1.38	2126.8	1.21	1682.7	0.96	1907.5	1.08	1816.1	1.03	2171.7	1.23	2007.4	1.14
P61970	Nuclear transport factor 2 O	4546.1	4776.5	1.05	3725	0.82	3291.9	0.72	4216.4	0.93	5200.8	1.14	5235.9	1.15	4608.7	1.01
Q53HF3	Galactosidase, alpha variant	1888	2257.9	1.20	1522.3	0.81	1554.9	0.82	1689.8	0.90	2162.2	1.15	2305	1.22	1481.8	0.78
A8K7E0	cDNA FLJ76911, highly sin	1780.1	2175.2	1.22	1777.9	1.00	1639.2	0.92	2115.3	1.19	2503.3	1.41	2498	1.40	1949.8	1.10
A0A0X9T7	MS-D3 heavy chain variable	988	1066.4	1.08	825.6	0.84	970.3	0.98	970.9	0.98	1053.7	1.07	912.5	0.92	1027.9	1.04
P26842	CD27 antigen OS=Homo sa	8475.3	7241.3	0.85	8781.8	1.04	5361	0.63	8328	0.98	7492	0.88	8459.2	1.00	8591.4	1.01
Q9UHG2	ProSAAS OS=Homo sapien	1948.4	2006.3	1.03	1615.3	0.83	1372.2	0.70	1626.7	0.83	1858.4	0.95	1994.7	1.02	1628.7	0.84
P39059	Collagen alpha-1(XV) chain	5038.6	5465.6	1.08	4044.1	0.80	4524.8	0.90	5523.7	1.10	5827.2	1.16	5977.5	1.19	4145.9	0.82
D3DX01	Amine oxidase OS=Homo s	2004.7	2049.2	1.02	2070.4	1.03	1802	0.90	1655.5	0.83	2233.3	1.11	1984.6	0.99	1995.2	1.00
S6BGF5	IgG H chain OS=Homo sapi	1031.6	1192.1	1.16	845	0.82	1179.8	1.14	1052	1.02	1038.8	1.01	1036.4	1.00	1099.6	1.07
Q0ZCJ2	Immunglobulin heavy chain	24.3	38.8	1.60	37.4	1.54	28.2	1.16	41.6	1.71	35.7	1.47	27.7	1.14	26.7	1.10
Q59EP2	Angiotensinogen variant (Fr	2105.4	2605.1	1.24	2571.8	1.22	2309.7	1.10	2338.5	1.11	2834.5	1.35	2294.5	1.09	2443	1.16
A6XNE2	Complement factor D prepro	564.6	781.8	1.38	804.9	1.43	590.3	1.05	636.8	1.13	704	1.25	786.6	1.39	728.6	1.29
A0A068LKI	Ig heavy chain variable regio	666.2	671.9	1.01	506.6	0.76	675.4	1.01	667.3	1.00	777.7	1.17	516.7	0.78	714.8	1.07
Q9HD89	Resistin OS=Homo sapiens	1785.5	2217.6	1.24	1779.3	1.00	1616.5	0.91	2079	1.16	2087.2	1.17	2264.9	1.27	2240.8	1.25
P07900	Heat shock protein HSP 90-	1472.5	1982.8	1.35	1471.1	1.00	1355.8	0.92	1550	1.05	1803.6	1.22	1925.8	1.31	1429.5	0.97
Q9HCN6	Platelet glycoprotein VI OS=	5334.1	5401.9	1.01	4653	0.87	3841	0.72	4876.3	0.91	5472.5	1.03	4774.4	0.90	4989.3	0.94
P05937	Calbindin OS=Homo sapien	2335.8	3120.3	1.34	2021.4	0.87	2552.3	1.09	2504.5	1.07	2977.9	1.27	2806	1.20	3014.6	1.29
Q13332	Receptor-type tyrosine-prote	855.9	915.3	1.07	826.1	0.97	720.2	0.84	816.5	0.95	883.4	1.03	844	0.99	710.9	0.83
P05164	Myeloperoxidase OS=Homo	1404.2	2474.9	1.76	1795.9	1.28	1263.4	0.90	1544.4	1.10	1808.1	1.29	2952.7	2.10	2311.4	1.65
P08138	Tumor necrosis factor recep	6441.7	6089.6	0.95	5018.9	0.78	4209.4	0.65	6183.3	0.96	5837.5	0.91	5233.7	0.81	5392	0.84
Q5TFQ8	Signal-regulatory protein be															
X5D7A8	Cell adhesion molecule 1 iso	4714.4	4355.4	0.92	3410.7	0.72	3144.4	0.67	4044	0.86	4109.3	0.87	4185.9	0.89	4041.7	0.86
P00352	Retinal dehydrogenase 1 OS	1152.8	1665.4	1.44	1147	0.99	1404.6	1.22	1296	1.12	1586.3	1.38	1485.7	1.29	1109.5	0.96

Q16769	Glutaminyl-peptide cyclotra	6127	5273.8	0.86	4544.2	0.74	3676	0.60	4501.6	0.73	5319.2	0.87	5943	0.97	4307.9	0.70
A0A024R5L	Prolylcarboxypeptidase (An	2154.2	2348.5	1.09	1816.3	0.84	1623.9	0.75	2294.1	1.06	2412.9	1.12	2636.9	1.22	2051.7	0.95
Q8TDQ0	Hepatitis A virus cellular rec	12319.8	11849.5	0.96	10111.4	0.82	8585.3	0.70	12179.6	0.99	12614.3	1.02	11397.2	0.93	12392.8	1.01
Q9UIB8	SLAM family member 5 OS	2814.1	2794.8	0.99	2510.8	0.89	2060	0.73	2804	1.00	2646.8	0.94	2772.8	0.99	2812.1	1.00
A0A087WZ	Ribonuclease T2 OS=Homo	1668.3	1726.6	1.03	1731.7	1.04	1292.8	0.77	1821.6	1.09	1698.9	1.02	1554	0.93	1615.7	0.97
A0A024RA	Betaine-homocysteine methy	221.3	337.8	1.53	237.5	1.07	290.9	1.31	222.5	1.01	268.4	1.21	288.7	1.30	217.4	0.98
O94910	Adhesion G protein-coupled	1235.7	1408.8	1.14	1034.8	0.84	1152.5	0.93	1145.8	0.93	1207	0.98	1139.3	0.92	1024.2	0.83
Q9Y2E5	Epididymis-specific alpha-m	1984.9	2511.5	1.27	1880.1	0.95	1749.3	0.88	2266.8	1.14	2665.7	1.34	2456.1	1.24	1958.5	0.99
Q6ICS1	Putative uncharacterized pro	30.7	29.8	0.97	68.7	2.24	29.7	0.97	27.5	0.90	40.9	1.33	61.3	2.00	41.6	1.36
P07737	Profilin-1 OS=Homo sapien	2994	4046.8	1.35	4969.7	1.66	2210.9	0.74	3424.1	1.14	3815.9	1.27	3843.3	1.28	3985.6	1.33
O96009	Napsin-A OS=Homo sapien	5563.1	7511.5	1.35	5588.8	1.00	4732.2	0.85	6183.3	1.11	8213.1	1.48	8080.5	1.45	6626.3	1.19
O43707	Alpha-actinin-4 OS=Homo s	766.8	1027.5	1.34	816.9	1.07	685.2	0.89	836.7	1.09	908.5	1.18	865.1	1.13	836.4	1.09
B3KQF4	cDNA FLJ90373 fis, clone I	1549.3	1424.4	0.92	1623.5	1.05	1361.2	0.88	1355.5	0.87	1601	1.03	910.3	0.59	1127.4	0.73
E7EX29	14-3-3 protein zeta/delta (Fr	2159.2	3019.6	1.40	2253.7	1.04	1805.9	0.84	2220.8	1.03	2654.9	1.23	2262.3	1.05	2440.6	1.13
A0A024RA	Carboxypeptidase OS=Hom	1637.3	2082.3	1.27	1494.3	0.91	1678.4	1.03	1755.8	1.07	1866.3	1.14	2239.4	1.37	1659.7	1.01
Q03591	Complement factor H-relate	1680.8	1255.8	0.75	1348.3	0.80	1029.9	0.61	1501	0.89	1546.7	0.92	1320.3	0.79	1395	0.83
Q0ZCI8	Immunglobulin heavy chain	3187.9	3687	1.16	2573.4	0.81	3131.5	0.98	3709.9	1.16	3624.2	1.14	3036.7	0.95	4086.2	1.28
Q86T13	C-type lectin domain family	3765.5	3762.7	1.00	3412.6	0.91	2841.4	0.75	3417.4	0.91	3589.2	0.95	3429	0.91	3131.1	0.83
B2R5M8	Isocitrate dehydrogenase [N	1994.9	2637.9	1.32	2191.9	1.10	1916.5	0.96	2227.2	1.12	2655.5	1.33	2079	1.04	1758.9	0.88
F5H265	Polyubiquitin-C (Fragment)	9502.6	8762.6	0.92	9627.2	1.01	6689.7	0.70	9272.1	0.98	9372.1	0.99	9688.2	1.02	8035.3	0.85
P12273	Prolactin-inducible protein (	5909.5	6014.5	1.02	5684.5	0.96	3471.5	0.59	12194.5	2.06	6161	1.04	4771.9	0.81	4172.1	0.71
A8K4K1	cDNA FLJ78216, highly sin	2325.6	2706.7	1.16	2104.8	0.91	2049.9	0.88	2339	1.01	2351.3	1.01	2887.8	1.24	2295.4	0.99
V9HW43	Epididymis secretory protei	1213.8	1537.5	1.27	1307	1.08	1189.3	0.98	1315.5	1.08	1427	1.18	1456.7	1.20	1291.5	1.06
A8K5S3	cDNA FLJ78449 OS=Homo	5655.8	5732.7	1.01	4725.4	0.84	4608.2	0.81	5445.7	0.96	6607.4	1.17	5516.1	0.98	4576.7	0.81
P07686	Beta-hexosaminidase subun	3543.4	4263.5	1.20	2996	0.85	3163.9	0.89	3938.5	1.11	4365	1.23	4680.2	1.32	3690.5	1.04
P23470	Receptor-type tyrosine-prote	1622.6	1654.2	1.02	1650.3	1.02	1264.4	0.78	1576.3	0.97	1615.9	1.00	1460.7	0.90	1574	0.97
P35527	Keratin, type I cytoskeletal 9	4522.6	5294.4	1.17	3524	0.78	4589.8	1.01	4269.9	0.94	3950.4	0.87	4250.1	0.94	3597.8	0.80
P55291	Cadherin-15 OS=Homo sap	325.8	288	0.88	234.6	0.72	279.5	0.86	229.6	0.70	254.8	0.78	309.3	0.95	265.3	0.81
Q92896	Golgi apparatus protein 1 O	1840.1	2052.1	1.12	1838.1	1.00	1529.4	0.83	1753.1	0.95	1923.1	1.05	1985.1	1.08	1659.2	0.90
P19013	Keratin, type II cytoskeletal	765.5	1337.8	1.75	812.9	1.06	978.6	1.28	804.4	1.05	941.4	1.23	1621.4	2.12	1858.7	2.43
A0A0X9T7	GCT-A4 light chain variable	9483.6	9768.6	1.03	12718.8	1.34	6822	0.72	10547.4	1.11	9206.9	0.97	11836.9	1.25	10789.2	1.14
Q9GZX9	Twisted gastrulation protein	2924.7	2504.2	0.86	2159.2	0.74	1627.6	0.56	2503.7	0.86	2641.7	0.90	2572.4	0.88	2643.7	0.90
A0A024RA	Oxidised low density lipopro	1957.6	2452.2	1.25	1716.2	0.88	1613.3	0.82	2061.5	1.05	2389.4	1.22	1942.1	0.99	1780	0.91
P02452	Collagen alpha-1(I) chain O	3460.5	3928.9	1.14	3402.1	0.98	2538	0.73	4811.3	1.39	3750.4	1.08	4245.3	1.23	4631.9	1.34
P35052	Glypican-1 OS=Homo sapie	1240.3	1244.5	1.00	1141.8	0.92	1047.5	0.84	1204.3	0.97	1242.2	1.00	1150	0.93	1060	0.85
Q9UBC9	Small proline-rich protein 3	9233.8	5390.1	0.58	12762	1.38	9129	0.99	10573.1	1.15	8360.2	0.91	6416	0.69	11899.2	1.29
A0N5G1	Rheumatoid factor C6 light	2204.4	2360.1	1.07	1989.1	0.90	1960.5	0.89	1873.6	0.85	2111.2	0.96	2171	0.98	2250.6	1.02
O43490	Prominin-1 OS=Homo sapie	1212.4	1222.6	1.01	997	0.82	966.4	0.80	1167.5	0.96	1296	1.07	1105.4	0.91	952.1	0.79
A2NZ55	Variable immnoglobulin ant	102.9	147.8	1.44	123.4	1.20	193.1	1.88	127.9	1.24	134.6	1.31	110.3	1.07	102.6	1.00
Q8IWU5	Extracellular sulfatase Sulf-	10985.6	10334.4	0.94	8417.1	0.77	7503.2	0.68	10138.2	0.92	10534.8	0.96	12935.1	1.18	9686.6	0.88
P02533	Keratin, type I cytoskeletal 1															
K4DIA0	ICOS ligand OS=Homo sap	5031.9	5079.2	1.01	4272.8	0.85	3998.4	0.79	4664.2	0.93	4927	0.98	5008.9	1.00	4414	0.88
G9K388	YWHAE/FAM22A fusion p	553.6	679.3	1.23	470.5	0.85	511.8	0.92	544.3	0.98	687.1	1.24	594.4	1.07	534.4	0.97
P01036	Cystatin-S OS=Homo sapien	1649.6	1438.1	0.87	1542.3	0.93	1180.3	0.72	1476.5	0.90	1583.9	0.96	1129.7	0.68	1276.4	0.77
A0A140VK	Testicular secretory protein	1333	1582.4	1.19	931.6	0.70	1596.7	1.20	1267.1	0.95	1527.6	1.15	1622.4	1.22	1341.4	1.01
P61626	Lysozyme C OS=Homo sap	2357.3	3032.5	1.29	2727.6	1.16	1886.7	0.80	2625.8	1.11	2860.8	1.21	2982.3	1.27	2609.5	1.11
E7EMB3	Calmodulin OS=Homo sapi	2471	2441.8	0.99	1992	0.81	1697.6	0.69	2329.9	0.94	2483.3	1.00	2298	0.93	2206.6	0.89
G5EA09	Syndecan binding protein (S	873.8	883.9	1.01	839.2	0.96	687.6	0.79	935.9	1.07	1056.2	1.21	818.4	0.94	776.2	0.89
B0YJ88	Radixin OS=Homo sapiens	241.6	311.9	1.29	233.5	0.97	281.2	1.16	249.1	1.03	280.8	1.16	295.1	1.22	224.3	0.93
Q5SZK8	FRAS1-related extracellular	719	792.6	1.10	737.9	1.03	594.6	0.83	761.9	1.06	773.3	1.08	804.4	1.12	675	0.94
B7ZLI0	Reticulon 4 receptor-like 2 (	2004.2	2245.9	1.12	1658.8	0.83	1591.9	0.79	1972.1	0.98	2026.8	1.01	2360.3	1.18	1624.4	0.81
P36980	Complement factor H-relate	120.1	134.1	1.12	423.3	3.52	103.5	0.86	108.3	0.90	139.9	1.16	112.3	0.94	117.5	0.98
P08779	Keratin, type I cytoskeletal 1	560.6	654	1.17	462.9	0.83	554.2	0.99	480	0.86	472.5	0.84	584	1.04	484	0.86
A0A0B4J1X	Immunoglobulin heavy varia	655	728.4	1.11	496.3	0.76	518.8	0.79	706.7	1.08	598.3	0.91	825	1.26	743.9	1.14
Q9NZZ3	Charged multivesicular body	2219.9	2103.7	0.95	1732.4	0.78	1767.8	0.80	1974.2	0.89	2283.4	1.03	2130.3	0.96	1763.5	0.79
P54760	Ephrin type-B receptor 4 OS	3845.3	4150.1	1.08	3597.8	0.94	3661.3	0.95	3917.5	1.02	4111.6	1.07	3935.4	1.02	3866.3	1.01
B5BU24	14-3-3 protein beta/alpha OS	417.7	540.2	1.29	366.4	0.88	401.3	0.96	437.2	1.05	510.3	1.22	484	1.16	437.3	1.05
P00918	Carbonic anhydrase 2 OS=H	2325.9	3153.7	1.36	2148.2	0.92	2165.8	0.93	2471.6	1.06	3115.3	1.34	3077.6	1.32	2633.3	1.13

Q9NXI0	cDNA FLJ20242 fis, clone	4622.8	4281.8	0.93	4055.2	0.88	3679.7	0.80	4597	0.99	5375.9	1.16	4133.3	0.89	3868	0.84
B4DRC8	cDNA FLJ50047 OS=Hom	1667.8	1742.5	1.04	1525.8	0.91	1295	0.78	1580.4	0.95	1695.9	1.02	1801.9	1.08	1645.9	0.99
P13727	Bone marrow proteoglycan	1435.9	1267.2	0.88	1601.6	1.12	1054.8	0.73	1330.5	0.93	1262.3	0.88	1452.9	1.01	979.8	0.68
A0A024R6U	G protein-coupled receptor	1492.5	1542.9	1.03	1553.7	1.04	1174.7	0.79	1514.9	1.02	1606.5	1.08	1744.4	1.17	1340.7	0.90
Q1HP67	Lipoprotein, Lp(A) OS=Hor	2730.5	2174.3	0.80	2279.4	0.83	2206.5	0.81	2303.6	0.84	2567.9	0.94	2156.5	0.79	2616.5	0.96
P08727	Keratin, type I cytoskeletal	1779.3	2142	1.20	1364.5	0.77	1990.4	1.12	1636.9	0.92	1657.7	0.93	1925.6	1.08	2072.4	1.16
Q9UL86	Myosin-reactive immunoglo	1607.2	1789.8	1.11	1610.9	1.00	1129.5	0.70	1322.2	0.82	1775.7	1.10	1837.6	1.14	2061.6	1.28
P08572	Collagen alpha-2(IV) chain	1789.4	2006.7	1.12	1716.7	0.96	1584.4	0.89	1839.5	1.03	1975.3	1.10	1847.8	1.03	1623.4	0.91
P01037	Cystatin-SN OS=Homo sapi	21.5	24.8	1.15	28.8	1.34	27.4	1.27	21.9	1.02	22.3	1.04	19.6	0.91	19.4	0.90
B7ZL91	Meprin A subunit OS=Hom	2072.7	2291.6	1.11	2357.6	1.14	1893.1	0.91	1972	0.95	2081.8	1.00	2006.6	0.97	1956.4	0.94
Q562M3	Actin-like protein (Fragmen	55.1	74.8	1.36	64.7	1.17	55.9	1.01	55.6	1.01	75.9	1.38	66.1	1.20	64.7	1.17
P08697	Alpha-2-antiplasmin OS=H	1902.8	2105.7	1.11	1715.7	0.90	1461.5	0.77	1904.1	1.00	2323.6	1.22	2062.6	1.08	1827.3	0.96
O43653	Prostate stem cell antigen O	14644.6	12636	0.86	8903	0.61	7470.4	0.51	12814.1	0.88	19102.5	1.30	9102	0.62	7892.3	0.54
B1N7B8	Cryocrystalglobulin CC1 ka	287.4	317.5	1.10	330.5	1.15	195.3	0.68	310.9	1.08	269.9	0.94	372.9	1.30	295.6	1.03
J3KPS3	Fructose-bisphosphate aldol	1637.1	2061.1	1.26	1731.8	1.06	1659.4	1.01	1871	1.14	1945.5	1.19	1787	1.09	1970.7	1.20
E9PKP4	Macrophage colony-stimula	117	161	1.38	130.3	1.11	93.3	0.80	156	1.33	122.2	1.04	153.8	1.31	125.3	1.07
G3V3D1	Epididymal secretory protei	5185.3	5071.2	0.98	4544.1	0.88	4043.5	0.78	5687.7	1.10	5665	1.09	5183.7	1.00	4749.9	0.92
M9MML6	Low affinity immunoglobuli	5907.2	5733	0.97	4810.8	0.81	4415.7	0.75	5931	1.00	5551.6	0.94	4437.6	0.75	5472.1	0.93
K7EKE8	Nectin-2 (Fragment) OS=H	69.3	70.9	1.02	60.7	0.88	61.4	0.89	64.3	0.93	70.6	1.02	67.9	0.98	56.4	0.81
P05787	Keratin, type II cytoskeletal	378.4	585.3	1.55	414.8	1.10	335.9	0.89	397.8	1.05	473.5	1.25	529	1.40	784.1	2.07
V9HW77	Epididymis luminal protein	765.9	884.9	1.16	860	1.12	777.8	1.02	764.6	1.00	831.3	1.09	647.8	0.85	591.7	0.77
Q96KP4	Cytosolic non-specific dipe	620	933.4	1.51	723	1.17	968.5	1.56	723.4	1.17	839.6	1.35	784.8	1.27	687.6	1.11
A0A140VJI	Dipeptidase OS=Homo sapi	1991.3	2516.8	1.26	1651.9	0.83	1699.5	0.85	1992.2	1.00	2163.6	1.09	2087.6	1.05	1612.2	0.81
Q9HCC1	Single chain Fv (Fragment)	59.9	55.8	0.93	45.9	0.77	53.9	0.90	44.4	0.74	50.4	0.84	44	0.73	47.9	0.80
P01624	Immunoglobulin kappa vari	394.7	321.4	0.81	341.2	0.86	274.6	0.70	335.5	0.85	342.8	0.87	380.3	0.96	421.6	1.07
P08238	Heat shock protein HSP 90-	175.5	223.9	1.28	161.2	0.92	144.4	0.82	165.2	0.94	201.9	1.15	220.2	1.25	164.8	0.94
A8K486	Peptidyl-prolyl cis-trans iso	2486.2	3466	1.39	3071.8	1.24	2421.9	0.97	3017.3	1.21	3213.8	1.29	2980.3	1.20	2738	1.10
A8K3I0	cDNA FLJ78437, highly sin	1548.7	1635.7	1.06	1251.2	0.81	1235.1	0.80	1517.7	0.98	1531.9	0.99	1335.8	0.86	1377.3	0.89
H3BUX1	Mesothelin (Fragment) OS=	170.1	249.1	1.46	163.7	0.96	158.5	0.93	163.8	0.96	195.9	1.15	179.5	1.06	159.4	0.94
A5PLM9	Cathepsin L1 OS=Homo sa	2498	2778.1	1.11	2427.9	0.97	1997.4	0.80	2643	1.06	2692.1	1.08	2451.3	0.98	2328	0.93
A0A140VK	Testis tissue sperm-binding	486.1	712.7	1.47	614.6	1.26	655.5	1.35	545.8	1.12	594.5	1.22	648.9	1.33	610.7	1.26
A0A0C4DG	Poliovirus receptor OS=Hor	4425	3861.3	0.87	3487.2	0.79	3365.3	0.76	4192.5	0.95	4276.3	0.97	4492.2	1.02	3807.5	0.86
A0A0G2JN	Ephrin type-B receptor 6 O	1083.3	1132.9	1.05	1158.7	1.07	848.8	0.78	1042.2	0.96	1122.4	1.04	1062.7	0.98	929	0.86
Q92692	Nectin-2 OS=Homo sapiens	2261.7	2270.3	1.00	1939.5	0.86	1743.8	0.77	2226	0.98	2396.9	1.06	2911.6	1.29	2197.8	0.97
H9ZYJ2	Thioredoxin OS=Homo sap	5069.4	5920.6	1.17	4943.4	0.98	3751.3	0.74	4701.9	0.93	5871.3	1.16	4765.7	0.94	5276.5	1.04
V9HW87	Abhydrolase domain contai	868.9	1439.8	1.66	1138.1	1.31	739.7	0.85	917.8	1.06	1029.2	1.18	1228.9	1.41	1033.4	1.19
A0A0S2Z4	Secretoglobin family 1A me	13735	5373.7	0.39	10779.9	0.78	5773	0.42	7533.6	0.55	32076.5	2.34	6110.8	0.44	10733.6	0.78
C9JC84	Fibrinogen gamma chain O	884.6	1762.4	1.99	1127.8	1.27	921.8	1.04	1226.6	1.39	1006.6	1.14	1831.3	2.07	1206.5	1.36
P09228	Cystatin-SA OS=Homo sapi	244.6	278.3	1.14	284.7	1.16	226.7	0.93	292.8	1.20	253.7	1.04	251.4	1.03	237.9	0.97
A8K4G7	cDNA FLJ78528, highly sin	601.9	638	1.06	489.7	0.81	518.1	0.86	602.5	1.00	679.2	1.13	581.9	0.97	499.3	0.83
C9JKV3	Tissue factor pathway inhibi	1101.7	1181.8	1.07	852.8	0.77	901.8	0.82	942.7	0.86	1054.1	0.96	869.6	0.79	927	0.84
S6BGD4	IgG H chain OS=Homo sapi	351.9	392.9	1.12	315.7	0.90	333.3	0.95	324.3	0.92	320.8	0.91	328.6	0.93	425.2	1.21
V9HWE0	Annexin OS=Homo sapiens	1625.8	1873.1	1.15	1360.8	0.84	1963.9	1.21	1753.5	1.08	1865.3	1.15	2098.8	1.29	1405.2	0.86
Q14982	Opioid-binding protein/cell	1754.3	1496.7	0.85	1676.5	0.96	1148.8	0.65	1532.7	0.87	1632.2	0.93	1519.2	0.87	1356	0.77
P13647	Keratin, type II cytoskeletal	11	21.6	1.96	9.1	0.83	7.1	0.65	8.4	0.76	16.2	1.47	22.9	2.08	22.9	2.08
B4E0X1	Beta-2-microglobulin OS=H	8163.5	8072.4	0.99	27383.9	3.35	6068.4	0.74	8892.8	1.09	12376.4	1.52	9033.9	1.11	8006.1	0.98
Q05707	Collagen alpha-1(XIV) chai	720.9	783.6	1.09	614.6	0.85	602.1	0.84	688	0.95	701.7	0.97	696.8	0.97	716.8	0.99
Q76LA1	CSTB protein OS=Homo sa	1626.1	1792.2	1.10	2064.4	1.27	1233	0.76	1965.6	1.21	1713.9	1.05	1753	1.08	1810	1.11
A8K6A7	Alpha-mannosidase OS=Ho	844.5	1033.4	1.22	998	1.18	803.8	0.95	1030.8	1.22	1014.1	1.20	1059.9	1.26	973.7	1.15
Q8N2S1	Latent-transforming growth	739	768	1.04	688	0.93	519.9	0.70	703.5	0.95	698.4	0.95	661.8	0.90	604	0.82
V9HVY1	Epididymis secretory sperm	1138.4	2437.8	2.14	1557.3	1.37	1260.8	1.11	1609.1	1.41	1335.7	1.17	2788.9	2.45	1464.8	1.29
P22352	Glutathione peroxidase 3 O	2075.2	2563.6	1.24	1964.2	0.95	1772.7	0.85	2023.5	0.98	2386.6	1.15	2515.8	1.21	2003.7	0.97
P11717	Cation-independent mannos	1096.8	1217.3	1.11	1113.5	1.02	1042.7	0.95	1078	0.98	1216.5	1.11	1059.4	0.97	956.8	0.87
P39060	Collagen alpha-1(XVIII) ch	1567.8	1910.3	1.22	1467.7	0.94	1426.3	0.91	1755.9	1.12	1902.5	1.21	2016	1.29	1518	0.97
P27348	14-3-3 protein theta OS=Ho	37.8	52.7	1.39	37.6	0.99	34.9	0.92	39	1.03	41.4	1.10	41.7	1.10	39	1.03
B1B5Q3	N-acylsphingosine amidohy	1108.7	1559	1.41	1181	1.07	1166.2	1.05	1323.7	1.19	1705.7	1.54	1682.7	1.52	1500.7	1.35
A0A109PSY	MS-A1 light chain variable	411.4	455	1.11	431.8	1.05	370.7	0.90	444.7	1.08	431.1	1.05	411.4	1.00	428.1	1.04
A0A024R39	Chromosome 11 open readi	1446.6	1945.7	1.35	1614.2	1.12	1283.9	0.89	1666.3	1.15	1650	1.14	2075.1	1.43	1584.4	1.10

Q13421	Mesothelin OS=Homo sapie	1462.1	1033	0.71	1805.3	1.23	751	0.51	1180.2	0.81	1765.3	1.21	1419.5	0.97	1448	0.99
A8K6D3	cDNA FLJ75873, highly sin	1294	1358.1	1.05	1103.2	0.85	988	0.76	1166.2	0.90	1415.7	1.09	1275.2	0.99	1133.8	0.88
Q8NDA2	Hemicentin-2 OS=Homo sa	679.7	741.1	1.09	706.9	1.04	610.5	0.90	663.3	0.98	641.7	0.94	672	0.99	596.5	0.88
A0A024R1C	N-acetylgalactosaminidase,	1806.1	2261.6	1.25	1739.8	0.96	1422.2	0.79	1984.7	1.10	2477.3	1.37	2374.5	1.31	1700.8	0.94
A8K6Q6	cDNA FLJ78639 OS=Homo	837.3	775.7	0.93	685.6	0.82	553	0.66	734.5	0.88	755.4	0.90	737.5	0.88	753.9	0.90
P23284	Peptidyl-prolyl cis-trans isom	1480	1717.7	1.16	1360.3	0.92	1310	0.89	1516.1	1.02	1553.2	1.05	1487.8	1.01	1248.4	0.84
Q14126	Desmoglein-2 OS=Homo sa	704.3	794.2	1.13	800	1.14	692.7	0.98	683.2	0.97	726.5	1.03	770.9	1.09	578.8	0.82
Q59ED3	Intercellular adhesion molec	1945.1	1898.1	0.98	1659.3	0.85	1243.9	0.64	1789.1	0.92	1746.1	0.90	2055.2	1.06	2153.6	1.11
B7Z831	cDNA FLJ55176, highly sin	2556.4	2676.3	1.05	2457.3	0.96	2803.3	1.10	2612.9	1.02	2971.4	1.16	2351.5	0.92	2022.2	0.79
P01700	Immunoglobulin lambda var	1537.5	1520.9	0.99	1749.6	1.14	1154.2	0.75	1661.1	1.08	1558.9	1.01	1592.1	1.04	1656.5	1.08
P29508	Serpin B3 OS=Homo sapier	519.3	561.3	1.08	590.2	1.14	447.5	0.86	790.4	1.52	582.3	1.12	466.5	0.90	684.4	1.32
P98172	Ephrin-B1 OS=Homo sapier	3677.8	3050.7	0.83	3759	1.02	2013.2	0.55	3383.4	0.92	3661.4	1.00	3374.9	0.92	3188.6	0.87
E7EX88	Aggrecan core protein OS=I	1450.7	2024.7	1.40	1575.7	1.09	1306.7	0.90	1555.7	1.07	1556.8	1.07	1473.4	1.02	1417.3	0.98
A2JA16	Anti-mucin1 light chain vari	377.5	702.5	1.86	405.7	1.07	430.9	1.14	485.7	1.29	382.2	1.01	438	1.16	467.6	1.24
Q96J84	Kin of IRRE-like protein 1 (	1097.2	1318.9	1.20	840.3	0.77	986.8	0.90	1015.7	0.93	1048.9	0.96	1137.5	1.04	990.5	0.90
P68104	Elongation factor 1-alpha 1	1493.6	1705.1	1.14	1612.9	1.08	1168.8	0.78	1489.6	1.00	1749.6	1.17	1777.2	1.19	1548.6	1.04
A0A087WV	Osteoclast-associated immu	2857.4	3216.3	1.13	3158.9	1.11	2454.7	0.86	3172.2	1.11	2889.2	1.01	3037.9	1.06	2923.9	1.02
A8KAM8	Platelet-derived growth facto	442.6	488.6	1.10	387.3	0.88	391.2	0.88	417.2	0.94	473.1	1.07	453.9	1.03	423.8	0.96
O43866	CD5 antigen-like OS=Homo	2812.2	2915.9	1.04	2302.2	0.82	2112.3	0.75	2813.1	1.00	2794.9	0.99	2669.5	0.95	2457.7	0.87
Q12841	Follistatin-related protein 1 (	2041.7	2137.1	1.05	2142	1.05	1770.6	0.87	1852	0.91	2137.1	1.05	1484.7	0.73	1429.3	0.70
P07204	Thrombomodulin OS=Homo	383.3	448.8	1.17	342.6	0.89	303.9	0.79	350.7	0.91	372.6	0.97	385.9	1.01	329.5	0.86
I6QTG3	Glypican 3 isoform 2 OS=H	2113.6	2226.9	1.05	1935.4	0.92	1992	0.94	2312	1.09	2217.1	1.05	1851	0.88	1851.5	0.88
P00966	Argininosuccinate synthase	1519.8	2130.2	1.40	1520.8	1.00	1479.7	0.97	1635.5	1.08	2091.1	1.38	1963.9	1.29	1334.6	0.88
Q13621	Solute carrier family 12 mer	1547.5	1528.9	0.99	1645.3	1.06	1148	0.74	1545.9	1.00	1657.9	1.07	1432.5	0.93	1143	0.74
P55285	Cadherin-6 OS=Homo sapie	1088.4	916.1	0.84	913	0.84	740.3	0.68	936.4	0.86	994.5	0.91	1120.4	1.03	965	0.89
B0AZL7	cDNA, FLJ79457, highly sin	753.7	840.1	1.11	715.5	0.95	642.2	0.85	684.2	0.91	780.4	1.04	800.9	1.06	638.8	0.85
A0A075B7E	Protein IGHV3OR16-12 (Fr	1917.7	1959.5	1.02	1475.5	0.77	1827.5	0.95	1905.3	0.99	1850.1	0.96	1647.3	0.86	1845.8	0.96
A0A024R3F	Sortilin-related receptor, L(I	1095.4	993.6	0.91	1061.1	0.97	784.7	0.72	1063.6	0.97	1101.1	1.01	1075	0.98	876.1	0.80
P31151	Protein S100-A7 OS=Homo	1722.6	956.5	0.56	741.2	0.43	659.2	0.38	1743.2	1.01	2212	1.28	1347.3	0.78	1983.1	1.15
M1VKI3	Tyrosine-protein kinase rece	2867	2391.1	0.83	1882.6	0.66	1857.1	0.65	2297.2	0.80	2672.2	0.93	2363.3	0.82	2185.3	0.76
A0A024R03	Complement component 9, i	1203	1434.3	1.19	1387.5	1.15	1429	1.19	1288.4	1.07	1302.7	1.08	1542.2	1.28	1810.6	1.51
Q5KU26	Collectin-12 OS=Homo sap	6136.8	5931.4	0.97	6072	0.99	3325.5	0.54	4204.7	0.69	4897.4	0.80	6084	0.99	4796.8	0.78
Q9UGN4	CMRF35-like molecule 8 O	6215.1	5299.1	0.85	5026.6	0.81	3620.8	0.58	5487.6	0.88	5906.4	0.95	5248	0.84	5819.6	0.94
B7Z1Z5	cDNA FLJ57265, highly sin	1546.6	1432.8	0.93	1276.1	0.83	1208.8	0.78	1321.2	0.85	1382.2	0.89	1366.6	0.88	1347.1	0.87
Q9H8L6	Multimerin-2 OS=Homo sap	2241.8	2527.5	1.13	1925.8	0.86	1892.7	0.84	2305.8	1.03	2331.8	1.04	2521.4	1.12	2189.7	0.98
A8KAQ3	cDNA FLJ76489, highly sin	574.4	660.4	1.15	587.1	1.02	570.5	0.99	630.7	1.10	648.9	1.13	634.6	1.10	557.9	0.97
P40925	Malate dehydrogenase, cyto	1746.5	2093.6	1.20	1746.1	1.00	1809.7	1.04	1889.8	1.08	2335.3	1.34	1978.7	1.13	1738.8	1.00
D3DTX7	Collagen, type I, alpha 1, iso															
A0A109PS5	GCT-A8 light chain variable	213.7	204.4	0.96	205.8	0.96	130.5	0.61	173.9	0.81	187	0.88	207.6	0.97	189.8	0.89
P56537	Eukaryotic translation initiat	805.9	954.1	1.18	753.4	0.93	767.9	0.95	848.3	1.05	864	1.07	941.5	1.17	893.8	1.11
B2R5M3	cDNA, FLJ92530, highly sin	2743.8	2417.7	0.88	1964.8	0.72	2140.9	0.78	2074.8	0.76	2335	0.85	2773.4	1.01	2251.9	0.82
B7Z1F8	cDNA FLJ53025, highly sin															
P08123	Collagen alpha-2(I) chain O	599.1	706.4	1.18	689.9	1.15	526.5	0.88	683.7	1.14	683.1	1.14	830.1	1.39	687.6	1.15
P11047	Laminin subunit gamma-1 O	796.9	780.4	0.98	625.4	0.78	808.8	1.01	698.5	0.88	754	0.95	736.5	0.92	647.9	0.81
A0A024RCJ	Discoidin domain receptor f	805.3	890.6	1.11	650	0.81	705.5	0.88	781.8	0.97	851	1.06	882.7	1.10	712.9	0.89
P14780	Matrix metalloproteinase-9 (	1798	2452.9	1.36	1530.3	0.85	1080.4	0.60	1809.8	1.01	1992.7	1.11	2260.4	1.26	2177.8	1.21
Q8NCC3	Group XV phospholipase A2	1266.8	1659.2	1.31	1849.4	1.46	1093.8	0.86	1593.5	1.26	1799.2	1.42	1720.4	1.36	1399.9	1.11
B3KNB4	cDNA FLJ14168 fis, clone I	102.1	141.7	1.39	103	1.01	143.4	1.40	120	1.18	108.6	1.06	137.7	1.35	103.4	1.01
A0A087WT	Transthyretin OS=Homo sap	1353.3	1516.3	1.12	1328	0.98	1449.7	1.07	1274.6	0.94	1364.1	1.01	1482	1.10	1330	0.98
P19827	Inter-alpha-trypsin inhibitor	440.1	613.5	1.39	471.8	1.07	573.4	1.30	494.3	1.12	480.6	1.09	616.8	1.40	506.3	1.15
Q9UN37	Vacuolar protein sorting-ass	2652.8	2674	1.01	2306.8	0.87	1958.7	0.74	2677.2	1.01	3099.7	1.17	2625.1	0.99	2040	0.77
A0A0S2Z45	Serpin peptidase inhibitor cl	1294.4	1521.9	1.18	1022.6	0.79	1249.1	0.97	1268.9	0.98	1514.8	1.17	1478.5	1.14	1156.2	0.89
P31997	Carcinoembryonic antigen-r	2990.3	3511.7	1.17	3216.7	1.08	2157.7	0.72	3216.6	1.08	3211.6	1.07	2946.1	0.99	3237.4	1.08
Q96JQ0	Protocadherin-16 OS=Homo	663.9	699.5	1.05	615.2	0.93	638.2	0.96	665.4	1.00	699.8	1.05	691.7	1.04	572.8	0.86
P18510	Interleukin-1 receptor antago	254.6	246.8	0.97	266.9	1.05	212.5	0.83	317.9	1.25	259.6	1.02	259.3	1.02	311.8	1.22
B3KUI5	Hyaluronidase OS=Homo sa	584	879.3	1.51	465.3	0.80	570.1	0.98	764.9	1.31	705.2	1.21	691.5	1.18	570.8	0.98
Q15375	Ephrin type-A receptor 7 OS	865.6	1043.8	1.21	781.1	0.90	651.9	0.75	832.8	0.96	827.2	0.96	753.2	0.87	663	0.77
O15230	Laminin subunit alpha-5 OS	1128.4	1277.4	1.13	1384.1	1.23	1257	1.11	1455.4	1.29	1219.5	1.08	1256.7	1.11	1030.6	0.91

Q9ULI3	Protein HEG homolog 1 OS	4254.6	3599.4	0.85	3451.9	0.81	2855.2	0.67	3841.9	0.90	3894.5	0.92	3781.3	0.89	3725.1	0.88
A6NI73	Leukocyte immunoglobulin-	1420.6	1419.2	1.00	1255.4	0.88	1066.2	0.75	1303.5	0.92	1416.8	1.00	1362.8	0.96	1342.9	0.95
Q9Y279	V-set and immunoglobulin d	1900.6	1502.7	0.79	1657.1	0.87	914.9	0.48	1971.7	1.04	1805.8	0.95	1450.6	0.76	1453.1	0.76
S6BAQ4	IgG H chain OS=Homo sapi															
B2R4R0	Histone H4 OS=Homo sapie	1469.8	3267.7	2.22	2510.2	1.71	1327.3	0.90	1672.2	1.14	1782.4	1.21	3202.8	2.18	3831.6	2.61
Q15485	Ficolin-2 OS=Homo sapiens	2120.4	2249.4	1.06	1752.2	0.83	1371.5	0.65	1262.5	0.60	1880.5	0.89	2102.4	0.99	2340.7	1.10
P00338	L-lactate dehydrogenase A c	637.4	828.7	1.30	888	1.39	543.8	0.85	861.6	1.35	945.2	1.48	993.3	1.56	924.8	1.45
Q96S96	Phosphatidylethanolamine-b	4021.4	5705.1	1.42	5915.8	1.47	2936.1	0.73	3959.4	0.98	4666.1	1.16	4031.1	1.00	3204.2	0.80
K7EPJ4	Cartilage intermediate layer	1220.8	1309.1	1.07	1192.2	0.98	944.6	0.77	1219.8	1.00	1235.8	1.01	1188.1	0.97	1081.7	0.89
Q7KYR7	Butyrophilin subfamily 2 me	6869.3	5880.3	0.86	4902.5	0.71	4426.9	0.64	5645.1	0.82	5865.5	0.85	5946.4	0.87	5279.6	0.77
A0A087WX	Mucosal addressin cell adhe	1956.5	1635	0.84	1422.1	0.73	1311.7	0.67	1740	0.89	1698.8	0.87	2030.6	1.04	1689.1	0.86
A0A024RD	Lymphocyte cytosolic prote	623.7	1083.2	1.74	784	1.26	516.2	0.83	823.9	1.32	836.9	1.34	886.5	1.42	1142.3	1.83
A0A0A0N0	CNTFR isoform 3 OS=Hom	3175.4	3060.4	0.96	2938.5	0.93	2418.7	0.76	2809.6	0.88	2998.2	0.94	2725.8	0.86	2676.7	0.84
B2R8I2	cDNA, FLJ93914, highly si	932.5	1246	1.34	1141.8	1.22	840.1	0.90	1117.5	1.20	1001.6	1.07	1314.3	1.41	1197	1.28
P29323	Ephrin type-B receptor 2 OS	252	229.4	0.91	239.8	0.95	184.5	0.73	236.1	0.94	243.8	0.97	239.6	0.95	195.7	0.78
P68363	Tubulin alpha-1B chain OS=	15.9	20.9	1.31	19	1.19	11.8	0.74	18.9	1.19	18.5	1.16	19.7	1.24	16.4	1.03
A8K6K4	cDNA FLJ77565, highly sin	1392.7	1483.1	1.06	1504.4	1.08	1208.4	0.87	1401.7	1.01	1489	1.07	1408.5	1.01	1384.1	0.99
P51688	N-sulphoglucosamine sulpho	582.5	917.1	1.57	561.5	0.96	525.9	0.90	676.5	1.16	662.6	1.14	827.3	1.42	624.4	1.07
B7Z1K5	Tubulin alpha chain OS=Ho	325.4	465.8	1.43	406.6	1.25	342.3	1.05	353.2	1.09	397.9	1.22	385.2	1.18	328.3	1.01
S6BAP4	IgG H chain OS=Homo sapi	14.6	26.2	1.79	36.7	2.51	23.2	1.59	53.7	3.68	20.7	1.42	13.6	0.93	19.7	1.35
B4DPQ0	Complement C1r subcompo	239.7	283.8	1.18	233.2	0.97	191.1	0.80	247.3	1.03	237.5	0.99	244.2	1.02	251.2	1.05
B7ZKQ8	PODXL protein OS=Homo	3403.1	3202.6	0.94	2843.3	0.84	2466.7	0.72	3141.1	0.92	3425.7	1.01	3439.7	1.01	2693.7	0.79
O75487	Glypican-4 OS=Homo sapie	581.4	606.7	1.04	544	0.94	536	0.92	593.3	1.02	617.9	1.06	615.2	1.06	507.1	0.87
B2R701	cDNA, FLJ93202, Homo sa	30.3	25.1	0.83	30	0.99	20.1	0.66	25.4	0.84	24.7	0.82	23.6	0.78	23.4	0.77
P52758	Ribonuclease UK114 OS=H	539.6	669.4	1.24	526.4	0.98	508	0.94	569.9	1.06	663.4	1.23	621.3	1.15	498.3	0.92
G3XAK1	Hepatocyte growth factor-lil	1427.9	1531.2	1.07	1406.8	0.99	1271	0.89	1426.6	1.00	1515.2	1.06	1369.1	0.96	1451.5	1.02
V9HWC7	Epididymis secretory sperm	1373.1	1758.3	1.28	1425.6	1.04	1328.4	0.97	1458.9	1.06	1803.2	1.31	1594.8	1.16	1296.7	0.94
Q9H665	IGF-like family receptor 1 C	1955.6	1816.2	0.93	1642.3	0.84	1422.6	0.73	1945.8	0.99	1979.3	1.01	1855.6	0.95	1805.5	0.92
A0A125U0V	MS-F1 heavy chain variable															
B8ZWD9	Diazepam binding inhibitor,	1884.1	1883	1.00	1852.7	0.98	1540.4	0.82	1782.7	0.95	1839.2	0.98	1173.8	0.62	1657.3	0.88
P25940	Collagen alpha-3(V) chain C	258.1	269	1.04	230.1	0.89	314.2	1.22	250.9	0.97	252.9	0.98	229.1	0.89	235.6	0.91
O75942	Major prion protein OS=Ho	3584	3095	0.86	2786.1	0.78	2538	0.71	2956.3	0.82	3476.6	0.97	2890.9	0.81	2604.5	0.73
P0DJD8	Pepsin A-3 OS=Homo sapie	22700.4	19671	0.87	18981.8	0.84	15407.6	0.68	26908.4	1.19	24011.9	1.06	34342.5	1.51	19145.7	0.84
Q07954	Prolow-density lipoprotein r	2483.3	2617.6	1.05	2206.5	0.89	1916.2	0.77	2396.9	0.97	2485.3	1.00	2467.2	0.99	2086.3	0.84
O14745	Na(+)/H(+) exchange regula	1481.2	1769.7	1.19	1223.1	0.83	1195.9	0.81	1283	0.87	1539.7	1.04	1621.2	1.09	1203	0.81
A0A0A0MR	Immunoglobulin kappa varia	103.8	111.8	1.08	105.3	1.01	81.6	0.79	106.7	1.03	108.3	1.04	110.6	1.07	98.8	0.95
A2NXP8	Heavy chain variable region	85.1	93.6	1.10	87.4	1.03	107.2	1.26	87.3	1.03	100.2	1.18	88.4	1.04	97.5	1.15
P05546	Heparin cofactor 2 OS=Hon	886.9	964.9	1.09	883.6	1.00	856	0.97	944.4	1.06	953.3	1.07	1098.7	1.24	921.4	1.04
P34896	Serine hydroxymethyltransfe	382.3	549.9	1.44	402.2	1.05	369.5	0.97	396.3	1.04	505.3	1.32	520.1	1.36	398.3	1.04
A0A024R05	Guanine nucleotide binding	80.9	84.4	1.04	96.5	1.19	64.2	0.79	84.8	1.05	85.2	1.05	79.6	0.98	69.6	0.86
A0A087WV	CD177 antigen OS=Homo s	2340.8	2384.2	1.02	2654.3	1.13	2125.4	0.91	2433.4	1.04	2768.1	1.18	2120.2	0.91	1900.9	0.81
P30044	Peroxiredoxin-5, mitochond	908.4	982.8	1.08	936.2	1.03	647.4	0.71	893.8	0.98	979	1.08	982.2	1.08	894.2	0.98
P05362	Intercellular adhesion molec	1699.8	1658.4	0.98	1661.3	0.98	1164.3	0.68	1576.1	0.93	1659.3	0.98	1764.4	1.04	1716.8	1.01
B2R7S8	cDNA, FLJ93586, highly si	426.3	448.1	1.05	353.8	0.83	398.5	0.93	382	0.90	440.4	1.03	426.9	1.00	424.1	0.99
A8K5I6	cDNA FLJ78643, highly sin	1669.6	2198.4	1.32	1626.9	0.97	1167.8	0.70	1735.9	1.04	1692.7	1.01	1782.4	1.07	2625.8	1.57
P48061	Stromal cell-derived factor 1	2541.3	2991.8	1.18	3366.1	1.32	2422.9	0.95	3085.8	1.21	3437.8	1.35	3411.9	1.34	3247.7	1.28
Q6V0I7	Protocadherin Fat 4 OS=Ho	719.2	736.9	1.02	728.6	1.01	580.1	0.81	732.5	1.02	748.9	1.04	753.1	1.05	594	0.83
Q5U000	Cathepsin Z OS=Homo sapi	3261.1	3462.5	1.06	4858.2	1.49	2648.4	0.81	3428.6	1.05	3545.8	1.09	3525.8	1.08	3138.2	0.96
Q9BRK5	45 kDa calcium-binding pro	1047.6	1225.7	1.17	966.4	0.92	980.6	0.94	1020.7	0.97	1077.1	1.03	1197.8	1.14	955.2	0.91
P55000	Secreted Ly-6/uPAR-related	663.4	419.5	0.63	470.6	0.71	283.5	0.43	441.1	0.66	531.6	0.80	468.5	0.71	399.8	0.60
P54753	Ephrin type-B receptor 3 OS	352.1	336	0.95	295.4	0.84	292.5	0.83	336.9	0.96	354.3	1.01	347.4	0.99	331.4	0.94
H3BLU2	Limbic system-associated m	686.6	599.9	0.87	604.2	0.88	601.9	0.88	570.2	0.83	628.9	0.92	554.6	0.81	551.7	0.80
Q59FR8	Galectin (Fragment) OS=Ho	800.5	871.6	1.09	777.7	0.97	636.1	0.79	816.7	1.02	937.7	1.17	857.6	1.07	767.2	0.96
P16152	Carbonyl reductase [NADPH	385.5	462.6	1.20	440.1	1.14	335.6	0.87	405.9	1.05	465.1	1.21	431	1.12	341.6	0.89
Q9UBX7	Kallikrein-11 OS=Homo sap	2722.4	2886.9	1.06	3948.2	1.45	2651.8	0.97	2340.6	0.86	3219.7	1.18	1809.4	0.66	1938.5	0.71
A0A0C4DH	Immunoglobulin kappa varia	2826.7	2440.4	0.86	3158.5	1.12	1930.8	0.68	2450.8	0.87	2371.9	0.84	2725.7	0.96	3150.4	1.11
A0A024R64	Ceroid-lipofuscinosis neuror	1477.6	1759.1	1.19	1227	0.83	1184.9	0.80	1466.2	0.99	1856.7	1.26	1724.2	1.17	1388.2	0.94
P09237	Matrilysin OS=Homo sapien	1214.8	1379.4	1.14	1155.3	0.95	1118.1	0.92	1249.5	1.03	1364.3	1.12	1583.5	1.30	1306.9	1.08

A0A1A7UP	Galactosamine (N-acetyl)-6-	997.6	1416.8	1.42	735.3	0.74	965.6	0.97	1109.4	1.11	970.4	0.97	1418.9	1.42	1134.1	1.14
Q16849	Receptor-type tyrosine-prote	1455.7	1406.2	0.97	1093	0.75	1081.4	0.74	1228	0.84	1230.5	0.85	1692.9	1.16	1051.5	0.72
A0A125U0U	MS-C1 heavy chain variable	1014	952	0.94	793.7	0.78	912.6	0.90	934.2	0.92	1014.9	1.00	876.2	0.86	991.6	0.98
Q9HB40	Retinoid-inducible serine ca	904.6	1368.6	1.51	929.5	1.03	855	0.95	1095.1	1.21	1193.1	1.32	1368.5	1.51	883.1	0.98
A0A024R0C	Phospholipase D family, me	1692.7	2048.1	1.21	1356.2	0.80	1394.3	0.82	1730.1	1.02	1788.3	1.06	2098.2	1.24	1540	0.91
A0A0A0MT	Glucose-6-phosphate isomer	945.3	1311.3	1.39	995.2	1.05	873.4	0.92	1108.3	1.17	1193.8	1.26	1075.8	1.14	975.4	1.03
Q02747	Guanylin OS=Homo sapiens	2797.1	2596.8	0.93	6099.4	2.18	2609	0.93	2855.8	1.02	3234.9	1.16	2920.1	1.04	2336.8	0.84
A0A024R5N	Tumor necrosis factor recep	829.6	818.8	0.99	644.6	0.78	636.5	0.77	707.2	0.85	718.3	0.87	843.3	1.02	755	0.91
A0A024R5F	UDP-GlcNAc:betaGal beta-	751	921.3	1.23	710	0.95	707.1	0.94	823.4	1.10	950.1	1.27	955.7	1.27	763.9	1.02
P09564	T-cell antigen CD7 OS=Hor	271.3	233.9	0.86	178.6	0.66	345.4	1.27	206	0.76	250.6	0.92	226.9	0.84	245.6	0.91
E9PK25	Cofilin-1 OS=Homo sapiens	2864.2	3775.1	1.32	2670.2	0.93	2332.4	0.81	3004.5	1.05	3519.9	1.23	3036.5	1.06	3144.5	1.10
A0A087WV	Receptor-type tyrosine-prote	682.1	767.1	1.12	538.4	0.79	734.8	1.08	649	0.95	717.5	1.05	641.8	0.94	594	0.87
A4GW21	Programmed cell death ligar	1066.1	1167.3	1.09	955.3	0.90	862	0.81	974.5	0.91	1115.6	1.05	1052.4	0.99	1021.5	0.96
O75339	Cartilage intermediate layer	1295.6	1246.8	0.96	1186.7	0.92	939.7	0.73	1242.8	0.96	1197.2	0.92	1059.7	0.82	1119.3	0.86
A2JA19	Anti-mucin1 light chain vari	138.6	163	1.18	168.3	1.21	124	0.89	141.4	1.02	147	1.06	144.1	1.04	136.8	0.99
Q99497	Protein deglycase DJ-1 OS=	1867.4	2063.6	1.11	1872.6	1.00	1541.6	0.83	1821.2	0.98	2230.1	1.19	1856.2	0.99	1771.2	0.95
P18827	Syndecan-1 OS=Homo sapie	1571.3	1616.4	1.03	1237.2	0.79	1224.3	0.78	1457.9	0.93	1727.9	1.10	1534.3	0.98	1402.8	0.89
Q9NPF0	CD320 antigen OS=Homo s	3112.6	2926.8	0.94	2570	0.83	1843.2	0.59	2671.6	0.86	3013.7	0.97	2917.4	0.94	2555.1	0.82
P00915	Carbonic anhydrase 1 OS=H	816.3	1363.4	1.67	1485.8	1.82	623.1	0.76	738.5	0.90	1088.6	1.33	923.7	1.13	994.8	1.22
P04155	Trefoil factor 1 OS=Homo s	2289.6	2198.7	0.96	2084.6	0.91	2264.9	0.99	2235.2	0.98	2342.5	1.02	3474.8	1.52	3091.6	1.35
O95998	Interleukin-18-binding prote	4387.8	4158.9	0.95	3175.2	0.72	3770.7	0.86	3832.7	0.87	3758.2	0.86	4524	1.03	4016.7	0.92
Q99969	Retinoic acid receptor respo	449.9	505.7	1.12	508.8	1.13	437.5	0.97	486.1	1.08	541.1	1.20	481.2	1.07	430.8	0.96
E5RH16	Platelet-derived growth fact	59.7	60.5	1.01	60.7	1.02	50.1	0.84	60.9	1.02	57.2	0.96	59.8	1.00	58.9	0.99
P62879	Guanine nucleotide-binding	1397.1	1616.4	1.16	1366.8	0.98	1145.1	0.82	1487.9	1.06	1739.7	1.25	1691.8	1.21	1196.3	0.86
Q01469	Fatty acid-binding protein, e	974.9	934.5	0.96	1062.7	1.09	1055.3	1.08	1016.3	1.04	918.7	0.94	1262.6	1.30	1265.8	1.30
V9HWJ5	Nicotinate-nucleotide pyrop	719.5	1006	1.40	939.3	1.31	811.6	1.13	825.3	1.15	849.6	1.18	779.3	1.08	745.4	1.04
P23526	Adenosylhomocysteinase O	875	1319.8	1.51	1072.6	1.23	802	0.92	993.8	1.14	1252	1.43	1151.6	1.32	866.3	0.99
Q8TB96	T-cell immunomodulatory p	386.3	442.7	1.15	408.2	1.06	358.1	0.93	417.6	1.08	417.3	1.08	414.8	1.07	370	0.96
P11279	Lysosome-associated memb	5517	5279.3	0.96	4792	0.87	3348.7	0.61	5389.9	0.98	5921.8	1.07	4877.7	0.88	5000.5	0.91
D3YTC8	Leukocyte-associated immu															
Q9BX67	Junctional adhesion molecul	473.9	440.1	0.93	407.4	0.86	405.7	0.86	460.6	0.97	492.1	1.04	441.2	0.93	451.5	0.95
A0A024RD	EGF-like-domain, multiple 9	518.3	541.6	1.04	467.8	0.90	478.3	0.92	493	0.95	484.3	0.93	490.5	0.95	492.3	0.95
P19438	Tumor necrosis factor recep	513.2	539.5	1.05	540.6	1.05	434.3	0.85	510.2	0.99	496.5	0.97	524.7	1.02	466.3	0.91
Q8NHP8	Putative phospholipase B-lik	2249.8	2171.8	0.97	1924	0.86	1367.5	0.61	2235.3	0.99	2171.5	0.97	2546	1.13	2186.1	0.97
P05023	Sodium/potassium-transport	411.9	417.9	1.01	350.6	0.85	341.9	0.83	392.1	0.95	373	0.91	482.4	1.17	310.2	0.75
P20160	Azurocidin OS=Homo sapie	611.6	960.4	1.57	888.2	1.45	623.3	1.02	761.4	1.24	742.3	1.21	1039.5	1.70	905.4	1.48
B7Z5W1	cDNA FLJ54854, highly sin	1842.3	1509.2	0.82	1737.6	0.94	1145.9	0.62	1702	0.92	1928	1.05	1726.8	0.94	1606.1	0.87
A0A140VJX	Sulfurtransferase OS=Homo	403.3	549.9	1.36	448.9	1.11	369.6	0.92	470.2	1.17	472.9	1.17	576	1.43	509.9	1.26
A0A120HG	GCT-A10 heavy chain varia	1703.1	1922.5	1.13	1638.4	0.96	1808	1.06	2062.5	1.21	1959.5	1.15	1575.8	0.93	1823.5	1.07
Q5M8T4	Connective tissue growth fa	1247.4	1202.6	0.96	1235.1	0.99	850.1	0.68	1162.3	0.93	1233	0.99	1104.8	0.89	1088.2	0.87
Q9BRT3	Migration and invasion enha	1376.7	1383.7	1.01	1089.7	0.79	975.1	0.71	1201.4	0.87	1505.4	1.09	1386.6	1.01	1229.2	0.89
P49788	Retinoic acid receptor respo	965.3	899.5	0.93	705	0.73	816.9	0.85	719.7	0.75	1750.1	1.81	767.3	0.79	819.1	0.85
P31025	Lipocalin-1 OS=Homo sapie	1461.4	1277.4	0.87	1986	1.36	1010.4	0.69	1643.2	1.12	1591	1.09	1342.4	0.92	1319	0.90
A0A075B6H	Protein IGKV3-7 (Fragment	489.9	409.2	0.84	411.4	0.84	299.4	0.61	370.9	0.76	404	0.82	428.4	0.87	433.5	0.88
Q9BZG9	Ly-6/neurotoxin-like protein	8447.9	7236.8	0.86	6557.9	0.78	4311.3	0.51	6534.3	0.77	7253.8	0.86	6545.8	0.77	5953.8	0.70
A2VCQ3	ROR1 protein (Fragment) O	951.7	890.4	0.94	802.8	0.84	658.5	0.69	911.6	0.96	893.9	0.94	1004.3	1.06	840.5	0.88
P36957	Dihydrolipoyllysine-residue	5668.5	5434.4	0.96	4096	0.72	3714.7	0.66	4739.8	0.84	5642	1.00	5392.6	0.95	4462.1	0.79
A0A1C9J6T	B cell receptor heavy chain															
A0A024R1A	Testicular secretory protein	133.1	235.7	1.77	162.6	1.22	154.9	1.16	138	1.04	175.3	1.32	166	1.25	144.1	1.08
Q5JWF2	Guanine nucleotide-binding	907.3	945.2	1.04	776.6	0.86	641.8	0.71	896	0.99	1042.8	1.15	887.2	0.98	665.8	0.73
A0A0G2JM	Cadherin-related family mer	249	287.3	1.15	188.5	0.76	181.9	0.73	193.4	0.78	244.6	0.98	218.6	0.88	205	0.82
P13473	Lysosome-associated memb	8958.6	7456.8	0.83	5745.4	0.64	4694	0.52	8648.5	0.97	8424.4	0.94	8179.5	0.91	8798.8	0.98
A0A024R93	Proteoglycan 4, isoform CR	5168.8	5246.9	1.02	6325.3	1.22	3913.3	0.76	5340.3	1.03	5389.9	1.04	4946.1	0.96	4970.3	0.96
P12821	Angiotensin-converting enzy	747.9	740.1	0.99	581.7	0.78	632.1	0.85	680.9	0.91	787.1	1.05	598.5	0.80	535.7	0.72
B7Z4R8	cDNA FLJ53364, highly sin	47.5	41.2	0.87	45.6	0.96	32.5	0.68	41.7	0.88	43	0.91	39.1	0.82	46.8	0.99
A0A024R99	Copine III, isoform CRA_a	638.9	691.8	1.08	541.4	0.85	644.4	1.01	631.9	0.99	758.2	1.19	614.3	0.96	679.3	1.06
Q5U0I6	H.sapiens ras-related Hrab1.	545.7	686.2	1.26	503.6	0.92	422.1	0.77	533.1	0.98	639.4	1.17	578.7	1.06	473.3	0.87
A0A068LL6	Ig heavy chain variable regi	689.2	743.5	1.08	507.5	0.74	616.8	0.89	684.1	0.99	647.7	0.94	614.2	0.89	812.6	1.18

A0A024R84	RAB14, member RAS onco	825.9	917.1	1.11	801.1	0.97	655.2	0.79	803.2	0.97	925.2	1.12	778.1	0.94	659.6	0.80
P12814	Alpha-actinin-1 OS=Homo s															
Q14393	Growth arrest-specific prote	343.2	327.5	0.95	255.8	0.75	262.6	0.77	325.5	0.95	352.3	1.03	338	0.98	295.3	0.86
P05413	Fatty acid-binding protein, h	1140.6	1274.9	1.12	1111.2	0.97	1041.8	0.91	1062.1	0.93	1301.3	1.14	1110.2	0.97	1069.6	0.94
A0A140VJT	Testicular tissue protein Li 1	298.5	400.6	1.34	273.3	0.92	353.4	1.18	316.9	1.06	365.3	1.22	342.3	1.15	300.4	1.01
A0A0C4DH	Immunoglobulin kappa varia	606.6	715.6	1.18	871	1.44	621.8	1.03	757.6	1.25	637.5	1.05	602.2	0.99	673.1	1.11
Q09666	Neuroblast differentiation-as	587.5	663.1	1.13	632.7	1.08	568.9	0.97	614.5	1.05	578.4	0.98	594.6	1.01	610.6	1.04
P14550	Alcohol dehydrogenase [NA	354	410.8	1.16	336.3	0.95	313.8	0.89	345.3	0.98	456.9	1.29	424.2	1.20	442.7	1.25
Q16832	Discoidin domain-containing	411.7	459.6	1.12	366.7	0.89	357.2	0.87	390.8	0.95	492.4	1.20	440.7	1.07	357.1	0.87
P16284	Platelet endothelial cell adhe															
Q9HC84	Mucin-5B OS=Homo sapien	821.2	643.3	0.78	529.5	0.64	669.4	0.82	613.9	0.75	811.7	0.99	567.5	0.69	538.9	0.66
Q92859	Neogenin OS=Homo sapien	797.5	786.5	0.99	701.2	0.88	594.3	0.75	757.9	0.95	790.5	0.99	746.3	0.94	683.4	0.86
Q7Z738	PCDH12 protein OS=Homo	177.3	207.9	1.17	150	0.85	237.3	1.34	168.5	0.95	164.1	0.93	183.6	1.04	157.8	0.89
Q05CP7	FABP1 protein (Fragment) (	1692.4	2216	1.31	1837.7	1.09	1758	1.04	1669.3	0.99	2070.9	1.22	1954.2	1.15	1419.3	0.84
H0Y3Z8	Uncharacterized protein (Fra	789	733.1	0.93	563.1	0.71	587	0.74	656.7	0.83	701.9	0.89	724.7	0.92	661	0.84
A0A024RA	Glutaredoxin (Thioltransfera	717.9	764.1	1.06	984.8	1.37	498.7	0.69	703.7	0.98	803.2	1.12	812.2	1.13	651.2	0.91
Q99988	Growth/differentiation facto	1308.6	1396.2	1.07	1660.6	1.27	867.2	0.66	1524.9	1.17	1300.5	0.99	1446.9	1.11	1648.4	1.26
A0A087WW	Immunoglobulin kappa varia	286.9	333.2	1.16	383	1.33	336.3	1.17	385	1.34	318.4	1.11	302.9	1.06	297.2	1.04
Q14315	Filamin-C OS=Homo sapien	464.2	545.7	1.18	449	0.97	372.6	0.80	492.7	1.06	499.1	1.08	492.8	1.06	391.6	0.84
A0A0X9V9	MS-F1 light chain variable r	1807.9	1476.4	0.82	1846.8	1.02	1351	0.75	1398.8	0.77	1486.8	0.82	2127.1	1.18	1949	1.08
A0A125U0	GCT-A1 heavy chain variab	105.3	106.2	1.01	88.3	0.84	83.2	0.79	106.5	1.01	105.8	1.00	164.3	1.56	97.5	0.93
Q9BVM4	Gamma-glutamylaminecyclo	675.7	840.9	1.24	684.9	1.01	543	0.80	658.2	0.97	753.6	1.12	840	1.24	641.6	0.95
Q5IJ48	Protein crumbs homolog 2 (	157.7	177	1.12	176.9	1.12	129.6	0.82	146.6	0.93	163	1.03	191.6	1.21	151.1	0.96
Q14247	Src substrate cortactin OS=I	1133.4	1053.6	0.93	1066.9	0.94	675.4	0.60	1153.7	1.02	1152	1.02	1409	1.24	985.8	0.87
A0A075B73	Platelet endothelial cell adhe	1259	1254.7	1.00	1009.5	0.80	957.3	0.76	1165.9	0.93	1238.1	0.98	1242.9	0.99	1141.4	0.91
P13671	Complement component C6	601.5	671.3	1.12	485.9	0.81	566	0.94	606.5	1.01	682.6	1.13	704.2	1.17	721.3	1.20
A0A024R32	Filamin B, beta (Actin bindi	491.2	528.1	1.08	466.7	0.95	436.8	0.89	526.7	1.07	484.2	0.99	578.2	1.18	464	0.94
Q0IIN1	Keratin 77 OS=Homo sapie															
B2R6X2	Beta-glucuronidase OS=Hor	770.1	799.7	1.04	637.9	0.83	550.7	0.72	661.5	0.86	939	1.22	651.2	0.85	616.5	0.80
P05556	Integrin beta-1 OS=Homo s	1556.3	1658.1	1.07	1430.4	0.92	1255	0.81	1600.5	1.03	1658	1.07	1609	1.03	1466.7	0.94
P55017	Solute carrier family 12 mer	580.6	602.3	1.04	476.9	0.82	399.7	0.69	580	1.00	657.4	1.13	615	1.06	457.6	0.79
P02461	Collagen alpha-1(III) chain (	1488.3	1504.5	1.01	1365.4	0.92	890.7	0.60	2066.9	1.39	1607.6	1.08	1983.7	1.33	2122.8	1.43
A0A0S2Z2	Annexin (Fragment) OS=Ho	709.9	1041.6	1.47	728	1.03	683.4	0.96	838	1.18	870.3	1.23	895.2	1.26	700.5	0.99
B1N7B6	Cryocrystalglobulin CC1 he	1256.3	1379.4	1.10	1130.3	0.90	1393.4	1.11	1481.1	1.18	1286.5	1.02	1308.8	1.04	1399.1	1.11
A2NJV5	Kappa light chain variable re	127.9	86.5	0.68	132.6	1.04	79.8	0.62	100.4	0.78	118.7	0.93	129.3	1.01	135.3	1.06
Q86Y38	Xylosyltransferase 1 OS=Ho	547.6	508.5	0.93	548.5	1.00	434.3	0.79	608.2	1.11	512.6	0.94	666.5	1.22	465.9	0.85
Q96AP7	Endothelial cell-selective ad	1100.2	949.4	0.86	1030.7	0.94	771.5	0.70	983.9	0.89	1078.9	0.98	1073.7	0.98	948.1	0.86
A0A024RD	G protein-coupled receptor	327.9	325.9	0.99	314.8	0.96	275.7	0.84	293.6	0.90	341.7	1.04	312	0.95	274	0.84
Q16651	Prostasin OS=Homo sapiens	2330.8	1888.9	0.81	2031.5	0.87	1920.1	0.82	1707.8	0.73	2054.7	0.88	1905.5	0.82	1788.3	0.77
P12429	Annexin A3 OS=Homo sapi	120.9	175.6	1.45	148.9	1.23	160	1.32	183.5	1.52	145.8	1.21	149.1	1.23	138.5	1.15
A8K0I8	cDNA FLJ76207, highly sin	574	553.4	0.96	460.4	0.80	432.1	0.75	512.7	0.89	562	0.98	518.6	0.90	462.7	0.81
P01766	Immunoglobulin heavy varia	115.1	143.9	1.25	143.3	1.25	137.3	1.19	178.7	1.55	125.9	1.09	133.1	1.16	141.5	1.23
O60888	Protein CutA OS=Homo sap	922.3	802.2	0.87	671.5	0.73	646.8	0.70	717.4	0.78	852.5	0.92	839.7	0.91	722	0.78
B2R657	Annexin OS=Homo sapiens	64.6	87.2	1.35	56	0.87	72.6	1.12	72	1.11	85.5	1.32	75.1	1.16	64.4	1.00
Q6FIE5	PHP14 protein OS=Homo s	1824.2	2307.3	1.26	1936.1	1.06	1452.8	0.80	2029.7	1.11	2186.5	1.20	1953.3	1.07	2081.4	1.14
B2RAM2	cDNA, FLJ94999, highly sir	670.9	772.1	1.15	718.3	1.07	675.4	1.01	724.5	1.08	780.6	1.16	623	0.93	528.4	0.79
O00337	Sodium/nucleoside cotransp	198.9	189	0.95	148.6	0.75	147.2	0.74	183.1	0.92	184	0.93	125.3	0.63	147.1	0.74
A8K987	cDNA FLJ77911, highly sin	3254.9	4857.3	1.49	3701	1.14	2361.3	0.73	3403.1	1.05	5215.5	1.60	3920.5	1.20	2928.1	0.90
P08263	Glutathione S-transferase A	331	515.5	1.56	439.4	1.33	348.2	1.05	386.9	1.17	470.7	1.42	586.6	1.77	350.8	1.06
A0A024R52	Dihydroxyacetone kinase 2	347.7	448.1	1.29	386.8	1.11	288.1	0.83	368.8	1.06	456.2	1.31	513	1.48	339.9	0.98
P40189	Interleukin-6 receptor subun	1487.4	1502.2	1.01	1535.8	1.03	1236.8	0.83	1489	1.00	1571.1	1.06	1537.7	1.03	1304.8	0.88
Q5NV90	V2-17 protein (Fragment) O	601.5	719.5	1.20	731.6	1.22	703	1.17	678.5	1.13	774.5	1.29	746.6	1.24	714.1	1.19
P22528	Cornifin-B OS=Homo sapie	2109	1716.5	0.81	2446.7	1.16	1832.6	0.87	2536.8	1.20	2307.6	1.09	1763.9	0.84	2761.7	1.31
Q9UN70	Protocadherin gamma-C3 O	2507.6	2723.8	1.09	2196.7	0.88	2021.5	0.81	2364.5	0.94	2665.5	1.06	2478.2	0.99	2172.8	0.87
A8MTF8	Protein FAM3B OS=Homo	692.4	701.2	1.01	620.2	0.90	492.3	0.71	644.8	0.93	757	1.09	662	0.96	621	0.90
P20336	Ras-related protein Rab-3A	264.4	288	1.09	323.8	1.22	274.1	1.04	270.9	1.02	263.1	1.00	213.9	0.81	203.9	0.77
Q8WVQ1	Soluble calcium-activated nu	308.3	330.6	1.07	258.7	0.84	273.8	0.89	272.7	0.88	352.1	1.14	284	0.92	239.9	0.78
B8PRF2	CD160 antigen OS=Homo s	779.3	721.9	0.93	696	0.89	675.2	0.87	659.5	0.85	763.6	0.98	935.9	1.20	605	0.78

A0A0A0MR	Coagulation factor V OS=H	358.9	300.9	0.84	315.4	0.88	245	0.68	312	0.87	361.4	1.01	336.1	0.94	333.1	0.93
Q7LBR1	Charged multivesicular body	2977.5	2648.7	0.89	2505.2	0.84	2824	0.95	3025.5	1.02	3153.4	1.06	2759	0.93	2295.7	0.77
P13797	Plastin-3 OS=Homo sapiens	38.2	72.7	1.90	101.6	2.66	41.1	1.08	42.1	1.10	40.3	1.05	39.3	1.03	40.6	1.06
Q53G35	Phosphoglycerate mutase (F	231.3	281.4	1.22	242.1	1.05	226.4	0.98	255.6	1.11	219.9	0.95	238.6	1.03	228	0.99
Q9NY97	N-acetyllactosaminide beta-	721.8	919.2	1.27	824.8	1.14	809.8	1.12	820.4	1.14	864.8	1.20	836.3	1.16	737.5	1.02
A0A024R1S	LIM and SH3 protein 1, iso	429.2	403.7	0.94	359.7	0.84	339.5	0.79	378.4	0.88	432.5	1.01	367.5	0.86	354.1	0.83
Q9Y509	VH3 protein (Fragment) OS	704.9	906.8	1.29	727.1	1.03	827.2	1.17	808.2	1.15	768.7	1.09	750.8	1.07	821.1	1.16
G8JLH6	Tetraspanin (Fragment) OS=	7477.1	6145.2	0.82	6435.4	0.86	5012.1	0.67	7231.1	0.97	8554.5	1.14	5538.3	0.74	4966.4	0.66
Q9BTY2	Plasma alpha-L-fucosidase (	625.1	746.3	1.19	610.9	0.98	619.2	0.99	687	1.10	796.3	1.27	708.6	1.13	715	1.14
P22732	Solute carrier family 2, facil	1407.4	1530	1.09	1084.8	0.77	1228	0.87	1507.9	1.07	1666.2	1.18	1386.6	0.99	903.4	0.64
P35443	Thrombospondin-4 OS=Hon	147.1	119.7	0.81	124.8	0.85	148.9	1.01	135.3	0.92	123.5	0.84	121.2	0.82	138.8	0.94
A0A0A1HA	H.sapiens ras-related Hrab3															
Q9UL84	Myosin-reactive immunoglo	216.8	307.4	1.42	208	0.96	219	1.01	216.9	1.00	226.7	1.05	233	1.07	343.2	1.58
Q9NQ36	Signal peptide, CUB and EC	688.2	589	0.86	696.4	1.01	445.5	0.65	694	1.01	649.5	0.94	673.3	0.98	556.2	0.81
A8K2Q6	Peptidyl-prolyl cis-trans isor	712.3	821.1	1.15	599.6	0.84	531.2	0.75	791.4	1.11	838.2	1.18	931.8	1.31	658.8	0.92
Q14894	Ketimine reductase mu-crys	400.1	566.4	1.42	422.1	1.05	567.6	1.42	444.8	1.11	511.4	1.28	480.9	1.20	432.6	1.08
Q9ULV1	Frizzled-4 OS=Homo sapien	2293.8	2061.7	0.90	2021.7	0.88	1398.8	0.61	2209.9	0.96	2362.7	1.03	1983.4	0.86	1839.2	0.80
Q9Y2S2	Lambda-crystallin homolog	653.3	1726.9	2.64	870.9	1.33	639.3	0.98	725.1	1.11	893.1	1.37	1034.1	1.58	693	1.06
Q6UX71	Plexin domain-containing pr	879.6	1042.3	1.18	812.5	0.92	697.6	0.79	857.4	0.97	847.8	0.96	987.5	1.12	751.1	0.85
P04066	Tissue alpha-L-fucosidase C	592.1	782	1.32	578.6	0.98	594.6	1.00	729.5	1.23	854.4	1.44	783.6	1.32	660.8	1.12
Q9H6X2	Anthrax toxin receptor 1 OS	962.8	915.6	0.95	1105.3	1.15	892.6	0.93	928.1	0.96	932.3	0.97	905.1	0.94	923.1	0.96
P20138	Myeloid cell surface antigen	880.8	975.8	1.11	822.3	0.93	754	0.86	875	0.99	806.8	0.92	915.9	1.04	748.2	0.85
E7EQR8	Protein YIPF3 OS=Homo sa	23740.1	21072.3	0.89	17617.5	0.74	18663	0.79	21307.5	0.90	22466	0.95	21160	0.89	20061.3	0.85
A0A024RE1	Prominin 2, isoform CRA_a	960.6	1009	1.05	945.6	0.98	802.3	0.84	1009.4	1.05	1102.6	1.15	1014.6	1.06	764.5	0.80
P35321	Cornifin-A OS=Homo sapie	378.6	339.1	0.90	372.9	0.98	500.2	1.32	379.1	1.00	361.8	0.96	330.9	0.87	437.9	1.16
P36896	Activin receptor type-1B OS	2014	1670	0.83	1388.4	0.69	1232.7	0.61	1677.5	0.83	1893.2	0.94	1729.5	0.86	1604.6	0.80
Q9H444	Charged multivesicular body	418.7	478.7	1.14	411.2	0.98	382	0.91	419.4	1.00	476.3	1.14	460.7	1.10	348.4	0.83
A6NMH8	Tetraspanin OS=Homo sapi	183.4	203.9	1.11	202.5	1.10	155	0.85	194.5	1.06	184.8	1.01	193.4	1.05	159.1	0.87
Q8WV92	MIT domain-containing pro	314.4	385.2	1.23	284	0.90	348.4	1.11	363.2	1.16	398.2	1.27	463.8	1.48	295.9	0.94
A8K4E0	cDNA FLJ78122 OS=Homo	377.6	401.7	1.06	325.8	0.86	258.6	0.68	369.2	0.98	335.7	0.89	380.5	1.01	323.5	0.86
A0A0X9T0	GCT-A5 heavy chain variab	1288.3	1363.3	1.06	1109.9	0.86	1365	1.06	1454.9	1.13	1610.6	1.25	1234.6	0.96	1571.4	1.22
Q8N7G1	Purine nucleoside phosphory	121.9	184.8	1.52	121.1	0.99	132.2	1.08	134.9	1.11	177.7	1.46	163.1	1.34	132.4	1.09
A8K8G3	cDNA FLJ77237, highly sin	1058.4	1113.3	1.05	939	0.89	913.6	0.86	1008.8	0.95	1122.8	1.06	1067	1.01	898.4	0.85
D6RAR4	Hepatocyte growth factor ac	1403.6	1206.9	0.86	1120	0.80	1146	0.82	1360.3	0.97	1370	0.98	1166.7	0.83	1038.7	0.74
Q9NP29	Microfibrillar protein 2 (Fra	648.7	570.7	0.88	720.9	1.11	475.9	0.73	681.6	1.05	792.3	1.22	561.8	0.87	614.9	0.95
B2R7D2	cDNA, FLJ93389, highly si	181.1	294.3	1.63	201.4	1.11	207	1.14	188.3	1.04	223	1.23	258.5	1.43	184.8	1.02
Q8NC42	E3 ubiquitin-protein ligase R	998.1	1022.1	1.02	916.9	0.92	837.9	0.84	985.8	0.99	942.7	0.94	1003.4	1.01	905	0.91
P27105	Erythrocyte band 7 integral	432	561.6	1.30	422.1	0.98	399.2	0.92	493.6	1.14	496.4	1.15	522.5	1.21	417.1	0.97
P81172	Hepcidin OS=Homo sapiens	2320.3	2372.8	1.02	2930.4	1.26	1814.3	0.78	2823.5	1.22	2868.3	1.24	2697.5	1.16	2796.6	1.21
Q9HBR0	Putative sodium-coupled ne	203.3	217.9	1.07	174.9	0.86	171.3	0.84	211.2	1.04	219.9	1.08	230.7	1.13	158.7	0.78
Q5VW32	BRO1 domain-containing pr	680.8	605.9	0.89	544.8	0.80	530.8	0.78	617	0.91	739.6	1.09	672.3	0.99	546.9	0.80
Q15286	Ras-related protein Rab-35 (															
A0A075B6J	Immunoglobulin lambda var	1136.9	900.5	0.79	1060.4	0.93	755.2	0.66	1166.2	1.03	1020.6	0.90	1022	0.90	1031.3	0.91
Q0ZCF6	Immunglobulin heavy chain															
P10645	Chromogranin-A OS=Homo	1150	1315.1	1.14	1390.3	1.21	847.6	0.74	1127.3	0.98	1153.3	1.00	1322.4	1.15	899.4	0.78
A2IPI6	HRV Fab 027-VL (Fragmen	288.1	275.8	0.96	326.1	1.13	265.7	0.92	311.2	1.08	295.8	1.03	329.5	1.14	338.2	1.17
P01040	Cystatin-A OS=Homo sapie	1303.1	1120.2	0.86	1185.4	0.91	1183.6	0.91	1327.6	1.02	1145	0.88	1292.5	0.99	1320.2	1.01
Q14019	Coactosin-like protein OS=I	2695.4	3304	1.23	3709	1.38	2098.1	0.78	2691.4	1.00	3876.4	1.44	2990.7	1.11	2315.1	0.86
B0YIW2	Apolipoprotein C-III OS=H	535.4	689	1.29	823	1.54	543.1	1.01	534.9	1.00	585.2	1.09	744.4	1.39	578.8	1.08
Q9UKU6	Thyrotropin-releasing horm	353.4	567.4	1.61	457.8	1.30	343.5	0.97	411.3	1.16	412.6	1.17	399.1	1.13	332.2	0.94
A0A158RFU	RAB7, member RAS oncog	305.3	376.1	1.23	256.4	0.84	293.9	0.96	314.5	1.03	335.4	1.10	336.9	1.10	291.1	0.95
Q9GZM7	Tubulointerstitial nephritis a	355.7	416.6	1.17	336	0.94	312.1	0.88	391.7	1.10	357.4	1.00	343.1	0.96	346.4	0.97
P55259	Pancreatic secretory granule	152.2	152.2	1.00	187.9	1.23	136.9	0.90	168	1.10	162.6	1.07	120.5	0.79	134.8	0.89
Q8N289	cDNA FLJ33655 fis, clone	130.7	132.1	1.01	133.3	1.02	127.7	0.98	161	1.23	140.4	1.07	113.5	0.87	112.5	0.86
Q8WW52	Protein FAM151A OS=Hon	851.1	989.6	1.16	689	0.81	567	0.67	737.5	0.87	830.1	0.98	912.5	1.07	609.1	0.72
A0A024R1U	RAB5C, member RAS onco	412.1	473.8	1.15	354.3	0.86	363.6	0.88	469.5	1.14	529.5	1.28	408.8	0.99	345.6	0.84
A0A090KFV	HLA class I antigen OS=Ho	138.3	171.7	1.24	135.1	0.98	103.3	0.75	147.5	1.07	142.7	1.03	137.3	0.99	120.9	0.87
P20062	Transcobalamin-2 OS=Hom	241.5	313.5	1.30	235.1	0.97	220.3	0.91	266.1	1.10	274.3	1.14	299.1	1.24	246.5	1.02

Q5IWS5	Intelectin 1 OS=Homo sapie	793.9	662.1	0.83	403	0.51	407.9	0.51	509.9	0.64	416.2	0.52	593.9	0.75	316.1	0.40
P15121	Aldose reductase OS=Homo	209.9	178.4	0.85	196.8	0.94	143.8	0.69	186	0.89	255.8	1.22	232.8	1.11	304.2	1.45
O43692	Peptidase inhibitor 15 OS=H	2345.7	2388.7	1.02	1711.9	0.73	1882.8	0.80	2695.6	1.15	2144.6	0.91	2346.3	1.00	2147.8	0.92
O95460	Matrilin-4 OS=Homo sapien	789.3	769.4	0.97	532.9	0.68	555	0.70	706.2	0.89	654.5	0.83	616.9	0.78	491.1	0.62
B4E1U9	cDNA FLJ54776, highly sin	373.9	502.9	1.35	394.2	1.05	338.7	0.91	400.9	1.07	534.3	1.43	441.2	1.18	423.8	1.13
V9HW62	Lactoylglutathione lyase OS	1116.1	1304.7	1.17	1214.5	1.09	901.8	0.81	1117.3	1.00	1269.3	1.14	1140.9	1.02	975.5	0.87
Q2UY09	Collagen alpha-1(XXVIII) c	1588.2	1460.8	0.92	1358.3	0.86	988.9	0.62	1457.2	0.92	1426.5	0.90	1433.7	0.90	1260.7	0.79
Q06481	Amyloid-like protein 2 OS=	659.8	922.6	1.40	901.1	1.37	712.4	1.08	887.7	1.35	820.9	1.24	885.7	1.34	697.6	1.06
Q15746	Myosin light chain kinase, s	1162.6	1140.3	0.98	958.7	0.82	861.9	0.74	1077.8	0.93	1058.3	0.91	1254.5	1.08	1083.5	0.93
P21399	Cytoplasmic aconitate hydra	281.1	385.3	1.37	309.4	1.10	258.9	0.92	321.4	1.14	372.5	1.33	330	1.17	271.2	0.96
P02792	Ferritin light chain OS=Hon	100.4	159.7	1.59	121.1	1.21	113.5	1.13	119.3	1.19	109.9	1.09	109.6	1.09	129.7	1.29
P04899	Guanine nucleotide-binding	147.3	172.2	1.17	160.6	1.09	149.7	1.02	205	1.39	221.7	1.51	157.2	1.07	160.8	1.09
V9HW91	Epididymis secretory sperm	65.2	119.5	1.83	65	1.00	66.2	1.02	66.4	1.02	86.5	1.33	90.5	1.39	57.3	0.88
A8K037	cDNA FLJ77641 OS=Homo	651.7	774.5	1.19	651.3	1.00	508.3	0.78	670.9	1.03	654.8	1.00	785	1.20	593.8	0.91
A6NC48	ADP-ribosyl cyclase/cyclic	286.2	279	0.97	248.2	0.87	202.9	0.71	261.6	0.91	249.8	0.87	246.8	0.86	230.9	0.81
Q53GE4	Leucine rich repeat containi	228.6	266.2	1.16	221.6	0.97	206.3	0.90	239.9	1.05	239	1.05	252.9	1.11	195.6	0.86
B4DRY1	cDNA FLJ54428, highly sin	306	347.9	1.14	348.9	1.14	265.1	0.87	318.1	1.04	347.4	1.14	332.6	1.09	311.7	1.02
P50053	Ketohexokinase OS=Homo	379	505.3	1.33	381.4	1.01	294.5	0.78	366.7	0.97	496.3	1.31	422.9	1.12	352.5	0.93
A0A024R7I	Solute carrier family 44, me	864.2	989.2	1.14	1000.2	1.16	753.4	0.87	1022.9	1.18	1044.3	1.21	840.7	0.97	773.4	0.89
A0A024R8S	Protein disulfide-isomerase	223.5	282.9	1.27	214.3	0.96	206.7	0.92	228.6	1.02	215.5	0.96	232.5	1.04	214	0.96
K7N7A8	Uncharacterized protein (Fra	12.6	13.6	1.08	13.8	1.10	15.8	1.25	16.1	1.28	18.9	1.50	15.1	1.20	14.2	1.13
A0A109PP8	MS-C2 light chain variable	644.8	675	1.05	642.2	1.00	563	0.87	554.6	0.86	610.9	0.95	787.8	1.22	625.4	0.97
A0A087WW	Trypsin-1 OS=Homo sapien	683	934.1	1.37	517	0.76	804.9	1.18	753	1.10	588.5	0.86	698.1	1.02	612.1	0.90
B7ZLM6	X-prolyl aminopeptidase (A	399.9	475.6	1.19	392	0.98	298.2	0.75	425.1	1.06	434.6	1.09	393.2	0.98	281.5	0.70
Q96DR8	Mucin-like protein 1 OS=Ho	7477.2	7509.4	1.00	8039.1	1.08	6954.1	0.93	8901.6	1.19	8034.1	1.07	4241.8	0.57	5934.1	0.79
P36639	7,8-dihydro-8-oxoguanine tr	46.1	46.7	1.01	35.7	0.77	27.4	0.59	37.7	0.82	45	0.98	45.8	0.99	39.6	0.86
A9UFC0	Caspase 14 OS=Homo sapie	272.4	215.4	0.79	233.5	0.86	178.4	0.65	257.8	0.95	218.9	0.80	253.9	0.93	274	1.01
Q15952	Agrin (Fragment) OS=Homo															
V9HW48	SH3 domain-binding glutam	56.2	89	1.58	114.7	2.04	92.2	1.64	70.4	1.25	60.5	1.08	68	1.21	75.6	1.35
P02743	Serum amyloid P-componen	1124.2	912.8	0.81	727.3	0.65	697.9	0.62	704.4	0.63	811.7	0.72	880.2	0.78	977.9	0.87
Q68D85	Natural cytotoxicity triggeri	202.5	193.7	0.96	177	0.87	164.2	0.81	180.1	0.89	188.2	0.93	189.8	0.94	183.7	0.91
D3DRR6	Inter-alpha (Globulin) inhibi	249.7	446.9	1.79	336	1.35	459.8	1.84	351.5	1.41	296.5	1.19	432.7	1.73	327.5	1.31
Q59FG9	Chondroitin sulfate proteogl	517.4	499.4	0.97	334.5	0.65	388.2	0.75	461.9	0.89	497.8	0.96	485.2	0.94	436.1	0.84
P48960	CD97 antigen OS=Homo sa	338	338.7	1.00	308.5	0.91	258.8	0.77	295.4	0.87	365.7	1.08	418.6	1.24	266.7	0.79
O43291	Kunitz-type protease inhibit	935.7	788.1	0.84	788.7	0.84	558.5	0.60	759.9	0.81	858.5	0.92	751.6	0.80	781.3	0.83
A8K2X4	cDNA FLJ75401, highly sin	236.8	254.2	1.07	221.6	0.94	278.7	1.18	245.1	1.04	246.7	1.04	241.7	1.02	214.8	0.91
P21796	Voltage-dependent anion-se	204.8	291.5	1.42	253.3	1.24	237.4	1.16	276.9	1.35	231.4	1.13	286.4	1.40	266.2	1.30
A0A140T8X	Lysosomal thioesterase PPT	225.9	282.1	1.25	214.6	0.95	187.1	0.83	265.8	1.18	266.3	1.18	310.6	1.37	229.1	1.01
B2R778	cDNA, FLJ93317, highly si	323.7	338.4	1.05	255.6	0.79	273.4	0.84	265.3	0.82	293.1	0.91	386.7	1.19	219.8	0.68
B3KWI4	cDNA FLJ43122 fis, clone	120.9	186.2	1.54	141.6	1.17	156.4	1.29	150.5	1.24	144.1	1.19	154.9	1.28	137.6	1.14
A0A140VK	Aspartate aminotransferase	402.1	572.1	1.42	435.2	1.08	451.3	1.12	490.8	1.22	626.6	1.56	470.5	1.17	402	1.00
O60635	Tetraspanin-1 OS=Homo sa	121.1	181.3	1.50	116.9	0.97	104.5	0.86	126.7	1.05	132.7	1.10	135.5	1.12	103	0.85
Q5T2L0	V-set domain-containing T-	960.5	1386	1.44	624.6	0.65	758.7	0.79	844	0.88	832	0.87	907.4	0.94	811.8	0.85
P24158	Myeloblastin OS=Homo sap	289.9	615.9	2.12	413.6	1.43	295.9	1.02	368.9	1.27	390.2	1.35	623	2.15	677.4	2.34
P68371	Tubulin beta-4B chain OS=I	23.6	26.1	1.11	18.2	0.77	35.9	1.52	20.9	0.89	20.3	0.86	22.9	0.97	26.6	1.13
A0A075B6I	Immunoglobulin lambda var	2023.3	1770.1	0.87	2483	1.23	2168.7	1.07	2079.2	1.03	1873.2	0.93	3276.7	1.62	2795	1.38
P53801	Pituitary tumor-transforming	688.6	719.7	1.05	593.6	0.86	498.6	0.72	672.6	0.98	683.5	0.99	692.9	1.01	584.8	0.85
B2R907	cDNA, FLJ94149, highly si	346.7	280.5	0.81	261.4	0.75	223.3	0.64	247.2	0.71	299.8	0.86	352.9	1.02	286.9	0.83
A0A024R57	EH domain-containing prote	231	230.6	1.00	191.1	0.83	140	0.61	221	0.96	275.8	1.19	256.4	1.11	187.4	0.81
Q99536	Synaptic vesicle membrane	130.3	147.4	1.13	135.9	1.04	133.5	1.02	135.5	1.04	144	1.11	146.5	1.12	129.6	0.99
E7EUF1	Ectonucleotide pyrophospha	343.1	382.5	1.11	334.8	0.98	317.6	0.93	321.1	0.94	371.3	1.08	344.8	1.00	278.6	0.81
P20933	N(4)-(beta-N-acetylglucosar	404.3	461.4	1.14	372.8	0.92	418.8	1.04	441.1	1.09	415.2	1.03	405.2	1.00	402.6	1.00
A0A0S2Z3N	Dopa decarboxylase isoforn	494.9	623.3	1.26	493	1.00	418.8	0.85	507	1.02	614.7	1.24	582.5	1.18	496.5	1.00
Q6UX15	Layilin OS=Homo sapiens (	442.6	465.8	1.05	467.6	1.06	353	0.80	491.7	1.11	476.1	1.08	528	1.19	409.6	0.93
B4DKN3	cDNA FLJ50980 OS=Homo	210.3	248	1.18	225.7	1.07	183.8	0.87	210.3	1.00	240.4	1.14	248	1.18	204.9	0.97
A0A0A0MT	UPF0764 protein C16orf89	879.9	947.7	1.08	795.9	0.90	793.3	0.90	743.5	0.84	1208.5	1.37	899	1.02	733.4	0.83
Q66K79	Carboxypeptidase Z OS=Ho	2103.6	2090.1	0.99	2060.2	0.98	1403.2	0.67	2560.9	1.22	2240.2	1.06	2475.9	1.18	2134.7	1.01
B4DR52	Histone H2B OS=Homo sap	992.5	1928	1.94	1482.9	1.49	826.5	0.83	1087.7	1.10	1195	1.20	1902.3	1.92	2601.2	2.62

B3KQS9	cDNA PSEC0141 fis, clone	773.7	918.8	1.19	688.6	0.89	633	0.82	750.2	0.97	900.2	1.16	788.1	1.02	835.3	1.08
Q08257	Quinone oxidoreductase OS	319.8	477.5	1.49	325.6	1.02	294.8	0.92	327	1.02	454.5	1.42	355.2	1.11	293.3	0.92
Q59G10	Aldehyde dehydrogenase 1	170.9	215.2	1.26	158.9	0.93	151.1	0.88	161	0.94	201.3	1.18	219.5	1.28	148.9	0.87
B4DLV7	Rab GDP dissociation inhib	472.6	621.6	1.32	512.2	1.08	420.2	0.89	524.1	1.11	562	1.19	568.7	1.20	475.1	1.01
P30043	Flavin reductase (NADPH)	143.4	203.2	1.42	171.6	1.20	166.2	1.16	173	1.21	162.3	1.13	185.4	1.29	150.6	1.05
Q400G7	B and T lymphocyte attenua	106.5	92.5	0.87	71	0.67	80.2	0.75	75.5	0.71	90.5	0.85	92.8	0.87	91.7	0.86
Q9Y3B3	Transmembrane emp24 dom	446	430	0.96	442	0.99	374.7	0.84	411	0.92	450.4	1.01	400	0.90	435.7	0.98
P59665	Neutrophil defensin 1 OS=H	1746.8	2669.7	1.53	2632.3	1.51	1359.4	0.78	2256.9	1.29	2139	1.22	4014.5	2.30	4344.9	2.49
A0A0R7FJH	Coagulation factor XII OS=	83.3	132	1.58	114.1	1.37	91.6	1.10	96.1	1.15	89.6	1.08	107.7	1.29	95.5	1.15
B2RBC8	cDNA, FLJ95444, highly sir	875.9	762.3	0.87	686.1	0.78	530.6	0.61	751.3	0.86	847.2	0.97	764.2	0.87	717	0.82
P16035	Metalloproteinase inhibitor	890.7	808.9	0.91	682	0.77	925.9	1.04	901.6	1.01	865.5	0.97	873.9	0.98	706.7	0.79
P61204	ADP-ribosylation factor 3 C	528.6	671.3	1.27	534.7	1.01	486.1	0.92	627.4	1.19	643.2	1.22	608.7	1.15	613.1	1.16
A8K6C1	cDNA FLJ76868, highly sin	461.4	508.1	1.10	509	1.10	420.8	0.91	490	1.06	501.5	1.09	492.2	1.07	482.6	1.05
V9HWH9	Protein S100 OS=Homo sap	1224.2	1275.4	1.04	1048.9	0.86	979.6	0.80	1149.1	0.94	1119	0.91	1259.7	1.03	1780.4	1.45
A0A1B0RP(	Fibroblast growth factor rec	493.9	546.6	1.11	460.8	0.93	379.5	0.77	479.6	0.97	487.4	0.99	484.2	0.98	446.5	0.90
O00182	Galectin-9 OS=Homo sapier	878.9	1045.3	1.19	676.1	0.77	737.8	0.84	930.6	1.06	1037.1	1.18	1067.8	1.21	872.9	0.99
B2R7S7	cDNA, FLJ93585, highly sir	165.1	199.8	1.21	176	1.07	131.7	0.80	186.5	1.13	198.7	1.20	197.4	1.20	149.8	0.91
Q9NZT1	Calmodulin-like protein 5 O	169.4	150.4	0.89	119.4	0.70	135.3	0.80	141.9	0.84	147.2	0.87	146.9	0.87	187.8	1.11
A0A0G2JPH	Leukocyte-associated immu	339.7	358.4	1.06	324.8	0.96	293.2	0.86	266.2	0.78	374.8	1.10	307.5	0.91	300.5	0.88
P36871	Phosphoglucomutase-1 OS=	380.9	459.6	1.21	364.2	0.96	431.6	1.13	387.2	1.02	432.3	1.13	432.8	1.14	369	0.97
A2N7P4	Immunoglobulin mu-chain D															
Q96SB0	Anti-streptococcal/anti-myo	94.1	103.3	1.10	105.1	1.12	83.6	0.89	124.1	1.32	95.9	1.02	159.3	1.69	128.6	1.37
A0A024R9N	EH-domain containing 4, isc	74.6	83.8	1.12	62.1	0.83	80.9	1.08	65.4	0.88	73.5	0.99	75.6	1.01	66.8	0.90
X5DR95	Semaphorin 5A isoform A (	177	163.8	0.93	133.1	0.75	125.9	0.71	174.9	0.99	182.3	1.03	167.5	0.95	128.8	0.73
Q99816	Tumor susceptibility gene 1(	390.7	528.8	1.35	361.9	0.93	382.6	0.98	413.9	1.06	458.6	1.17	540.4	1.38	350.4	0.90
P20333	Tumor necrosis factor recep	706.7	737.1	1.04	596.5	0.84	516.7	0.73	657.8	0.93	571.3	0.81	1127.5	1.60	680.3	0.96
Q5U043	S-(hydroxymethyl)glutathio	183	298	1.63	217.4	1.19	185.4	1.01	220.4	1.20	254.7	1.39	214.3	1.17	197.9	1.08
Q5JRA6	Melanoma inhibitory activit	297.4	285	0.96	309.2	1.04	227.4	0.76	296.8	1.00	275.4	0.93	256.7	0.86	276.2	0.93
I3L4C2	Brain-specific angiogenesis	77.2	79.9	1.03	66.5	0.86	81.2	1.05	67.8	0.88	88.2	1.14	69.2	0.90	63.5	0.82
Q9BRA2	Thioredoxin domain-contair	785.9	875.4	1.11	691.2	0.88	566.4	0.72	824.8	1.05	928.7	1.18	889.1	1.13	769.6	0.98
Q9UL89	Myosin-reactive immunoglo	84.9	104.1	1.23	79.9	0.94	126.5	1.49	115.9	1.37	75.1	0.88	109.9	1.29	102.6	1.21
A0A0A6YY	Trefoil factor 3 OS=Homo s	4753.6	5290.9	1.11	6041.5	1.27	3228.5	0.68	5198.1	1.09	5099.7	1.07	4859.1	1.02	5565.3	1.17
A8K556	cDNA FLJ78217 OS=Homo	66.2	58.7	0.89	55.2	0.83	41.1	0.62	54.3	0.82	68.5	1.03	61.2	0.92	78.9	1.19
P41181	Aquaporin-2 OS=Homo sap	738.1	769.4	1.04	814.4	1.10	541.7	0.73	802.1	1.09	943.5	1.28	759	1.03	695.1	0.94
A0A024R7N	Interferon, gamma-inducible	330.6	402.6	1.22	352.3	1.07	289	0.87	434.6	1.31	329	1.00	447.9	1.35	352.1	1.07
Q13508	Ecto-ADP-ribosyltransferas	3963.6	3432.9	0.87	3392.8	0.86	2121.5	0.54	3256.2	0.82	3934.9	0.99	3277	0.83	2456.4	0.62
Q8N4F0	BPI fold-containing family	975	698.4	0.72	482	0.49	566.6	0.58	533.5	0.55	911.4	0.93	549	0.56	538.1	0.55
A1L4F5	Receptor tyrosine kinase-lik	539.3	674	1.25	486.7	0.90	482.7	0.90	589.5	1.09	542.8	1.01	616.9	1.14	539.6	1.00
Q7Z5F5	Liver-expressed antimicrobi	2352.3	2169.4	0.92	2019.6	0.86	1508.1	0.64	2123.8	0.90	2118.7	0.90	2721.5	1.16	2031.8	0.86
Q6MZK8	Putative uncharacterized pro	1055.5	855.3	0.81	753.2	0.71	602.8	0.57	824.9	0.78	1052.1	1.00	815.5	0.77	884.4	0.84
A0A0G2JNI	Leukocyte immunoglobulin-	506.9	727.8	1.44	569.3	1.12	471.4	0.93	639.9	1.26	650.6	1.28	758.7	1.50	604.4	1.19
Q9UK41	Vacuolar protein sorting-ass	125.4	168.4	1.34	135.1	1.08	131.4	1.05	135.1	1.08	139.5	1.11	134.7	1.07	115.1	0.92
B1AKK2	Dimethylarginine dimethyla	237.8	361.4	1.52	224.5	0.94	236.1	0.99	227.3	0.96	316.5	1.33	262.5	1.10	254.1	1.07
Q13201	Multimerin-1 OS=Homo sap	3084.4	2564.2	0.83	2383.5	0.77	1835.3	0.60	2261.2	0.73	2741.7	0.89	3078.9	1.00	2514.8	0.82
Q6PCB0	von Willebrand factor A doi	360.7	686.2	1.90	405.7	1.12	301.9	0.84	381.8	1.06	389	1.08	484.1	1.34	312.9	0.87
Q15904	V-type proton ATPase subu	385.4	417.9	1.08	529.1	1.37	297.8	0.77	374.5	0.97	464	1.20	308.2	0.80	356.3	0.92
H0Y8X4	2'-deoxynucleoside 5'-phosp	281.2	414.4	1.47	324	1.15	305.7	1.09	351.5	1.25	377.2	1.34	305	1.08	272.2	0.97
P07585	Decorin OS=Homo sapiens	336.7	405	1.20	330.1	0.98	330.8	0.98	366	1.09	354.2	1.05	363.2	1.08	359.3	1.07
B3KPR2	cDNA FLJ32087 fis, clone (	426.5	481.3	1.13	551	1.29	426.2	1.00	475.2	1.11	421.6	0.99	462.8	1.09	373	0.87
Q03405	Urokinase plasminogen acti	107.7	127.2	1.18	158.8	1.47	115.4	1.07	127.4	1.18	117	1.09	122	1.13	109.9	1.02
Q32Q12	Nucleoside diphosphate kin	380.3	543	1.43	451.4	1.19	400.9	1.05	497.6	1.31	535.8	1.41	501.1	1.32	491.2	1.29
Q9ULC0	Endomucin OS=Homo sapi	772.7	808	1.05	759.5	0.98	456.2	0.59	869.5	1.13	842.1	1.09	601.8	0.78	867.5	1.12
O00322	Uroplakin-1a OS=Homo sap	506.4	488.1	0.96	477.2	0.94	370.2	0.73	501.9	0.99	484.3	0.96	500.6	0.99	421.2	0.83
P24593	Insulin-like growth factor-bi	422.2	542.3	1.28	491	1.16	409.2	0.97	491.8	1.16	463.7	1.10	516	1.22	480.8	1.14
B1B0D4	ADAMTS-like protein 2 OS	111.6	116.7	1.05	99.4	0.89	82.5	0.74	111.7	1.00	111.1	1.00	113	1.01	105.9	0.95
Q13308	Inactive tyrosine-protein kin	238	280.4	1.18	218.1	0.92	203.3	0.85	250	1.05	240.6	1.01	192.5	0.81	222.6	0.94
O43570	Carbonic anhydrase 12 OS=	12.2	13.3	1.09	11.4	0.93	8.7	0.71	13.7	1.12	16.3	1.34	12.6	1.03	10.4	0.85
A0A0X9UW	IBM-A3 heavy chain variab	45	93.6	2.08	64.4	1.43	53	1.18	61	1.36	55.5	1.23	64.3	1.43	52	1.16

Q9NQ38	Serine protease inhibitor Ka	2121.7	1745.1	0.82	2618.2	1.23	1196.8	0.56	2027.1	0.96	2058.5	0.97	1807.5	0.85	1876.7	0.88
Q9NY25	C-type lectin domain family	366.3	351.3	0.96	284.6	0.78	275	0.75	294.5	0.80	332.6	0.91	334.2	0.91	329	0.90
Q15223	Nectin-1 OS=Homo sapiens	980.7	923.9	0.94	798	0.81	666.1	0.68	1008.6	1.03	951.5	0.97	945.8	0.96	900.1	0.92
Q53RD9	Fibulin-7 OS=Homo sapiens	722	669.2	0.93	772.3	1.07	512.5	0.71	663.2	0.92	655.9	0.91	708.5	0.98	603.7	0.84
Q16706	Alpha-mannosidase 2 OS=H	283.9	313.3	1.10	279.2	0.98	211.3	0.74	288.8	1.02	318.6	1.12	301.1	1.06	276	0.97
P48551	Interferon alpha/beta recepto	1228.8	969.7	0.79	966.2	0.79	532.1	0.43	832.4	0.68	832.9	0.68	938.9	0.76	1155.2	0.94
A0A068LKC	Ig heavy chain variable regio	241.8	223.1	0.92	168	0.69	183.1	0.76	214.1	0.89	275.5	1.14	194.4	0.80	225.9	0.93
Q14314	Fibroleukin OS=Homo sapie	676.9	907	1.34	602.9	0.89	714.1	1.05	759	1.12	756.1	1.12	883.9	1.31	611.3	0.90
P36405	ADP-ribosylation factor-like	74.8	89.8	1.20	76.7	1.03	74.6	1.00	82.7	1.11	84.3	1.13	76.4	1.02	73.4	0.98
O15240	Neurosecretory protein VGF	1220.8	1246.6	1.02	1077.7	0.88	1069.5	0.88	1201.3	0.98	1129.4	0.93	1204.7	0.99	944.5	0.77
J3KS40	Secreted and transmembrane	105.4	105.1	1.00	109.4	1.04	88.8	0.84	105.4	1.00	121.7	1.15	113.9	1.08	86.2	0.82
A0A024R6L	Delta-like 1 homolog (Dros	7066.9	5585.3	0.79	4701.3	0.67	3555.6	0.50	5313.7	0.75	5481.2	0.78	4643.6	0.66	4609.3	0.65
Q05DB4	HEBP2 protein (Fragment)	131.4	166.8	1.27	129.9	0.99	121.3	0.92	133	1.01	147.9	1.13	146.2	1.11	137.4	1.05
B2R9S4	cDNA, FLJ94534, highly si	345.9	429.5	1.24	374.1	1.08	304.8	0.88	409.4	1.18	418.8	1.21	414.3	1.20	386.5	1.12
Q9UNF0	Protein kinase C and casein	87.7	113.8	1.30	88.1	1.00	82	0.94	86.2	0.98	96.6	1.10	86.8	0.99	88.5	1.01
Q92626	Peroxidasin homolog OS=H	473.6	502.6	1.06	374.6	0.79	450.9	0.95	519.3	1.10	480.2	1.01	484.8	1.02	411.6	0.87
A7U7M2	Prion protein 2 (Dublet) OS	541.8	491.1	0.91	387.7	0.72	394.6	0.73	557.2	1.03	463.6	0.86	502.4	0.93	445.8	0.82
A0A024R32	Ras homolog gene family, m	180.3	241.7	1.34	211.6	1.17	151.2	0.84	216.2	1.20	200.1	1.11	211.9	1.18	171.5	0.95
A0A140VJH	Testicular tissue protein Li 4	1629	1606.4	0.99	1615.9	0.99	1322.8	0.81	1825.1	1.12	2007.2	1.23	1269.3	0.78	1273.9	0.78
B4DNE1	cDNA FLJ52708, highly sin	1315.6	1232.1	0.94	1051.4	0.80	965.8	0.73	1151.8	0.88	1223.4	0.93	1385.8	1.05	1101.1	0.84
P40199	Carcinoembryonic antigen-r	325	349.8	1.08	353	1.09	250.2	0.77	367	1.13	327.1	1.01	365.5	1.12	316.6	0.97
Q00796	Sorbitol dehydrogenase OS=	446.2	686.6	1.54	565.3	1.27	402	0.90	526.4	1.18	544.3	1.22	482.9	1.08	356.4	0.80
Q16610	Extracellular matrix protein	111.2	138.6	1.25	99.2	0.89	111.4	1.00	123.6	1.11	109.2	0.98	115.1	1.04	108.8	0.98
A0A193CHI	10E8 heavy chain variable r	299.2	323	1.08	277.2	0.93	277.4	0.93	324.6	1.08	344.5	1.15	356.2	1.19	334.3	1.12
B2R597	cDNA, FLJ92390, highly si	12181.9	9325.5	0.77	9386.4	0.77	7418.6	0.61	9548.3	0.78	9352.6	0.77	2737.9	0.22	4333.9	0.36
P20151	Kallikrein-2 OS=Homo sapi															
Q9Y376	Calcium-binding protein 39	197	241	1.22	199.4	1.01	144.7	0.73	218.1	1.11	224.5	1.14	205.7	1.04	147.1	0.75
A0A140VJK	Testicular tissue protein Li 8	138.2	169.6	1.23	136.7	0.99	123.7	0.90	148.1	1.07	149.7	1.08	139.7	1.01	130.9	0.95
Q0ZCH6	Immunglobulin heavy chain	522	587.5	1.13	508.3	0.97	538.1	1.03	771.4	1.48	534.1	1.02	589.1	1.13	544.5	1.04
Q9BXN2	C-type lectin domain family	420.9	416.2	0.99	382.7	0.91	326.9	0.78	393.8	0.94	385	0.91	526.4	1.25	402.3	0.96
Q8TCD5	5'(3')-deoxyribonucleotidase	528.8	586.3	1.11	496.2	0.94	436	0.82	541.1	1.02	570.5	1.08	585.2	1.11	467.6	0.88
B4DMS4	cDNA FLJ59350 OS=Homo	369.1	558.8	1.51	527	1.43	395.8	1.07	590.7	1.60	608.2	1.65	594.4	1.61	495.7	1.34
Q9NP79	Vacuolar protein sorting-ass	37.1	46.8	1.26	30.5	0.82	34.5	0.93	36.9	0.99	42.8	1.15	39.2	1.06	46.4	1.25
Q9UGM3	Deleted in malignant brain t	214.2	195.1	0.91	201.8	0.94	178.1	0.83	171.8	0.80	160.3	0.75	202.5	0.95	200.3	0.94
A8K0T9	cDNA FLJ75422, highly sin	238.9	370.4	1.55	374.5	1.57	216.2	0.90	349	1.46	362.7	1.52	369.4	1.55	279.8	1.17
P23468	Receptor-type tyrosine-prote	48.4	54.4	1.12	39.6	0.82	39.9	0.82	32.9	0.68	42.9	0.89	82.3	1.70	31	0.64
P24592	Insulin-like growth factor-bi	785	865.4	1.10	908.8	1.16	760.7	0.97	865	1.10	812.3	1.03	844.8	1.08	829.2	1.06
P20827	Ephrin-A1 OS=Homo sapie	1456.4	1424.5	0.98	1376.3	0.95	976.8	0.67	1436	0.99	1513.2	1.04	1470.8	1.01	1186.4	0.81
Q9UIU0	Dihydropyridine receptor al	439	442.2	1.01	448.9	1.02	434.8	0.99	458.6	1.04	534.3	1.22	450.8	1.03	378.3	0.86
P01210	Proenkephalin-A OS=Homo	752.9	888.3	1.18	775	1.03	1029.9	1.37	853.9	1.13	785.3	1.04	764.7	1.02	703.1	0.93
P06703	Protein S100-A6 OS=Homo	2629.1	2621.2	1.00	2536.2	0.96	1732.2	0.66	2589.1	0.98	3375.7	1.28	3079.8	1.17	3340.9	1.27
A0A024RB0	Tetraspanin OS=Homo sapi	1265.1	1531.3	1.21	1467.4	1.16	1195.2	0.94	1469.9	1.16	1382.3	1.09	1209.3	0.96	1181.8	0.93
Q9H0B8	Cysteine-rich secretory prot	601.1	627.2	1.04	623.6	1.04	559.7	0.93	583.5	0.97	584.2	0.97	467.9	0.78	479.4	0.80
P08729	Keratin, type II cytoskeletal	78.3	137.6	1.76	73.7	0.94	60.5	0.77	69.4	0.89	74.3	0.95	109.6	1.40	151.1	1.93
P25815	Protein S100-P OS=Homo s	900.9	1048.1	1.16	841.3	0.93	548.2	0.61	869.6	0.97	1204.9	1.34	1073.2	1.19	1676.1	1.86
P14625	Endoplasmin OS=Homo sap															
A0A024R13	Glycerol-3-phosphate dehyd	437.7	871.9	1.99	478.6	1.09	510.2	1.17	500.7	1.14	553.8	1.27	512.5	1.17	409.5	0.94
B1PS43	Myosin heavy chain 11 smo	156.6	277.4	1.77	174.6	1.11	171.4	1.09	191.6	1.22	185.1	1.18	211.5	1.35	207.8	1.33
A0A087WY	Seizure 6-like protein 2 OS=	107.6	178	1.65	98.7	0.92	99.9	0.93	105.3	0.98	110.5	1.03	109.3	1.02	104.6	0.97
P01709	Immunoglobulin lambda var	1720.5	1762.5	1.02	2050.9	1.19	1622.8	0.94	1830.7	1.06	1804.7	1.05	1761.2	1.02	1809	1.05
B2R983	cDNA, FLJ94267, highly si	356.1	497.6	1.40	350.4	0.98	404.1	1.13	417.9	1.17	450	1.26	461.9	1.30	372.9	1.05
A0A1C3PHI	MHC class I antigen OS=Ho															
A0A024RD	Scavenger receptor class B,	254.2	268.1	1.05	209.6	0.82	200.4	0.79	251.5	0.99	248.5	0.98	257	1.01	241.1	0.95
P13640	Metallothionein-1G OS=Ho	559.1	642.5	1.15	672.2	1.20	426.7	0.76	749.1	1.34	744.3	1.33	866.3	1.55	768.9	1.38
B4DJQ5	cDNA FLJ59211, highly sin	436.2	503.5	1.15	471.9	1.08	357	0.82	544.3	1.25	507.3	1.16	479.7	1.10	412	0.94
Q16661	Guanylate cyclase activator	58.1	77.1	1.33	255.4	4.40	55.6	0.96	74.8	1.29	74.3	1.28	83	1.43	64.4	1.11
Q96C23	Aldose 1-epimerase OS=Ho	33.6	40.1	1.19	37.1	1.10	34.5	1.03	45.4	1.35	42.7	1.27	51.1	1.52	39.9	1.19
J3KNB4	Cathelicidin antimicrobial pe	767.1	824.5	1.07	776.3	1.01	687.1	0.90	854.3	1.11	778.5	1.01	945.5	1.23	955.3	1.25

Q86UD1	Out at first protein homolog	662.9	741.8	1.12	579.9	0.87	648.7	0.98	639.2	0.96	774.1	1.17	816.5	1.23	652.4	0.98
J3KPA1	Cysteine-rich secretory prot	1153.4	846.6	0.73	678.2	0.59	686.2	0.59	815.2	0.71	1100.2	0.95	768.3	0.67	908.6	0.79
A0A1C9J6Q	B cell receptor kappa chain	475.4	562	1.18	568.8	1.20	540.6	1.14	476.3	1.00	525	1.10	524.9	1.10	483.8	1.02
G3XAI2	Laminin subunit beta-1 OS=	400.8	451.9	1.13	413.4	1.03	392.9	0.98	399.7	1.00	394.8	0.99	492.2	1.23	361	0.90
Q9H6B4	CXADR-like membrane pro	1006.1	885.2	0.88	1107.5	1.10	697.3	0.69	940.9	0.94	968.6	0.96	931.8	0.93	1076.6	1.07
M0R1T5	Charged multivesicular body	898.2	980.7	1.09	828.5	0.92	748.1	0.83	1024	1.14	1025.2	1.14	991.9	1.10	771.8	0.86
B2RD36	cDNA, FLJ96437 OS=Hom	855.7	1492.3	1.74	900.7	1.05	954.5	1.12	1036.2	1.21	1323.4	1.55	1136.8	1.33	869.9	1.02
A8K2T6	cDNA FLJ76013, highly sin	665.8	703.3	1.06	624	0.94	587.6	0.88	715.9	1.08	667.7	1.00	722.9	1.09	635.5	0.95
V9HW90	Epididymis luminal protein	185.3	295.1	1.59	211.7	1.14	192.2	1.04	232.4	1.25	238.6	1.29	224.1	1.21	218.7	1.18
A2N4P8	IL2R protein (Fragment) OS	217.5	253.3	1.16	187.2	0.86	203.7	0.94	229	1.05	207.5	0.95	218.4	1.00	244	1.12
A2NW97	Rheumatoid factor Vh I regi	395.2	438.5	1.11	385.4	0.98	497.1	1.26	397.1	1.00	387.4	0.98	383.6	0.97	441.3	1.12
H7C2F2	CD99 antigen (Fragment) O	165.1	154.8	0.94	175.9	1.07	131.9	0.80	149.6	0.91	136.9	0.83	168.1	1.02	129.4	0.78
V9GYM3	Apolipoprotein A-II OS=Ho	315.6	521.7	1.65	362.2	1.15	408.4	1.29	414.5	1.31	356.9	1.13	635.1	2.01	438.5	1.39
B2R7Z2	cDNA, FLJ93669, highly sin	286.3	183.6	0.64	453.3	1.58	131	0.46	307.3	1.07	240.1	0.84	294.5	1.03	324.2	1.13
P12724	Eosinophil cationic protein	77.9	120.6	1.55	114.8	1.47	119.5	1.53	103	1.32	122.3	1.57	103.4	1.33	136.5	1.75
A0A0A0MQ	Laminin subunit alpha-4 OS	554.8	576.6	1.04	592.8	1.07	437.2	0.79	740.9	1.34	557	1.00	567.9	1.02	490.8	0.88
O00526	Uroplakin-2 OS=Homo sapi	169.9	148	0.87	145	0.85	112	0.66	145.4	0.86	154.1	0.91	151.4	0.89	111.1	0.65
A0A0C4DH	Immunoglobulin kappa varia	320.2	330.6	1.03	334	1.04	352.9	1.10	300.3	0.94	293.5	0.92	359.9	1.12	314.8	0.98
A0N7J6	REV25-2 (Fragment) OS=H	2623.9	2349.9	0.90	3333.3	1.27	1832.2	0.70	2334.3	0.89	2874	1.10	2715.3	1.03	3016.2	1.15
Q8N3J6	Cell adhesion molecule 2 OS	226.2	220.8	0.98	222.4	0.98	135.6	0.60	198.5	0.88	202.8	0.90	203.5	0.90	193.6	0.86
P27482	Calmodulin-like protein 3 O	76.5	84.2	1.10	86.7	1.13	68.6	0.90	92.8	1.21	81.6	1.07	95.7	1.25	76.1	0.99
P04732	Metallothionein-1E OS=Ho	191.4	254	1.33	251.7	1.32	158	0.83	264	1.38	223.3	1.17	246.3	1.29	267.3	1.40
Q53FE8	cDNA FLJ36526 fis, clone	1131.9	1238.4	1.09	960.9	0.85	1061.7	0.94	1097.3	0.97	1221.9	1.08	1089.9	0.96	1157.5	1.02
B2RA03	cDNA, FLJ94640, highly sin	40.2	55.1	1.37	40.8	1.01	37.9	0.94	44.4	1.10	46.9	1.17	49.8	1.24	84.5	2.10
O94772	Lymphocyte antigen 6H OS	647.1	610.4	0.94	724.7	1.12	443.8	0.69	673.8	1.04	688.9	1.06	882.3	1.36	634.8	0.98
C3VMY8	Alpha B crystallin OS=Hom	449.9	670.4	1.49	480.8	1.07	514.7	1.14	530.1	1.18	657.6	1.46	546.5	1.21	531.7	1.18
A0A024RD	3-hydroxybutyrate dehydrog	86.1	124.4	1.44	92.5	1.07	119.7	1.39	93.9	1.09	95.3	1.11	90.5	1.05	79.4	0.92
A0A075B6I	Immunoglobulin lambda var	1686	1561.4	0.93	2106.3	1.25	1329.5	0.79	1787	1.06	1749.1	1.04	2090.8	1.24	2051.3	1.22
Q969H8	Myeloid-derived growth fac	628.9	598.6	0.95	813.4	1.29	547	0.87	611.4	0.97	608.5	0.97	600.9	0.96	695.2	1.11
A0A024RD	Tumor necrosis factor recep	233.8	241.4	1.03	262.3	1.12	209.8	0.90	237.7	1.02	239.2	1.02	242.4	1.04	204.1	0.87
P81605	Dermcidin OS=Homo sapie	563.4	618	1.10	1243.9	2.21	520.9	0.92	708.1	1.26	690.7	1.23	658.5	1.17	818.3	1.45
Q02413	Desmoglein-1 OS=Homo sa	117.5	138.6	1.18	120.1	1.02	132.3	1.13	120.5	1.03	118.2	1.01	122.6	1.04	112	0.95
Q9Y6Q6	Tumor necrosis factor recep	139.8	169.1	1.21	162.6	1.16	104.8	0.75	158.9	1.14	145.6	1.04	149.7	1.07	134.6	0.96
P15291	Beta-1,4-galactosyltransfera	589.3	783	1.33	466.4	0.79	567	0.96	648.5	1.10	951	1.61	997.2	1.69	484.1	0.82
A8K2U0	Alpha-2-macroglobulin-like	313.5	366.3	1.17	309.3	0.99	284.2	0.91	315.2	1.01	295.3	0.94	282.6	0.90	361.2	1.15
Q9HC38	Glyoxalase domain-containi	331.6	498.6	1.50	388.2	1.17	319	0.96	357.1	1.08	408	1.23	360.4	1.09	321.7	0.97
B1AH90	Signal peptide, CUB and EC	118.8	100.6	0.85	170.1	1.43	79.4	0.67	116.6	0.98	101.8	0.86	107	0.90	116	0.98
C0JYY2	Apolipoprotein B (Including	40.5	99.2	2.45	64	1.58	64.4	1.59	61.9	1.53	50.3	1.24	95.9	2.37	49.6	1.22
Q53SS8	Epididymis secretory protein	44.4	62.8	1.41	59.6	1.34	37.2	0.84	56.6	1.27	63.6	1.43	56.5	1.27	58.3	1.31
P07307	Asialoglycoprotein receptor	40.5	42.3	1.04	43	1.06	57.1	1.41	41.9	1.03	47.7	1.18	40	0.99	35.8	0.88
Q8NES3	Beta-1,3-N-acetylglucosami	47.5	40.4	0.85	40.6	0.85	31.8	0.67	38.6	0.81	42	0.88	35.1	0.74	42.7	0.90
P19021	Peptidyl-glycine alpha-amid	96.2	98.6	1.02	88.9	0.92	75.9	0.79	92.7	0.96	100.1	1.04	129.3	1.34	83.3	0.87
Q9UEF7	Klotho OS=Homo sapiens	214.6	279.8	1.30	257.9	1.20	230.4	1.07	264.9	1.23	250	1.16	258.7	1.21	200.7	0.94
A8K865	cDNA FLJ75789, highly sin	271.8	345.7	1.27	270.3	0.99	197.2	0.73	341.9	1.26	299.2	1.10	283.5	1.04	354	1.30
O75340	Programmed cell death prot	205	189	0.92	153.1	0.75	173.9	0.85	182.4	0.89	212.6	1.04	186.2	0.91	181.1	0.88
P21266	Glutathione S-transferase M	61.6	66	1.07	50.1	0.81	39.2	0.64	48.3	0.78	72.2	1.17	63.9	1.04	48.8	0.79
A0A0B4J2D	Immunoglobulin kappa varia	101.1	89.8	0.89	85.7	0.85	85.5	0.85	77.9	0.77	82.1	0.81	94.4	0.93	67.6	0.67
Q6IB39	RNASE6 protein OS=Homo	310.4	351.3	1.13	500.5	1.61	279.2	0.90	465.7	1.50	368.4	1.19	370.8	1.19	347.1	1.12
B2RCJ5	cDNA, FLJ96113, highly sin	89	125	1.40	90.1	1.01	115.3	1.30	103.1	1.16	104.1	1.17	107.2	1.20	95	1.07
Q59F30	Fibroblast growth factor rec	33.7	36.9	1.09	33.4	0.99	27.5	0.82	27.7	0.82	31.3	0.93	38.8	1.15	37.8	1.12
C7FDU7	MHC class I antigen (Fragm															
A0A024RBU	Vacuolar protein sorting 37															
H7C2N1	Prothymosin alpha (Fragme	488.7	569.2	1.16	522.1	1.07	314.9	0.64	576.9	1.18	527.6	1.08	654.5	1.34	549.3	1.12
Q495M3	Proton-coupled amino acid	204.8	221.4	1.08	214.9	1.05	152.7	0.75	203.2	0.99	194.1	0.95	232.3	1.13	167.2	0.82
Q04721	Neurogenic locus notch hom	466.6	481.2	1.03	394.6	0.85	439	0.94	462.4	0.99	423.5	0.91	417.2	0.89	442.6	0.95
A0A087WT	Neuroblastoma suppressor c	2274.8	1931.4	0.85	2167	0.95	1555.1	0.68	2070.6	0.91	2151.1	0.95	1769.4	0.78	1990.9	0.88
Q9NZU0	Leucine-rich repeat transme	23.7	20	0.84	17.8	0.75	13.4	0.57	18.3	0.77	26	1.10	19.6	0.83	18.1	0.76
A0A0A0MT	Titin OS=Homo sapiens GN	154.4	181	1.17	143.9	0.93	131.7	0.85	172.5	1.12	184.8	1.20	256	1.66	195.2	1.26

E7ERL8	Neurexin-1-beta OS=Homo	982.7	1014.5	1.03	785.4	0.80	812.8	0.83	993.7	1.01	949.7	0.97	900.2	0.92	846.1	0.86
O96033	Molybdopterin synthase sulf	328.1	326.8	1.00	303.4	0.92	280.7	0.86	312.9	0.95	361.7	1.10	331.1	1.01	305.8	0.93
Q9NP85	Podocin OS=Homo sapiens	105.2	101.3	0.96	102.8	0.98	92.9	0.88	95.8	0.91	99.4	0.94	113.6	1.08	80.7	0.77
P04430	Immunoglobulin kappa varia															
A0A140VK.	Testis secretory sperm-bindi	201.6	306	1.52	265.6	1.32	193.6	0.96	267	1.32	278.8	1.38	245.7	1.22	218.2	1.08
Q59EA3	Cadherin 5, type 2 prepropr	31.7	27	0.85	43.2	1.36	24.1	0.76	36.3	1.15	37.2	1.17	26.3	0.83	45.4	1.43
B3KQT8	cDNA PSEC0172 fis, clone	391.1	384.5	0.98	386.2	0.99	320.4	0.82	383.5	0.98	415.3	1.06	464.9	1.19	350.9	0.90
J3QQX2	Rho GDP-dissociation inhib	379.3	418.3	1.10	384.4	1.01	291.8	0.77	383.7	1.01	406.8	1.07	341.3	0.90	324.2	0.85
Q13145	BMP and activin membrane	609.5	721.2	1.18	716.1	1.17	499.1	0.82	686.7	1.13	651.3	1.07	736.3	1.21	548.2	0.90
P52209	6-phosphogluconate dehydr	38.5	65.6	1.70	44.4	1.15	28.9	0.75	75.7	1.97	59.6	1.55	63.6	1.65	66.1	1.72
E1U340	ZNF511/PRAP1 fusion prot	132.7	165.1	1.24	180.6	1.36	109.3	0.82	108.7	0.82	149.5	1.13	140.2	1.06	120.8	0.91
A1L3A3	Contactin 2 (Axonal) OS=H	90.3	100.3	1.11	77.4	0.86	95	1.05	74.3	0.82	85	0.94	89.4	0.99	76.8	0.85
Q6FHG5	Gamma-synuclein OS=Hom	107.4	125.4	1.17	98.2	0.91	99.8	0.93	88.4	0.82	109.2	1.02	168.6	1.57	120	1.12
A8K4A5	cDNA FLJ77482, highly sin	364.1	453.2	1.24	345.1	0.95	446.9	1.23	402.3	1.10	466.4	1.28	436.6	1.20	293.7	0.81
Q59FB9	Toll interacting protein varia	32.7	40.8	1.25	33.5	1.02	34.7	1.06	27.8	0.85	34.3	1.05	43.9	1.34	27	0.83
Q9H6S3	Epidermal growth factor rec	260.1	262.5	1.01	249.9	0.96	190.6	0.73	271.4	1.04	313.3	1.20	238.5	0.92	248.1	0.95
A0A0B4J1R	4-hydroxyphenylpyruvate di	90	135.8	1.51	93.1	1.03	99.6	1.11	99.8	1.11	124.5	1.38	113.8	1.26	77.7	0.86
A0A024R3U	RAB4A, member RAS onco	32.2	28.9	0.90	39.9	1.24	24.3	0.75	33.8	1.05	36.2	1.12	32.5	1.01	26.2	0.81
P03973	Antileukoproteinase OS=Ho	431.3	423.3	0.98	599.4	1.39	265.8	0.62	552.7	1.28	486.9	1.13	358.9	0.83	398.8	0.92
P51170	Amiloride-sensitive sodium	446.9	377.2	0.84	270.6	0.61	256.5	0.57	322.6	0.72	414.9	0.93	372	0.83	303	0.68
Q9H461	Frizzled-8 OS=Homo sapien	671.3	660.3	0.98	701	1.04	481.4	0.72	692.9	1.03	680.7	1.01	536.9	0.80	744.7	1.11
Q9UIH2	Tumor necrosis factor recep															
Q8IX04	Ubiquitin-conjugating enzyn	171	196.9	1.15	171.8	1.00	165.5	0.97	201.4	1.18	198.6	1.16	192.3	1.12	143.1	0.84
E0D851	Platelet glycoprotein Ib alph	339.9	303.1	0.89	480.4	1.41	241.3	0.71	306.7	0.90	289.2	0.85	319.5	0.94	387.8	1.14
Q08629	Testican-1 OS=Homo sapien	171.3	178.7	1.04	123.5	0.72	137.4	0.80	123.8	0.72	178	1.04	244.7	1.43	161.4	0.94
Q6FHJ7	Secreted frizzled-related pro															
M0R2A0	ER membrane protein comp	363.1	434.3	1.20	403.2	1.11	333.2	0.92	392.1	1.08	403.6	1.11	418	1.15	380.2	1.05
P41217	OX-2 membrane glycoprote	206.1	281.4	1.37	163.2	0.79	156	0.76	177.1	0.86	208.6	1.01	198.4	0.96	190.4	0.92
P04040	Catalase OS=Homo sapiens	121	208.2	1.72	118.8	0.98	176.6	1.46	136.9	1.13	122.1	1.01	150.9	1.25	142.6	1.18
Q5T9B7	Adenylate kinase isoenzyme	184.3	220.3	1.20	237.8	1.29	160.4	0.87	199.6	1.08	214.7	1.16	222.1	1.21	186	1.01
Q6DKI7	Transmembrane protein PVI	38	32.6	0.86	37.4	0.98	31.4	0.83	29.6	0.78	37.1	0.98	37	0.97	36.6	0.96
Q5TBU5	HCG1773630 OS=Homo sa	329.8	357.7	1.08	354.9	1.08	235.1	0.71	329.4	1.00	387.6	1.18	376.1	1.14	334.3	1.01
P05091	Aldehyde dehydrogenase, m	445.7	405.9	0.91	337.3	0.76	286.8	0.64	386.3	0.87	392	0.88	498	1.12	460.4	1.03
P30047	GTP cyclohydrolase 1 feedb	235.6	271.7	1.15	211.3	0.90	400.7	1.70	226.7	0.96	227.2	0.96	255.3	1.08	256.1	1.09
H0YBZ2	HLA class II histocompatibi	274.6	269.2	0.98	297.7	1.08	207.8	0.76	261.5	0.95	267.9	0.98	251	0.91	279.4	1.02
Q9NX12	cDNA FLJ20496 fis, clone I	15.3	22.1	1.44	17	1.11	19.4	1.27	16.7	1.09	17.3	1.13	18.2	1.19	20.1	1.31
P04275	von Willebrand factor OS=I	93.5	105	1.12	110	1.18	70.9	0.76	112.1	1.20	100.8	1.08	128.3	1.37	91.8	0.98
A4D0V4	Capping protein (Actin filan	20.4	38	1.86	24.1	1.18	19.3	0.95	27.7	1.36	28.4	1.39	25.3	1.24	23.7	1.16
A0A024R4A	Alkaline phosphatase OS=H	60.2	95.8	1.59	84	1.40	42.8	0.71	64.5	1.07	71.3	1.18	54.8	0.91	77.3	1.28
A0A0B4J1Y	Immunoglobulin heavy varia	818.1	866.3	1.06	781.2	0.95	845	1.03	956.7	1.17	917	1.12	770.6	0.94	938.3	1.15
A0A0C4DH	Protein IGHV3-38 (Fragmen	198.3	329.9	1.66	264.4	1.33	234.3	1.18	265.1	1.34	232	1.17	232.8	1.17	197.9	1.00
A0A140VJR	Testicular tissue protein Li 1	45.8	62.9	1.37	44.8	0.98	45	0.98	42.9	0.94	48.2	1.05	48.5	1.06	44.2	0.97
P38606	V-type proton ATPase catal	392.4	579.3	1.48	472.7	1.20	392.9	1.00	491.1	1.25	551.3	1.40	557.4	1.42	412.8	1.05
Q13443	Disintegrin and metalloprote	139.8	138.9	0.99	101.6	0.73	111.1	0.79	119.6	0.86	137.6	0.98	125.2	0.90	124.1	0.89
P03951	Coagulation factor XI OS=I	77.2	89.1	1.15	92.9	1.20	65.7	0.85	73	0.95	80.2	1.04	71.8	0.93	81	1.05
P13688	Carcinoembryonic antigen-r	77.3	101.9	1.32	81.2	1.05	145	1.88	97.9	1.27	93.1	1.20	90.4	1.17	64.8	0.84
F1T0L0	LY6/PLAUR domain contai	291.3	254.5	0.87	198.8	0.68	474.9	1.63	235	0.81	245.6	0.84	197.6	0.68	199.8	0.69
A0A0F7TA	IGHV5-51 protein (Fragmen	298.3	341.1	1.14	267.2	0.90	284.3	0.95	304.6	1.02	319.3	1.07	352.1	1.18	334.6	1.12
Q68CJ9	Cyclic AMP-responsive elen	1983.8	1786.2	0.90	1292.7	0.65	1177.1	0.59	1312.2	0.66	1536.9	0.77	1710.6	0.86	1563.2	0.79
P48740	Mannan-binding lectin serin	79.2	95.1	1.20	87	1.10	77.2	0.97	83.6	1.06	78.7	0.99	79.9	1.01	65.6	0.83
H0YMD1	Low-density lipoprotein rece	1237.7	1161.4	0.94	1156.6	0.93	819.6	0.66	1033.7	0.84	1101.8	0.89	1176.6	0.95	994.7	0.80
A0A140VJE	Guanylate cyclase OS=Hom	82.9	122.6	1.48	80.2	0.97	107.8	1.30	76.9	0.93	87.7	1.06	104.5	1.26	68.1	0.82
Q8N474	Secreted frizzled-related pro	63.3	69.1	1.09	72.5	1.15	46.7	0.74	66.6	1.05	78.1	1.23	97.4	1.54	53.2	0.84
B2RDI5	cDNA, FLJ96627, highly si	146.3	177.4	1.21	176.8	1.21	133.6	0.91	178	1.22	171	1.17	153.7	1.05	156.2	1.07
A0A024R2C	Cysteine-rich with EGF-like	54.1	54.2	1.00	33.9	0.63	42.3	0.78	40.8	0.75	50.5	0.93	55.8	1.03	52.7	0.97
P01601	Immunoglobulin kappa varia	45.5	36.6	0.80	46.7	1.03	35.4	0.78	38.1	0.84	42.3	0.93	46	1.01	41.2	0.91
P08246	Neutrophil elastase OS=Hor	343.6	698	2.03	435.3	1.27	307.3	0.89	476.7	1.39	467.3	1.36	783.7	2.28	579	1.69
Q9NP84	Tumor necrosis factor recep	882.1	783	0.89	972.6	1.10	539.2	0.61	805.6	0.91	815.5	0.92	1053.8	1.19	932.4	1.06

P61604	10 kDa heat shock protein, r	96.8	81.8	0.85	68.9	0.71	45.4	0.47	67	0.69	89.2	0.92	94.9	0.98	63.5	0.66
Q06828	Fibromodulin OS=Homo sa	345.6	329.9	0.95	449.2	1.30	272.6	0.79	305.2	0.88	322.7	0.93	329.6	0.95	269.3	0.78
Q86SQ8	Beta-defensin-1 (Fragment)	268.5	304.5	1.13	470.9	1.75	245.3	0.91	347.5	1.29	277.7	1.03	309.6	1.15	583.1	2.17
A6XMV9	Protease serine 2 preproprot	95.5	103.6	1.08	144	1.51	75.1	0.79	86.9	0.91	108.9	1.14	87	0.91	97.4	1.02
A8K669	cDNA FLJ78452, highly sin	30.4	48.8	1.61	35.1	1.15	69.4	2.28	29.6	0.97	27	0.89	40.1	1.32	34.2	1.13
P09529	Inhibin beta B chain OS=Ho	189.4	193.1	1.02	181.7	0.96	158.5	0.84	180.3	0.95	203	1.07	216.8	1.14	175.3	0.93
P29966	Myristoylated alanine-rich C	270.4	286.4	1.06	232.1	0.86	274.9	1.02	281.1	1.04	333.6	1.23	319.7	1.18	267.5	0.99
A2J1M8	Rheumatoid factor RF-IP12															
A0A0K0K11	Epididymis secretory sperm	331.6	527.2	1.59	321.9	0.97	331.9	1.00	393.6	1.19	454.7	1.37	466.8	1.41	389.6	1.17
B4DQD2	cDNA FLJ52935, highly sin	141	162.5	1.15	163.7	1.16	120.1	0.85	147.7	1.05	148.9	1.06	178.4	1.27	182.9	1.30
P01706	Immunoglobulin lambda var	669.4	595.3	0.89	572.2	0.85	482.2	0.72	505.5	0.76	498.8	0.75	632.5	0.94	881.9	1.32
Q8NBS9	Thioredoxin domain-contair	401.7	455	1.13	427.6	1.06	373.8	0.93	415.5	1.03	450.1	1.12	418.8	1.04	437.5	1.09
D3DRD5	Fibroblast growth factor rec	462.5	443.1	0.96	377.3	0.82	337.9	0.73	395.2	0.85	419.3	0.91	410.9	0.89	350.7	0.76
Q8TCT8	Signal peptide peptidase-like	348.3	324.4	0.93	255.8	0.73	225.6	0.65	298.9	0.86	321.8	0.92	252.6	0.73	300.1	0.86
J3KQ18	D-dopachrome decarboxylas	220.3	368.8	1.67	226.4	1.03	211	0.96	209.5	0.95	294.9	1.34	265.2	1.20	220.2	1.00
Q53GD1	Guanine nucleotide-binding	93.5	123.9	1.33	102	1.09	115.1	1.23	94.5	1.01	103.4	1.11	99.5	1.06	98	1.05
A0A087WY	Glycoprotein hormones alph	415.7	439.2	1.06	500.6	1.20	302.8	0.73	385.9	0.93	442.7	1.06	516.7	1.24	398	0.96
P14174	Macrophage migration inhib	1538	2360.8	1.53	1882.7	1.22	1457.4	0.95	1941.2	1.26	2284.4	1.49	2061.6	1.34	1939.5	1.26
P50897	Palmitoyl-protein thioesteras	155.9	178.1	1.14	142.9	0.92	153.5	0.98	153.4	0.98	175.5	1.13	155.9	1.00	136.5	0.88
A0A024R8Z	L-selectin OS=Homo sapien	313	249.1	0.80	228.8	0.73	238.7	0.76	271.7	0.87	235.9	0.75	240.4	0.77	289.1	0.92
Q7L0X0	TLR4 interactor with leucin	179.7	187.3	1.04	173.1	0.96	115.2	0.64	164.8	0.92	187.9	1.05	186.2	1.04	147.4	0.82
Q53H93	Diphtheria toxin receptor (H															
P05387	60S acidic ribosomal protein															
A0A0J9YX3	Uncharacterized protein (Fra	201.9	259.7	1.29	205.1	1.02	225.1	1.11	222.3	1.10	237.7	1.18	210.6	1.04	210.6	1.04
J3QL71	Secernin-2 OS=Homo sapie	68.1	64.9	0.95	53	0.78	89.4	1.31	62.7	0.92	69	1.01	68.1	1.00	62.9	0.92
P30039	Phenazine biosynthesis-like	152.6	187.9	1.23	134.4	0.88	286.2	1.88	158.6	1.04	167.2	1.10	157.2	1.03	147.8	0.97
A2NH53	Immunogobulin kappa, VJ r	76.9	71	0.92	95	1.24	94.4	1.23	82.9	1.08	84.2	1.09	76.1	0.99	72.6	0.94
P83110	Serine protease HTRA3 OS	63.3	68.8	1.09	85.8	1.36	54.8	0.87	74	1.17	81.9	1.29	67.3	1.06	71.2	1.12
Q9UN73	Protocadherin alpha-6 OS=I	40.4	31.1	0.77	31.4	0.78	29.3	0.73	36.9	0.91	43.9	1.09	29.8	0.74	37	0.92
O75223	Gamma-glutamylcyclotransf	195.6	224.9	1.15	183.1	0.94	139.5	0.71	198.5	1.01	196.5	1.00	192.5	0.98	198.6	1.02
Q86V85	Integral membrane protein C	196.6	176.3	0.90	212.7	1.08	185.7	0.94	190.2	0.97	191.9	0.98	186	0.95	184.9	0.94
Q9UBX1	Cathepsin F OS=Homo sapi	111.4	122.2	1.10	112.4	1.01	74.8	0.67	106.5	0.96	117.2	1.05	117	1.05	107.9	0.97
P54727	UV excision repair protein F	221.8	245.5	1.11	263.1	1.19	184.6	0.83	226.1	1.02	247.5	1.12	243.2	1.10	225.8	1.02
A0A0U1RR	Histone H2A OS=Homo sap	601.7	1265.2	2.10	1004.6	1.67	657	1.09	736.5	1.22	768.7	1.28	1312.5	2.18	1501.5	2.50
A0A024RA.	Arylsulfatase B, isoform CR	236.9	348.4	1.47	208.9	0.88	177.6	0.75	252.2	1.06	249	1.05	377.4	1.59	280.4	1.18
C6SUN5	Agouti related protein homo	40.8	57.6	1.41	51.7	1.27	45.4	1.11	57.9	1.42	59.5	1.46	56.1	1.38	44.4	1.09
P01258	Calcitonin OS=Homo sapier	403.3	337.3	0.84	256.4	0.64	244.8	0.61	394.7	0.98	377.9	0.94	484.4	1.20	337.8	0.84
A8K9M5	cDNA FLJ77947, highly sin	227.6	266.1	1.17	179	0.79	218.4	0.96	235.1	1.03	237.7	1.04	231.4	1.02	297.9	1.31
C9JJE7	Mucin-20 OS=Homo sapien	232.7	231.2	0.99	183.3	0.79	172.6	0.74	174.9	0.75	230.8	0.99	225.1	0.97	214	0.92
B2R880	cDNA, FLJ93778, highly si	44.1	59.7	1.35	50.9	1.15	44.9	1.02	37.9	0.86	43.2	0.98	25.8	0.59	28.1	0.64
B4E3D4	cDNA FLJ56293, highly sin	227.7	247.5	1.09	237.5	1.04	207.8	0.91	288.2	1.27	251.5	1.10	268.3	1.18	235.3	1.03
B4E0V9	cDNA FLJ61198, highly sin	247.7	321.7	1.30	188.6	0.76	267.3	1.08	234.5	0.95	234.7	0.95	313.8	1.27	269.9	1.09
A0A024RAI	Bridging integrator 1, isofor	97.9	102.2	1.04	99.4	1.02	80.5	0.82	99.3	1.01	100.3	1.02	93.2	0.95	104.4	1.07
P29401	Transketolase OS=Homo sa	98.4	169.1	1.72	153.2	1.56	86.8	0.88	120.2	1.22	135.8	1.38	144.1	1.46	143	1.45
A0A0K0K11	Proteasome subunit alpha ty	153.3	193.9	1.26	139.1	0.91	136.1	0.89	143.2	0.93	168	1.10	156.9	1.02	135.8	0.89
A8K2N0	cDNA FLJ77835, highly sin	191.2	248.6	1.30	174.1	0.91	212.7	1.11	218.5	1.14	219.3	1.15	215.7	1.13	198.7	1.04
P16402	Histone H1.3 OS=Homo sap	316	425.3	1.35	325.7	1.03	311.8	0.99	319.2	1.01	351.6	1.11	338.8	1.07	357.1	1.13
Q92484	Acid sphingomyelinase-like	52	64.5	1.24	69.1	1.33	58.4	1.12	76.4	1.47	59.9	1.15	50.5	0.97	56.8	1.09
Q4G0I0	Protein CCSMST1 OS=Hor	42	46.9	1.12	48.4	1.15	40	0.95	40.9	0.97	38.6	0.92	52.8	1.26	42	1.00
B4DHQ3	Phosphoserine aminotransfe	55.9	68.4	1.22	66.7	1.19	45.6	0.82	56.6	1.01	71.6	1.28	78	1.40	38.4	0.69
A0A0F7RQ	Luteinizing hormone beta po	148.7	203.3	1.37	177.7	1.20	116.7	0.78	157.6	1.06	162.8	1.09	197.7	1.33	141.3	0.95
Q9H1C7	Cysteine-rich and transmem	70	66.4	0.95	79.3	1.13	49.4	0.71	76.9	1.10	75.7	1.08	72.3	1.03	81.3	1.16
Q02388	Collagen alpha-1(VII) chain	159.4	200	1.25	209.7	1.32	150.4	0.94	208.5	1.31	191.1	1.20	184.6	1.16	152.2	0.95
A0A0A1HA	H.sapiens ras-related Hrab2	92.1	208.1	2.26	93.3	1.01	122.1	1.33	95.6	1.04	100.7	1.09	107.1	1.16	91	0.99
Q6E0U4	Dermokine OS=Homo sapie	502.4	434.7	0.87	622	1.24	343	0.68	499.8	0.99	432.9	0.86	610.5	1.22	569.4	1.13
P30626	Sorcin OS=Homo sapiens G	172.9	208.4	1.21	190.1	1.10	185.6	1.07	186.5	1.08	193.4	1.12	193.2	1.12	175.9	1.02
B4DMN1	cDNA FLJ61136, highly sin	97.6	101.6	1.04	87.7	0.90	83.3	0.85	97	0.99	102.7	1.05	99.1	1.02	92.1	0.94
A0A024R8C	Ubiquitin-related modifier 1	75.6	94.2	1.25	79.6	1.05	89.2	1.18	85.5	1.13	91.8	1.21	83.7	1.11	80.4	1.06

P24298	Alanine aminotransferase 1	57.6	107.8	1.87	66.1	1.15	60.3	1.05	75.7	1.31	80.6	1.40	75	1.30	58	1.01
V9HW88	Calreticulin, isoform CRA_	75	81.1	1.08	58.9	0.79	57.1	0.76	59.9	0.80	74.5	0.99	70.8	0.94	75.2	1.00
Q6UY14	ADAMTS-like protein 4 OS	255.7	196.7	0.77	182.4	0.71	159.1	0.62	186.5	0.73	216.3	0.85	223.5	0.87	230.7	0.90
P07360	Complement component C8	45.6	56.8	1.25	57.3	1.26	47.2	1.04	45.5	1.00	54.2	1.19	59.1	1.30	41.9	0.92
E7EMK3	Flotillin-2 OS=Homo sapier	27.6	47.2	1.71	32.1	1.16	29.1	1.05	33	1.20	27.2	0.99	29.6	1.07	23.8	0.86
P49773	Histidine triad nucleotide-bi	222.4	303.5	1.36	250.6	1.13	213.6	0.96	265	1.19	317.5	1.43	283.1	1.27	231.7	1.04
Q6PK18	2-oxoglutarate and iron-dep	16.9	22.6	1.34	16.8	0.99	10.6	0.63	16.8	0.99	17.5	1.04	22.5	1.33	17.3	1.02
B2R6U9	Delta-like protein OS=Hom	856.5	760.5	0.89	673	0.79	681.9	0.80	773.3	0.90	799	0.93	730.2	0.85	685.2	0.80
O94985	Calsyntenin-1 OS=Homo sa	316.3	369.1	1.17	348.3	1.10	362.2	1.15	317.9	1.01	351.9	1.11	320.1	1.01	299.6	0.95
O95196	Chondroitin sulfate proteog	103.8	97.4	0.94	99.4	0.96	71.7	0.69	97.3	0.94	87.8	0.85	100.5	0.97	91	0.88
B4DJ06	cDNA FLJ56620 OS=Hom	626.6	625	1.00	596.7	0.95	514.3	0.82	607.2	0.97	615.8	0.98	661.2	1.06	602.6	0.96
Q9UM47	Neurogenic locus notch hon	65.6	77.9	1.19	56.7	0.86	56.7	0.86	63.1	0.96	64.2	0.98	55.7	0.85	59.5	0.91
Q9UL82	Myosin-reactive immunoglo	916.9	743.1	0.81	1333.8	1.45	663.3	0.72	916.6	1.00	800	0.87	1182.3	1.29	1116.4	1.22
Q12929	Epidermal growth factor rec	99.3	155.1	1.56	115.3	1.16	95.4	0.96	125	1.26	132.6	1.34	137.4	1.38	107	1.08
Q86VB7	Scavenger receptor cysteine	313	412.1	1.32	419	1.34	271.5	0.87	341.3	1.09	329.9	1.05	317.9	1.02	385.1	1.23
A0A024RC2	Desmocollin 3, isoform CR	207.8	188.6	0.91	189.1	0.91	144.7	0.70	204	0.98	213.5	1.03	188.5	0.91	171.5	0.83
A8K144	cDNA FLJ76746, highly sin	882.3	930.9	1.06	848.6	0.96	1135.9	1.29	842.5	0.95	861.5	0.98	991.5	1.12	634.4	0.72
A0A125U0	GCT-A2 heavy chain variab	115.8	140.8	1.22	103.1	0.89	176.5	1.52	110.8	0.96	113.3	0.98	132	1.14	112.7	0.97
A0A024RC	CD99 antigen-like 2, isofor	94.6	161.6	1.71	144.2	1.52	84.6	0.89	120.6	1.27	104.1	1.10	107.1	1.13	112	1.18
O15144	Actin-related protein 2/3 co	87	96.8	1.11	118.4	1.36	58.1	0.67	97.7	1.12	91.1	1.05	119.6	1.37	70.8	0.81
Q6UWI4	Protein shisa-2 homolog OS	160.1	202.1	1.26	167.2	1.04	173.4	1.08	150.2	0.94	163	1.02	165.9	1.04	158.3	0.99
Q9UL75	Myosin-reactive immunoglo	216.4	204	0.94	157.4	0.73	158.5	0.73	196.9	0.91	212.6	0.98	186.8	0.86	210.6	0.97
Q6UXB4	C-type lectin domain family	2672	2538.5	0.95	2606.1	0.98	2702.7	1.01	2706.2	1.01	2743.9	1.03	2656.2	0.99	2493.7	0.93
P06734	Low affinity immunoglobuli	203.8	267.8	1.31	227.4	1.12	167.2	0.82	234.7	1.15	236.7	1.16	250.8	1.23	192.1	0.94
P99999	Cytochrome c OS=Homo sa	193.2	201.5	1.04	149.1	0.77	130.2	0.67	175.2	0.91	218.2	1.13	205	1.06	198.7	1.03
Q59EB6	Complement component 1,	115.4	109	0.94	111.3	0.96	89.9	0.78	107.9	0.94	112.1	0.97	108.5	0.94	107.6	0.93
A0A087WY	Proline-rich protein 4 OS=H	379.1	479.1	1.26	437.1	1.15	306.2	0.81	521.4	1.38	446.1	1.18	479.8	1.27	579.7	1.53
P55786	Puromycin-sensitive aminop	23.7	36.4	1.54	23	0.97	20.8	0.88	29.7	1.25	24.1	1.02	32.9	1.39	19.7	0.83
P08253	72 kDa type IV collagenase	397.7	450.4	1.13	374.4	0.94	319.4	0.80	455.1	1.14	467.9	1.18	427.8	1.08	440.5	1.11
A0A0X9US	MS-A6 heavy chain variable	89	112.7	1.27	93.7	1.05	128	1.44	95.1	1.07	93.6	1.05	99.8	1.12	83.3	0.94
P05026	Sodium/potassium-transport	330.7	356.5	1.08	377.7	1.14	234.5	0.71	333.6	1.01	337	1.02	348.8	1.05	260.5	0.79
A0A0C4DH	Protein IGHV3-35 (Fragme	655.5	675.8	1.03	478.8	0.73	693.6	1.06	631.1	0.96	625.7	0.95	519.5	0.79	629.1	0.96
P49908	Selenoprotein P OS=Homo	1007.4	877.6	0.87	810.3	0.80	674.7	0.67	816.2	0.81	890.8	0.88	881.1	0.87	832.8	0.83
A0A0A0MT	Protein IGKJ4 (Fragment)	836.2	890.9	1.07	947.2	1.13	637.4	0.76	760	0.91	1080	1.29	920.6	1.10	710.9	0.85
Q14210	Lymphocyte antigen 6D OS	834.8	892.2	1.07	1131.4	1.36	695.9	0.83	1037.5	1.24	1030.1	1.23	1101.4	1.32	710.4	0.85
Q59ER5	WD repeat-containing prote	59.9	103.8	1.73	59.3	0.99	55.6	0.93	64.5	1.08	74.1	1.24	90.3	1.51	65.6	1.10
B3KTG0	cDNA FLJ38180 fis, clone	36.2	41.2	1.14	32.3	0.89	46.7	1.29	36.6	1.01	44.9	1.24	47	1.30	32.5	0.90
A2N2F4	VK3 protein (Fragment) OS	285.9	254.5	0.89	491.3	1.72	198.9	0.70	269.7	0.94	305.8	1.07	289.6	1.01	279.5	0.98
A0A193CH	10E8 light chain variable re	444	372.6	0.84	422.7	0.95	502.2	1.13	415.8	0.94	365.1	0.82	544.3	1.23	476.1	1.07
Q9Y4L1	Hypoxia up-regulated protei	20.5	22.8	1.11	15.6	0.76	24.1	1.18	15.4	0.75	20.8	1.01	19.8	0.97	16.2	0.79
P27930	Interleukin-1 receptor type 2															
A8K6C9	cDNA FLJ78037, highly sin	272.2	240.9	0.89	365.4	1.34	149	0.55	244.8	0.90	221.6	0.81	240.3	0.88	301	1.11
A0A140VK	Transaldolase OS=Homo sa	211	292.6	1.39	233.8	1.11	171.3	0.81	269.6	1.28	253	1.20	318.9	1.51	364.6	1.73
P37802	Transgelin-2 OS=Homo sap	116.5	141.4	1.21	104.2	0.89	169.7	1.46	130.8	1.12	138.6	1.19	125.8	1.08	143.9	1.24
Q9UQN3	Charged multivesicular body	188.2	234.2	1.24	189.7	1.01	168.8	0.90	214.4	1.14	206.1	1.10	235.6	1.25	156.6	0.83
B2R4F3	cDNA, FLJ92068, highly si	117.3	168.2	1.43	99.1	0.84	95.3	0.81	132.3	1.13	118.3	1.01	126.8	1.08	143.1	1.22
J3QRS3	Myosin regulatory light chai	35.4	42.5	1.20	39.1	1.10	23.9	0.68	31	0.88	30.9	0.87	40.9	1.16	39.6	1.12
Q96QS0	Putative matrix cell adhesio	126.5	120.8	0.95	106.1	0.84	111.5	0.88	106.1	0.84	125	0.99	105.8	0.84	131	1.04
P18428	Lipopolysaccharide-binding	258	352	1.36	289.9	1.12	263	1.02	288.8	1.12	340.7	1.32	328	1.27	428.6	1.66
P98161	Polycystin-1 OS=Homo sap	161.1	180.8	1.12	161.5	1.00	151.3	0.94	154.8	0.96	161.3	1.00	156.1	0.97	133.4	0.83
Q14112	Nidogen-2 OS=Homo sapie	113.6	136.9	1.21	111	0.98	115.9	1.02	117.9	1.04	105	0.92	110.9	0.98	102	0.90
A0A1B1CY	Vitamin D binding protein (	346.4	504.5	1.46	309.5	0.89	867.1	2.50	484.5	1.40	320.7	0.93	325.4	0.94	236.3	0.68
I3L504	Eukaryotic translation initia	170.1	246.8	1.45	234.1	1.38	145.1	0.85	217.5	1.28	209.8	1.23	214.1	1.26	171.6	1.01
Q53HU8	Vimentin variant (Fragment	35.3	52.8	1.50	36.1	1.02	30.6	0.87	31.6	0.90	31.1	0.88	40.4	1.14	39.4	1.12
Q8TBP5	Membrane protein FAM174	157.3	147.6	0.94	111	0.71	106.1	0.67	153.3	0.97	148.7	0.95	138.3	0.88	127.4	0.81
B2R6W4	cDNA, FLJ93149, highly si	394.5	389.3	0.99	514.4	1.30	297.3	0.75	432.2	1.10	419.4	1.06	394.1	1.00	333.2	0.84
V6A6E5	MHC class I antigen OS=H	78.4	74.1	0.95	88.9	1.13	64.2	0.82	84.8	1.08	76.4	0.97	82.1	1.05	65.1	0.83
P82980	Retinol-binding protein 5 O	701.1	757.5	1.08	632	0.90	453.8	0.65	676.4	0.96	864.8	1.23	746.1	1.06	595.4	0.85

A0A0X9T7I	MS-A6 light chain variable	54.3	72.9	1.34	60.7	1.12	55.9	1.03	58.5	1.08	52.7	0.97	68.7	1.27	54.3	1.00
F8RHH9	MHC class I antigen (Fragm															
Q14117	Dihydropyrimidinase OS=H	130.9	201.8	1.54	141.6	1.08	111	0.85	138.3	1.06	209.1	1.60	172.8	1.32	124.8	0.95
P19256	Lymphocyte function-associ	773.2	776.7	1.00	626.6	0.81	595.5	0.77	697.1	0.90	721.6	0.93	753.2	0.97	653.8	0.85
Q86XT2	Vacuolar protein sorting-ass	66	58.7	0.89	62.4	0.95	53.8	0.82	65.6	0.99	69.1	1.05	61.2	0.93	66	1.00
D2IYL2	Corneodesmosin OS=Homo	415	520.3	1.25	544.5	1.31	442.9	1.07	475.2	1.15	444.8	1.07	430.4	1.04	364.4	0.88
Q5HYL0	Putative uncharacterized pro	48.9	51.4	1.05	49.3	1.01	38.6	0.79	41	0.84	49.9	1.02	28.4	0.58	32.2	0.66
Q6UWV6	Ectonucleotide pyrophospha	193.3	219.4	1.14	157.8	0.82	192.7	1.00	178.4	0.92	197.1	1.02	172.8	0.89	150.7	0.78
K7ES00	Histone H3.3 (Fragment) OS	602.6	1428.3	2.37	1016.6	1.69	681.7	1.13	754.7	1.25	827.1	1.37	1406.8	2.33	1900.6	3.15
Q8NC54	Keratinocyte-associated tran	485.1	490.9	1.01	422.4	0.87	421.1	0.87	420.2	0.87	493.8	1.02	456.3	0.94	497.4	1.03
P15907	Beta-galactoside alpha-2,6-s	75.5	91.2	1.21	67.6	0.90	79.4	1.05	77.6	1.03	75.9	1.01	104.5	1.38	67.3	0.89
P22223	Cadherin-3 OS=Homo sapie															
A0A0X9TD	GCT-A3 heavy chain variab															
Q96MM7	Heparan-sulfate 6-O-sulfotra	94.5	138.9	1.47	122	1.29	121	1.28	106.4	1.13	117.8	1.25	127.9	1.35	89.3	0.94
P25774	Cathepsin S OS=Homo sapi	115.9	108.5	0.94	98.8	0.85	80.5	0.69	87.4	0.75	102.4	0.88	121.4	1.05	97.5	0.84
P19075	Tetraspanin-8 OS=Homo sa	182	207.2	1.14	165.4	0.91	218	1.20	182.1	1.00	194	1.07	130.4	0.72	138.4	0.76
B3KT21	cDNA FLJ37476 fis, clone	99.1	92.6	0.93	74.3	0.75	70.6	0.71	75.1	0.76	87.6	0.88	100.3	1.01	74.6	0.75
V9HW80	Epididymis luminal protein															
Q9BS26	Endoplasmic reticulum resic	229.6	243	1.06	228.2	0.99	158.4	0.69	204.3	0.89	229.6	1.00	239.3	1.04	208.1	0.91
Q6FGQ5	ASGR1 protein (Fragment)	266.7	229.7	0.86	206.2	0.77	158.4	0.59	233.7	0.88	247.8	0.93	269.9	1.01	231.5	0.87
A8K243	cDNA FLJ78172, highly sin	55	78.2	1.42	45.5	0.83	62.4	1.13	50.4	0.92	55	1.00	57.2	1.04	40.8	0.74
P22748	Carbonic anhydrase 4 OS=H	300.4	335.3	1.12	298.2	0.99	233.2	0.78	357.8	1.19	421.7	1.40	312.2	1.04	296.8	0.99
B4DW34	cDNA FLJ56798, highly sin	44.2	49.7	1.12	38.2	0.86	44.3	1.00	47.3	1.07	42	0.95	52	1.18	40.8	0.92
Q9UN66	Protocadherin beta-8 OS=H	45.3	45	0.99	31	0.68	49.9	1.10	33.1	0.73	40.7	0.90	41.5	0.92	31.4	0.69
A4D1W8	Ependymin related protein 1	403.7	406.2	1.01	412.1	1.02	301.2	0.75	360.1	0.89	393.5	0.97	365.2	0.90	408.3	1.01
O43488	Aflatoxin B1 aldehyde redu	18.6	24.5	1.32	21.3	1.15	15.4	0.83	15.7	0.84	27	1.45	23.8	1.28	15.2	0.82
Q8WWZ8	Oncoprotein-induced transc	90.4	130.7	1.45	103.9	1.15	78.8	0.87	104.1	1.15	90.5	1.00	106.3	1.18	102.2	1.13
B2R7D1	cDNA, FLJ93387, highly si	39.4	55.5	1.41	20.2	0.51	47.3	1.20	39.5	1.00	47.6	1.21	23	0.58	17.2	0.44
Q9BUD6	Spondin-2 OS=Homo sapie	86.9	84.6	0.97	153	1.76	94.8	1.09	94.6	1.09	108.7	1.25	72.1	0.83	73	0.84
B5MCA4	Epithelial cell adhesion mol															
A0AVF1	Intraflagellar transport prote	1223.2	989.9	0.81	1068.8	0.87	738.7	0.60	1308.1	1.07	1250	1.02	1127.4	0.92	850.5	0.70
Q9NZV1	Cysteine-rich motor neuron	79.9	75.1	0.94	85.6	1.07	67.2	0.84	89.7	1.12	82.2	1.03	96.3	1.21	66.9	0.84
Q86X29	Lipolysis-stimulated lipopro	57.1	69.9	1.22	72.6	1.27	58.2	1.02	69	1.21	77	1.35	56	0.98	62.5	1.09
A0A024R88	ATPase, H+ transporting, ly	71.8	99.1	1.38	83.5	1.16	70.3	0.98	98.9	1.38	90.4	1.26	82.9	1.15	71.4	0.99
P49767	Vascular endothelial growth															
A0A024R17	Leukotriene B4 12-hydroxy	62.7	77.5	1.24	61.8	0.99	51.5	0.82	61.2	0.98	64.7	1.03	58.9	0.94	63.2	1.01
A1L471	ATP-binding cassette, sub-f	121	125.8	1.04	86.1	0.71	96.3	0.80	96.5	0.80	119.6	0.99	123.2	1.02	74	0.61
A8K2T7	Receptor protein-tyrosine ki															
V9HW55	Proteasome endopeptidase c	211.7	260.2	1.23	296.6	1.40	189.1	0.89	259.1	1.22	262.2	1.24	247.5	1.17	244	1.15
P03950	Angiogenin OS=Homo sapi	422.6	404.3	0.96	446.7	1.06	333	0.79	406.2	0.96	389.5	0.92	423.6	1.00	441.8	1.05
A0A0S2Z4E	Solute carrier family 3 (Cyst	54.8	80.7	1.47	62.9	1.15	44.2	0.81	61.5	1.12	64	1.17	57.6	1.05	55.3	1.01
Q03167	Transforming growth factor	66	97.9	1.48	80.6	1.22	58.7	0.89	84.1	1.27	80.2	1.22	93.4	1.42	88.1	1.33
C9IYI1	Dermokine OS=Homo sapie															
H7C215	Reticulon-4 receptor (Fragm	96.5	93.9	0.97	111	1.15	89.7	0.93	97.2	1.01	106.2	1.10	102.9	1.07	87	0.90
P55107	Growth/differentiation facto	234.2	219.7	0.94	161.8	0.69	160.6	0.69	172.8	0.74	181.2	0.77	183.7	0.78	155	0.66
P14784	Interleukin-2 receptor subun	188.2	179.6	0.95	170.7	0.91	131.8	0.70	161.9	0.86	159.9	0.85	177.7	0.94	149.1	0.79
V9HWH1	Epididymis luminal protein	59.9	86.5	1.44	65.8	1.10	58.1	0.97	85.4	1.43	68.1	1.14	78.1	1.30	76.8	1.28
O43405	Cochlin OS=Homo sapiens	452.5	346.4	0.77	511.1	1.13	275.7	0.61	415	0.92	442.1	0.98	351.6	0.78	386.2	0.85
A0A1B0GW	Alpha-aminoadipic semialde	23.3	31	1.33	29.2	1.25	24.1	1.03	23.1	0.99	24.2	1.04	34.6	1.48	25.3	1.09
F2RM35	Serine protease OS=Homo s															
Q9NR34	Mannosyl-oligosaccharide 1															
O00299	Chloride intracellular chann	80	104.1	1.30	95.7	1.20	71.5	0.89	94.6	1.18	102.6	1.28	87.8	1.10	80.7	1.01
A0A024R9E	Mal, T-cell differentiation p	104	132	1.27	109.8	1.06	84.5	0.81	94	0.90	101.7	0.98	107.9	1.04	88.7	0.85
P31150	Rab GDP dissociation inhib	91.8	114.8	1.25	83.7	0.91	86.9	0.95	103.1	1.12	99.2	1.08	109	1.19	78	0.85
A0A024R31	Laminin, beta 2 (Laminin S)	122.5	134.8	1.10	142.9	1.17	111	0.91	114.4	0.93	122.6	1.00	150.8	1.23	107.1	0.87
B2R7N9	cDNA, FLJ93532, highly si	309.7	338.1	1.09	349.5	1.13	238.1	0.77	343.8	1.11	309.6	1.00	357.4	1.15	319.5	1.03
P01704	Immunoglobulin lambda var	246.2	304.8	1.24	454.4	1.85	233	0.95	303.9	1.23	281.4	1.14	342.4	1.39	229.9	0.93
Q8N126	Cell adhesion molecule 3 OS	80.7	73.7	0.91	95.3	1.18	77.6	0.96	79.7	0.99	78.2	0.97	95.7	1.19	96.6	1.20

A0A0X9V9	IBM-B2 light chain variable	293.4	266.4	0.91	237.5	0.81	191.9	0.65	303.4	1.03	357.1	1.22	284.3	0.97	328.7	1.12
Q9HBJ8	Collectrin OS=Homo sapien	94	109.6	1.17	79.1	0.84	82.2	0.87	100.7	1.07	125.9	1.34	115.8	1.23	83.4	0.89
Q86UZ8	FZD2 protein (Fragment) O	609.8	550.6	0.90	579.5	0.95	371.6	0.61	612.1	1.00	620.5	1.02	612.7	1.00	574.2	0.94
H3BQD0	Cerebellin-1 OS=Homo sap	333	317.7	0.95	722.6	2.17	197	0.59	399.5	1.20	338.3	1.02	205.1	0.62	329.1	0.99
Q96MU8	Kremen protein 1 OS=Homo	27.5	48.9	1.78	33.9	1.23	29.8	1.08	33.6	1.22	29	1.05	33.5	1.22	27.3	0.99
P58546	Myotrophin OS=Homo sapi	58.6	53.1	0.91	55.5	0.95	41.4	0.71	55.5	0.95	60.5	1.03	59.3	1.01	66.4	1.13
P22304	Iduronate 2-sulfatase OS=H	123.9	142.9	1.15	111.3	0.90	112.3	0.91	119.5	0.96	122.3	0.99	122.3	0.99	114.7	0.93
B3KNV8	Polypeptide N-acetylgalacto	143.7	157.5	1.10	140.2	0.98	128.1	0.89	159.4	1.11	206.1	1.43	178	1.24	126.5	0.88
O75787	Renin receptor OS=Homo s	450.3	973.9	2.16	474.6	1.05	387.6	0.86	490.8	1.09	579.7	1.29	599.7	1.33	413.1	0.92
Q5T7S2	Receptor protein serine/thre															
O75071	EF-hand calcium-binding do	337.1	330.6	0.98	300.4	0.89	204.3	0.61	311.9	0.93	342.7	1.02	292.9	0.87	321.9	0.95
D2JYI1	TGF-beta receptor type-2 O	55.8	75.2	1.35	79.8	1.43	66	1.18	58	1.04	57.9	1.04	57.7	1.03	60.7	1.09
P04118	Colipase OS=Homo sapiens	777.9	838.9	1.08	902.8	1.16	513.9	0.66	804.5	1.03	1277.9	1.64	865.7	1.11	738.1	0.95
O43567	E3 ubiquitin-protein ligase R	675	649.5	0.96	599.2	0.89	425.8	0.63	697.7	1.03	681.4	1.01	632.1	0.94	715.8	1.06
A0A0G2JN4	Mucin-6 OS=Homo sapiens															
Q13790	Apolipoprotein F OS=Homo	235.1	161	0.68	116.9	0.50	111	0.47	115	0.49	175.3	0.75	183	0.78	196.6	0.84
Q9H9P2	Chondrolectin OS=Homo sa	15.2	13.6	0.89	10.7	0.70	10.2	0.67	9.1	0.60	12.1	0.80	13.9	0.91	13.3	0.88
P08887	Interleukin-6 receptor subun	57.9	65.3	1.13	66.6	1.15	55.5	0.96	67.5	1.17	58.2	1.01	74.7	1.29	61.2	1.06
A0A024RAI	Calpastatin, isoform CRA_a															
Q6UWH4	Protein FAM198B OS=Hom	373.9	323.8	0.87	311.7	0.83	325.3	0.87	464.7	1.24	424.6	1.14	365.6	0.98	310.8	0.83
B2RDY9	Adenylyl cyclase-associated	273.4	218	0.80	156.3	0.57	174.4	0.64	161.1	0.59	153.9	0.56	175.6	0.64	157.4	0.58
A0A087X0I	Growth hormone receptor O	105.8	111.5	1.05	87.9	0.83	75	0.71	90.5	0.86	99.4	0.94	101.9	0.96	99	0.94
P51606	N-acylglucosamine 2-epime	55.8	88.6	1.59	67.2	1.20	88.8	1.59	73.3	1.31	70.8	1.27	73.7	1.32	51.2	0.92
P27824	Calnexin OS=Homo sapiens	29.3	30.4	1.04	34.9	1.19	38.5	1.31	26.9	0.92	26.2	0.89	31.8	1.09	29.9	1.02
B2RAN2	cDNA, FLJ95014, highly si	235.9	354.5	1.50	285.9	1.21	320.7	1.36	249.9	1.06	274.4	1.16	243.3	1.03	262.6	1.11
Q9UGM5	Fetuin-B OS=Homo sapiens	93.5	140	1.50	114.8	1.23	112.9	1.21	118.9	1.27	111.8	1.20	131.1	1.40	125.2	1.34
B9EKV4	Aldehyde dehydrogenase 9	11.7	20.2	1.73	9.8	0.84	16.8	1.44	12.6	1.08	16.9	1.44	13.9	1.19	10.9	0.93
P78310	Coxsackievirus and adenovi	31.6	47.1	1.49	37.6	1.19	35.6	1.13	40	1.27	38.2	1.21	27.4	0.87	31.1	0.98
O75074	Low-density lipoprotein rec	218.8	189.3	0.87	150.2	0.69	159.1	0.73	185.1	0.85	210.7	0.96	203.7	0.93	194.8	0.89
Q0VDC6	Peptidylprolyl isomerase OS	33.7	35.3	1.05	33.8	1.00	40.5	1.20	34.5	1.02	35	1.04	37.7	1.12	38.2	1.13
A8K5Y2	cDNA FLJ77228, highly sin	420.4	405.2	0.96	432	1.03	309.4	0.74	414.4	0.99	409.5	0.97	361.2	0.86	330.4	0.79
Q96FE5	Leucine-rich repeat and imm	587.4	521.7	0.89	476.9	0.81	443.6	0.76	523.5	0.89	515	0.88	537.2	0.91	379.8	0.65
Q96MG2	Junctional sarcoplasmic reti	262.2	258.5	0.99	272.2	1.04	176.9	0.67	234.6	0.89	310.4	1.18	225	0.86	192.1	0.73
A8K9U9	cDNA FLJ75862, highly sin															
D6W5K2	Thymosin, beta 10, isoform	878.5	1247	1.42	1089.4	1.24	536.4	0.61	1030.5	1.17	1004.3	1.14	1341.3	1.53	1170.9	1.33
A0A0B5HR	Serine/threonine-protein kin	128.2	117.6	0.92	109.8	0.86	106.5	0.83	123.8	0.97	133	1.04	114.8	0.90	113.3	0.88
Q9NWV4	UPF0587 protein C1orf123	206.9	259.2	1.25	373.2	1.80	182.5	0.88	221.3	1.07	246.3	1.19	177.1	0.86	199.5	0.96
P02545	Prelamin-A/C OS=Homo sa	64.1	173.5	2.71	98.4	1.54	115.4	1.80	86	1.34	73	1.14	89.4	1.39	66.8	1.04
A4D2P0	Ras-related C3 botulinum to	78.2	84.6	1.08	71	0.91	71.3	0.91	84.1	1.08	105.5	1.35	82.4	1.05	93.3	1.19
Q86YT9	Junctional adhesion molecul	101.9	124.7	1.22	180	1.77	88.4	0.87	138.5	1.36	122.5	1.20	103.1	1.01	101.5	1.00
P15529	Membrane cofactor protein	40.8	35.9	0.88	45.1	1.11	31.2	0.76	44.7	1.10	39.4	0.97	33.8	0.83	35.4	0.87
Q9BYE4	Small proline-rich protein 2	217.1	225.5	1.04	254.8	1.17	318.1	1.47	255.2	1.18	243.8	1.12	263.5	1.21	244.6	1.13
H7BZJ3	Protein disulfide-isomerase															
B2RC95	cDNA, FLJ95923, highly si	122.9	144.5	1.18	134.2	1.09	114.9	0.93	142.2	1.16	146.9	1.20	130	1.06	120.5	0.98
Q9BYJ0	Fibroblast growth factor-bin	64.1	58	0.90	64	1.00	38.3	0.60	57.8	0.90	70.6	1.10	49.3	0.77	57.2	0.89
Q96PC5	Melanoma inhibitory activit	116.3	112.2	0.96	94.7	0.81	98.6	0.85	106.7	0.92	106.4	0.91	110.9	0.95	102.6	0.88
Q9UMX5	Neudesin OS=Homo sapien	235.1	229.2	0.97	209.7	0.89	167.5	0.71	226.5	0.96	244.1	1.04	222.8	0.95	206.9	0.88
Q9Y4I9	Tyrosine phosphatase IA-2b	150.1	155.4	1.04	147.2	0.98	121.1	0.81	139.4	0.93	156.3	1.04	131.5	0.88	109	0.73
P11234	Ras-related protein Ral-B O	19.1	29.3	1.53	22.5	1.18	20.2	1.06	20.2	1.06	25.2	1.32	39.8	2.08	17.4	0.91
Q16581	C3a anaphylatoxin chemota	63.4	70.9	1.12	63.9	1.01	67.8	1.07	64.7	1.02	70.1	1.11	65.3	1.03	63.1	1.00
B2R6S5	UMP-CMP kinase OS=Hon	319.9	406.5	1.27	384.8	1.20	240.2	0.75	339.2	1.06	404	1.26	438.8	1.37	308.5	0.96
A0A1B1RV	Lipoprotein lipase OS=Hom	56.3	80.3	1.43	60.9	1.08	70.8	1.26	74.6	1.33	80.2	1.42	69.5	1.23	64.4	1.14
Q59ED5	Tetraspanin (Fragment) OS=	67.4	62.9	0.93	84.1	1.25	55.4	0.82	67.3	1.00	65.1	0.97	51.7	0.77	63.2	0.94
A8K139	cDNA FLJ76744, highly sin	68	96.3	1.42	73.4	1.08	67.1	0.99	72.2	1.06	85.9	1.26	68.7	1.01	69	1.01
G3V5Z7	Proteasome subunit alpha ty	106.8	170.1	1.59	150.6	1.41	125.1	1.17	142.8	1.34	125.9	1.18	146.2	1.37	113.5	1.06
Q8NFU3	Thiosulfate sulfurtransferase															
P32241	Vasoactive intestinal polype	35.1	40.8	1.16	43	1.23	32.2	0.92	42.1	1.20	34.4	0.98	35	1.00	35.3	1.01
Q8IYJ0	PILR alpha-associated neura	120.9	128.5	1.06	124.5	1.03	84.4	0.70	124.2	1.03	117.3	0.97	122.3	1.01	89.9	0.74

A0A075B6S	Protein IGKV1-37 (Fragmen	20.1	36.2	1.80	41.8	2.08	32.6	1.62	23.3	1.16	32	1.59	39.4	1.96	25.9	1.29
A0A087WY	Tumor protein D54 OS=Ho	215.4	266	1.23	228.2	1.06	169.7	0.79	242.9	1.13	214.3	0.99	266.8	1.24	244.4	1.13
Q03164	Histone-lysine N-methyltran	606.1	472.5	0.78	2094.7	3.46	378.3	0.62	540	0.89	729.5	1.20	922.7	1.52	521.2	0.86
P13639	Elongation factor 2 OS=Hon															
O95502	Neuronal pentraxin receptor															
X5D2G8	Fibroblast growth factor rec	237.9	316.4	1.33	228.2	0.96	256.8	1.08	255.9	1.08	249.8	1.05	294.5	1.24	206.5	0.87
A0A024R3V	Eukaryotic translation elong															
Q8WY15	Sodium/glucose cotransport															
C5IX07	Tumor-associated calcium s	127.5	130.5	1.02	115.3	0.90	103.3	0.81	129.5	1.02	127.1	1.00	125.5	0.98	120.7	0.95
Q9H2A7	C-X-C motif chemokine 16															
A0A075B7D	Protein IGHV1OR15-1 (Fra															
I3L0A0	HCG2044781 OS=Homo sa	148.5	377.8	2.54	175.7	1.18	137.8	0.93	193	1.30	212.5	1.43	188.3	1.27	177.6	1.20
Q7L5L3	Glycerophosphodiester phos	11.7	18.2	1.56	12.9	1.10	10.4	0.89	9.7	0.83	12.6	1.08	16.3	1.39	15.3	1.31
Q9GZP0	Platelet-derived growth fact															
A8K061	cDNA FLJ77880, highly sin	74.5	64.3	0.86	67.7	0.91	76.5	1.03	67	0.90	89.8	1.21	81.7	1.10	73.2	0.98
O00622	Protein CYR61 OS=Homo s	39.4	41.8	1.06	41	1.04	33.5	0.85	39.3	1.00	44.8	1.14	42.5	1.08	38	0.96
A0A1K0FU	Globin E1 OS=Homo sapien	7.8	18.4	2.36	13.1	1.68	13.3	1.71	9.4	1.21	11	1.41	19	2.44	11.2	1.44
P10721	Mast/stem cell growth facto	56.5	59	1.04	57.5	1.02	50.6	0.90	62	1.10	59.2	1.05	53.7	0.95	51.9	0.92
Q9ULK6	RING finger protein 150 OS	263.9	261.6	0.99	235	0.89	217	0.82	266.4	1.01	292.7	1.11	242.5	0.92	220.3	0.83
Q8NG20	Plasminogen/activator kring	180.8	122.3	0.68	103.2	0.57	93.3	0.52	119.2	0.66	128.8	0.71	136.6	0.76	211.5	1.17
Q8N2G4	Ly6/PLAUR domain-contain															
A0A0G2JH	Myelin-oligodendrocyte gly	99.1	99.9	1.01	90.3	0.91	80.3	0.81	99.6	1.01	104.5	1.05	91.2	0.92	80.9	0.82
Q53Y06	ATPase, H+ transporting, ly	145.2	222.5	1.53	215.2	1.48	198.8	1.37	192.5	1.33	212.5	1.46	224.7	1.55	172.8	1.19
B5TMG5	Histidine rich calcium bindi	636.8	603	0.95	401.1	0.63	373.4	0.59	572.2	0.90	635.3	1.00	520.7	0.82	435.4	0.68
B7Z6S9	Glucosylceramidase OS=Ho	86.2	122.6	1.42	87.3	1.01	87.1	1.01	96	1.11	128	1.48	102.8	1.19	80.7	0.94
Q9H6Y7	E3 ubiquitin-protein ligase R	93.1	101.6	1.09	68.8	0.74	62.8	0.67	85	0.91	78.9	0.85	83.8	0.90	78.9	0.85
Q6ZSJ9	Protein shisa-6 homolog OS	109.9	116.6	1.06	120	1.09	86.5	0.79	107.4	0.98	104.5	0.95	123	1.12	91.1	0.83
G9FP35	Guanine nucleotide binding	122.5	134.5	1.10	134.8	1.10	103.7	0.85	151	1.23	151.6	1.24	153.1	1.25	124.5	1.02
O14786	Neuropilin-1 OS=Homo sap	11.3	17	1.50	13.9	1.23	10.9	0.96	9.3	0.82	8.1	0.72	13.4	1.19	12.5	1.11
Q7L9L4	MOB kinase activator 1B O	10.2	20.6	2.02	9.6	0.94	11.8	1.16	11.5	1.13	18.8	1.84	15.3	1.50	12	1.18
Q59FM9	TYRO3 protein tyrosine kin															
B2RB70	Neurocalcin delta, isoform C	155	193.8	1.25	147.6	0.95	126.8	0.82	158.4	1.02	186.1	1.20	177.7	1.15	138.9	0.90
A0A024R23	Tyrosine-protein kinase rece	25.5	24.4	0.96	28.1	1.10	13.5	0.53	27.7	1.09	22.5	0.88	22.9	0.90	22.5	0.88
Q53TN4	Cytochrome b reductase 1 O	186.2	212.3	1.14	168.5	0.90	174.6	0.94	222	1.19	237.1	1.27	180.6	0.97	148.3	0.80
Q15043	Zinc transporter ZIP14 OS=	133	163.4	1.23	128.1	0.96	122.7	0.92	168	1.26	153	1.15	257.6	1.94	128.8	0.97
Q08708	CMRF35-like molecule 6 O	425.2	433.4	1.02	357.3	0.84	289.4	0.68	479.1	1.13	520.1	1.22	507.8	1.19	424.8	1.00
A0A0B4J1S	High affinity immunoglobul	380.5	321.4	0.84	269	0.71	236	0.62	340.9	0.90	291.6	0.77	368.7	0.97	347.6	0.91
P0DKV0	Spermatogenesis-associated	633.5	605.1	0.96	512.5	0.81	762.5	1.20	501.2	0.79	473	0.75	581.2	0.92	639.1	1.01
A0A087X1	Serpin B6 OS=Homo sapien	59.1	63.8	1.08	65.1	1.10	50	0.85	56.8	0.96	64.3	1.09	59.2	1.00	57.3	0.97
V9GYJ8	Apolipoprotein C-II OS=Ho															
Q7Z7G8	Vacuolar protein sorting-ass	184	192.1	1.04	268	1.46	132.2	0.72	204.4	1.11	219.2	1.19	153.4	0.83	185.6	1.01
A0A0X9US	MS-D2 light chain variable															
A8K9D8	cDNA FLJ78129, highly sin	79.3	103.8	1.31	78.4	0.99	76.9	0.97	75	0.95	75.2	0.95	77.5	0.98	73.2	0.92
A0A087WT	Protein sidekick-1 OS=Hom															
P61457	Pterin-4-alpha-carbinolamin	182.4	247.2	1.36	161.1	0.88	146.9	0.81	164.6	0.90	199	1.09	176.3	0.97	161.5	0.89
P80511	Protein S100-A12 OS=Hom	750.5	1210	1.61	875.6	1.17	607.9	0.81	1073.4	1.43	1010.6	1.35	1011.6	1.35	1638.1	2.18
Q8WWQ8	Stabilin-2 OS=Homo sapien	11.6	26.1	2.25	15.4	1.33	19.7	1.70	16	1.38	21.3	1.84	17.4	1.50	17	1.47
A0A087WS	Nucleobindin 2, isoform CR	49.3	86.5	1.75	56.8	1.15	43.7	0.89	72.2	1.46	64.8	1.31	62.8	1.27	53.6	1.09
A0A125U0U	MS-C3 light chain variable	98.8	117.6	1.19	79.2	0.80	155.7	1.58	107.3	1.09	114.3	1.16	84.8	0.86	93	0.94
Q6UW88	Epigen OS=Homo sapiens O	50.2	84.1	1.68	51.8	1.03	54.8	1.09	63.1	1.26	64.6	1.29	60.6	1.21	54.9	1.09
P09382	Galectin-1 OS=Homo sapien	68.1	78.6	1.15	83.7	1.23	69.6	1.02	86	1.26	77.4	1.14	71.7	1.05	53.6	0.79
P62070	Ras-related protein R-Ras2 O	61.1	83	1.36	54.3	0.89	65.6	1.07	75.1	1.23	77.2	1.26	81.2	1.33	54.4	0.89
B2RCC8	cDNA, FLJ95990 OS=Hom	17.6	34.1	1.94	21.8	1.24	17.4	0.99	21	1.19	22.2	1.26	30.9	1.76	18.9	1.07
Q96RZ2	N-acetylglucosamine-1-phos	59.5	63.6	1.07	47.3	0.79	40.9	0.69	49	0.82	66.4	1.12	53	0.89	47.9	0.81
P01127	Platelet-derived growth fact	56.2	74.1	1.32	50.8	0.90	50.1	0.89	53.7	0.96	54.8	0.98	62.9	1.12	49.5	0.88
A0A0C4DH	Protein IGKV6D-41 (Fragm	163.8	185.7	1.13	152.5	0.93	136.9	0.84	154.5	0.94	154.7	0.94	161	0.98	193.7	1.18
B2R9K8	cDNA, FLJ94440, highly si	17.3	45.5	2.63	22.9	1.32	21.2	1.23	23.8	1.38	29	1.68	38.9	2.25	27.8	1.61
Q9Y6U3	Adseverin OS=Homo sapien	70.8	92.6	1.31	92.8	1.31	64	0.90	79.2	1.12	73	1.03	84.9	1.20	85.4	1.21

O43493	Trans-Golgi network integra	164.8	163	0.99	121.1	0.73	104.1	0.63	144.2	0.88	141.3	0.86	178.1	1.08	128.6	0.78
Q9HCM3	UPF0606 protein KIAA154	22.7	26.1	1.15	16.9	0.74	15.7	0.69	19.2	0.85	21.6	0.95	21.3	0.94	18.8	0.83
P13521	Secretogranin-2 OS=Homo	132.3	186.6	1.41	232.1	1.75	131.2	0.99	217.4	1.64	159.4	1.20	226.3	1.71	149.7	1.13
P16234	Platelet-derived growth fact	29.7	33.2	1.12	26	0.88	30.5	1.03	26.2	0.88	29.4	0.99	22.1	0.74	27.9	0.94
Q96AX9	E3 ubiquitin-protein ligase M	88.1	84.1	0.95	28.5	0.32	30.2	0.34	59.4	0.67	52.6	0.60	78	0.89	45.5	0.52
Q86YD5	Low-density lipoprotein rec	10.8	14.4	1.33	17.7	1.64	9.9	0.92	16.5	1.53	11.8	1.09	14.2	1.31	11.6	1.07
A6NL88	Protein shisa-7 OS=Homo s	75.4	80	1.06	57	0.76	133	1.76	76.5	1.01	74.3	0.99	73.3	0.97	62.3	0.83
E5RIM7	Copper transport protein AT	29.3	31.9	1.09	34.8	1.19	32.1	1.10	25.7	0.88	27.2	0.93	34.5	1.18	35	1.19
Q9NQ79	Cartilage acidic protein 1 O	82.3	91.6	1.11	71.7	0.87	85	1.03	85.4	1.04	74.4	0.90	96	1.17	73.4	0.89
O94991	SLIT and NTRK-like protei	98.7	135.1	1.37	156.6	1.59	100.1	1.01	122.8	1.24	109.4	1.11	155.5	1.58	94.3	0.96
B4DZM1	cDNA FLJ58310, highly sin	261.6	243	0.93	208.8	0.80	295.4	1.13	222.6	0.85	225.1	0.86	244.1	0.93	219.2	0.84
B7Z6Z4	Myosin light polypeptide 6	66.5	80.6	1.21	56.1	0.84	37.9	0.57	54.2	0.82	63.5	0.95	67.3	1.01	69.6	1.05
O14494	Phospholipid phosphatase 1	152.4	196.6	1.29	151.5	0.99	147.6	0.97	149.9	0.98	176.9	1.16	126.3	0.83	151.3	0.99
Q8N386	Leucine-rich repeat-containi															
Q9UHY7	Enolase-phosphatase E1 OS	11.8	17.8	1.51	13.5	1.14	11	0.93	10.6	0.90	11.3	0.96	13.5	1.14	10.1	0.86
P17927	Complement receptor type 1	48.3	52	1.08	53.3	1.10	36.8	0.76	37.4	0.77	47.2	0.98	55.1	1.14	35.7	0.74
P29317	Ephrin type-A receptor 2 OS															
Q96S16	JmjC domain-containing pro															
P56159	GDNF family receptor alpha	334.3	412.4	1.23	328.2	0.98	386.6	1.16	351.5	1.05	317.1	0.95	499	1.49	312.1	0.93
Q9Y490	Talin-1 OS=Homo sapiens	46.5	49.7	1.07	55.6	1.20	32.6	0.70	40.9	0.88	45.8	0.98	57.4	1.23	49.1	1.06
Q59GD9	Myelin associated glycoprot															
A0A024R6I	Galactosylceramidase, isofo	63.7	99.9	1.57	78.5	1.23	61.6	0.97	82.6	1.30	77.4	1.22	78.2	1.23	78.9	1.24
A0A024R1K	Parvalbumin, isoform CRA_															
A0A024RB2	Solute carrier family 39 (M	65.3	103.6	1.59	57.4	0.88	45	0.69	58.4	0.89	77	1.18	79.3	1.21	42.8	0.66
Q5T7Y6	Protein S100 OS=Homo sap															
Q59EU6	EF hand domain family, me	10.2	22.4	2.20	12.7	1.25	13.6	1.33	12.2	1.20	18.2	1.78	17.3	1.70	13.4	1.31
Q7Z4W1	L-xylulose reductase OS=H	161	245.2	1.52	167.9	1.04	190.3	1.18	178.1	1.11	206.6	1.28	172.8	1.07	148.9	0.92
Q6I929	HCG1978654 OS=Homo sa	27.4	34.1	1.24	22.8	0.83	32	1.17	28.4	1.04	29.2	1.07	25.4	0.93	25.8	0.94
A0A0F7RQ	Follicle stimulating hormon	303.2	306.7	1.01	243.5	0.80	193.2	0.64	218.8	0.72	312.8	1.03	311	1.03	291.6	0.96
V9HWJ1	Glutathione synthetase OS=															
S4R347	Formin-binding protein 1-lik															
A0A1B0GU	Uncharacterized protein OS	27.8	25.5	0.92	14.2	0.51	22.1	0.79	17.1	0.62	16.8	0.60	23.3	0.84	22.6	0.81
A0A087WU	Protein SYNJ2BP-COX16 (															
Q9H741	UPF0454 protein C12orf49	29.4	30.5	1.04	34.9	1.19	27.2	0.93	29.7	1.01	28	0.95	26.2	0.89	23.4	0.80
P30101	Protein disulfide-isomerase	33	45.8	1.39	50	1.52	38.6	1.17	41.1	1.25	37.8	1.15	48.8	1.48	39.5	1.20
P22692	Insulin-like growth factor-bi	130.8	149.9	1.15	148.4	1.13	158.8	1.21	128.6	0.98	133.5	1.02	145.8	1.11	128.8	0.98
Q9H0X4	Protein FAM234A OS=Hor	42.8	61.4	1.43	44.4	1.04	48.8	1.14	50.7	1.18	53.1	1.24	49.7	1.16	37	0.86
Q6ZMU0	Delta-aminolevulinic acid de															
Q8TBD0	Uncharacterized protein (Fr	77.9	84.6	1.09	76.7	0.98	96	1.23	96.2	1.23	84.9	1.09	80.6	1.03	110.1	1.41
X6R7Y7	Intraflagellar transport prot	26.7	34.8	1.30	34.4	1.29	22.2	0.83	36	1.35	28	1.05	31.7	1.19	30.1	1.13
B4DU44	Involucrin OS=Homo sapien	29.9	43.4	1.45	25.7	0.86	34.3	1.15	28.3	0.95	30.1	1.01	26.8	0.90	39.5	1.32
Q8TER0	Sushi, nidogen and EGF-lik															
Q9Y3R5	Protein dopey-2 OS=Homo	18.7	33.4	1.79	52.7	2.82	33.8	1.81	23.2	1.24	25.8	1.38	24.2	1.29	17.6	0.94
B2R8C2	Tyrosine-protein kinase OS=															
P09543	2',3'-cyclic-nucleotide 3'-ph															
A0A024QZ	NAD(P)H dehydrogenase,	66.2	99.8	1.51	61.1	0.92	137.6	2.08	84.7	1.28	91.9	1.39	87.9	1.33	77.4	1.17
P51159	Ras-related protein Rab-27	190.9	207.2	1.09	259.3	1.36	193.5	1.01	185.5	0.97	183	0.96	126.7	0.66	123.1	0.64
P55010	Eukaryotic translation initia	346.4	330.4	0.95	250.3	0.72	242.2	0.70	292.2	0.84	297.8	0.86	333.6	0.96	296.7	0.86
P48509	CD151 antigen OS=Homo s	30.4	43.6	1.43	32.2	1.06	27.4	0.90	33.7	1.11	34.8	1.14	40.1	1.32	27.9	0.92
P18085	ADP-ribosylation factor 4 C	20.6	36.6	1.78	24.6	1.19	32.1	1.56	20.4	0.99	23.7	1.15	18.6	0.90	18.6	0.90
Q53H88	Dynactin 2 variant (Fragme	101.2	92.2	0.91	90.7	0.90	67.6	0.67	89.7	0.89	97.4	0.96	83.6	0.83	99.5	0.98
A8K8U1	cDNA FLJ77762, highly sin	26	36.4	1.40	29.7	1.14	24.4	0.94	26.6	1.02	34.4	1.32	30.3	1.17	28.7	1.10
B3KR33	cDNA FLJ33589 fis, clone	155.8	195.3	1.25	159.1	1.02	118.3	0.76	162.6	1.04	152.5	0.98	146.7	0.94	135.4	0.87
P54826	Growth arrest-specific prote	887	955.2	1.08	877.9	0.99	660.9	0.75	943.3	1.06	941.9	1.06	946.1	1.07	817.2	0.92
B4DWA6	cDNA FLJ60094, highly sin	153.1	188.6	1.23	155.8	1.02	147.6	0.96	140.3	0.92	151.8	0.99	157	1.03	157.1	1.03
D6W551	Chromosome 2 open reading	73.6	62.1	0.84	49.9	0.68	45.2	0.61	56.3	0.76	53.7	0.73	93.7	1.27	61.1	0.83
A0A140VJJ	S-formylglutathione hydrola															
A0A024R3I	Sodium channel, voltage-ga	167.9	169	1.01	131.3	0.78	121.3	0.72	136	0.81	165.8	0.99	129.4	0.77	130.2	0.78

A0A0A7E7	Endoplasmic reticulum amir															
O95861	3'(2'),5'-bisphosphate nucleo	21.3	35.5	1.67	23.6	1.11	20.7	0.97	26.8	1.26	33.3	1.56	31.7	1.49	25.7	1.21
A2NW98	Rheumatoid factor light cha	186.7	184.1	0.99	175.2	0.94	115.5	0.62	138.6	0.74	151.4	0.81	138	0.74	204.9	1.10
Q9HD42	Charged multivesicular body	126.2	131	1.04	102.8	0.81	87.8	0.70	109.3	0.87	136.5	1.08	115.9	0.92	107	0.85
B7Z6C9	Transmembrane 9 superfam	32.7	34.2	1.05	23.2	0.71	32.1	0.98	28.8	0.88	36.5	1.12	27	0.83	24.3	0.74
B2RAF9	Suppressor of tumorigenicit	33.1	48.5	1.47	61.9	1.87	42.9	1.30	45.8	1.38	50.1	1.51	48.5	1.47	43.1	1.30
P80723	Brain acid soluble protein 1	268	314.6	1.17	286.2	1.07	207.2	0.77	300.1	1.12	245.8	0.92	255.1	0.95	209	0.78
Q92743	Serine protease HTRA1 OS	74.4	299.2	4.02	82	1.10	52.3	0.70	72.8	0.98	66.7	0.90	123.9	1.67	70.4	0.95
Q8WU39	Marginal zone B- and B1-ce	35	39.7	1.13	45.7	1.31	28.3	0.81	46.6	1.33	36.5	1.04	35.9	1.03	39	1.11
O15031	Plexin-B2 OS=Homo sapien															
Q9UBV8	Peflin OS=Homo sapiens G	99.1	153.7	1.55	104.5	1.05	114.8	1.16	123.8	1.25	160	1.61	110.4	1.11	104.2	1.05
Q13740	CD166 antigen OS=Homo s	24.8	22.8	0.92	24.7	1.00	23	0.93	22.7	0.92	23.7	0.96	21	0.85	19.2	0.77
C9JIF9	Acylamino-acid-releasing en	37.4	50.4	1.35	48.8	1.30	39.8	1.06	45.2	1.21	45	1.20	55.3	1.48	33.9	0.91
A0N5G7	Rheumatoid factor D5 heav	88.1	107.1	1.22	80.3	0.91	141.9	1.61	77.7	0.88	82.6	0.94	93.1	1.06	102	1.16
U3KQ56	Glyoxylate reductase/hydro	11.8	18.4	1.56	9.6	0.81	14.1	1.19	9.9	0.84	14.3	1.21	12.3	1.04	10.1	0.86
P46952	3-hydroxyanthranilate 3,4-d	70.8	85.5	1.21	71.6	1.01	66.2	0.94	73.7	1.04	85.9	1.21	76.9	1.09	59.6	0.84
Q9BQR3	Serine protease 27 OS=Hon	62.1	65.2	1.05	64.6	1.04	51.4	0.83	74.6	1.20	71.8	1.16	76	1.22	74.8	1.20
Q13277	Syntaxin-3 OS=Homo sapie	106	140.8	1.33	89	0.84	118.1	1.11	125.9	1.19	121	1.14	118.4	1.12	103	0.97
Q14764	Major vault protein OS=Ho															
D6R956	Ubiquitin carboxyl-terminal	147.3	169.8	1.15	108.5	0.74	139.3	0.95	134.8	0.92	165.9	1.13	171.8	1.17	143.1	0.97
P16930	Fumarylacetoacetase OS=H															
A2TDC0	Titin-cap (Telethonin) OS=I															
Q99584	Protein S100-A13 OS=Hom	100	108.2	1.08	85.2	0.85	96.5	0.97	90.9	0.91	113.3	1.13	121.5	1.22	98.4	0.98
A0A0S2Z61	Transmembrane protein 67 i															
Q15366	Poly(rC)-binding protein 2 (															
L7RSM2	Mitogen-activated protein k	120.6	121.3	1.01	89	0.74	86.5	0.72	128.3	1.06	96.7	0.80	114.6	0.95	131.8	1.09
Q86SQ4	Adhesion G-protein coupled															
P29373	Cellular retinoic acid-bindin	32.7	47.2	1.44	40.6	1.24	28.7	0.88	39.7	1.21	40.1	1.23	44.5	1.36	50.4	1.54
C9JVC9	Voltage-dependent calcium	225.8	212	0.94	192.2	0.85	201.4	0.89	215.3	0.95	217	0.96	205.6	0.91	203.3	0.90
A0A024R63	Endothelin receptor type B,	216.1	227.3	1.05	195.1	0.90	217.3	1.01	220.6	1.02	229.2	1.06	234.7	1.09	194.8	0.90
Q9NX62	Inositol monophosphatase 3	13.2	22.3	1.69	18.3	1.39	15	1.14	17	1.29	23	1.74	24	1.82	17.2	1.30
Q9UBG0	C-type mannose receptor 2 (															
P28072	Proteasome subunit beta typ	55.8	61.9	1.11	39	0.70	53.2	0.95	40.9	0.73	49.1	0.88	47.2	0.85	43.9	0.79
P40197	Platelet glycoprotein V OS=															
P46781	40S ribosomal protein S9 O	93.2	156.7	1.68	139.9	1.50	111.5	1.20	155.4	1.67	134.7	1.45	90.5	0.97	199.3	2.14
A0A024RA	HCG2009644, isoform CRA	63	93.7	1.49	58.4	0.93	58.6	0.93	48.5	0.77	57.5	0.91	67.9	1.08	59.5	0.94
Q5R3I4	Tetratricopeptide repeat pro															
A0PJK1	Sodium/glucose cotransport															
A0A140VK	Leukotriene A(4) hydrolase	28.3	49.7	1.76	34.5	1.22	30	1.06	42.1	1.49	50	1.77	29.8	1.05	38.7	1.37
P23434	Glycine cleavage system H p	81.5	101.3	1.24	94.3	1.16	100.8	1.24	94.9	1.16	95.6	1.17	102.4	1.26	86.1	1.06
Q96HA4	Uncharacterized protein C1	194.7	186.6	0.96	156.9	0.81	179.8	0.92	192.1	0.99	167.4	0.86	158	0.81	185.4	0.95
Q96ID5	Immunoglobulin superfamil	35.5	28.9	0.81	32	0.90	17.1	0.48	31.9	0.90	26.6	0.75	25.8	0.73	22.6	0.64
J3QRU1	Tyrosine-protein kinase OS=	81.5	98.1	1.20	69.7	0.86	95.8	1.18	90.3	1.11	92.8	1.14	94.3	1.16	70.2	0.86
A0A0U1RQ	Neurexin-3-beta OS=Homo															
D0AB04	Rhesus blood group CcEe a															
D3DSM8	Formiminotransferase cyclo	26.4	46.7	1.77	34.5	1.31	25.4	0.96	26.1	0.99	42	1.59	36	1.36	20.6	0.78
A0A024R5U	ADAM metallopeptidase do	93.2	371.3	3.98	122.3	1.31	98.8	1.06	113.3	1.22	106.5	1.14	113.2	1.21	85.4	0.92
A0A024R1C	Tyrosylprotein sulfotransfer	88.3	77.1	0.87	96.7	1.10	65.4	0.74	77.8	0.88	80.1	0.91	60.1	0.68	81	0.92
P28827	Receptor-type tyrosine-prot															
G3V4P8	Glia maturation factor beta (	28.9	36.3	1.26	31.5	1.09	22.9	0.79	29.2	1.01	34.5	1.19	30.5	1.06	28.9	1.00
Q8IWX7	Protein unc-45 homolog B (															
Q9Y639	Neuroplastin OS=Homo sap	263.4	273.7	1.04	220.7	0.84	233.1	0.88	240.6	0.91	234.9	0.89	281.1	1.07	209.7	0.80
Q8IY95	Transmembrane protein 192															
P14778	Interleukin-1 receptor type 1	90.2	98.9	1.10	109.7	1.22	77.3	0.86	98.7	1.09	132	1.46	90	1.00	76.5	0.85
P46108	Adapter molecule crk OS=H	139.5	170.4	1.22	145.4	1.04	93.8	0.67	128.3	0.92	163.7	1.17	186.5	1.34	125.6	0.90
Q9Y546	Leucine-rich repeat-containi	408	580	1.42	771.1	1.89	389.3	0.95	745.5	1.83	575.7	1.41	444.1	1.09	517.5	1.27
B2R6Q2	Alkaline phosphatase OS=H	12	20.5	1.71	12.8	1.07	30.9	2.58	18.2	1.52	16.3	1.36	17.3	1.44	16	1.33
Q9H772	Gremlin-2 OS=Homo sapier	86.7	72.7	0.84	99.2	1.14	63.1	0.73	96	1.11	89.2	1.03	87	1.00	77.2	0.89

P10109	Adrenodoxin, mitochondrial	205.4	282.1	1.37	287.3	1.40	212.5	1.03	228.2	1.11	224.3	1.09	267.8	1.30	212.3	1.03
P23458	Tyrosine-protein kinase JAK															
Q8N353	TMEM106B protein (Fragm	215.6	249.7	1.16	232	1.08	285.2	1.32	289.5	1.34	240.2	1.11	269.5	1.25	296.3	1.37
Q8NF12	FLJ00390 protein (Fragmen															
Q7RU04	Aminopeptidase B OS=Hon															
P0C7U0	Protein ELFN1 OS=Homo s															
Q6ZUY8	Lipase OS=Homo sapiens P	40.4	45.1	1.12	34.9	0.86	57.1	1.41	45.1	1.12	45.9	1.14	45	1.11	37.5	0.93
Q6LEU6	HLA-DMB protein (Fragme	30.4	37.6	1.24	28.8	0.95	35.7	1.17	28.2	0.93	25.5	0.84	29.5	0.97	24.1	0.79
P10586	Receptor-type tyrosine-prote	121.8	109.7	0.90	122.4	1.00	119.4	0.98	110.8	0.91	116.3	0.95	122.4	1.00	95.8	0.79
A8K1K1	cDNA FLJ76342, highly sin	63.4	83.8	1.32	61.6	0.97	94.7	1.49	56.6	0.89	65.2	1.03	67.2	1.06	60.9	0.96
Q8TDY8	Immunoglobulin superfamily															
A0A087WU	ADP-ribosylation factor-like	32	33.6	1.05	58.9	1.84	30.3	0.95	37.4	1.17	33.8	1.06	31.1	0.97	29.3	0.92
Q99835	Smoothened homolog OS=H	361.7	292.2	0.81	222.3	0.61	203.6	0.56	298.2	0.82	367.2	1.02	328.6	0.91	232.8	0.64
B3KQQ9	cDNA PSEC0048 fis, clone	79	97.5	1.23	62.7	0.79	88.2	1.12	82.1	1.04	80.9	1.02	84.8	1.07	69.6	0.88
Q6ZP37	cDNA FLJ26554 fis, clone I															
O15393	Transmembrane protease ser															
O00159	Unconventional myosin-Ic O															
A0A024RDI	Tyrosylprotein sulfotransfer															
P19957	Elafin OS=Homo sapiens G	296.5	204	0.69	327.2	1.10	133.1	0.45	251.3	0.85	208.2	0.70	235.6	0.79	373.9	1.26
Q5T1S8	Noncompact myelin-associa	2795.3	2252	0.81	1819.3	0.65	1528.2	0.55	2147.8	0.77	2612.8	0.93	2270.6	0.81	1851.4	0.66
B2RAK1	cDNA, FLJ94965, highly sir															
A6NH11	Glycolipid transfer protein d															
P08833	Insulin-like growth factor-bi															
Q99795	Cell surface A33 antigen OS															
Q96TA2	ATP-dependent zinc metallo	165.7	108.6	0.66	85.8	0.52	72.9	0.44	123.8	0.75	115.8	0.70	164.1	0.99	164.7	0.99
F6SYP7	Integral membrane protein I	280.9	262.6	0.93	279	0.99	195.5	0.70	282.6	1.01	250.6	0.89	267	0.95	259	0.92
B2R6I6	cDNA, FLJ92965, highly sir															
E9PGC5	Receptor-type tyrosine-prote															
Q8TE68	Epidermal growth factor rec	39.2	47.8	1.22	44.8	1.14	34.9	0.89	46.7	1.19	42.8	1.09	47.2	1.20	36.8	0.94
Q14165	Malectin OS=Homo sapiens	67.8	67.4	0.99	64.6	0.95	55	0.81	59.6	0.88	62.8	0.93	56.9	0.84	56.1	0.83
A2KUC4	UGa8L (Fragment) OS=Hor	372.5	375.7	1.01	319	0.86	316.4	0.85	301.3	0.81	390.5	1.05	367.6	0.99	435.5	1.17
M1VPF4	Tyrosine-protein kinase rece	49	81.7	1.67	71.7	1.46	61.5	1.26	73.3	1.50	52.8	1.08	53.8	1.10	55.3	1.13
D3DQ48	Transmembrane protein 59,	123.5	178.8	1.45	126	1.02	154.3	1.25	153.8	1.25	119.6	0.97	169	1.37	123.9	1.00
B4DT57	cDNA FLJ61383, highly sin	54.3	60.3	1.11	54	0.99	42.4	0.78	62.1	1.14	66.2	1.22	57.4	1.06	44.3	0.82
Q9ULL1	Pleckstrin homology domain	396.8	520.4	1.31	833.4	2.10	440.1	1.11	566.1	1.43	484.9	1.22	464.1	1.17	457.1	1.15
A0A0B4J1S	Protein SELENOF OS=Hon															
Q8N729	Neuropeptide W OS=Homo	17.8	21	1.18	19.1	1.07	14.8	0.83	17.4	0.98	23.6	1.33	19.7	1.11	15.6	0.88
B3KPC7	Actin-related protein 2/3 cor															
B4DH02	cDNA FLJ50510, highly sin															
A0A024R23	Guanine deaminase, isoform															
A0A068LRV	Ig heavy chain variable regi	495.7	446.9	0.90	414	0.84	578	1.17	631.1	1.27	520.9	1.05	521.7	1.05	561.7	1.13
O94967	WD repeat-containing prote	112.6	140.5	1.25	384.9	3.42	106.5	0.95	191.3	1.70	119.3	1.06	112.5	1.00	111.5	0.99
A8K3H0	cDNA FLJ75548, highly sin															
J3KR56	Receptor activity-modifying	510.1	329.3	0.65	439.9	0.86	193.7	0.38	410.1	0.80	402.2	0.79	500	0.98	457.5	0.90
O75095	Multiple epidermal growth f	178.7	188.7	1.06	164.9	0.92	178.7	1.00	187.4	1.05	194.9	1.09	219.6	1.23	160.7	0.90
Q06278	Aldehyde oxidase OS=Hom	16.3	43.4	2.66	20.8	1.28	15.3	0.94	17.6	1.08	20.7	1.27	21.5	1.32	15.2	0.93
A0A0G2JS6	Mucin-4 OS=Homo sapiens															
P35606	Coatomer subunit beta' OS=	66.5	90	1.35	81.5	1.23	62.5	0.94	73.5	1.11	80.8	1.22	93.2	1.40	72.6	1.09
P16401	Histone H1.5 OS=Homo sap															
A8K8R5	Receptor protein serine/thre															
Q86SF2	N-acetylgalactosaminyltrans															
Q96JF0	Beta-galactoside alpha-2,6-s	148	105.2	0.71	120.9	0.82	73.2	0.49	126.2	0.85	123.8	0.84	134.8	0.91	99.8	0.67
Q9Y3E7	Charged multivesicular body															
B3KUE5	Phospholipid transfer protei	232	221.8	0.96	269.5	1.16	156.5	0.67	250.5	1.08	233.3	1.01	235.6	1.02	237.2	1.02
Q68DC2	Ankyrin repeat and SAM do	647	910.8	1.41	609.8	0.94	706.6	1.09	1120.5	1.73	1042.4	1.61	634.6	0.98	748.4	1.16
O60437	Periplakin OS=Homo sapier	13.4	15.7	1.17	15.8	1.18	9.9	0.74	9	0.67	13.1	0.98	14.6	1.09	14.4	1.07
Q96IY4	Carboxypeptidase B2 OS=H	27.9	46.5	1.67	32.7	1.17	42	1.51	40.9	1.47	34.9	1.25	53.3	1.91	41	1.47
O60241	Adhesion G protein-coupled															

P13686	Tartrate-resistant acid phosp															
Q93045	Stathmin-2 OS=Homo sapie															
Q6IPH7	RPL14 protein OS=Homo s															
Q8N0V5	N-acetyllactosaminide beta-	15.5	23	1.48	22.7	1.46	17.1	1.10	23.8	1.54	25.7	1.66	19.9	1.28	16.4	1.06
Q9UK23	N-acetylglucosamine-1-phos	148.4	164.9	1.11	155.5	1.05	155.1	1.05	158.2	1.07	151.2	1.02	159.5	1.07	132.9	0.90
Q05D71	TNFRSF10C protein (Fragn	615.2	463.1	0.75	402.3	0.65	316.9	0.52	560.5	0.91	547.4	0.89	541.1	0.88	565.9	0.92
A0A096LNU	Uncharacterized protein OS	272.3	257.3	0.94	160.6	0.59	152.7	0.56	267	0.98	198.8	0.73	225.7	0.83	151	0.55
Q9UHG3	Prenylcysteine oxidase 1 OS	96.8	93.5	0.97	91.2	0.94	76.7	0.79	88	0.91	86.7	0.90	88.3	0.91	85.8	0.89
Q96D15	Reticulocalbin-3 OS=Homo	394.9	439.9	1.11	329.7	0.83	295.4	0.75	381.3	0.97	436.7	1.11	413.3	1.05	362.4	0.92
A0A0X9T0I	IBM-A2 light chain variable	68	70.5	1.04	67.8	1.00	53.6	0.79	55.9	0.82	64.1	0.94	60.7	0.89	92.6	1.36
Q9H477	Ribokinase OS=Homo sapie	22.4	31.2	1.39	15.1	0.67	13	0.58	16	0.71	24	1.07	22.8	1.02	16.4	0.73
B3KV71	cDNA FLJ16199 fis, clone	45.8	79.2	1.73	54	1.18	137.5	3.00	60.9	1.33	44.3	0.97	55.2	1.21	67.7	1.48
Q6UWP8	Suprabasin OS=Homo sapie	321.2	322.1	1.00	431.8	1.34	235.8	0.73	493.5	1.54	343.8	1.07	357.5	1.11	311.2	0.97
B2R736	cDNA, FLJ93263, highly si	75	100.6	1.34	86.3	1.15	53.9	0.72	74.3	0.99	80.8	1.08	103.4	1.38	78.4	1.05
Q9P2B2	Prostaglandin F2 receptor n	139.1	151.1	1.09	136.9	0.98	192.4	1.38	141.8	1.02	142.8	1.03	165	1.19	119.9	0.86
A2J1N7	Rheumatoid factor RF-ET1	266.9	244.2	0.91	264.7	0.99	236	0.88	253	0.95	250.6	0.94	244.8	0.92	276.4	1.04
P12319	High affinity immunoglobul	127.2	107.2	0.84	73.3	0.58	94.3	0.74	99.6	0.78	98.7	0.78	104.8	0.82	95.3	0.75
B3KQP7	cDNA FLJ90843 fis, clone	105.3	94	0.89	94.9	0.90	122	1.16	95.6	0.91	94.1	0.89	166.6	1.58	113.1	1.07
A0A193CHI	10E8 light chain variable re															
Q5JU67	Cilia- and flagella-associate	31.8	41.2	1.30	40	1.26	36.5	1.15	42.3	1.33	36	1.13	39.4	1.24	42.7	1.34
B1PZN7	MHC class I antigen OS=H															
P09958	Furin OS=Homo sapiens GN															
Q6YHK3	CD109 antigen OS=Homo s															
B0LPF3	Growth factor receptor-bour															
Q9BZZ2	Sialoadhesin OS=Homo sap	111.3	101.5	0.91	120.2	1.08	93.5	0.84	101.2	0.91	107.5	0.97	104.6	0.94	89.1	0.80
Q53FP0	Pyridoxine 5'-phosphate oxi	24.6	35.5	1.44	30.2	1.23	26.9	1.09	27.5	1.12	41.4	1.68	30.4	1.24	33.1	1.35
A0A024R0T	Apolipoprotein C-I, isoform	52	95.6	1.84	75.6	1.45	74	1.42	57.9	1.11	71.5	1.38	95.9	1.84	71.2	1.37
A0A024RA	Endothelin converting enzy	207.5	166.3	0.80	175.3	0.84	120.2	0.58	174.5	0.84	180.5	0.87	154.8	0.75	182.9	0.88
P04424	Argininosuccinate lyase OS	106.8	125.4	1.17	75.4	0.71	121.3	1.14	103.6	0.97	133.6	1.25	87.1	0.82	110	1.03
Q6ZWJ1	Syntaxin-binding protein 4															
A0A024RD	Integrin-binding sialoprotei															
P20142	Gastricsin OS=Homo sapie	78.6	107.5	1.37	137	1.74	62.2	0.79	150.6	1.92	96.6	1.23	71	0.90	91.8	1.17
Q92542	Nicastrin OS=Homo sapien	35.1	50.6	1.44	32.3	0.92	29.2	0.83	37.5	1.07	37.2	1.06	37.7	1.07	31	0.88
Q9Y2G1	Myelin regulatory factor OS	91.9	108.2	1.18	99	1.08	77	0.84	81.1	0.88	90.1	0.98	85.7	0.93	74.5	0.81
B3KTQ4	Hexosyltransferase OS=Ho	107.7	184.1	1.71	153.1	1.42	210.4	1.95	144.9	1.35	125.8	1.17	147.3	1.37	122.5	1.14
B6V6K6	Mutant myocilin OS=Homo	251.9	273.1	1.08	434.3	1.72	230.1	0.91	302.4	1.20	342.3	1.36	270.8	1.08	270.1	1.07
B2R960	cDNA, FLJ94230, highly si	184.8	162.8	0.88	186.9	1.01	112.4	0.61	160.4	0.87	164.5	0.89	183.7	0.99	151	0.82
B4E3I3	cDNA FLJ59614, highly sin	61.1	91.9	1.50	67.7	1.11	66.8	1.09	61.4	1.00	62.7	1.03	64.8	1.06	53.7	0.88
Q14703	Membrane-bound transcript															
P23471	Receptor-type tyrosine-prot	29.3	33.9	1.16	26.3	0.90	35.7	1.22	35.2	1.20	29.6	1.01	28.6	0.98	24.3	0.83
Q6P4E1	Protein CASC4 OS=Homo															
A0A087WX	ST3 beta-galactoside alpha-	45.8	62.4	1.36	35.8	0.78	72.8	1.59	40.3	0.88	56.4	1.23	47.6	1.04	42.7	0.93
E7EV01	Calpain-5 OS=Homo sapien															
J3QR18	DNA topoisomerase OS=H															
P05162	Galectin-2 OS=Homo sapie															
B4DI63	cDNA FLJ59205, highly sin	151.7	163.4	1.08	242.1	1.60	171.3	1.13	178.4	1.18	172.3	1.14	186.1	1.23	162.8	1.07
P15428	15-hydroxyprostaglandin de	22.7	20	0.88	20.2	0.89	13.2	0.58	18.4	0.81	20.9	0.92	23.6	1.04	18	0.79
P35625	Metalloproteinase inhibitor	269.6	280.1	1.04	280.9	1.04	314	1.16	325.4	1.21	298.7	1.11	320.3	1.19	290	1.08
A0A024R1Y	Follistatin-like 3 (Secreted g															
Q96FN5	Kinesin-like protein KIF12															
P62701	40S ribosomal protein S4, X	83.2	93.9	1.13	84.3	1.01	66.1	0.79	83.4	1.00	87.5	1.05	96	1.15	82.7	0.99
J3QSE8	Fanconi anemia group J prot															
P15104	Glutamine synthetase OS=H	26.4	40.6	1.54	31.8	1.20	16.7	0.63	44.4	1.68	34.3	1.30	39.4	1.49	58.3	2.21
K7EJ20	Glutathione peroxidase OS=	60.1	102.6	1.71	88	1.46	73.2	1.22	81.5	1.36	84.1	1.40	74.9	1.25	62.5	1.04
Q8N5I2	Arrestin domain-containing															
F5H056	4F2 cell-surface antigen hea	61.4	70.2	1.14	62.3	1.01	58.1	0.95	69.9	1.14	61.8	1.01	65.6	1.07	50.8	0.83
Q13183	Solute carrier family 13 mer	82.4	89.1	1.08	64.2	0.78	67.7	0.82	74.1	0.90	85.8	1.04	78.8	0.96	70.6	0.86
Q9BYW2	Histone-lysine N-methyltran															

B3KUZ7	cDNA FLJ40986 fis, clone U	86.5	118.1	1.37	102.8	1.19	83.6	0.97	90.2	1.04	122.5	1.42	128.6	1.49	71.3	0.82
Q9BZJ0	Crooked neck-like protein 1															
Q5FWE3	Proline-rich transmembrane															
P35318	ADM OS=Homo sapiens Gl															
Q86TD4	Sarcalumenin OS=Homo sa	61.5	99.6	1.62	93.1	1.51	59.7	0.97	76.3	1.24	69.1	1.12	88	1.43	81.3	1.32
Q09328	Alpha-1,6-mannosylglycopr	100.2	129.8	1.30	117.4	1.17	108	1.08	112.7	1.12	140.3	1.40	136.2	1.36	102.3	1.02
B3KM35	cDNA FLJ10144 fis, clone l															
O60637	Tetraspanin-3 OS=Homo sa	341.4	395.2	1.16	268.3	0.79	289	0.85	348.9	1.02	353.3	1.03	333.7	0.98	300.9	0.88
A0A024RB(	Kit ligand OS=Homo sapier	39.5	39.5	1.00	44.8	1.13	29.4	0.74	36.3	0.92	38.1	0.96	44.1	1.12	41.1	1.04
A0A140VJL	Testicular tissue protein Li 9	15.8	17.9	1.13	12.6	0.80	15.9	1.01	13.5	0.85	17.9	1.13	19.4	1.23	14.6	0.92
Q9H9V9	JmjC domain-containing pro															
A8K690	cDNA FLJ76863, highly sin	142.3	172.5	1.21	157.4	1.11	139.5	0.98	157.4	1.11	163.5	1.15	170.8	1.20	138.2	0.97
B7Z7X3	cDNA FLJ51770, highly sin	538.2	484.1	0.90	715.3	1.33	269	0.50	437	0.81	486.7	0.90	602.3	1.12	547.3	1.02
A0A0G2JNI	Leukocyte immunoglobulin-															
B7ZLW2	HELZ protein OS=Homo sa	24.3	15.4	0.63	18.6	0.77	16.7	0.69	23.1	0.95	18.1	0.74	14.5	0.60	12.8	0.53
P48547	Potassium voltage-gated cha	19	25.6	1.35	21.6	1.14	17.5	0.92	20	1.05	26.7	1.41	30	1.58	31	1.63
O14618	Copper chaperone for supero															
H3BSR6	Fractalkine OS=Homo sapie	81.4	101.7	1.25	88.8	1.09	178.5	2.19	92.8	1.14	99.7	1.22	83.8	1.03	90.2	1.11
A6NKB5	Pecanex-like protein 2 OS=I															
Q8N436	Inactive carboxypeptidase-li															
Q6UWL6	Kin of IRRE-like protein 2 (	13.9	17	1.22	13.8	0.99	12.7	0.91	12.9	0.93	12.6	0.91	14.5	1.04	12.9	0.93
Q92618	Zinc finger protein 516 OS=															
H9STE0	Cytochrome c oxidase subur	204.9	198.6	0.97	183.3	0.89	146.3	0.71	207.7	1.01	168.9	0.82	233.6	1.14	183.2	0.89
Q96NL6	Sodium channel and clathrin	199.9	297.6	1.49	364.1	1.82	401.2	2.01	291.1	1.46	255.7	1.28	212.8	1.06	257.4	1.29
B2RDG0	Proteasome subunit alpha ty															
B4DPQ3	cDNA FLJ51034, highly sin	21.5	28.9	1.34	20.1	0.93	16.1	0.75	24.1	1.12	23	1.07	24.7	1.15	21.3	0.99
O43175	D-3-phosphoglycerate dehyd															
H9KV90	SH3 and multiple ankyrin re	24.1	32.4	1.34	59.1	2.45	19.8	0.82	34.2	1.42	26.4	1.10	25.5	1.06	25.7	1.07
Q6ZMJ2	Scavenger receptor class A r	63.4	80	1.26	55.5	0.88	57.2	0.90	59.4	0.94	60.6	0.96	63.7	1.00	55.6	0.88
Q9BV40	Vesicle-associated membrar															
A0A0A0MT	Coiled-coil domain-containi	202.2	281.2	1.39	262.2	1.30	280.7	1.39	293.6	1.45	264.5	1.31	208	1.03	192.8	0.95
A0A024R8A	Ubiquitinyl hydrolase 1 OS=	41.4	112.1	2.71	107.2	2.59	78.8	1.90	69.7	1.68	85.9	2.07	42.1	1.02	105.9	2.56
O15162	Phospholipid scramblase 1 (	154.9	205.7	1.33	163.4	1.05	106.6	0.69	181.5	1.17	210.2	1.36	132.2	0.85	135	0.87
A0A0A0MT	TATA-binding protein-asso	119.7	150.2	1.25	175.6	1.47	91.6	0.77	143.8	1.20	122.6	1.02	121.4	1.01	128.8	1.08
A6NFX8	ADP-sugar pyrophosphatase	29.5	46.1	1.56	39.3	1.33	35.6	1.21	37.6	1.27	40.7	1.38	36.5	1.24	34.2	1.16
Q2M204	HCG1642804, isoform CRA															
H7BXI7	Ly6/PLAUR domain-contai	26.6	25.9	0.97	23.7	0.89	18.6	0.70	31.7	1.19	27.9	1.05	23.4	0.88	30.1	1.13
Q9UKR3	Kallikrein-13 OS=Homo sap															
D6RH31	Nephronectin (Fragment) O	231.7	184	0.79	165.7	0.72	166	0.72	194.7	0.84	211.6	0.91	215.6	0.93	190.8	0.82
O60662	Kelch-like protein 41 OS=H	188	206.4	1.10	201.9	1.07	144.9	0.77	184.8	0.98	165.7	0.88	83.2	0.44	100.6	0.54
A0A087WU	C-C motif chemokine 16 OS															
Q5HYA8	Meckelin OS=Homo sapiens	21.4	32.6	1.52	60.4	2.82	41.1	1.92	38.3	1.79	27.6	1.29	23.4	1.09	20.2	0.94
Q96AM1	Mas-related G-protein coupl	34.8	27.7	0.80	26.4	0.76	25.6	0.74	31.8	0.91	32.9	0.95	30.5	0.88	29.3	0.84
B3KQQ3	cDNA PSEC0016 fis, clone	59.6	71.9	1.21	51.7	0.87	60.1	1.01	64.1	1.08	70.2	1.18	82	1.38	62.3	1.05
Q9BQG1	Synaptotagmin-3 OS=Homo															
O95445	Apolipoprotein M OS=Hom	354.3	331.8	0.94	428.4	1.21	231.7	0.65	343.3	0.97	397.3	1.12	419.1	1.18	288	0.81
