# Supplementary material for: LC-MS/MS-Based Quantitative Proteomics Analysis of Different Stages of Non-Small-Cell Lung Cancer
Source: Biomed Res Int. 2021 Feb 26;2021:5561569. doi: 10.1155/2021/5561569 (PMC7937045; doi:10.1155/2021/5561569)
Supplement: Supplementary 4 — Table S4: a further study on the LC-MS analysis results of serum in Table S1. Serum samples from healthy controls were compared to serum samples from different lung adenocarcinoma groups to obtain differential protein levels. [file 5561569.f4.pdf]

Uniprot Acc	proteins	121	113	ratio(113/12	114	ratio(114/12	115	ratio(115/12	116	ratio(116/12	117	ratio(117/12	118	ratio(118/12	119	ratio(119/12
C0JYY2	Apolipoprot	510456.9	587089.8	1.15	722432.2	1.42	577726.5	1.13	553022.3	1.08	631905.3	1.24	553297.9	1.08	487383.6	0.95
V9HWA9	Epididymis	1086362.4	1038495.5	0.96	987768	0.91	1014543.4	0.93	1059000	0.97	1036688.9	0.95	1058344.2	0.97	1125990.4	1.04
A0A140TA	Complement	10069.7	10298.9	1.02	11246.3	1.12	10190.4	1.01	9787.3	0.97	10555.8	1.05	9545.9	0.95	7018.6	0.70
A0A140TA	Complement C4-A OS=Homo sapiens GN=C4A PE=1 SV=1															
P0C0L4	Complement	379640.7	354515.7	0.93	358550.6	0.94	353533.8	0.93	371096.1	0.98	369917.8	0.97	392042.8	1.03	352593.5	0.93
A8K5A4	cDNA FLJ7	181927.1	184669.2	1.02	167773	0.92	172794.9	0.95	189477.9	1.04	181011.4	0.99	192480.9	1.06	188787.6	1.04
P08603	Complement	127138.9	139326.8	1.10	138368.7	1.09	152055.5	1.20	147415.7	1.16	134161.2	1.06	124277.4	0.98	143670	1.13
A8K5T0	cDNA FLJ7	1595.6	2166.5	1.36	1478.4	0.93	1667	1.04	1764.5	1.11	1600.3	1.00	1443.2	0.90	1747.7	1.10
Q7Z7Q0	APOB prote	369.3	378.4		434.3	1.18	176	0.48	338.2	0.92	290.4	0.79	163	0.44	248.4	0.67
E9PFZ2	Ceruloplasm	232	305.6	1.32	1344.1	5.79	340.1	1.47	294.9	1.27	245.3	1.06	225.6	0.97	226.5	0.98
P01023	Alpha-2-ma	155893	159242.4	1.02	150022.1	0.96	148585.9	0.95	127199.8	0.82	139287.4	0.89	196290.4	1.26	150833.5	0.97
B4E1Z4	cDNA FLJ5	179991.2	168887.5	0.94	175330.4	0.97	170379.3	0.95	181366.8	1.01	186553.2	1.04	182496	1.01	179532.4	1.00
P02768	Serum albun	374998.6	393627.8	1.05	415295.5	1.11	553515.2	1.48	401168.8	1.07	313800.8	0.84	273544.9	0.73	216497.6	0.58
P00747	Plasminoger	110712.8	123328	1.11	118392.6	1.07	116615.2	1.05	117254.4	1.06	115644.8	1.04	104325.5	0.94	105844.6	0.96
P02790	Hemopexin	407277.3	374618.6	0.92	386591	0.95	389342.2	0.96	396054.9	0.97	408398.8	1.00	392817.7	0.96	388013.2	0.95
P00738	Haptoglobin	185933.7	185102	1.00	162600.1	0.87	155639.9	0.84	279577.3	1.50	162525.6	0.87	258436.5	1.39	410792.3	2.21
A0A024R3I	Apolipoprot	411885.3	338583	0.82	381625.8	0.93	366565	0.89	373110.1	0.91	364223.9	0.88	375076	0.91	391751.5	0.95
P00734	Prothrombin	57305.1	69844.1	1.22	67971.2	1.19	72856.6	1.27	63897.5	1.12	65950.1	1.15	56143.8	0.98	58040.4	1.01
B4E1C2	Kininogen 1	131253.4	125717	0.96	122596.9	0.93	124775.5	0.95	130027.4	0.99	132243.9	1.01	130159.7	0.99	124968.7	0.95
P01031	Complement	93176.8	94890.7	1.02	90259.9	0.97	89862.7	0.96	87867	0.94	94067.8	1.01	91781.4	0.99	92978.4	1.00
D3DNU8	Kininogen 1	7787.9	6418.8	0.82	6744.1	0.87	6171.8	0.79	6016	0.77	6433	0.83	9059.8	1.16	8373.3	1.08
B4E1B2	cDNA FLJ5	83286.1	92039.8	1.11	81713.4	0.98	90197.9	1.08	70892.5	0.85	76534	0.92	83395	1.00	66649.1	0.80
E9KL23	Epididymis	6306.2	8613.5	1.37	5499.6	0.87	5387.8	0.85	4010.7	0.64	5925.8	0.94	6670.1	1.06	4887.9	0.78
A0A024R6I	Alpha-1-anti	165755.8	129336.8	0.78	145830.8	0.88	133687.4	0.81	163476	0.99	143911.9	0.87	158637.4	0.96	254797.8	1.54
A0A0K0K1	Epididymis	866.1	905.6	1.05	975.5	1.13	1107.8	1.28	873	1.01	818.1	0.94	1068.3	1.23	849.7	0.98
D6RF35	Vitamin D-b	203990.6	191458.5	0.94	193095.1	0.95	175613.7	0.86	171725	0.84	181511.4	0.89	200116.5	0.98	203654.9	1.00
V9HWI6	Epididymis	1938.8	1493.4	0.77	1742	0.90	1355.4	0.70	1572.2	0.81	2818.7	1.45	2236	1.15	1573.4	0.81
D3DRR6	Inter-alpha (	108810.6	108481.7	1.00	98996.5	0.91	99369.2	0.91	94019.8	0.86	105151.6	0.97	106137.5	0.98	95323.2	0.88
B2RMS9	Inter-alpha (	104677.5	108110.8	1.03	103576.1	0.99	102139.3	0.98	101346.1	0.97	109288.1	1.04	106762.4	1.02	106066.8	1.01
A8K2T4	cDNA FLJ7	34968	39819.6	1.14	35815.5	1.02	37899.4	1.08	38127.9	1.09	37936.9	1.08	36598.8	1.05	35816.4	1.02
B7Z539	cDNA FLJ5	53820	54625.6	1.01	50116.6	0.93	55433.1	1.03	58397.6	1.09	56789.4	1.06	51279.7	0.95	46599.2	0.87
P06727	Apolipoprot	132070.1	121784.2	0.92	122790	0.93	118549.5	0.90	111855.3	0.85	125959.3	0.95	118246.5	0.90	98547	0.75
P04003	C4b-binding	89101.1	98140.4	1.10	87061.7	0.98	94314.1	1.06	96031.3	1.08	95727.1	1.07	96432.8	1.08	93592.4	1.05
B2R6W1	cDNA, FLJ9	340	467.6	1.38	371.3	1.09	403.2	1.19	355.5	1.05	366.1	1.08	384.4	1.13	402.6	1.18
B4E1D8	cDNA FLJ5	356.1	419.3	1.18	405.5	1.14	393	1.10	441.7	1.24	454.7	1.28	390.7	1.10	344.8	0.97
P00739	Haptoglobin	2635.5	2606.9	0.99	2512.7	0.95	2255.3	0.86	2714.9	1.03	2471.1	0.94	3397.7	1.29	4403	1.67
B7Z1F8	cDNA FLJ5	5945	6148.8	1.03	5133	0.86	6345.8	1.07	5999.2	1.01	5232.9	0.88	5756.8	0.97	5222.4	0.88
P06396	Gelsolin OS-	53072.9	51360.2	0.97	48208.4	0.91	50016.7	0.94	46518.3	0.88	50644.9	0.95	50760.1	0.96	44495	0.84
D9IWP9	Beta-2-glyco	95095.7	95869.3	1.01	94684.3	1.00	92089.5	0.97	92442.1	0.97	97157.4	1.02	95290.3	1.00	97045.8	1.02
A0A024R94	Serpin pepti	76446.5	81924	1.07	75341.2	0.99	73519.1	0.96	66972.7	0.88	79640.7	1.04	77286.2	1.01	59152.2	0.77
B3KS79	cDNA FLJ3	99858	93851	0.94	98190.2	0.98	91704	0.92	98831.5	0.99	103081.2	1.03	95366.8	0.96	132515.7	1.33
P13671	Complement	34641.2	36577.2	1.06	33403.3	0.96	34161.9	0.99	36176	1.04	36281.2	1.05	33438.1	0.97	34559.2	1.00
E9PGN7	Plasma prote	110273.8	100248.8	0.91	112257.1	1.02	98724.4	0.90	106678.1	0.97	114992.3	1.04	110430.4	1.00	101300.1	0.92
P02760	Protein AMI	38024	40442	1.06	36134.6	0.95	39749.4	1.05	40433.6	1.06	41020	1.08	38398	1.01	34490.9	0.91
A8K8Z4	cDNA FLJ7	91.7	91.3	1.00	102.6	1.12	84.4	0.92	91.7	1.00	76	0.83	104.5	1.14	85.7	0.93
B2R8I2	cDNA, FLJ9	53687.5	56488.4	1.05	55925.9	1.04	42986.9	0.80	48963.1	0.91	56480.5	1.05	61840.2	1.15	50180	0.93

H0YAC1	Plasma kalli	33082.6	34217.8	1.03	32918	1.00	34183.6	1.03	32889.7	0.99	34399	1.04	32603.1	0.99	29661.8	0.90
P04196	Histidine-ric	661.8	866.1	1.31	992.4	1.50	590.2	0.89	622.6	0.94	688.2	1.04	799.8	1.21	706.1	1.07
P07358	Complement	25346.9	24943.9	0.98	24633.6	0.97	24581.7	0.97	24823.5	0.98	24915.6	0.98	24972.2	0.99	23582.2	0.93
P43652	Afamin OS=	60761.3	62649.1	1.03	58267.1	0.96	59622.5	0.98	58119.3	0.96	61682.8	1.02	62913.2	1.04	54287.5	0.89
P06681	Complement	5096.4	5761.7	1.13	5226.7	1.03	5861.4	1.15	5486.3	1.08	5452.7	1.07	5408.6	1.06	5137.8	1.01
P04275	von Willebra	10196	13089.5	1.28	11473.5	1.13	12553.1	1.23	13082	1.28	13206.5	1.30	13496.9	1.32	11111.4	1.09
A0A024R9(	Thrombospo	21666.3	26614.2	1.23	21742.9	1.00	25643.9	1.18	21725	1.00	27021.2	1.25	22529.8	1.04	20044.7	0.93
P07996	Thrombospo	392.8	557.4	1.42	499.6	1.27	559	1.42	467	1.19	606.6	1.54	606.1	1.54	458.5	1.17
P10909	Clusterin OS	33598.7	50095.5	1.49	45302.4	1.35	48175.9	1.43	48440.7	1.44	47018.7	1.40	34883.3	1.04	34107.1	1.02
O75882	Attractin OS	25522.8	27658.2	1.08	25777.2	1.01	25986.1	1.02	25151.2	0.99	26915.2	1.05	24672.1	0.97	23733.1	0.93
E7ETH0	Complement	26360	28620.6	1.09	26667.1	1.01	27252.9	1.03	26287.2	1.00	27657.8	1.05	25695.2	0.97	27742.1	1.05
L8E853	von Willebra															
B3KNX0	cDNA FLJ3	24353	27673.9	1.14	25972.1	1.07	24866.1	1.02	26889	1.10	25674.2	1.05	23715.2	0.97	25775	1.06
A0A024R46	Fibronectin	8653	14350.7	1.66	12920.5	1.49	17208	1.99	13008.3	1.50	18122.1	2.09	8789.5	1.02	7557	0.87
B4DPQ0	Complement	27059.9	29398.6	1.09	29623.9	1.09	30198.6	1.12	30114.2	1.11	28399.3	1.05	25563.7	0.94	28589.3	1.06
P02649	Apolipoprot	54616	60795.2	1.11	53606.3	0.98	61843.3	1.13	62405.6	1.14	66560.6	1.22	58524.1	1.07	55467.3	1.02
P07357	Complement	19863.5	21076	1.06	19665.2	0.99	20194.4	1.02	19189.5	0.97	20228.2	1.02	19921.3	1.00	19774.3	1.00
A0A024R03	Complement	31276.8	28186.5	0.90	29862.6	0.95	26992.6	0.86	31248.7	1.00	30833.4	0.99	31964.7	1.02	39963.3	1.28
Q6LAM1	Heavy chain	7000	5884.2	0.84	5922.3	0.85	5458.6	0.78	5879.3	0.84	6019	0.86	6552.1	0.94	9038.4	1.29
P05546	Heparin cofa	40235.4	42058.8	1.05	39849.4	0.99	38495	0.96	41473.5	1.03	42452	1.06	44464.2	1.11	39529.8	0.98
Q9UNU2	Complement	2086.8	2438.3	1.17	2216	1.06	2211.7	1.06	1819.1	0.87	2243	1.07	1953.8	0.94	2485	1.19
V9HWD8	Epididymis s	87354.8	85465.5	0.98	85173.4	0.98	82675.7	0.95	85248.3	0.98	87032.2	1.00	83186	0.95	82018.4	0.94
A0A0S2Z4I	Protein S iso	20335.7	20521.9	1.01	19479.9	0.96	20695.3	1.02	18904.4	0.93	20998.2	1.03	20174.6	0.99	20330.7	1.00
D9ZGG2	Vitronectin (	45241.8	47676.1	1.05	42103.8	0.93	50280.8	1.11	50872	1.12	50370.3	1.11	44782.5	0.99	40512.9	0.90
B7Z8Q2	cDNA FLJ5	78902.1	72510.6	0.92	71430.7	0.91	69072.4	0.88	68315.1	0.87	75454.7	0.96	69386.5	0.88	66683.6	0.85
D9YZU5	Beta-globin	26416.6	26762	1.01	25508.7	0.97	21785.1	0.82	27519.4	1.04	20787.3	0.79	30161.5	1.14	30166.4	1.14
B7Z8B6	cDNA FLJ5	3715.2	4851.3	1.31	3182.4	0.86	3426.6	0.92	2914.7	0.78	3116.9	0.84	4486.4	1.21	3978.8	1.07
A0A1B0GU	Ig mu chain	41758.8	33261.6	0.80	33963.5	0.81	33291.6	0.80	31512.1	0.75	39637.9	0.95	55273.6	1.32	42527.6	1.02
B2R950	cDNA, FLJ9	2893	2645.4	0.91	2669.2	0.92	2807.5	0.97	2688	0.93	3024	1.05	2831.3	0.98	3108.6	1.07
Q96PD5	N-acetylmur	18131.8	20449.1	1.13	16845.4	0.93	17634.1	0.97	17893.7	0.99	18152.3	1.00	17621.4	0.97	16026.7	0.88
P02763	Alpha-1-acid	21588.2	20978.4	0.97	23424.7	1.09	17359	0.80	26223.1	1.21	21535.6	1.00	24203.1	1.12	45114	2.09
B0AZL7	cDNA, FLJ7	18319.3	22324.3	1.22	19098.3	1.04	20124.5	1.10	19414.7	1.06	21036	1.15	19426.1	1.06	16382.5	0.89
B2R815	cDNA, FLJ9	18523.7	20326.1	1.10	19314.7	1.04	17136.1	0.93	15861.9	0.86	19134.1	1.03	18435.3	1.00	13815.6	0.75
P08697	Alpha-2-anti	16208.5	15568.9	0.96	15471.1	0.95	16057.6	0.99	15651.7	0.97	15778.6	0.97	15332.5	0.95	15477.2	0.95
P25311	Zinc-alpha-2	22823.9	24197	1.06	22776.3	1.00	22025	0.96	20191.2	0.88	24868.6	1.09	21172.2	0.93	17749	0.78
Q86TT1	Full-length c	628.3	731.5	1.16	612.5	0.97	656.3	1.04	623.5	0.99	686.7	1.09	818	1.30	646.6	1.03
V9HW68	Epididymis l	14318	14200.7	0.99	13471.4	0.94	14091.7	0.98	14781.3	1.03	14841.2	1.04	12780.1	0.89	15179.6	1.06
Q06033	Inter-alpha-t	12468.4	13567.8	1.09	12959.8	1.04	12149.5	0.97	12249.6	0.98	12309.6	0.99	12294.9	0.99	12842.5	1.03
P80108	Phosphatidy	9864.1	11082	1.12	10784.2	1.09	10919.1	1.11	10552.3	1.07	10448.7	1.06	10629.6	1.08	9586.6	0.97
P07360	Complement	10063.5	10529.2	1.05	9986.8	0.99	10975.9	1.09	10507.7	1.04	10028.2	1.00	10438.8	1.04	9234.4	0.92
P22792	Carboxypept	16767.1	18835.2	1.12	18865.2	1.13	17056.8	1.02	16192.8	0.97	17940.2	1.07	16797.5	1.00	14238.6	0.85
A0A140VK	Testis tissue	22611	25131.6	1.11	22440.4	0.99	22366.6	0.99	20490.3	0.91	23660.7	1.05	22065.7	0.98	21306.7	0.94
P51884	Lumican OS	21478.9	21871.6	1.02	20178.2	0.94	20998.2	0.98	20168.7	0.94	21854.5	1.02	20957.9	0.98	18878.5	0.88
A0A0A0MF	Coagulation	6480.2	8192.9	1.26	7006.1	1.08	7385.3	1.14	6954.2	1.07	8063.6	1.24	6384.5	0.99	6217.3	0.96
Q59EP2	Angiotensin	24089.4	24341.2	1.01	23979.5	1.00	23177.3	0.96	23539.2	0.98	25181.7	1.05	23425	0.97	22223.6	0.92
B2R582	cDNA, FLJ9	798	921.9	1.16	782	0.98	830.3	1.04	714.9	0.90	798.1	1.00	808.3	1.01	819.5	1.03
Q4TZM4	Hemoglobin	1332.1	1405	1.05	1277.4	0.96	979.5	0.74	1322.2	0.99	935.1	0.70	1697.9	1.27	1700.3	1.28

Q6MZQ6	Putative unc															
P27169	Serum parac	26425.9	24841.5	0.94	26246	0.99	25042.9	0.95	24302	0.92	27227.6	1.03	26242.8	0.99	22453.9	0.85
D6RF20	Vitamin D-b	24.1	28.9	1.20	18.6	0.77	31.5	1.31	25.4	1.05	23.9	0.99	27.1	1.12	21.2	0.88
Q96K68	cDNA FLJ1	9509.7	9530.9	1.00	9767.8	1.03	10313.7	1.08	9694.6	1.02	9181.8	0.97	9114	0.96	12195.3	1.28
G3XAK1	Hepatocyte g	6404.4	6901.6	1.08	6318.4	0.99	6943.5	1.08	6491.8	1.01	6537.2	1.02	5956.3	0.93	5849.2	0.91
A0A024R2(	C-type lectir	7606	8337.9	1.10	7454.1	0.98	7456.1	0.98	6832.4	0.90	8007.7	1.05	7034	0.92	6269.8	0.82
B2R9F2	cDNA, FLJ9	21182.2	24324.2	1.15	24773.4	1.17	18653.9	0.88	20418.2	0.96	23084.3	1.09	23221.8	1.10	21849.1	1.03
P05543	Thyroxine-b	10522.1	10969	1.04	11223.3	1.07	10323.5	0.98	10070	0.96	11408.6	1.08	11465.7	1.09	10219.8	0.97
A0N071	Delta globin	110.6	124.5	1.13	109	0.99	104.9	0.95	128.9	1.17	97.3	0.88	127.9	1.16	133	1.20
P02753	Retinol-bind	33157.6	34673.7	1.05	31209.4	0.94	35599.5	1.07	34712.9	1.05	35446.5	1.07	33435.2	1.01	27320.1	0.82
C9JF17	Apolipoprote	24386.8	26206.3	1.07	26020.4	1.07	27458.5	1.13	23217.7	0.95	27609.1	1.13	23713.6	0.97	21141.3	0.87
A8K1K1	cDNA FLJ7	6742.3	7542.4	1.12	7262.6	1.08	7434.9	1.10	6611.6	0.98	6953.1	1.03	6538.6	0.97	5646.1	0.84
A0A1K0GX	Globin C1 C	14723.1	14157.8	0.96	14141.1	0.96	9465.6	0.64	14883	1.01	10905.9	0.74	15034.8	1.02	18249.7	1.24
D6RAR4	Hepatocyte g	76.6	88.7	1.16	66.5	0.87	89.3	1.17	80.3	1.05	82.1	1.07	88.3	1.15	60.3	0.79
Q16610	Extracellular	4476.7	4777.3	1.07	4009.6	0.90	4909.4	1.10	4730.8	1.06	4439.4	0.99	4352.6	0.97	3766.9	0.84
Q04756	Hepatocyte g	6012.3	6577.1	1.09	5836.5	0.97	6665.3	1.11	5873.9	0.98	6133.9	1.02	5968.9	0.99	5362	0.89
Q8TCD0	Uncharacter	466.9	415.3	0.89	418.3	0.90	400.5	0.86	341.9	0.73	406.3	0.87	519.1	1.11	440.6	0.94
P03951	Coagulation	4345.1	5192.9	1.20	4314.6	0.99	4788.6	1.10	4719.5	1.09	4850.2	1.12	4739	1.09	4267.9	0.98
Q03591	Complement	1223.8	1251.5	1.02	1403.4	1.15	1466.7	1.20	1296.1	1.06	1136.9	0.93	1164.7	0.95	1201	0.98
A0A0S2Z3Y	Lectin galac	6950.6	7320.9	1.05	6436	0.93	6925.7	1.00	6615.1	0.95	6572.9	0.95	8034.7	1.16	8214.9	1.18
Q6PIL8	IGK@ prote	7153.1	7606.5	1.06	6323.7	0.88	5824.9	0.81	5681	0.79	5845.4	0.82	8047.9	1.13	8245.1	1.15
P02746	Complement	8644	8778.3	1.02	8648.3	1.00	9690.7	1.12	9963.4	1.15	8865	1.03	8777.6	1.02	8280.1	0.96
A0A096LPE	Protein SAA	10462.9	10688.6	1.02	10717.2	1.02	11896	1.14	12038.4	1.15	11679.4	1.12	11065.4	1.06	11844.1	1.13
Q6MZU6	Putative unc	3853.2	4359	1.13	3634.2	0.94	4226.4	1.10	4274.9	1.11	4095.3	1.06	3280.3	0.85	3843.7	1.00
P02750	Leucine-rich	20434.1	19280.5	0.94	19204.6	0.94	19544.7	0.96	22329.2	1.09	19626.4	0.96	21509.2	1.05	27178.7	1.33
P48740	Mannan-bin	5616.9	7116.6	1.27	6236	1.11	6539.9	1.16	6207.5	1.11	6035.4	1.07	6160.2	1.10	5467.1	0.97
P02775	Platelet basi	15640.4	17782.9	1.14	15010.3	0.96	17397.2	1.11	17472.2	1.12	19286.4	1.23	15447.8	0.99	15413.3	0.99
A6XNE2	Complement	3098.3	3661.6	1.18	3079	0.99	3909.2	1.26	4144.5	1.34	3542.2	1.14	3244.9	1.05	2871.5	0.93
P02747	Complement	8282.3	8248.2	1.00	8431.2	1.02	8152.3	0.98	8062.2	0.97	7885.1	0.95	7807.7	0.94	8072.6	0.97
P15169	Carboxypept	6584.9	6941.6	1.05	6477.7	0.98	7257	1.10	7312.7	1.11	7140.8	1.08	6848.2	1.04	6112.5	0.93
P00742	Coagulation	8124.9	8976.6	1.10	8245.7	1.01	8527.9	1.05	8393.9	1.03	8808	1.08	7701.3	0.95	7101.8	0.87
P19652	Alpha-1-acid	19263.5	19165	0.99	20571.4	1.07	17406.3	0.90	19854.6	1.03	18726.5	0.97	18554.9	0.96	27901.1	1.45
Q14520	Hyaluronan-	11039.1	11316.7	1.03	10532.6	0.95	11084.5	1.00	11173.5	1.01	11337.9	1.03	10612	0.96	10238.1	0.93
F2RM37	Coagulation	6388.3	6699.2	1.05	6153.5	0.96	6191.1	0.97	6563.2	1.03	6383.7	1.00	6621.1	1.04	6072.5	0.95
Q96IY4	Carboxypept	11789.6	12269.4	1.04	11550.9	0.98	13003.2	1.10	12811.5	1.09	12370	1.05	12796.3	1.09	10423.8	0.88
A2NUT2	Lambda-cha	14755.2	15187.4	1.03	14275.1	0.97	15722.5	1.07	15169.7	1.03	16572.9	1.12	14111.3	0.96	15719	1.07
O75636	Ficolin-3 OS	15367.2	17638.8	1.15	18165.6	1.18	16665	1.08	14385.8	0.94	16004	1.04	16769.9	1.09	14579.7	0.95
Q9NPP6	Immunoglob	439.2	454.8	1.04	457.1	1.04	412.1	0.94	401.7	0.91	470.5	1.07	460.3	1.05	526.6	1.20
Q567P1	IGL@ protei	1174.7	1092.4	0.93	1442.3	1.23	1467.9	1.25	1287.8	1.10	1469.6	1.25	959	0.82	1225	1.04
A0A024R6N	Serpin pepti	3529	4175.3	1.18	3766.7	1.07	4461.6	1.26	4485.9	1.27	4064	1.15	4083.4	1.16	2912.8	0.83
H6VRG1	Keratin 1 OS	2159.3	3451.6	1.60	3228.1	1.49	2797.8	1.30	3184.2	1.47	3565.9	1.65	2341.6	1.08	2726	1.26
A0A0R7FJE	Coagulation	2366.7	2998.3	1.27	2976.7	1.26	2986.4	1.26	2883.8	1.22	2602.2	1.10	2378.2	1.00	2213.3	0.94
P20851	C4b-binding	8738.8	9027.5	1.03	8504	0.97	9754.4	1.12	9465.3	1.08	9543.2	1.09	9385.3	1.07	9386.6	1.07
P63261	Actin, cytop	3679	5034.2	1.37	4421.8	1.20	4569	1.24	4498.6	1.22	4666.4	1.27	4301.8	1.17	3602.9	0.98
S6AWE6	IgG L chain	443.9	446.9	1.01	440.7	0.99	498.9	1.12	495.6	1.12	479.3	1.08	490	1.10	532	1.20
A0A024R07	Apolipoprote	20202.7	21308.9	1.05	26544.7	1.31	20263.1	1.00	17356.9	0.86	22290.7	1.10	21967.1	1.09	21581	1.07
O00391	Sulfhydryl o	2526.4	3092.7	1.22	2603.2	1.03	2911.4	1.15	2915	1.15	2811.2	1.11	2625.9	1.04	2246.1	0.89

P02743	Serum amyl	12930.8	13581.8	1.05	12697.6	0.98	13609.2	1.05	12915.9	1.00	14808.5	1.15	11096.7	0.86	9575.8	0.74
P02776	Platelet facto	7708.1	7328	0.95	14120.9	1.83	14224.7	1.85	9230.1	1.20	9519.2	1.23	9673.7	1.26	7847.5	1.02
P09172	Dopamine b	2072.2	2206.3	1.06	2263.1	1.09	2213	1.07	2303	1.11	1895.8	0.91	2180.4	1.05	1778.4	0.86
P43251	Biotinidase (	3353.8	4160.3	1.24	3812.2	1.14	3998.6	1.19	3618	1.08	3739.1	1.11	3523.6	1.05	2741.5	0.82
P17936	Insulin-like g	5061.2	5551.8	1.10	4713.9	0.93	4955.8	0.98	5015.6	0.99	5662.5	1.12	5154.3	1.02	4313.3	0.85
A6XGL1	Transthyretin	6699.7	7120.2	1.06	5982.7	0.89	6096.6	0.91	5738.3	0.86	6744	1.01	7894.5	1.18	6887.9	1.03
V9GYM3	Apolipoprote	58257.6	44115.6	0.76	54074.9	0.93	48466.6	0.83	40529.6	0.70	47555	0.82	51115.9	0.88	44102	0.76
B4DPQ3	cDNA FLJ5	3019.2	3156	1.05	2875.5	0.95	3008.6	1.00	3181.3	1.05	3251	1.08	3124.7	1.03	2837.3	0.94
Q1HP67	Lipoprotein,	4008	3572.9	0.89	3229.5	0.81	5065.3	1.26	3792.2	0.95	3698.1	0.92	3973.9	0.99	3843.8	0.96
B2R888	Monocyte di	1767.4	2126.2	1.20	1838	1.04	2245	1.27	2263	1.28	2124.1	1.20	1927.7	1.09	1746.3	0.99
Q6GMV7	Uncharacteri	874.1	719.1	0.82	817	0.93	835.9	0.96	834.4	0.95	988	1.13	992.9	1.14	973.6	1.11
P13645	Keratin, type	4187.6	6299.9	1.50	5141.4	1.23	5083.2	1.21	5122.4	1.22	5879.9	1.40	4691.1	1.12	4037.6	0.96
A0A024R1(	Apolipoprote	5734.4	6244	1.09	5517.6	0.96	7231.8	1.26	5956.6	1.04	5785.2	1.01	6265	1.09	6103	1.06
P36980	Complement	655.8	697.3	1.06	723	1.10	663.2	1.01	683.4	1.04	651.3	0.99	658.5	1.00	640.3	0.98
O95445	Apolipoprote	8414.8	8296.5	0.99	9042	1.07	8840.9	1.05	8126.8	0.97	8617.6	1.02	8163.2	0.97	7562.5	0.90
Q6MZX7	Putative unc	1228.3	1342.6	1.09	1183.7	0.96	1099.8	0.90	1043.5	0.85	1109.7	0.90	938.7	0.76	1199.3	0.98
P22352	Glutathione	9294	10412	1.12	9504.2	1.02	9810.5	1.06	9756.1	1.05	9806.6	1.06	9337.8	1.00	8045.1	0.87
P04278	Sex hormone	2653.1	3130.4	1.18	2779.4	1.05	2783.5	1.05	2998.7	1.13	2665.9	1.00	2874.3	1.08	2493.8	0.94
K7ER74	Protein APC	13980.3	13740.2	0.98	12868.2	0.92	16333.2	1.17	13350.9	0.95	13390.5	0.96	13986.8	1.00	16334.8	1.17
Q8NF17	FLJ00385 pr	242.4	320.1	1.32	247.7	1.02	331.5	1.37	279.1	1.15	251.7	1.04	370.4	1.53	292.4	1.21
S6BGE0	IgG H chain															
A0A0S2Z4I	Complement	3322.3	4095.2	1.23	3644.8	1.10	3830.2	1.15	3920.4	1.18	4101.1	1.23	4187.3	1.26	3549.1	1.07
D3DNN4	Carboxylic e	5130.5	5064.1	0.99	5240.3	1.02	5522.4	1.08	4966.8	0.97	5218	1.02	4797.5	0.94	4317.1	0.84
A0A161I20	Lactoferrin (	879.4	1147.8	1.31	1147.8	1.31	1211.5	1.38	1145.7	1.30	1138.4	1.29	1107.8	1.26	981.9	1.12
P00915	Carbonic an	2002.4	2162.4	1.08	2609.2	1.30	1957.8	0.98	2055.7	1.03	1812.4	0.91	2027.6	1.01	2103.9	1.05
Q9UGM5	Fetuin-B OS	4107.9	4351.3	1.06	3769.4	0.92	4123.9	1.00	4226.1	1.03	4171.8	1.02	4422.2	1.08	3868.4	0.94
A0A0F7G8J	Plasminogen															
Q8TCZ8	Apolipoprote	380.9	476.4	1.25	343.4	0.90	456.2	1.20	890	2.34	571.4	1.50	559.4	1.47	698.9	1.83
P11226	Mannose-bin	1549.9	2010.3	1.30	1675.5	1.08	1836.5	1.18	1840.5	1.19	1926	1.24	1449.9	0.94	1492.7	0.96
P14780	Matrix meta	1462.7	1945.1	1.33	1718.1	1.17	1892.8	1.29	1825.1	1.25	1878.1	1.28	1733.5	1.19	1533.9	1.05
Q9NZP8	Complement	2150.2	2606.4	1.21	2149.6	1.00	2431	1.13	2787.5	1.30	2422.7	1.13	2219.6	1.03	2302	1.07
A8K3K1	cDNA FLJ7	62.2	82.1	1.32	62.4	1.00	92.7	1.49	91.6	1.47	73.8	1.19	70.5	1.13	64.1	1.03
P35527	Keratin, type	3567.9	4828.9	1.35	4184.2	1.17	4681.3	1.31	4297.7	1.20	4160.6	1.17	3806.3	1.07	3832.4	1.07
A8KAJ3	cDNA FLJ7	1343.2	1837.5	1.37	1648.2	1.23	1889.4	1.41	1779.4	1.32	1797	1.34	1394.9	1.04	1262.5	0.94
Q9UHG3	Prenylcystein	1396.4	1623	1.16	1448	1.04	1676.7	1.20	1553.9	1.11	1683.2	1.21	1543.2	1.11	1253.5	0.90
P10720	Platelet facto															
S6B2A1	IgG L chain	15.4	16.1	1.05	15.7	1.02	18.2	1.18	14.9	0.97	14	0.91	20.7	1.34	13.1	0.85
B0YIW2	Apolipoprote	15931.1	15946.8	1.00	14299.1	0.90	16740.7	1.05	12479.1	0.78	14347.2	0.90	18360.1	1.15	14244.5	0.89
Q13790	Apolipoprote	2324.8	2383.7	1.03	2210.3	0.95	2269.3	0.98	2033.8	0.87	2229.3	0.96	2090	0.90	1990.1	0.86
P35908	Keratin, type	701.5	1259.7	1.80	955	1.36	943.1	1.34	1154.5	1.65	1302.1	1.86	818.6	1.17	752.4	1.07
A0A024RD	Lymphocyte	1247.4	1464.3	1.17	1267.8	1.02	1430.2	1.15	1427	1.14	1398	1.12	1284.8	1.03	1092.9	0.88
P40197	Platelet glyc	882	992.6	1.13	1028.8	1.17	1166.6	1.32	1082.7	1.23	1030.5	1.17	1056.7	1.20	824.5	0.93
P16070	CD44 antige	3109.9	3521.4	1.13	3226.7	1.04	3652	1.17	3836.3	1.23	3838	1.23	3254.7	1.05	2930.4	0.94
A0A024R93	Proteoglycan	3044.2	4220.7	1.39	3217.9	1.06	3556.5	1.17	3585.4	1.18	3689.6	1.21	3751.3	1.23	3316.8	1.09
A2KBC2	Anti-(ED-B)	1576.5	1521.9	0.97	1577	1.00	1624.3	1.03	1472.1	0.93	1518.2	0.96	1839.6	1.17	1784.3	1.13
A5YAK2	Apolipoprote	3983.5	4272.5	1.07	3741.9	0.94	4575.6	1.15	4034	1.01	4428.9	1.11	4371	1.10	4010.6	1.01
G3V2W1	Protein Z-de	1874.1	2292.1	1.22	2045.7	1.09	2003.8	1.07	1918.6	1.02	2069.7	1.10	2209.2	1.18	2075.1	1.11

A0A140VK	Testicular se	2477.8	2812	1.13	2487.7	1.00	2736.8	1.10	2375.6	0.96	2635.4	1.06	2525.6	1.02	2230.7	0.90
O43866	CD5 antigen	1243.5	1335.5	1.07	1384.5	1.11	1606.8	1.29	1391.1	1.12	1358.8	1.09	1626.1	1.31	1351.8	1.09
P01591	Immunoglob	3550.8	3113	0.88	3322.8	0.94	3143.9	0.89	3267.3	0.92	3614.4	1.02	3594.7	1.01	3742.6	1.05
A8K3I0	cDNA FLJ7	549.7	652.6	1.19	540.7	0.98	621.8	1.13	590.1	1.07	595.1	1.08	527.4	0.96	506.8	0.92
Q9H804	cDNA FLJ1	156.4	188.7	1.21	150.8	0.96	254.6	1.63	222.8	1.42	105.8	0.68	138.6	0.89	212.9	1.36
B0YJC6	Vitamin K-d	933.3	1103.8	1.18	1108.9	1.19	1160.9	1.24	1103.9	1.18	913.5	0.98	890.5	0.95	832.8	0.89
Q6UXB8	Peptidase in	818.1	905.3	1.11	840.5	1.03	932.9	1.14	822	1.00	835.3	1.02	645.3	0.79	631.8	0.77
B3KWB5	cDNA FLJ4															
P08709	Coagulation	328	407.9	1.24	288.2	0.88	410	1.25	429.3	1.31	361	1.10	314.7	0.96	263.8	0.80
V9HWB4	Epididymis s	597.3	779.4	1.30	658	1.10	736.6	1.23	713.6	1.19	670.1	1.12	707.2	1.18	577.6	0.97
P02741	C-reactive p	1082.9	830.7	0.77	1080.4	1.00	1074.1	0.99	1604.3	1.48	1017.9	0.94	930.1	0.86	4798.1	4.43
Q9UL72	Myosin-reac	364.5	481.2	1.32	387	1.06	447.7	1.23	412.4	1.13	424.7	1.17	408.7	1.12	401.4	1.10
P49908	Selenoprotei	3441.9	3795	1.10	3274.3	0.95	3369.5	0.98	3224.2	0.94	3638.3	1.06	3596.3	1.04	2561.1	0.74
V9HW12	Epididymis s	1538.3	1592.7	1.04	1603.5	1.04	1412.8	0.92	1548.9	1.01	1461.7	0.95	1574.2	1.02	1641.4	1.07
P07359	Platelet glyc	2084.5	2648.4	1.27	2443.9	1.17	2770	1.33	2679.9	1.29	2718.9	1.30	2386.6	1.14	2117.4	1.02
D3DQH8	Secreted pro	2098	2082.9	0.99	1926.3	0.92	2272.4	1.08	2198	1.05	1998.8	0.95	1964.4	0.94	1970.9	0.94
P08779	Keratin, type	1452.8	1891.4	1.30	1570.9	1.08	1774.5	1.22	1739.5	1.20	1762.6	1.21	1580.2	1.09	1378	0.95
P61626	Lysozyme C	1103.7	1304.7	1.18	1215.2	1.10	1329.5	1.20	1310.4	1.19	1321.3	1.20	1230.1	1.11	1218.8	1.10
A0A024R8Z	L-selectin O	1228.5	1334.2	1.09	1161.3	0.95	1309.8	1.07	1228.2	1.00	1343.6	1.09	1204.4	0.98	1086.6	0.88
S6BAQ4	IgG H chain															
Q59E93	Membrane a	406	515.6	1.27	400.2	0.99	502.8	1.24	474.2	1.17	465	1.15	389	0.96	363.5	0.90
A0A193CH	10E8 heavy	157.8	158.8	1.01	163.2	1.03	155.1	0.98	171.8	1.09	181.3	1.15	163	1.03	182.6	1.16
Q15166	Serum parac	355.2	388.4	1.09	362.1	1.02	443.9	1.25	424	1.19	404.1	1.14	387.6	1.09	306.9	0.86
B2RA39	cDNA, FLJ9	781.4	981	1.26	877	1.12	968.8	1.24	973.7	1.25	916.8	1.17	847.9	1.09	950.7	1.22
P0DJI9	Serum amyl	294.1	332.3	1.13	257.1	0.87	325.2	1.11	329	1.12	287.9	0.98	283.8	0.96	467.6	1.59
P0DJI8	Serum amyl	389.3	384.7	0.99	312.6	0.80	424.2	1.09	400.2	1.03	334.5	0.86	316.6	0.81	683.4	1.76
A0A068LK	Ig heavy cha															
Q15582	Transformin	1222.9	1217.3	1.00	1181.9	0.97	1282.9	1.05	1272.7	1.04	1157.6	0.95	1193.1	0.98	1078.2	0.88
A0A024R8	Prostaglandi	727.8	868.5	1.19	780.4	1.07	887.8	1.22	877.6	1.21	863.8	1.19	666	0.92	610.6	0.84
O00187	Mannan-bin	1989	2314.3	1.16	2064	1.04	2289	1.15	2051	1.03	2010.4	1.01	1745.3	0.88	1793.4	0.90
Q65ZC9	Single-chain															
P18065	Insulin-like	301.5	411.4	1.36	349.1	1.16	408.5	1.35	403.6	1.34	332.6	1.10	324.6	1.08	270.7	0.90
A0A140TA	Tenascin-X	362.7	402.7	1.11	380.1	1.05	427.2	1.18	385.6	1.06	348.8	0.96	371.3	1.02	319.7	0.88
P18428	Lipopolysac	996.7	1117.8	1.12	1038.8	1.04	1148.7	1.15	1059.3	1.06	991.4	0.99	1082.1	1.09	1446.9	1.45
A0A1B1CY	Vitamin D b	10424.2	10328.3	0.99	9334.3	0.90	10642.8	1.02	10623.2	1.02	7147.6	0.69	8648.4	0.83	3808.9	0.37
A2NYQ9	Anti-folate b	53.9	45.6	0.85	42	0.78	53.7	1.00	41.3	0.77	43.9	0.81	53.3	0.99	44.4	0.82
Q9Y5Y7	Lymphatic v	1364.9	1481	1.09	1276.5	0.94	1382.1	1.01	1324.6	0.97	1456	1.07	1268.8	0.93	964.9	0.71
P02671	Fibrinogen a	771.5	989.7	1.28	850.9	1.10	726.5	0.94	790.5	1.02	773.6	1.00	947.4	1.23	939.9	1.22
P02533	Keratin, type	55.3	86.1	1.56	62.7	1.13	80	1.45	78.1	1.41	73.8	1.33	79	1.43	51	0.92
Q6EMK4	Vasorin OS=	363.3	507	1.40	411	1.13	474.9	1.31	446.2	1.23	485.5	1.34	398	1.10	338	0.93
A0A0S2Z42	HCG203981	406.5	557.3	1.37	425.9	1.05	473.6	1.17	446.2	1.10	492.1	1.21	391.6	0.96	358.9	0.88
B7Z4R8	cDNA FLJ5															
B3KUE5	Phospholipi	901.5	1026.7	1.14	1001.6	1.11	1132.5	1.26	1000.6	1.11	1019.8	1.13	992.1	1.10	662.1	0.73
A2NB46	Cold aggluti	397.6	438.5	1.10	393.2	0.99	457.3	1.15	431.9	1.09	398.7	1.00	419.1	1.05	400.9	1.01
B2R773	cDNA, FLJ9	546.2	835.1	1.53	1129.4	2.07	751.6	1.38	746	1.37	632.2	1.16	663.9	1.22	539.3	0.99
A8K6K4	cDNA FLJ7	1308	1248.6	0.95	1077.3	0.82	1346	1.03	1536.6	1.17	1482.1	1.13	1344.5	1.03	1075.5	0.82
A0A087WX	Cadherin-1	167.8	177.2	1.06	154.2	0.92	206.2	1.23	194.4	1.16	167	1.00	155.5	0.93	132.6	0.79

A8K6A6	cDNA FLJ7	640.8	759.3	1.18	704	1.10	749.6	1.17	701.3	1.09	759.5	1.19	878.3	1.37	610.8	0.95
B3KQF4	cDNA FLJ9	166.7	222.9	1.34	170.1	1.02	180	1.08	186.7	1.12	152.8	0.92	165.9	1.00	169.3	1.02
A8K430	Fructose-bis	111.1	159.5	1.44	123.1	1.11	169.1	1.52	169.2	1.52	125.7	1.13	152.2	1.37	103.7	0.93
A0A0K0K1	Cystatin OS	801.8	842.1	1.05	825.4	1.03	805.6	1.00	821	1.02	888.1	1.11	813	1.01	778.2	0.97
A8K335	cDNA FLJ7	626.5	727.5	1.16	654.2	1.04	707.8	1.13	763.3	1.22	715.1	1.14	602	0.96	560	0.89
Q9NQ79	Cartilage aci	792.2	908.4	1.15	874.4	1.10	1227.9	1.55	829.9	1.05	874.3	1.10	746.8	0.94	679.4	0.86
Q15485	Ficolin-2 OS	1232.5	1636.6	1.33	1319.3	1.07	1375.2	1.12	1231.1	1.00	1469.1	1.19	1458.5	1.18	1153.2	0.94
B4E0X1	Beta-2-micro	1181.8	1324.5	1.12	1277	1.08	1268.4	1.07	1283.3	1.09	1219.7	1.03	1453.9	1.23	1226.4	1.04
P05362	Intercellular	202.9	238.6	1.18	210.4	1.04	221.3	1.09	230.1	1.13	208.3	1.03	214.7	1.06	218.3	1.08
Q76LX8	A disintegrin	311.2	543.8	1.75	327.7	1.05	425.6	1.37	395.9	1.27	310.8	1.00	349.8	1.12	260.2	0.84
A0A075B7I	Protein IGH															
P21333	Filamin-A C	123.6	169.3	1.37	148	1.20	168.1	1.36	169.7	1.37	144.1	1.17	141.4	1.14	110.8	0.90
Q92496	Complement	1422.9	1447.6	1.02	1316	0.92	1489.3	1.05	1467.8	1.03	1465.4	1.03	1237.4	0.87	1561.3	1.10
A0A024RD	Phospholipa	431.5	565	1.31	425.7	0.99	544.9	1.26	555.5	1.29	541	1.25	451.2	1.05	424.7	0.98
Q86VB7	Scavenger re	167.3	208.9	1.25	181	1.08	222.9	1.33	208.4	1.25	189.1	1.13	157.1	0.94	166.1	0.99
P07737	Profilin-1 O	247.6	387.8	1.57	353.3	1.43	350.2	1.41	331.5	1.34	356.3	1.44	337.3	1.36	253.1	1.02
P37802	Transgelin-2	445.5	690.4	1.55	625.6	1.40	751.4	1.69	676.2	1.52	673.6	1.51	605.5	1.36	526.5	1.18
Q13103	Secreted pho	286.8	285.6	1.00	310.5	1.08	260.6	0.91	242.5	0.85	241.1	0.84	256.7	0.90	215.2	0.75
B1AHL2	Fibulin-1 OS	181.8	239.3	1.32	197.7	1.09	215.8	1.19	222.6	1.22	261.5	1.44	166	0.91	171.8	0.94
Q59EA3	Cadherin 5,	314	558.2	1.78	497.9	1.59	596.1	1.90	503.1	1.60	496.3	1.58	329.5	1.05	293.5	0.93
Q0IIN1	Keratin 77 C															
Q16853	Membrane p	99.9	129.3	1.29	94.8	0.95	137.7	1.38	148	1.48	126.3	1.26	94.6	0.95	76.7	0.77
P02745	Complement	2193.5	2073.6	0.95	2290.7	1.04	2089.9	0.95	2080.8	0.95	2050.6	0.93	2014.3	0.92	2287.5	1.04
A0N5G5	Rheumatoid	94.5	136.1	1.44	97.1	1.03	132.6	1.40	124.5	1.32	110.3	1.17	101.2	1.07	108.4	1.15
Q9HCC1	Single chain	19	31.1	1.64	15.9	0.84	22.6	1.19	19.8	1.04	18.6	0.98	20.8	1.09	18.8	0.99
Q9Y490	Talin-1 OS=															
P33908	Mannosyl-ol	296.9	388.2	1.31	349.4	1.18	419.1	1.41	421.2	1.42	385.4	1.30	302.6	1.02	327	1.10
A8K6C9	cDNA FLJ7	934.1	1228.8	1.32	985.8	1.06	1209.8	1.30	986.1	1.06	1181.6	1.26	934.7	1.00	818.7	0.88
A0A087WT	Neural cell a	888.9	1126.6	1.27	994.6	1.12	1094.7	1.23	1013.7	1.14	997.6	1.12	1054.1	1.19	759.1	0.85
V9H1C1	Gelsolin exo															
Q9HDC9	Adipocyte p	668.2	696.8	1.04	716.8	1.07	782.3	1.17	793.5	1.19	763.3	1.14	642.7	0.96	620.9	0.93
A0A068LL	Ig heavy cha	59.3	55.8	0.94	60.9	1.03	56.5	0.95	48	0.81	77.1	1.30	57.8	0.97	80.2	1.35
O00533	Neural cell a	308.4	401.8	1.30	325.1	1.05	460.4	1.49	348.5	1.13	365.9	1.19	330.3	1.07	276.8	0.90
P35443	Thrombospo	35.7	47.3	1.32	37.8	1.06	54.5	1.53	40.4	1.13	45.5	1.27	37.1	1.04	36.5	1.02
P23470	Receptor-typ	147.9	180.8	1.22	144	0.97	201.5	1.36	200.5	1.36	186.1	1.26	134.1	0.91	104.6	0.71
E9PR17	CD59 glycop	192.7	191.7	0.99	175.9	0.91	212	1.10	214.1	1.11	197.9	1.03	177	0.92	156.6	0.81
B2R9V7	Superoxide	103	123.8	1.20	106.3	1.03	144.8	1.41	116.1	1.13	109.3	1.06	109.1	1.06	73.1	0.71
A0A125QY	GCT-A9 ligh	100.7	97.4	0.97	98.7	0.98	124.6	1.24	111.1	1.10	94.1	0.93	115.4	1.15	270.5	2.69
V9HW31	ATP synthas	83.6	149.2	1.78	140.3	1.68	154.3	1.85	152	1.82	138.6	1.66	89.4	1.07	122.9	1.47
M9MML0	Fc of IgG lov	525.7	649.1	1.23	555.7	1.06	596.8	1.14	601.8	1.14	549.8	1.05	534.9	1.02	510.7	0.97
Q13201	Multimerin-	484.6	578.8	1.19	527.2	1.09	603.9	1.25	553.1	1.14	542.8	1.12	563.8	1.16	501.9	1.04
P05164	Myeloperoxi	150.6	216.2	1.44	219.4	1.46	213.8	1.42	211.2	1.40	238.4	1.58	216	1.43	165.9	1.10
B2R7D2	cDNA, FLJ9	180	201.9	1.12	179.8	1.00	203.8	1.13	180.4	1.00	175.6	0.98	167.6	0.93	145.5	0.81
G9K388	YWHAE/FA	139.1	382.4	2.75	307.1	2.21	367.6	2.64	384.3	2.76	322.6	2.32	149.6	1.08	340.3	2.45
A0A125QY	IBM-B2 hea	338.5	351.7	1.04	286.3	0.85	377.4	1.11	331.4	0.98	326.2	0.96	328.2	0.97	296.8	0.88
A0A125QY	GCT-A7 hea															
Q8NBP7	Proprotein c	138.5	190.5	1.38	139.5	1.01	186.7	1.35	178	1.29	184.5	1.33	130.9	0.95	92.9	0.67

A4D2D2	Procollagen	134.5	157.4	1.17	139.2	1.03	137.5	1.02	118.9	0.88	136	1.01	122.5	0.91	96.7	0.72
P07195	L-lactate deh	187.3	219	1.17	207.3	1.11	205.5	1.10	176	0.94	197.2	1.05	192.4	1.03	191.6	1.02
E7EQ64	Trypsin-1 O	329.5	485.9	1.47	434	1.32	340.8	1.03	333.8	1.01	442.1	1.34	322.1	0.98	229.1	0.70
Q9UL85	Myosin-reac	1213.3	1238.1	1.02	1267.8	1.04	1233.3	1.02	1110.3	0.92	1252.6	1.03	1142.2	0.94	1319.2	1.09
P19320	Vascular cel	119.6	165.9	1.39	124.6	1.04	135.8	1.14	129.9	1.09	127.7	1.07	132.7	1.11	109.2	0.91
A0A140VJF	Testicular tis	106.7	133.4	1.25	108.5	1.02	133.7	1.25	124.3	1.16	118.2	1.11	114.2	1.07	84.4	0.79
P05019	Insulin-like g															
Q15430	Coagulation															
A2J1N5	Rheumatoid	112.9	128.6	1.14	126.1	1.12	218.9	1.94	147	1.30	127.4	1.13	123.3	1.09	107.3	0.95
Q07954	Prolow-dens	22.3	23.3	1.04	22.5	1.01	29.5	1.32	30	1.35	27.7	1.24	25.7	1.15	31.1	1.39
P24592	Insulin-like g	120.4	139.1	1.16	119	0.99	147.8	1.23	160	1.33	139.5	1.16	141	1.17	102.9	0.85
B2RBF5	cDNA, FLJ9	95.9	110	1.15	98.6	1.03	119.6	1.25	98.5	1.03	117	1.22	103.3	1.08	121.3	1.26
P12111	Collagen alp	83.9	112.8	1.34	84.9	1.01	109	1.30	100.6	1.20	97.6	1.16	87.7	1.05	70.1	0.84
B7Z9B1	cDNA FLJ5	183.5	213	1.16	182	0.99	223.4	1.22	245.4	1.34	200.6	1.09	175	0.95	158.9	0.87
B2R4R0	Histone H4 (	266.4	875.9	3.29	676.1	2.54	749.1	2.81	753.8	2.83	704.1	2.64	320.6	1.20	564.9	2.12
A8K6C1	cDNA FLJ7	679.2	730.8	1.08	691	1.02	887	1.31	885.6	1.30	756.5	1.11	766.5	1.13	666.3	0.98
A0N5G1	Rheumatoid	236.4	293.3	1.24	244.9	1.04	279.1	1.18	241.5	1.02	264.9	1.12	263.1	1.11	246.1	1.04
X6RBG4	Uromodulin	174.1	213.9	1.23	141	0.81	221.9	1.27	221.6	1.27	205.7	1.18	147.3	0.85	149.8	0.86
P49746	Thrombospo	626.2	765.2	1.22	612.7	0.98	777.6	1.24	651.6	1.04	854.1	1.36	638.2	1.02	558.4	0.89
A0A0X9UW	MS-D4 heav	644.4	710.5	1.10	592.1	0.92	723.2	1.12	664	1.03	740.1	1.15	668.3	1.04	688.6	1.07
P00918	Carbonic anh	122.8	163.1	1.33	160	1.30	141.6	1.15	152	1.24	162.7	1.32	170	1.38	149.8	1.22
P08253	72 kDa type															
J3KPA1	Cysteine-rich	1673.4	2029.3	1.21	1726.2	1.03	1542.2	0.92	1556.9	0.93	1752.8	1.05	1671	1.00	1279.2	0.76
P22692	Insulin-like g															
Q4ZG40	Macrophage	171.9	200.9	1.17	143.7	0.84	187.4	1.09	181.2	1.05	159.4	0.93	157.2	0.91	131.7	0.77
E2RVJ0	Anion excha	23.2	45.7	1.97	41.6	1.79	51.8	2.23	36.3	1.56	32.8	1.41	41.9	1.81	22.8	0.98
A6NC48	ADP-ribosyl	236.2	275.1	1.16	235.9	1.00	310.2	1.31	251.1	1.06	259.8	1.10	236.7	1.00	225.3	0.95
Q6MZL2	Putative unc	569.9	701.4	1.23	643.4	1.13	639.9	1.12	593	1.04	599.9	1.05	603.8	1.06	543.8	0.95
P04040	Catalase OS	182.4	325.4	1.78	234.3	1.28	271	1.49	208.5	1.14	178	0.98	199.2	1.09	194.1	1.06
D3YTG3	Target of Ne	153.2	157.3	1.03	163.3	1.07	221.3	1.44	176.3	1.15	176	1.15	133.7	0.87	123.8	0.81
Q9UL89	Myosin-reac	74.9	83.3	1.11	66	0.88	98.2	1.31	70.9	0.95	86	1.15	68.2	0.91	64.1	0.86
X6R8F3	Neutrophil g	264.9	369.7	1.40	353.7	1.34	377.1	1.42	359.3	1.36	354	1.34	316.2	1.19	289.9	1.09
A0A024RD	SPARC-like	67.4	108.7	1.61	78	1.16	88.3	1.31	113.9	1.69	86.9	1.29	81.4	1.21	62.4	0.93
P35916	Vascular end	44.5	45.6	1.02	35.5	0.80	48.1	1.08	50.6	1.14	34.5	0.78	40.7	0.91	31.2	0.70
B7ZMD7	Alpha-amyla	35.3	56.2	1.59	35.9	1.02	53.3	1.51	50.1	1.42	44	1.25	49	1.39	42.1	1.19
J3KPS3	Fructose-bis	133.9	197.8	1.48	164.3	1.23	206.9	1.55	205.3	1.53	173.7	1.30	144.9	1.08	133.8	1.00
Q14126	Desmoglein-															
P23284	Peptidyl-pro															
Q8WZ75	Roundabout	152.6	221.9	1.45	170.2	1.12	207.2	1.36	220.8	1.45	185.4	1.21	179.8	1.18	158.2	1.04
A0A087X1	Calcium-dep	298	326.4	1.10	282.7	0.95	391.4	1.31	337.5	1.13	297.2	1.00	291.3	0.98	284.7	0.96
A0A0B4J1Y	Immunoglob															
Q86YW5	Trem-like tra	115.4	143.9	1.25	116.3	1.01	143.2	1.24	132.4	1.15	126.9	1.10	121.4	1.05	99.9	0.87
A0A024R9J	Collectin sub															
Q6UY14	ADAMTS-li	42.9	62.8	1.46	54.2	1.26	65.4	1.52	56.2	1.31	53.3	1.24	47	1.10	39.6	0.92
Q6UX71	Plexin doma	179.8	226.6	1.26	197.4	1.10	227.5	1.27	181.4	1.01	199.7	1.11	211.7	1.18	150.5	0.84
P03950	Angiogenin	363.2	420	1.16	390.8	1.08	489.1	1.35	518.6	1.43	472.9	1.30	389.7	1.07	343.2	0.94
Q05639	Elongation f	40.3	127.6	3.17	111.4	2.76	84.2	2.09	85.3	2.12	136.9	3.40	56.2	1.39	104.1	2.58

Q59ED3	Intercellular	68.1	87.6	1.29	66	0.97	99.7	1.46	102.8	1.51	70	1.03	66	0.97	56.5	0.83
Q8NGE6	Olfactory rec	1201.7	1274.4	1.06	1087.1	0.90	1695.4	1.41	1970.5	1.64	1579.6	1.31	898.4	0.75	1144.2	0.95
P07333	Macrophage	32.6	52.1	1.60	37.1	1.14	42.2	1.29	44.6	1.37	33.9	1.04	37.4	1.15	31.8	0.98
Q9H8L6	Multimerin-2	160.8	227.9	1.42	155.2	0.97	220	1.37	188.5	1.17	186.5	1.16	178.5	1.11	142.2	0.88
P04066	Tissue alpha	124.2	152.2	1.23	118.8	0.96	179.1	1.44	166.6	1.34	134.4	1.08	125	1.01	104	0.84
A8K7T4	cDNA FLJ7															
A0A140VJC	Testicular tis	134.5	139.9	1.04	137	1.02	167.7	1.25	161.9	1.20	160.1	1.19	141.1	1.05	137.8	1.02
A0A1C9J6F	B cell recept	61.6	87.8	1.43	61.3	1.00	96.2	1.56	87.2	1.42	86.2	1.40	79	1.28	58.1	0.94
B4DZM1	cDNA FLJ5	1025.2	1136	1.11	998.7	0.97	907.2	0.88	924.7	0.90	1086.3	1.06	1158.3	1.13	1028.7	1.00
A0A024R6Y	Dipeptidase															
Q4LE64	NUMA1 var	802.5	1029.2	1.28	821	1.02	1000.5	1.25	1075	1.34	1117.1	1.39	684	0.85	571.4	0.71
P11279	Lysosome-as	238.8	315.2	1.32	278.2	1.16	311.7	1.31	266.4	1.12	250.5	1.05	229.6	0.96	221.9	0.93
Q99784	Noelin OS=I	236.4	230.4	0.97	224.4	0.95	214.6	0.91	214.5	0.91	211.6	0.90	203.3	0.86	219.5	0.93
Q9UIU0	Dihydropyri	64.3	86.9	1.35	72.3	1.12	95.2	1.48	65.3	1.02	68.7	1.07	75.9	1.18	56.5	0.88
P07998	Ribonucleas	34.2	36.7	1.07	32	0.94	38.4	1.12	41.7	1.22	42.1	1.23	36.3	1.06	29.5	0.86
K4DIA0	ICOS ligand	522.3	570.7	1.09	574.2	1.10	552.6	1.06	517.5	0.99	534.7	1.02	498.4	0.95	476.9	0.91
Q9ULV0	Unconventio	53.7	63.5	1.18	46.6	0.87	53.9	1.00	82.8	1.54	56	1.04	64.5	1.20	84.6	1.58
O14786	Neuropilin-1															
B7Z3G0	cDNA FLJ5	295.9	219.8	0.74	169.4	0.57	259.1	0.88	320.3	1.08	272.7	0.92	313.1	1.06	191.9	0.65
Q99969	Retinoic aci	40.7	55.7	1.37	50.7	1.25	68.1	1.67	56.8	1.40	50.2	1.23	45.7	1.12	42.4	1.04
Q9HCI6	E3 SUMO-p	247.6	303.5	1.23	614.6	2.48	350.2	1.41	420.3	1.70	379.6	1.53	224.3	0.91	259.3	1.05
A0A087WV	Receptor-typ	58	75	1.29	66.9	1.15	70.1	1.21	55	0.95	58.1	1.00	70	1.21	55	0.95
P67936	Tropomyosin	328.1	474	1.44	453.2	1.38	532.2	1.62	517.2	1.58	458.3	1.40	433.9	1.32	343.7	1.05
A0A024RC	Peroxisome	446.8	477.6	1.07	483.8	1.08	540.7	1.21	509.1	1.14	603.2	1.35	356.8	0.80	456.9	1.02
P98160	Basement m	91.9	180.2	1.96	126.8	1.38	126.6	1.38	132.4	1.44	138.4	1.51	159.1	1.73	95.7	1.04
A0A0S4XQ	MHC Class															
Q8IY21	Probable AT	795	976.1	1.23	858.9	1.08	1005.7	1.27	930.4	1.17	1047.1	1.32	730.4	0.92	754.9	0.95
A0A0U1RR	Histone H2A	218.4	257.8	1.18	229.1	1.05	272.6	1.25	266.6	1.22	263.5	1.21	232.6	1.07	186	0.85
G3XAI2	Laminin sub	99.6	118.5	1.19	108.6	1.09	150.7	1.51	133.7	1.34	113.4	1.14	107.6	1.08	80.6	0.81
C9JC84	Fibrinogen g															
Q9H7P6	Multivesicul															
B7Z351	Secreted pho	40.3	47.7	1.18	36.1	0.90	40.2	1.00	46.4	1.15	41.8	1.04	46.3	1.15	34.7	0.86
Q9BWP8	Collectin-11	18.3	36	1.97	28.1	1.54	40.2	2.20	40.5	2.21	40.3	2.20	21.1	1.15	16.9	0.92
A0A024QZ	Serine/threo	359.7	297.4	0.83	286.2	0.80	380.1	1.06	538.4	1.50	430	1.20	246.8	0.69	223.6	0.62
A0A024QZ	Proteoglycan	132.4	161.4	1.22	127.2	0.96	176.4	1.33	128.5	0.97	136.2	1.03	156.7	1.18	108.9	0.82
A8K9X5	cDNA FLJ7	1282.7	1183.1	0.92	1057.9	0.82	1665	1.30	1951.7	1.52	1903.3	1.48	2038.2	1.59	880.6	0.69
P11717	Cation-indep															
A0A024R2V	Dystroglycan															
D6W5L6	Pulmonary s	27.1	35.8	1.32	23.6	0.87	32.4	1.20	34.2	1.26	28.6	1.06	30.3	1.12	38.6	1.42
P20023	Complement															
A0A060VC	MHC class I	1005.6	990.7	0.99	1154.2	1.15	874.8	0.87	858.5	0.85	929.5	0.92	1310.8	1.30	899.2	0.89
A8K2T7	Receptor pro															
A0A024R10	Contactin 1,	41.9	50	1.19	47.3	1.13	56.2	1.34	40.4	0.96	46.9	1.12	43.8	1.05	29.5	0.70
Q9BTY2	Plasma alpha	237.3	282.1	1.19	242.6	1.02	252.4	1.06	280.7	1.18	273.7	1.15	242.1	1.02	220.1	0.93
Q8IXL6	Extracellular	121.2	88.7	0.73	82.9	0.68	112.4	0.93	78.9	0.65	103.9	0.86	92.9	0.77	48.6	0.40
A0A075B6F	Dynein heav	130.9	147.4	1.13	89.2	0.68	150.5	1.15	147.8	1.13	144.5	1.10	106.1	0.81	77	0.59
A0A087WX	IgGFc-bindi	260.4	310.1	1.19	245.6	0.94	327.3	1.26	268	1.03	250	0.96	265.7	1.02	235.5	0.90

Q99727	Metalloprote	135	93.4	0.69	109.7	0.81	130.5	0.97	142.1	1.05	111.3	0.82	116.6	0.86	118.5	0.88
Q0ZCH6	Immunglobu	71.2	85.4	1.20	69.3	0.97	100	1.40	97.9	1.38	77.4	1.09	76.1	1.07	67.1	0.94
Q9UNN8	Endothelial	148.8	146.2	0.98	153.2	1.03	181.9	1.22	174.8	1.17	162.8	1.09	146.9	0.99	150.4	1.01
A0A0C4DG	Poliovirus re	336.2	336	1.00	313.3	0.93	359.9	1.07	385.4	1.15	333.7	0.99	411	1.22	253.4	0.75
J3KNB4	Cathelicidin	316.2	351.3	1.11	340.1	1.08	421	1.33	395.6	1.25	385.8	1.22	382.4	1.21	289.6	0.92
Q96RZ2	N-acetylgluc	45	70.6	1.57	65.6	1.46	59.8	1.33	47.2	1.05	49.8	1.11	40	0.89	42.2	0.94
Q4LE33	TNC variant															
A8K486	Peptidyl-pro	9.5	24.4	2.57	19	2.00	13.1	1.38	15.1	1.59	13.1	1.38	13.8	1.45	8.2	0.86
B2R6V9	cDNA, FLJ	52.7	69.4	1.32	56.9	1.08	69.2	1.31	56.4	1.07	69.9	1.33	60.9	1.16	37.2	0.71
Q16706	Alpha-mann															
H3BQD0	Cerebellin-1	13	13.6	1.05	18.9	1.45	16.2	1.25	12	0.92	15.3	1.18	18.1	1.39	12.7	0.98
Q86U17	Serpin A11	54	74	1.37	58.2	1.08	76.4	1.41	76.2	1.41	78.9	1.46	89.4	1.66	54	1.00
A0A140VK	Leukotriene	31.4	35.1	1.12	29	0.92	31.9	1.02	27.7	0.88	33.2	1.06	24	0.76	28.4	0.90
A0A140GX	Platelet mem	40.9	62.2	1.52	51.3	1.25	81.4	1.99	44.4	1.09	39.6	0.97	41.9	1.02	35.7	0.87
Q96S96	Phosphatidy	32.3	34.8	1.08	29	0.90	43.1	1.33	35	1.08	33.3	1.03	46.8	1.45	26.7	0.83
O00151	PDZ and LI	78.2	103.2	1.32	93.2	1.19	99.7	1.27	104.3	1.33	97.3	1.24	94.3	1.21	70.4	0.90
A0A068B0	Cis-AB glyc	74.1	92.9	1.25	74.9	1.01	93.7	1.26	95.4	1.29	75.6	1.02	124.2	1.68	72.3	0.98
Q6YHK3	CD109 antig	163.1	197.5	1.21	155.1	0.95	190.1	1.17	219.3	1.34	176.1	1.08	195.4	1.20	149.5	0.92
B3KSS6	Phosphodies	385.6	316.3	0.82	291.1	0.75	329.9	0.86	343.6	0.89	295.2	0.77	417.6	1.08	321.5	0.83
B4DQY7	cDNA FLJ5	15.6	22.3	1.43	20.1	1.29	20.8	1.33	17.9	1.15	17.2	1.10	20	1.28	22.6	1.45
A0A024RD	Periostin, os															
P06733	Alpha-enola	49.7	71.8	1.44	57.6	1.16	92.1	1.85	66.6	1.34	70	1.41	62.9	1.27	52.2	1.05
Q86SQ4	Adhesion G-															
Q8TER0	Sushi, nidog															
A1L4H1	Soluble scav															
Q13740	CD166 antig	39.8	60	1.51	43.9	1.10	54.1	1.36	50.8	1.28	48.3	1.21	47	1.18	37.2	0.93
O94818	Nucleolar pr	60.9	66.8	1.10	54.2	0.89	83	1.36	70.8	1.16	68.8	1.13	56.3	0.92	44.9	0.74
P06703	Protein S10															
D3DPK5	SH3 domain	63.5	99	1.56	106.2	1.67	123.2	1.94	78.5	1.24	81.8	1.29	83.4	1.31	85	1.34
E9PK25	Cofilin-1 OS															
A0A125U0	GCT-A2 hea	18.9	24.4	1.29	19.9	1.05	30.3	1.60	26.6	1.41	20.8	1.10	26.9	1.42	15.2	0.80
D6W5K2	Thymosin, b	88	215	2.44	183.4	2.08	144.4	1.64	169.2	1.92	204.7	2.33	181.3	2.06	76.5	0.87
Q6PIY7	Poly(A) RN	67.2	60.9	0.91	63.3	0.94	61.6	0.92	56.5	0.84	52.3	0.78	61.6	0.92	43	0.64
Q96AN5	Transmembr	90	156.9	1.74	268.8	2.99	443.2	4.92	179.8	2.00	136.5	1.52	83.9	0.93	93.9	1.04
Q6P4F1	Alpha-(1,3)-															
Q9UL82	Myosin-reac															
Q7Z7M0	Multiple epi															
Q2KHR2	DNA-bindin															
Q75KY0	Putative unc															
V9HVY1	Epididymis	51.6	85.5	1.66	127.8	2.48	209.8	4.07	85.8	1.66	64.8	1.26	51.9	1.01	68	1.32
Q59EB6	Complement	49.4	66.1	1.34	46.1	0.93	55.1	1.12	58.8	1.19	50.3	1.02	63.9	1.29	59.5	1.20
Q13131	5'-AMP-acti															
Q96RR4	Calcium/cal															
A0A0R4J2	Spermatoger	22.3	78.2	3.51	53.8	2.41	56.5	2.53	70.6	3.17	68.9	3.09	36.4	1.63	52.8	2.37
P25391	Laminin sub	67.6	83.6	1.24	71.2	1.05	80.7	1.19	80.8	1.20	84.7	1.25	74	1.09	49.1	0.73
A0A0G2JN	Transcriptio															
E5RG27	Casein kinas	115.8	125	1.08	109	0.94	168.1	1.45	173.4	1.50	152.4	1.32	80	0.69	88.9	0.77

Q12841	Follistatin-re	26.6	34.7	1.30	35.6	1.34	29.1	1.09	27.7	1.04	31.4	1.18	39.1	1.47	31.4	1.18
Q92833	Protein Jumo	606.3	532.8	0.88	542.3	0.89	572.6	0.94	447.8	0.74	479.1	0.79	615.9	1.02	610.2	1.01
J3KMX3	Alpha-fetopr	61.9	292.2	4.72	88.4	1.43	153.4	2.48	117.6	1.90	104.8	1.69	75.4	1.22	46.4	0.75
R4GMU1	GDH/6PGL	47.6	63	1.32	46.1	0.97	62.3	1.31	49.4	1.04	56.1	1.18	53.7	1.13	39.6	0.83
Q14766	Latent-transf	32.2	34.6	1.07	33.9	1.05	34.6	1.07	32.3	1.00	33.2	1.03	26.6	0.83	38	1.18
A0A068LRY	Ig heavy cha	104.6	84.7	0.81	83.7	0.80	147	1.41	163.1	1.56	112.7	1.08	102.3	0.98	73.3	0.70
K7ELC7	60S ribosom															
A0A1B0GW	GRAM dom	33.8	45.3	1.34	36	1.07	32.6	0.96	36.1	1.07	41.3	1.22	36.9	1.09	46.9	1.39
V9HW26	ATP synthas	117.8	175.9	1.49	164.9	1.40	168.9	1.43	169.9	1.44	162.5	1.38	110.7	0.94	140.9	1.20
Q9H628	Ras-related a	196	209.3	1.07	167.6	0.86	194	0.99	317.1	1.62	223.3	1.14	295.1	1.51	349.6	1.78
B2RDG0	Proteasome s	67.9	143.8	2.12	116.1	1.71	156.9	2.31	166.8	2.46	127.9	1.88	113.5	1.67	120.2	1.77
Q8WWZ8	Oncoprotein	53.5	61.3	1.15	50.8	0.95	61.7	1.15	76.5	1.43	55.4	1.04	45.1	0.84	43.2	0.81
Q9Y5Z9	UbiA prenyl	81.4	94.4	1.16	89.7	1.10	109.1	1.34	111.7	1.37	112	1.38	82.5	1.01	80.9	0.99
B2R7N9	cDNA, FLJ9															
A0A024R8C	Glucosidase,	61.2	61.9	1.01	56.9	0.93	74.9	1.22	75	1.23	63.9	1.04	66.8	1.09	42	0.69
Q96JG6	Syndetin OS	264.9	269.8	1.02	285.1	1.08	225.2	0.85	172.5	0.65	241.3	0.91	264.5	1.00	189.2	0.71
Q66K66	Transmembr	522.4	650.3	1.24	714.8	1.37	829.3	1.59	821.1	1.57	623.6	1.19	527.9	1.01	380.9	0.73
A0A024R7I	DNA (cytosi															
Q6ZWJ1	Syntaxin-bir															
